# Enantioselective synthesis of configurationally stable [5]helicenes containing 1,2-azaborine units

**DOI:** 10.1039/d6sc02344d

**Published:** 2026-04-23

**Authors:** Catherine Olguin, Christian Tabacaru, Lennart Besse, Martin Simon, Christopher Golz, Marcos Humanes, Manuel A. Fernández-Rodríguez, Patricia García-García, Maike Mücke, Ricardo A. Mata, Manuel Alcarazo

**Affiliations:** a Institut für Organische und Biomolekulare Chemie, Georg-August-Universität Göttingen Tammannstr 2 Göttingen 37077 Germany manuel.alcarazo@chemie.uni-goettingen.de; b Universidad de Alcalá (IRYCIS). Departamento de Química Orgánica y Química Inorgánica, Instituto de Investigación Química “Andrés M. del Río” (IQAR) 28805 Alcalá de Henares Madrid Spain; c Institut für Physikalische Chemie, Georg-August-Universität Göttingen Tammannstr 6 Göttingen 37077 Germany

## Abstract

Two different families of BN-doped [5]helicenes have been efficiently synthesized through a highly enantioselective, intramolecular, Au-catalyzed alkyne hydroarylation reaction. Key for the success of the method is the use of BINOL-derived cationic phosphonites as ancillary ligands (BINOL: 1,1-bi-2-naphthol). The inversion barriers of the structures obtained have been determined both experimentally and theoretically, and are essentially identical to those reported for non-dopped carbo[5]helicenes of otherwise identical structure. Contrarily, the newly prepared BN-doped helicenes exhibit intensified absorption spectra at long wavelength (*λ* ≈ 400 nm) and fluorescence when compared with their only-carbon counterparts. These effects are particularly pronounced for the naphtho[2,1-*c*]phenanthro[1,2-*e*][1,2]azaborinine series, in which the BN-unit is located at the rim of the helix. Preliminary studies on the post-synthetic functionalization of these structures are also described; specifically, the naphtho[2,1-*c*]phenanthro[1,2-*e*][1,2]azaborinine structure can be site-selective brominated at position 4. In addition, the unprecedented deborilation of these helices to afford axially chiral anilines has been observed by treatment with DDQ.

## Abstract 2

Two different families of BN-doped [5]helicenes have been efficiently synthesized through a highly enantioselective, intramolecular, Au-catalyzed alkyne hydroarylation reaction.

## Graphical abstract

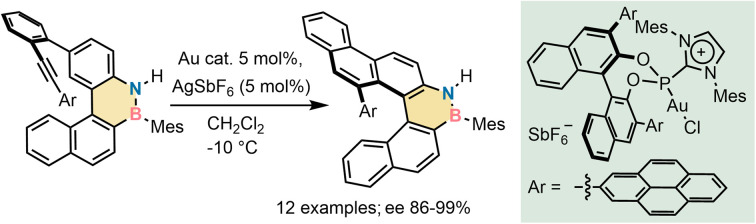

## Introduction

In polycyclic aromatic scaffolds, the formal replacement of two consecutive carbon atoms by an isoelectronic BN-unit barely modifies the geometric parameters of the original structure because the atomic radii of the three elements is quite similar, and the aromatized framework is retained.^1–5^ Yet, that exchange generates a perturbation on the electronic distribution that completely redefines the reactivity and photophysical properties of the resulting BN-doped materials.^6–8^ For example, it is well-documented that electrophilic aromatic substitution more readily occurs after formal BN-doping than in their carbon-only precursors;^9,10^ moreover, the substitution is highly regioselective and takes place at the position(s) adjacent to boron if these are unsubstituted and sterically accessible.^11–13^ Hydrogenation,^14^ photoisomerization reactions,^15^ and [4 + 2] cycloadditions with dienophiles of diverse nature are reactions that also get facilitated in 1,2-azaborinine derivatives due to the reduced aromaticity of this ring.^16,17^ An enhanced dipolar moment that affects solubility and molecular stacking is also expected by BN-doping; however, the most significant impact is often observed in the photophysical properties of the resulting materials.^18^ The asymmetry generated by the formal CC/BN-replacement deeply modifies the shapes and energetic distribution of the original frontier orbitals in a magnitude that depends upon the number of BN-units introduced,^19,20^ and their specific location within the polyaromatic scaffold.^21^ It is for this reason that BN-doping has emerged as an appealing strategy to optimize the luminescent properties of polycyclic aromatic hydrocarbon materials.^22,23^ In fact, BN-doped arenes often depict higher photoluminescence quantum yields (*φ*_PL_) than their all-carbon counterparts, making them superior components in electronic devices such as organic light emitting diodes (OLEDs),^24^ organic field-effect transistors (OFETs)^25^ or solar cells.^26^ Their use for selective fluoride anion detection has been reported as well.^27^

**Fig. 1:**
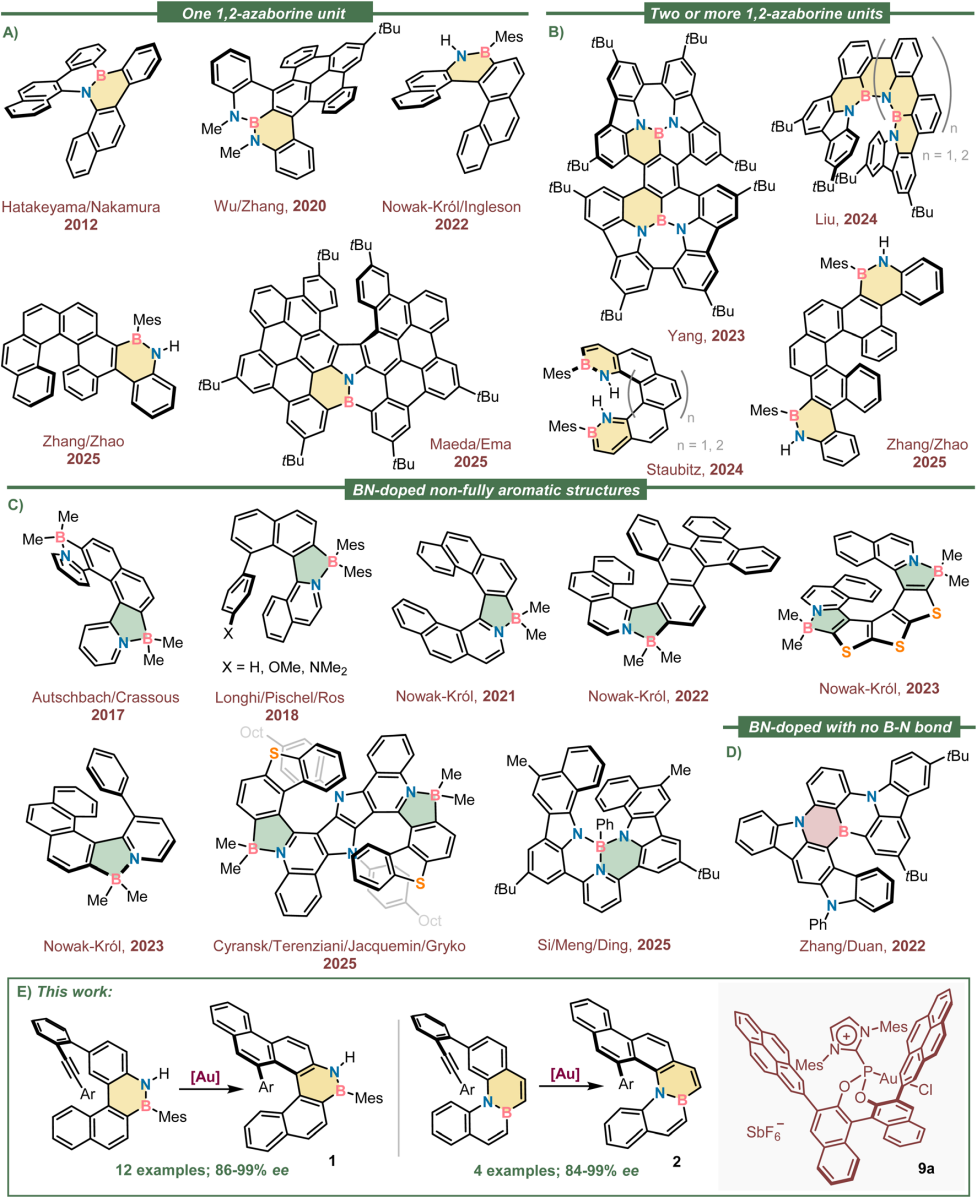
Selected structures of configurationally stable BN-doped helicenes. (A) Structures containing only one 1,2-azaborine; (B) helical scaffold with more than one 1,2-azaborine embedded; (C) 1,2-azaborole containing helicenes; (D) helicenes doped with an 1,4-azaborine unit; (E) overview of this work.

In addition, helical shape structures have been recognized as promising circularly polarized luminescence (CPL) dyes^28,29^ with potential application in diverse areas such as bioimaging,^30^ chiral switches,^31–33^ and data storage^34^ among others;^35^ however, unmodified carbohelicenes are typically characterized by low performance in terms of *φ*_PL_ and luminescence dissymmetry factors (*g*_lum_).^36,37^ It is for that reason that the installment of BN-moieties in helicenes has not gone unnoticed as a tool to fine tune their chiroptical properties without altering their shape.^38^Fig. 1A and B shows a selection of conformationally stable helicenes containing one,^39–43^ two,^42,44,45^ or more 1,2-azaborinine rings;^46^ while Fig. 1C depicts non-completely aromatic helical scaffolds in which the BN-doping unit is a 1,2-azaborole,^47–53^ and Fig. 1D shows a 1,4-diazaborinine containing helicene.

Looking at the structures shown in Fig. 1, it is surprising that with no exception they all were obtained through racemic syntheses that invariably require a final chromatographic separation of the enantiomeric constituents using a chiral stationary phase. In an attempt to address this synthetic limitation, we speculated that the enantioselective π-extension of 1,2-azaborine containing polyarenes through an intramolecular alkyne hydroarylation reaction might be the appropriate entry to enantioenriched BN-doped helicenes of different sizes and substitution patterns.^54,55^ In addition, our previous experience in the topic made us believe that Au(i)-catalysts bearing chiral α-cationic ancillary ligands^56^ are predestined to successfully achieve that transformation.^57–61^ Herein, we describe the materialization of that idea with the highly enantioselective syntheses of two different families of boraaza[5]helicenes sharing a common naphtho[2,1-*c*]chrysene scaffold (Fig. 1E). In compounds of general structure 1 the BN-unit is incorporated at the rim of the skeleton (7-,8-positions), while in 2 it is placed internally, in the bridging 8a, 14b-positions. The racemization barriers of both heterohelicenes are investigated through theoretical and experimental methods, and their photochemical characterization reported (UV-vis absorption, *φ*_PL_ and *g*_lum_ spectra). As expected, the introduction of the polar BN-moiety within the hydrocarbon framework drastically improved the *φ*_PL_ in both cases when compared with the parent naphtho[2,1-*c*]chrysene (*φ*_PL_ = 2%), albeit more significantly in 1-type structures (*φ*_PL_(1b) = 20%).

## Results and discussion

### Synthesis of precursors, catalyst optimization and scope

**Scheme 1:**
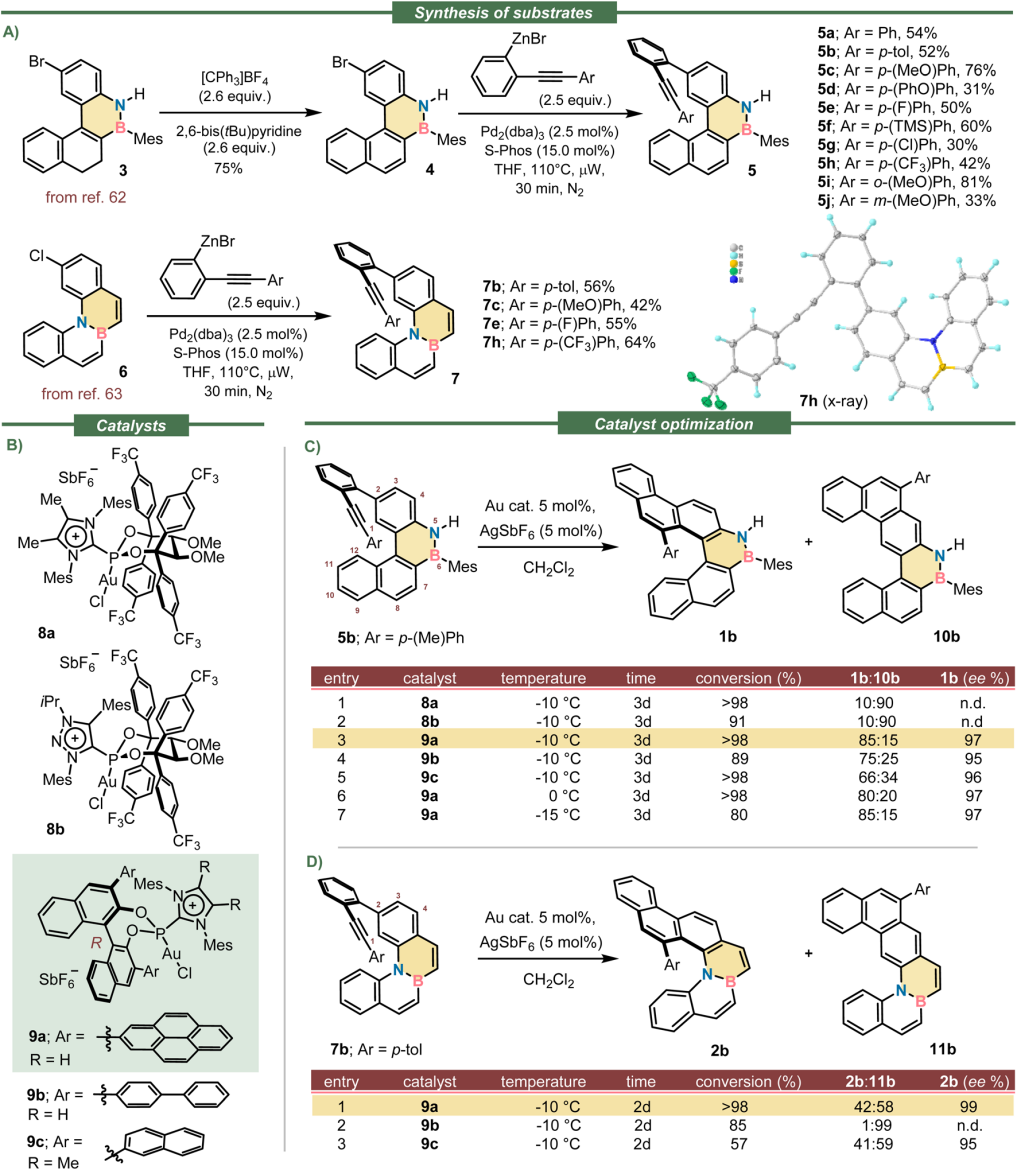
Reaction conditions and catalyst optimization. (A) Synthesis of substrates 5 and 7; (B) catalysts used for the screening; (C and D) optimization tables. Conversions and regioisomeric ratios were determined by ^1^H NMR of crude samples; ee values determined by chiral HPLC.

We initiated our studies with the synthesis of appropriate substrates for the planned enantioselective hydroarylation. Thus, alkynes 5a–j were prepared from reported bromide 3 through an initial oxidation to obtain the fully aromatized benzo[*e*]naphtho[2,1-*c*][1,2]azaborine 4,^62^ followed by the installation of the necessary alkyne moiety *via* Negishi coupling. Substrates of general formula 7 were prepared following a similar route but starting from the already aromatic precursor 6 (Scheme 1A).^63^ The experimental details regarding the syntheses and the spectroscopic characterization of all new substrates are described in the SI.

Compound 5b was used as model and initially submitted to the effect of the five Au-catalysts that, considering our previous experience, were the most promising for the key cyclization (Scheme 1B).^57–61^ Unfortunately, catalysts 8a and 8b, both sharing an acyclic version of TADDOL (tetraaryl-2,2-disubstituted-1,3-dioxolane-4,5-dimethanol), and decorated with *p*-(CF_3_)phenyl substituents, performed badly; they provided the undesired planar regioisomer 10b as the main product of the hydroarylation reaction (Scheme 1C, entries 1–2).

On the other hand, catalysts 9a–c derived from the BINOL (1,1′-bi-2-naphthol) platform demonstrated to be suitable for this reaction. They three delivered helicene 1b with excellent enantioselectivity (Scheme 1C, entries 3–5), and importantly, 9a was even able to override the natural tendency of the substrate to direct the intramolecular hydroarylation to 10b. Specifically, the use of 9a raised the regioselectivity to a remarkable 85 : 15 ratio for the azabora[5]helicene 1b while providing excellent optical purity as well (97% ee). Catalyst 9a also promoted the cyclisation of 7b into the corresponding azabora[5]helicene 2b with excellent optical purity (99% ee; Scheme 1D, entry 1); yet, the main product was the BN-doped dibenzo[*a*,*m*]tetraphene 11b. The use of catalysts 9b–c did not improve the regioselectivity of this hydroarylation step, which is highly substrate dependent.

**Fig. 2:**
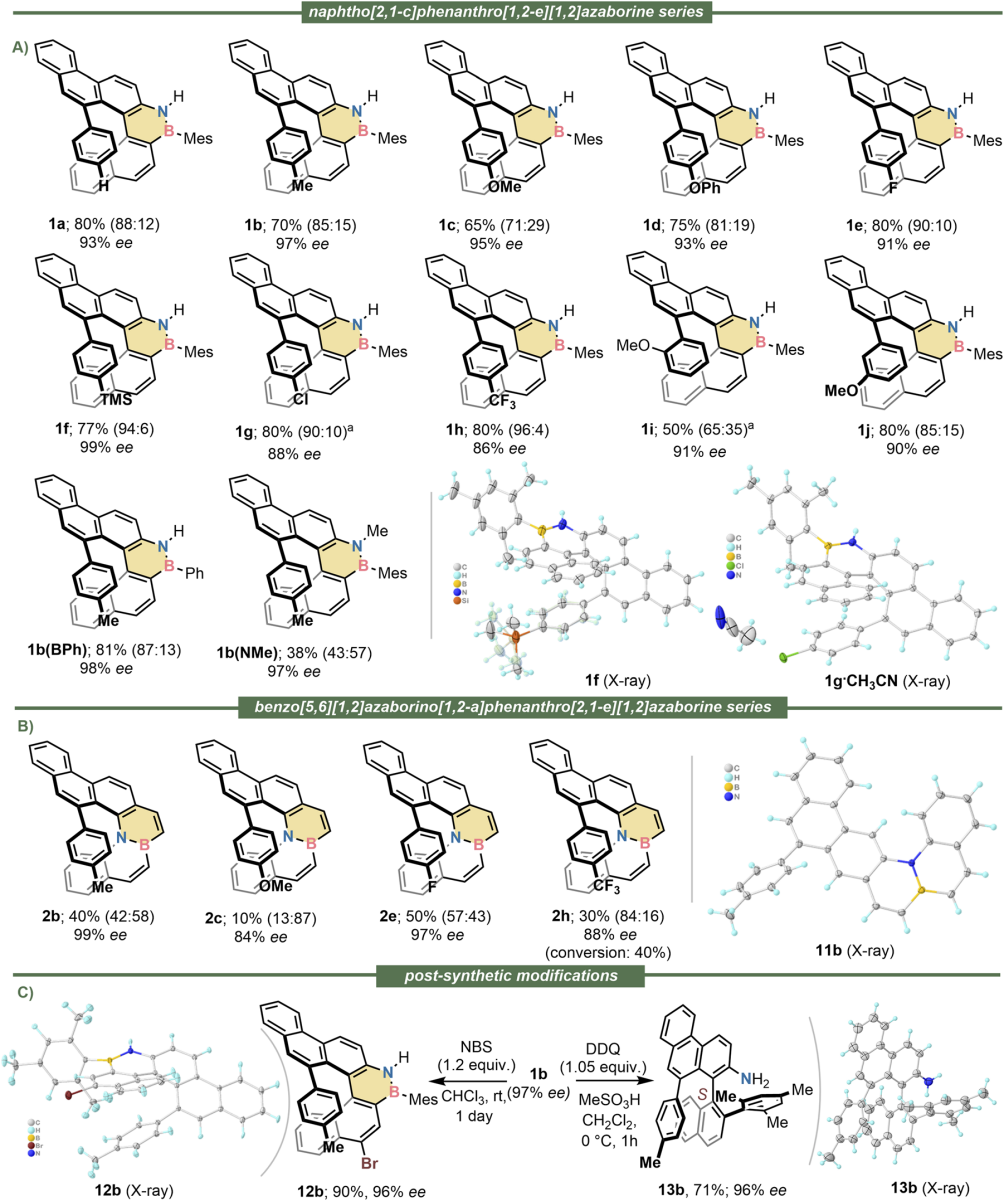
Substrate scope and limitations. (A) Scope of the naphtho[2,1-*c*]phenanthro[1,2-*e*][1,2]azaborine series; (B) scope of the benzo[5,6][1,2]azaborino[1,2-*a*]phenanthro[2,1-*e*][1,2]azaborine series; (C) post-synthetic modifications. All yields are of the isolated helical products after complete consumption of the starting materials. Regioisomeric ratios determined by ^1^H NMR of the crude reaction mixtures are shown in parenthesis; the first value corresponds to the helical component. ee values determined by chiral HPLC. Hydrogen atoms and solvent molecules have been omitted from the X-ray structures for clarity. ^*a*^Reactions carried out at 0 °C.

Having identified catalyst 9a as the most suitable, the scope of the cyclisation was evaluated using alkynes 5a–j, which contain substituents of diverse electron nature at their aromatic termini. We were pleased to observe that all reactions proceeded until complete conversion of the substrates; very high levels of enantioinduction were imparted in all cases as well (90–99% ee). The regioselectivity of the cyclisation ranged from mediocre (1i; 65 : 35) to excellent (1f; 94 : 6), but despite that inconvenience, the helicene products were always the major component of the reaction mixtures and were gained in analytically pure form and good yields through preparative HPLC separation. It was also observed that the mesityl substituent at boron does not play a prominent role in this transformation; its formal exchange by a phenyl group neither alters the remarkable level of enantioselectivity, nor the regioselectivity in which 1b(BPh) is obtained. Nonetheless, the N-atom from substrates 5 must be kept unsubstituted; just methylation at that position inverts the regioselectivity of the cyclisation making planar S10b(NMe) the main product of the reaction. Even in that unfavourable case the azabora [5]helicene 1b(NMe) could be isolated in 38% yield with excellent optical purity (97% ee). The isolation and characterization of all side products of general formula 10 can be found in the SI. Crystals suitable for X-ray diffraction analysis of enantiopure 1b, 1d, 1e, 1f, 1g and 1h were obtained; from these measurements the absolute configuration of the six helicenes was assigned as *P* (Fig. 2A and S39–S44). By extension, we assumed the configuration of the whole 1a–j series to be the same.

Subsequently, the extension of the synthetic protocol to the internally BN-doped helicenes 2 was attempted. By submitting alkynes 7 to the reaction conditions already optimized the expected 6-*endo*-dig cyclisation took place in all cases, but the regioselectivity of the process was strongly substrate dependent. Electron donating substituents at the hanging alkyne facilitate the transformation in terms of conversion, but they preferentially direct the cyclisation to the undesired 3-position of the benzo[*e*]benzo[5,6][1,2]azaborino[1,2-*a*][1,2]azaborine core. Thus, for substrates 7b and 7c the main products isolated are 11b and S11c, respectively. Helicenes 2b and 2c are still obtained from these reactions with high ee's but diminished yield. In contrast, electron withdrawing groups at the hanging alkyne preferentially guide the hydroarylation towards the formation of the helicenes 2e and 2h. Hence, azabora[5]helicenes 2b, 2c, 2e and 2h were isolated with high optical purity (84–99% ee) (See Fig. 2B). No X-ray quality crystals of these compounds were obtained, but comparison of the shape and sign of their electronic circular dichroism spectra with those of the 1 series allows us to confidently assign their absolute configuration also as *P*.

We also evaluated the possibility of functionalizing the helicenes just prepared. Thus, treatment of 1b with *N*-bromosuccinimide (NBS) cleanly afforded 12b, in which the electrophilic bromination occurred exclusively at the 4-position of the naphtho[2,1-*c*]phenanthro[1,2-*e*][1,2]azaborine skeleton (For the X-ray structure of 12b see Fig. 2C and S53). This exquisite regioselectivity is remarkable considering that apart of the BN-doping no directing groups were employed.

More unexpected was the clean DDQ-promoted oxidative deborylation of 1b with concomitant C–C coupling of the two aryl moieties originally at boron to deliver aniline 13b (For the X-ray structure of 13b see Fig. 2C and S55). The formation of 13b, which occurs with complete helical to axial chirality transfer, suggests that the oxidation-induced deborylation/C–C coupling manifold known to be operative in the ring contraction of chlorodibenzoborepines into phenanthrenes^64^ might be more general than anticipated, and also functional for structurally related dibenzo[*c*,*e*][1,2]azaborines.

### Racemization dynamics

**Fig. 3:**
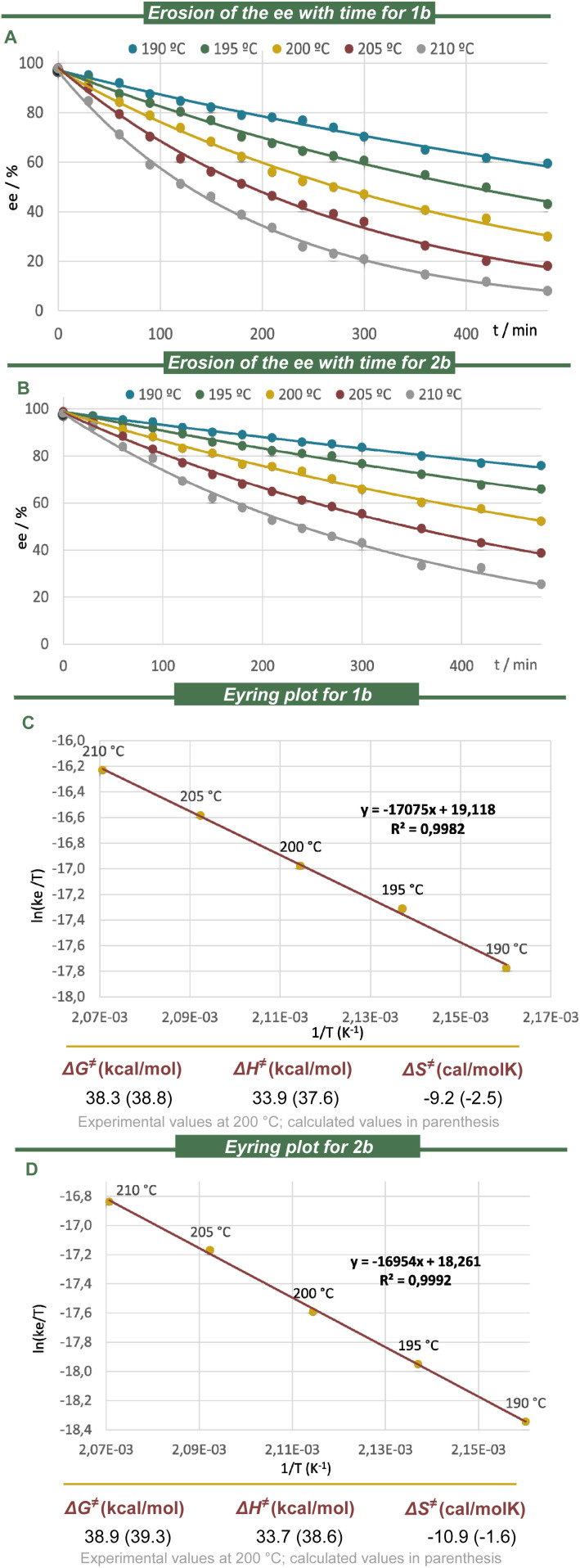
Racemization dynamics. (A–D) Experimental activation free energies of racemization of 1b and 2b at 200 °C and calculated values (in parentheses) at the B3LYP-D3(BJ)/def2-TZVP/CPCM(DCM) level of theory.

The racemization dynamics of the newly prepared azabora[5]helicenes were experimentally determined by heating enantiopure samples of model substrates 1b and 2b in 1,2,4-trichlorobenzene. The racemization rate constants were measured between 190 and 210 °C, and the activation-free energies (Δ*G*^‡^) for the inversion were calculated through an Eyring plot (Fig. 3). As expected from the minimal geometric distortion introduced by BN-doping, the racemization of both compounds, 1b (Δ*G*^‡^ = 38.3 kcal mol^−1^ at 200 °C) and 2b (Δ*G*^‡^ = 38.9 kcal mol^−1^ at 200 °C) is very similar to that of the parent carbo[5]helicene 14 (Δ*G*^‡^ = 37.6 kcal mol^−1^ at 200 °C).^57^ We also conclude that the location of the BN-unit, either in the rim or internally, has very minor effect on the enantiomerization barriers because the wedge angles (*φ*) of benzene and [1,2]-azaborine are basically identical, and B–N bond cleavage is not involved in the process.^65^ The calculated free energies of racemization at the B3LYP-D3/def2-TZVP level closely match the experimentally determined values (Fig. 3C and D);^66–69^ the geometric data of the corresponding transition states are collected in the SI. All structure optimisations were carried out with the Gaussian 16 (Rev. A.03) program package.^70^

### Photophysical and chiroptical characterization

**Fig. 4:**
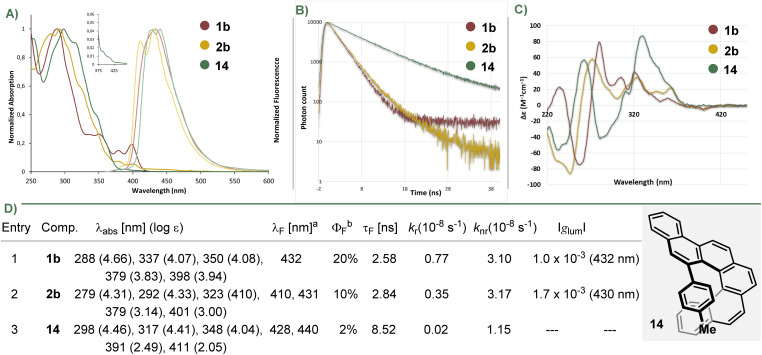
Photophysical and chiroptical properties of selected azabora[5]helicenes. (A) UV/vis (continuous line) and fluorescence spectra (faded line) of selected compounds in CH_2_Cl_2_ (1–2 × 10^−5^ M) at r.t.; (B) fluorescence decay profiles; (C) CD spectra in CH_2_Cl_2_ (8 × 10^−5^ M) at r.t. (D) summary table of optical properties. ^*a*^Compounds 1b and 14 were excited at *λ* = 350 nm.; 2b excited at 370 nm. ^*b*^Compound 1b excited at *λ* = 350 nm.; 2b excited at 300 nm.; 14 excited at 295 nm.; *τ*_F_: fluorescence exited state lifetime; *k*_r_: radiative rate constant; *k*_nr_: non-radiative rate constant.

The UV-vis absorption and fluorescence spectra, as well as fluorescence life times of 1b, 2b were recorded in CH_2_Cl_2_ and compared with those of their parent hydrocarbon 14; they are shown in Fig. 4 (also see Fig. S1–S5). The three model compounds show comparable absorption maxima (*λ*_abs_ = 288 nm, 1b; 279 nm, 2b; 298 nm, 14) with similar molar extinction coefficients (log *ε* = 4.66, 1b; 4.31, 2b; 4.46, 14), but helicenes 1b and 2b are characterized by a significant intensification of the least energetic band (1b, *λ*_abs_ = 398 nm, log *ε* = 3.94; 2b, *λ*_abs_ = 401 nm, log *ε* = 3.00), an effect related with BN-doping.

In the fluorescence spectrum of 1b, only one band centered at *λ*_F_ = 432 nm is observed, while in that of 2b, at least two vibronic bands (*λ*_F_ = 410, 431 nm) are recognized. In terms of fluorescence quantum yields, the BN-doped helicenes were substantially brighter (*φ*_PL_ = 0.20, 1b; 0.10, 2b) than the barely emissive parent carbohelicene (*φ*_PL_ = 0.02, 14). The fluorescence lifetime are very similar for both BN-doped structures (*τ*_F_ = 2.6 ns, 1b; 2.8 ns, 2b) and shorter than that of 14 (*τ*_F_ = 8.5 ns). The small Stokes shifts, and the observable vibrational structures of the emission spectra are indicative of high rigid structures.

The electronic circular dichroism (ECD) spectra of 1b, 2b and 14 were recorded (Fig. 4c; for the complete study see Fig. S10–30). Enantiomer *P*-1b exhibits an intense negative CD band at 258 nm (Δ*ε* = −74.6 M^−1^ cm^−1^) and positive cotton effects in the region from 271 to *ca.* 377 nm (Δ*ε* = 78.3 M^−1^ cm^−1^ at 281 nm; Δ*ε* = 35.1 M^−1^ cm^−1^ at 306 nm, and Δ*ε* = 41.1 M^−1^ cm^−1^ at 322 nm). Similarly, *P*-2b depicts an intense negative CD band in the high energy region centred at 247 nm (Δ*ε* = −85.6 M^−1^ cm^−1^) and positive cotton effects in the region from 259 to *ca.* 380 nm (Δ*ε* = 58.0 M^−1^ cm^−1^ at 274 nm; Δ*ε* = 33.7 M^−1^ cm^−1^ at 326 nm, and Δ*ε* = 22.4 M^−1^ cm^−1^ at 364 nm). CPL spectra for 1b and 2b were recorded (Fig. 4D and S31–34). Both compounds depict comparable luminescence dissymmetry factors (|*g*_lum_| = 1–2 × 10^−3^), which are in the same range as these of [5]helicenes.

**Fig. 5:**
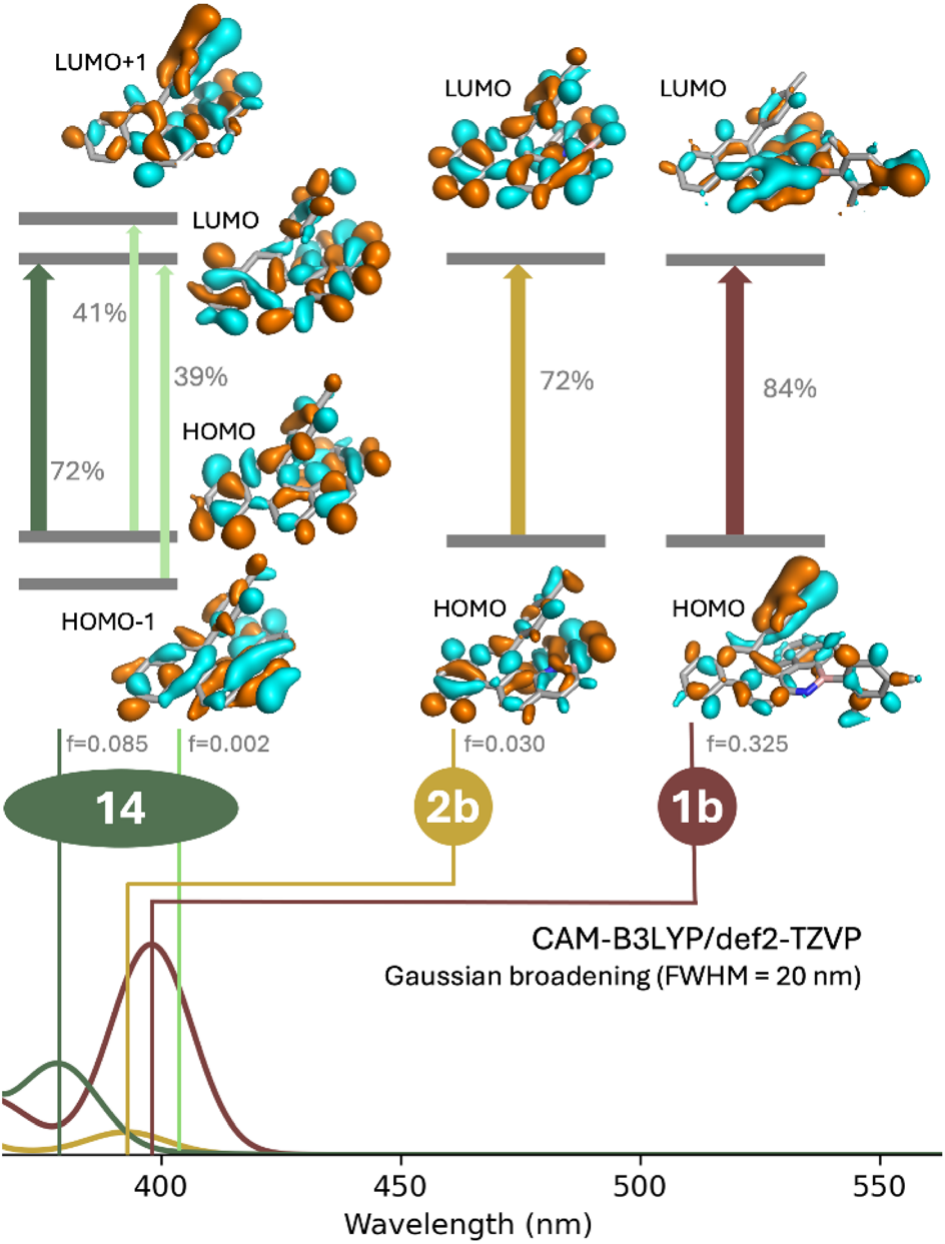
Plots of the frontier molecular orbitals with the transition oscillator strength *f* for the major compositions of S_0_ → S_1_ and the TD-DFT simulated spectra.

To obtain deeper understanding of the impact of BN-doping in these helicenes, we compared the measured UV/vis absorption spectra of the model compounds with simulated results. In line with a previous study carried out by some of us,^71^ we employed simplified time-dependent density functional theory (sTD-DFT) as a fast method to calculate a large portion of the spectra.^72^ The basis set employed was def2-TZVP, with all sTD-DFT calculations carried out with the Orca program package (version 6.0.1).^73^ A uniform shift of −0.4 eV was applied to the transition energies in order to better align the theoretical and experimental spectra. In our calculations, we found necessary to make use of a long-range corrected functional, as some of the bands exhibited a significant charge-transfer character. The study was focused on the first absorption bands of 1b, 2b and 14 around 400 nm (Fig. 5).

The TD-DFT calculations for 1b and 2b revealed the lowest energy transitions to be dominated in both cases by the HOMO–LUMO transition, with weights of 84% and 72% respectively. In the case of 14, CAM-B3LYP/def2-TZVP registers this transition as the second absorption band, with the first transition exhibiting a very low oscillator strength. In analogy to previous reports with carbo[5]helicenes, the S_0_ → S_1_ transition can be primarily described as a combination of two major contributions: HOMO → LUMO +1 and HOMO −1 → LUMO with a low calculated oscillator strength (0.002) if compared to those of 1b (0.325) and 2b (0.030). The isodensity surfaces for the frontier orbitals of all three compounds is provided in Fig. 5. It is noticeable that 2b and 14 show a similar picture, with the transition largely delocalised across the aromatic rings. The main difference is found in 1b, which depicts the most intense band. This comes about through a charge transfer from the non-fused aromatic ring to the BN-containing helicene scaffold. In the case of 2b such an effect is not observed because a charge accumulation in a structure containing the BN bond at a junction between two rings is more penalising to the aromaticity of the helical moiety.

## Conclusions

In summary, we have prepared two families of BN-doped [5]helicenes through a synthetic route that uses a highly enantioselective Au-catalysed hydroarylation as key step. Comparison of the racemization dynamics and photophysical properties of these structures with those of their all-carbon analogue allows an accurate evaluation of the impact of BN-doping. The influence on the inversion barriers is negligible, and also seem to be rather small on *λ*_abs_ at long wavelength (Δ*λ* ≈ 10–15 nm); however, both the intensities of the absorptions and the *φ*_F_ values were significantly enhanced for BN-doped helicenes; particularly, in the case of 1b. TD-DFT calculations attribute this observation to a charge transfer from a non-fused aromatic ring to the BN-containing helicene scaffold, which is specially favoured for 1b.

## Author contributions

C. O., C. T. and L. B. performed the syntheses, routine analytics, racemization kinetics, and chiroptical properties measurements. M. S. performed the HPLC analyses. C. G. performed the X-ray diffraction analyses. M. H, M. A. F. R and P. G. G. developed the synthetic route to compound 6. M. M. and R. M. carried out the DFT calculations. M. A. conceived the project and wrote the manuscript. All authors reviewed the manuscript.

## Conflicts of interest

There are no conflicts to declare.

## Supplementary Material

SC-017-D6SC02344D-s001

SC-017-D6SC02344D-s002

## Data Availability

CCDC 2506342–2506358 contain the supplementary crystallographic data for this paper.^74*a*–*q*^ All data regarding this article can be found in the supplementary information (SI). Supplementary information is available. See DOI: https://doi.org/10.1039/d6sc02344d.
