# Supplementary material for: Enantioselective synthesis of configurationally stable [5]helicenes containing 1,2-azaborine units
Source: Chem Sci. 2026 Apr 23;17(22):11219–28. doi: 10.1039/d6sc02344d (PMC13131005; doi:10.1039/d6sc02344d)
Supplement: SC-017-D6SC02344D-s001 [file SC-017-D6SC02344D-s001.pdf]

Supplementary Information (SI) for Chemical Science.
This journal is © The Royal Society of Chemistry 2026

Supporting Information

Enantioselective Synthesis of Configurationally Stable [5]helicenes Containing 1,2-Azaborine Units

Catherine Olguin, Christian Tabacaru, Lennart Besse, Martin Simon, Christopher Golz, Marcos Humanes, Manuel A. Fernández-Rodríguez, Patricia García-García, Maike Mücke, Ricardo A. Mata* and Manuel Alcarazo**

Table of contents

1. Materials and Methods 4

2. Synthesis and Characterization 7

2.1. Synthetic route of brominated BN-Polyarenes **4**, **S4(BPh)** and **S4(NMe)**.................7

Step 1. Synthesis of 4-bromo-2-(4-phenylbut-1-yn-1-yl)aniline **S6**[6] 7

Step 2. Synthesis of dihydro BN-benzo[c]phenanthrenes **3** and **S3(BPh)** 7

Step 3. Synthesis of BN-benzo[c]phenanthrenes **4** and **S4(BPh)** (oxidation step)............9

N-Methylation. Synthesis of Br-substituted BN-Polyarene **S4(NMe)**............................10

2.2. Synthetic route of chlorinated BN-Polyarene **6** 10

2.3. Negishi cross-couplings. Synthesis of **5a-j**, **7b-h**, **S5b(BPh)** and **S5b(NMe)**..........13

2.4. Au-catalyzed hydroarylation reaction towards azabora[5]helicenes **1a-j**, **1b(BPh)**, **1b(NMe)** and **2b-h**..25

2.5. Post-modification reactions. Synthesis of **1b** and **13b** 45

3. NMR-Spectra 48

4. HPLC-Chromatograms 158

5. Electro-Optical Properties...176

5.1. Absorption (UV-vis) and Fluorescence spectra 176

5.2. Quantum Yield 179

5.3. Lifetime measurement 180

5.4. Circular Dichroism of Helicenes..181

5.5. Circular Polarized Luminescence of Helicenes 189

6. Racemization Experiments 192

7. Crystallographic Supplement...194

7.1. **1b**◦MeCN 194

7.2. **1d**◦CH_2Cl_2..195

7.3. **1e**◦MeCN 197

7.4. **1f** 198

7.5. **1g**◦MeCN 199

7.6. **1h** 200

7.7. **4** 201

7.8. **S4(NMe)** 202

7.9. **S4(BPh)** 203

7.10. **5a** 204

7.11. **5c**◦$HCCl_3$ 205

7.12. **7h** 206

7.13. **S10b**(NMe)◦0.5CH_2Cl_2 207

7.14. **11b** 208

7.15. **12b** 209

7.16. **12b**◦[0.5 CH_2Cl_2; 0.5 MeCN] 210

7.17. **13b**◦1.5 $HCCl_3$ 211

8. Computational Details 212

9. References 223

1. Materials and Methods

Unless stated otherwise, all reactions were carried out using pre-dried glassware under an inert atmosphere (nitrogen or argon) using standard Schlenk techniques, or in a MBraun UNIlab plus glovebox.

Solvents: anhydrous and degassed solvents (DCM, THF, toluene, DCE) were obtained from a *MBraun Solvent Purification System (MB-SPS-800)* or by distillation over the appropriate drying agent and stored under a protective gas atmosphere.

Chromatography: Thin-Layer Chromatography (TLC) was performed with polygram SIL G/UV254 from Macherey-Nagel, and plates were visualized with short-wave UV light (254 and 366 nm). Flash chromatography was performed on Macherey-Nagel 60 (40-63 μm) silica gel.

Starting Materials: all reagents were used as received from commercial suppliers (BLD Pharmatech GmbH, Fisher Scientific GmbH, Sigma-Aldrich Chemie GmbH, ChemPur GmbH and TCI Deutschland GmbH). Silver hexafluoroantimonate ($AgSbF_6$) was purchased from Sigma-Aldrich and transferred into a glovebox. The chiral BINOL-based Au(I) complexes **9a-b**[1] and **9c**,[2] the chiral TADDOL-based Au(I) complex **8a-b**,[3] and compound **14** were prepared according to a reported procedure.[2]

NMR: spectra were recorded on Bruker Avance Neo 600, Avance Neo 400, Avance III HD 400, Avance III 400 or Avance III HD 300 spectrometers. ^{1}H and ^{13}C chemical shifts (δ) are reported in ppm relative to TMS using the solvent signals as reference in Chloroform-d (^{1}H: 7.26 ppm, ^{13}C: 77.16 ppm), Methylene chloride-d_2 (^{1}H: 5.32 ppm, ^{13}C: 53.84 ppm), Tetrahydrofuran-d_8 (^{1}H: 3.58 ppm, ^{13}C: 67.57 ppm), 1,1,2,2-Tetrachloroethane-d_2 (^{1}H: 5.99 ppm, ^{13}C: 73.78 ppm) and benzene-d_6 (^{1}H: 7.16 ppm). Coupling constants (*J*) are given in Hertz (Hz). Data is reported as follows: s = singlet, d = doublet, t = triplet, q = quartet, m = multiplet, br = broad; coupling constants in Hz; integration.

HRMS: spectra were recorded using Bruker Daltonik maXis Q-TOF (ESI), Bruker Daltonik micrOTOF (ESI), Thermo Scietific LTQ Orbitrap XL (ESI), Thermo Scietific Exactive GC-Orbitrap-MS (EI) or Jeol AccuTOF (EI) instruments. Dimensionless mass-to-charge ratios (*m/z*) are given

IR: infrared spectra were recorded on a FT/IR-4600 spectrometer and reported in wavenumbers (cm^{-1}).

Melting point: melting points were measured with a Büchi M-560 apparatus with a heating rate of 5 °C/min.

Specific rotations: were collected using Jasco P-2000 polarimeters at the stated temperature under a Na/Hg lamp, λ = 589 nm (*c* in g/100 mL).

Circular Dichroism: spectra were measured on a Jasco J-1500 spectrometer using a 1.0 mm quartz sample cell.

Circularly Polarized Luminescence: Circularly Polarized Luminescence spectra were performed in an Olis CPL SOLO spectrophotometer using a 1.0 cm path-length quartz cell. A

fixed wavelength LED (310 nm or 270 nm) was used as the excitation source. For compounds **1b**, **2b** and **14**, to prevent sample decomposition during data acquisition, a reduced wavelength range was used to more accurately determine g_{lum}.

UV/Vis: spectra were measured on a Specord S600 or Jasco V-750 spectrometer, using 10 mm quartz sample cells.

Fluorescence spectra: were measured using an Edinburgh Instruments FS5 Spectrofluorometer and a 10 mm quartz sample cell.

Fluorescence Lifetime: measurements were conducted using an Edinburgh Instruments FS5 Spectrofluorometer using a SC-05 cuvette holder. For Irradiation an Edinburgh Instruments EPLED-310 (λ= 310 ± 10 nm, P= 40 µW) was used using a pulse period of 50 or 100 nm. Emission was detected by a PMT-900 detector. Operation of the spectrofluorometer and analysis was performed using the Fluoracle software. The detection wavelength of the instrument was set to the maximum of the emission of the sample with a shutter length of 20-25 nm and using 1024 channels and a time range of 50-100 ns. The measurement was conducted for 30 minutes or until a photon count of 10000 was achieved. Measurements were conducted using 10x10 mm quartz cuvettes. For the IRF, a scattering non-emissive sample (prepared from LUDOX® AM colloidal silica 30 wt.% suspension in water purchased from Sigma Aldrich) was used.

Quantum Yield: absolute photoluminiscence quantum yield measurements were performed using an FS5 spectrofluorometer (Edinburgh Instruments Ltd) equipped with an SC-30 integrating sphere (150 mm diameter, Edinburgh Instruments Ltd) with a surface machined from a PTFE-based material. Identical cuvettes were used for the sample and blank (solvent only). Excitation was provided by a xenon arc lamp and double monochromator system. Emission was detected by a PMT-900 detector. Operation of the spectrofluorometer and analysis was performed using the Fluoracle software. Liquid samples were measured in standard 10 mm × 10 mm quartz cuvettes with PTFE stoppers. Concentrations were adjusted to keep the absorbance below 0.1 for quantum yield measurements.

HPLC: achiral analyses were performed using a Shimadzu Nexera-i LC2040C 3D compact HPLC; preparative HPLC methods were scaled from the analytical methods and processed using an Interchim PuriFlash 4250 combined flash and preparative HPLC system. The columns employed for achiral separations were Agilent Zorbax SB-C18, 4.6x250mm, 3.5µm particles and 21.2x250mm, 7µm particles, for analytical and preparative methods respectively. The enantiomeric excesses of the products were determined using a Waters Acquity multidimensional high-performance liquid chromatograph (MD-UPLC), custom configuration with column switching in both separation dimensions. System comprised of Waters Acquity Sample Manager FT-N, Binary Solvent Manager, Column Manager with additional CM-Aux module, PDA Detector for fast single dimension method screening in first dimension, Quaternary Solvent Manager, Column Manager with 2 additional CM-Aux modules, PDA Detector and SQD2 mass spectrometer in second dimension. Specific conditions such as column type used, eluent mixtures, flow rates and temperatures are stated for each compound. For chiral measurements conditions are denoted for each compound. System control and chromatogram analysis were carried out with Empower 3 (Waters), LabSolutions (Shimadzu) or Intersoft (Interchim) software.

General Crystallography: Data collection was done on two dual source equipped *Bruker D8 Venture* four-circle-diffractometer from *Bruker AXS GmbH*; used X-ray sources: microfocus *IµS 2.0* Cu/Mo and microfocus *IµS 3.0* Ag/Mo from *Incoatec GmbH* with mirror optics *HELIOS* and single-hole collimator from *Bruker AXS GmbH*; used detector: *Photon III CE14* (Cu/Mo) and *Photon III HE* (Ag/Mo) from *Bruker AXS GmbH*. Used programs: *APEX3 Suite* (v2019.11-0) for data collection and therein integrated programs *SAINT* V8.40A (Integration) und *SADABS* 2016/2 (Absorption correction) from *Bruker AXS GmbH*; structure solution was done with *SHELXT*, refinement with *SHELXL*-2018/3;[4] *OLEX*2[5] and *FinalCIF* V85 were used for data finalization.

Special Utilities: *SMZ1270* stereomicroscope from *Nikon Metrology GmbH* was used for sample preparation; crystals were mounted on *MicroMounts* or *MicroLoops* from *MiTeGen* in NVH oil; crystals were cooled to given temperature with *Cryostream 800* from *Oxford Cryosystems*.

2. Synthesis and Characterization

2.1. Synthetic route of brominated BN-Polyarenes **4**, **S4(BPh)** and **S4(NMe)**

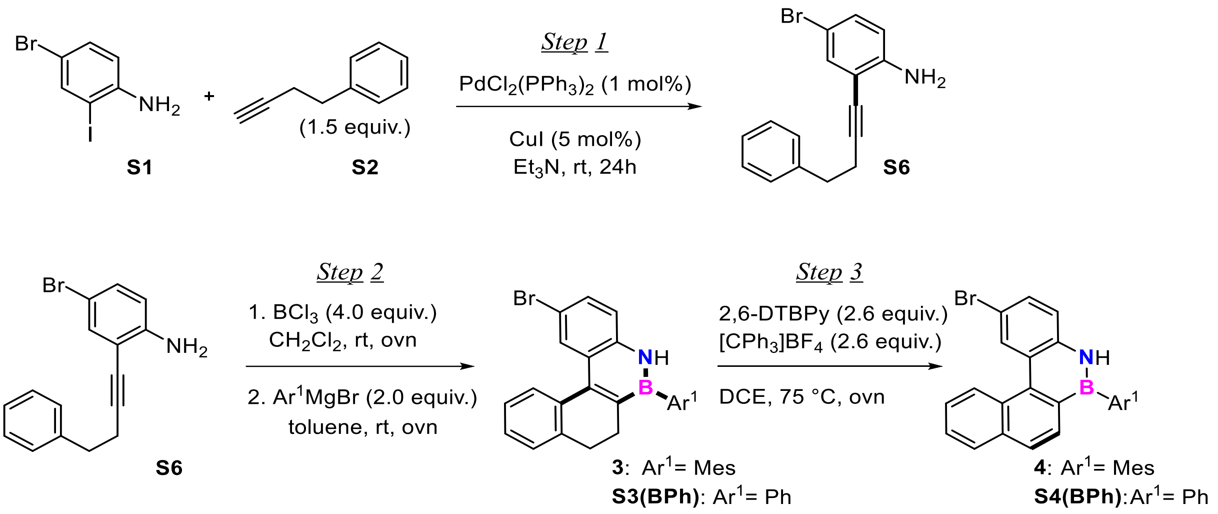

Scheme S1 Synthesis of brominated BN-Polyarenes **4** and **S4(BPh)**

*Step 1. Synthesis of 4-bromo-2-(4-phenylbut-1-yn-1-yl)aniline **S6***[6]

Protocol. A schlenk flask was loaded with 4-bromo-2-iodoaniline **S1** (1.0 g, 3.36 mmol, 1.0 equiv.), CuI (32 mg, 0.168 mmol, 5 mol %) and $PdCl_2(PPh_3)_2$ (24 mg, 0.034 mmol, 1 mol %). Then the schlenk was evacuated and purged with argon five times. Triethylamine (6.4 mL) was added, and the resulting suspension was stirred at room temperature for 30 min. Then, 4-phenyl-1-butyne **S2** (0.71 mL, 5.04 mmol, 1.5 equiv.) was added, and the mixture was stirred at room temperature for 24 h. The resulting mixture was extracted with DCM and the combined organic layers were dried over Na_2SO_4, filtered and concentrated under reduced pressure. Silica gel column chromatography (hexane/AcOEt 95:5) yielded product **S6** (958 mg, 3.19 mmol, 95%) as a yellow-orange oil.

*Step 2. Synthesis of dihydro BN-benzo[c]phenanthrenes **3** and **S3(BPh)***[6]

General protocol. A schlenk flask was loaded with 4-bromo-2-(4-phenylbut-1-yn-1-yl)aniline **S6** (1.0 equiv.) and dissolved in anhydrous DCM (0.25 M). Then a cold (≈ 5 ºC) boron trichloride solution (1.0 M in DCM, 4.0 equiv.) was added dropwise and the solution was left stirring overnight at room temperature. The reaction mixture was concentrated under reduced pressure using Schlenk techniques. Then, the resulting B–Cl intermediate was redissolved in anhydrous toluene (0.25 M) and the corresponding Grignard reagent (2.0 equiv.) was added and the reaction mixture was left stirring overnight. The remaining Grignard was quenched with a 2-propanol/toluene solution (2:8) and the resulting crude mixture was filtered through a Celite plug. The filtrate was concentrated and further purified via silica gel column chromatography.

Dihydro BN-benzo[c]phenanthrene **3**

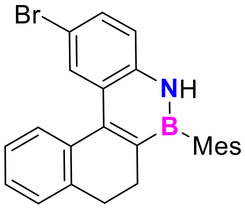

Compound **3** was synthesized following the general procedure from aniline **S6** (450 mg, 1.5 mmol, 1.0 equiv.) and BCl_3 (1.0 M in DCM, 6 mL, 4.0 equiv.). Then, 2-mesitylmagnesium bromide (1.0 M in THF, 3 mL, 2.0 equiv.) was added to the resulting B–Cl intermediate. Silica gel column chromatography (hexane/DCM 5:1) yielded product **3** as a white solid (384 mg, 0.90 mmol, 60%).

^{1}H NMR (300 MHz, $CDCl_3$) δ = 8.44 (d, J = 2.0 Hz, 1H), 7.80 (d, J = 7.6 Hz, 1H), 7.76 (br. s, 1H), 7.50 (dd, J = 8.6, 2.1 Hz, 1H), 7.46 – 7.27 (m, 3H), 7.16 (d, J = 8.6 Hz, 1H), 6.91 (s, 2H), 2.69 – 2.60 (m, 2H), 2.45 – 2.38 (m, 2H), 2.34 (s, 3H), 2.15 (s, 6H) ppm.

^{13}C NMR (101 MHz, $CDCl_3$) δ = 144.5 (1Cq), 141.2 (1Cq), 140.1 (2Cq), 139.4 (1Cq), 137.7 (1Cq), 133.2 (1Cq), 130.04 (1CH), 129.96 (1CH), 128.1 (1CH), 128.0 (2CH), 127.3 (2CH), 126.2 (1CH), 124.6 (1Cq), 120.4 (1CH), 113.7 (1Cq), 29.2 (1CH_2), 27.5 (1CH_2), 22.7 (2CH_3), 21.4 (1CH_3) ppm. The carbon atoms bonded to boron are not observed.

^{11}B NMR (161 MHz, $CDCl_3$) δ = 37.35 ppm.

IR (ATR) $\tilde{\nu}$ = 3364, 2931, 2829, 1605, 1532, 1448, 1429, 1410, 1329, 1263, 1158, 981, 866, 813, 776, 746 cm^{-1}.

HRMS calcd *m/z* for $C_{25}H_{24}BBrN$ $[M+H]^+$: 428.1181; found (pAPCI): 428.1182

M.p. = 110 °C

Dihydro BN-benzo[c]phenanthrene **S3(BPh)**

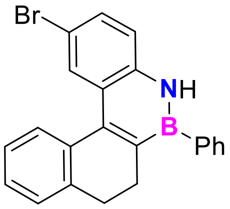

Compound **S3(BPh)** was synthesized following the general procedure from aniline **S6** (450 mg, 1.5 mmol, 1.0 equiv.) and BCl_3 (1.0 M in DCM, 6 mL, 4.0 equiv.). Then, phenylmagnesium bromide (1.0 M in THF, 3 mL, 2.0 equiv.) was added to the resulting B–Cl intermediate.[6] Silica gel column chromatography (hexane/AcOEt 95:5) yielded product **S3(BPh)** as a white solid (369 mg, 0.96 mmol, 63%).

^{1}H NMR (300 MHz, $CDCl_3$) δ = 8.42 (d, J = 1.7 Hz, 1H), 7.90 (br. s, 1H), 7.76 (d, J = 7.2 Hz, 1H), 7.72 – 7.65 (m, 2H), 7.54 – 7.32 (m, 7H), 7.23 (d, J = 8.6 Hz, 1H), 2.86 – 2.76 (m, 2H), 2.73 – 2.63 (m, 2H) ppm.

^{13}C NMR (101 MHz, $CDCl_3$) δ = 145.6 (1Cq), 141.0 (1Cq), 139.3 (1Cq), 133.23 (1Cq), 133.0 (2CH), 130.2 (1CH), 130.1 (1CH), 128.9 (1CH), 128.2 (2CH), 128.1 (1CH), 128.0 (1CH), 127.9 (1CH), 126.2 (1CH), 124.6 (1Cq), 120.4 (1CH), 113.7 (1Cq), 29.3 (1CH_2), 28.0 (1CH_2) ppm. The carbon atoms bonded to boron are not observed.

^{11}B NMR (128 MHz, $CDCl_3$) δ = 35.03 ppm.

HRMS calcd *m/z* for $C_{22}H_{18}BBrN$ $[M+H]^+$: 386.0711; found (pAPCI): 386.0706

*Step 3. Synthesis of BN-benzo[c]phenanthrenes **4** and **S4(BPh)** (oxidation step)*[6]

General protocol. The oxidation of the dihydro BN-benzo[c]phenanthrenes was carried out following a previously reported procedure but using 2,6-di-*tert*-butylpyridine (DTBPy) instead of 2,4,6-tri-*tert*-butylpyridine (TTBP) as sterically-hindered base.[6] A Schlenk flask was loaded with the corresponding dihydro BN-benzo[*c*]phenanthrene (1.0 equiv.), DTBPy (2.6 equiv.) and $[Ph_3C]BF_4$ (2.6 equiv.) and dissolved in anhydrous DCE (0.1 M). The reaction mixture was stirred overnight at 75 °C. The resulting crude mixture was filtered over a Celite pad using DCM/Hexane (20:80) as eluent and the filtrate was concentrated and purified via silica gel column chromatography.

BN-benzo[c]phenanthrene **4**

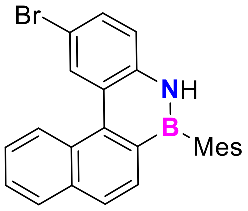

Compound **4** was synthesized following the general procedure from dihydro BN-benzo[c]phenanthrene **3** (139 mg, 0.32 mmol, 1.0 equiv.), DTBPy (0.19 mL, 0.85 mmol, 2.6 equiv.) and $[Ph_3C]BF_4$ (279 mg, 0.85 mmol, 2.6 equiv.) in anhydrous DCE (0.1 M, 3.2 mL). Silica gel column chromatography (hexane/toluene 7:1 to 3:1) yielded product **4** as a white solid (104 mg, 0.24 mmol, 75%).

^{1}H NMR (300 MHz, CD_2Cl_2) δ = 8.98 – 8.87 (m, 2H), 8.05 – 7.96 (m, 1H), 7.95 (br. s, 1H), 7.82 (d, J = 8.2 Hz, 1H), 7.73 – 7.61 (m, 3H), 7.59 (dd, J = 8.5, 2.2 Hz, 1H), 7.32 (d, J = 8.6 Hz, 1H), 6.95 (s, 2H), 2.36 (s, 3H), 2.11 (s, 6H) ppm.

^{13}C NMR (101 MHz, $CDCl_3$) δ = 140.5 (2Cq), 138.8 (1Cq), 138.0 (1Cq), 137.0 (1Cq), 136.4 (1Cq), 132.4 (1CH), 131.1 (1CH), 130.3 (1CH), 129.7 (1Cq), 128.8 (1CH), 127.6 (1CH), 127.44 (1CH), 127.36 (2CH), 127.1 (1CH), 126.3 (1CH), 125.3 (1Cq), 120.5 (1CH), 113.8 (1Cq), 23.0 ($2CH_3$), 21.4 ($1CH_3$) ppm. The carbon atoms bonded to boron are not observed.

^{11}B NMR (161 MHz, CD_2Cl_2) δ = 39.25 ppm.

IR (ATR) $\tilde{\nu}$ = 3375, 1597, 1545, 1472, 1429, 1326, 1247, 854, 814, 744, 717 cm^{-1}.

HRMS calcd *m/z* for $C_{25}H_{21}BBrN$ $[M]^+$: 425.1060; found (ESI): 425.0942

M.p. = 197 °C

BN-benzo[c]phenanthrene **S4(BPh)**

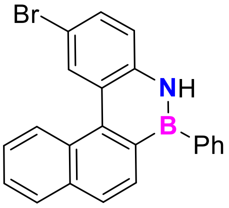

Compound **S4(BPh)** was synthesized following the general procedure from dihydro BN-benzo[c]phenanthrene **S3(BPh)** (1.00 g, 2.59 mmol, 1.0 equiv.), DTBPy (1.5 mL, 6.7 mmol, 2.6 equiv.) and $[Ph_3C]BF_4$ (2.212 g, 6.7 mmol, 2.6 equiv.) in anhydrous DCE (0.1 M, 26 mL). Silica gel column chromatography (hexane/ethyl acetate/toluene 95:2:5) yielded product **S4(BPh)** as a white solid (580 mg, 1.51 mmol, 58%).

^{1}H NMR (400 MHz, $CDCl_3$) δ = 8.92 – 8.87 (m, 1H), 8.85 (d, J = 2.0 Hz, 1H), 8.15 (d, J = 8.2 Hz, 1H), 8.04 – 7.99 (m, 1H), 7.88 (d, J = 8.4 Hz, 1H), 7.86 (br. s, 1H), 7.81 – 7.77 (m, 2H), 7.71 – 7.64 (m, 2H), 7.58 (dd, J = 8.5, 2.3 Hz, 1H), 7.56 – 7.48 (m, 3H), 7.30 (d, J = 8.6 Hz, 1H) ppm.

^{13}C NMR (101 MHz, $CDCl_3$) δ = 138.7 (1Cq), 137.9 (1Cq), 136.1 (1Cq), 133.5 (2CH), 132.5 (1CH), 131.1 (1CH), 130.5 (1CH), 129.6 (1Cq), 129.0 (1CH), 128.7 (1CH), 128.3 (2CH), 127.7 (1CH), 127.2 (2CH), 126.3 (1CH), 125.2 (1Cq), 120.5 (1CH), 113.8 (1Cq) ppm. The carbon atoms bonded to boron are not observed.

^{11}B NMR (128 MHz, $CDCl_3$) δ = 37.37 ppm.

HRMS calcd *m/z* for $C_{22}H_{16}BBrN$ $[M+H]^+$: 384.0554; found (pAPCI): 384.0550

*N-Methylation. Synthesis of Br-substituted BN-Polyarene **S4(NMe)***[7]

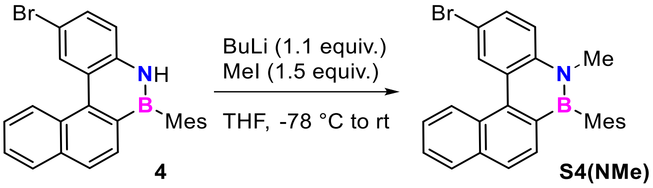

Scheme S2 *N*–Methylation of compound **4**

Protocol. BN-benzo[*c*]phenanthrene **4** (100 mg, 0.23 mmol, 1.0 equiv.) was dissolved in anhydrous THF (0.2 M, 1.1 mL) and *n*-BuLi (0.1 mL, 2.5 M in hexane, 1.1 equiv.) was added at −78 °C. After stirring for 10 minutes, MeI (22 µL, 0.35 mmol, 1.5 equiv.) was added at the same temperature and the reaction mixture left stirring at room temperature overnight. The mixture was diluted with EtOAc and washed with brine and the resulting aqueous solution extracted again with EtOAc. The combined organic fractions were dried over Na_2SO_4 and concentrated under reduced pressure. HPLC purification (acetonitrile:H_2O 95:5→100:0 over 5 min, flow rate 1.0 mL/min at 295 K) yielded product **S4(NMe)** (72 mg, 0.16 mmol, 70%) as a white solid.

^{1}H NMR (300 MHz, $CDCl_3$) δ = 9.04 (d, *J* = 1.8 Hz, 1H), 8.83 (d, *J* = 8.4 Hz, 1H), 7.96 (d, *J* = 7.5 Hz, 1H), 7.85 (dd, *J* = 8.6, 1.6 Hz, 1H), 7.76 – 7.59 (m, 3H), 7.51 – 7.43 (m, 2H), 6.95 (s, 2H), 3.53 (s, 3H), 2.39 (s, 3H), 2.01 (s, 6H) ppm.

^{13}C NMR (101 MHz, $CDCl_3$) δ = 141.7 (1Cq), 139.7 (2Cq), 138.8 (1CH), 137.5 (1Cq), 136.5 (1Cq), 136.3 (1Cq), 136.0 (1CH), 131.1 (1CH), 129.3 (1Cq), 128.7 (1CH), 127.8 (1CH), 127.35 (2CH), 127.27 (1CH), 127.24 (1Cq), 126.9 (1CH), 126.2 (1CH), 117.5 (1CH), 84.0 (1Cq), 36.5 ($1CH_3$), 22.6 ($2CH_3$), 21.5 ($1CH_3$) ppm. The carbon atoms bonded to boron are not observed.

^{11}B NMR (161 MHz, $CDCl_3$) δ = 41.36 ppm.

IR (ATR) $\tilde{\nu}$ = 2912, 1541, 1469, 1412, 1358, 1307, 1281, 849, 809, 751 cm^{-1}.

HRMS calcd *m/z* for $C_{26}H_{24}BBrN$ $[M+H]^+$: 441.1205; found (pAPCI): 441.1209

M. p. = 98 °C

2.2. Synthetic route of chlorinated BN-Polyarene **6**

The synthesis of **6** was carried out by adapting a known protocol for other BN-benzo[*c*]phenanthrenes.[8]

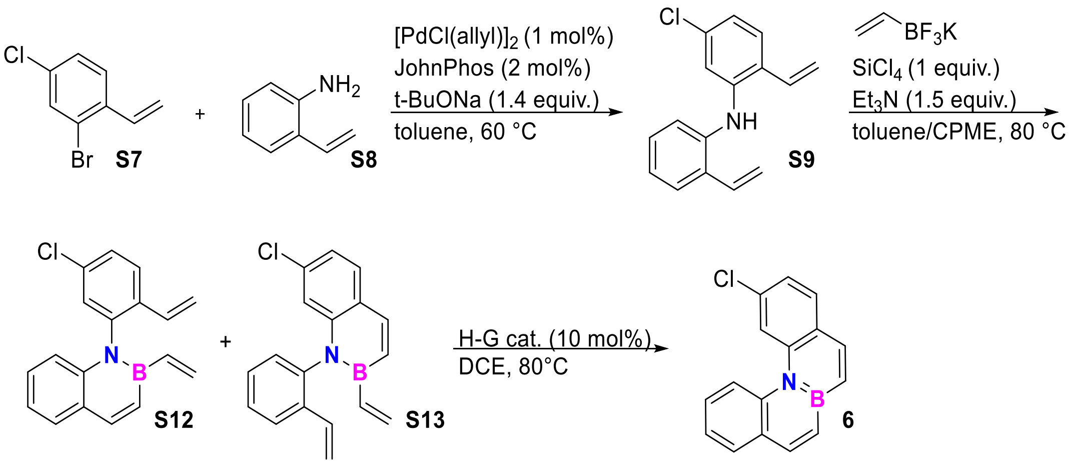

Scheme S3 Synthesis of the chlorinated BN-Polyarene **6**

5-Chloro-2-vinyl-*N*-(2-vinylphenyl)aniline **S9**

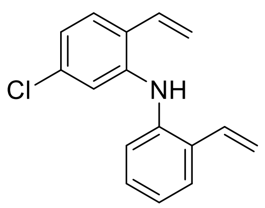

Protocol. To a sealable Schlenk flask with a stir bar were added $[PdCl(allyl)]_2$ (40.4 mg, 0.11 mmol, 1 mol%), JohnPhos (65.9 mg, 0.22 mmol, 2 mol%), and *t*BuONa (1.49 g, 15.44 mmol, 1.4 equiv.). The flask was purged with Ar, and the reagents were suspended in dry toluene (0.6 M). Sequentially, 2-bromo-4-chlorostyrene **S7**[9] (2.40 g, 11.03 mmol, 1.0 equiv.) and 2-vinylaniline **S8** (1.55 mL, 13.2 mmol, 1.2 equiv.) were added. The resulting mixture was heated to 60 ºC and stirred until full consumption of 2-bromo-4-chlorostyrene **S7** (16 h). The reaction mixture was cooled to room temperature, diluted with Et_2O, and filtered over Celite. The solvent was removed under reduced pressure, and the resulting crude mixture was purified by flash column chromatography on silica gel using Hexane/AcOEt 99:1 as eluent to yield product **S9** (2.22 g, 79%).

Obtained as pale yellow oil (2.22 g, 8.68 mmol, 79 %). $R_f = 0.28$ (Hexane/AcOEt 99:1).

^{1}H NMR (400 MHz, $CDCl_3$) δ = 7.53 (dd, *J* = 7.6, 1.6 Hz, 1H), 7.34 (d, *J* = 7.9 Hz, 1H), 7.28–7.22 (m, 1H), 7.14–7.07 (m, 2H), 6.93–6.89 (m, 1H), 6.92 (s, 1H), 6.88–6.79 (m, 2H), 5.75 (dd, *J* = 10.6, 1.4 Hz, 1H), 5.70 (dd, *J* = 10.5, 1.3 Hz, 1H), 5.53 (bs, 1H), 5.39 (dd, *J* = 11.0, 1.4 Hz, 1H), 5.35 (dd, *J* = 11.0, 1.4 Hz, 1H) ppm.

^{13}C NMR (101 MHz, $CDCl_3$) δ = 142.8 (1Cq), 139.5 (1Cq), 134.4 (1Cq), 132.6 (1CH), 131.9 (1CH), 130.7 (1Cq), 128.9 (1CH), 128.5 (1CH), 127.4 (1CH), 126.2 (1Cq), 123.5 (1CH), 121.4 (1CH), 121.1 (1CH), 117.3 (1CH), 117.2 ($1CH_2$), 116.8 ($1CH_2$) ppm.

HRMS (ESI-TOF) m/z: $[M+H]^+$ Calcd for $[C_{16}H_{15}ClN]^+$ 256.0888. Found 256.0887.

1-(3-Chloro-6-vinylphenyl)-2-vinyl-1-aza-2-boranaphthalene **S12** and 7-chloro-2-vinyl-1-(2-vinylphenyl)-1-aza-2-boranaphthalene **S13**

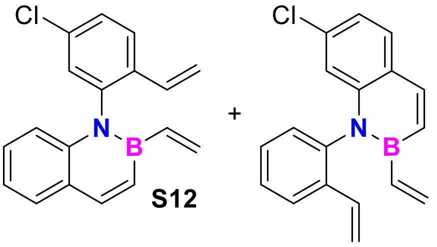
S12 **S13**

Protocol. To a sealable Schlenk flask with a stir bar was added potassium vinyltrifluoroborate (872.9 mg, 6.52 mmol, 1.0 equiv.). The flask was purged with Ar, and cyclopentyl methyl ether and toluene (1:1, 0.25 M) were added, followed by aniline **S9** (2.09 g, 7.8 mmol, 1.2 equiv.), $SiCl_4$ (0.75 mL, 6.52 mmol, 1.0 equiv.) and Et_3N (1.36 mL, 9.78 mmol, 1.5 equiv.). The flask was sealed and the resulting mixture was heated to 80 ºC for 16 h. Then, the reaction mixture was cooled to room temperature, diluted with Et_2O, filtered over a plug of silica gel and flushed with Et_2O. The solvent was removed under reduced pressure and the resulting crude mixture was purified via flash column chromatography on silica gel (hexanes) to provide a mixture of both possible isomers **S12** and **S13** (1.57 g, 83%)

Obtained as colorless oil (1.57 g, 5.38 mmol, 83%, 1/1 mixture of regioisomers). R_f = 0.33 (Hexane).

^{1}H NMR (400 MHz, $CDCl_3$) δ = 8.09 (d, J = 11.6 Hz, 1H), 8.04 (d, J = 11.6 Hz, 1H), 7.79 (dd, J = 7.5, 1.9 Hz, 1H), 7.71 (d, J = 8.5 Hz, 1H), 7.67 (dd, J = 7.7, 1.6 Hz, 1H), 7.58 (d, J = 8.3 Hz, 1H), 7.51–7.41 (m, 3H), 7.31–7.26 (m, 1H), 7.22–7.06 (m, 6H), 6.72–6.66 (m, 2H), 6.29–6.11 (m, 4H), 5.94–5.73 (m, 4H), 5.70 (dd, J = 10.7, 1.1 Hz, 1H), 5.66 (dd, J = 10.7, 1.1 Hz, 1H), 5.13–5.05 (m, 2H) ppm.

^{11}B NMR (128 MHz, $CDCl_3$) δ = 33.7 ppm

^{13}C NMR (101 MHz, $CDCl_3$) δ = 145.3 (1CH), 144.4 (1CH), 143.0 (1Cq), 142.5 (1Cq), 141.8 (1Cq), 140.8 (1Cq), 135.3 (1Cq), 134.34 (1Cq), 134.33 (1Cq), 134.2 (1Cq), 134.02 ($1CH_2$), 133.99 ($1CH_2$), 131.6 (1CH), 130.9 (1CH), 130.8 (1CH), 129.9 (1CH), 129.6 (1CH), 129.4 (1CH), 129.3 (1CH), 128.6 (1CH), 128.23 (1CH), 128.22 (1CH), 127.2 (1CH), 126.4 (1CH), 126.3 (1Cq), 124.8 (1Cq), 121.8 (1CH), 121.5 (1CH), 117.4 (2CH), 116.9 ($1CH_2$), 116.7 ($1CH_2$) ppm. The signals of the C atoms directly bonded to B are not observed.

HRMS (ESI-TOF) m/z: $[M+H]^+$ Calcd for $[C_{18}H_{16}BClN]^+$ 292.1062. Found 292.1060.

2-Chloro-*12b*-aza-*6a*-borabenzo[*c*]phenanthrene **6**

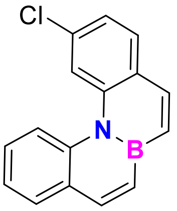

Protocol. To a solution of compounds **S12** and **S13** (1.85 g, 6.34 mmol, 1.0 equiv.) in anhydrous 1,2-dichloroethane (63 mL, 0.1 M), prepared under argon in a Schlenk flask, was added a solution of Hoveyda-Grubbs second generation catalyst (397.6 mg, 0.634 mmol, 10 mol%) in anhydrous 1,2-dichloroethane (12 mL, 0.05 M). The flask was sealed and the reaction mixture was stirred at 80 ºC for 24 h. After cooling to room temperature, the mixture was diluted with DCM, filtered over a plug of silica gel and flushed with DCM. The solvent was removed under reduced pressure, and the crude residue was purified by flash column chromatography on silica gel (hexane) to afford product **6** (1.17 g, 70%).

Obtained as white powder (1.17 g, 4.44 mmol, 70%,). R_f = 0.5 (Hexane). M.p.: 88-90 ºC

^{1}H NMR (400 MHz, $CDCl_3$) δ = 8.53 (ddd, J = 2.0, 0.9, 0.3 Hz, 1H), 8.47-8.44 (m, 1H), 8.01 (d, J = 11.2 Hz, 1H), 7.97 (d, J = 11.2 Hz, 1H), 7.78 (dd, J = 7.7, 1.7 Hz, 1H), 7.69 (d, J = 8.3 Hz, 1H), 7.49 (ddd, J = 8.7, 7.1, 1.7 Hz, 1H), 7.36 (ddd, J = 7.8, 7.1, 1.1 Hz, 1H), 7.30 (dd, J = 8.4, 2.0 Hz, 1H), 7.23 (d, J = 11.2 Hz, 1H), 7.21 (d, J = 11.2 Hz, 1H) ppm.

^{11}B NMR (128 MHz, $CDCl_3$) δ = 30.4 ppm.

^{13}C NMR (101 MHz, $CDCl_3$) δ = 143.5 (1CH), 142.4 (1CH), 139.6 (1Cq), 138.6 (1Cq), 132.5 (1Cq), 130.9 (1CH), 130.7 (2CH, broad signal), 130.1 (1CH), 129.8 (1Cq), 128.1 (1Cq), 126.9 (1CH), 123.4 (1CH), 123.3 (1CH), 120.9 (1CH), 120.8 (1CH) ppm.

HRMS (ESI-TOF) m/z: $[M+H]^+$ Calcd for $[C_{16}H_{11}BClN]^+$ 264.0746. Found 264.0750.

2.3. Negishi cross-couplings. Synthesis of **5a-j**, **7b-h**, **S5b(BPh)** and **S5b(NMe)**

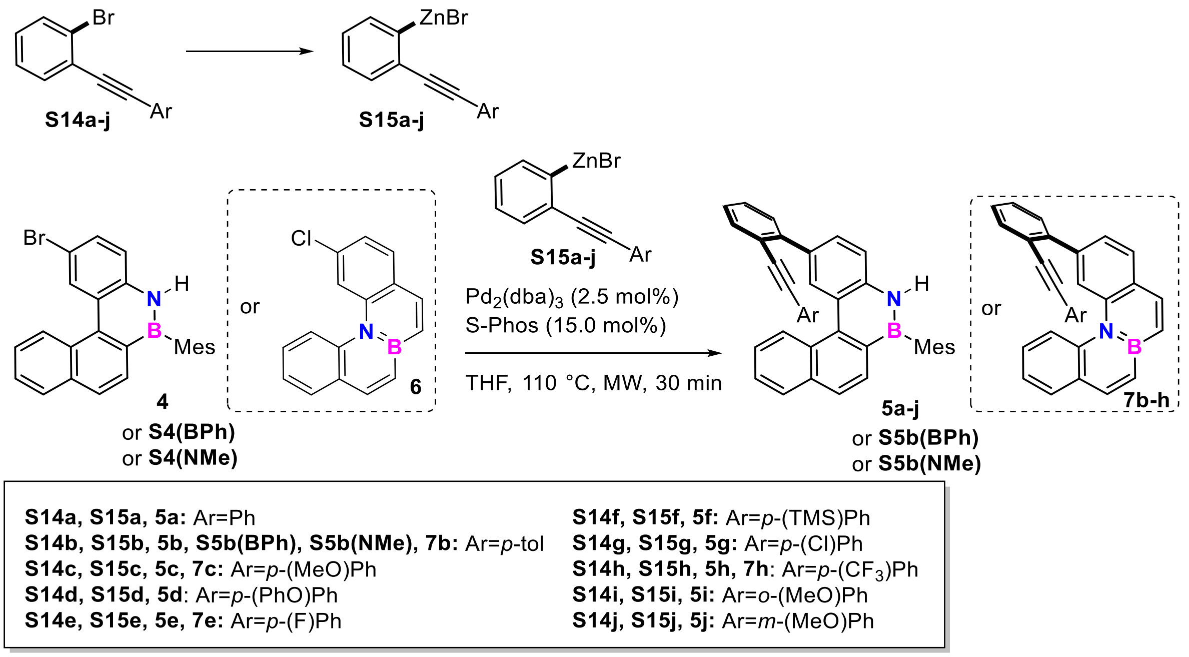

Scheme S4 Negishi-Cross coupling reaction between BN-Polyarenes and organozinc reagents

Bromoalkynes **S14a-j** were prepared according to literature procedures[10],[11],[12],[13],[14],[15]

General protocol for the synthesis of Arylzinc reagents.[16] In a Schlenk flask, the bromoethynyl arene (**S14a-j**) (1.0 equiv.) was dissolved in anhydrous THF (0.25 M) under nitrogen atmosphere. The solution was cooled down to −78 ºC, and *n*-BuLi (2.5 M in hexanes, 1.05 equiv.) was added dropwise. The reaction mixture was stirred for 40 min and then, it was allowed to warm up to −50 °C and a freshly prepared solution of $ZnBr_2$ in anhydrous THF (1.0 M, 1.3 equiv.) was added dropwise. The mixture was stirred vigorously at −50 °C over 60 min and after that the cooling bath was removed, and the reaction mixture was allowed to warm up to room temperature. After stirring the reaction mixture at room temperature for an additional 3 h, the excess of solvent was partially evaporated in vacuo affording a concentrated solution of the organozinc compound suitable for use in the Negishi cross-coupling reaction. (Normally the final solution concentration was 0.6–0.8 M, as determined by titration following Knochel procedure).[17]

General protocol for the Negishi cross-couplings.[16] An oven-dried microwave vial was equipped with a magnetic stirring bar and charged with the respective brominated (**4**, **S4(BPh)**, **S4(NMe)**) or chlorinated (**6**) BN-polyarene (1.0 equiv.), $Pd_2(dba)_3$ (2.5 mol%) and S-Phos (15.0 mol%). The microwave vial was crimped on top with a PFT rubber septum. Then, it was evacuated and refilled with nitrogen (twice). After that, anhydrous THF (0.25 M) was added followed by the organozinc solution (2.5 equiv.). Subsequently, the vial was heated at 110 °C for 30 min under microwave irradiation. Then, the reaction was quenched by addition of MeOH (0.1 mL) and filtered over a pad of Celite with DCM. The filtrate was concentrated and purified by silica gel column chromatography (Hexane/toluene).

Compound **5a**

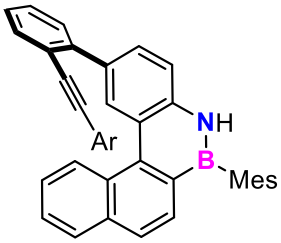

Ar= C_6H_5

Compound **5a** was synthesized following the general procedure from brominated BN-polyarene **4** (150 mg, 0.353 mmol), $Pd_2(dba)_3$ (8 mg, 0.009 mmol, 2.5 mol%), S-Phos (22 mg, 0.053 mmol, 15.0 mol%) and arylzinc derivative **S15a** (0.882 mmol, 2.5 equiv.). Silica gel column chromatography (hexane/toluene 5:1 to 3:1) yielded the product (100 mg, 0.191 mmol, 54%) as a white solid.

^{1}H NMR (400 MHz, CD_2Cl_2) δ = 9.12 (d, J = 8.7 Hz, 1H), 9.09 (s, 1H), 8.05 (br. s, 1H), 7.96 (d, J = 8.1 Hz, 1H), 7.83 (dd, J = 8.2, 1.8 Hz, 1H), 7.79 (d, J = 8.2 Hz, 1H), 7.73 (d, J = 7.6 Hz, 1H), 7.65 (dd, J = 8.2, 1.4 Hz, 1H), 7.59 – 7.51 (m, 3H), 7.50 – 7.36 (m, 3H), 7.26 – 7.14 (m, 5H), 6.96 (s, 2H), 2.37 (s, 3H), 2.15 (s, 6H) ppm.

^{13}C NMR (101 MHz, CD_2Cl_2) δ = 144.3 (1Cq), 140.7 (2Cq), 139.9 (1Cq), 138.6 (1Cq), 138.0 (1Cq), 136.6 (1Cq), 133.8 (1Cq), 133.6 (1CH), 131.7 (2CH), 131.4 (1CH), 131.2 (1CH), 130.2 (1CH), 130.1 (1Cq), 129.2 (1CH), 129.1 (1CH), 128.8 (1CH), 128.63 (2CH), 128.57 (1CH), 128.5 (1CH), 127.5 (2CH), 127.3 (1CH), 127.2 (1CH), 127.0 (1CH), 126.1 (1CH), 123.6 (1Cq), 123.5 (1Cq), 122.0 (1Cq), 119.1 (1CH), 92.6 (1Cq), 90.1 (1Cq), 23.0 ($2CH_3$), 21.4 ($1CH_3$) ppm. The carbon atoms bonded to boron are not observed.

^{11}B NMR (161 MHz, CD_2Cl_2) δ = 39.27 ppm.

IR (ATR) $\tilde{\nu}$ = 3383, 2912, 1550, 1474, 1426, 1364, 1364, 1319, 1152, 913, 820, 753, 718, 686 cm^{-1}.

HRMS calcd m/z for $C_{39}H_{30}BNNa$ $[M+Na]^+$: 546.2370; found (ESI): 546.2384

M. p. = 125 °C

Compound **5b**

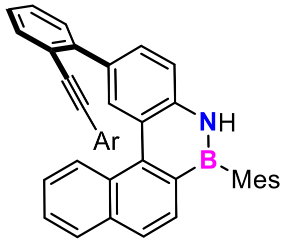

Ar= p-(Me)C_6H_4

Compound **5b** was synthesized following the general procedure from brominated BN-polyarene **4** (1.30 g, 3.060 mmol, 1.0 equiv.), $Pd_2(dba)_3$ (70 mg, 0.077 mmol, 2.5 mol%), S-Phos (188 mg, 0.459 mmol, 15.0 mol%) and arylzinc derivative **S15b** (7.650 mmol, 2.5 equiv.). Silica gel column chromatography (hexane/toluene 5:1 to 3:1) yielded the product (854 mg, 1.590 mmol, 52%) as a white solid.

^{1}H NMR (400 MHz, $CDCl_3$) δ = 9.12 (d, J = 8.5 Hz, 1H), 9.06 (d, J = 1.6 Hz, 1H), 7.94 (dd, J = 8.1, 1.0 Hz, 1H), 7.89 (br. s, 1H), 7.81 (dd, J = 8.3, 1.9 Hz, 1H), 7.76 (d, J = 8.1 Hz, 1H), 7.72 (dd, J = 7.6, 1.4 Hz, 1H), 7.68 (d, J = 8.2 Hz, 1H), 7.54 (ddd, J = 8.1, 6.9, 1.0 Hz, 1H), 7.50 (dd, J = 7.6, 1.2 Hz, 1H), 7.47 – 7.38 (m, 3H), 7.36 (td, J = 7.5, 1.5 Hz, 1H), 7.15 (d, J = 8.2 Hz, 2H), 6.99 – 6.95 (m, 4H), 2.40 (s, 3H), 2.28 (s, 3H), 2.17 (s, 6H) ppm.

^{13}C NMR (101 MHz, $CDCl_3$) δ = 143.9 (1Cq), 140.7 (2Cq), 139.4 (1Cq), 138.42 (1Cq), 138.36 (1Cq), 137.9 (1Cq), 136.3 (1Cq), 133.7 (1Cq), 133.40 (1CH), 131.5 (2CH), 131.20 (1CH), 131.16 (1CH), 129.92 (1CH), 129.86 (1Cq), 129.1 (2CH), 128.9 (1CH), 128.6 (1CH), 128.5 (1CH), 128.4 (1CH), 127.3 (2CH), 127.0 (1CH), 126.9 (1CH), 126.8 (1CH), 125.9 (1CH), 123.5 (1Cq), 122.1 (1Cq), 120.3 (1Cq), 118.7 (1CH), 92.7 (1Cq), 89.3 (1Cq), 23.1 ($2CH_3$), 21.6 ($1CH_3$), 21.4 ($1CH_3$) ppm. The carbon atoms bonded to boron are not observed.

^{11}B NMR (161 MHz, $CDCl_3$) δ = 40.76 ppm.

IR (ATR) $\tilde{\nu}$ = 3372, 3022, 2907, 2851, 1608, 1547, 1509, 1469, 1427, 1325, 814, 750 cm^{-1}.

HRMS calcd *m/z* for $C_{40}H_{32}BNNa$ $[M+Na]^+$: 560.2527; found (ESI): 560.2518

M. p. = 130 °C

Compound **5c**

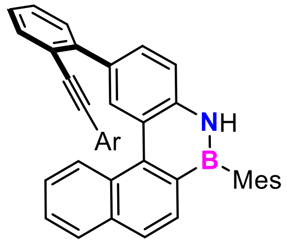

Ar= p-(OMe)C_6H_4

Compound **5c** was synthesized following the general procedure from brominated BN-polyarene **4** (72 mg, 0.169 mmol, 1.0 equiv.), $Pd_2(dba)_3$ (4 mg, 0.004 mmol, 2.5 mol%), S-Phos (10 mg, 0.025 mmol, 15.0 mol%) and arylzinc derivative **S15c** (0.422 mmol, 2.5 equiv.). Silica gel column chromatography (hexane/toluene 5:1 to 3:1) yielded the product (72 mg, 0.130 mmol, 76%) as a white solid.

^{1}H NMR (400 MHz, $CDCl_3$) δ = 9.14 (d, J = 8.6 Hz, 1H), 9.09 (d, J = 1.4 Hz, 1H), 7.95 (d, J = 8.1 Hz, 1H), 7.90 (br. s, 1H), 7.81 (dd, J = 8.3, 2.0 Hz, 1H), 7.77 (d, J = 8.2 Hz, 1H), 7.74 – 7.67 (m, 2H), 7.57 – 7.52 (m, 1H), 7.50 (dd, J = 7.6, 1.2 Hz, 1H), 7.45 (d, J = 8.2 Hz, 1H), 7.44 – 7.39 (m, 2H), 7.36 (td, J = 7.5, 1.5 Hz, 1H), 7.19 (d, J = 8.8 Hz, 2H), 6.99 (s, 2H), 6.69 (d, J = 8.8 Hz, 2H), 3.75 (s, 3H), 2.41 (s, 3H), 2.18 (s, 6H) ppm.

^{13}C NMR (101 MHz, $CDCl_3$) δ = 159.6 (1Cq), 143.7 (1Cq), 140.6 (2Cq), 139.4 (1Cq), 138.4 (1Cq), 137.9 (1Cq), 136.3 (1Cq), 133.8 (1Cq), 133.2 (1CH), 133.0 (2CH), 131.20 (1CH), 131.16 (1CH), 129.90 (1CH), 129.85 (1Cq), 128.9 (1CH), 128.5 (1CH), 128.4 (2CH), 127.3 (2CH), 126.94 (1CH), 126.90 (1CH), 126.7 (1CH), 125.9 (1CH), 123.5 (1Cq), 122.3 (1Cq), 118.7 (1CH), 115.6 (1Cq), 114.0 (2CH), 92.5 (1Cq), 88.7 (1Cq), 55.3 ($1CH_3$), 23.1 ($2CH_3$), 21.4 ($1CH_3$) ppm. The carbon atoms bonded to boron are not observed.

^{11}B NMR (161 MHz, $CDCl_3$) δ = 40.02 ppm.

IR (ATR) $\tilde{\nu}$ = 3371, 2917, 1606, 1560, 1550, 1507, 1472, 1457, 1437, 1430, 1327, 1288, 1270, 1247, 1175, 1031, 910, 825, 730 cm^{-1}.

HRMS calcd *m/z* for $C_{40}H_{32}BNNaO$ $[M+Na]^+$: 576.2476; found (ESI): 576.2478

Compound **5d**

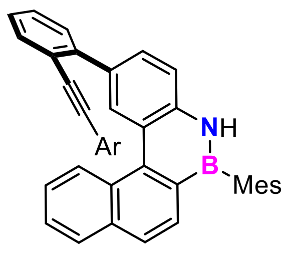

Ar= *p*-(OPh)C_6H_4

Compound **5d** was synthesized following the general procedure from brominated BN-polyarene **4** (150 mg, 0.353 mmol, 1.0 equiv.), $Pd_2(dba)_3$ (8 mg, 0.009 mmol, 2.5 mol%), S-Phos (22 mg, 0.053 mmol, 15.0 mol%) and arylzinc derivative **S15d** (0.882 mmol, 2.5 equiv.). Silica gel column chromatography (hexane/toluene 5:1 to 3:1)) yielded the product (67 mg, 0.109 mmol, 31%) as a yellow solid.

^{1}H NMR (400 MHz, $CDCl_3$) δ = 9.12 (d, J = 8.6 Hz, 1H), 9.09 (d, J = 1.6 Hz, 1H), 7.94 (d, J = 7.8 Hz, 1H), 7.89 (br. s, 1H), 7.80 (dd, J = 8.2, 1.8 Hz, 1H), 7.75 (d, J = 8.2 Hz, 1H), 7.71 (dd, J = 7.6, 1.4 Hz, 1H), 7.68 (d, J = 8.2 Hz, 1H), 7.58 – 7.53 (m, 1H), 7.52 (dd, J = 7.7, 1.4 Hz, 1H), 7.47 – 7.40 (m, 3H), 7.37 (dd, J = 7.5, 1.4 Hz, 1H), 7.36 – 7.30 (m, 2H), 7.18 (d, J = 8.8 Hz, 2H), 7.12 (t, J = 7.4 Hz, 1H), 6.99 – 6.93 (m, 4H), 6.77 (d, J = 8.7 Hz, 2H), 2.40 (s, 3H), 2.16 (s, 6H) ppm.

^{13}C NMR (101 MHz, $CDCl_3$) δ = 157.5 (1Cq), 156.6 (1Cq), 143.9 (1Cq), 140.6 (2Cq), 139.5 (1Cq), 138.4 (1Cq), 137.9 (1Cq), 136.3 (1Cq), 133.7 (1Cq), 133.3 (1CH), 133.1 (2CH), 131.2 (1CH), 131.1 (1CH), 130.0 (2CH), 129.9 (1CH), 129.8 (1Cq), 128.8 (1CH), 128.7 (1CH), 128.5 (1CH), 128.4 (1CH), 127.3 (2CH), 127.0 (1CH), 126.9 (1CH), 126.8 (1CH), 125.9 (1CH), 123.9 (1CH), 123.5 (1Cq), 122.0 (1Cq), 119.4 (2CH), 118.7 (1CH), 118.5 (2CH), 118.0 (1Cq), 92.1 (1Cq), 89.4 (1Cq), 23.1 (2CH_3), 21.4 (1CH_3) ppm. The carbon atoms bonded to boron are not observed.

^{11}B NMR (161 MHz, $CDCl_3$) δ = 40.39 ppm.

IR (ATR) $\tilde{\nu}$ = 3366, 3039, 2959, 2911, 2851, 1608, 1585, 1549, 1503, 1486, 1473, 1427, 1234, 821, 750, 691 cm^{-1}.

HRMS calcd *m/z* for $C_{45}H_{34}BNNaO$ $[M+Na]^+$: 638.2633; found (ESI): 638.2653

M. p. = 116 °C

Compound **5e**

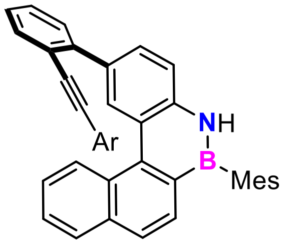

Ar= *p*-(F)C_6H_4

Compound **5e** was synthesized following the general procedure from brominated BN-polyarene **4** (150 mg, 0.353 mmol, 1.0 equiv.), $Pd_2(dba)_3$ (8 mg, 0.008 mmol, 2.5 mol%), S-Phos (22 mg, 0.053 mmol, 15.0 mol%) and arylzinc derivative **S15e** (0.882 mmol, 2.5 equiv.). Purification by HPLC (acetonitrile/H_2O 90:10→100:0 over 10 min, flow rate 1.0 mL/min at 295 K) yielded the product (95 mg, 0.176 mmol, 50%) as a white solid.

^{1}H NMR (400 MHz, $CDCl_3$) δ = 9.14 – 9.07 (m, 2H), 7.95 (d, J = 7.8 Hz, 1H), 7.90 (br. s, 1H), 7.81 – 7.76 (m, 2H), 7.73 – 7.68 (m, 2H), 7.58 – 7.50 (m, 2H), 7.48 – 7.39 (m, 3H), 7.37 (td, J = 7.5, 1.4 Hz, 1H), 7.16 (dd, J = 8.9, 5.4 Hz, 2H), 6.98 (s, 2H), 6.83 (t, J = 8.7 Hz, 2H), 2.40 (s, 3H), 2.17 (s, 6H) ppm.

^{13}C NMR (101 MHz, $CDCl_3$) δ = 162.4 (d, $^1J_{C\text{-}F}$ = 249.6 Hz, 1Cq), 143.9 (1Cq), 140.6 (2Cq), 139.5 (1Cq), 138.3 (1Cq), 137.9 (1Cq), 136.4 (1Cq), 133.6 (1Cq), 133.37 (d, $^3J_{C\text{-}F}$ = 8.4 Hz, 2CH), 133.36 (1CH), 131.3 (1CH), 131.1 (1CH), 129.9 (1CH), 129.8 (1Cq), 128.9 (1CH), 128.8 (1CH), 128.6 (1CH), 128.3 (1CH), 127.4 (2CH), 127.0 (1CH), 126.9 (1CH), 126.8 (1CH), 125.9 (1CH), 123.5 (1Cq), 121.7 (1Cq), 119.5 (d, $^4J_{Cq\text{-}F}$ = 3.4 Hz, 1Cq), 118.7 (1CH), 115.6 (d, $^2J_{C\text{-}F}$ = 22.0 Hz, 2CH), 91.4 (1Cq), 89.6 (1Cq), 23.1 (2CH_3), 21.4 (1CH_3) ppm. The carbon atoms bonded to boron are not observed.

^{11}B NMR (161 MHz, $CDCl_3$) δ = 39.67 ppm.

^{19}F NMR (282 MHz, $CDCl_3$) δ = -111.03 ppm.

IR (ATR) $\tilde{\nu}$ = 3370, 3045, 2912, 2853, 2728, 1607, 1549, 1505, 1471, 1427, 1227, 1154, 821, 752 cm^{-1}.

HRMS calcd *m/z* for $C_{39}H_{29}BFNaN$ $[M+Na]^+$: 564.2276; found (ESI): 564.2279

M.p. = 123 °C

Compound **5f**

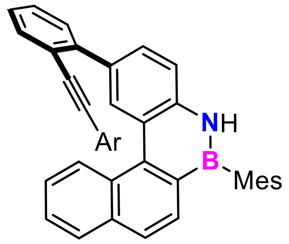

Ar= *p*-(TMS)C_6H_4

Compound **5f** was synthesized following the general procedure from brominated BN-polyarene **4** (82 mg, 0.193 mmol, 1.0 equiv.), $Pd_2(dba)_3$ (4 mg, 0.005 mmol, 2.5 mol%), S-Phos (12 mg, 0.029 mmol, 15.0 mol%) and arylzinc derivative **S15f** (0.480 mmol, 2.5 equiv.). Silica gel column chromatography (hexane/toluene 5:1 to 3:1)) yielded the product (69 mg, 0.116 mmol, 60%) as a white solid.

^{1}H NMR (300 MHz, $CDCl_3$) δ = 9.12 (d, J = 8.7 Hz, 1H), 9.04 (s, 1H), 7.94 (d, J = 8.0 Hz, 1H), 7.89 (br. s, 1H), 7.83 (dd, J = 8.3, 1.9 Hz, 1H), 7.79 – 7.66 (m, 3H), 7.58 – 7.49 (m, 2H), 7.47 – 7.36 (m, 4H), 7.32 (d, J = 8.3 Hz, 2H), 7.23 (d, J = 7.9 Hz, 2H), 6.98 (s, 2H), 2.40 (s, 3H), 2.18 (s, 6H), 0.21 (s, 9H) ppm.

^{13}C NMR (101 MHz, $CDCl_3$) δ = 144.1 (1Cq), 141.0 (1Cq), 140.6 (2Cq), 139.5 (1Cq), 138.4 (1Cq), 137.9 (1Cq), 136.3 (1Cq), 133.7 (1Cq), 133.4 (1CH), 133.2 (2CH), 131.2 (1CH), 131.1

(1CH), 130.6 (2CH), 129.90 (1CH), 129.86 (1Cq), 128.9 (1CH), 128.8 (1CH), 128.5 (1CH), 128.4 (1CH), 127.3 (2CH), 127.0 (1CH), 126.9 (1CH), 126.8 (1CH), 125.9 (1CH), 123.7 (1Cq), 123.5 (1Cq), 122.0 (1Cq), 118.6 (1CH), 92.7 (1Cq), 90.4 (1Cq), 23.1 ($2CH_3$), 21.4 ($1CH_3$), -1.1 ($3CH_3$) ppm. The carbon atoms bonded to boron are not observed.

^{11}B NMR (161 MHz, $CDCl_3$) $\delta = 39.71$ ppm.

IR (ATR) $\tilde{\nu}$ = 3373, 2916, 1610, 1550, 1473, 1429, 1321, 1167, 1126, 1105, 1065, 841, 821, 753 cm^{-1}.

HRMS calcd *m/z* for $C_{42}H_{38}BNNaSi$ $[M+Na]^+$: 618.2766; found (ESI): 618.2788

M. p. = 138 °C

Compound **5g**

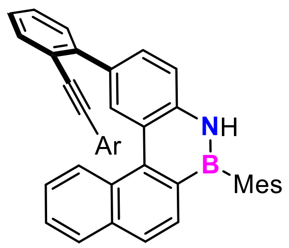

Ar= *p*-(Cl)C_6H_4

Compound **5g** was synthesized following the general procedure from brominated BN-polyarene **4** (200 mg, 0.470 mmol, 1.0 equiv.), $Pd_2(dba)_3$ (11 mg, 0.012 mmol, 2.5 mol%), S-Phos (29 mg, 0.070 mmol, 15.0 mol%) and arylzinc derivative **S15g** (1.175 mmol, 2.5 equiv.). Silica gel column chromatography (hexane/toluene 5:1 to 3:1)) yielded the product (78 mg, 0.140 mmol, 30%) as a white solid.

^{1}H NMR (600 MHz, CD_2Cl_2) δ = 9.11 (s, 1H), 9.09 (d, *J* = 8.6 Hz, 1H), 8.05 (br. s, 1H), 7.97 (d, *J* = 8.0 Hz, 1H), 7.80 (d, *J* = 8.1 Hz, 2H), 7.71 (d, *J* = 7.6 Hz, 1H), 7.65 (d, *J* = 8.3 Hz, 1H), 7.59 – 7.54 (m, 2H), 7.52 (d, *J* = 8.1 Hz, 1H), 7.48 (t, *J* = 7.4 Hz, 1H), 7.46 – 7.42 (m, 1H), 7.39 (t, *J* = 7.6 Hz, 1H), 7.12 (d, *J* = 8.0 Hz, 2H), 7.09 (d, *J* = 8.7 Hz, 2H), 6.96 (s, 2H), 2.37 (s, 3H), 2.15 (s, 6H) ppm.

^{13}C NMR (101 MHz, $CDCl_3$) δ = 144.0 (1Cq), 140.6 (2Cq), 139.5 (1Cq), 138.3 (1Cq), 137.9 (1Cq), 136.4 (1Cq), 134.2 (1Cq), 133.5 (1Cq), 133.4 (1CH), 132.7 (2CH), 131.3 (1CH), 131.1 (1CH), 129.9 (1CH), 129.8 (1Cq), 129.0 (1CH), 128.8 (1CH), 128.7 (2CH), 128.6 (1CH), 128.3 (1CH), 127.4 (2CH), 127.0 (1CH), 126.9 (1CH), 126.8 (1CH), 125.9 (1CH), 123.5 (1Cq), 121.9 (1Cq), 121.6 (1Cq), 118.8 (1CH), 91.4 (1Cq), 90.9 (1Cq), 23.1 ($2CH_3$), 21.4 ($1CH_3$) ppm. The carbon atoms bonded to boron are not observed.

^{11}B NMR (161 MHz, $CDCl_3$) $\delta = 39.84$ ppm.

IR (ATR) $\tilde{\nu}$ = 3368, 2912, 1607, 1549, 1489, 1472, 1427, 1090, 822, 753 cm^{-1}.

HRMS calcd *m/z* for $C_{39}H_{29}BClNNa$ $[M+Na]^+$: 580.1980; found (ESI): 580.2012

M. p. = 115 °C

Compound **5h**

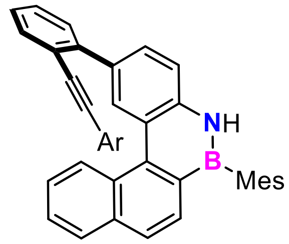

Ar= *p*-$(CF_3)C_6H_4$

Compound **5h** was synthesized following the general procedure from brominated BN-polyarene **4** (90 mg, 0.212 mmol, 1.0 equiv.), $Pd_2(dba)_3$ (5 mg, 0.005 mmol, 2.5 mol%), S-Phos (13 mg, 0.032 mmol, 15.0 mol%) and arylzinc derivative **S15h** (0.530 mmol, 2.5 equiv.). Silica gel column chromatography (hexane/toluene 5:1 to 3:1)) yielded the product (53 mg, 0.090 mmol, 42%) as a white solid.

^{1}H NMR (600 MHz, $CDCl_3$) δ = 9.11 (d, J = 1.5 Hz, 1H), 9.09 (d, J = 8.6 Hz, 1H), 7.95 (d, J = 7.9 Hz, 1H), 7.91 (br. s, 1H), 7.80 – 7.76 (m, 2H), 7.74 (dd, J = 7.8, 1.1 Hz, 1H), 7.70 (d, J = 8.1 Hz, 1H), 7.57 – 7.53 (m, 2H), 7.50 – 7.45 (m, 2H), 7.44 – 7.36 (m, 4H), 7.26 (d, J = 8.1 Hz, 2H), 6.98 (s, 2H), 2.40 (s, 3H), 2.17 (s, 6H) ppm.

^{13}C NMR (126 MHz, $CDCl_3$) δ = 144.3 (1Cq), 140.6 (2Cq), 139.6 (1Cq), 138.3 (1Cq), 138.0 (1Cq), 136.4 (1Cq), 133.6 (1CH), 133.4 (1Cq), 131.6 (2CH), 131.3 (1CH), 131.1 (1CH), 130.0 (1CH), 129.78 (1Cq), 129.76 (q, $^2J_{C–F}$ = 32.4 Hz, 1Cq), 129.4 (1CH), 128.7 (1CH), 128.6 (1CH), 128.2 (1CH), 127.4 (2CH), 127.2 (1Cq), 127.1 (1CH), 126.92 (1CH), 126.87 (1CH), 125.8 (1CH), 125.2 (q, $^3J_{C–F}$ = 3.8 Hz, 2CH), 124.0 (q, $^1J_{C–F}$ = 272.3 Hz, 1Cq), 123.5 (1Cq), 121.2 (1Cq), 118.8 (1CH), 92.4 (1Cq), 91.1 (1Cq), 23.1 ($2CH_3$), 21.4 ($1CH_3$) ppm. The carbon atoms bonded to boron are not observed.

^{11}B NMR (161 MHz, $CDCl_3$) δ = 40.26 ppm.

^{19}F NMR (377 MHz, $CDCl_3$) δ = -62.82 ppm.

IR (ATR) $\tilde{\nu}$ = 3369, 3040, 2915, 2851, 1609, 1550, 1472, 1428, 1319, 1166, 1124, 1104, 1065, 840, 821, 752 cm^{-1}.

HRMS calcd *m/z* for $C_{40}H_{29}BF_3NNa$ $[M+Na]^+$: 614.2244; found (ESI): 614.2275

M. p. = 125 °C

Compound **5i**

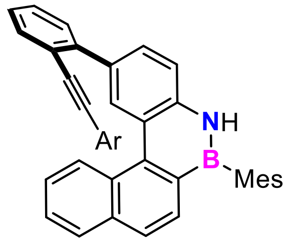

Ar= *o*-(OMe)C_6H_4

Compound **5i** was synthesized following the general procedure from brominated BN-polyarene **4** (310 mg, 0.729 mmol, 1.0 equiv.), $Pd_2(dba)_3$ (17 mg, 0.018 mmol, 2.5 mol%), S-Phos (45 mg, 0.109 mmol, 15.0 mol%) and arylzinc derivative **S15i** (1.822 mmol, 2.5 equiv.). Silica gel column chromatography (hexane/toluene 3:1 to 1:1)) yielded the product (328 mg, 0.593 mmol, 81%) as a yellow solid.

^{1}H NMR (300 MHz, $CDCl_3$) δ = 9.14 (d, J = 8.2 Hz, 1H), 9.05 (s, 1H), 7.96 – 7.83 (m, 3H), 7.80 – 7.72 (m, 2H), 7.67 (d, J = 8.3 Hz, 1H), 7.57 – 7.32 (m, 6H), 7.23 – 7.13 (m, 2H), 6.97 (s, 2H), 6.79 – 6.70 (m, 2H), 3.61 (s, 3H), 2.39 (s, 3H), 2.16 (s, 6H) ppm.

^{13}C NMR (101 MHz, $CDCl_3$) δ = 160.0 (1Cq), 143.9 (1Cq), 140.6 (2Cq), 139.4 (1Cq), 138.5 (1Cq), 137.9 (1Cq), 136.3 (1Cq), 133.8 (1Cq), 133.5 (1CH), 133.4 (1CH), 131.2 (1CH), 131.1

(1CH), 129.90 (1Cq), 129.88 (1CH), 129.7 (1CH), 129.0 (1CH), 128.6 (1CH), 128.5 (1CH), 128.4 (1CH), 127.3 (2CH), 126.90 (1CH), 126.87 (1CH), 126.7 (1CH), 125.8 (1CH), 123.5 (1Cq), 122.4 (1Cq), 120.4 (1CH), 118.6 (1CH), 112.8 (1Cq), 110.7 (1CH), 93.9 (1Cq), 89.0 (1Cq), 55.6 ($1CH_3$), 23.1 ($2CH_3$), 21.4 ($1CH_3$) ppm. The carbon atoms bonded to boron are not observed.

^{11}B NMR (161 MHz, $CDCl_3$) δ = 39.71 ppm.

IR (ATR) $\tilde{\nu}$ = 3368, 2911, 1607, 1548, 1471, 1428, 1275, 1244, 1023, 905, 821, 748, 725 cm^{-1}.

HRMS calcd *m/z* for $C_{40}H_{32}BNNaO$ $[M+Na]^+$: 576.2476; found (ESI): 576.2463

M. p. = 125 °C

Compound **5j**

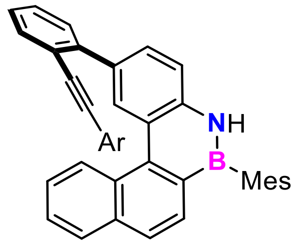

Ar= *m*-(OMe)C_6H_4

Compound **5j** was synthesized following the general procedure from brominated BN-polyarene **4** (100 mg, 0.235 mmol, 1.0 equiv.), $Pd_2(dba)_3$ (5 mg, 0.006 mmol, 2.5 mol%), S-Phos (14 mg, 0.035 mmol, 15.0 mol%) and arylzinc derivative **S15j** (0.587 mmol, 2.5 equiv.). Silica gel column chromatography (hexane/toluene 5:1 to 3:1) yielded the product (43 mg, 0.078 mmol, 33%) as a white solid.

^{1}H NMR (400 MHz, $CDCl_3$) δ = 9.12 (d, *J* = 8.7 Hz, 1H), 9.05 (s, 1H), 7.93 (d, *J* = 8.1 Hz, 1H), 7.89 (br. s, 1H), 7.82 (dd, *J* = 8.4, 1.8 Hz, 1H), 7.78 – 7.71 (m, 2H), 7.68 (d, *J* = 7.9 Hz, 1H), 7.54 (t, *J* = 7.5 Hz, 1H), 7.51 (dd, *J* = 7.8, 1.1 Hz, 1H), 7.48 – 7.41 (m, 3H), 7.37 (td, *J* = 7.5, 1.4 Hz, 1H), 7.07 (t, *J* = 7.9 Hz, 1H), 6.97 (s, 2H), 6.85 (d, *J* = 7.6 Hz, 1H), 6.82 – 6.79 (m, 1H), 6.79 – 6.75 (m, 1H), 3.62 (s, 3H), 2.39 (s, 3H), 2.16 (s, 6H) ppm.

^{13}C NMR (101 MHz, $CDCl_3$) δ = 159.3 (1Cq), 144.1 (1Cq), 140.6 (2Cq), 139.5 (1Cq), 138.3 (1Cq), 137.9 (1Cq), 136.3 (1Cq), 133.7 (1Cq), 133.4 (1CH), 131.2 (1CH), 131.1 (1CH), 129.9 (1CH), 129.8 (1Cq), 129.4 (1CH), 128.9 (1CH), 128.8 (1CH), 128.5 (1CH), 128.4 (1CH), 127.3 (2CH), 127.0 (1CH), 126.9 (1CH), 126.8 (1CH), 125.9 (1CH), 124.4 (1Cq), 124.1 (1CH), 123.5 (1Cq), 121.9 (1Cq), 118.7 (1CH), 116.2 (1CH), 115.0 (1CH), 92.4 (1Cq), 89.8 (1Cq), 55.2 ($1CH_3$), 23.1 ($2CH_3$), 21.4 ($1CH_3$) ppm. The carbon atoms bonded to boron are not observed.

^{11}B NMR (161 MHz, $CDCl_3$) δ = 40.61 ppm.

IR (ATR) $\tilde{\nu}$ = 3366, 2960, 2907, 1576, 1548, 1473, 1427, 1321, 1260, 1221, 1035, 820, 751, 685, 668 cm^{-1}.

HRMS calcd *m/z* for $C_{40}H_{32}BNNaO$ $[M+Na]^+$: 576.2476; found (ESI): 576.2486

M. p. = 119 °C

Compound **S5b(BPh)**

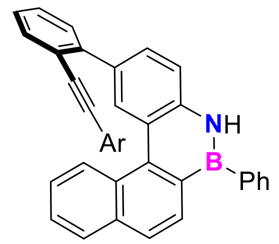

Ar= p-(Me)C_6H_4

Compound **S5b(BPh)** was synthesized following the general procedure from brominated BN-polyarene **S4(BPh)** (300 mg, 0.783 mmol, 1.0 equiv.), $Pd_2(dba)_3$ (18 mg, 0.019 mmol, 2.5 mol%), S-Phos (48 mg, 0.117 mmol, 15.0 mol%) and arylzinc derivative **S15b** (1.957 mmol, 2.5 equiv.). Silica gel column chromatography (hexane/toluene 5:1 to 3:1)) yielded the product (226 mg, 0.456 mmol, 58%) as a white solid.

^{1}H NMR (300 MHz, CD_2Cl_2) δ = 9.09 (d, J = 8.6 Hz, 1H), 9.05 (d, J = 1.8 Hz, 1H), 8.18 (d, J = 8.2 Hz, 1H), 8.13 (br. s, 1H), 8.00 (d, J = 8.0 Hz, 1H), 7.94 – 7.79 (m, 4H), 7.70 (dd, J = 7.5, 1.5 Hz, 1H), 7.62 – 7.50 (m, 6H), 7.48 – 7.40 (m, 2H), 7.37 (td, J = 7.5, 1.5 Hz, 1H), 7.06 (d, J = 8.2 Hz, 2H), 6.97 (d, J = 8.0 Hz, 2H), 2.26 (s, 3H) ppm.

^{13}C NMR (101 MHz, CD_2Cl_2) δ = 144.1 (1Cq), 139.7 (1Cq), 139.4 (1Cq), 139.0 (1Cq), 136.4 (1Cq), 133.94 (1Cq), 133.89 (2CH), 133.5 (1CH), 131.5 (2CH), 131.4 (1CH), 131.3 (1CH), 130.1 (1CH), 130.0 (1Cq), 129.4 (2CH), 129.3 (1CH), 129.1 (1CH), 129.0 (1CH), 128.7 (1CH), 128.6 (1CH), 128.5 (2CH), 127.3 (2CH), 126.8 (1CH), 126.1 (1CH), 123.5 (1Cq), 122.2 (1Cq), 120.4 (1Cq), 119.0 (1CH), 92.8 (1Cq), 89.4 (1Cq), 21.5 ($1CH_3$) ppm. The carbon atoms bonded to boron are not observed.

^{11}B NMR (161 MHz, CD_2Cl_2) δ = 37.28 ppm.

IR (ATR) $\tilde{\nu}$ = 3375, 3049, 2210, 1509, 1548, 1471, 815, 737, 691 cm^{-1}.

HRMS calcd *m/z* for $C_{37}H_{26}BN$ $[M]^+$: 495.2159; found (ESI): 495.2163.

M. p. = 130 °C

Compound **S5b(NMe)**

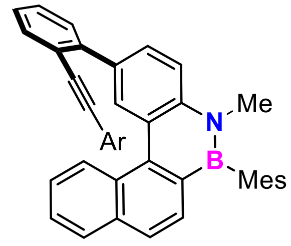

Ar= *p*-(Me)C_6H_4

Compound **S5b(NMe)** was synthesized following the general procedure from brominated BN-polyarene **S4(NMe)** (88 mg, 0.200 mmol, 1.0 equiv.), $Pd_2(dba)_3$ (5 mg, 0.005 mmol, 2.5 mol%), S-Phos (12 mg, 0.030 mmol, 15.0 mol%) and arylzinc derivative **S15b** (0.500 mmol, 2.5 equiv.). Silica gel column chromatography (hexane/toluene 5:1 to 3:1)) yielded the product (70 mg, 0.127 mmol, 63%) as a white solid.

^{1}H NMR (600 MHz, $CDCl_3$) δ = 9.07 (d, J = 2.1 Hz, 1H), 9.05 (d, J = 8.4 Hz, 1H), 7.94 (dd, J = 8.6, 2.1 Hz, 1H), 7.91 (d, J = 8.0 Hz, 1H), 7.82 (d, J = 8.7 Hz, 1H), 7.73 – 7.69 (m, 2H), 7.54 – 7.50 (m, 2H), 7.50 (d, J = 8.1 Hz, 1H), 7.44 – 7.38 (m, 2H), 7.36 (dd, J = 7.6, 1.4 Hz, 1H), 7.12 (d, J = 8.2 Hz, 2H), 6.98 – 6.95 (m, 4H), 3.63 (s, 3H), 2.41 (s, 3H), 2.28 (s, 3H), 2.06 (s, 6H) ppm.

^{13}C NMR (101 MHz, $CDCl_3$) δ = 143.7 (1Cq), 141.7 (1Cq), 139.8 (2Cq), 138.3 (1Cq), 138.1 (1Cq), 137.4 (1Cq), 136.3 (1Cq), 133.4 (1CH), 133.3 (1Cq), 131.5 (1CH), 131.4 (2CH), 131.2 (1CH), 129.9 (1CH), 129.5 (1Cq), 129.1 (2CH), 128.8 (1CH), 128.60 (1CH), 128.59 (1CH), 128.4 (1CH), 127.3 (2CH), 126.9 (1CH), 126.7 (1CH), 126.6 (1CH), 125.8 (1CH), 124.8 (1Cq),

122.1 (1Cq), 120.4 (1Cq), 115.1 (1CH), 92.7 (1Cq), 89.3 (1Cq), 36.7 (1CH$_3$), 22.7 (2CH$_3$), 21.6 (1CH$_3$), 21.5 (1CH$_3$) ppm. The carbon atoms bonded to boron are not observed.

^{11}B NMR (161 MHz, CDCl$_3$) δ = 41.07 ppm.

IR (ATR) $\tilde{\nu}$ = 3049, 2910, 2851, 1608, 1547, 1508, 1469, 1291, 814, 751 cm^{-1}.

HRMS calcd *m/z* for $C_{41}H_{34}BNNa$ [M+Na]$^+$: 574.2683; found (ESI): 574.2698

M. p. = 125 °C

Compound **7b**

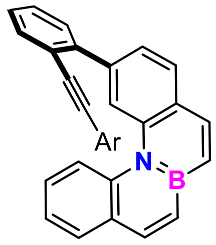

Ar= *p*-(Me)C$_6$H$_4$

Compound **7b** was synthesized following the general procedure from chlorinated BN-polyarene **6** (400 mg, 1.520 mmol, 1.0 equiv.), $Pd_2(dba)_3$ (35 mg, 0.038 mmol, 2.5 mol%), S-Phos (94 mg, 0.228 mmol, 15.0 mol%) and arylzinc derivative **S15b** (3.801 mmol, 2.5 equiv.). Silica gel column chromatography (hexane/toluene 10:1) yielded the product (354 mg, 0.845 mmol, 56%) as a yellow solid.

^{1}H NMR (400 MHz, CDCl$_3$) δ = 8.80 (d, J = 1.5 Hz, 1H), 8.65 (dd, J = 7.8, 1.3 Hz, 1H), 8.09 (d, J = 11.2 Hz, 1H), 8.01 (d, J = 11.2 Hz, 1H), 7.86 (d, J = 8.1 Hz, 1H), 7.73 (dd, J = 7.2, 2.1 Hz, 1H), 7.70 – 7.66 (m, 1H), 7.63 (dd, J = 7.9, 1.6 Hz, 1H), 7.44 – 7.33 (m, 3H), 7.29 – 7.19 (m, 4H), 7.14 (d, J = 8.2 Hz, 2H), 7.01 (d, J = 8.0 Hz, 2H), 2.29 (s, 3H) ppm.

^{13}C NMR (101 MHz, CDCl$_3$) δ = 143.6 (1Cq), 143.2 (1CH), 143.0 (1CH), 139.4 (1Cq), 138.9 (1Cq), 138.7 (1Cq), 138.5 (1Cq), 133.2 (1CH), 131.4 (2CH), 129.9 (1CH), 129.8 (1CH), 129.6 (1Cq), 129.5 (1CH), 129.2 (2CH), 128.8 (1Cq), 128.5 (1CH), 127.4 (1CH), 126.7 (1CH), 124.3 (1CH), 123.0 (1CH), 122.2 (1Cq), 122.1 (1CH), 121.5 (1CH), 120.2 (1Cq), 92.9 (1Cq), 89.0 (1Cq), 21.6 (1CH$_3$) ppm. The carbon atoms bonded to boron are not observed.

^{11}B NMR (161 MHz, CDCl$_3$) δ = 31.36 ppm.

IR (ATR) $\tilde{\nu}$ = 3019, 2918, 2862, 1592, 1555, 1532, 1509, 1475, 1431, 1342, 1282, 1263, 1213, 1165, 894, 828, 817, 799, 753, 735 cm^{-1}.

HRMS calcd *m/z* for $C_{31}H_{22}BNNa$ [M+Na]$^+$: 442.1743; found (ESI): 442.1763

Compound **7c**

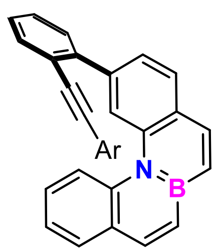

Ar= *p*-(OMe)C$_6$H$_4$

Compound **7c** was synthesized following the general procedure from chlorinated BN-polyarene **6** (100 mg, 0.380 mmol, 1.0 equiv.), $Pd_2(dba)_3$ (9 mg, 0.009 mmol, 2.5 mol%), S-Phos (23 mg, 0.057 mmol, 15.0 mol%) and arylzinc derivative **S15c** (0.950 mmol, 2.5 equiv.). Silica gel column chromatography (hexane/toluene 10:1)) yielded the product (69 mg, 0.159 mmol, 42%) as a white solid.

^{1}H NMR (400 MHz, CDCl$_3$) δ = 8.81 (d, J = 1.2 Hz, 1H), 8.65 (dd, J = 7.7, 1.4 Hz, 1H), 8.09 (d, J = 11.2 Hz, 1H), 8.01 (d, J = 11.0 Hz, 1H), 7.86 (d, J

= 8.1 Hz, 1H), 7.75 – 7.71 (m, 1H), 7.68 – 7.65 (m, 1H), 7.63 (dd, J = 8.0, 1.6 Hz, 1H), 7.44 – 7.41 (m, 1H), 7.40 – 7.32 (m, 2H), 7.29 – 7.19 (m, 4H), 7.17 (d, J = 8.9 Hz, 2H), 6.73 (d, J = 8.9 Hz, 2H), 3.76 (s, 3H) ppm.

^{13}C NMR (101 MHz, $CDCl_3$) δ = 159.7 (1Cq), 143.4 (1Cq), 143.1 (1CH), 143.0 (1CH), 139.4 (1Cq), 138.9 (1Cq), 138.6 (1Cq), 133.1 (1CH), 133.0 (2CH), 129.84 (1CH), 129.77 (1CH), 129.6 (1Cq), 129.5 (1CH), 128.8 (1Cq), 128.3 (1CH), 127.4 (1CH), 126.7 (1CH), 124.3 (1CH), 123.0 (1CH), 122.4 (1Cq), 122.1 (1CH), 121.5 (1CH), 115.4 (1Cq), 114.1 (2CH), 92.7 (1Cq), 88.4 (1Cq), 55.4 (1CH_3) ppm. The carbon atoms bonded to boron are not observed.

^{11}B NMR (128 MHz, $CDCl_3$) δ = 30.69 ppm.

HRMS calcd *m/z* for $C_{31}H_{23}BNO$ $[M+H]^+$: 436.1869; found (ESI): 436.1872

Compound **7e**

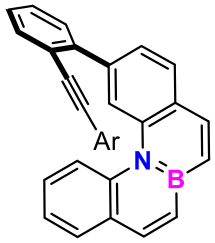

Ar= *p*-(F)C_6H_4

Compound **7e** was synthesized following the general procedure from chlorinated BN-polyarene **6** (50 mg, 0.190 mmol, 1.0 equiv.), $Pd_2(dba)_3$ (4 mg, 0.005 mmol, 2.5 mol%), S-Phos (12 mg, 0.028 mmol, 15.0 mol%) and arylzinc derivative **S15e** (0.475 mmol, 2.5 equiv.). HPLC purification (acetonitrile:H_2O 90:10→100:0 over 10 min, flow rate 1.0 mL/min at 295 K) yielded the product (44 mg, 0.104 mmol, 55%) as a white solid.

^{1}H NMR (400 MHz, $CDCl_3$) δ = 8.81 (s, 1H), 8.62 – 8.57 (m, 1H), 8.06 (d, J = 11.2 Hz, 1H), 7.99 (d, J = 11.2 Hz, 1H), 7.83 (d, J = 8.1 Hz, 1H), 7.73 – 7.68 (m, 1H), 7.63 (dd, J = 7.5, 1.4 Hz, 1H), 7.58 (dd, J = 8.1, 1.6 Hz, 1H), 7.43 (dd, J = 7.6, 1.6 Hz, 1H), 7.38 (td, J = 7.5, 1.5 Hz, 1H), 7.32 (td, J = 7.4, 1.7 Hz, 1H), 7.25 – 7.17 (m, 4H), 7.13 (dd, J = 8.8, 5.4 Hz, 2H), 6.85 (t, J = 8.8 Hz, 2H) ppm.

^{13}C NMR (101 MHz, $CDCl_3$) δ = 162.52 (d, $^1J_{C\text{-}F}$ = 249.7 Hz, 1Cq), 143.67 (1Cq), 143.16 (1CH), 142.92 (1CH), 139.16 (1Cq), 138.81 (1Cq), 138.64 (1Cq), 133.31 (d, $^3J_{C\text{-}F}$ = 8.4 Hz, 2CH), 133.19 (1CH), 129.86 (1CH), 129.83 (1CH), 129.67 (1Cq), 129.57 (1CH), 128.87 (1Cq), 128.78 (1CH), 127.46 (1CH), 126.63 (1CH), 124.25 (1CH), 123.06 (1CH), 121.96 (1CH), 121.81 (1Cq), 121.41 (1CH), 119.36 (d, $^4J_{Cq\text{-}F}$ = 3.3 Hz, 1Cq), 115.71 (d, $^2J_{C\text{-}F}$ = 22.1 Hz, 2CH), 91.62 (1Cq), 89.28 (d, $^5J_{Cq\text{-}F}$ = 1.4 Hz, 1Cq) ppm. The carbon atoms bonded to boron are not observed.

^{11}B NMR (161 MHz, $CDCl_3$) δ = 30.83 ppm.

^{19}F NMR (282 MHz, $CDCl_3$) δ = -110.82 ppm.

IR (ATR) $\tilde{\nu}$ = 3014, 1591, 1550, 1506, 1473, 1219, 1154, 1092, 834, 810, 757, 736 cm^{-1}.

HRMS calcd *m/z* for $C_{30}H_{19}BFNNa$ $[M+Na]^+$: 446.1492; found (ESI): 446.1490

Compound **7h**

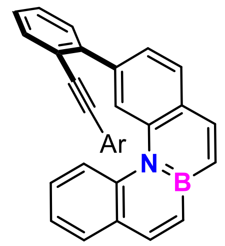

Ar= *p*-$(CF_3)C_6H_4$

Compound **7h** was synthesized following the general procedure from chlorinated BN-polyarene **6** (50 mg, 0.190 mmol, 1.0 equiv.), $Pd_2(dba)_3$ (4 mg, 0.005 mmol, 2.5 mol%), S-Phos (12 mg, 0.028 mmol, 15.0 mol%) and arylzinc derivative **S15h** (0.475 mmol, 2.5 equiv.). Silica gel column chromatography (hexane/toluene 10:1)) yielded the product (58 mg, 0.123 mmol, 64%) as a white solid.

^{1}H NMR (400 MHz, $CDCl_3$) δ = 8.84 (d, J = 1.5 Hz, 1H), 8.63 – 8.58 (m, 1H), 8.09 (d, J = 11.1 Hz, 1H), 8.02 (d, J = 11.3 Hz, 1H), 7.87 (d, J = 8.0 Hz, 1H), 7.76 – 7.72 (m, 1H), 7.69 (dd, J = 7.6, 1.0 Hz, 1H), 7.62 (dd, J = 8.0, 1.6 Hz, 1H), 7.51 – 7.42 (m, 4H), 7.38 (td, J = 7.4, 1.7 Hz, 1H), 7.31 – 7.23 (m, 6H) ppm.

^{13}C NMR (126 MHz, $CDCl_3$) δ = 144.0 (1Cq), 143.2 (1CH), 142.9 (1CH), 138.9 (1Cq), 138.8 (1Cq), 138.7 (1Cq), 133.4 (1CH), 131.6 (2CH), 131.1 (br, 1CH-B), 130.7 (br, 1CH-B), 129.90 (1CH), 129.88 (1CH), 129.85 (q, $^{2}J_{C\text{–}F}$ = 32.8 Hz, 1Cq), 129.7 (1Cq), 129.6 (1CH), 129.3 (1CH), 129.0 (1Cq), 127.5 (1CH), 127.0 (1Cq), 126.6 (1CH), 125.3 (q, $^{3}J_{C\text{–}F}$ = 3.8 Hz, 2CH), 124.2 (1CH), 124.0 (q, $^{1}J_{C\text{–}F}$ = 272.1 Hz, 1Cq), 123.1 (1CH), 121.9 (1CH), 121.4 (1CH), 121.3 (1Cq), 92.0 (1Cq), 91.3 (1Cq) ppm.

^{11}B NMR (161 MHz, $CDCl_3$) δ = 31.22 ppm.

^{19}F NMR (377 MHz, $CDCl_3$) δ = -62.85 ppm.

IR (ATR) $\tilde{\nu}$ = 1590, 1320, 1166, 1101, 1065, 1015, 949, 908, 836, 811, 754, 744 cm^{-1}.

HRMS calcd *m/z* for $C_{31}H_{19}BF_3NNa$ $[M+Na]^+$: 496.1460; found (ESI): 496.1455

M. p. = 130 °C

2.4. Au-catalyzed hydroarylation reaction towards azabora[5]helicenes **1a-j**, **1b(BPh)**, **1b(NMe)** and **2b-h**

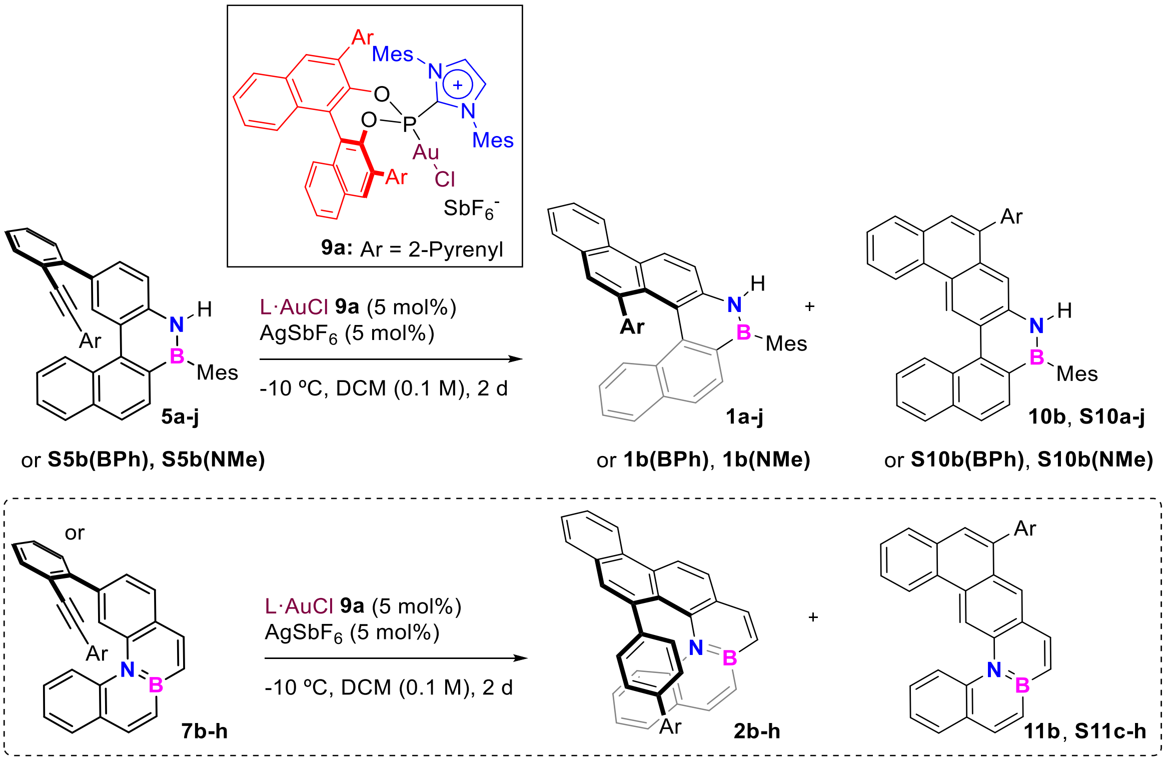

1a, S10a: Ar=Ph
1b, 10b, 1b(BPh), S10b(BPh), 1b(NMe), S10b(NMe), 2b, 11b: Ar=*p*-tol
1c, S10c, 2c, S11c: Ar=*p*-(MeO)Ph
1d, S10d1: Ar=*p*-(PhO)Ph
1e, S10e, 2e, S11e: Ar=*p*-(F)Ph
1f, S10f: Ar=*p*-(TMS)Ph
1g, S10g: Ar=*p*-(Cl)Ph
1h, S10h, 2h, S11h: Ar=*p*-(CF_3)Ph
1i, S10i: Ar=*o*-(MeO)Ph
1j, S10j: Ar=*m*-(MeO)Ph

Scheme S5 *Synthesis of azabora[5]helicenes with chiral gold catalyst* ***9a****.*

General protocol. In a Schlenk flask equipped with a stirring bar, the respective helicene precursors (25 µmol, 1.0 equiv.) and the chiral gold catalyst **9a** (1.8 mg, 5.0 mol%) were thoroughly dried under vacuo for 30 min. The mixture was dissolved in anhydrous DCM (0.1M, 0.25 mL) and cooled to the desired temperature (-10 °C, unless otherwise stated). A freshly prepared solution of $AgSbF_6$ (0.05 M in DCM, 25 µL, 5.0 mol%) was added dropwise. The reaction mixture was stirred for 48 h, then filtered through a plug of silica using DCM as eluent. The solvent was removed under reduced pressure and purification by preparative HPLC afforded the isolated helicenes.

For enantiomeric excess (ee) determination, racemic reactions were performed with achiral gold catalyst Au(PPh_3)Cl[18] under the same procedure except the reaction were perfromed at room temperature.

Azabora[5]helicene (+)-1a

The product was obtained following the general procedure from **5a** (13.1 mg, 25 µmol). NMR analysis of the crude mixture showed a regioselectivity of 88:12 (**1a**:**S10a**). The product was isolated as a white solid (10.5 mg, 20 µmol, 80%) with **93% *ee*** after purification by HPLC (acetonitrile:H_2O 95:5→100:0 over 5 min, flow rate 1.0 mL/min at 295 K).

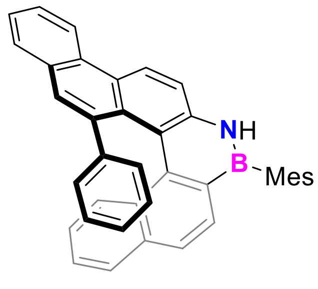

^{1}**H NMR** (500 MHz, CD_2Cl_2, –35 °C) *δ* = 8.88 (d, *J* = 8.9 Hz, 1H), 8.78 (d, *J* = 8.3 Hz, 1H), 8.13 (s, 1H), 7.95 (d, *J* = 7.5 Hz, 1H), 7.75 (d, *J* = 8.6 Hz, 1H), 7.72 (ddd, *J* = 8.3, 7.0, 1.4 Hz, 1H), 7.62 (ddd, *J* = 7.9, 7.0, 0.8 Hz, 1H), 7.54 (d, *J* = 7.8 Hz, 1H), 7.52 – 7.48 (m, 2H), 7.36 (ddd, *J* = 8.0, 6.8, 1.1 Hz, 1H), 7.34 (d, *J* = 8.0 Hz, 1H), 7.31 (d, *J* = 8.2 Hz, 1H), 7.09 (ddd, *J* = 8.3, 6.8, 1.4 Hz, 1H), 6.96 (s, 1H), 6.92 (s, 1H), 6.68 (td, *J* = 7.5, 1.1 Hz, 1H), 6.62 (tt, *J* = 7.4, 1.2 Hz, 1H), 6.55 (d, *J* = 7.6 Hz, 1H), 6.31 (td, *J* = 7.6, 1.1 Hz, 1H), 5.95 (d, *J* = 7.8 Hz, 1H), 2.34 (s, 3H), 2.27 (s, 3H), 2.03 (s, 3H) ppm.

^{1}**H NMR** (300 MHz, CD_2Cl_2) *δ* = 8.89 (d, *J* = 8.9 Hz, 1H), 8.79 (d, *J* = 8.3 Hz, 1H), 8.06 (s, 1H), 7.96 (d, *J* = 7.8 Hz, 1H), 7.78 – 7.69 (m, 2H), 7.63 (t, *J* = 7.1 Hz, 1H), 7.59 – 7.48 (m, 3H), 7.43 – 7.30 (m, 3H), 7.11 (t, *J* = 7.8 Hz, 1H), 6.99 (s, 1H), 6.94 (s, 1H), 6.68 – 6.61 (m, 1H), 6.78 – 5.88 (m, 4H), 2.37 (s, 3H), 2.31 (s, 3H), 2.07 (s, 3H) ppm.

^{13}**C NMR** (126 MHz, CD_2Cl_2, –35 °C) *δ* = 141.7 (1Cq), 140.5 (1Cq), 140.1 (1Cq), 139.4 (1Cq), 139.1 (1Cq), 138.8 (1Cq), 137.6 (1Cq), 135.8 (br, 1Cq-B), 133.9 (1Cq), 131.2 (br, 1Cq-B), 131.1 (1Cq), 130.3 (1Cq), 129.9 (1Cq), 129.82 (1CH), 129.76 (1Cq), 129.0 (1CH), 128.5 (1CH), 127.6 (1CH), 127.2 (1CH), 127.1 (1CH), 127.0 (1CH), 126.9 (1CH), 126.8 (1CH), 126.7 (1CH), 126.3 (1Cq), 126.1 (2CH), 126.0 (1CH), 125.9 (1CH), 125.6 (1CH), 125.0 (1CH), 124.9 (1CH), 122.9 (1CH), 122.5 (1CH), 118.7 (1CH), 116.9 (1Cq), 22.7 ($1CH_3$), 22.6 ($1CH_3$), 21.1 ($1CH_3$) ppm.

^{11}**B NMR** (161 MHz, CD_2Cl_2) *δ* = 40.26 ppm.

IR (ATR) $\tilde{\nu}$ = 3369, 2915, 2848, 1550, 1489, 1457, 1276, 1258, 750 cm^{-1}.

HRMS calcd *m/z* for $C_{39}H_{30}BNNa$ $[M+Na]^+$: 546.2370; found (ESI) 546.2352

$[\alpha]_{25}^{D}$: +615° (c = 0.07 in DCM) for **93% *ee***.

Azabora[5]helicene (+)-1b

The product was obtained following the general procedure from **5b** (13.0 mg, 25 µmol). NMR analysis of the crude mixture showed a regioselectivity of 85:15 (**1b**:**10b**). The product was isolated as a white solid (9.0 mg, 17 µmol, 70%) with **97% *ee*** after purification by HPLC (acetonitrile:H_2O 95:5→100:0 over 5 min, flow rate 1.0 mL/min at 295 K).

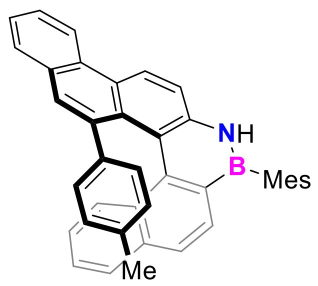

^{1}**H NMR** (600 MHz, CD_2Cl_2, –35 °C) *δ* = 8.86 (d, *J* = 9.2 Hz, 1H), 8.77 (d, *J* = 8.2 Hz, 1H), 8.11 (s, 1H), 7.93 (d, *J* = 7.8 Hz, 1H), 7.73 (d, *J* = 8.6 Hz, 1H), 7.70 (ddd, *J* = 8.3, 6.9, 1.4 Hz, 1H), 7.61 (ddd, *J* = 7.9, 6.9, 1.0 Hz, 1H), 7.54 (dd, *J* = 8.1, 0.8 Hz, 1H), 7.50 (s, 1H), 7.46 (d, *J* = 8.4 Hz, 1H), 7.36 – 7.32 (m, 3H), 7.06 (ddd, *J* = 8.3, 6.7, 1.3 Hz, 1H), 6.97 (s, 1H), 6.93 (s, 1H), 6.46 (d, *J* = 7.9 Hz, 1H), 6.41 (dd, *J* = 7.8, 2.0 Hz, 1H), 6.07 (d, *J* = 7.9 Hz, 1H), 5.80 (dd, *J* = 7.8, 2.0 Hz, 1H), 2.34 (s, 3H), 2.27 (s, 3H), 2.04 (s, 3H), 2.00 (s, 3H) ppm.

^{1}**H NMR** (300 MHz, CD_2Cl_2) *δ* = 8.88 (d, *J* = 8.9 Hz, 1H), 8.78 (d, *J* = 8.5 Hz, 1H), 8.04 (s, 1H), 7.95 (d, *J* = 8.0 Hz, 1H), 7.78 – 7.68 (m, 2H), 7.66 – 7.49 (m, 4H), 7.43 – 7.33 (m, 3H),

7.09 (ddd, J = 8.3, 6.9, 1.2 Hz, 1H), 6.99 (s, 1H), 6.95 (s, 1H), 6.12 (br. m, 4H), 2.38 (s, 3H), 2.31 (s, 3H), 2.07 (s, 3H), 2.03 (s, 3H) ppm.

^{13}C NMR (151 MHz, CD_2Cl_2, –35 °C) δ = 140.5 (1Cq), 140.0 (1Cq), 139.3 (1Cq), 139.0 (1Cq), 138.7 (1Cq), 138.6 (1Cq), 137.6 (1Cq), 135.9 (br, 1Cq-B), 134.4 (1Cq), 133.8 (1Cq), 131.1 (1Cq), 131.0 (br, 1Cq-B), 130.4 (1Cq), 130.0 (1Cq), 129.8 (1Cq), 129.3 (1CH), 129.0 (1CH), 128.3 (1CH), 127.5 (1CH), 127.4 (1CH), 127.0 (1CH), 126.9 (1CH), 126.8 (1CH), 126.6 (1CH), 126.5 (1CH), 126.30 (1Cq), 126.25 (1CH), 126.02 (1CH), 125.97 (1CH), 125.74 (1CH), 125.70 (1CH), 125.0 (1CH), 122.9 (1CH), 122.5 (1CH), 118.6 (1CH), 116.9 (1Cq), 22.65 ($1CH_3$), 22.57 ($1CH_3$), 21.1 ($1CH_3$), 20.4 ($1CH_3$) ppm.

^{11}B NMR (161 MHz, CD_2Cl_2) δ = 39.25 ppm.

IR (ATR) $\tilde{\nu}$ = 3375, 3043, 2915, 2853, 1608, 1550, 1490, 1457, 1411, 1329, 1245, 1043, 814 cm^{-1}.

HRMS calcd *m/z* for $C_{40}H_{32}BNNa$ $[M+Na]^+$: 560.2527; found (ESI) 560.2535

$[\alpha]_{25}^{D}$ **:** + 598° (c = 0.02 in DCM) for **97% *ee***.

Azabora[5]helicene (+)-1c

The product was obtained following the general procedure from **5c** (13.8 mg, 25 µmol). NMR analysis of the crude mixture showed a regioselectivity of 71:29 (**1c**:**S10c**). The product was isolated as a white solid (9.0 mg, 16 µmol, 65%) with **95% *ee*** after purification by HPLC (acetonitrile:H_2O 95:5→100:0 over 5 min, flow rate 1.0 mL/min at 295 K).

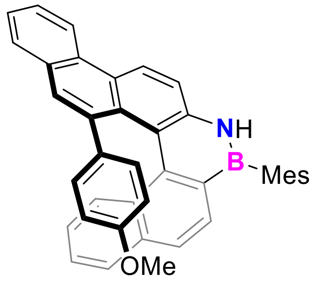

^{1}H NMR (600 MHz, CD_2Cl_2, –35 °C) δ = 8.86 (d, J = 9.2 Hz, 1H), 8.76 (d, J = 8.3 Hz, 1H), 8.13 (s, 1H), 7.93 (d, J = 7.5 Hz, 1H), 7.74 (d, J = 8.6 Hz, 1H), 7.70 (ddd, J = 8.4, 7.0, 1.4 Hz, 1H), 7.61 (ddd, J = 7.8, 7.1, 1.0 Hz, 1H), 7.58 (d, J = 8.1 Hz, 1H), 7.48 – 7.45 (m, 2H), 7.39 – 7.34 (m, 3H), 7.07 (ddd, J = 8.3, 6.8, 1.3 Hz, 1H), 6.96 (s, 1H), 6.93 (s, 1H), 6.47 (dd, J = 8.4, 2.3 Hz, 1H), 6.21 (dd, J = 8.4, 2.7 Hz, 1H), 5.86 (dd, J = 8.5, 2.4 Hz, 1H), 5.81 (dd, J = 8.5, 2.8 Hz, 1H), 3.56 (s, 3H), 2.34 (s, 3H), 2.27 (s, 3H), 2.04 (s, 3H) ppm.

^{1}H NMR (300 MHz, CD_2Cl_2) δ = 8.88 (d, J = 8.7 Hz, 1H), 8.78 (d, J = 8.9 Hz, 1H), 8.05 (s, 1H), 7.94 (d, J = 7.9 Hz, 1H), 7.77 – 7.67 (m, 2H), 7.66 – 7.57 (m, 2H), 7.55 – 7.48 (m, 2H), 7.45 – 7.34 (m, 3H), 7.14 – 7.06 (m, 1H), 6.99 (s, 1H), 6.95 (s, 1H), 6.00 (br. m, 4H), 3.59 (s, 3H), 2.37 (s, 3H), 2.31 (s, 3H), 2.07 (s, 3H) ppm.

^{13}C NMR (151 MHz, CD_2Cl_2, –35 °C) δ = 156.6 (1Cq), 140.5 (1Cq), 140.1 (1Cq), 139.3 (1Cq), 139.1 (1Cq), 138.3 (1Cq), 137.6 (1Cq), 135.8 (br, 1Cq-B), 134.4 (1Cq), 133.9 (1Cq), 131.1 (1Cq), 130.5 (1Cq), 130.1 (1Cq), 129.7 (1Cq), 129.14 (1CH), 129.11 (1CH), 128.3 (1CH), 128.0 (1CH), 127.6 (1CH), 127.05 (1CH), 127.03 (1CH), 126.9 (1CH), 126.6 (1CH), 126.5 (1CH), 126.3 (1Cq), 126.1 (1CH), 126.0 (1CH), 125.8 (1CH), 125.0 (1CH), 122.9 (1CH), 122.5 (1CH), 118.6 (1CH), 116.8 (1Cq), 112.1 (1CH), 111.1 (1CH), 55.1 ($1CH_3$), 22.7 ($1CH_3$), 22.6 ($1CH_3$), 21.1 ($1CH_3$) ppm. One carbon atom bonded to boron is not observed.

^{11}B NMR (161 MHz, CD_2Cl_2) δ = 39.54 ppm.

IR (ATR) $\tilde{\nu}$ = 3371, 3041, 2925, 1609, 1549, 1507, 1489, 1456, 1332, 1242, 1174, 1041, 818, 805, 745 cm^{-1}.

HRMS calcd *m/z* for $C_{40}H_{32}BNNaO$ $[M+Na]^+$: 576.2476; found (ESI) 576.2487

$[\alpha]_{25}^{D}$ **:** + 559° (c = 0.02 in DCM) for **95% *ee***.

Azabora[5]helicene (+)-1d

The product was obtained following the general procedure from **5d** (15.4 mg, 25 µmol). NMR analysis of the crude mixture showed a regioselectivity of 81:19 (**1d**:**S10d**). The product was isolated as a white solid (11.5 mg, 19 µmol, 75%) with **93% *ee*** after purification by HPLC (acetonitrile:H_2O 95:5→100:0 over 5 min, flow rate 1.0 mL/min at 295 K).

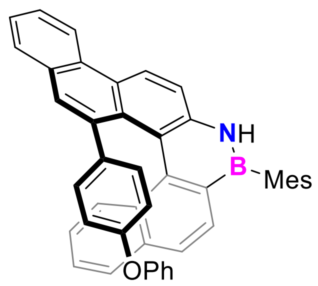

^{1}H NMR (500 MHz, THF, –35 °C) δ = 10.10 (s, 1H), 9.02 (d, *J* = 9.0 Hz, 1H), 8.90 (d, *J* = 8.2 Hz, 1H), 7.99 (d, *J* = 7.5 Hz, 1H), 7.91 (d, *J* = 8.7 Hz, 1H), 7.78 – 7.68 (m, 2H), 7.64 – 7.57 (m, 3H), 7.56 (s, 1H), 7.52 (d, *J* = 8.0 Hz, 1H), 7.43 – 7.36 (m, 3H), 7.16 (t, *J* = 7.1 Hz, 1H), 7.06 (t, *J* = 7.3 Hz, 1H), 6.92 (d, *J* = 10.3 Hz, 2H), 6.83 (d, *J* = 7.7 Hz, 2H), 6.62 (dd, *J* = 8.3, 2.4 Hz, 1H), 6.37 (dd, *J* = 8.5, 2.5 Hz, 1H), 6.05 (dd, *J* = 8.4, 2.5 Hz, 1H), 6.00 (dd, *J* = 8.4, 2.2 Hz, 1H), 2.34 (s, 3H), 2.24 (s, 3H), 2.08 (s, 3H) ppm.

^{1}H NMR (400 MHz, THF) δ 9.78 (s, 1H), 8.94 (d, *J* = 8.9 Hz, 1H), 8.83 (d, *J* = 8.2 Hz, 1H), 7.94 (d, *J* = 7.5 Hz, 1H), 7.89 (d, *J* = 8.6 Hz, 1H), 7.72 – 7.64 (m, 2H), 7.63 – 7.49 (m, 5H), 7.40 – 7.30 (m, 3H), 7.12 (ddd, *J* = 8.3, 6.9, 1.3 Hz, 1H), 7.03 (tt, *J* = 7.4, 1.2 Hz, 1H), 6.93 – 6.88 (m, 2H), 6.85 – 6.79 (m, 2H), 6.32 (br. m, 4H), 2.33 (s, 3H), 2.23 (s, 3H), 2.07 (s, 3H) ppm.

^{13}C NMR (126 MHz, THF, –35 °C) δ = 159.1 (1Cq), 154.9 (1Cq), 141.5 (1Cq), 141.0 (1Cq), 140.7 (2Cq), 139.4 (1Cq), 139.1 (1Cq), 137.9 (1Cq), 137.7 (br, 1Cq-B), 135.1 (1Cq), 132.8 (br, 1Cq-B), 132.4 (1Cq), 131.9 (1Cq), 131.4 (1Cq), 130.9 (1Cq), 130.5 (2CH), 130.4 (1CH), 129.7 (1CH), 129.5 (1CH), 128.8 (1CH), 128.5 (1CH), 128.1 (1CH), 128.0 (1CH), 127.9 (1CH), 127.8 (1CH), 127.5 (1Cq), 127.2 (1CH), 126.98 (1CH), 126.96 (1CH), 125.9 (1CH), 124.1 (1CH), 123.7 (1CH), 123.3 (2CH), 120.0 (1CH), 119.3 (1CH), 118.6 (2CH), 118.2 (1CH), 117.9 (1Cq), 23.4 ($1CH_3$), 23.2 ($1CH_3$), 21.7 ($1CH_3$) ppm.

^{11}B NMR (161 MHz, THF) δ = 38.26 ppm.

IR (ATR) $\tilde{\nu}$ = 3372, 3046, 2915, 2853, 1589, 1549, 1485, 1457, 1411, 1333, 1232, 748 cm^{-1}.

MS calcd *m/z* for $C_{45}H_{34}BNO$ $[M]^+$: 615.3; found (MALDI): 615.4

$[\alpha]_{25}^{D}$ **:** + 713° (c = 0.05 in DCM) for **93% *ee*.**

Azabora[5]helicene (+)-1e

The product was obtained following the general procedure from **5e** (13.5 mg, 25 µmol). NMR analysis of the crude mixture showed a regioselectivity of 90:10 (**1e**: **S10e**). The product was

isolated as a white solid (10.8 mg, 20 µmol, 80%) with **91% *ee*** after purification by HPLC (acetonitrile:H_2O 95:5→100:0 over 5 min, flow rate 1.0 mL/min at 295 K).

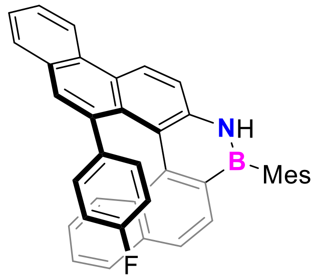

^{1}H NMR (600 MHz, CD_2Cl_2, –35 °C) δ = 8.87 (d, J = 8.7 Hz, 1H), 8.77 (d, J = 8.3 Hz, 1H), 8.14 (s, 1H), 7.94 (d, J = 7.7 Hz, 1H), 7.77 – 7.70 (m, 2H), 7.65 – 7.58 (m, 2H), 7.50 – 7.45 (m, 2H), 7.43 – 7.35 (m, 3H), 7.09 (t, J = 7.2 Hz, 1H), 6.97 (s, 1H), 6.93 (s, 1H), 6.55 – 6.50 (m, 1H), 6.39 (td, J = 8.8, 2.8 Hz, 1H), 6.01 (td, J = 8.6, 2.9 Hz, 1H), 5.95 – 5.90 (m, 1H), 2.34 (s, 3H), 2.26 (s, 3H), 2.03 (s, 3H) ppm.

^{1}H NMR (300 MHz, CD_2Cl_2) δ = 8.89 (d, J = 8.9 Hz, 1H), 8.79 (d, J = 8.2 Hz, 1H), 8.07 (s, 1H), 7.95 (d, J = 7.5 Hz, 1H), 7.78 – 7.70 (m, 2H), 7.67 – 7.60 (m, 2H), 7.55 – 7.48 (m, 2H), 7.46 – 7.35 (m, 3H), 7.12 (ddd, J = 8.2, 6.8, 1.3 Hz, 1H), 6.99 (s, 1H), 6.95 (s, 1H), 6.31 (br. m, 4H), 2.38 (s, 3H), 2.30 (s, 3H), 2.07 (s, 3H) ppm.

^{13}C NMR (151 MHz, CD_2Cl_2, –35 °C) δ = 160.1 (d, $^1J_{C\text{-}F}$ = 243.5 Hz, 1Cq), 140.4 (1Cq), 140.1 (1Cq), 139.2 (1Cq), 139.1 (1Cq), 137.9 (d, $^4J_{Cq\text{-}F}$ = 2.9 Hz, 1Cq), 137.7 (1Cq), 137.5 (1Cq), 135.7 (br, 1Cq-B), 133.8 (1Cq), 131.3 (br, 1Cq-B), 131.0 (1Cq), 130.3 (1Cq), 129.9 (1Cq), 129.73 (1Cq), 129.71 (1CH), 129.3 (1CH), 128.42 (1CH), 128.39 (d, $^3J_{C\text{-}F}$ = 9.5 Hz, 1CH), 127.7 (1CH), 127.5 (d, $^3J_{C\text{-}F}$ = 7.9 Hz, 1CH), 127.1 (1CH), 127.0 (1CH), 126.9 (1CH), 126.6 (1CH), 126.4 (1Cq), 126.22 (1CH), 126.15 (1CH), 125.9 (1CH), 125.2 (1CH), 123.0 (1CH), 122.5 (1CH), 118.8 (1CH), 116.7 (1Cq), 113.5 (d, $^2J_{C\text{-}F}$ = 21.1 Hz, 1CH), 112.3 (d, $^2J_{C\text{-}F}$ = 21.4 Hz, 1CH), 22.7 ($1CH_3$), 22.6 ($1CH_3$), 21.1 ($1CH_3$) ppm.

^{11}B NMR (161 MHz, CD_2Cl_2) δ = 39.54 ppm.

^{19}F NMR (282 MHz, CD_2Cl_2) δ = -118.83 ppm.

IR (ATR) $\tilde{\nu}$ = 3375, 3040, 2918, 2856, 1605, 1548, 1506, 1492, 1456, 1411, 1332, 1228, 1157, 1044, 820, 749 cm^{-1}.

HRMS calcd *m/z* for $C_{39}H_{30}BFN$ $[M+H]^+$: 542.2456; found (ESI): 542.2441

$[\alpha]_{25}^{D}$ **:** + 583° (c = 0.03 in DCM) for **91% *ee*.**

Azabora[5]helicene (+)-1f

The product was obtained following the general procedure from **5f** (14.9 mg, 25 µmol). NMR analysis of the crude mixture showed a regioselectivity of 94:6 (**1f**: **S10f**). The product was isolated as a white solid (11.4 mg, 19 µmol, 77%) with **99% *ee*** after purification by HPLC (acetonitrile:THF 95:5→85:15 over 20 min, flow rate 1.0 mL/min at 295 K).

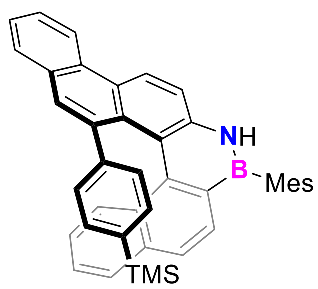

^{1}H NMR (500 MHz, CD_2Cl_2, –35 °C) δ = 8.88 (d, J = 9.0 Hz, 1H), 8.78 (d, J = 8.2 Hz, 1H), 8.13 (s, 1H), 7.94 (d, J = 7.7 Hz, 1H), 7.75 (d, J = 8.7 Hz, 1H), 7.72 (ddd, J = 8.3, 7.1, 1.4 Hz, 1H), 7.62 (ddd, J = 7.9, 7.2, 1.0 Hz, 1H), 7.53 (s, 1H), 7.48 (d, J = 7.9 Hz, 1H), 7.45 (d, J = 8.5 Hz, 1H), 7.36 – 7.31 (m, 2H), 7.25 (d, J = 8.1 Hz, 1H), 7.06 (ddd, J = 8.3, 6.7, 1.2 Hz, 1H), 6.98 (s, 1H), 6.92 (s, 1H), 6.81 (dd, J = 7.6, 0.8 Hz, 1H), 6.51 (dd, J = 7.6, 1.7 Hz, 1H), 6.39 (dd, J = 7.6, 1.0 Hz, 1H), 5.89 (dd, J = 7.6, 1.7 Hz, 1H), 2.34 (s, 3H), 2.28 (s, 3H), 2.04 (s, 3H), 0.12 (s, 9H) ppm.

^{1}H NMR (300 MHz, CD_2Cl_2) δ = 8.89 (d, J = 8.5 Hz, 1H), 8.79 (d, J = 8.2 Hz, 1H), 8.05 (s, 1H), 7.95 (d, J = 8.3 Hz, 1H), 7.79 – 7.69 (m, 2H), 7.62 (t, J = 7.4 Hz, 1H), 7.57 – 7.47 (m, 3H), 7.42 – 7.33 (m, 2H), 7.28 (d, J = 8.3 Hz, 1H), 7.09 (t, J = 7.7 Hz, 1H), 7.00 (s, 1H), 6.94 (s, 1H), 6.43 (br. m, 4H), 2.37 (s, 3H), 2.32 (s, 3H), 2.07 (s, 3H), 0.15 (s, 9H) ppm.

^{13}C NMR (126 MHz, CD_2Cl_2, –35 °C) δ = 141.8 (1Cq), 140.6 (1Cq), 140.1 (1Cq), 139.3 (1Cq), 139.1 (1Cq), 138.6 (1Cq), 137.6 (1Cq), 136.0 (1Cq), 135.9 (br, 1Cq-B), 133.8 (1Cq), 131.7 (1CH), 131.1 (br, 1Cq-B), 131.0 (1Cq), 130.5 (1CH), 130.2 (1Cq), 129.9 (1Cq), 129.8 (1Cq), 129.6 (1CH), 128.8 (1CH), 128.4 (1CH), 127.7 (1CH), 127.1 (1CH), 127.0 (1CH), 126.8 (1CH), 126.6 (1CH), 126.31 (1Cq), 126.30 (1CH), 126.26 (1CH), 126.1 (1CH), 126.0 (1CH), 125.1 (1CH), 124.9 (1CH), 122.9 (1CH), 122.5 (1CH), 118.7 (1CH), 116.9 (1Cq), 22.7 ($1CH_3$), 22.6 ($1CH_3$), 21.1 ($1CH_3$), -1.6 ($3CH_3$) ppm.

^{11}B NMR (161 MHz, CD_2Cl_2) δ = 38.79 ppm.

IR (ATR) $\tilde{\nu}$ = 3368, 2952, 1550, 1489, 1457, 1412, 1331, 1246, 1111, 1042, 839, 818, 749 cm^{-1}.

HRMS calcd *m/z* for $C_{42}H_{38}BNNaSi$ $[M+Na]^+$: 618.2766; found (ESI): 618.2752

$[\alpha]_{25}^{D}$ **:** + 385° (c = 0.03 in DCM) for **99% *ee*.**

Azabora[5]helicene (+)-1g

The product was obtained following the general procedure from **5g** (13.9 mg, 25 µmol) at 0 °C. NMR analysis of the crude mixture showed a regioselectivity of 90:10 (**1g**: **S10g**). The product was isolated as a white solid (11.1 mg, 20 µmol, 80%) with **88% *ee*** after purification by HPLC (acetonitrile:H_2O 90:10→100:0 over 10 min, flow rate 1.0 mL/min at 295 K).

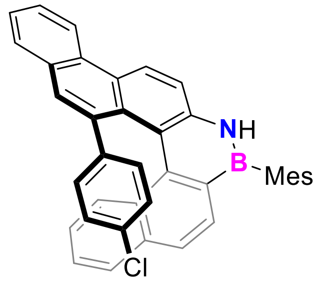

^{1}H NMR (500 MHz, CD_2Cl_2, –35 °C) δ = 8.88 (d, J = 9.1 Hz, 1H), 8.78 (d, J = 8.1 Hz, 1H), 8.15 (s, 1H), 7.94 (d, J = 7.3 Hz, 1H), 7.77 – 7.71 (m, 2H), 7.65 – 7.59 (m, 2H), 7.50 (s, 1H), 7.45 (d, J = 8.5 Hz, 1H), 7.44 – 7.35 (m, 3H), 7.09 (ddd, J = 8.2, 6.9, 1.1 Hz, 1H), 6.97 (s, 1H), 6.92 (s, 1H), 6.64 (dd, J = 8.3, 2.3 Hz, 1H), 6.49 (dd, J = 8.3, 2.4 Hz, 1H), 6.24 (dd, J = 8.3, 2.3 Hz, 1H), 5.88 (dd, J = 8.3, 2.2 Hz, 1H), 2.34 (s, 3H), 2.26 (s, 3H), 2.03 (s, 3H) ppm.

^{1}H NMR (300 MHz, CD_2Cl_2) δ = 8.89 (d, J = 9.0 Hz, 1H), 8.79 (d, J = 8.3 Hz, 1H), 8.08 (s, 1H), 7.95 (d, J = 7.8 Hz, 1H), 7.80 – 7.70 (m, 2H), 7.67 – 7.60 (m, 2H), 7.54 – 7.48 (m, 2H),

7.45 (s, 2H), 7.39 (ddd, J = 8.0, 6.9, 1.1 Hz, 1H), 7.11 (ddd, J = 8.2, 6.8, 1.2 Hz, 1H), 6.99 (s, 1H), 6.95 (s, 1H), 6.34 (br. m, 4H), 2.38 (s, 3H), 2.30 (s, 3H), 2.06 (s, 3H) ppm.

^{13}C NMR (126 MHz, CD_2Cl_2, –35 °C) δ = 140.4 (1Cq), 140.2 (1Cq), 140.0 (1Cq), 139.2 (1Cq), 139.1 (1Cq), 137.7 (1Cq), 137.3 (1Cq), 135.7 (br, 1Cq-B), 133.8 (1Cq), 131.3 (br, 1Cq-B), 130.9 (1Cq), 130.3 (1Cq), 130.2 (1Cq), 130.0 (1Cq), 129.7 (1CH), 129.4 (1Cq), 129.3 (1CH), 128.5 (1CH), 128.0 (1CH), 127.7 (1CH), 127.2 (1CH), 127.1 (1CH), 127.0 (1CH), 126.9 (1CH), 126.8 (1CH), 126.6 (1CH), 126.4 (1Cq), 126.23 (1CH), 126.17 (1CH), 125.8 (1CH), 125.5 (1CH), 125.2 (1CH), 123.0 (1CH), 122.5 (1CH), 118.9 (1CH), 116.6 (1Cq), 22.6 (2CH_3), 21.1 (1CH_3) ppm.

^{11}B NMR (161 MHz, CD_2Cl_2) δ = 38.96 ppm.

IR (ATR) $\tilde{\nu}$ = 3370, 2910, 1610, 1548, 1487, 1453, 1408, 1328, 1277, 1090, 977, 818, 748 cm^{-1}.

HRMS calcd *m/z* for $C_{39}H_{29}BClNNa$ $[M+Na]^+$: 580.1980; found (ESI): 580.1980

$[\alpha]_{25}^{D}$: + 543° (c = 0.06 in DCM) for **88% *ee*.**

Azabora[5]helicene (+)-1h

The product was obtained following the general procedure from **5h** (14.8 mg, 25 µmol). NMR analysis of the crude mixture showed a regioselectivity of 96:4 (**1h**: **S10h**). The product was isolated as a white solid (11.8 mg, 20 µmol, 80%) with **86% *ee*** after purification by HPLC (acetonitrile:H_2O 95:5→100:0 over 5 min, flow rate 1.0 mL/min at 295 K).

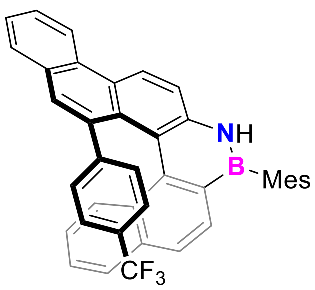

^{1}H NMR (500 MHz, CD_2Cl_2, –35 °C) δ = 8.90 (d, J = 9.2 Hz, 1H), 8.79 (d, J = 8.2 Hz, 1H), 8.17 (s, 1H), 7.96 (d, J = 7.9 Hz, 1H), 7.78 (d, J = 8.7 Hz, 1H), 7.75 (ddd, J = 8.4, 7.1, 1.4 Hz, 1H), 7.64 (ddd, J = 7.9, 7.0, 0.9 Hz, 1H), 7.56 (s, 1H), 7.53 (d, J = 7.8 Hz, 1H), 7.44 (d, J = 8.5 Hz, 1H), 7.38 – 7.34 (m, 2H), 7.31 (d, J = 8.1 Hz, 1H), 7.10 (ddd, J = 8.2, 6.8, 1.2 Hz, 1H), 6.97 (s, 1H), 6.95 – 6.91 (m, 2H), 6.68 (d, J = 8.0 Hz, 1H), 6.49 (dd, J = 8.1, 1.3 Hz, 1H), 6.04 (d, J = 8.2 Hz, 1H), 2.34 (s, 3H), 2.27 (s, 3H), 2.02 (s, 3H) ppm.

^{1}H NMR (300 MHz, CD_2Cl_2) δ = 8.91 (d, J = 9.0 Hz, 1H), 8.81 (d, J = 8.2 Hz, 1H), 8.10 (s, 1H), 7.97 (d, J = 7.5 Hz, 1H), 7.81 – 7.73 (m, 2H), 7.68 – 7.62 (m, 1H), 7.60 – 7.54 (m, 2H), 7.50 (d, J = 8.4 Hz, 1H), 7.45 – 7.32 (m, 3H), 7.13 (ddd, J = 8.4, 7.0, 1.2 Hz, 1H), 7.05 – 6.88 (m, 3H), 6.63 (br. m, J = 51.3 Hz, 2H), 6.09 (br. s, 1H), 2.37 (s, 3H), 2.31 (s, 3H), 2.05 (s, 3H) ppm.

^{13}C NMR (126 MHz, CD_2Cl_2, –35 °C) δ = 145.2 – 145.1 (m, 1Cq), 140.4 (1Cq), 140.1 (1Cq), 139.2 (1Cq), 139.0 (1Cq), 137.7 (1Cq), 137.0 (1Cq), 135.6 (br, 1Cq-B), 133.8 (1Cq), 131.3 (br, 1Cq-B), 130.8 (1Cq), 130.3 (1CH), 130.2 (1Cq), 130.0 (1Cq), 129.15 (1CH), 129.13 (1Cq), 128.6 (1CH), 127.8 (1CH), 127.3 (1CH), 127.1 (1CH), 126.9 (1CH), 126.71 (1CH), 126.66 (1CH), 126.4 (1Cq), 126.25 (1CH), 126.21 (1CH), 126.1 (1CH), 125.95 (1CH), 125.91 (q, $^2J_{C-F}$ = 31.7 Hz, 1Cq), 125.3 (1CH), 123.5 (q, $^3J_{C-F}$ = 3.6 Hz, 1CH), 123.14 (1CH), 123.11 (1Cq),

122.6 (1CH), 122.1 (q, $^{3}J_{C-F}$ = 3.3 Hz, 1CH), 118.9 (1CH), 116.4 (1Cq), 22.7 (1CH_3), 22.6 (1CH_3), 21.1 (1CH_3) ppm.

^{11}B NMR (161 MHz, CD_2Cl_2) δ = 40.39 ppm.

^{19}F NMR (377 MHz, $CDCl_3$) δ = -62.79 ppm.

IR (ATR) $\tilde{\nu}$ = 3372, 3045, 2916, 2856, 1611, 1550, 1489, 1458, 1414, 1323, 1165, 1121, 1110, 1066, 836, 749 cm^{-1}.

HRMS calcd *m/z* for $C_{40}H_{29}BF_3NNa$ [M+Na]$^+$: 614.2244; found (ESI): 614.2280

$[\alpha]_{25}^{D}$ **:** + 489° (c = 0.02 in DCM) for **86% *ee***.

Azabora[5]helicene (+)-1i

The product was obtained following the general procedure from **5i** (13.8 mg, 25 µmol) at 0 °C. NMR analysis of the crude mixture showed a regioselectivity of 65:35 (**1i**: **S10i**). The product was isolated as a white solid (7.0 mg, 12 µmol, 50%) with **91% *ee*** after purification by HPLC (acetonitrile:H_2O 95:5→100:0 over 5 min, flow rate 1.0 mL/min at 295 K).

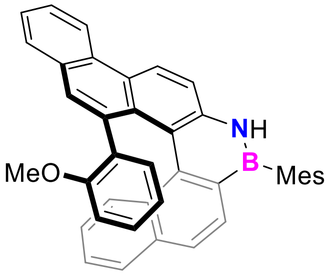

At room temperature two diastereoisomers are observed (dr=80:20, determined by ^{1}H NMR)

^{1}H NMR (400 MHz, $C_2D_2Cl_4$, 140 °C, signals coalesce to a single species) δ = 8.85 (d, *J* = 8.9 Hz, 1H), 8.80 (d, *J* = 7.9 Hz, 1H), 7.96 (dd, *J* = 7.8, 1.5 Hz, 1H), 7.86 (br.s, 1H), 7.78 – 7.46 (m, 7H), 7.42 – 7.37 (m, 2H), 7.16 (ddd, *J* = 8.5, 6.8, 1.4 Hz, 1H), 7.02 (s, 1H), 6.95 (s, 1H), 6.70 (t, *J* = 7.6 Hz, 1H), 6.54 – 6.03 (m, 3H), 3.60 – 2.77 (m, 3H), 2.45 – 2.41 (m, 6H), 2.01 (br.s, 3H) ppm.

^{13}C NMR (101 MHz, $CDCl_3$, 25 °C) of the major diastereosiomer: δ = 153.87 (1Cq), 141.06 (1Cq), 140.15 (1Cq), 139.26 (1Cq), 138.78 (1Cq), 137.78 (1Cq), 136.67 (1Cq), 133.94 (1Cq), 132.30 (1Cq), 131.96 (1Cq), 131.78 (1Cq), 131.47 (1Cq), 131.07 (1CH), 130.65 (1Cq), 129.41 (1CH), 128.78 (1CH), 128.61 (1CH), 128.00 (1CH), 127.51 (1CH), 127.16 (1CH), 127.01 (1CH), 126.84 (1CH), 126.71 (1CH), 126.04 (1CH), 125.91 (1CH), 125.85 (1CH), 125.82 (1Cq), 125.16 (1CH), 122.90 (1CH), 122.76 (1CH), 120.12 (1CH), 118.73 (1Cq), 118.54 (1CH), 109.48 (1CH), 54.42 (1CH_3), 23.48 (1CH_3), 22.97 (1CH_3), 21.40 (1CH_3) ppm. The carbon atoms bonded to boron are not observed.

^{11}B NMR (128 MHz, $CDCl_3$, 25 °C) δ = 39.87 ppm.

IR (ATR) $\tilde{\nu}$ = 3368, 3044, 2916, 1549, 1489, 1459, 1412, 1331, 1245, 1166, 1116, 1031, 948, 886, 849, 748 cm^{-1}.

HRMS calcd *m/z* for $C_{40}H_{32}BNNaO$ [M+Na]$^+$: 576.2476; found (ESI): 576.2481

$[\alpha]_{25}^{D}$ **:** + 495° (c = 0.06 in DCM) for **91% *ee***.

Azabora[5]helicene (+)-1j:

The product was obtained following the general procedure from **5j** (13.8 mg, 25 µmol). NMR analysis of the crude mixture showed a regioselectivity of 85:15 (**1j**:**S10j**). The product was isolated as a white solid (11.0 mg, 20 µmol, 80%) with **90% *ee*** after purification by HPLC (acetonitrile:H_2O 95:5→100:0 over 5 min, flow rate 1.0 mL/min at 295 K).

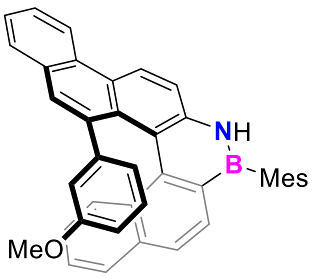

^{1}H NMR (600 MHz, $CDCl_3$, 60 °C) δ = 8.86 (d, J = 8.9 Hz, 1H), 8.77 (d, J = 8.3 Hz, 1H), 7.94 (d, J = 7.8 Hz, 1H), 7.86 (br. s, 1H), 7.71 (ddd, J = 8.4, 7.0, 1.4 Hz, 1H), 7.67 (d, J = 8.5 Hz, 1H), 7.63 – 7.57 (m, 3H), 7.55 (s, 1H), 7.48 (d, J = 8.1 Hz, 1H), 7.40 – 7.36 (m, 2H), 7.11 (t, J = 7.6 Hz, 1H), 7.00 (s, 1H), 6.94 (s, 1H), 6.23 (dd, J = 7.9, 2.0 Hz, 1H), 6.70 – 5.49 (m, 3H), 3.52 (br. s, 3H), 2.40 (s, 3H), 2.35 (s, 3H), 2.05 (s, 3H) ppm.

^{13}C NMR (126 MHz, $CDCl_3$, 60 °C) δ = 143.6 (1Cq), 140.7 (1Cq), 140.5 (1Cq), 140.2 (1Cq), 139.3 (1Cq), 137.9 (1Cq), 134.6 (1Cq), 131.6 (1Cq), 131.0 (1Cq), 130.6 (1Cq), 130.1 (1CH), 129.6 (1CH), 128.8 (1CH), 127.9 (1CH), 127.6 (1CH), 127.5 (1CH), 127.4 (1CH), 127.1 (1Cq), 127.0 (1CH), 126.3 (1CH), 126.23 (1CH), 126.20 (1CH), 124.9 (1CH), 123.2 (1CH), 122.7 (1CH), 118.7 (1CH), 117.8 (1Cq), 55.1 ($1CH_3$), 23.0 ($1CH_3$), 22.8 ($1CH_3$), 21.4 ($1CH_3$) ppm. The carbon atoms bonded to boron are not observed. and 4CH and 1Cq are suppresed due to rotation.

^{11}B NMR (161 MHz, CD_2Cl_2) δ = 39.72 ppm.

IR (ATR) $\tilde{\nu}$ = 3377, 2909, 1606, 1575, 1549, 1484, 1457, 1411, 1331, 1278, 1042, 747 cm^{-1}.

HRMS calcd *m/z* for $C_{40}H_{32}BNNaO$ [M+Na]$^+$: 576.2476; found (ESI) 576.2471

$[\alpha]_{25}^{D}$ **:** + 453° (c = 0.07 in DCM) for **90% *ee*.**

Azabora[5]helicene (+)-1b(BPh)

The product was obtained following the general procedure from precursor **S5b(BPh)** (12.4 mg, 25 µmol). NMR analysis of the crude mixture showed a regioselectivity of 87:13 (**1b(BPh)**: **S10b(BPh)**). The product was isolated as a white solid (10.0 mg, 20 µmol, 81%) with **98% *ee*** after purification by HPLC (acetonitrile:H_2O 95:5→100:0 over 5 min, flow rate 1.0 mL/min at 295 K).

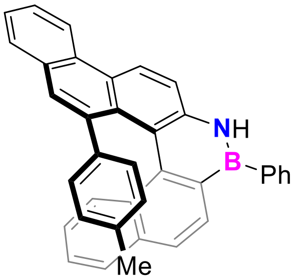

^{1}H NMR (600 MHz, CD_2Cl_2, –50 °C) δ = 8.90 (d, J = 8.5 Hz, 1H), 8.77 (d, J = 8.7 Hz, 1H), 8.21 (s, 1H), 7.96 – 7.90 (m, 4H), 7.81 (d, J = 8.6 Hz, 1H), 7.70 (t, J = 7.6 Hz, 1H), 7.61 (t, J = 7.4 Hz, 1H), 7.58 – 7.49 (m, 5H), 7.45 – 7.41 (m, 2H), 7.35 (t, J = 7.3 Hz, 1H), 7.05 (t, J = 7.6 Hz, 1H), 6.48 (d, J = 7.7 Hz, 1H), 6.39 (dd, J = 7.7, 1.7 Hz, 1H), 6.11 (d, J = 7.7 Hz, 1H), 5.82 (dd, J = 7.8, 1.8 Hz, 1H), 2.03 (s, 3H) ppm.

^{1}H NMR (300 MHz, CD_2Cl_2) δ = 8.91 (d, J = 9.0 Hz, 1H), 8.78 (d, J = 8.0 Hz, 1H), 8.14 (s, 1H), 7.99 – 7.88 (m, 4H), 7.80 (d, J = 8.7 Hz, 1H), 7.72 (ddd, J = 8.4, 7.0, 1.5 Hz, 1H), 7.65 –

7.43 (m, 8H), 7.37 (ddd, J = 8.2, 6.9, 1.3 Hz, 1H), 7.08 (ddd, J = 8.3, 6.8, 1.5 Hz, 1H), 6.34 (br. m, 4H), 2.06 (s, 3H) ppm.

^{13}C NMR (126 MHz, CD_2Cl_2, –35 °C) δ = 140.4 (1Cq), 139.0 (1Cq), 138.75 (1Cq), 138.74 (1Cq), 134.3 (1Cq), 133.7 (2CH), 133.4 (1Cq), 131.1 (1Cq), 130.5 (1Cq), 130.0 (1Cq), 129.8 (1Cq), 129.7 (1CH), 129.2 (1CH), 128.9 (1CH), 128.3 (1CH), 128.1 (2CH), 127.54 (1CH), 127.45 (1CH), 126.7 (1CH), 126.61 (1CH), 126.55 (1CH), 126.5 (1Cq), 126.4 (1CH), 126.08 (1CH), 126.06 (1CH), 125.7 (1CH), 125.6 (1CH), 124.9 (1CH), 123.2 (1CH), 122.5 (1CH), 118.6 (1CH), 116.8 (1Cq), 20.5 ($1CH_3$) ppm. The carbon atoms bonded to boron are not observed.

^{11}B NMR (161 MHz, CD_2Cl_2) δ = 37.31 ppm.

IR (ATR) $\tilde{\nu}$ = 3366, 3040, 2918, 2853, 1608, 1549, 1487, 1457, 1411, 1330, 1043, 814 cm^{-1}.

HRMS calcd *m/z* for $C_{37}H_{26}BNNa$ $[M+Na]^+$: 518.2057; found (ESI): 518.2066

$[\alpha]^{D}_{25}$: +531° (c = 0.02 in DCM) for **98% *ee***.

Azabora[5]helicene (+)-1b(NMe)

The product was obtained following the general procedure from **S5b(NMe)** (13.8 mg, 25 µmol). NMR analysis of the crude mixture showed a regioselectivity of 43:57 (**1b(NMe)**: **S10b(NMe)**). The product was isolated as a white solid (5.2 mg, 9 µmol, 38%) with **97% *ee*** after purification by HPLC (acetonitrile:H_2O 95:5→100:0 over 5 min, flow rate 1.0 mL/min at 295 K).

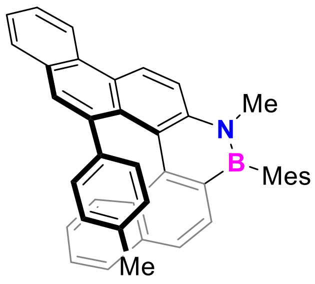

^{1}H NMR (500 MHz, CD_2Cl_2, –35 °C) δ = 8.98 (d, J = 9.5 Hz, 1H), 8.82 (d, J = 8.1 Hz, 1H), 8.13 (d, J = 9.1 Hz, 1H), 7.93 (d, J = 7.7 Hz, 1H), 7.72 (ddd, J = 8.3, 7.0, 1.4 Hz, 1H), 7.62 (ddd, J = 7.9, 7.1, 1.0 Hz, 1H), 7.53 (d, J = 7.9 Hz, 1H), 7.46 (s, 1H), 7.36 – 7.31 (m, 2H), 7.27 (d, J = 8.0 Hz, 1H), 7.14 (d, J = 8.1 Hz, 1H), 7.05 (ddd, J = 8.4, 6.8, 1.3 Hz, 1H), 6.98 (s, 1H), 6.95 (s, 1H), 6.45 (d, J = 8.0 Hz, 1H), 6.34 (dd, J = 7.8, 1.9 Hz, 1H), 6.08 (d, J = 8.0 Hz, 1H), 5.76 (dd, J = 7.9, 2.1 Hz, 1H), 3.69 (s, 3H), 2.37 (s, 3H), 2.16 (s, 3H), 1.98 (s, 3H), 1.95 (s, 3H) ppm.

^{13}C NMR (126 MHz, CD_2Cl_2, –35 °C) δ = 141.4 (1Cq), 139.7 (1Cq), 139.5 (1Cq), 139.1 (1Cq), 138.8 (1Cq), 138.6 (1Cq), 137.3 (br, 1Cq-B), 137.2 (1Cq), 134.4 (1Cq), 133.8 (1Cq), 131.2 (1Cq), 131.0 (br, 1Cq-B), 130.2 (1Cq), 129.8 (1Cq), 129.6 (1Cq), 129.4 (1CH), 129.1 (1CH), 128.3 (1CH), 127.6 (1CH), 127.4 (1CH), 127.01 (1CH), 126.95 (1CH), 126.9 (1CH), 126.7 (1CH), 126.6 (1CH), 126.4 (1CH), 126.1 (1CH), 125.82 (1CH), 125.78 (1CH), 125.7 (1Cq), 125.5 (1CH), 125.0 (1CH), 122.51 (1CH), 122.50 (1CH), 118.5 (1Cq), 114.9 (1CH), 36.4 ($1CH_3$), 22.3 ($1CH_3$), 22.2 ($1CH_3$), 21.2 ($1CH_3$), 20.4 ($1CH_3$) ppm.

^{11}B NMR (161 MHz, CD_2Cl_2) δ = 40.39 ppm.

IR (ATR) $\tilde{\nu}$ = 2915, 2853, 1541, 1477, 1456, 1394, 1325, 1233, 1040, 811, 746 cm^{-1}.

HRMS calcd *m/z* for $C_{41}H_{34}BN$ $[M]^+$: 551.2786; found (ESI): 551.2758

$[\alpha]^{D}_{25}$ **:** + 442° (c = 0.03 in DCM) for **97**% *ee*.

Azabora[5]helicene (+)-2b

The product was obtained following the general procedure from **7b** (10.5 mg, 25 µmol). NMR analysis of the crude mixture showed a regioselectivity of 42:58 (**2b**:**11b**). The product was isolated as a white solid (4.2 mg, 10 µmol, 40%) with **99% *ee*** after purification by HPLC (acetonitrile:H_2O 95:5→100:0 over 5 min, flow rate 1.0 mL/min at 295 K).

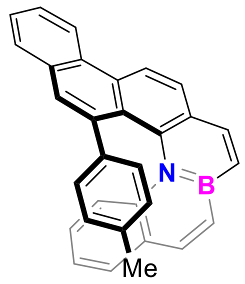

^{1}**H NMR** (500 MHz, CD_2Cl_2, –35 °C) δ = 8.83 (d, *J* = 8.2 Hz, 1H), 8.79 (d, *J* = 8.5 Hz, 1H), 8.22 (d, *J* = 11.0 Hz, 1H), 8.10 (d, *J* = 8.4 Hz, 1H), 7.91 (d, *J* = 7.7 Hz, 1H), 7.72 (t, *J* = 7.4 Hz, 1H), 7.65 (t, *J* = 7.3 Hz, 1H), 7.56 (d, *J* = 11.1 Hz, 1H), 7.43 (s, 1H), 7.36 (d, *J* = 11.0 Hz, 1H), 7.28 (d, *J* = 7.5 Hz, 1H), 7.07 (t, *J* = 7.2 Hz, 1H), 7.01 (d, *J* = 11.3 Hz, 1H), 6.88 (t, *J* = 7.7 Hz, 1H), 6.83 (d, *J* = 8.3 Hz, 1H), 6.68 (d, *J* = 7.8 Hz, 1H), 6.42 (dd, *J* = 7.6, 1.7 Hz, 1H), 6.33 (d, *J* = 7.7 Hz, 1H), 5.65 (dd, *J* = 7.7, 1.5 Hz, 1H), 2.16 (s, 3H) ppm.

^{1}**H NMR** (300 MHz, CD_2Cl_2) δ = 8.89 – 8.76 (m, 2H), 8.23 (d, *J* = 11.4 Hz, 1H), 8.10 (d, *J* = 8.5 Hz, 1H), 7.93 (d, *J* = 7.3 Hz, 1H), 7.78 – 7.62 (m, 2H), 7.58 (d, *J* = 11.1 Hz, 1H), 7.44 (s, 1H), 7.37 (d, *J* = 10.9 Hz, 1H), 7.30 (d, *J* = 7.8 Hz, 1H), 7.13 – 6.99 (m, 2H), 6.96 – 6.84 (m, 2H), 6.57 (br. m, 3H), 5.73 (br. s, 1H), 2.19 (s, 3H) ppm.

^{13}**C NMR** (126 MHz, CD_2Cl_2 , –35 °C) δ = 143.1 (1CH), 142.7 (1CH), 139.1 (1Cq), 137.0 (1Cq), 136.9 (1Cq), 135.3 (1Cq), 134.8 (1Cq), 131.7 (1Cq), 130.6 (1Cq), 129.6 (1Cq), 129.3 (1CH), 128.9 (1CH), 128.4 (1CH), 128.3 (1Cq), 127.9 (1CH), 127.6 (1CH), 127.5 (1Cq), 127.4 (1CH), 127.0 (1CH), 126.7 (1CH), 126.6 (1CH), 126.1 (1CH), 124.9 (1CH), 123.3 (1CH), 122.6 (1Cq), 121.9 (1CH), 119.5 (1CH), 118.7 (1CH), 20.7 ($1CH_3$) ppm. The carbon atoms bonded to boron are not observed.

^{11}**B NMR** (161 MHz, $CDCl_3$) δ = 30.76 ppm.

IR (ATR) $\tilde{\nu}$ = 3015, 1591, 1431, 1341, 1281, 1213, 827, 816, 798, 753 cm^{-1}.

HRMS calcd *m/z* for $C_{31}H_{22}BNNa$ $[M+Na]^+$: 442.1743; found (ESI): 442.1731

$[\alpha]^{D}_{25}$ **:** + 311° (c = 0.07 in DCM) for **99% *ee*.**

Azabora[5]helicene (+)-2c

The product was obtained following the general procedure from **7c** (10.9 mg, 25 µmol). NMR analysis of the crude mixture showed a regioselectivity of 13:87 (**2c**:**S11c**). The product was isolated as a white solid (1.1 mg, 3 µmol, 10%) with **84% *ee*** after purification by HPLC (acetonitrile:H_2O 90:10→100:0 over 20 min, flow rate 1.0 mL/min at 295 K).

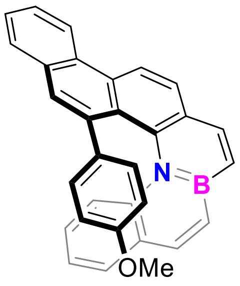

^{1}H NMR (600 MHz, $CDCl_3$, –35 °C) δ = 8.84 (d, J = 7.9 Hz, 1H), 8.78 (d, J = 8.3 Hz, 1H), 8.21 (d, J = 10.8 Hz, 1H), 8.11 – 8.07 (m, 1H), 7.91 (d, J = 7.7 Hz, 1H), 7.74 (t, J = 7.5 Hz, 1H), 7.67 (t, J = 7.3 Hz, 1H), 7.61 (d, J = 11.1 Hz, 1H), 7.42 (s, 1H), 7.37 (d, J = 10.7 Hz, 1H), 7.32 (d, J = 7.5 Hz, 1H), 7.08 (t, J = 7.2 Hz, 1H), 7.04 (d, J = 11.1 Hz, 1H), 6.91 (t, J = 7.2 Hz, 1H), 6.87 (d, J = 8.2 Hz, 1H), 6.50 (d, J = 8.2 Hz, 1H), 6.44 (d, J = 8.4 Hz, 1H), 6.14 (d, J = 8.6 Hz, 1H), 5.77 (d, J = 8.6 Hz, 1H), 3.72 (s, 3H) ppm.

^{13}C NMR (151 MHz, $CDCl_3$, –35 °C) δ = 157.4 (1Cq), 143.1 (1CH), 142.9 (1CH), 139.2 (1Cq), 136.6 (1Cq), 134.8 (1Cq), 132.9 (1Cq), 131.7 (1Cq), 130.7 (1Cq), 129.64 (1Cq), 129.56 (1CH), 129.4 (1CH), 128.9 (1CH), 128.5 (1CH), 128.3 (1Cq), 127.9 (1CH), 127.48 (1Cq), 127.47 (1CH), 127.0 (1CH), 126.7 (1CH), 126.3 (1CH), 126.2 (1CH), 123.4 (1CH), 122.8 (1Cq), 122.0 (1CH), 119.6 (1CH), 118.8 (1CH), 112.9 (1CH), 112.1 (1CH), 55.5 ($1CH_3$) ppm. The carbon atoms bonded to boron are not observed.

^{11}B NMR (128 MHz, $CDCl_3$) δ = 31.17 ppm.

HRMS calcd *m/z* for $C_{31}H_{23}BNO$ $[M+H]^+$: 436.1868; found (APCI): 436.1868

Azabora[5]helicene (+)-2e

The product was obtained following the general procedure from **7e** (10.6 mg, 25 µmol). NMR analysis of the crude mixture showed a regioselectivity of 57:43 (**2e**: **S11e**). The product was isolated as a white solid (5.2 mg, 12 µmol, 50%) with **97% *ee*** after purification by HPLC (acetonitrile:H_2O 90:10→100:0 over 10 min, flow rate 1.0 mL/min at 295 K).

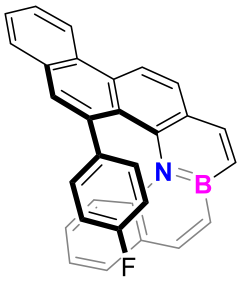

^{1}H NMR (500 MHz, $CDCl_3$, –35 °C) δ = 8.84 (d, J = 8.2 Hz, 1H), 8.79 (d, J = 8.6 Hz, 1H), 8.21 (d, J = 10.9 Hz, 1H), 8.09 (d, J = 8.4 Hz, 1H), 7.92 (dd, J = 7.8, 1.1 Hz, 1H), 7.76 (ddd, J = 8.3, 7.0, 1.4 Hz, 1H), 7.68 (ddd, J = 7.9, 7.0, 1.0 Hz, 1H), 7.64 (d, J = 11.1 Hz, 1H), 7.40 (s, 1H), 7.38 (d, J = 10.9 Hz, 1H), 7.34 (dd, J = 7.7, 1.3 Hz, 1H), 7.10 (ddd, J = 7.9, 7.0, 1.2 Hz, 1H), 7.06 (d, J = 11.0 Hz, 1H), 6.93 (ddd, J = 8.4, 6.8, 1.5 Hz, 1H), 6.87 (d, J = 8.4 Hz, 1H), 6.59 (td, J = 8.7, 2.7 Hz, 1H), 6.55 – 6.50 (m, 1H), 6.29 (td, J = 8.7, 2.7 Hz, 1H), 5.82 – 5.78 (m, 1H) ppm.

^{1}H NMR (300 MHz, $CDCl_3$) δ = 8.83 (d, J = 8.2 Hz, 1H), 8.78 (d, J = 8.6 Hz, 1H), 8.20 (d, J = 11.1 Hz, 1H), 8.08 (d, J = 8.5 Hz, 1H), 7.91 (dd, J = 7.8, 1.5 Hz, 1H), 7.74 (ddd, J = 8.4, 7.0, 1.5 Hz, 1H), 7.70 – 7.60 (m, 2H), 7.40 – 7.27 (m, 3H), 7.13 – 7.03 (m, 2H), 6.96 – 6.86 (m, 2H), 6.55 (br. s, 2H), 6.30 (br. s, 1H), 5.83 (br. s, 1H) ppm.

^{13}C NMR (126 MHz, $CDCl_3$, –35 °C) δ = 161.0 (d, $^{1}J_{C\text{-}F}$ = 244.4 Hz, 1Cq), 143.2 (1CH), 142.9 (1CH), 139.1 (1Cq), 136.1 (d, $^{4}J_{Cq\text{-}F}$ = 3.2 Hz, 1Cq), 135.9 (1Cq), 134.6 (1Cq), 131.9 (br, 1CH-B), 131.5 (1Cq), 130.6 (1Cq), 129.8 (1Cq), 129.5 (1CH), 129.3 (1CH), 128.8 (br, 1CH-B), 128.6 (1CH), 128.4 (1Cq), 128.2 (d, $^{3}J_{C\text{-}F}$ = 8.3 Hz, 1CH), 127.6 (1CH), 127.5 (1Cq), 127.2 (1CH), 127.0 (1CH), 126.7 (d, $^{3}J_{C\text{-}F}$ = 8.0 Hz, 1CH), 126.3 (1CH), 123.4 (1CH), 122.5 (1Cq), 122.1 (1CH), 119.8 (1CH), 118.9 (1CH), 114.0 (d, $^{2}J_{C\text{-}F}$ = 21.2 Hz, 1CH), 113.8 (d, $^{2}J_{C\text{-}F}$ = 21.6 Hz, 1CH) ppm.

^{11}B NMR (161 MHz, $CDCl_3$) δ = 30.64 ppm.

^{19}**F NMR** (282 MHz, $CDCl_3$) δ = -117.53 ppm.

IR (ATR) $\tilde{\nu}$ = 3019, 1593, 1555, 1532, 1505, 1482, 1431, 1342, 1282, 1219, 1156, 828, 799, 754, 728 cm^{-1}.

HRMS calcd *m/z* for $C_{30}H_{20}BFN$ $[M+H]^+$: 424.1673; found (ESI): 424.1662

$[\alpha]_{25}^{D}$ **:** + 916° (c = 0.06 M in DCM) for **97% *ee.***

Azabora[5]helicene (+)-2h

The product was obtained following the general procedure from **7h** (11.8 mg, 25 µmol). NMR analysis of the crude mixture showed a regioselectivity of 84:16 (**2h**: **S11h**). The product was isolated as a white solid (3.6 mg, 7 µmol, 30%) with **88% *ee*** after purification by HPLC (acetonitrile:H_2O 90:10→100:0 over 10 min, flow rate 1.0 mL/min at 295 K).

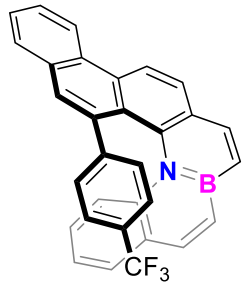

^{1}**H NMR** (500 MHz, $CDCl_3$, –35 °C) δ = 8.86 (d, *J* = 8.2 Hz, 1H), 8.81 (d, *J* = 8.6 Hz, 1H), 8.21 (d, *J* = 11.0 Hz, 1H), 8.11 (d, *J* = 8.2 Hz, 1H), 7.94 (d, *J* = 7.9 Hz, 1H), 7.78 (t, *J* = 7.7 Hz, 1H), 7.70 (t, *J* = 7.3 Hz, 1H), 7.52 (d, *J* = 11.2 Hz, 1H), 7.48 (s, 1H), 7.38 (d, *J* = 10.9 Hz, 1H), 7.28 – 7.27 (m, 1H), 7.14 (d, *J* = 8.0 Hz, 1H), 7.09 (t, *J* = 7.2 Hz, 1H), 7.04 (d, *J* = 11.1 Hz, 1H), 6.96 – 6.91 (m, 1H), 6.87 (d, *J* = 8.4 Hz, 1H), 6.82 (d, *J* = 8.2 Hz, 1H), 6.68 (d, *J* = 8.0 Hz, 1H), 5.94 (d, *J* = 8.2 Hz, 1H) ppm.

^{1}**H NMR** (300 MHz, $CDCl_3$) δ = 8.85 (d, *J* = 8.6 Hz, 1H), 8.80 (d, *J* = 8.5 Hz, 1H), 8.21 (d, *J* = 11.0 Hz, 1H), 8.10 (d, *J* = 8.5 Hz, 1H), 7.93 (dd, *J* = 7.8, 1.1 Hz, 1H), 7.77 (ddd, *J* = 8.4, 7.2, 1.5 Hz, 1H), 7.68 (ddd, *J* = 8.0, 7.0, 0.9 Hz, 1H), 7.53 (d, *J* = 11.4 Hz, 1H), 7.47 (s, 1H), 7.39 (d, *J* = 11.0 Hz, 1H), 7.31 – 7.27 (m, 1H), 7.18 – 7.01 (m, 3H), 6.98 – 6.80 (m, 3H), 6.69 (br. m, 1H), 5.97 (br. m, 1H) ppm.

^{13}**C NMR** (126 MHz, $CDCl_3$, –35 °C) δ = 143.3 (1CH), 143.1 (1Cq), 143.0 (1CH), 138.7 (1Cq), 135.6 (1Cq), 134.4 (1Cq), 131.3 (1Cq), 130.7 (1Cq), 130.1 (1Cq), 129.7 (1CH), 129.6 (1CH), 128.8 (1CH), 128.6 (1Cq), 127.9 (1CH), 127.5 (1Cq), 127.4 (1CH), 127.3 (1CH), 127.28 – 126.96 (m, 1Cq), 126.8 (1CH), 126.4 (1CH), 125.6 – 123.0 (m, 1Cq), 125.2 (1CH), 124.0 (q, $^{3}J_{C\text{–}F}$ = 3.6 Hz, 1CH), 123.8 (q, $^{3}J_{C\text{–}F}$ = 3.8 Hz, 1CH), 123.5 (1CH), 122.2 (1CH), 122.1 (1Cq), 120.1 (1CH), 119.0 (1CH) ppm. The carbon atoms bonded to boron are not observed.

^{11}**B NMR** (161 MHz, $CDCl_3$) δ = 30.76 ppm.

^{19}**F NMR** (282 MHz, $CDCl_3$) δ = -62.52 ppm.

IR (ATR) $\tilde{\nu}$ = 3021, 1593, 1323, 1281, 1164, 1120, 1066, 1015, 831, 756 cm^{-1}.

HRMS calcd *m/z* for $C_{31}H_{19}BF_3NNa$ $[M+Na]^+$: 496.1457; found (ESI): 496.2685

$[\alpha]_{25}^{D}$ **:** + 560° (c = 0.03 in DCM) for **88**% *ee*.

Compound **S10a**

Compound **S10a** was prepared following the general procedure from **5a** (13.1 mg, 25 µmol). NMR analysis of the crude mixture showed a regioselectivity of 88:12 (**1a**: **S10a)**. The product was isolated as a white solid (1.0 mg, 2 µmol, 7%) after purification by HPLC (acetonitrile:H_2O 95:5→100:0 over 5 min, flow rate 1.0 mL/min at 295 K).

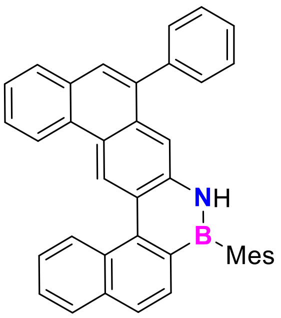

^{1}H NMR (300 MHz, $CDCl_3$) δ = 10.19 (s, 1H), 9.24 (d, J = 8.8 Hz, 1H), 8.79 (d, J = 8.1 Hz, 1H), 8.06 (d, J = 7.7 Hz, 1H), 7.93 (d, J = 7.9 Hz, 1H), 7.87 – 7.46 (m, 14H), 6.92 (s, 2H), 2.36 (s, 3H), 2.12 (s, 6H) ppm.

^{13}C NMR (126 MHz, $CDCl_3$) δ = 140.8 (1Cq), 140.4 (1Cq), 138.9 (1Cq), 138.2 (1Cq), 137.9 (1Cq), 137.8 (1Cq), 136.6 (1Cq), 131.4 (1CH), 131.2 (1Cq), 131.0 (1Cq), 130.8 (1Cq), 130.2 (1Cq), 130.1 (2CH), 129.1 (1CH), 129.0 (1CH), 128.7 (2CH), 128.5 (1CH), 128.0 (1CH), 127.8 (1CH), 127.28 (2CH), 127.26 (1CH), 127.24 (1CH), 127.0 (1CH), 126.4 (2CH), 125.2 (1CH), 125.1 (1Cq), 125.0 (1Cq), 123.6 (1Cq), 122.3 (1CH), 114.3 (1CH), 23.0 ($2CH_3$), 21.4 ($1CH_3$) ppm.

^{11}B NMR (161 MHz, $CDCl_3$) δ = 41.95 ppm.

IR (ATR) $\tilde{\nu}$ = 3364, 3051, 2918, 2851, 1614, 1550, 1469, 1431, 972, 748, 701 cm^{-1}.

HRMS calcd *m/z* for $C_{39}H_{31}BN$ $[M+H]^+$: 524.2551; found (ESI): 524.2550

Compound **10b**

Compound **10b** was prepared following the general procedure from **5b** (13.0 mg, 25 µmol). NMR analysis of the crude mixture showed a regioselectivity of 85:15 (**1b**:**10b**). The product was isolated as a white solid (1.0 mg, 2 µmol, 8%) after purification by HPLC (acetonitrile:H_2O 95:5→100:0 over 5 min, flow rate 1.0 mL/min at 295 K).

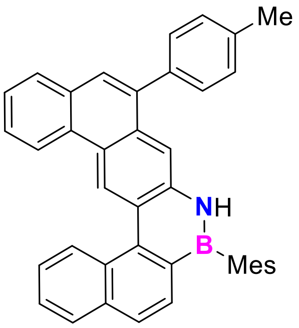

^{1}H NMR (400 MHz, CD_2Cl_2) δ = 10.19 (s, 1H), 9.25 (d, J = 8.5 Hz, 1H), 8.80 (d, J = 8.2 Hz, 1H), 8.07 (dd, J = 8.1, 1.4 Hz, 1H), 7.97 – 7.92 (m, 2H), 7.88 – 7.85 (m, 2H), 7.80 (ddd, J = 8.5, 6.9, 1.6 Hz, 1H), 7.75 – 7.70 (m, 3H), 7.67 – 7.60 (m, 2H), 7.56 (d, J = 8.0 Hz, 2H), 7.39 (d, J = 7.8 Hz, 2H), 6.92 (s, 2H), 2.49 (s, 3H), 2.35 (s, 3H), 2.11 (s, 6H) ppm.

^{13}C NMR (101 MHz, CD_2Cl_2) δ = 140.1 (2Cq), 138.9 (1Cq), 138.0 (1Cq), 137.8 (1Cq), 137.7 (1Cq), 137.6 (1Cq), 137.5 (1Cq), 136.5 (1Cq), 131.2 (1Cq), 131.1 (1CH), 131.0 (1Cq), 130.5 (1Cq), 130.1 (1Cq), 129.9 (2CH), 129.2 (2CH), 128.9 (1CH), 128.8 (1CH), 128.2 (1CH), 127.8 (1CH), 127.12 (3CH), 127.07 (1CH), 127.0 (1CH), 126.4 (1CH), 126.3 (1CH), 125.0 (1CH), 124.9 (1Cq), 123.4 (1Cq), 122.2 (1CH), 114.4 (1CH), 22.6 ($2CH_3$), 21.1 ($1CH_3$), 21.0 ($1CH_3$) ppm. The carbon atoms bonded to the boron were not observed.

^{11}B NMR (161 MHz, CD_2Cl_2) δ = 41.15 ppm.

IR (ATR) $\tilde{\nu}$ = 3364, 3025, 2912, 2851, 1610, 1550, 1469, 1428, 1336, 997, 820, 746 728 cm^{-1}.

HRMS calcd *m/z* for $C_{40}H_{33}BN$ $[M+H]^+$: 538.2707; found (ESI): 538.2709

M.p. = 186 °C

Compound **S10c**

Compound **S10c** was prepared following the general procedure from **5c** (13.8 mg, 25 µmol). NMR analysis of the crude mixture showed a regioselectivity of 71:29 (**1c**: **S10c**). The product was isolated as a white solid (2.2 mg, 4 µmol, 17%) after purification by HPLC (acetonitrile:H_2O 95:5→100:0 over 5 min, flow rate 1.0 mL/min at 295 K).

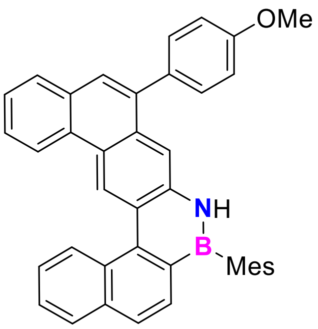

^{1}H NMR (400 MHz, CD_2Cl_2) δ = 10.19 (s, 1H), 9.25 (d, J = 8.5 Hz, 1H), 8.80 (d, J = 8.2 Hz, 1H), 8.07 (dd, J = 8.0, 1.4 Hz, 1H), 7.97 (br. s, 1H), 7.94 (dd, J = 7.8, 1.3 Hz, 1H), 7.88 – 7.85 (m, 2H), 7.80 (ddd, J = 8.5, 6.8, 1.6 Hz, 1H), 7.76 – 7.69 (m, 3H), 7.68 – 7.58 (m, 4H), 7.15 – 7.07 (m, 2H), 6.93 – 6.91 (m, 2H), 3.91 (s, 3H), 2.35 (s, 3H), 2.11 (s, 6H) ppm.

^{13}C NMR (101 MHz, CD_2Cl_2) δ = 159.4 (1Cq), 140.2 (3 Cq), 138.9 (1Cq), 138.0 (1Cq), 137.7 (1Cq), 137.4 (1Cq), 136.5 (1Cq), 132.9 (1Cq), 131.21 (1Cq), 131.15 (3CH), 130.5 (1Cq), 130.1 (1Cq), 128.9 (1CH), 128.8 (1CH), 128.2 (1CH), 127.8 (1CH), 127.1 (3CH), 127.02 (1CH), 126.97 (1CH), 126.4 (1CH), 126.3 (1CH), 125.01 (1CH), 124.95 (1Cq), 123.4 (1Cq), 122.2 (1CH), 114.4 (1CH), 114.0 (2CH), 55.5 ($1CH_3$), 22.6 ($2CH_3$), 21.0 ($1CH_3$) ppm. The carbon atoms bonded to the boron were not observed.

^{11}B NMR (161 MHz, CD_2Cl_2) δ = 41.02 ppm.

IR (ATR) $\tilde{\nu}$ = 3364, 3043, 2915, 2832, 1608, 1605, 1550, 1509, 1469, 1430, 1335, 1287, 1245, 1173, 1034, 827, 737 cm^{-1}.

HRMS: calcd *m/z* for $C_{40}H_{32}BNO$ $[M]^+$: 553.2572; found (ESI): 553.2570

Compound **S10d**

Compound **S10d** was prepared following the general procedure from **5d** (15.4 mg, 25 µmol). NMR analysis of the crude mixture showed a regioselectivity of 81:19 (**1d**: **S10d**). The product was isolated as a white solid (2.0 mg, 3 µmol, 10%) after purification by HPLC (acetonitrile:H_2O 95:5→100:0 over 5 min, flow rate 1.0 mL/min at 295 K).

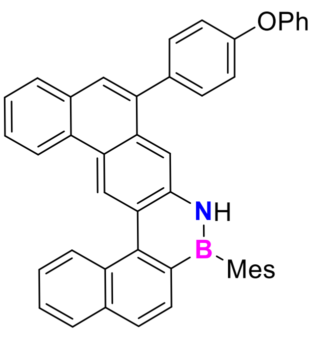

^{1}H NMR (300 MHz, CD_2Cl_2) δ = 10.20 (s, 1H), 9.25 (d, J = 8.4 Hz, 1H), 8.81 (d, J = 8.2 Hz, 1H), 8.08 (d, J = 7.9 Hz, 1H), 8.00 (br. s, 1H), 7.95 (d, J = 8.1 Hz, 1H), 7.90 – 7.85 (m, 2H), 7.81 (ddd, J = 8.5, 6.8, 1.5 Hz, 1H), 7.77 – 7.60 (m, 7H), 7.45 – 7.37 (m, 2H), 7.24 – 7.12 (m, 5H), 6.93 (s, 2H), 2.36 (s, 3H), 2.12 (s, 6H) ppm.

^{13}C NMR (101 MHz, CD_2Cl_2) δ = 157.6 (1Cq), 157.4 (1Cq), 140.5 (2Cq), 139.3 (1Cq), 138.4 (1Cq), 138.0 (1Cq), 137.5 (1Cq), 136.9 (1Cq), 135.9 (1Cq), 131.8 (2CH), 131.49 (1CH), 131.46 (1Cq), 131.3 (1Cq), 130.9 (1Cq), 130.4 (1Cq), 130.3 (2CH), 129.3 (1CH), 129.2 (1CH), 128.7 (1CH), 128.2 (1CH), 127.51 (2CH), 127.48 (2CH), 127.3 (1CH), 126.8 (1CH), 126.7 (1CH), 125.4 (1CH), 125.3 (1Cq), 123.9 (1CH), 123.8 (1Cq), 122.6 (1CH), 119.4 (2CH), 119.2 (2CH), 114.6 (1CH), 22.9 ($2CH_3$), 21.4 ($1CH_3$) ppm. The carbon atoms bonded to the boron were not observed.

^{11}B NMR (161 MHz, CD_2Cl_2) δ = 40.99 ppm.

IR (ATR) $\tilde{\nu}$ = 3369, 3040, 2912, 2853, 1618, 1608, 1588, 1550, 1503, 1488, 1470, 1431, 1236 cm^{-1}.

HRMS calcd *m/z* for $C_{45}H_{35}BNO$ $[M+H]^+$: 616.2814; found (ESI): 616.2796

Compound **S10e**

Compound **S10e** was prepared following the general procedure from **5e** (13.5 mg, 25 µmol). NMR analysis of the crude mixture showed a regioselectivity of 90:10 (**1e**: **S10e**). The product was isolated as a white solid (1.0 mg, 2 µmol, 7%) after purification by HPLC (acetonitrile:H_2O 95:5→100:0 over 5 min, flow rate 1.0 mL/min at 295 K).

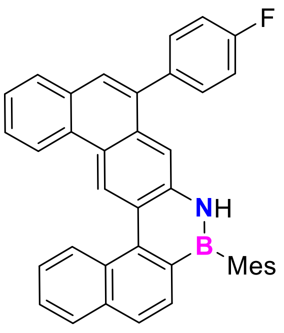

^{1}H NMR (500 MHz, CD_2Cl_2) δ = 10.20 (s, 1H), 9.24 (d, J = 8.5 Hz, 1H), 8.81 (d, J = 8.3 Hz, 1H), 8.08 (dd, J = 8.0, 1.0 Hz, 1H), 7.96 (br. s, 1H), 7.95 (dd, J = 7.9, 0.9 Hz, 1H), 7.87 (d, J = 8.1 Hz, 1H), 7.82 – 7.78 (m, 2H), 7.76 – 7.71 (m, 3H), 7.67 – 7.62 (m, 4H), 7.30 – 7.25 (m, 2H), 6.92 (s, 2H), 2.35 (s, 3H), 2.11 (s, 6H) ppm.

^{13}C NMR (126 MHz, CD_2Cl_2) δ = 162.9 (d, J = 245.8 Hz, 1Cq), 140.5 (2Cq), 139.3 (1Cq), 138.3 (1Cq), 138.0 (1Cq), 137.1 (1Cq), 137.0 (1Cq), 136.9 (1Cq), 132.12 (1CH), 132.05 (1CH), 131.5 (1CH), 131.3 (1Cq), 131.2 (1Cq), 131.0 (1Cq), 130.4 (1Cq), 129.3 (1CH), 129.2 (1CH), 128.9 (1CH), 128.1 (1CH), 127.6 (1CH), 127.54 (1CH), 127.47 (2CH), 127.3 (1CH), 126.8 (1CH), 126.7 (1CH), 125.4 (1CH), 125.2 (1Cq), 123.9 (1Cq), 122.6 (1CH), 115.8 (1CH), 115.7 (1CH), 114.5 (1CH), 22.9 ($2CH_3$), 21.4 ($1CH_3$) ppm. The carbon atoms bonded to boron were not observed.

^{11}B NMR (161 MHz, CD_2Cl_2) δ = 41.40 ppm.

^{19}F NMR (282 MHz, CD_2Cl_2) δ = -115.64 ppm.

IR (ATR) $\tilde{\nu}$ = 3373, 3045, 2920, 2854, 1608, 1550, 1507, 1470, 1431, 1226, 1157, 836, 750 cm^{-1}.

HRMS calcd *m/z* for $C_{39}H_{29}BFN$ $[M+H]^+$: 542.2456; found (ESI): 542.2437

Compound **S10i**

Compound **S10i** was prepared following the general procedure from **5i** (13.8 mg, 25 µmol) at 0 °C. NMR analysis of the crude mixture showed a regioselectivity of 65:35 (**1i**: **S10i**). The product was isolated as a white solid (4.1 mg, 7 µmol, 30%) after purification by HPLC (acetonitrile:H_2O 95:5→100:0 over 5 min, flow rate 1.0 mL/min at 295 K).

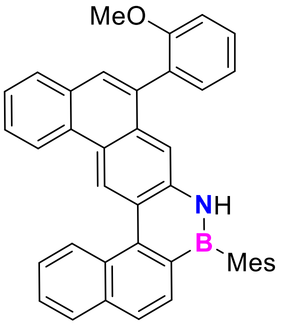

^{1}H NMR (300 MHz, CD_2Cl_2) δ = 10.17 (s, 1H), 9.25 (d, J = 8.8 Hz, 1H), 8.81 (d, J = 8.0 Hz, 1H), 8.07 (d, J = 8.2 Hz, 1H), 7.95 – 7.89 (m, 2H), 7.86 (d, J = 8.1 Hz, 1H), 7.83 – 7.58 (m, 6H), 7.54 – 7.47 (m, 2H), 7.44 (dd, J = 7.3, 1.6 Hz, 1H), 7.19 – 7.11 (m, 2H), 6.92 (s, 2H), 3.75 (s, 3H), 2.34 (s, 3H), 2.11 (s, 3H), 2.10 (s, 3H) ppm.

^{13}C NMR (126 MHz, $CDCl_3$) δ = 157.67 (1Cq), 140.42 (1Cq), 140.39 (1Cq), 138.85 (1Cq), 138.33 (1Cq), 137.74 (1Cq), 136.59 (1Cq), 134.84 (1Cq), 132.10 (1CH), 131.37 (1CH), 131.36 (1Cq), 131.28 (1Cq), 130.95 (1Cq), 130.20 (1Cq), 129.50 (1Cq), 129.49 (1CH), 129.08 (1CH), 128.95 (1CH), 128.90 (1CH), 128.06 (1CH), 127.28 (1CH), 127.23 (1CH), 127.11 (1CH), 127.07 (1CH), 126.94 (1CH), 126.27 (1CH), 126.15 (1CH), 125.04 (1CH), 124.80 (1Cq), 123.53 (1Cq), 122.33 (1CH), 120.96 (1CH), 114.32 (1CH), 111.30 (1CH), 55.87 (1CH_3), 23.02 (1CH_3), 22.97 (1CH_3), 21.41 (1CH_3) ppm. The carbon atoms bonded to boron were not observed.

^{11}B NMR (128 MHz, $CDCl_3$) δ = 41.08 ppm.

IR (ATR) $\tilde{\nu}$ = 3372, 3046, 2927, 2856, 1618, 1550, 1470, 1432, 1247, 1028 cm^{-1}.

HRMS: calcd m/z for $C_{40}H_{32}BNNaO$ $[M+Na]^+$: 576.2476; found (ESI) 576.2459

Compound **S10j**

Compound **S10j** was prepared following the general procedure from **5j** (13.8 mg, 25 µmol). NMR analysis of the crude mixture showed a regioselectivity of 85:15 (**1j**: **S10j**). The product was isolated as a white solid (1.2 mg, 2 µmol, 10 %) after purification by HPLC (acetonitrile:H_2O 95:5→100:0 over 5 min, flow rate 1.0 mL/min at 295 K).

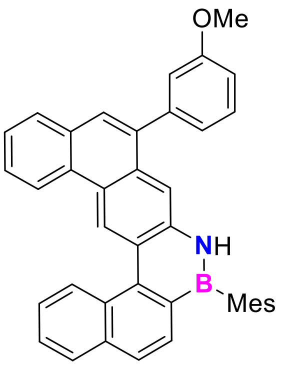

^{1}H NMR (500 MHz, CD_2Cl_2) δ = 10.19 (s, 1H), 9.24 (d, J = 8.5 Hz, 1H), 8.81 (d, J = 8.1 Hz, 1H), 8.07 (dd, J = 8.0, 1.5 Hz, 1H), 7.97 (br. s, 1H), 7.95 (dd, J = 7.7, 1.4 Hz, 1H), 7.88 – 7.85 (m, 2H), 7.80 (ddd, J = 8.5, 6.8, 1.5 Hz, 1H), 7.75 (s, 1H), 7.75 – 7.71 (m, 2H), 7.67 – 7.62 (m, 2H), 7.48 (ddd, J = 8.3, 7.5, 0.3 Hz, 1H), 7.25 (ddd, J = 7.5, 1.6, 1.0 Hz, 1H), 7.21 (dd, J = 2.5, 1.6 Hz, 1H), 7.04 (ddd, J = 8.3, 2.7, 1.0 Hz, 1H), 6.93 – 6.91 (m, 2H), 3.88 (s, 3H), 2.35 (s, 3H), 2.11 (s, 6H) ppm.

^{13}C NMR (126 MHz, CD_2Cl_2) δ = 160.3 (1Cq), 142.4 (1Cq), 140.5 (2Cq), 139.3 (1Cq), 138.3 (1Cq), 138.0 (2Cq), 136.9 (1Cq), 131.5 (1CH), 131.4 (1Cq), 131.1 (1Cq), 130.9 (1Cq), 130.4 (1Cq), 129.9 (1CH), 129.3 (1CH), 129.1 (1CH), 128.6 (1CH), 128.2 (1CH), 127.6 (1CH), 127.49 (1CH), 127.46 (2CH), 127.3 (1CH),

126.75 (1CH), 126.67 (1CH), 125.3 (1CH), 125.2 (1Cq), 123.8 (1Cq), 122.8 (1CH), 122.6 (1CH), 116.0 (1CH), 114.7 (1CH), 113.5 (1CH), 55.8 ($1CH_3$), 22.9 ($2CH_3$), 21.4 ($1CH_3$) ppm. The carbon atoms bonded to boron were not observed.

^{11}B NMR (161 MHz, CD_2Cl_2) δ = 40.26 ppm.

IR (ATR) $\tilde{\nu}$ = 3366, 2923, 2851, 1608, 1550, 1469, 1430, 1041, 991 cm^{-1}.

HRMS: calcd *m/z* for $C_{40}H_{32}BNNaO$ [M+Na]$^+$: 576.2476; found (ESI) 576.2454

Compound **S10b(BPh)**

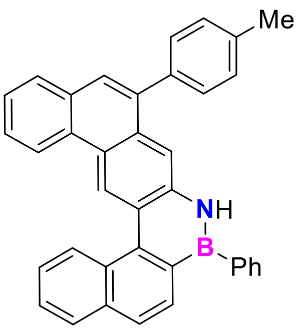

Compound **S10b(BPh)** was prepared following the general procedure from **S5b(BPh)** (12.4 mg, 25 μmol). NMR analysis of the crude mixture showed a regioselectivity of 87:13 (**1b(BPh)**: **S10b(BPh))**. The product was isolated as a white solid (1.0 mg, 2 μmol, 10 %) after purification by HPLC (acetonitrile:H_2O 95:5→100:0 over 5 min, flow rate 1.0 mL/min at 295 K).

^{1}H NMR (400 MHz, $CDCl_3$) δ = 10.14 (s, 1H), 9.20 (d, *J* = 8.4 Hz, 1H), 8.76 (d, *J* = 8.1 Hz, 1H), 8.21 (d, *J* = 8.2 Hz, 1H), 8.09 (dd, *J* = 8.1, 1.3 Hz, 1H), 7.96 – 7.89 (m, 3H), 7.85 (s, 1H), 7.82 – 7.68 (m, 6H), 7.61 (t, *J* = 7.3 Hz, 1H), 7.56 (d, *J* = 8.1 Hz, 2H), 7.53 – 7.46 (m, 3H), 7.40 (d, *J* = 7.9 Hz, 2H), 2.52 (s, 3H) ppm.

^{13}C NMR (101 MHz, $CDCl_3$) δ = 139.1 (1Cq), 138.8 (1Cq), 137.9 (1Cq), 137.8 (1Cq), 137.5 (1Cq), 136.4 (1Cq), 133.5 (2CH), 131.4 (1CH), 131.3 (1Cq), 131.2 (1Cq), 130.7 (1Cq), 130.15 (1Cq), 130.05 (2CH), 129.4 (2CH), 129.0 (1CH), 128.9 (1CH), 128.8 (1CH), 128.4 (1CH), 128.2 (2CH), 128.1 (1CH), 127.2 (1CH), 127.1 (1CH), 127.0 (1CH), 126.4 (2CH), 125.3 (1CH), 125.1 (1Cq), 123.5 (1Cq), 122.3 (1CH), 114.5 (1CH), 21.5 ($1CH_3$). The carbon atoms bonded to the boron were not observed.

^{11}B NMR (161 MHz, $CDCl_3$) δ = 38.43 ppm.

IR (ATR) $\tilde{\nu}$ = 3383, 3048, 3024, 2921, 2853, 1619, 1546, 1471, 1430, 1339, 821, 746, 704 cm^{-1}.

HRMS calcd *m/z* for $C_{37}H_{26}BNNa$ [M+Na]$^+$: 518.2057; found (ESI): 518.2049

Compound **S10b(NMe)**

Compound **S10b(NMe)** was prepared following the general procedure from **S5b(NMe)** (13.8 mg, 25 μmol). NMR analysis of the crude mixture showed a regioselectivity of 43:57 (**1b(NMe)**: **S10b(NMe))**. The product was isolated as a white solid (6.3 mg, 11 μmol, 46%) after purification by HPLC (acetonitrile:H_2O 95:5→100:0 over 5 min, flow rate 1.0 mL/min at 295 K).

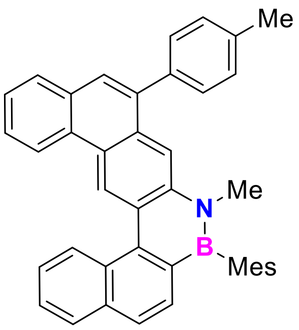

^{1}H NMR (500 MHz, CD_2Cl_2) δ = 10.12 (s, 1H), 9.12 (d, J = 8.5 Hz, 1H), 8.78 (d, J = 8.3 Hz, 1H), 8.25 (s, 1H), 8.04 (dd, J = 8.1, 1.1 Hz, 1H), 7.95 (dd, J = 8.0, 1.1 Hz, 1H), 7.81 – 7.74 (m, 3H), 7.73 – 7.68 (m, 2H), 7.65 – 7.60 (m, 3H), 7.42 (d, J = 8.0 Hz, 1H), 7.39 (d, J = 7.7 Hz, 2H), 6.93 (s, 2H), 3.43 (s, 3H), 2.47 (s, 3H), 2.34 (s, 3H), 1.98 (s, 6H) ppm.

^{13}C NMR (126 MHz, CD_2Cl_2) δ = 140.8 (1Cq), 139.5 (2Cq), 138.1 (1Cq), 137.54 (1Cq), 137.49 (1Cq), 137.44 (1Cq), 137.2 (1Cq), 137.1 (br, 1Cq-B), 136.1 (1Cq), 131.9 (br, 1Cq-B), 130.98 (1Cq), 130.96 (1CH), 130.3 (1Cq), 129.9 (1Cq), 129.7 (2CH), 129.4 (1Cq), 129.3 (2CH), 128.8 (1CH), 128.6 (1CH), 128.3 (1CH), 127.9 (1CH), 126.99 (2CH), 126.96 (1CH), 126.9 (1CH), 126.8 (1CH), 126.4 (1CH), 126.3 (1CH), 125.2 (1CH), 124.23 (1Cq), 124.17 (1Cq), 122.2 (1CH), 111.5 (1CH), 36.1 ($1CH_3$), 22.3 ($2CH_3$), 21.2 ($1CH_3$), 21.1 ($1CH_3$) ppm.

^{11}B NMR (161 MHz, CD_2Cl_2) δ = 40.37 ppm.

IR (ATR) $\tilde{\nu}$ = 2918, 1605, 1550, 1507, 1468, 1418, 1354, 1308, 1280, 817, 747 cm^{-1}.

HRMS calcd *m/z* for $C_{41}H_{34}BN$ $[M]^+$: 551.2786; found (ESI): 551.2799

Compound **11b**

Compound **11b** was prepared following the general procedure from **7b** (10.5 mg, 25 µmol). NMR analysis of the crude mixture showed a regioselectivity of 42:58 (**2b**:**11b)**. The product was isolated as a white solid (5.2 mg, 12 µmol, 50%) after purification by HPLC (acetonitrile:H_2O 95:5→100:0 over 5 min, flow rate 1.0 mL/min at 295 K).

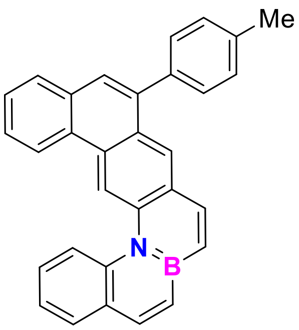

^{1}H NMR (400 MHz, $CDCl_3$) δ = 9.93 (s, 1H), 8.80 (d, J = 8.6 Hz, 1H), 8.63 – 8.58 (m, 1H), 8.30 (s, 1H), 8.09 (d, J = 11.3 Hz, 1H), 8.03 (d, J = 11.2 Hz, 1H), 7.93 – 7.89 (m, 1H), 7.86 (dd, J = 7.8, 1.6 Hz, 1H), 7.67 – 7.62 (m, 3H), 7.58 – 7.53 (m, 3H), 7.44 – 7.38 (m, 3H), 7.25 (d, J = 11.2 Hz, 1H), 7.21 (d, J = 11.2 Hz, 1H), 2.53 (s, 3H) ppm.

^{13}C NMR (101 MHz, $CDCl_3$) δ = 143.9 (1CH), 143.4 (1CH), 139.5 (1Cq), 138.6 (1Cq), 137.8 (1Cq), 137.5 (1Cq), 137.2 (1Cq), 132.4 (1Cq), 130.3 (1CH), 130.1 (2CH), 129.9 (1Cq), 129.8 (1Cq), 129.4 (2CH), 129.22 (1CH), 129.18 (1CH), 129.0 (1CH), 127.8 (1CH), 127.34 (1Cq), 127.31 (1CH), 127.0 (1CH), 126.8 (2CH), 123.0 (1CH), 122.8 (1CH), 120.6 (1CH), 114.8 (1CH), 21.5 ($1CH_3$) ppm. The carbon atoms bonded to boron were not observed.

^{11}B NMR (161 MHz, $CDCl_3$) δ = 31.36 ppm.

IR (ATR) $\tilde{\nu}$ = 3011, 2918, 2851, 1592, 1549, 1472, 1439, 1404, 1368, 1296, 1252, 1211, 1180, 905, 813, 785, 761, 746, 732 cm^{-1}.

HRMS: calcd *m/z* for $C_{31}H_{23}BN$ $[M+H]^+$: 420.1923; found (ESI) 420.1933

M.p. = 220 °C

Compound **S11c**

Compound **S11c** was prepared following the general procedure from **7c** (10.9 mg, 25 µmol). NMR analysis of the crude mixture showed a regioselectivity of 13:87 (**2c**: **S11c**). The product was isolated as a white solid (7.6 mg, 17 µmol, 70%) after purification by HPLC (acetonitrile:H_2O 90:10→100:0 over 20 min, flow rate 1.0 mL/min at 295 K).

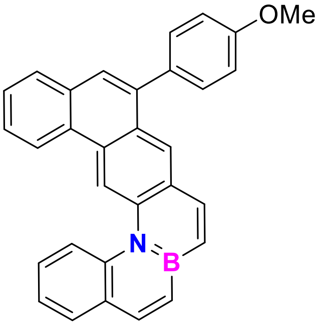

^{1}H NMR (400 MHz, $CDCl_3$) δ = 9.92 (s, 1H), 8.79 (d, J = 8.7 Hz, 1H), 8.63 – 8.58 (m, 1H), 8.29 (s, 1H), 8.09 (d, J = 11.2 Hz, 1H), 8.03 (d, J = 11.4 Hz, 1H), 7.93 – 7.89 (m, 1H), 7.86 (dd, J = 7.8, 1.6 Hz, 1H), 7.67 – 7.52 (m, 6H), 7.41 (ddd, J = 7.9, 7.1, 1.0 Hz, 1H), 7.25 (d, J = 11.2 Hz, 1H), 7.21 (d, J = 11.3 Hz, 1H), 7.15 – 7.11 (m, 2H), 3.96 (s, 3H) ppm.

^{13}C NMR (101 MHz, $CDCl_3$) δ = 159.4 (1Cq), 143.9 (1CH), 143.4 (1CH), 139.5 (1Cq), 138.3 (1Cq), 137.2 (1Cq), 133.1 (1Cq), 132.4 (1Cq), 131.3 (2CH), 130.3 (1CH), 129.82 (1Cq), 129.76 (1Cq), 129.23 (1Cq), 129.17 (1Cq), 128.9 (1CH), 127.8 (1CH), 127.5 (1Cq), 127.3 (1CH), 127.0 (1CH), 126.8 (2CH), 123.0 (1CH), 122.7 (1CH), 120.5 (1CH), 114.8 (1CH), 114.1 (2CH), 55.6 ($1CH_3$) ppm.

^{11}B NMR (128 MHz, $CDCl_3$) δ = 31.90 ppm.

IR (ATR) $\tilde{\nu}$ = 3008, 2832, 1592, 1509, 1285, 1247, 1029, 831, 809, 756 cm^{-1}.

HRMS: calcd *m/z* for $C_{31}H_{23}BNO$ $[M+H]^+$: 436.1867; found (ESI) 436.1871

Compound **S11e**

Compound **S11e** was prepared following the general procedure from **7e** (10.6 mg, 25 µmol). NMR analysis of the crude mixture showed a regioselectivity of 57:43 (**2e**: **S11e**). The product was isolated as a white solid (3.6 mg, 9 µmol, 35%) after purification by HPLC (acetonitrile:H_2O 90:10→100:0 over 10 min, flow rate 1.0 mL/min at 295 K).

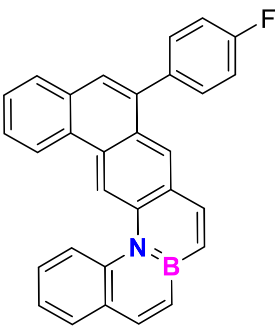

^{1}H NMR (400 MHz, $CDCl_3$) δ = 9.93 (s, 1H), 8.78 (d, J = 8.6 Hz, 1H), 8.63 – 8.59 (m, 1H), 8.19 (s, 1H), 8.10 (d, J = 11.1 Hz, 1H), 8.02 (d, J = 11.2 Hz, 1H), 7.93 – 7.90 (m, 1H), 7.86 (dd, J = 7.8, 1.6 Hz, 1H), 7.69 – 7.59 (m, 5H), 7.56 (ddd, J = 8.6, 7.0, 1.7 Hz, 1H), 7.42 (ddd, J = 8.0, 7.2, 1.0 Hz, 1H), 7.30 – 7.21 (m, 4H) ppm.

^{13}C NMR (101 MHz, $CDCl_3$) δ = 162.6 (d, $^1J_{C\text{-}F}$ = 246.5 Hz, 1Cq), 144.0 (1CH), 143.3 (1CH), 139.5 (1Cq), 137.6 (1Cq), 137.3 (1Cq), 136.7 (d, $^4J_{Cq\text{-}F}$ = 3.4 Hz, 1Cq), 132.2 (1Cq), 131.8 (d, $^3J_{C\text{-}F}$ = 8.0 Hz, 2CH), 130.3 (1CH), 130.0 (1Cq), 129.8 (1Cq), 129.25 (1Cq), 129.19 (1Cq), 129.0 (1CH), 127.5 (1CH), 127.4 (1CH), 127.2 (1Cq), 127.08 (1CH), 127.07 (1CH), 127.05 (1CH), 123.1 (1CH), 122.8 (1CH), 120.5 (1CH), 115.6 (d, $^2J_{C\text{-}F}$ = 21.3 Hz, 2CH), 114.9 (1CH) ppm. The carbon atoms bonded to boron were not observed.

^{11}B NMR (161 MHz, $CDCl_3$) δ = 31.66 ppm.

^{19}F NMR (282 MHz, $CDCl_3$) δ = -114.83 ppm.

IR (ATR) $\tilde{\nu}$ = 3730, 3701, 3626, 3599, 3054, 3019, 2921, 2853, 1593, 1549, 1508, 1222, 901, 835, 817, 759, 747 cm^{-1}.

HRMS: calcd *m/z* for $C_{30}H_{20}BFN$ $[M+H]^+$: 424.1673; found (ESI) 424.1656

2.5. Post-modification reactions. Synthesis of **1b** and **13b**

<u>*Bromination of helicene* **1b**</u>

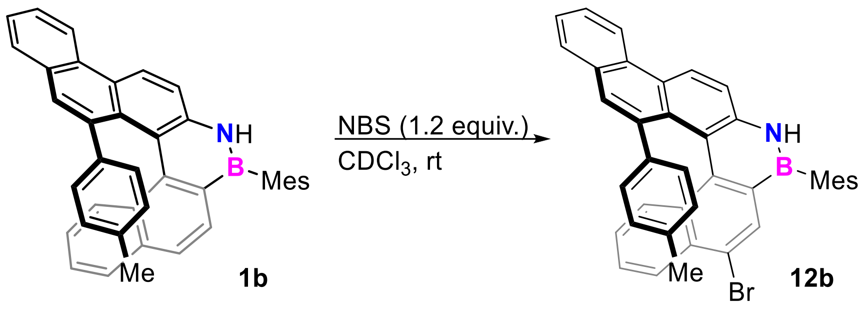

Scheme S6** Selective bromination of azabora[5]helicene towards compound **12b

To a mixture of *rac*-**1b** (10.0 mg, 18 µmol, 1.0 equiv.) in $CHCl_3$ (2 mL), *N*-bromosuccinimide (3.95 mg, 22 µmol, 1.2 equiv.) was added in one portion, and the resulting suspension was stirred at room temperature for 24 hours. Then, the reaction mixture was washed with H_2O (3 × 2 mL) and the organic phase dried over Na_2SO_4, filtered and concentrated under reduced pressure to afford the desired product *rac*-**12b** in 87% yield (10 mg, 16 µmol) as a white solid. Starting from (+)-**1b** (27 mg, 50 µmol), the product (+)-**12b** was obtained in 90% yield (28 mg, 45 µmol) without erosion of the enantiopurity (96% ee).

^{1}H NMR (500 MHz, CD_2Cl_2, –35 °C) δ = 8.88 (d, *J* = 9.1 Hz, 1H), 8.78 (d, *J* = 8.1 Hz, 1H), 8.00 (d, *J* = 8.2 Hz, 1H), 7.98 (br. s, 1H), 7.94 (dd, *J* = 8.0, 1.3 Hz, 1H), 7.75 (ddd, *J* = 8.3, 7.0, 1.4 Hz, 1H), 7.71 (s, 1H), 7.68 – 7.63 (m, 2H), 7.54 (s, 1H), 7.51 – 7.46 (m, 2H), 7.15 (ddd, *J* = 8.3, 6.9, 1.3 Hz, 1H), 7.04 (s, 1H), 6.99 (s, 1H), 6.54 (d, *J* = 8.1 Hz, 1H), 6.36 (dd, *J* = 7.8, 2.1 Hz, 1H), 6.18 (dd, *J* = 7.9, 1.4 Hz, 1H), 5.84 (dd, *J* = 7.9, 2.0 Hz, 1H), 2.41 (s, 3H), 2.33 (s, 3H), 2.14 (s, 3H), 2.07 (s, 3H) ppm.

^{1}H NMR (300 MHz, $CDCl_3$) δ = 8.88 (d, *J* = 9.0 Hz, 1H), 8.76 (d, *J* = 8.1 Hz, 1H), 8.03 (d, *J* = 7.9 Hz, 1H), 7.97 – 7.89 (m, 2H), 7.77 – 7.59 (m, 4H), 7.56 – 7.43 (m, 3H), 7.14 (ddd, *J* = 8.3, 7.0, 1.2 Hz, 1H), 7.02 (s, 1H), 6.97 (s, 1H), 6.23 (br. m, 4H), 2.41 (s, 3H), 2.33 (s, 3H), 2.14 (s, 3H), 2.08 (s, 3H) ppm.

^{13}C NMR (126 MHz, CD_2Cl_2, –35 °C) δ = 140.7 (1Cq), 140.5 (1Cq), 139.6 (1Cq), 138.9 (1Cq), 138.5 (1Cq), 138.3 (1Cq), 138.1 (1Cq), 135.2 (br, 1Cq-B), 135.0 (1Cq), 132.1 (1CH), 131.8 (1Cq), 131.6 (1Cq), 131.5 (br, 1Cq-B), 131.1 (1Cq), 130.1 (1Cq), 129.83 (1CH), 129.82 (1Cq), 128.6 (1CH), 127.6 (1CH), 127.34 (1CH), 127.28 (1CH), 127.22 (1CH), 127.20 (1CH), 126.9 (1CH), 126.82 (1CH), 126.79 (1CH), 126.74 (1CH), 126.71 (1Cq), 126.2 (1CH), 125.9 (1CH),

125.7 (1CH), 123.5 (1CH), 122.6 (1CH), 121.1 (1Cq), 118.5 (1CH), 116.6 (1Cq), 23.1 ($1CH_3$), 23.0 ($1CH_3$), 21.5 ($1CH_3$), 21.1 ($1CH_3$) ppm.

^{11}B NMR (161 MHz, $CDCl_3$) δ = 39.71 ppm.

IR (ATR) $\tilde{\nu}$ = 3367, 2909, 2850, 1568, 1546, 1496, 1448, 1410, 1315, 1237, 1045, 904, 814, 724 cm^{-1}.

HRMS: calcd *m/z* for $C_{40}H_{31}BBrN$ $[M]^+$: 615.1734; found (ESI) 615.1747

M. p. = 173 °C

Ring opening of **1b** to **13b**

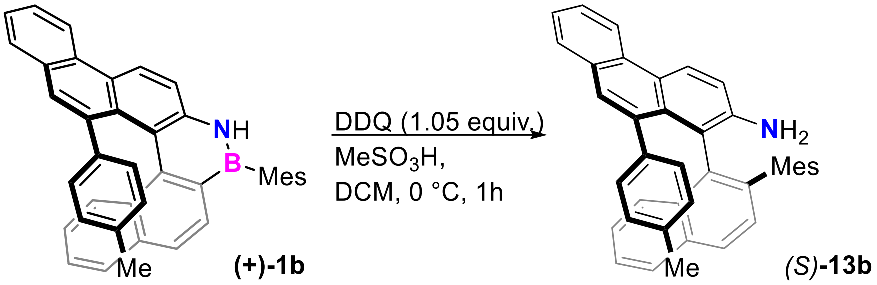

Scheme S7** Synthesis of axially chiral anline (R)-**13b

To a solution of helicene (+)-**1b** (10 mg, 18.6 µmol) in dry CH_2Cl_2 (2 mL), under argon at 0 °C, $MeSO_3H$ (0.1 mL) and DDQ (4.5 mg, 19.8 µmol, 1.05 equiv.) were added. The resulting mixture was stirred for 1 hour at the same temperature. Then, it was quenched with an aqueous solution of $NaHCO_3$, and extracted with CH_2Cl_2 (3× 2 mL). The combined organic phases were washed with H_2O and brine, dried over Na_2SO_4, and concentrated under reduced pressure. The crude product was purified by Silica gel column chromatography (hexane/toluene 3:1) yielding the product (*S*)-**13b** (7 mg, 13.0 µmol, 71%) as a white solid without erosion of the enantiopurity (96% ee).

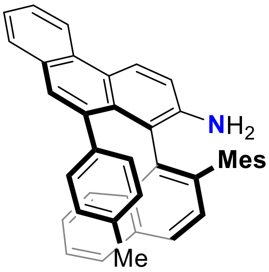

^{1}H NMR (500 MHz, $CDCl_3$, –35 °C) δ = 8.51 (d, *J* = 8.4 Hz, 1H), 8.46 (d, *J* = 9.2 Hz, 1H), 7.69 – 7.65 (m, 2H), 7.60 – 7.56 (m, 3H), 7.47 (ddd, *J* = 7.8, 7.1, 0.9 Hz, 1H), 7.35 (ddd, *J* = 8.0, 6.7, 1.2 Hz, 1H), 7.30 – 7.26 (m, 1H), 7.21 (s, 1H), 7.15 (d, *J* = 8.4 Hz, 1H), 6.97 (d, *J* = 8.8 Hz, 1H), 6.83 (d, *J* = 7.4 Hz, 1H), 6.58 (s, 1H), 6.48 (d, *J* = 7.6 Hz, 1H), 6.36 (d, *J* = 7.4 Hz, 1H), 6.28 (s, 1H), 5.99 (d, *J* = 7.5 Hz, 1H), 3.56 (s, 2H), 2.00 (s, 3H), 1.99 (s, 3H), 1.86 (s, 3H), 1.58 (s, 3H) ppm.

^{1}H NMR (400 MHz, $CDCl_3$) δ = 8.47 (d, *J* = 8.1 Hz, 1H), 8.42 (d, *J* = 9.1 Hz, 1H), 7.71 (d, *J* = 8.1 Hz, 1H), 7.66 (dd, *J* = 8.0, 1.2 Hz, 1H), 7.61 – 7.52 (m, 3H), 7.44 (ddd, *J* = 7.8, 7.1, 1.1 Hz, 1H), 7.34 (ddd, *J* = 8.2, 6.8, 1.4 Hz, 1H), 7.31 – 7.26 (m, 1H), 7.20 (s, 1H), 7.16 (d, *J* = 8.4 Hz, 1H), 6.94 (d, *J* = 8.9 Hz, 1H), 6.64 (br. s, 2H), 6.51 (s, 1H), 6.41 – 6.07 (m, 3H), 3.55 (s, 2H), 1.98 (s, 3H), 1.96 (s, 3H), 1.92 (s, 3H), 1.58 (s, 3H) ppm.

^{13}C NMR (126 MHz, $CDCl_3$, –35 °C) δ = 143.8 (1Cq), 139.7 (1Cq), 138.9 (1Cq), 138.1 (1Cq), 136.9 (1Cq), 136.5 (1Cq), 135.7 (1Cq), 135.6 (1Cq), 135.4 (1Cq), 133.9 (1Cq), 133.5 (1Cq),

133.1 (1Cq), 131.7 (1Cq), 130.9 (1CH), 130.1 (1Cq), 130.0 (1CH), 129.3 (1CH), 129.2 (1Cq), 128.0 (1CH), 127.9 (1CH), 127.52 (1CH), 127.48 (1CH), 127.4 (1CH), 127.3 (1CH), 126.5 (1CH), 126.4 (1CH), 126.2 (1CH), 125.4 (1CH), 125.2 (1CH), 125.1 (1CH), 125.0 (1CH), 124.1 (1Cq), 123.7 (1CH), 121.8 (1CH), 118.0 (1Cq), 116.3 (1CH), 22.4 ($1CH_3$), 21.1 ($1CH_3$), 21.0 ($1CH_3$), 20.8 ($1CH_3$) ppm.

IR (ATR) $\tilde{\nu}$ = 3385, 2920, 1609, 1456, 1377, 1276, 1260, 906, 811, 748 cm^{-1}.

HRMS: calcd *m/z* for $C_{40}H_{33}NNa$ $[M+Na]^+$: 550.2505; found (ESI) 550.2505

M. p. = 131 °C

3. NMR-Spectra

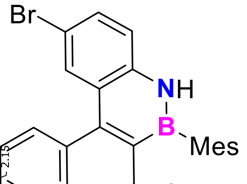

3

^{1}H NMR (300 MHz, $CDCl_3$) 3

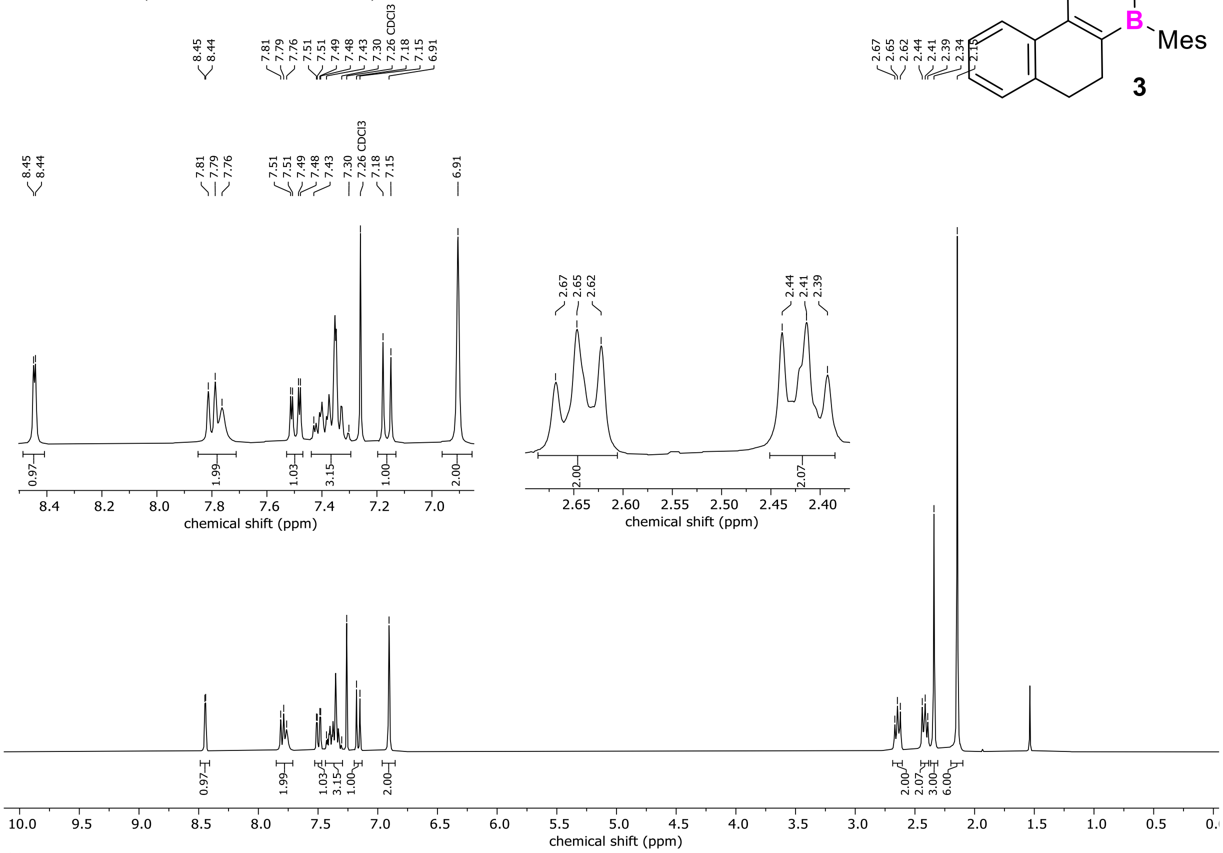

^{13}C NMR (101 MHz, $CDCl_3$)

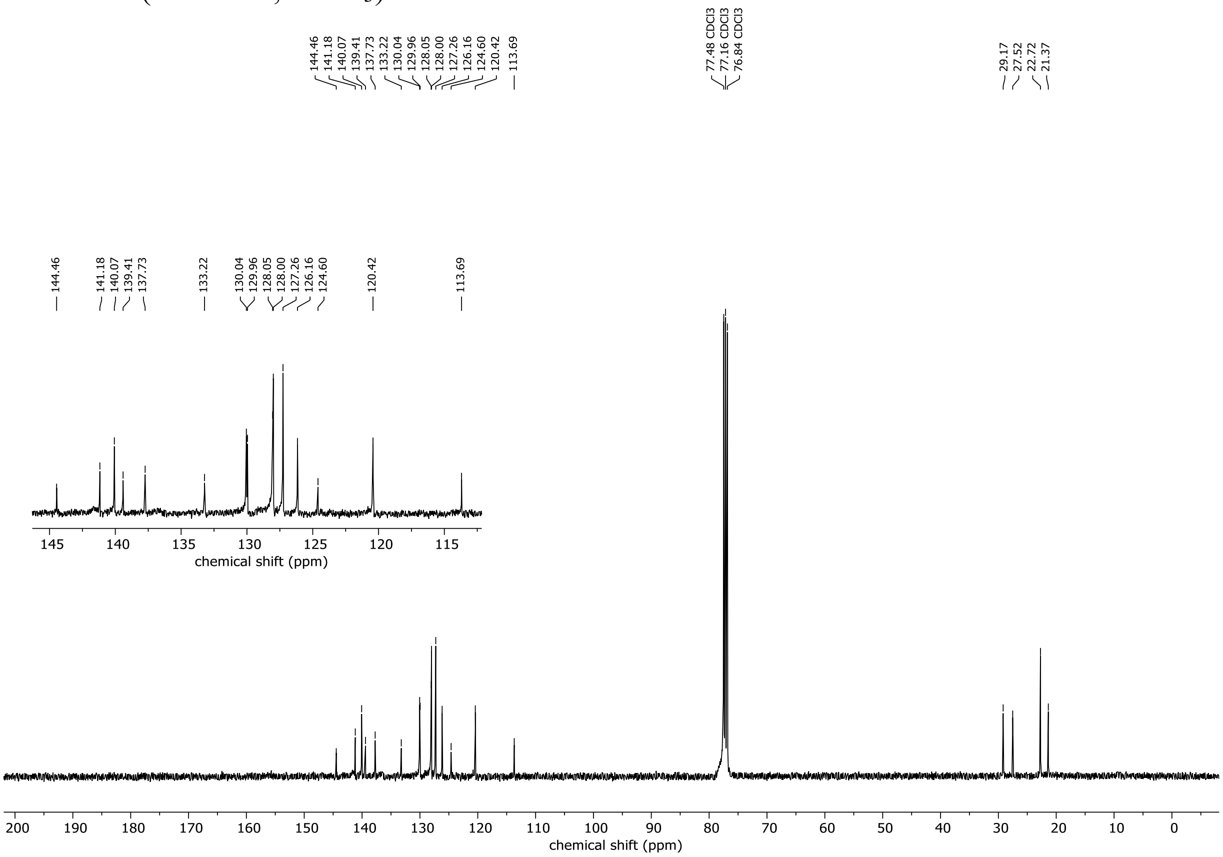

^{11}B NMR (161 MHz, $CDCl_3$)

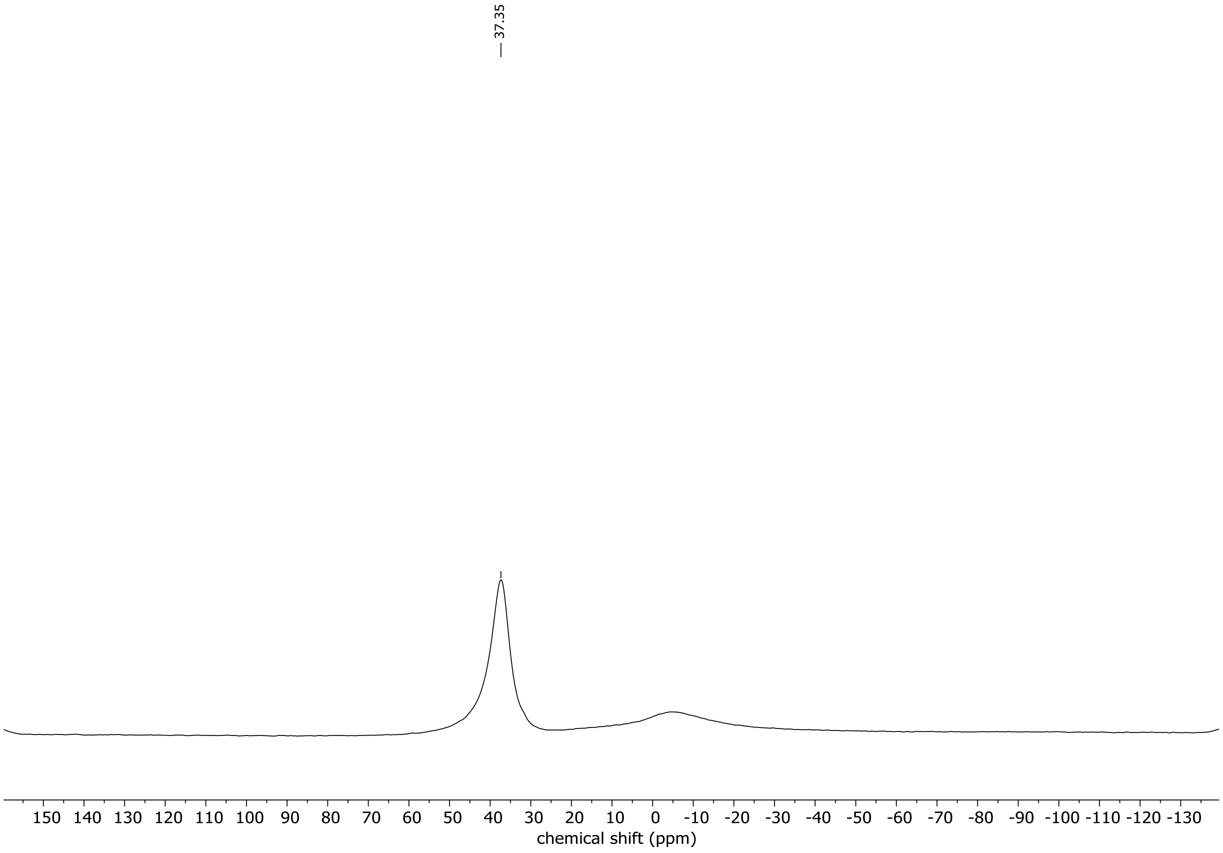

^{1}H NMR (300 MHz, $CDCl_3$) **S3(BPh)**

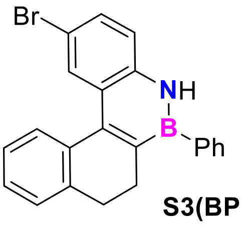

S3(BPh)

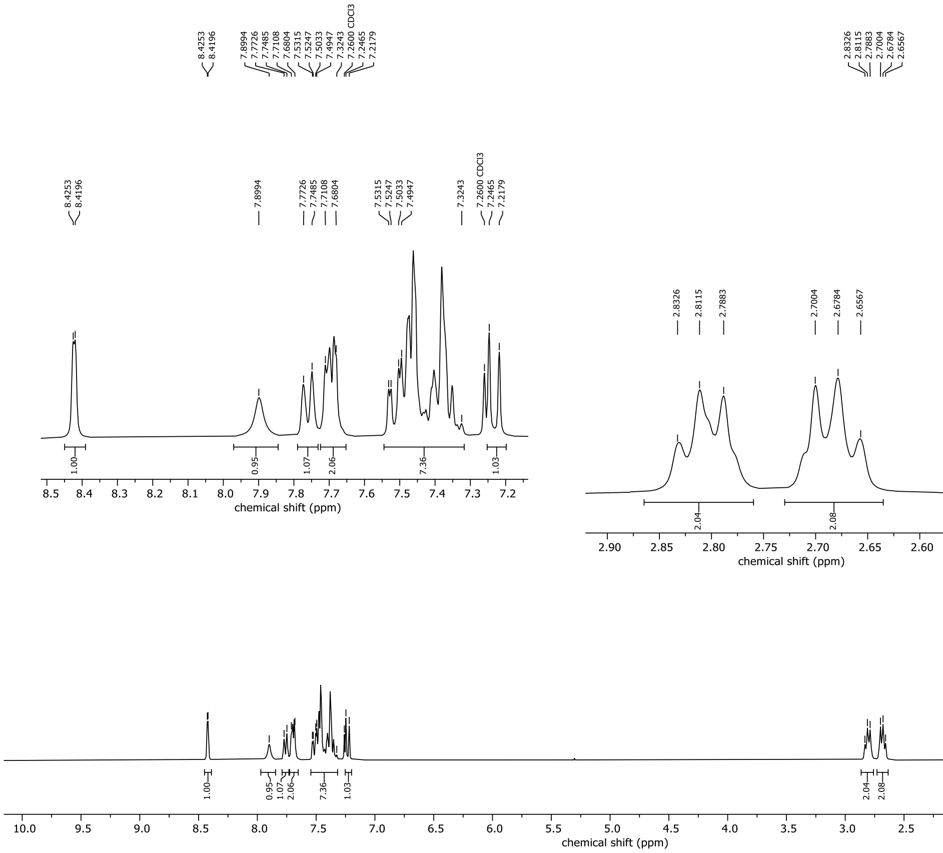

^{13}C NMR (101 MHz, $CDCl_3$)

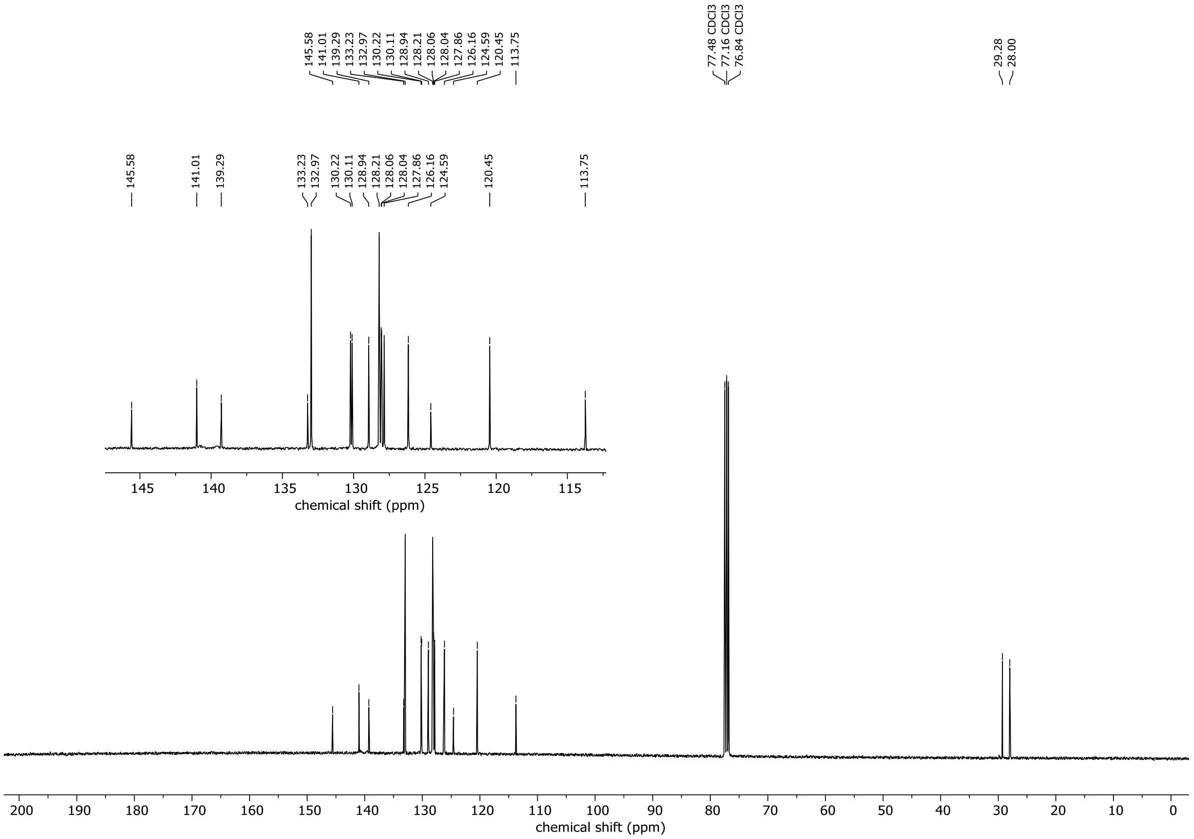

^{11}B NMR (128 MHz, $CDCl_3$)

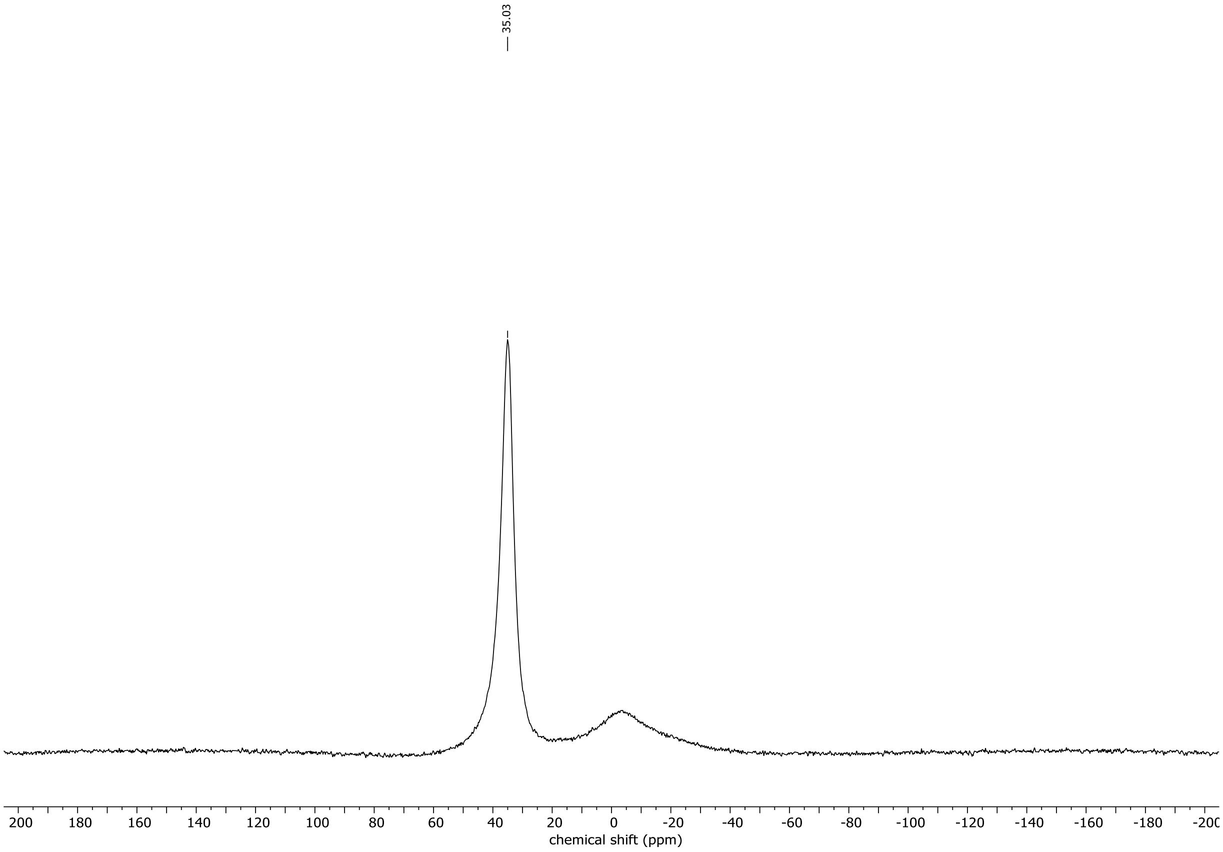

^{1}H NMR (300 MHz, CD_2Cl_2) **4**

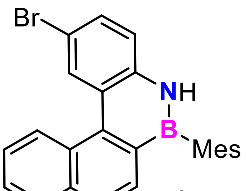

4

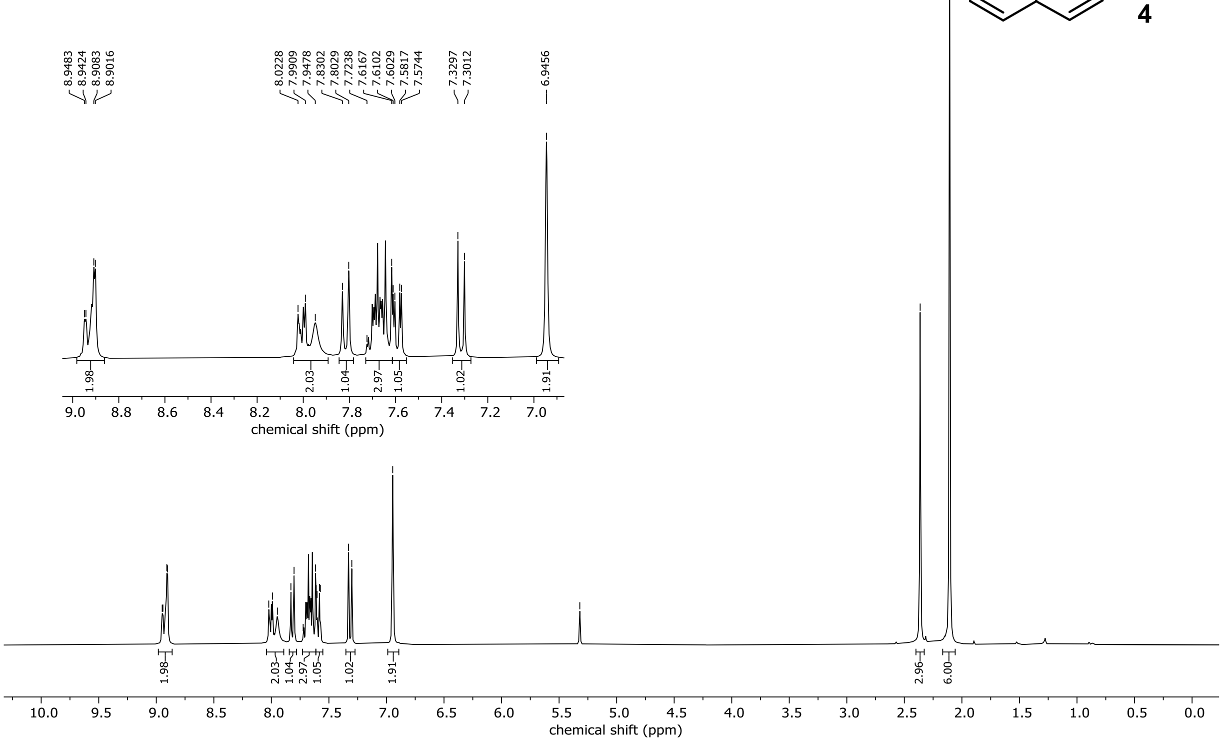

^{13}C NMR (101 MHz, $CDCl_3$)

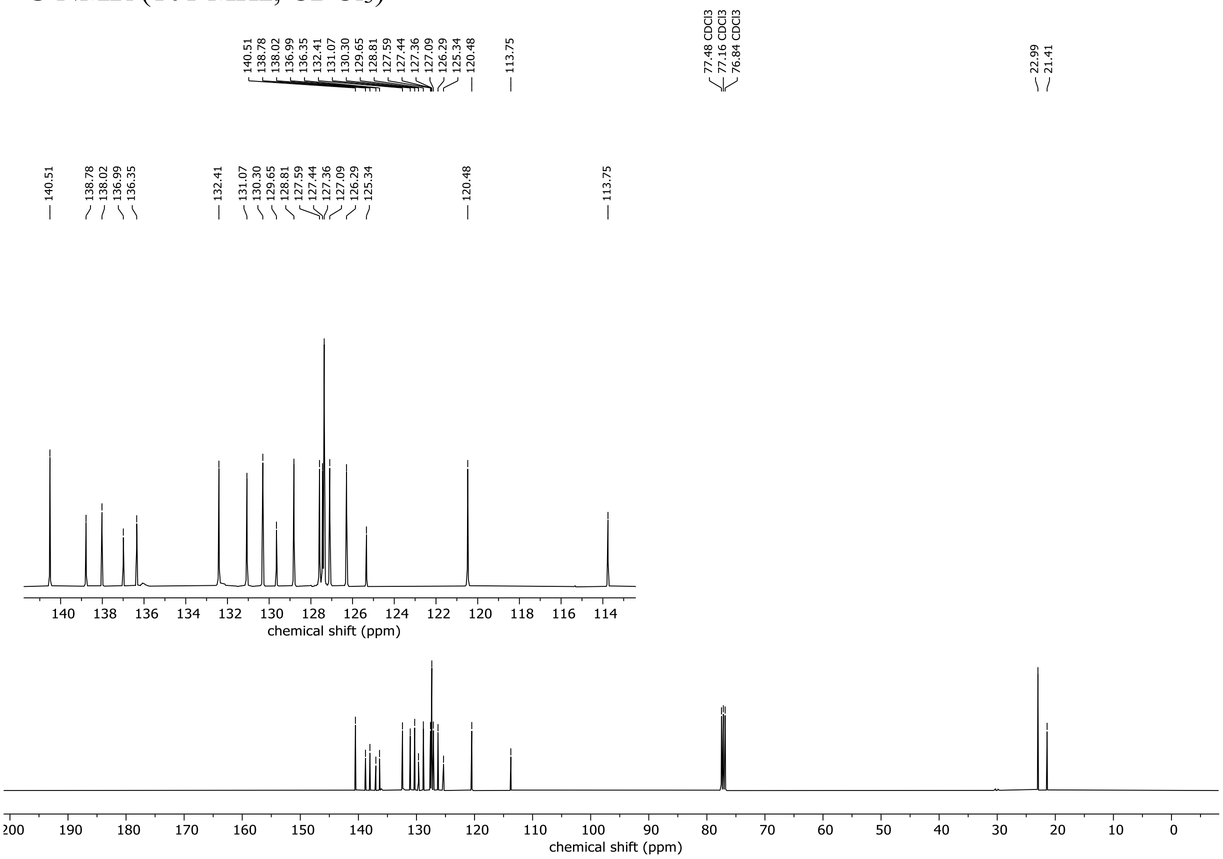

^{11}B NMR (161 MHz, CD_2Cl_2)

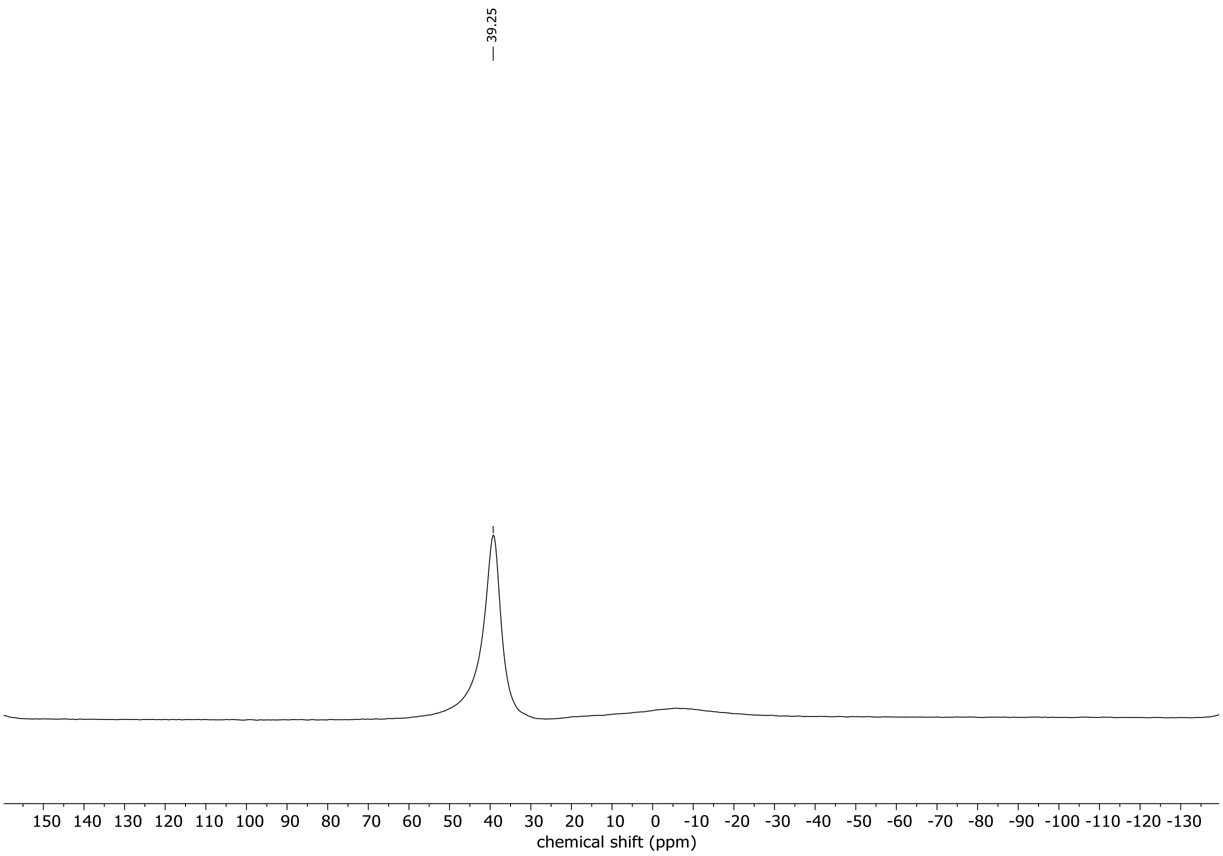

^{1}H NMR (400 MHz, $CDCl_3$) S4(BPh)

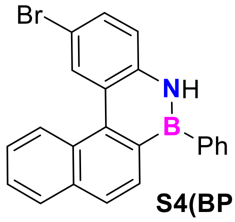

S4(BPh)

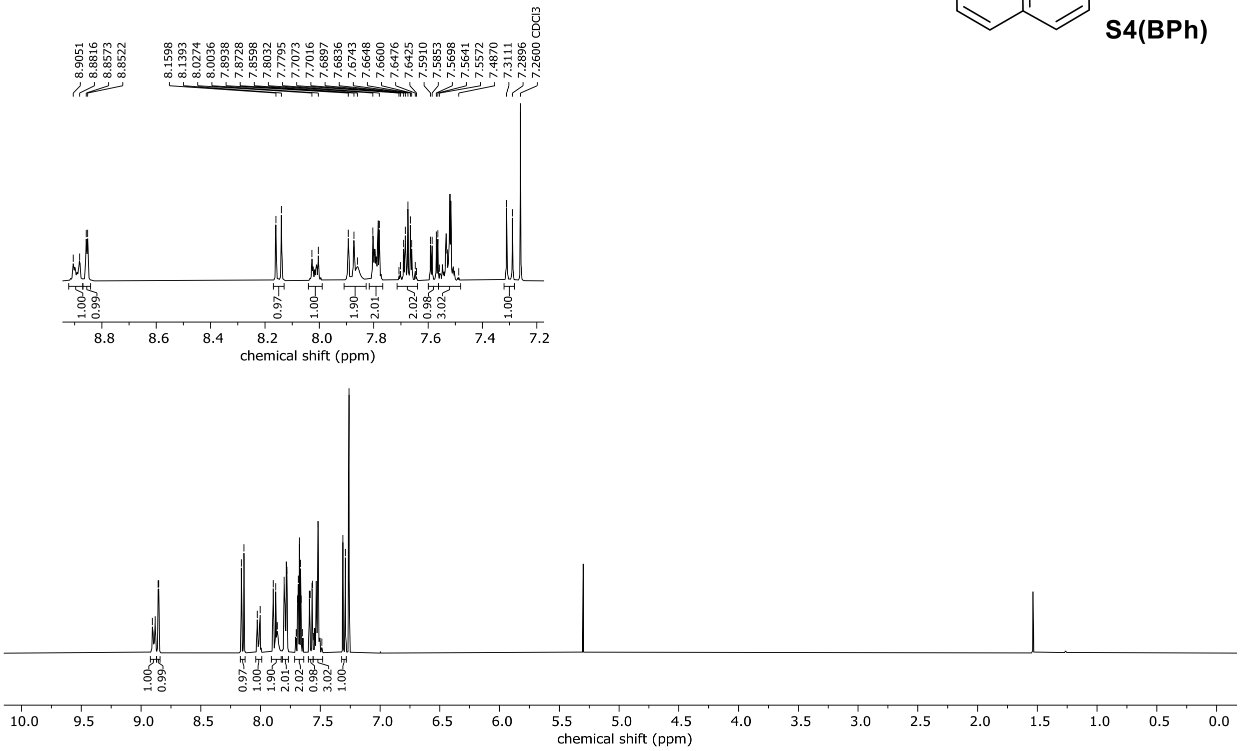

^{13}C NMR (101 MHz, $CDCl_3$)

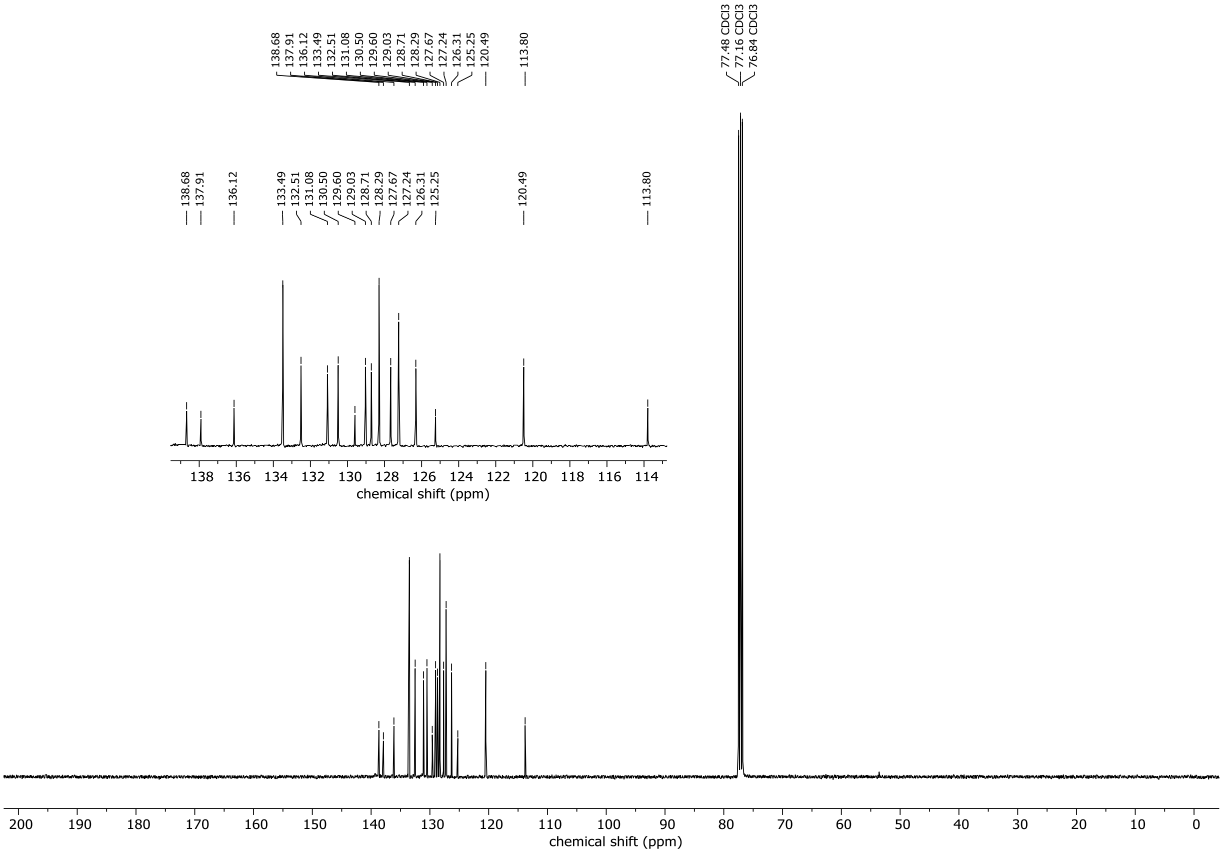

^{11}B NMR (128 MHz, $CDCl_3$)

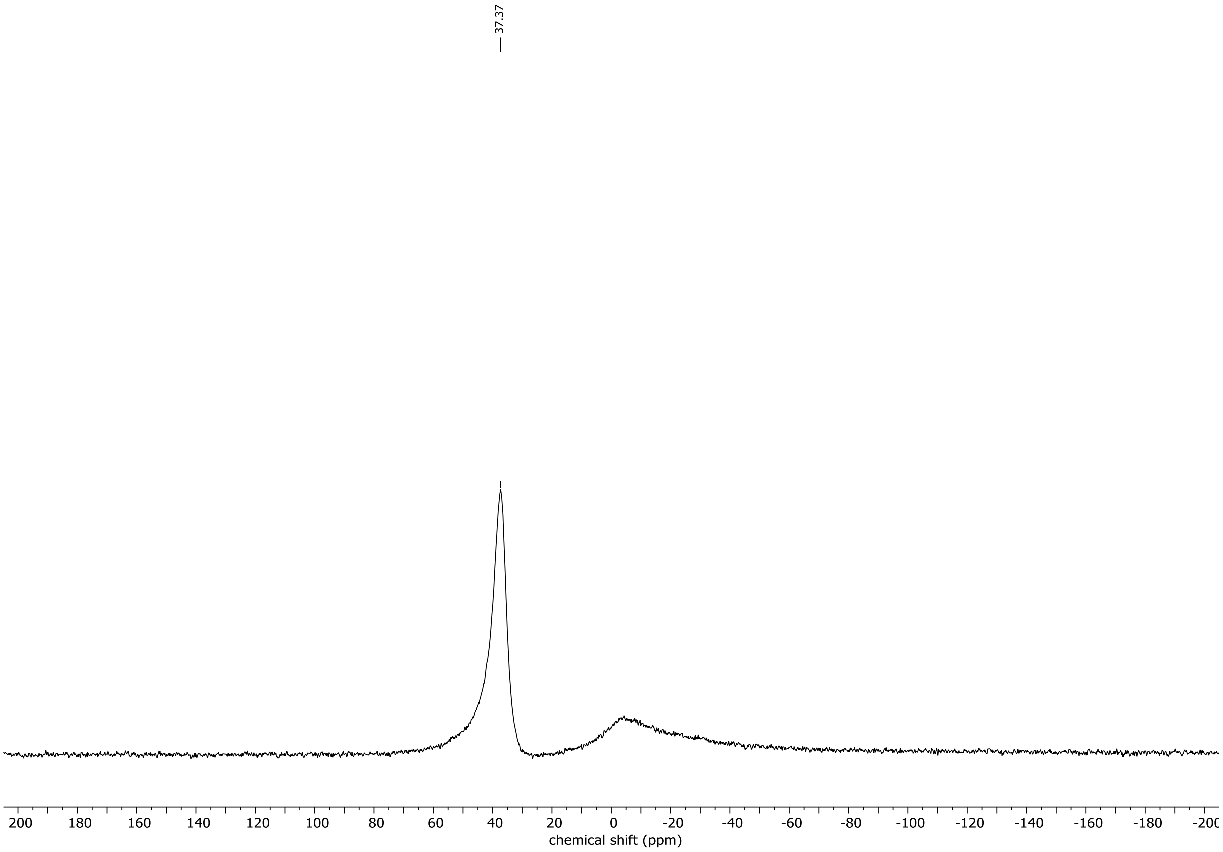

^{1}H NMR (300 MHz, $CDCl_3$) **S4(NMe)**

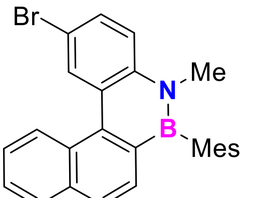

S4(NMe)

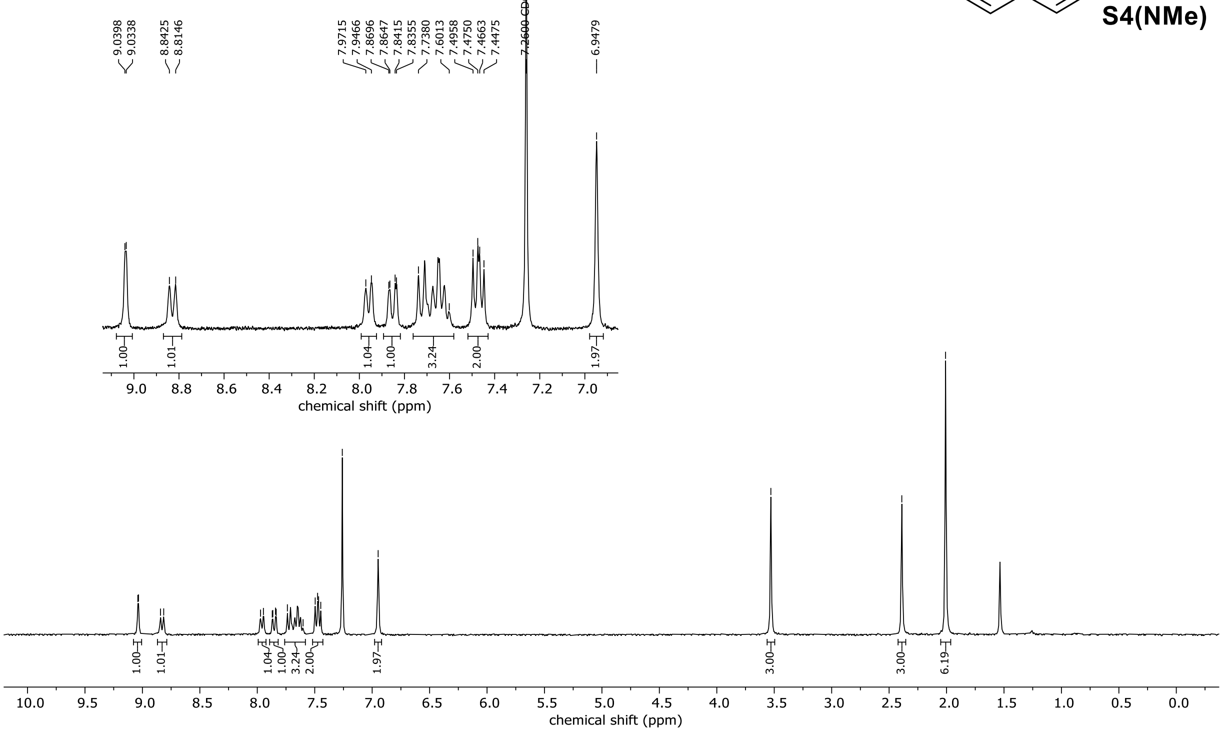

^{13}C NMR (101 MHz, $CDCl_3$)

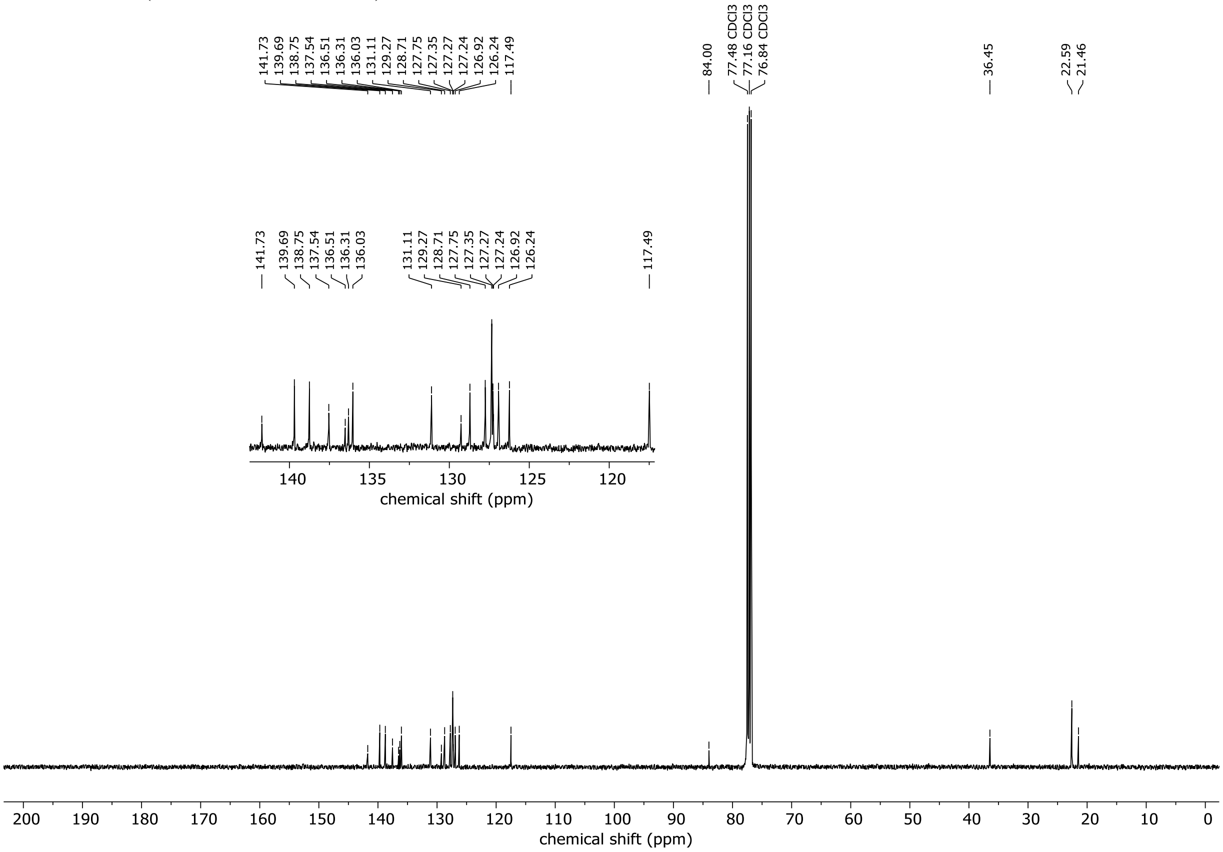

^{11}**B NMR** (161 MHz, $CDCl_3$)

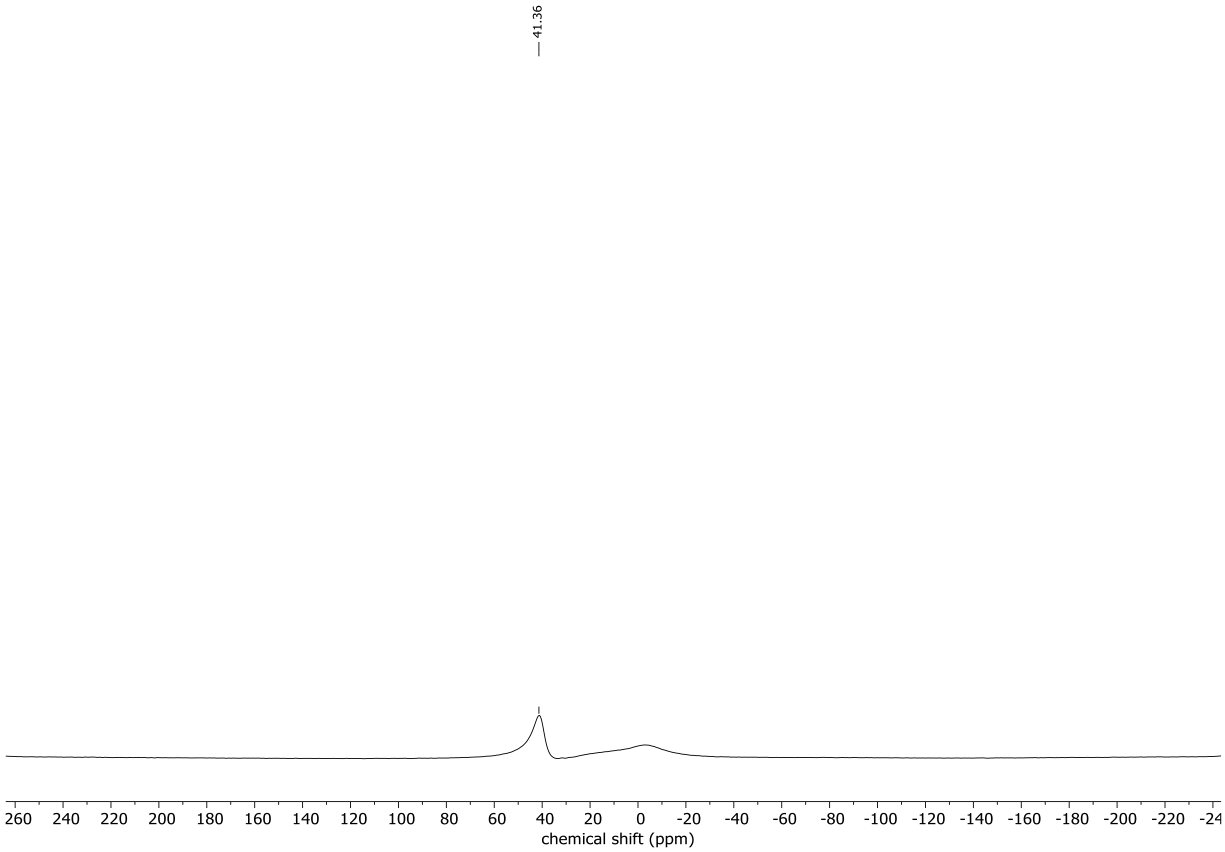

^{1}H-NMR (400 MHz, $CDCl_3$) **S9**

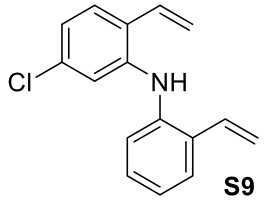

S9

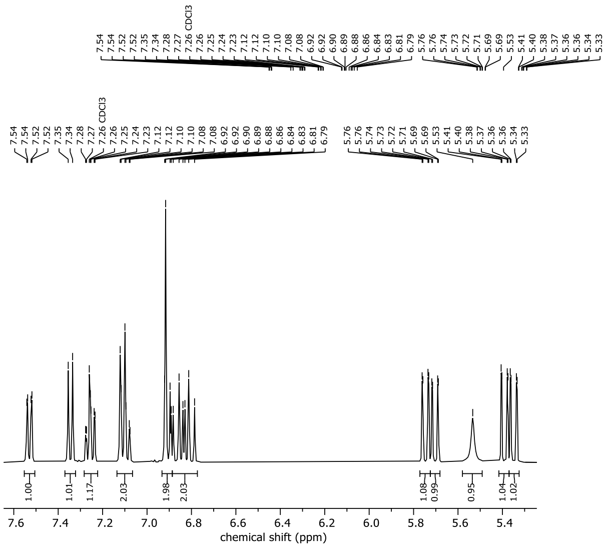

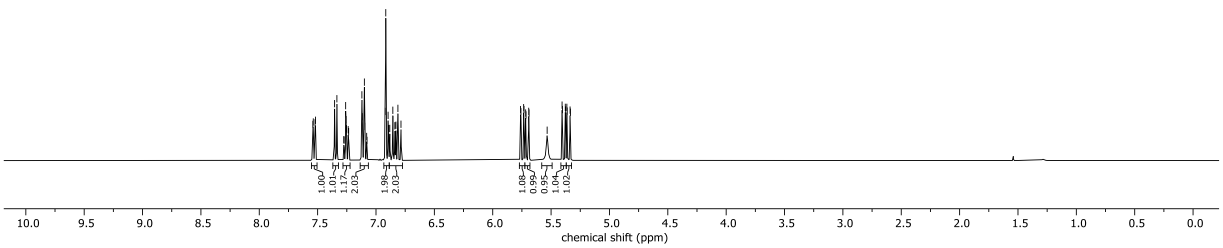

^{13}C-NMR (101 MHz, $CDCl_3$)

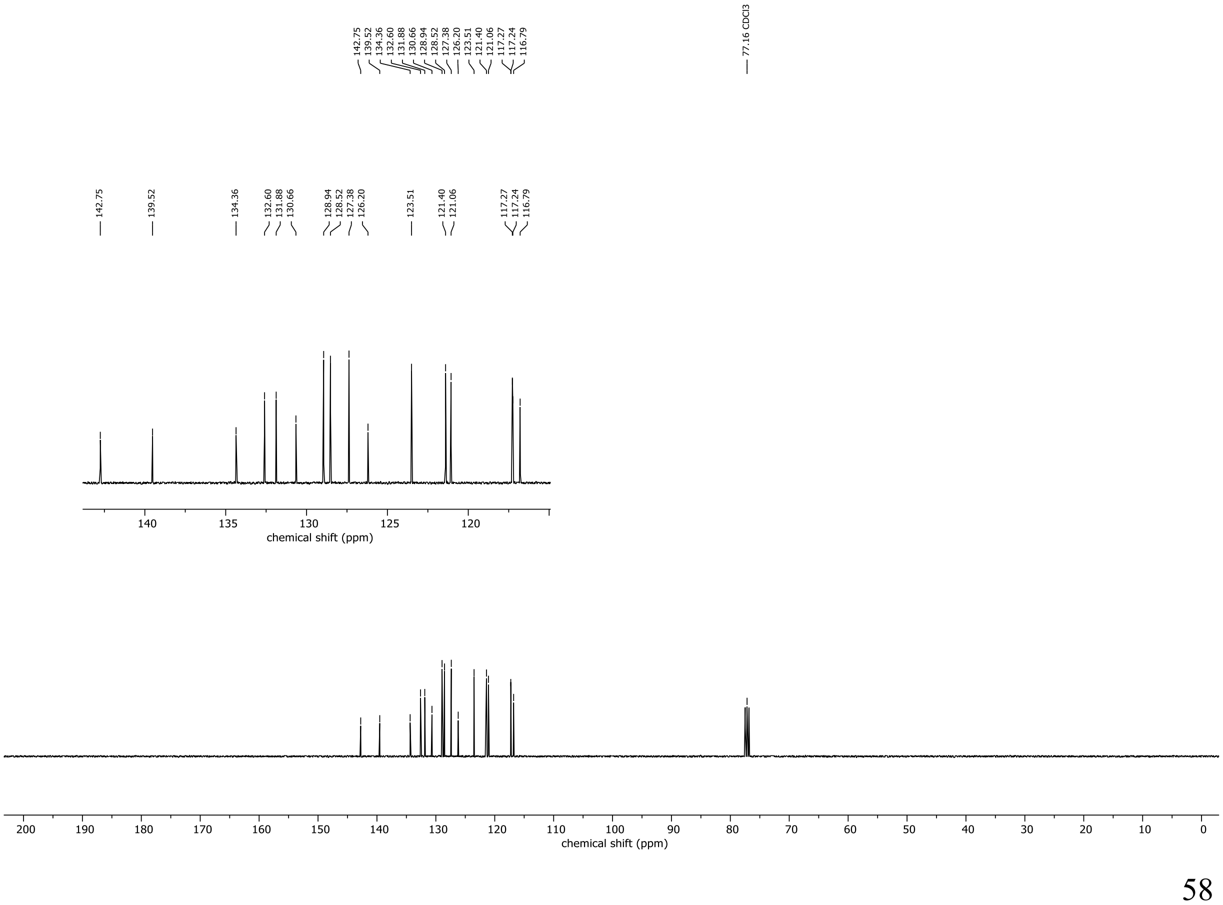

^{1}H-NMR (400 MHz, $CDCl_3$) **S12** and **S13**

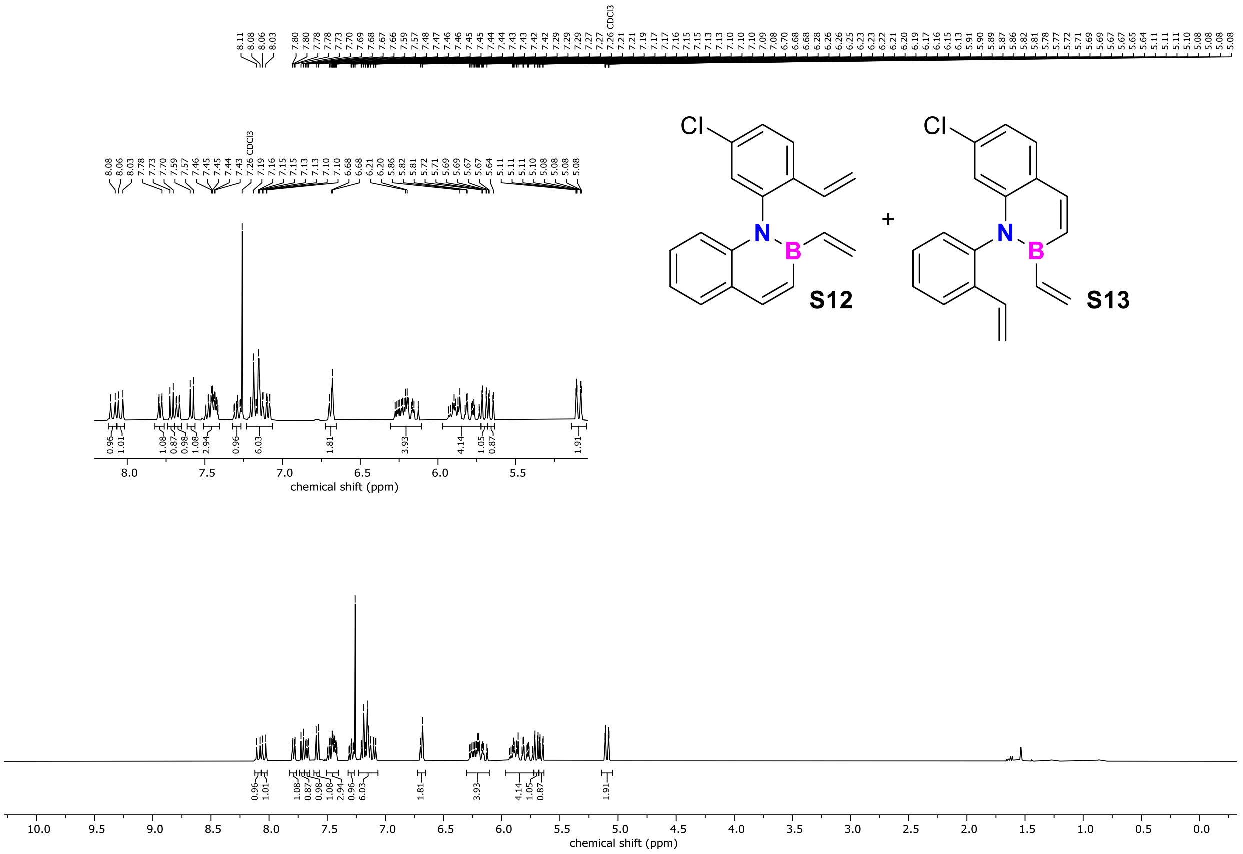

^{13}C-NMR (101 MHz, $CDCl_3$)

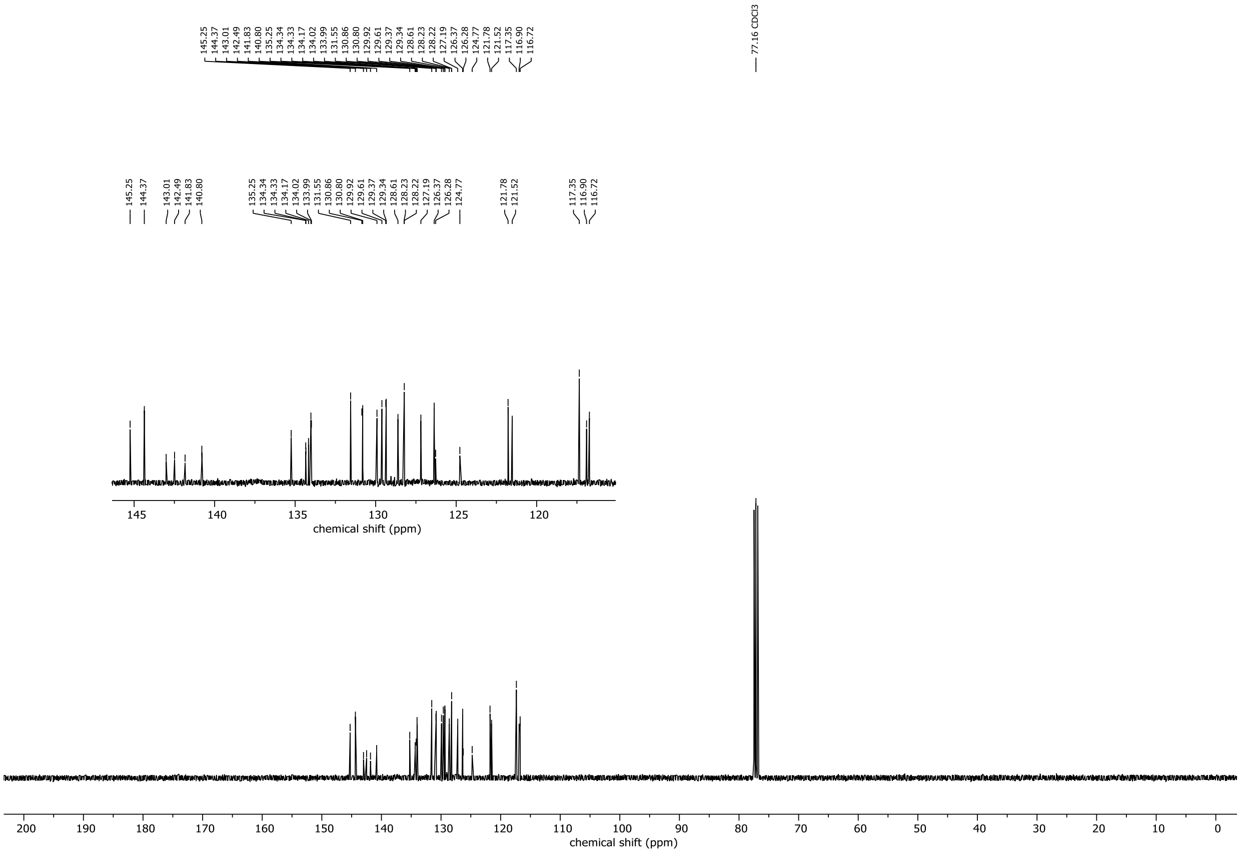

^{11}B-NMR (128 MHz, $CDCl_3$)

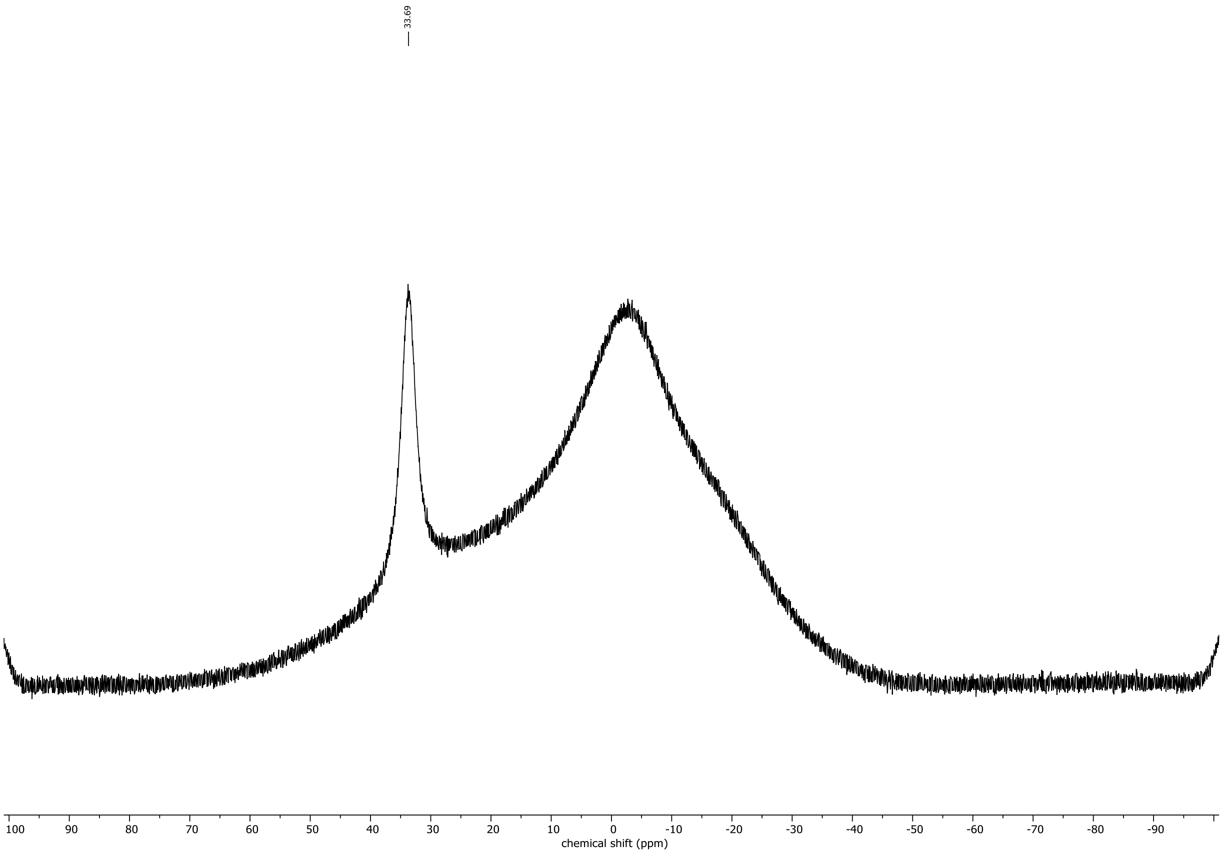

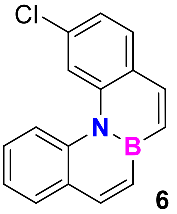

6

^{1}H-NMR (400 MHz, $CDCl_3$) **6**

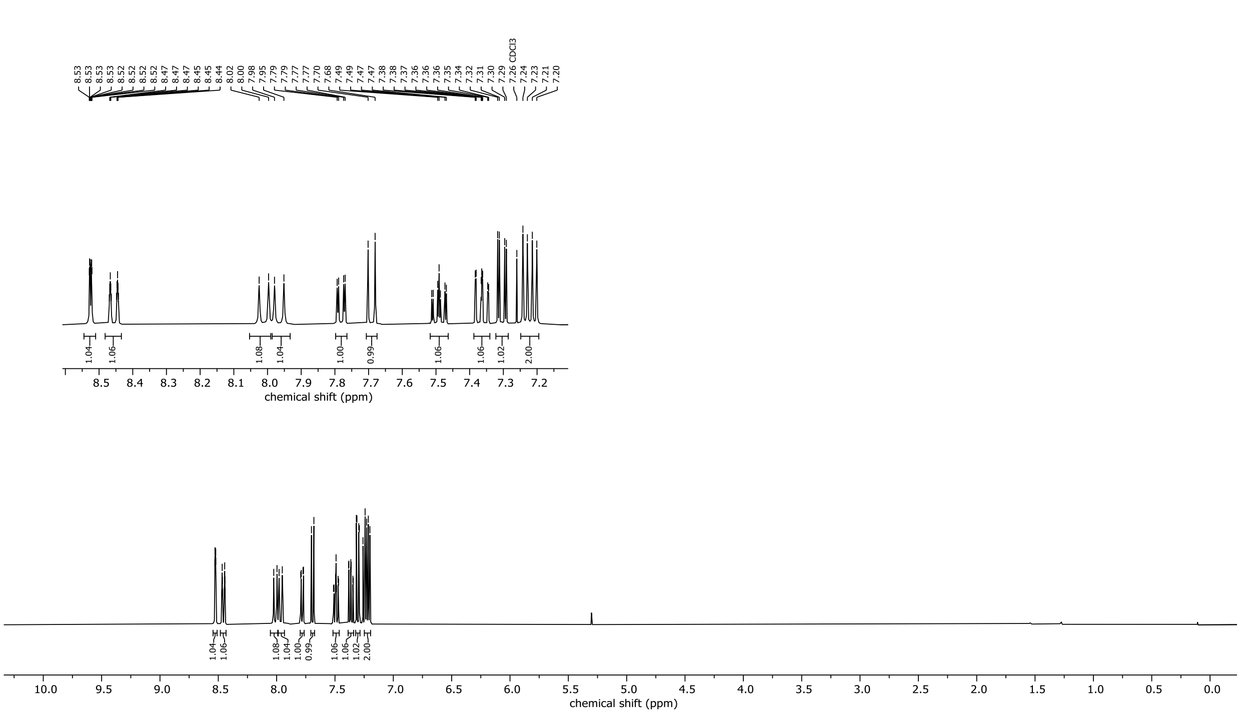

^{13}C-NMR (101 MHz, $CDCl_3$)

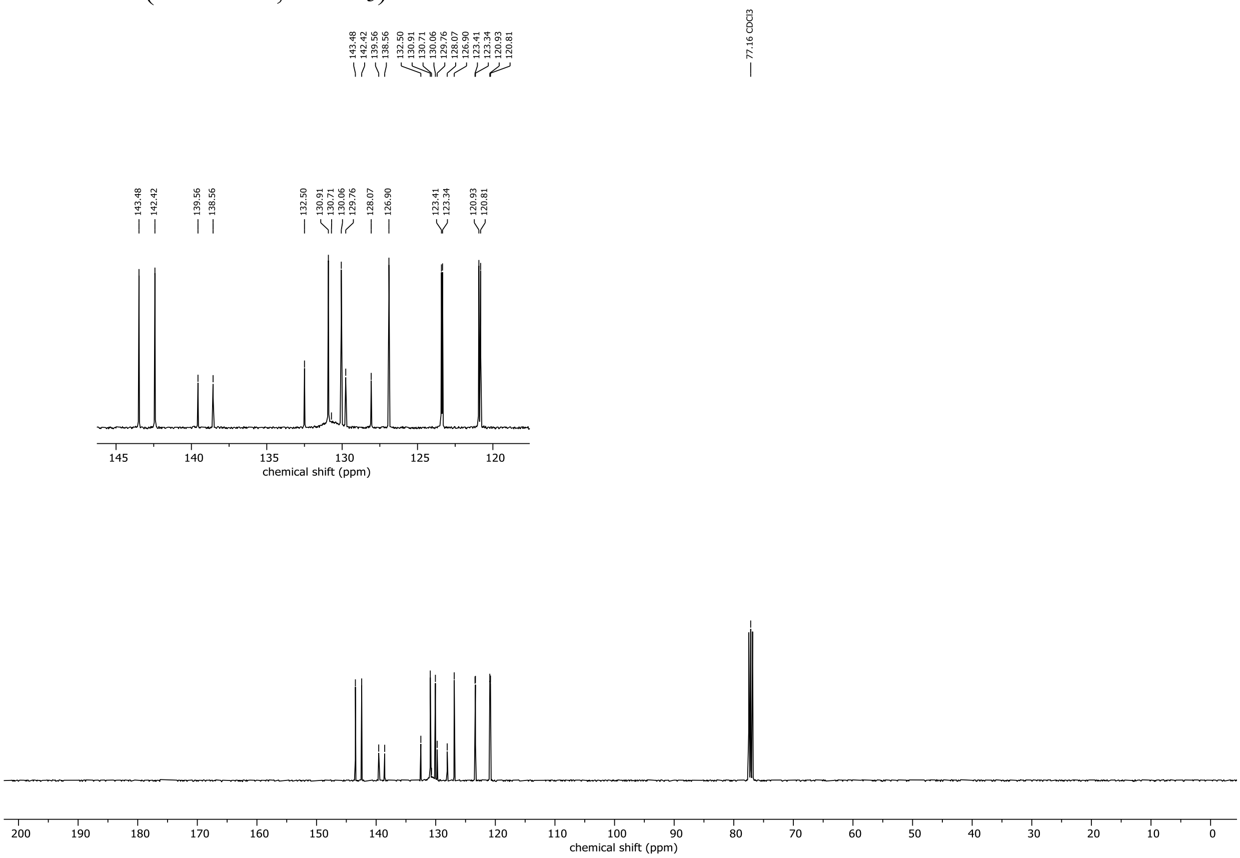

^{11}B-NMR (128 MHz, $CDCl_3$)

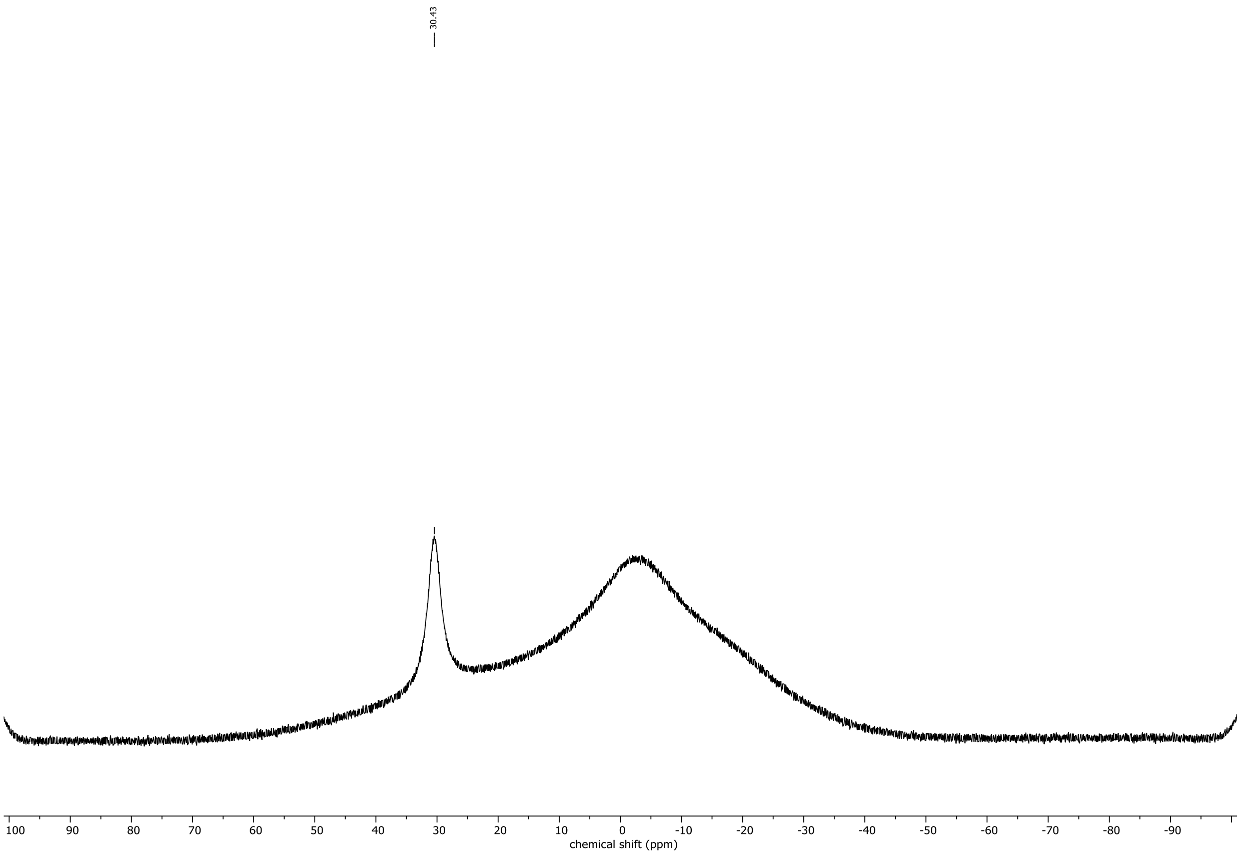

^{1}H NMR (400 MHz, CD_2Cl_2) **5a**

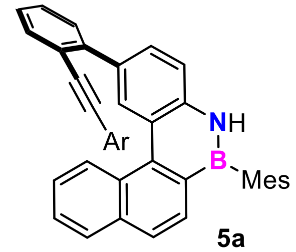

5a

Ar= C_6H_5

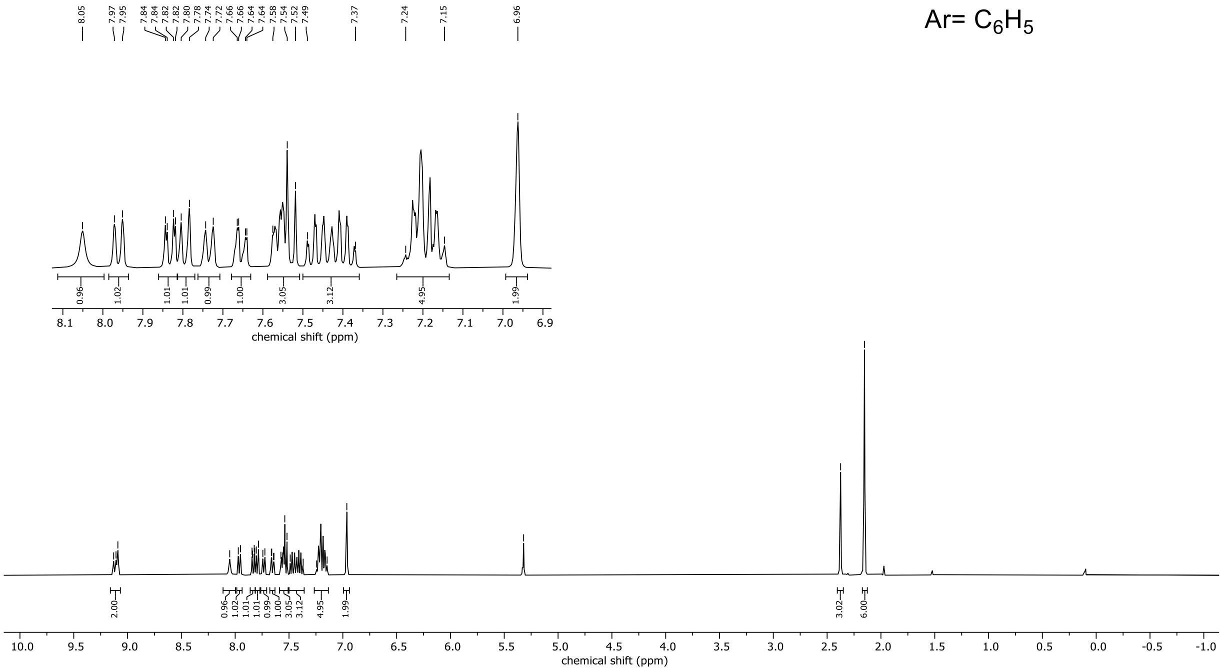

^{13}C NMR (101 MHz, CD_2Cl_2)

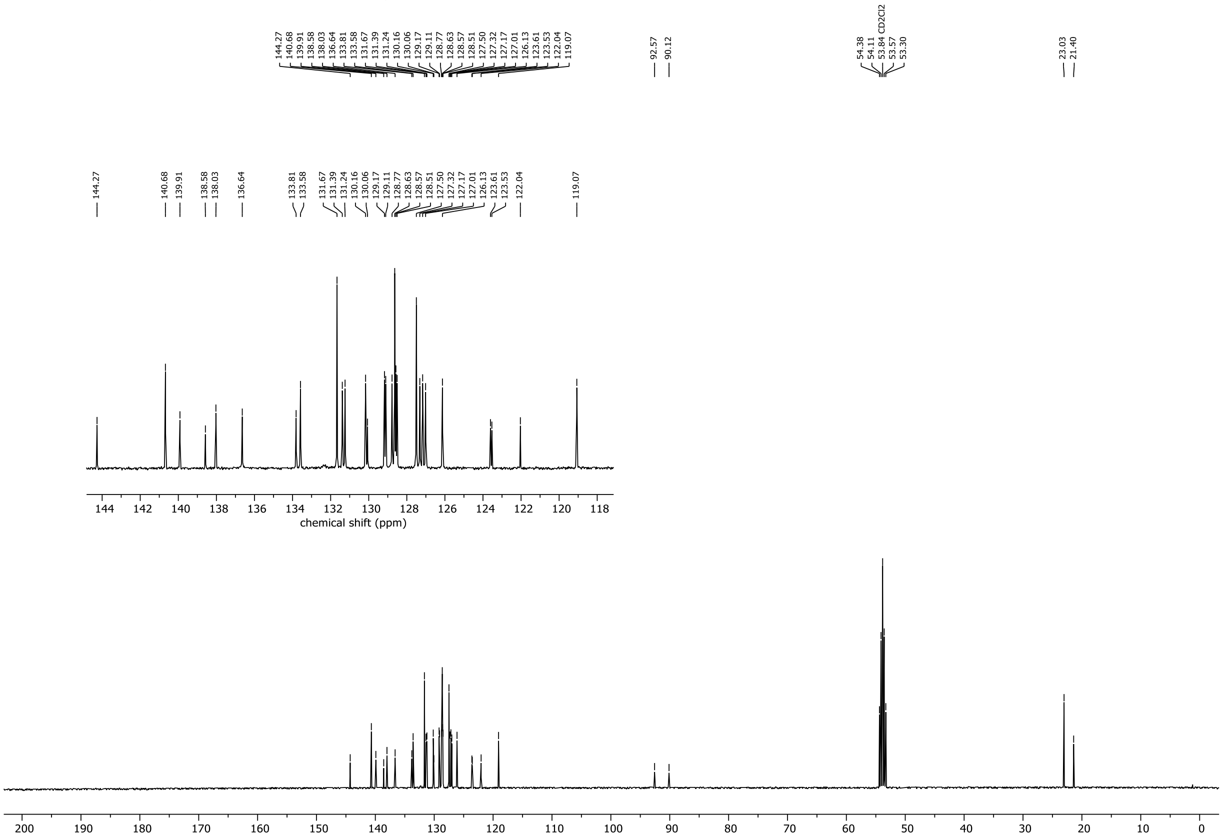

^{11}B NMR (161 MHz, CD_2Cl_2)

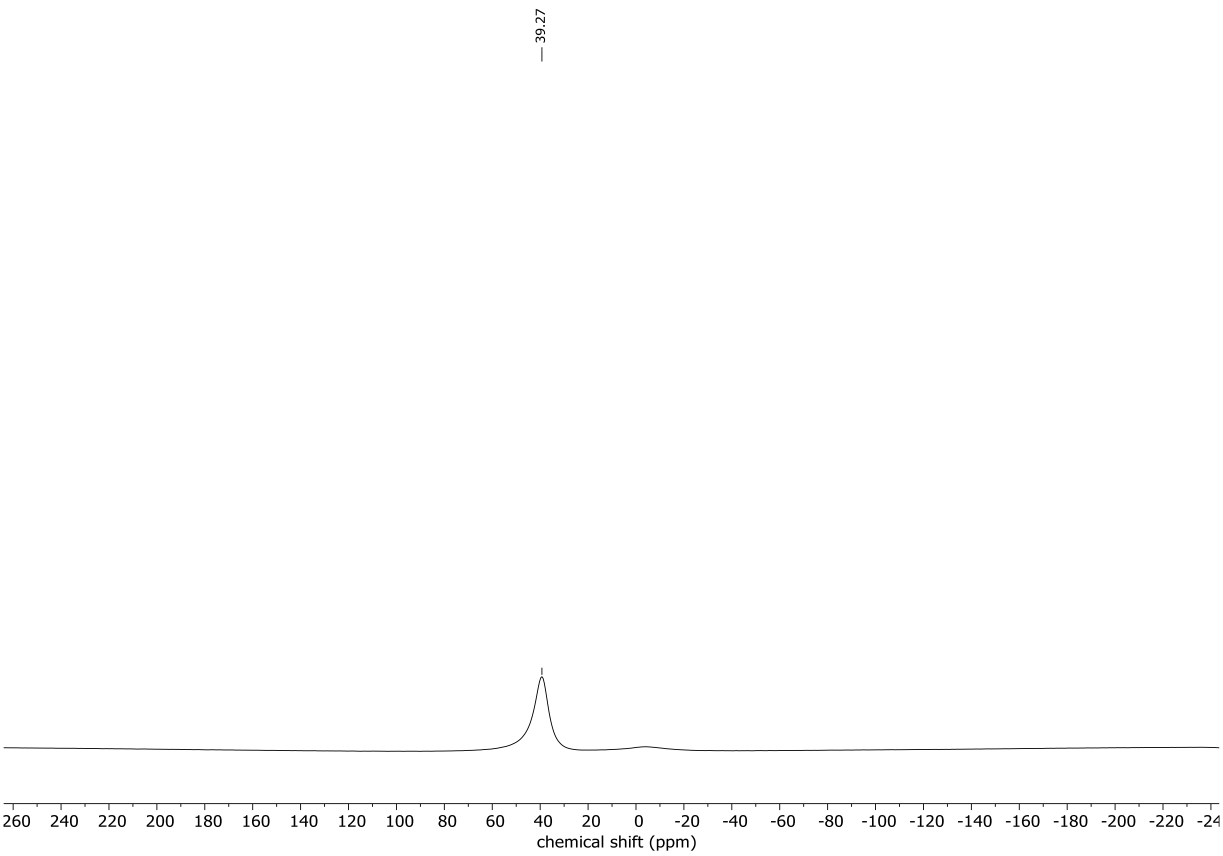

^{1}H NMR (400 MHz, $CDCl_3$) 5b

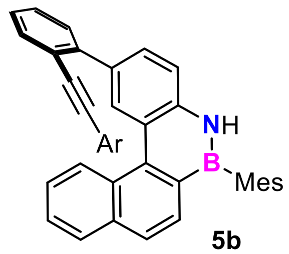

5b

Ar= p-(Me)C_6H_4

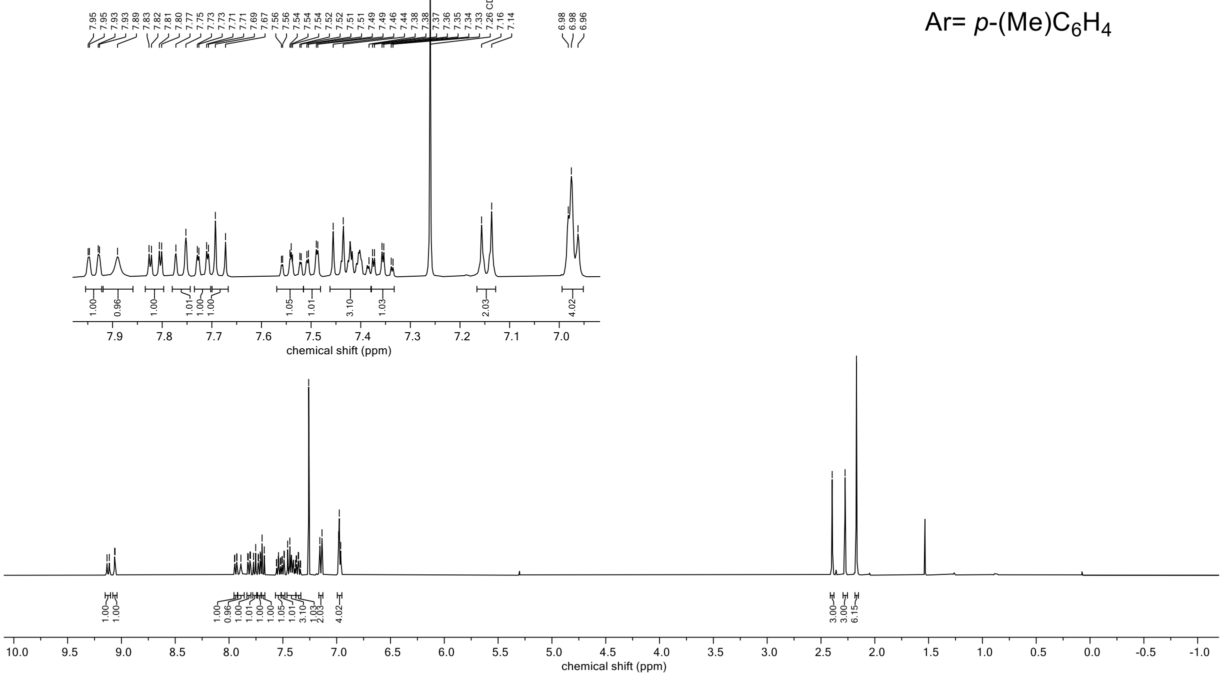

^{13}C NMR (101 MHz, $CDCl_3$)

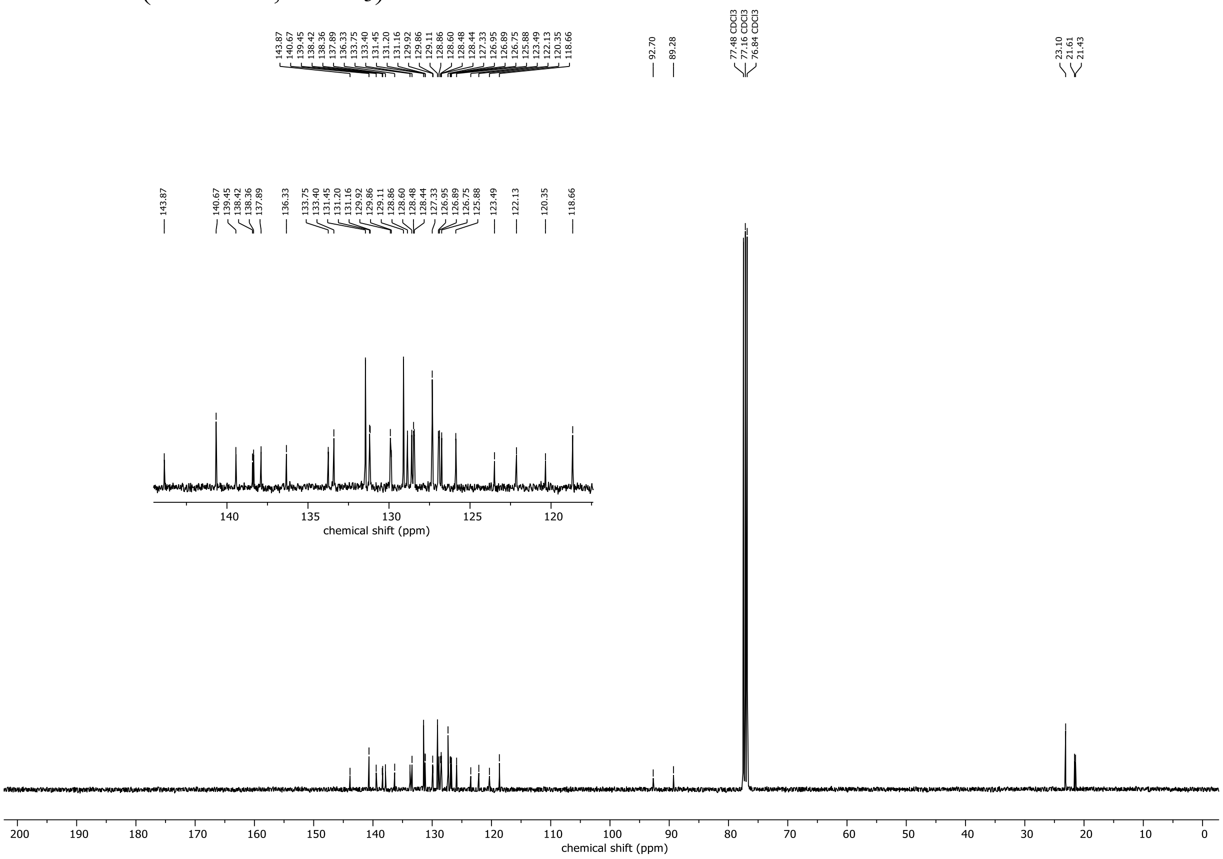

^{11}B NMR (161 MHz, $CDCl_3$)

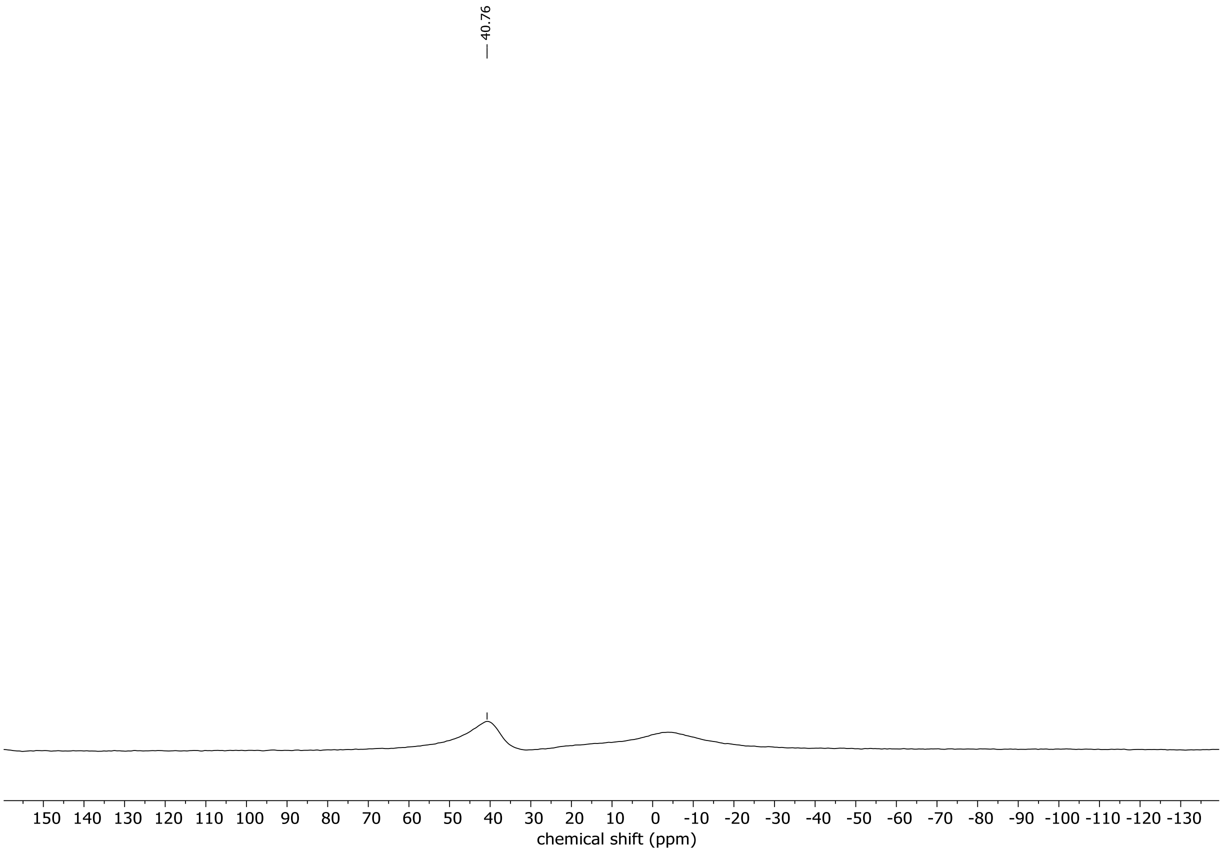

^{1}H NMR (400 MHz, $CDCl_3$) **5c**

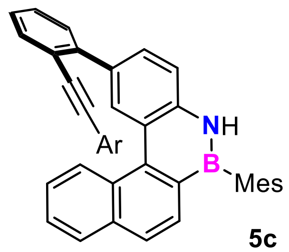

5c

Ar= *p*-(OMe)C_6H_4

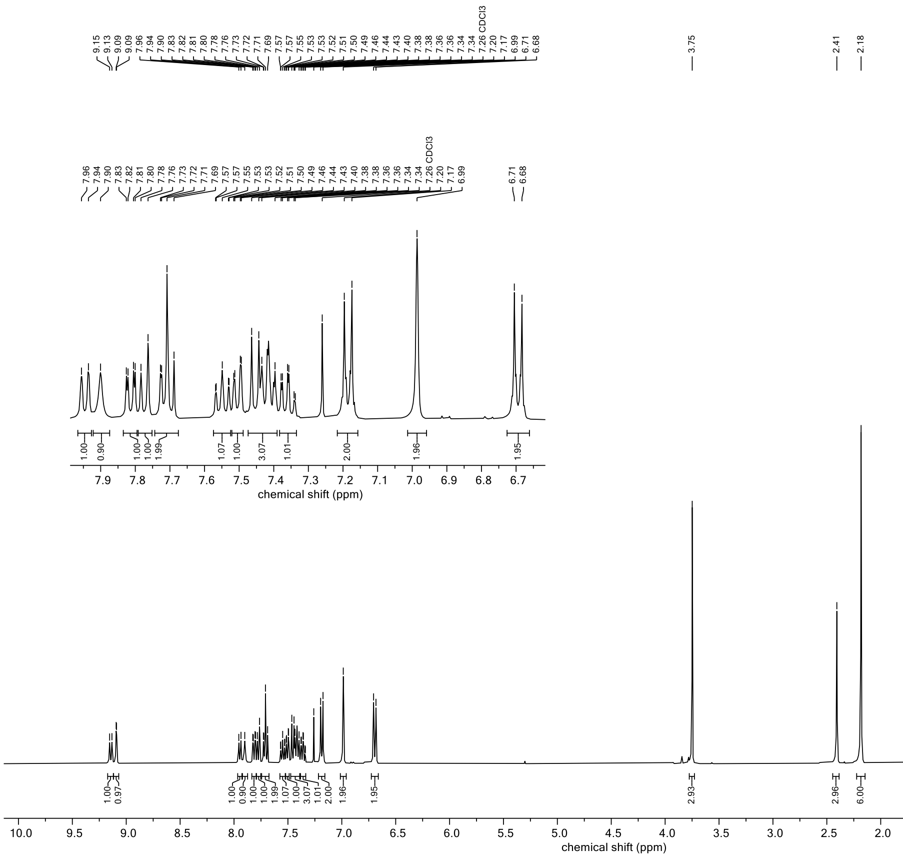

^{13}C NMR (101 MHz, $CDCl_3$)

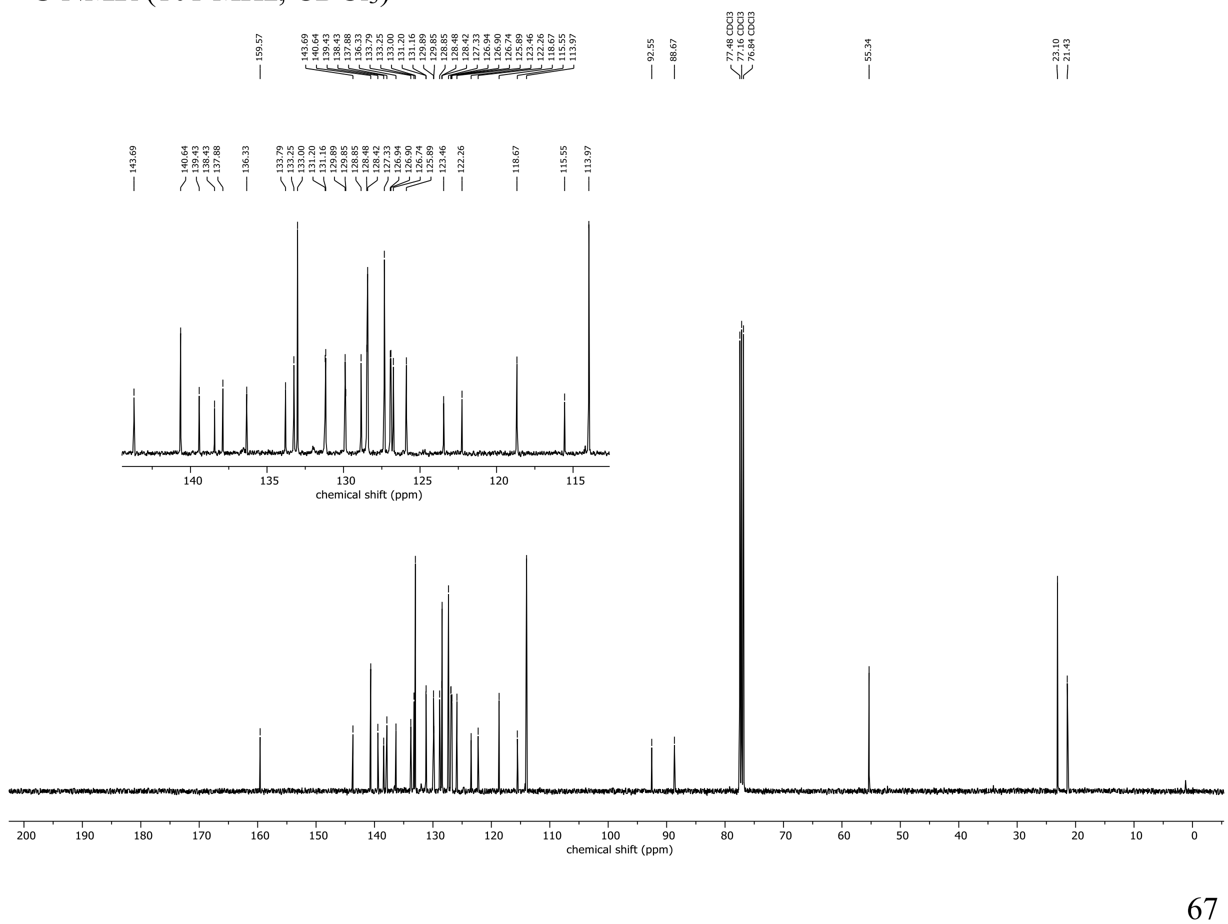

^{11}B NMR (161 MHz, $CDCl_3$)

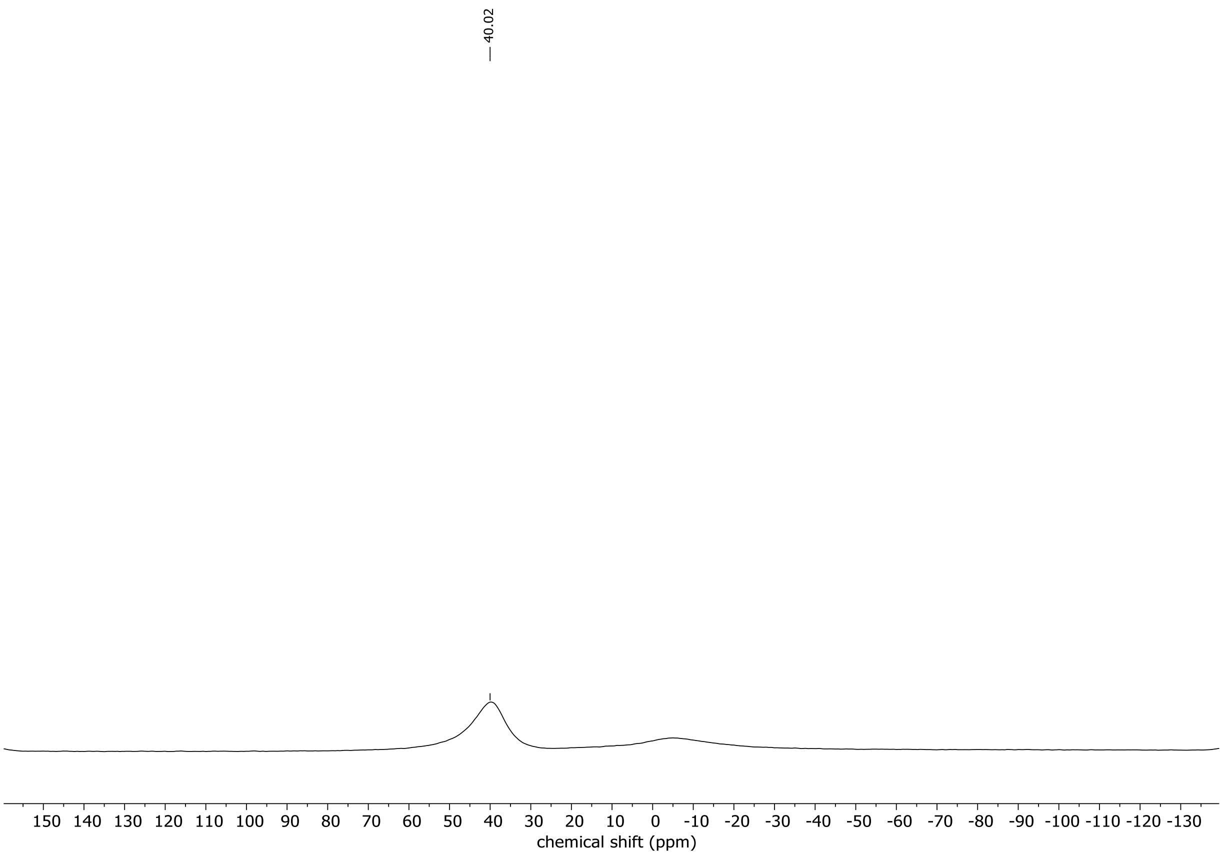

^{1}H NMR (400 MHz, $CDCl_3$) 5d

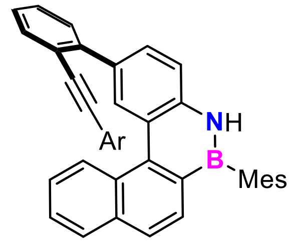

Ar= *p*-(OPh)C_6H_4 **5d**

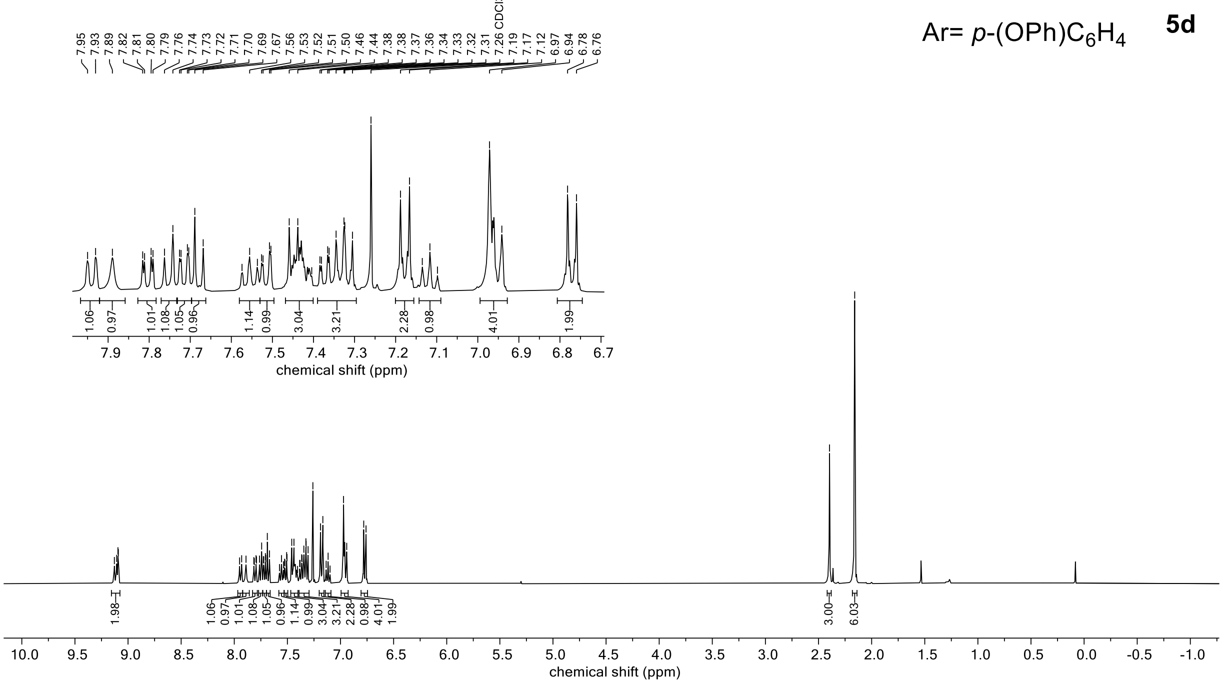

^{13}C NMR (101 MHz, $CDCl_3$)

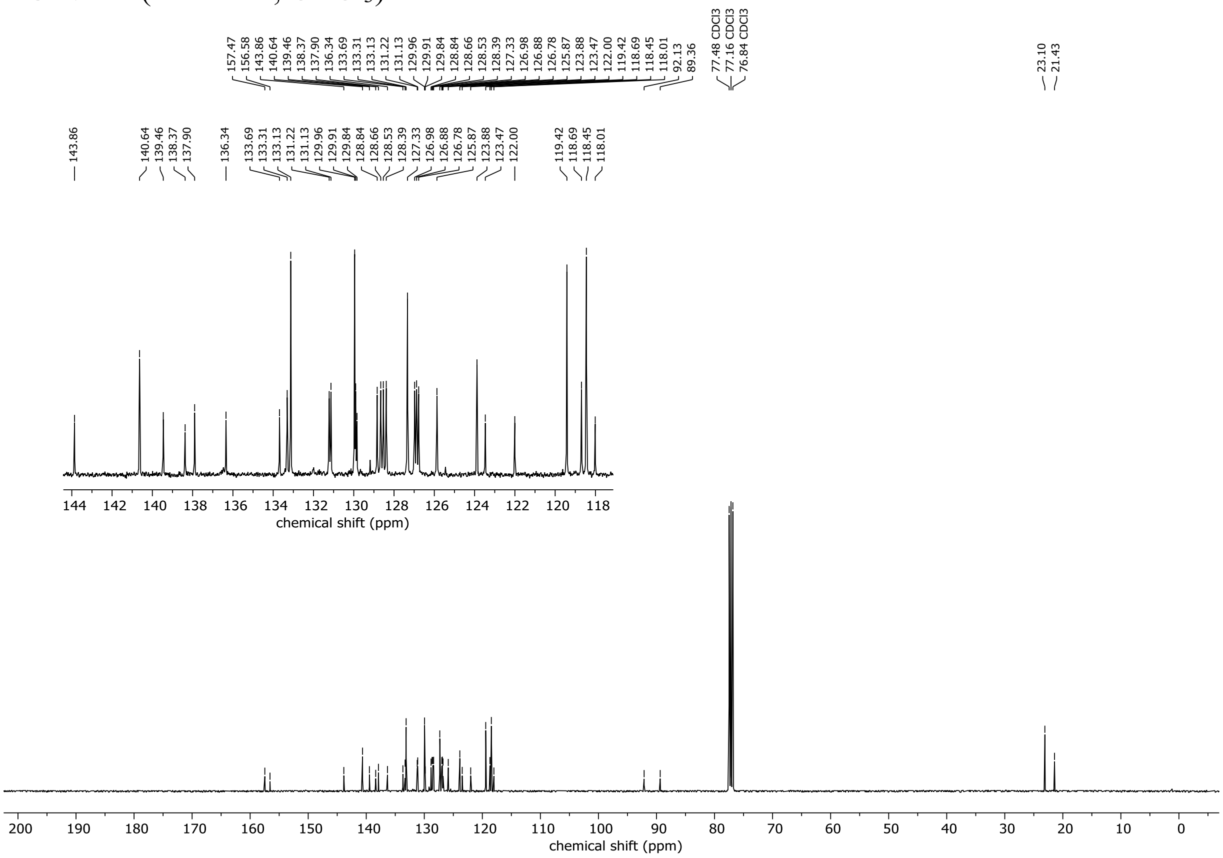

^{11}B NMR (161 MHz, $CDCl_3$)

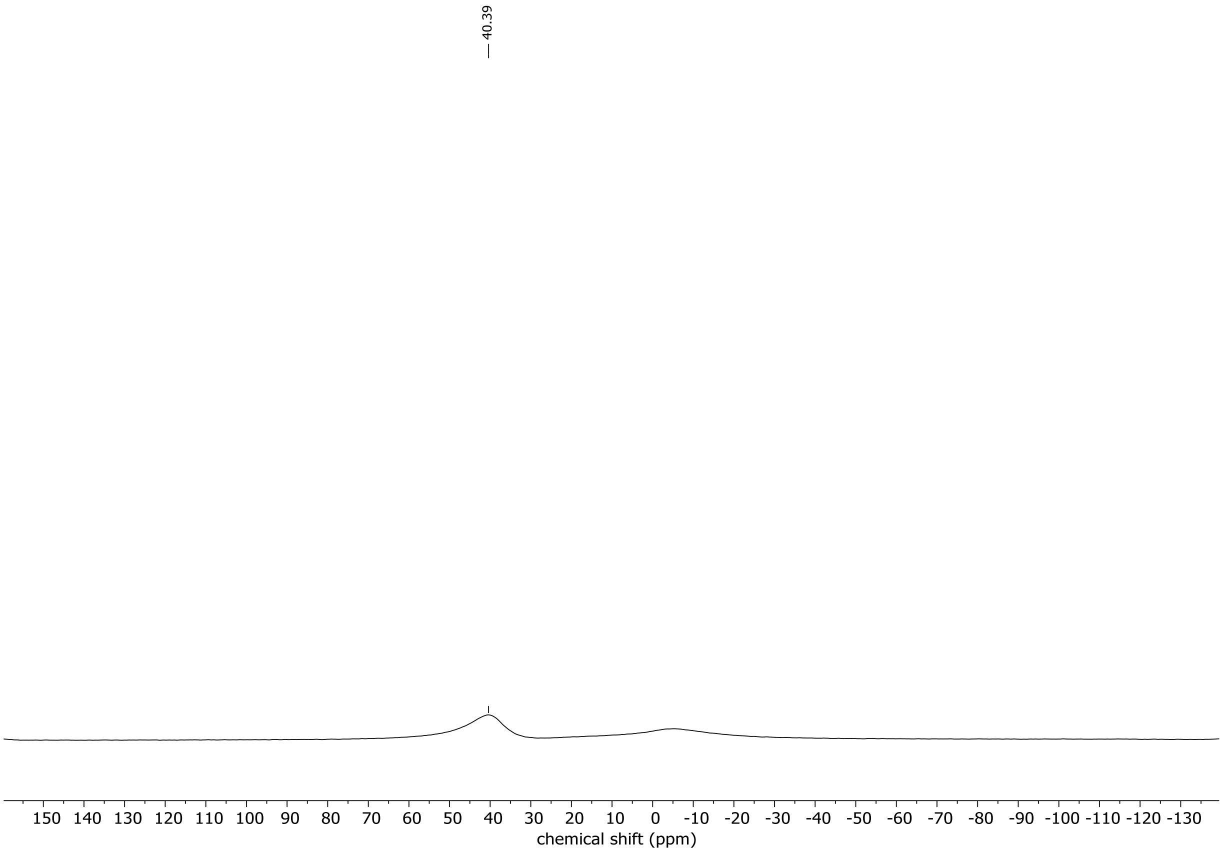

^{1}H NMR (400 MHz, $CDCl_3$) **5e**

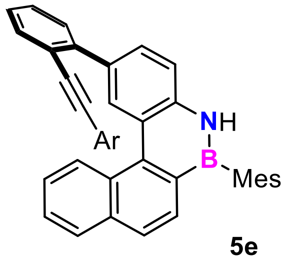

5e

Ar= *p*-(F)C_6H_4

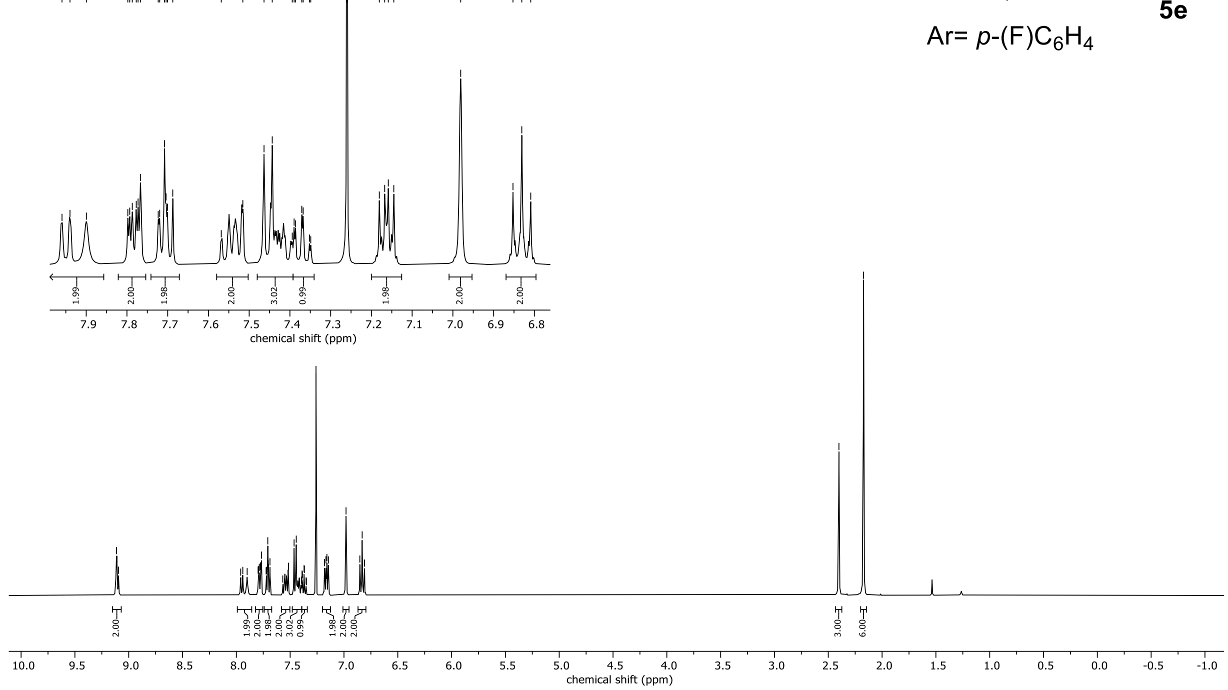

^{13}C NMR (101 MHz, $CDCl_3$)

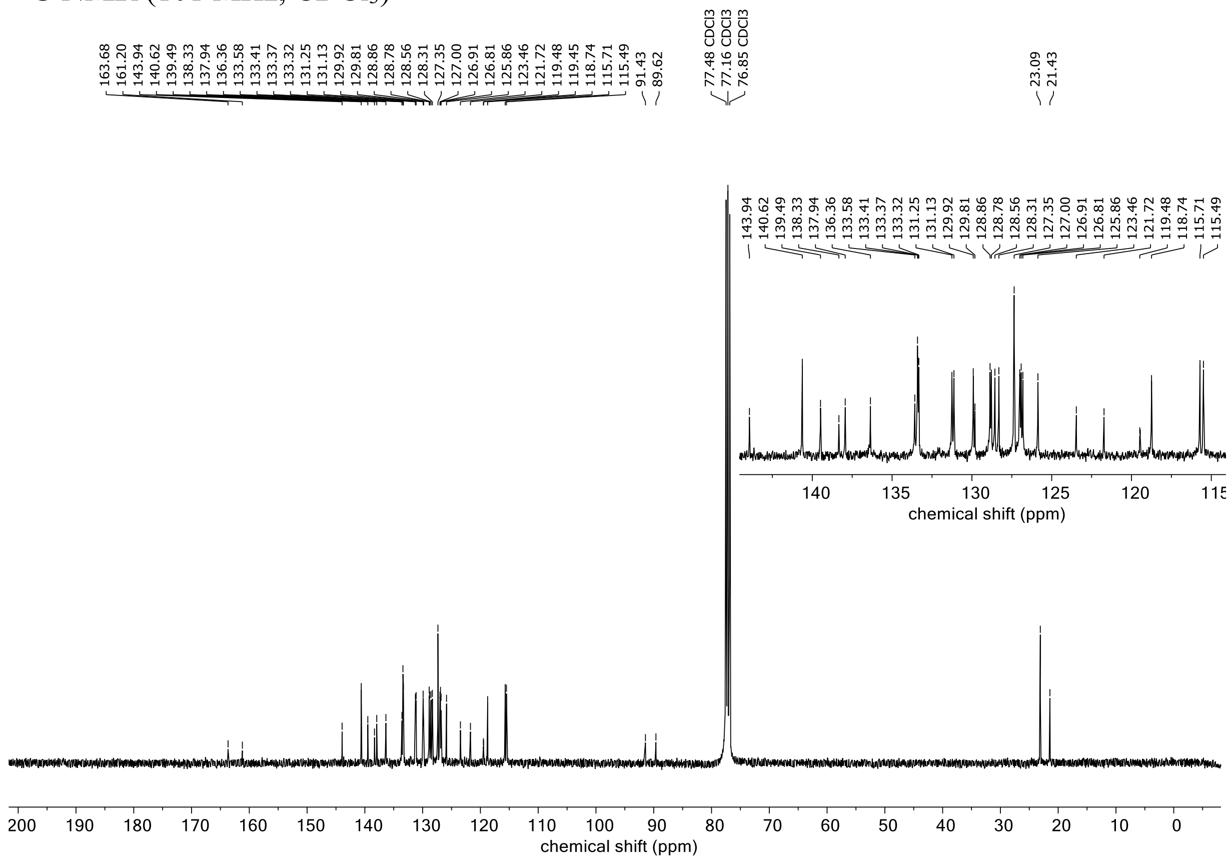

^{11}B NMR (161 MHz, $CDCl_3$)

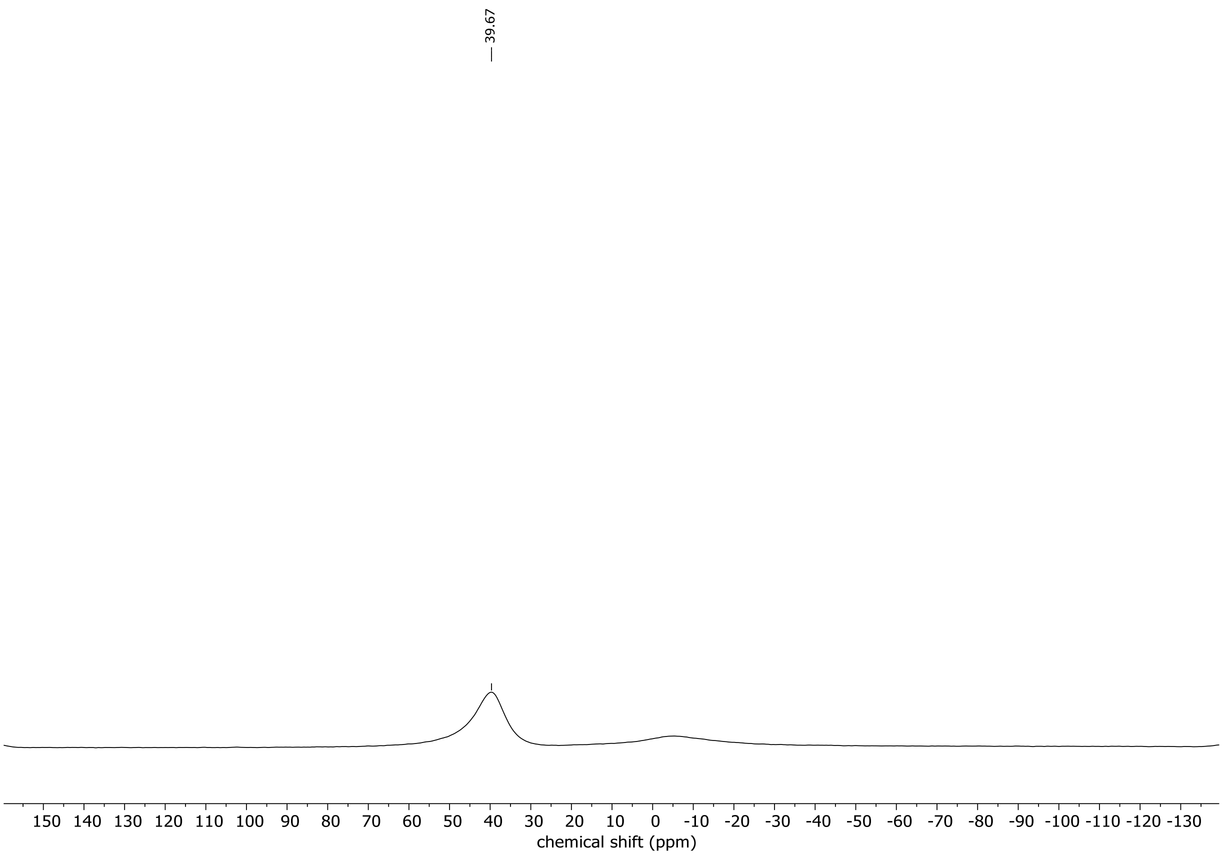

^{19}F NMR (282 MHz, $CDCl_3$)

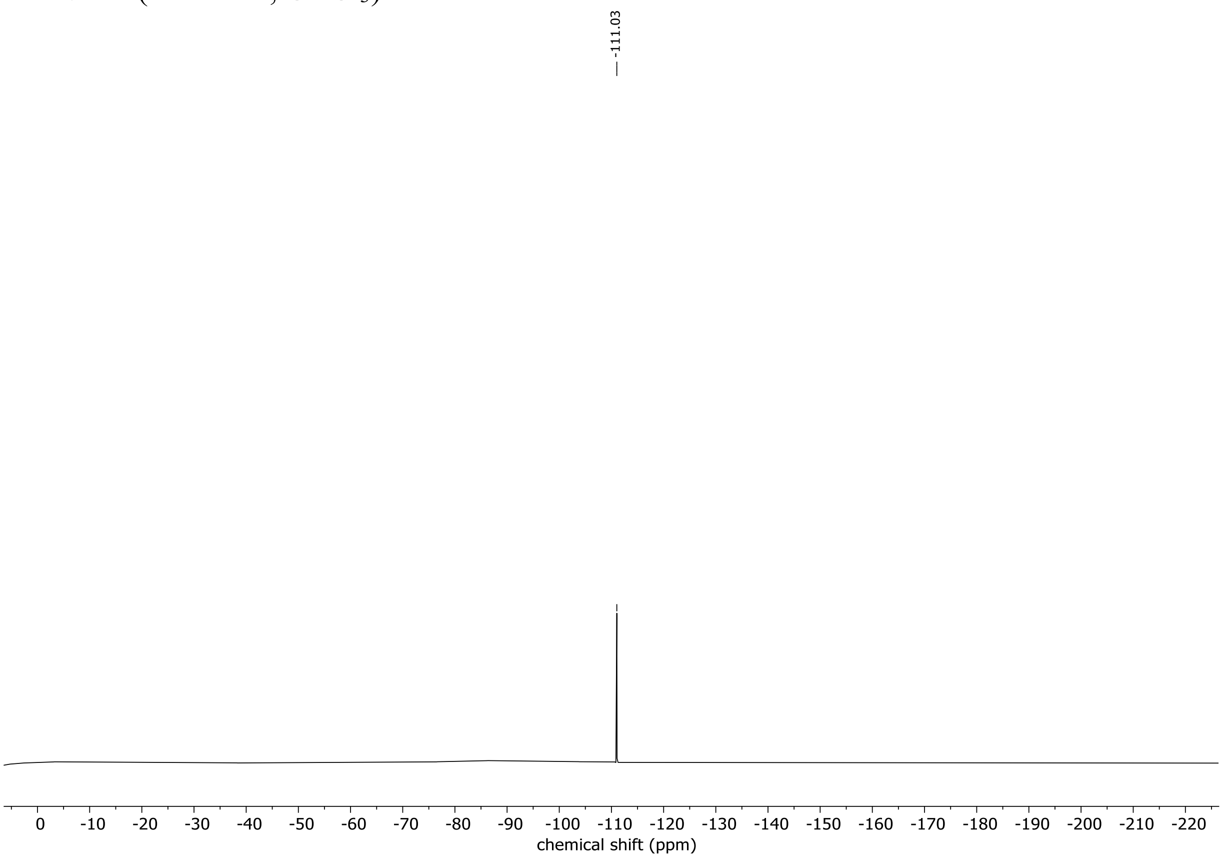

^{1}H NMR (300 MHz, CDCl$_3$) 5f

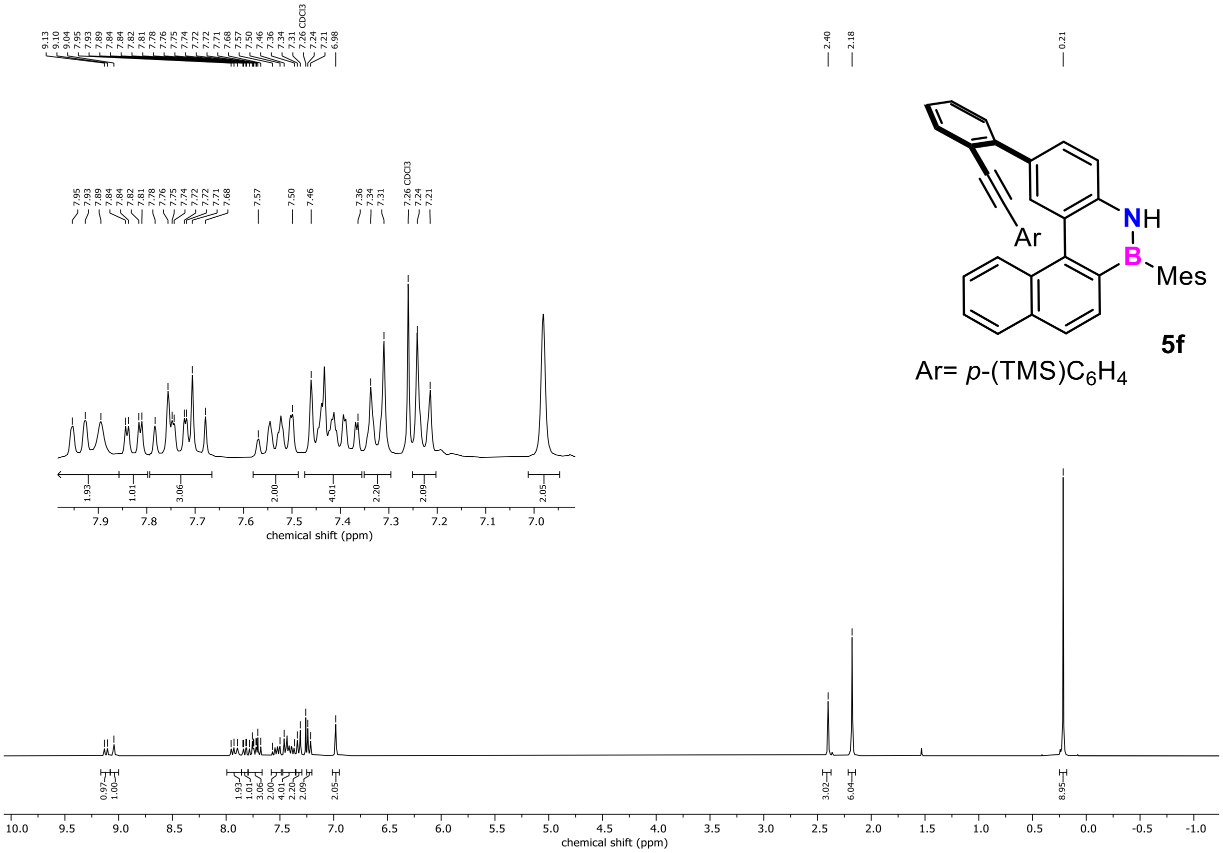

^{13}C NMR (101 MHz, CDCl$_3$)

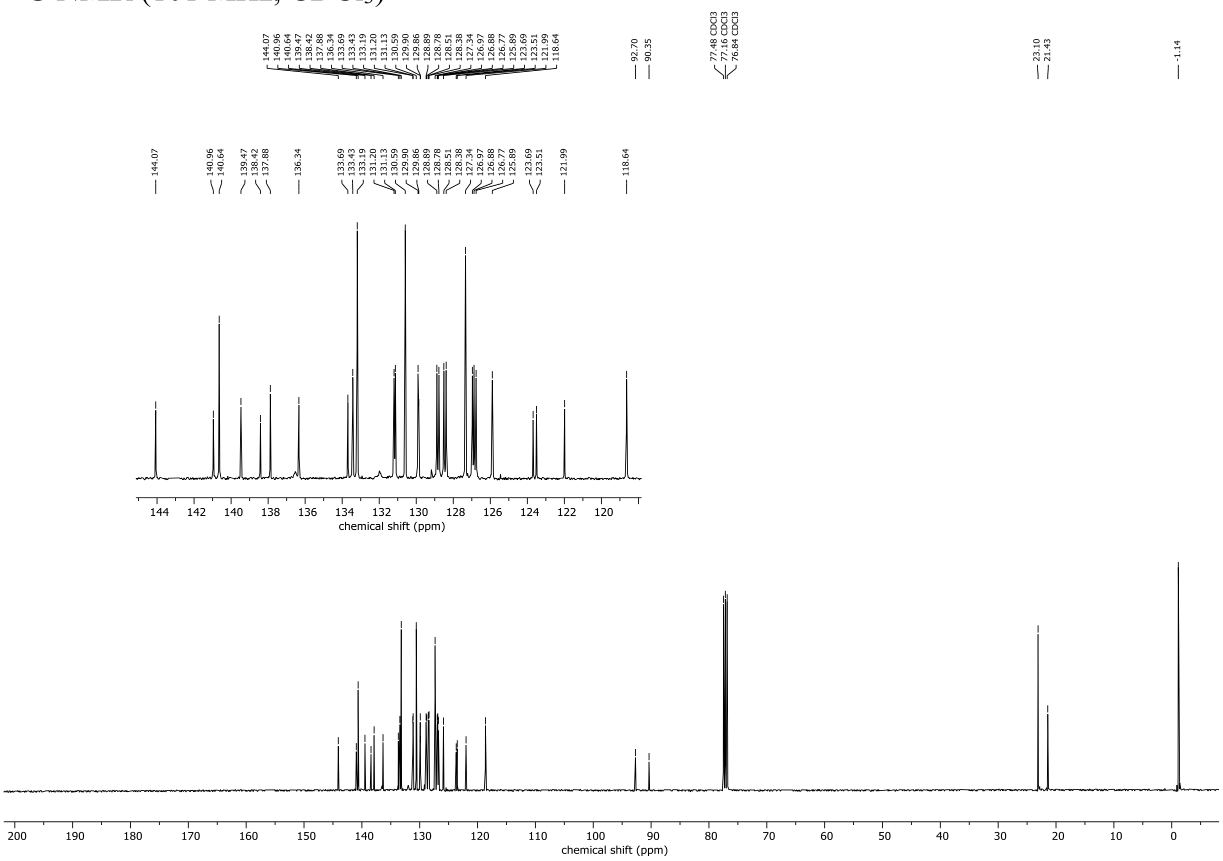

^{11}B NMR (161 MHz, $CDCl_3$)

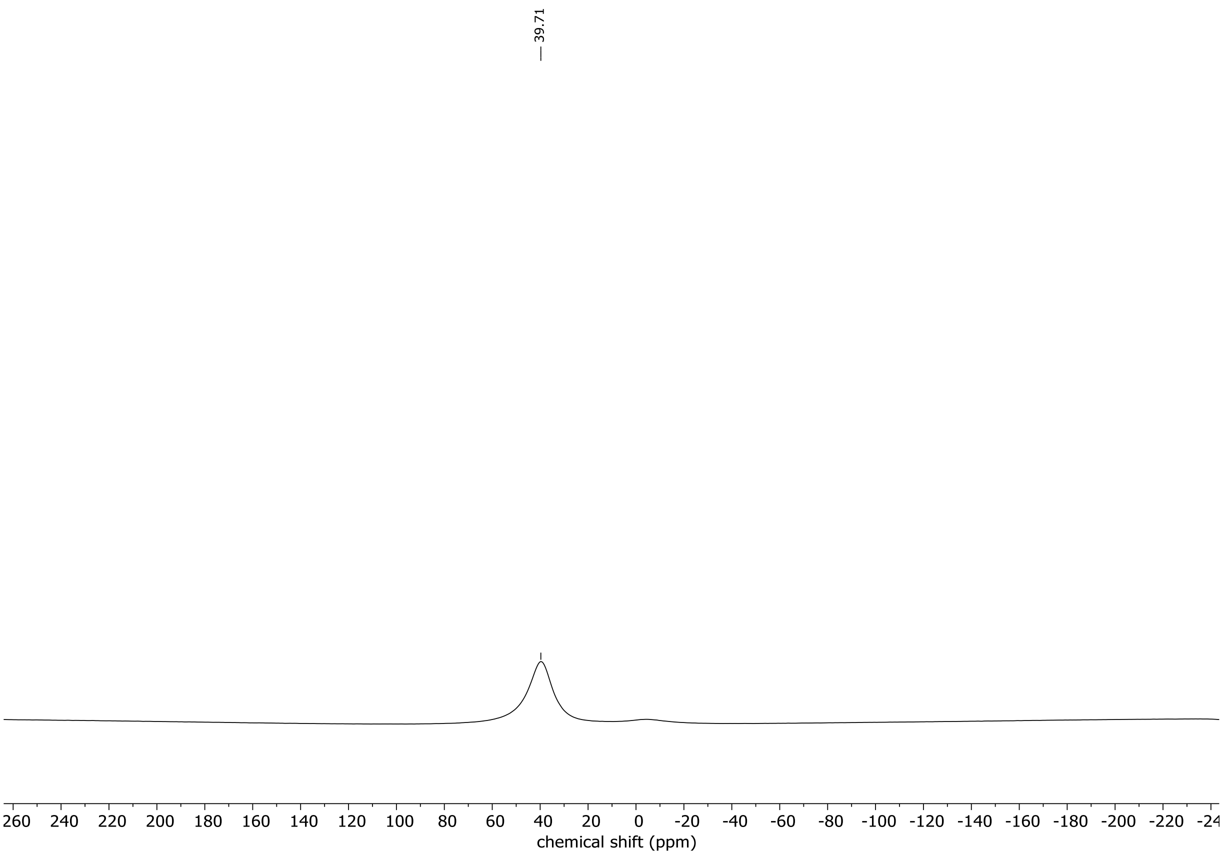

^{1}H NMR (600 MHz, CD_2Cl_2) **5g**

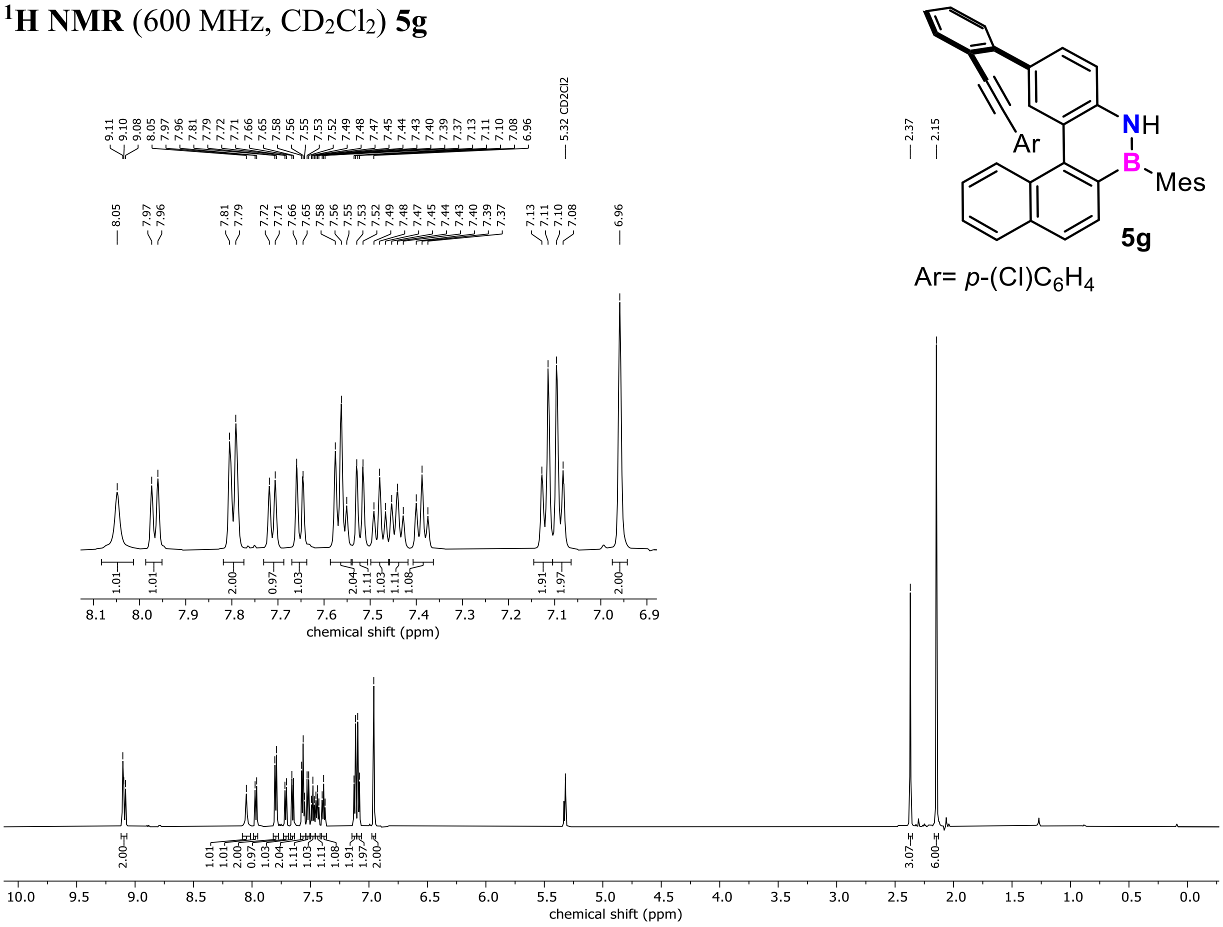

^{13}C NMR (101 MHz, $CDCl_3$)

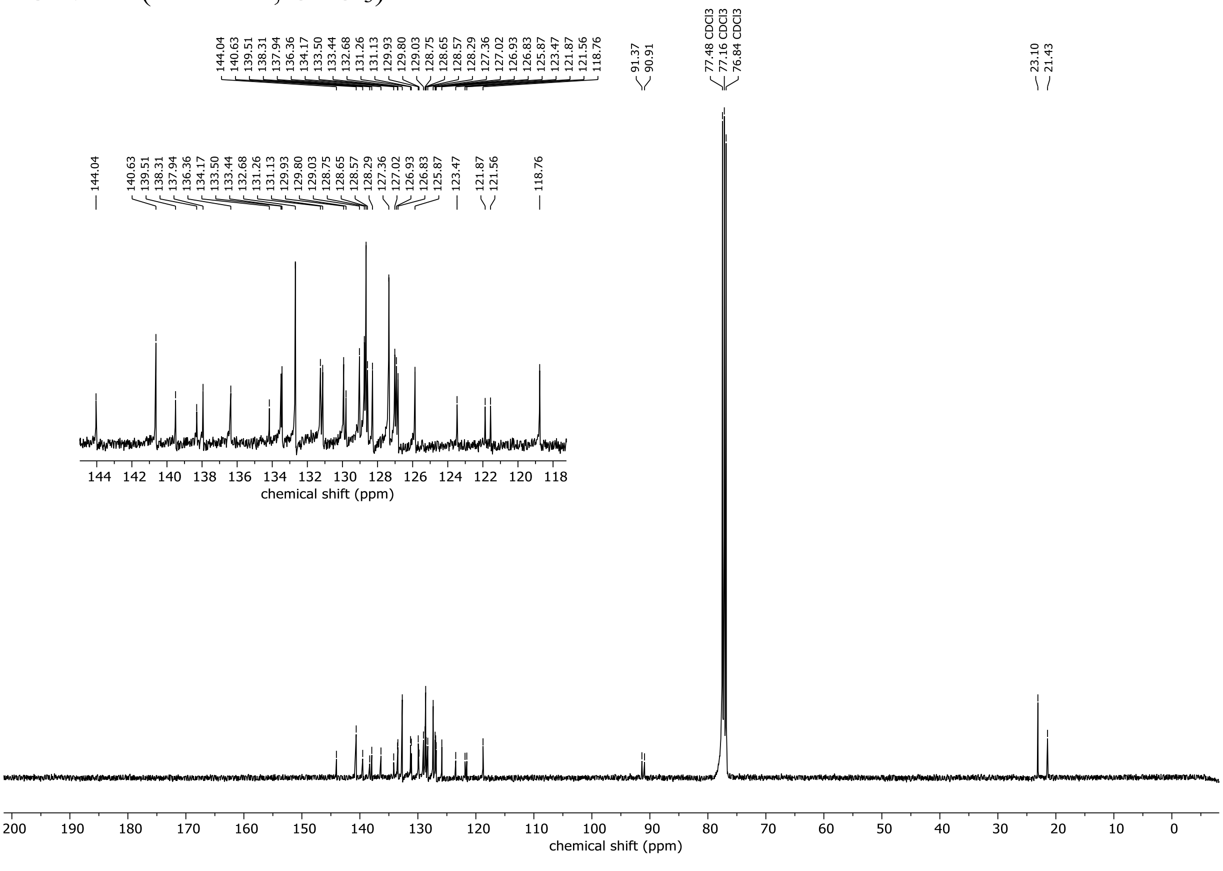

^{11}B NMR (161 MHz, $CDCl_3$)

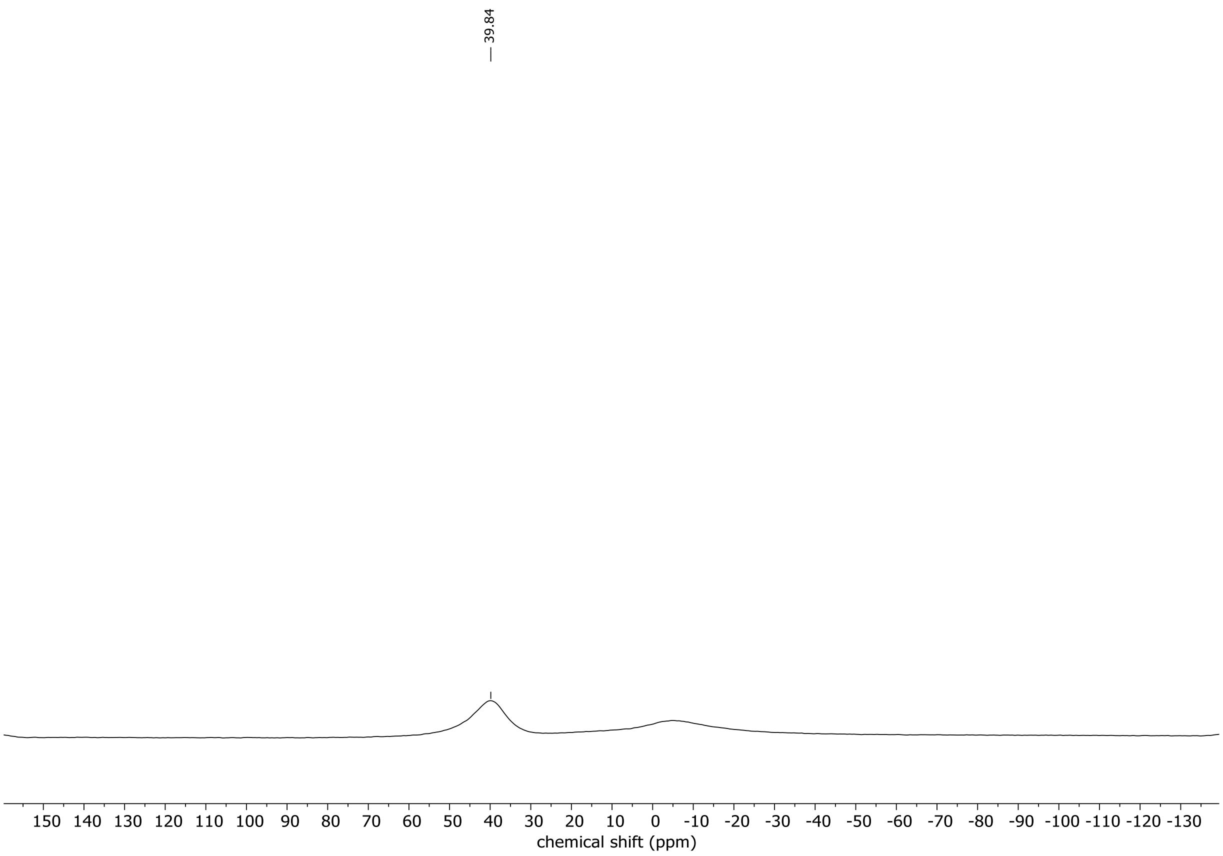

^{1}H NMR (300 MHz, $CDCl_3$) **5h**

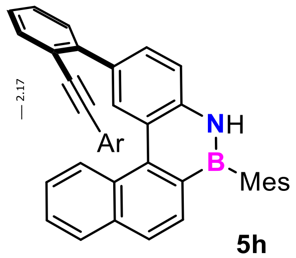

5h

Ar= *p*-(CF_3)C_6H_4

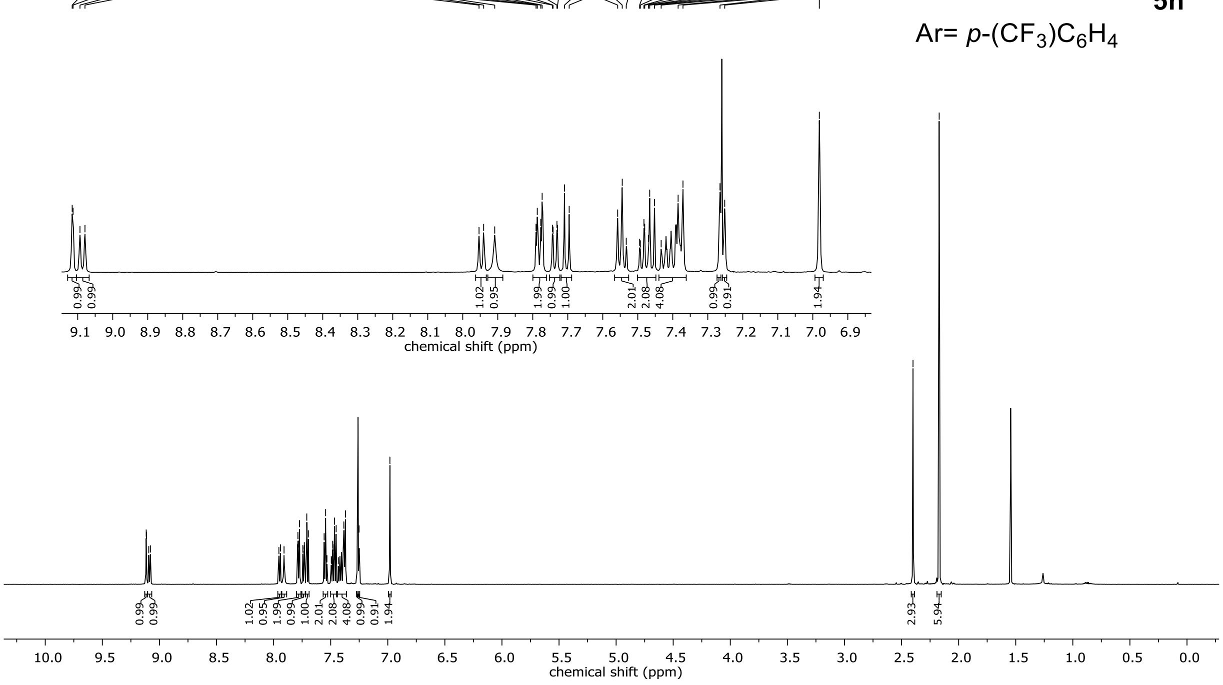

^{13}C NMR (126 MHz, $CDCl_3$)

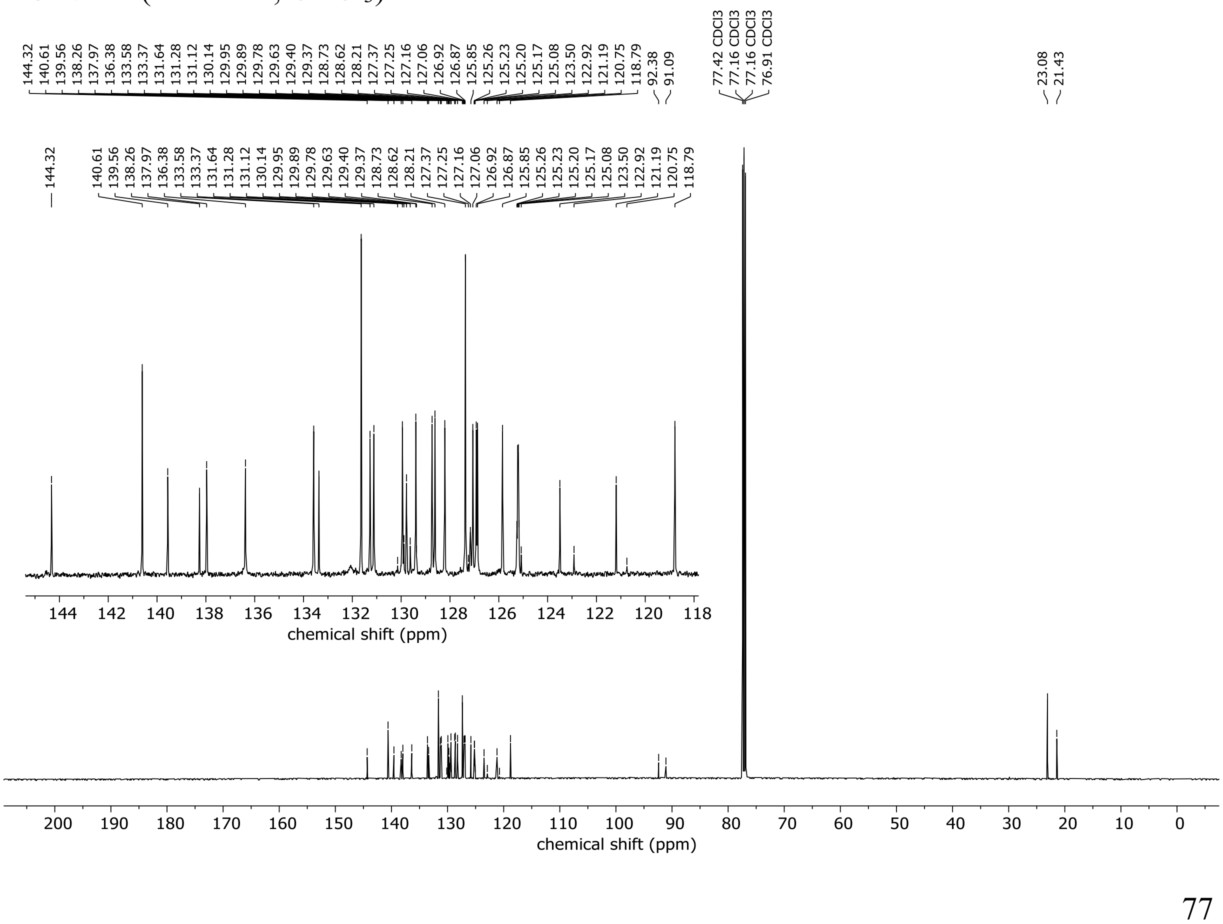

^{11}B NMR (161 MHz, $CDCl_3$)

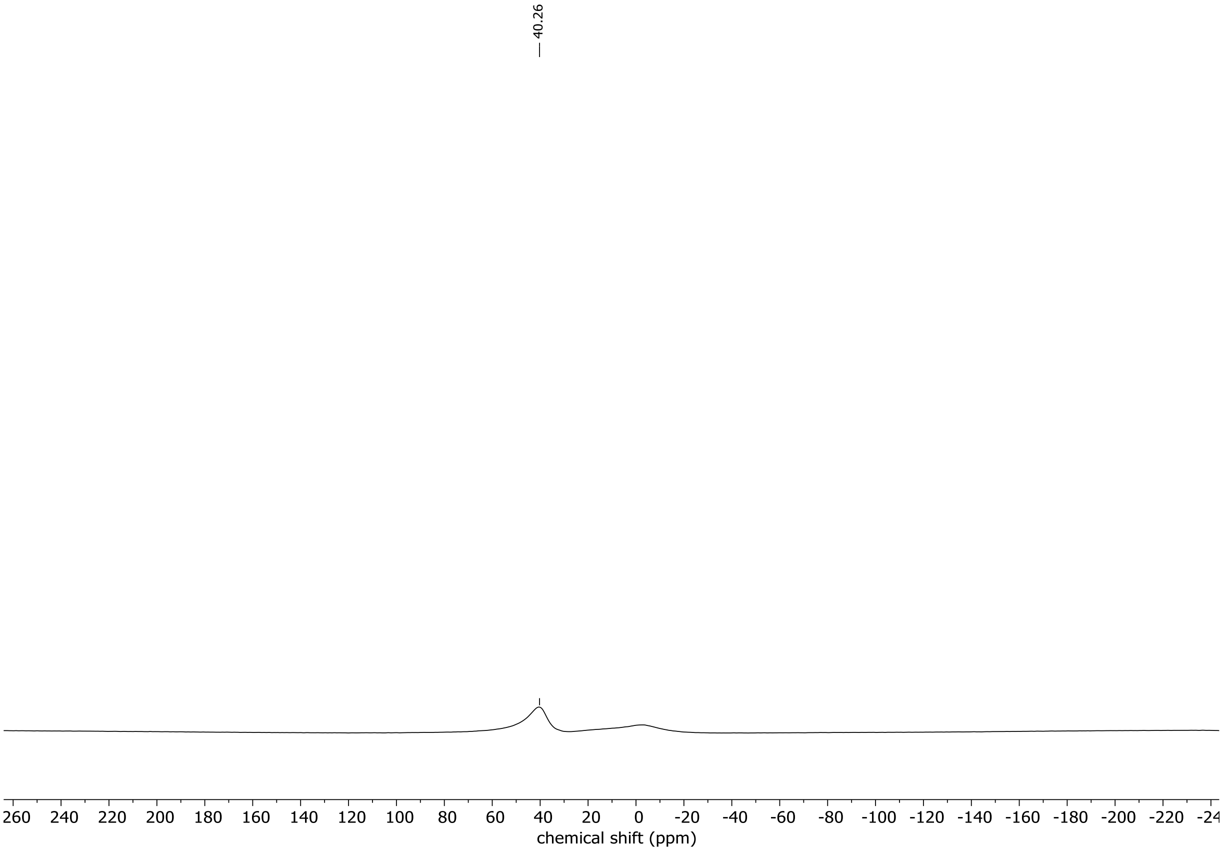

^{19}F NMR (377 MHz, $CDCl_3$)

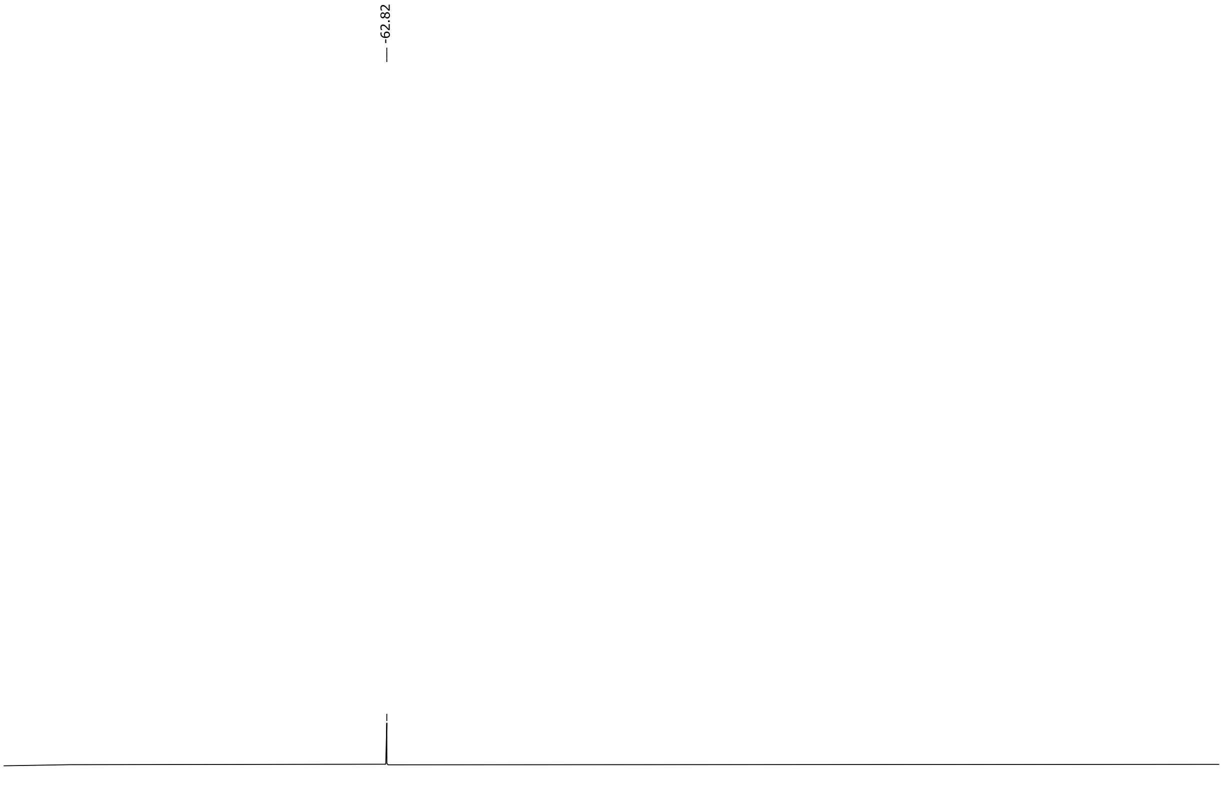

^{1}H NMR (300 MHz, $CDCl_3$) 5i

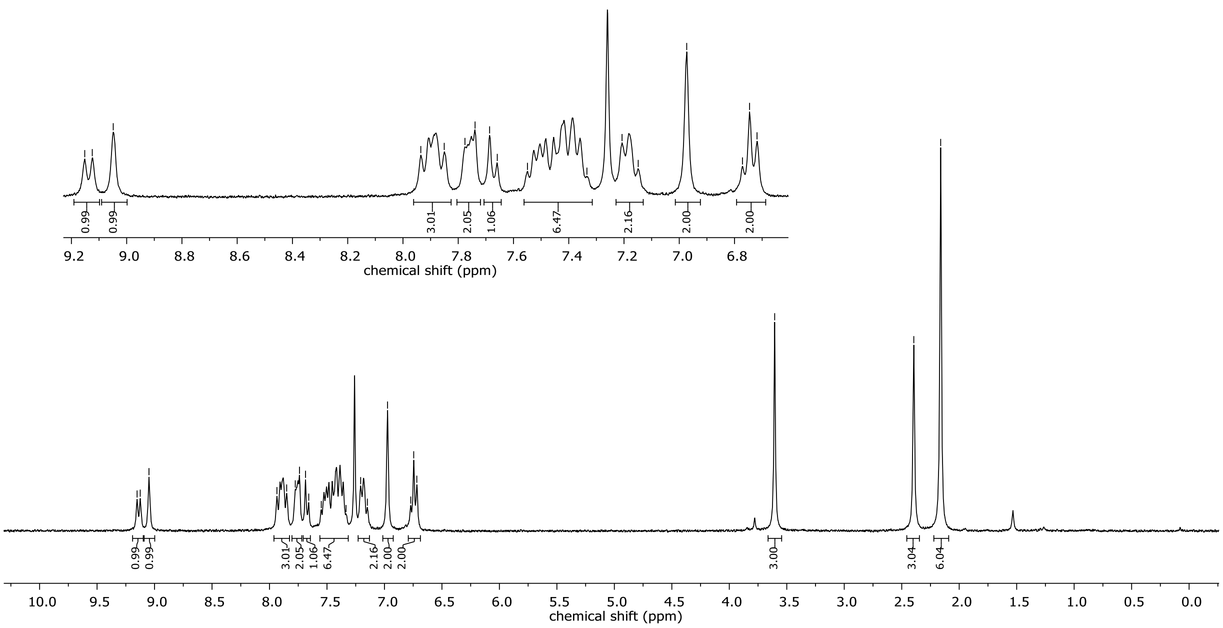

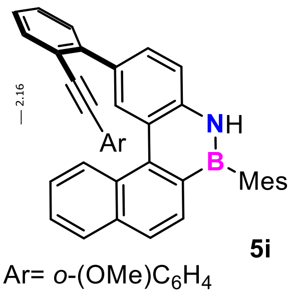

^{13}C NMR (101 MHz, $CDCl_3$)

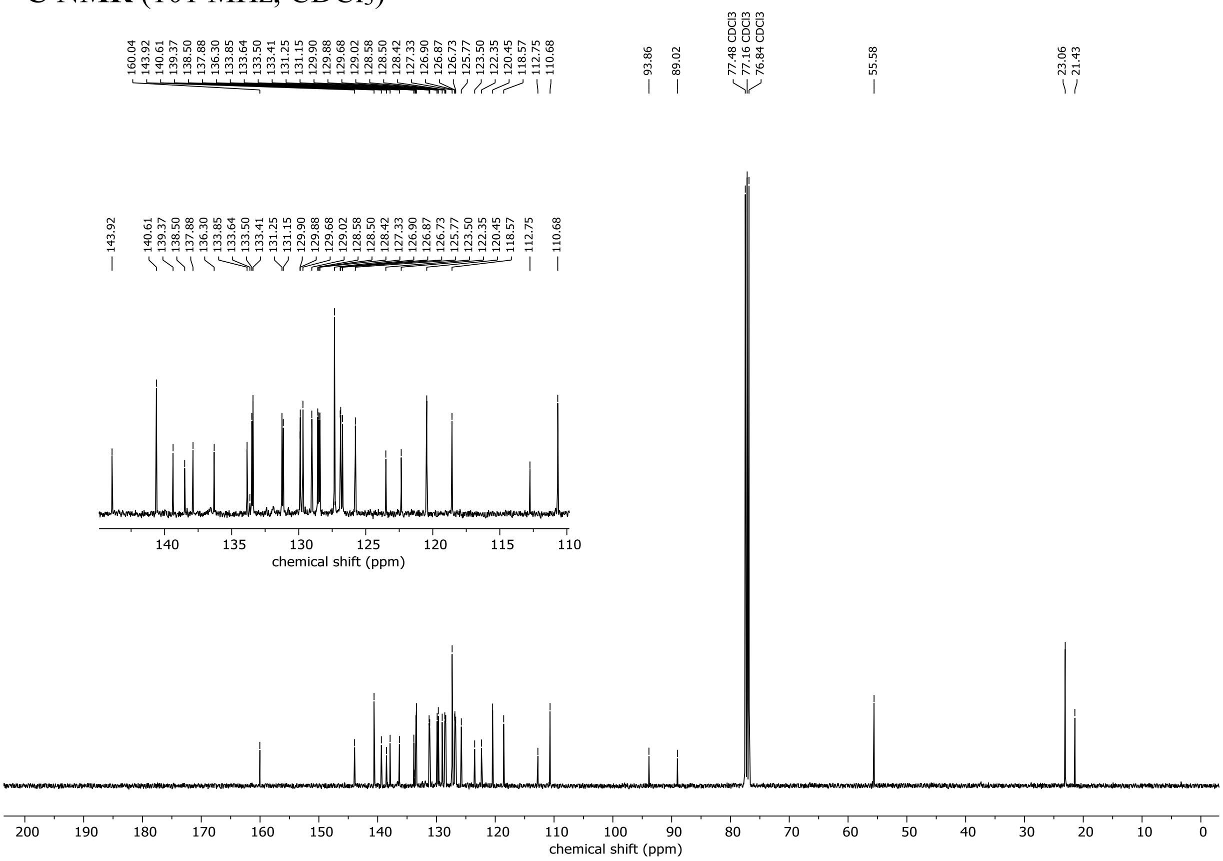

^{11}B NMR (161 MHz, $CDCl_3$)

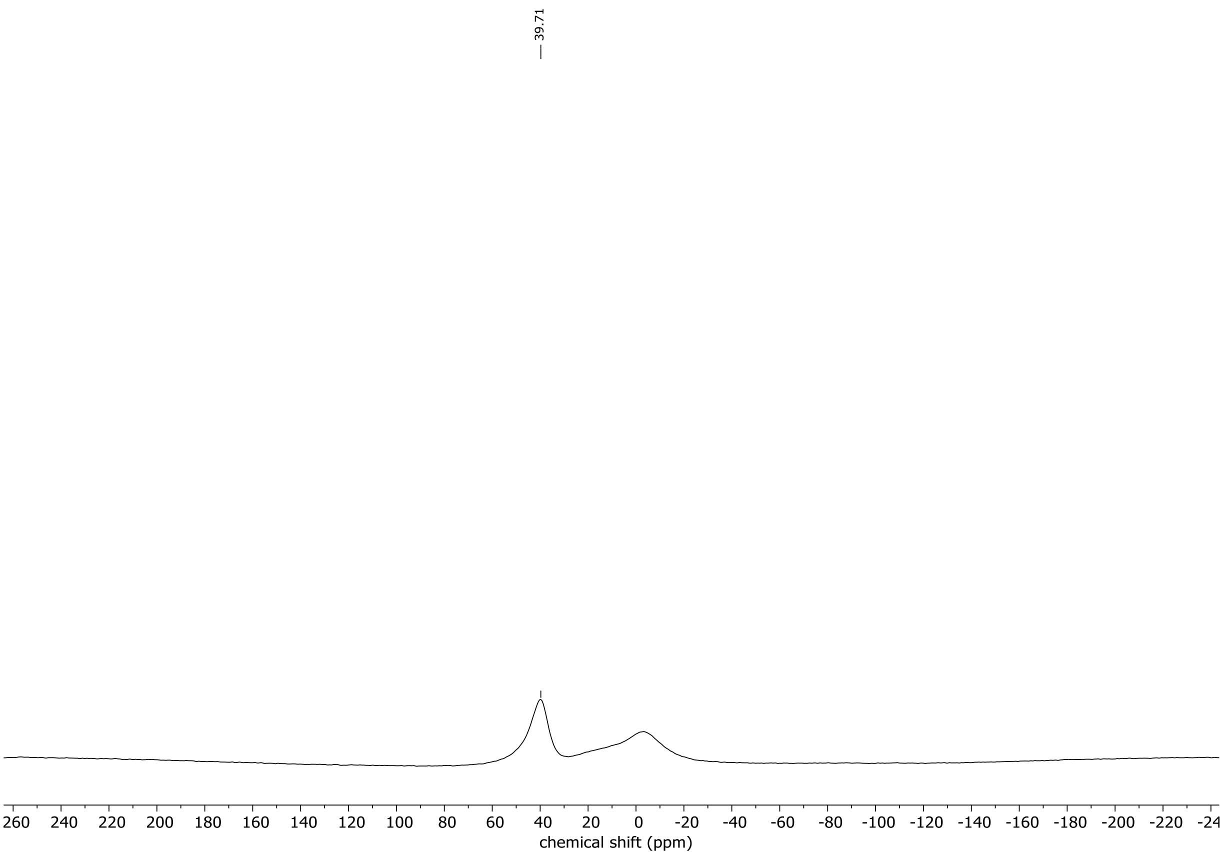

^{1}H NMR (400 MHz, $CDCl_3$) **5j**

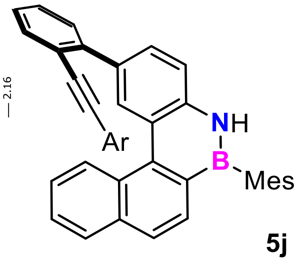

5j

Ar= *m*-(OMe)C_6H_4

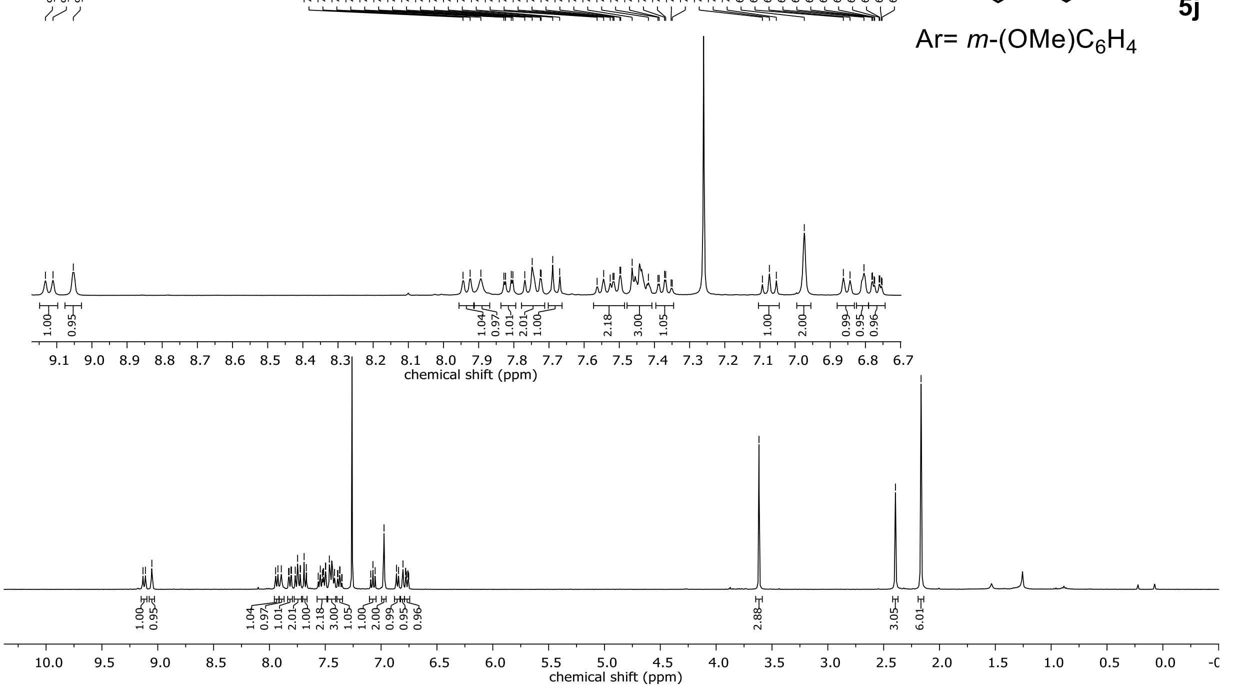

^{13}C NMR (101 MHz, $CDCl_3$)

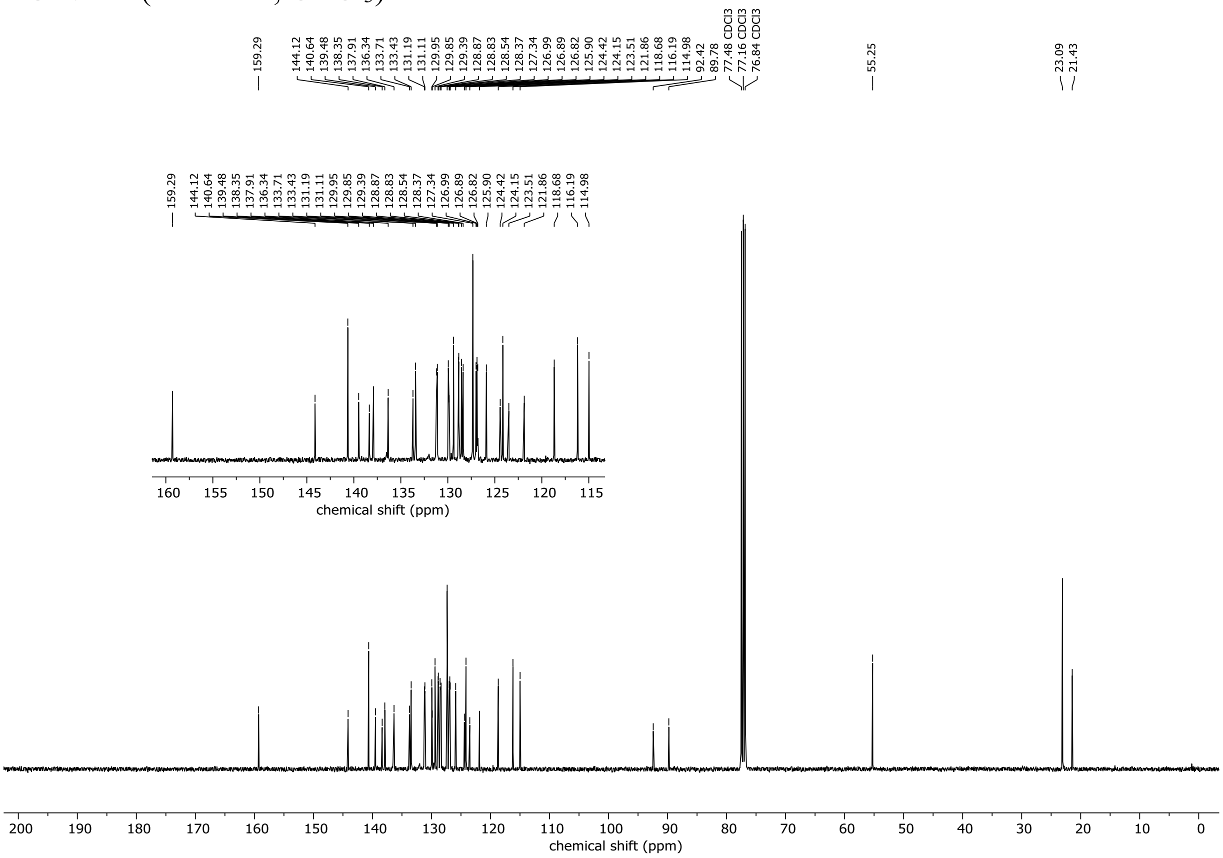

^{11}B NMR (161 MHz, $CDCl_3$)

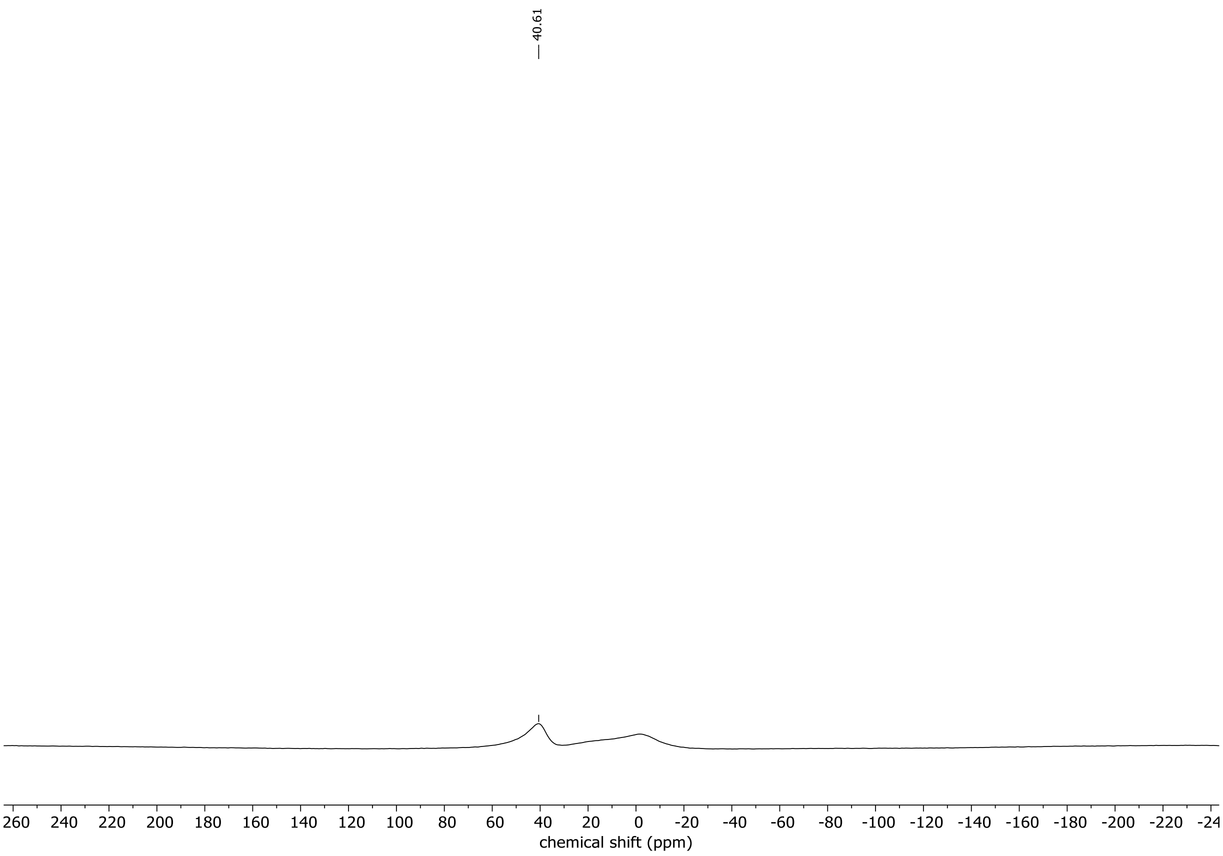

^{1}H NMR (300 MHz, CD_2Cl_2) **S5b(BPh)**

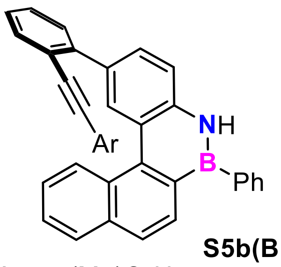

S5b(BPh)

Ar= *p*-(Me)C_6H_4

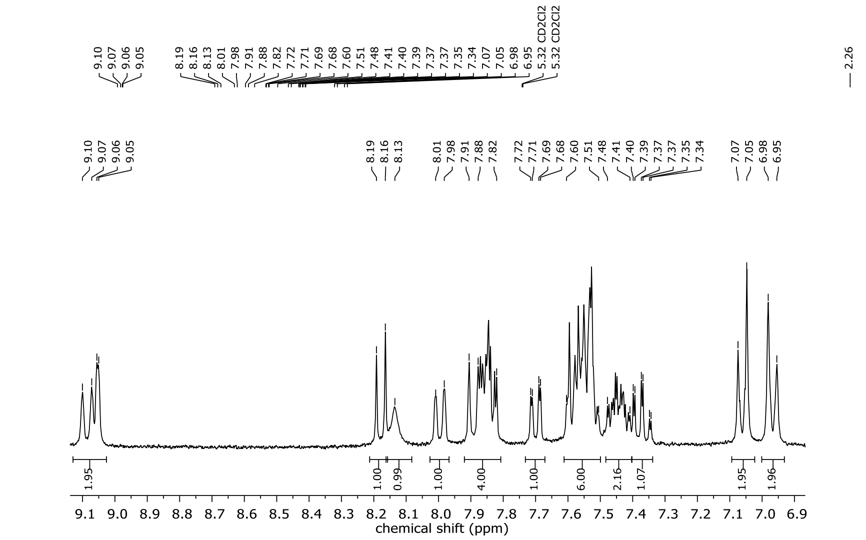

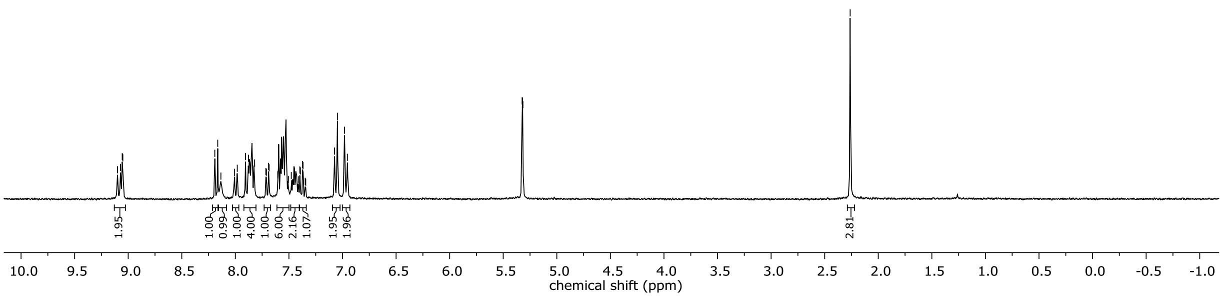

^{13}C NMR (101 MHz, CD_2Cl_2)

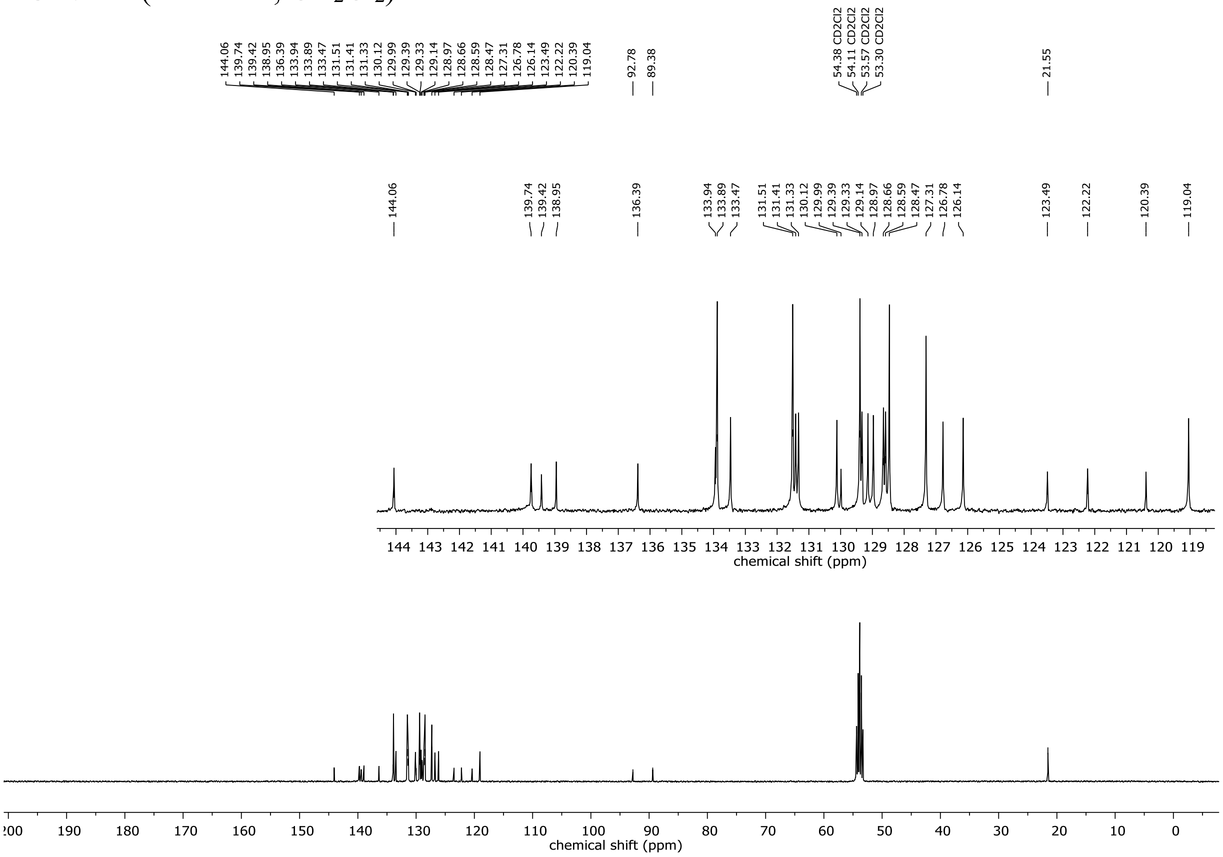

^{11}B NMR (161 MHz, CD_2Cl_2)

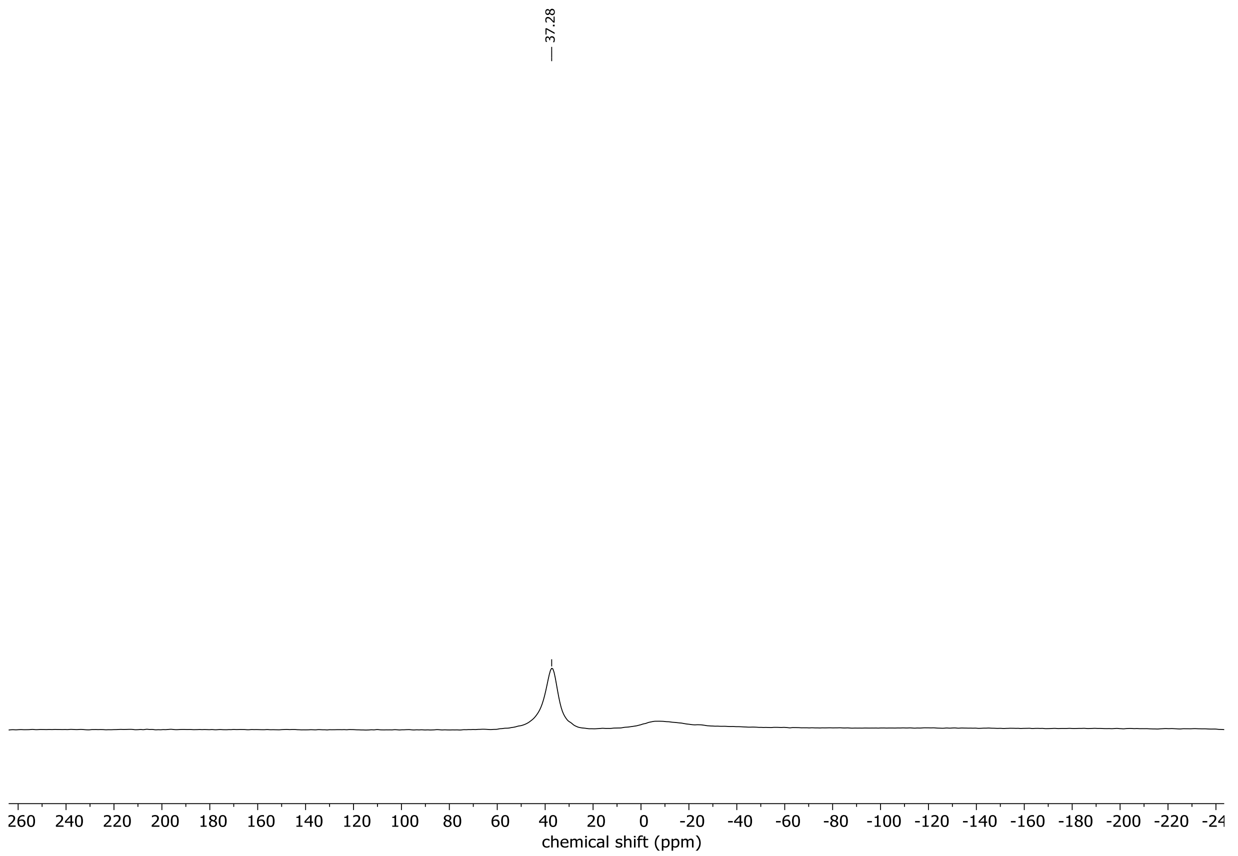

^{1}H NMR (600 MHz, $CDCl_3$) **S5b(NMe)**

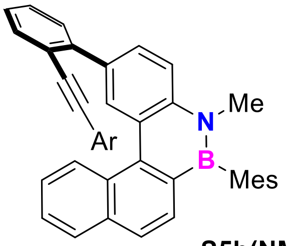

S5b(NMe)

Ar= p-(Me)C_6H_4

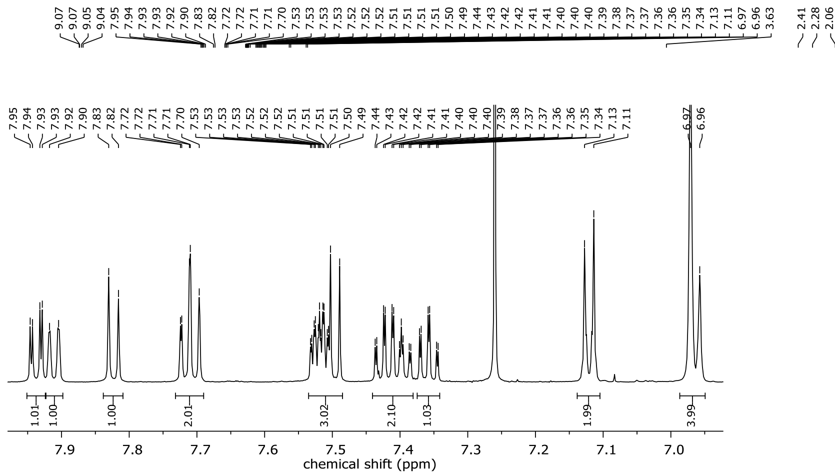

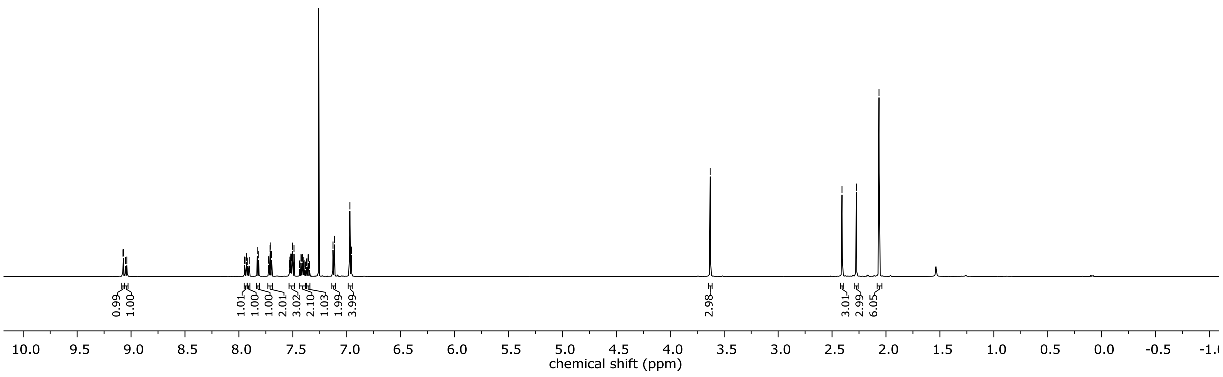

^{13}C NMR (101 MHz, $CDCl_3$)

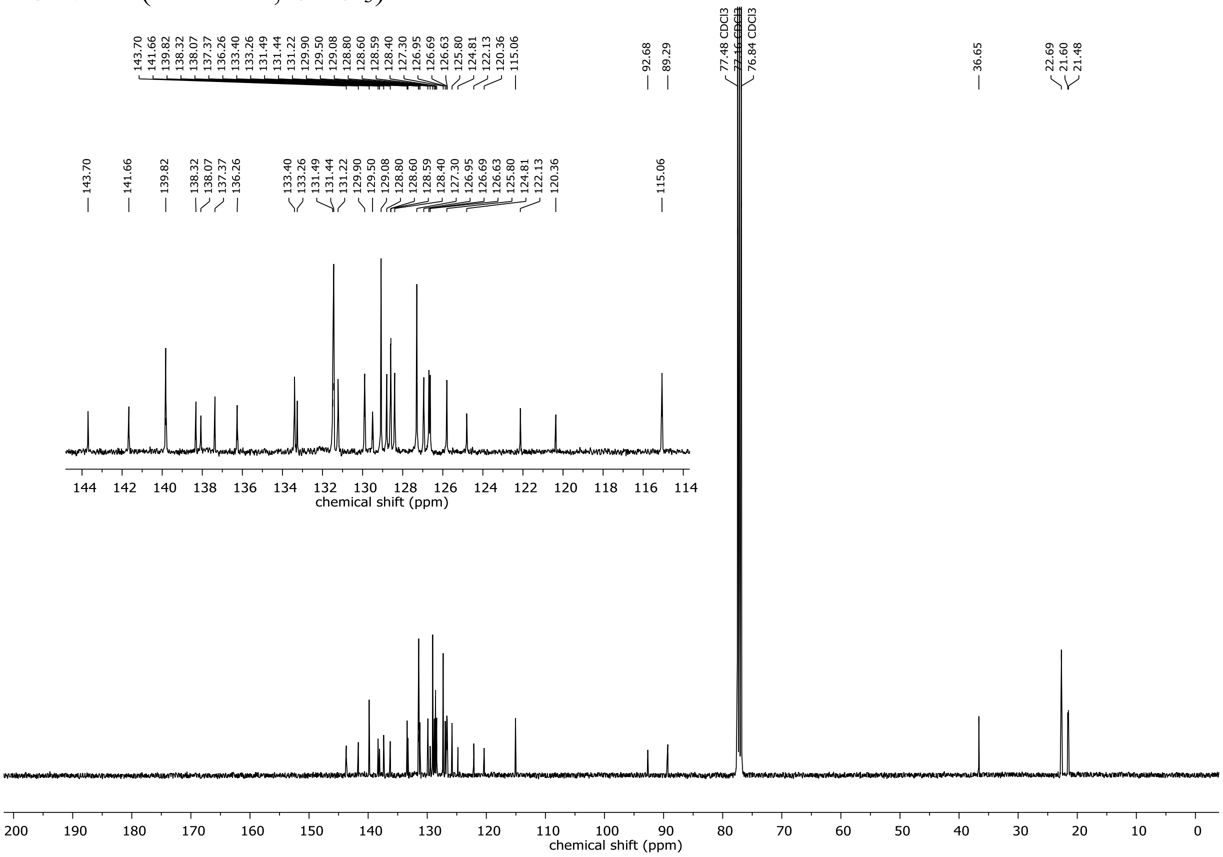

^{11}B NMR (161 MHz, $CDCl_3$)

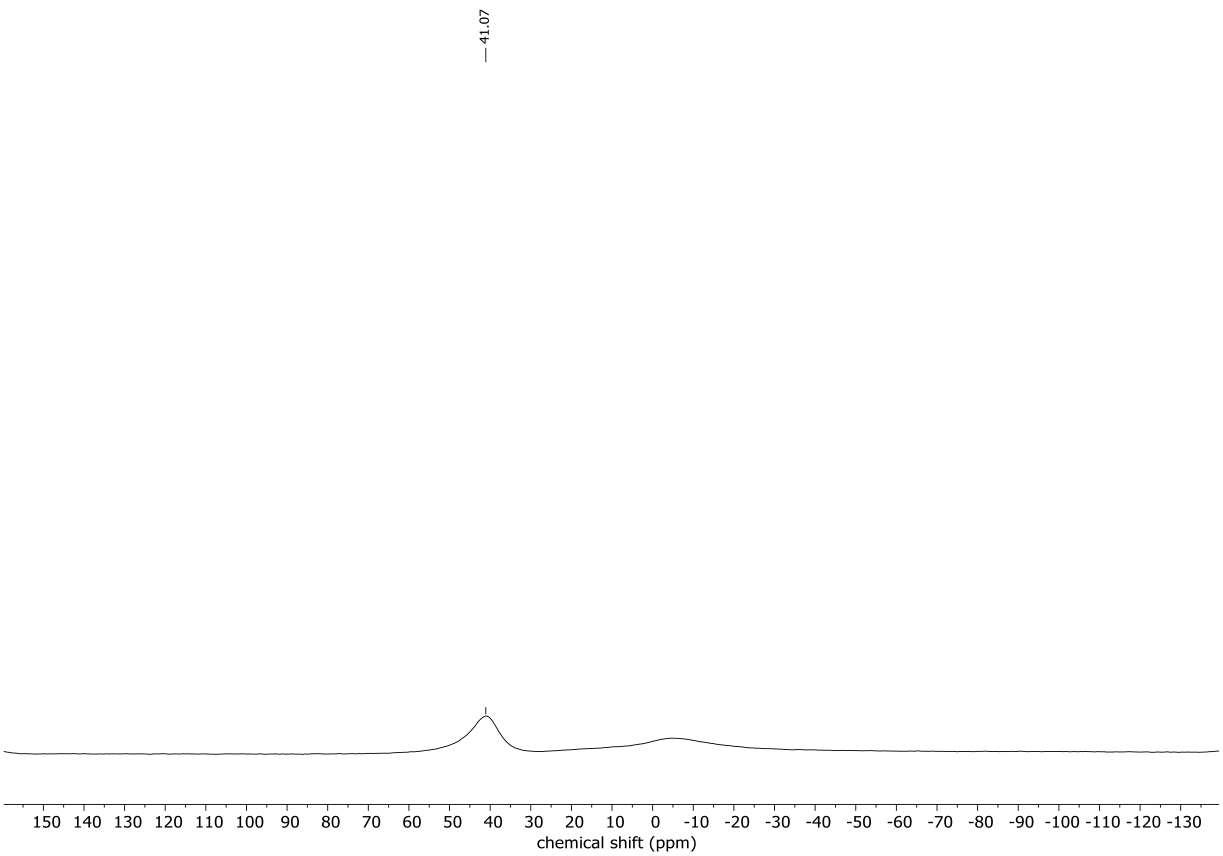

^{1}H NMR (400 MHz, $CDCl_3$) **7b**

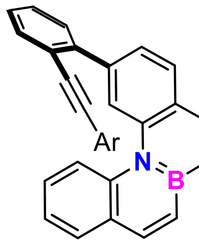

7b

Ar= *p*-(Me)C_6H_4

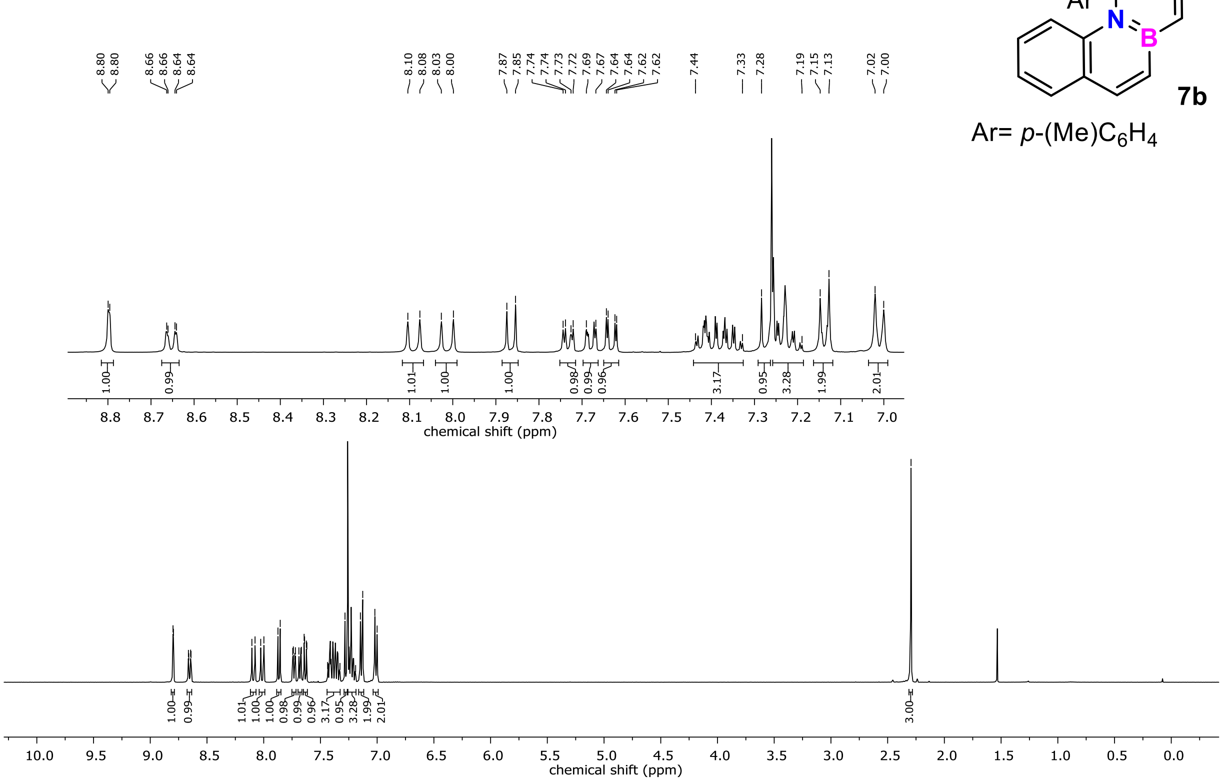

^{13}C NMR (101 MHz, $CDCl_3$)

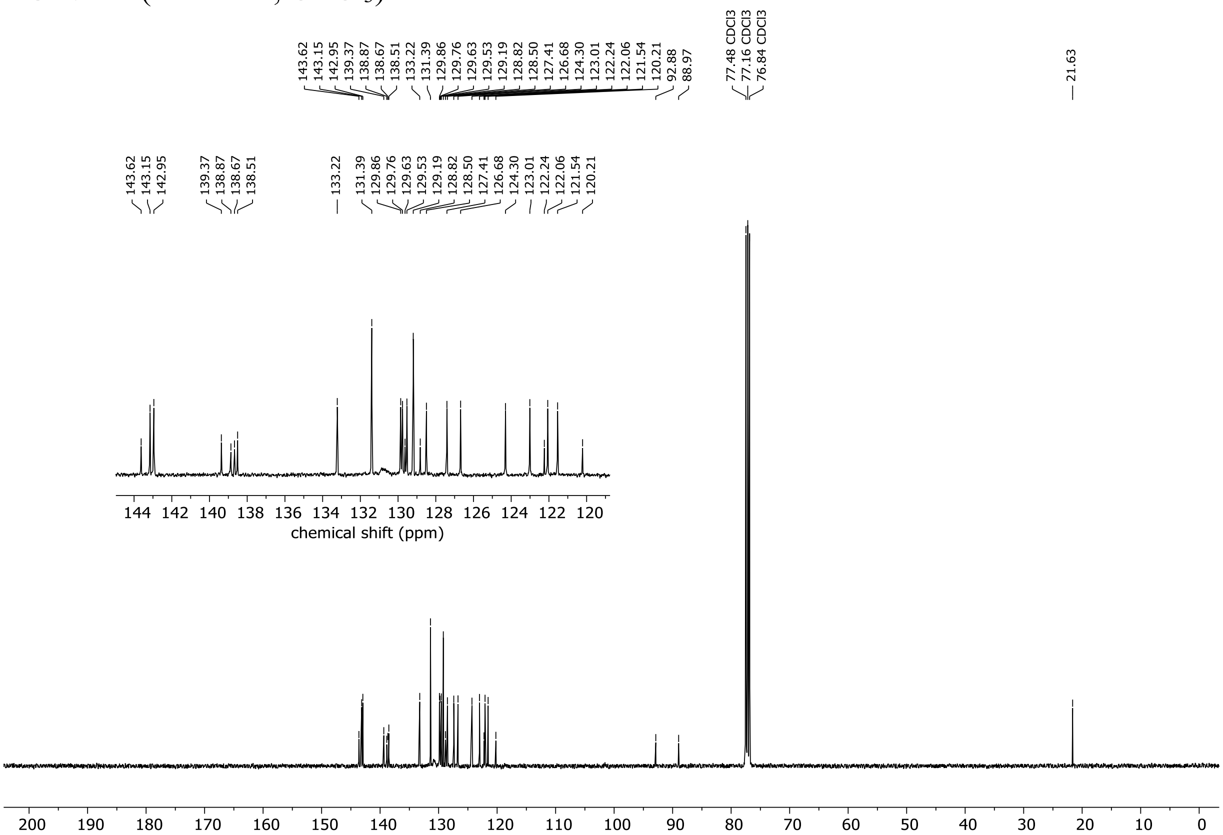

^{11}B NMR (161 MHz, $CDCl_3$)

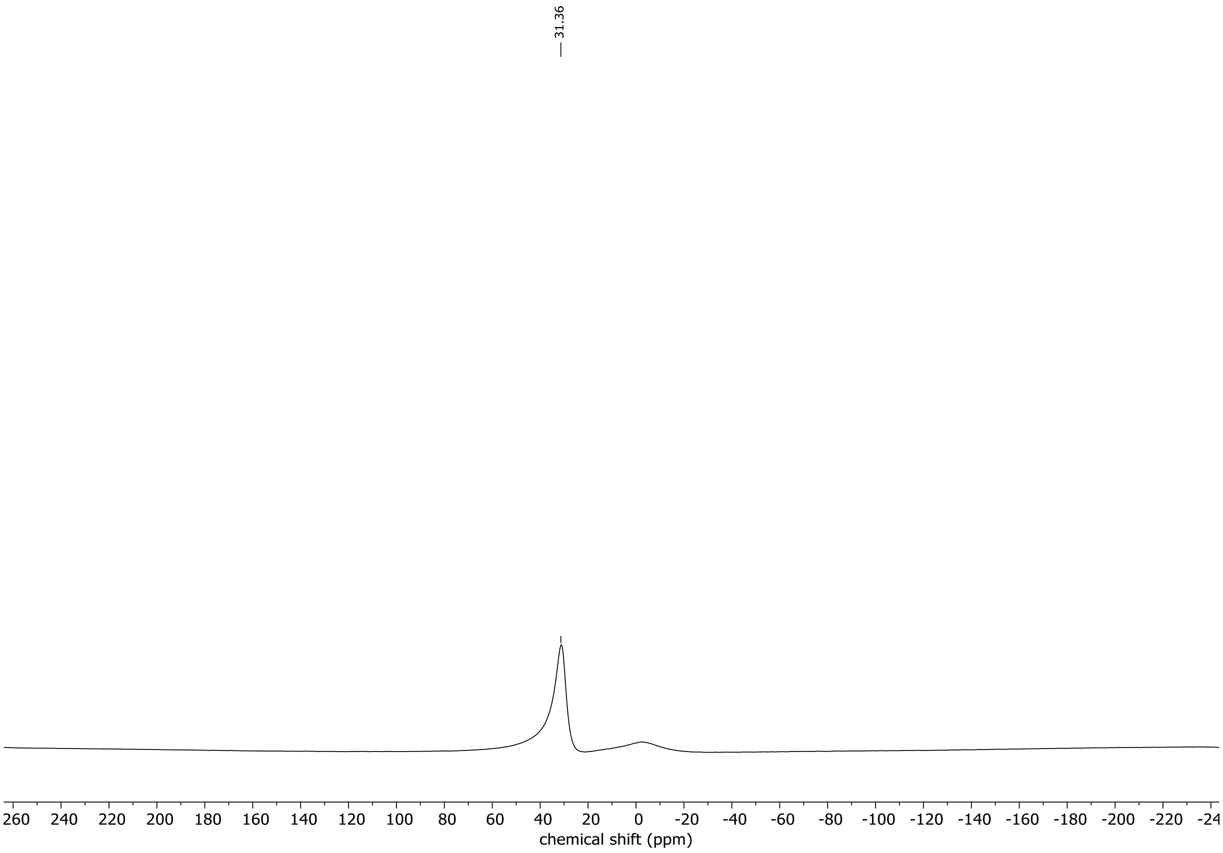

^{1}H NMR (400 MHz, $CDCl_3$) 7c

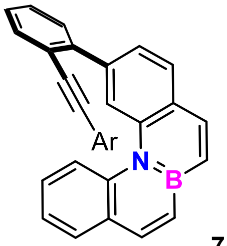

7c

Ar= *p*-(OMe)C_6H_4

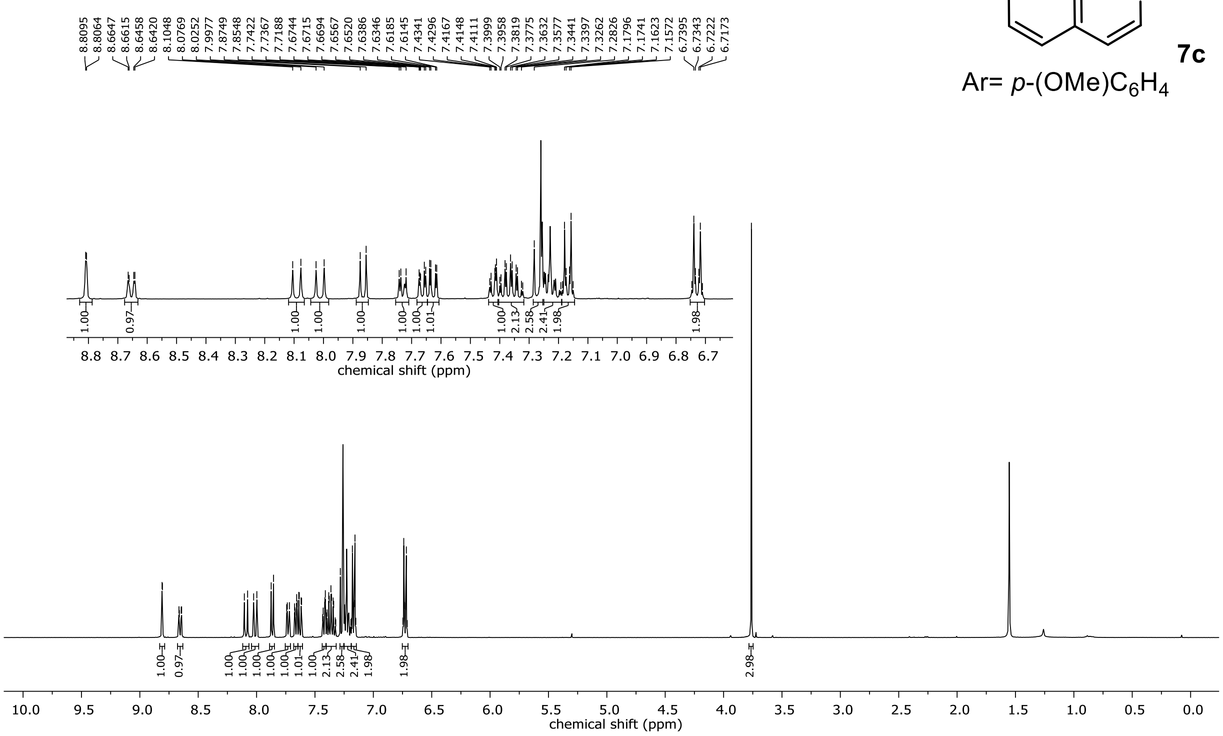

^{13}C NMR (101 MHz, $CDCl_3$)

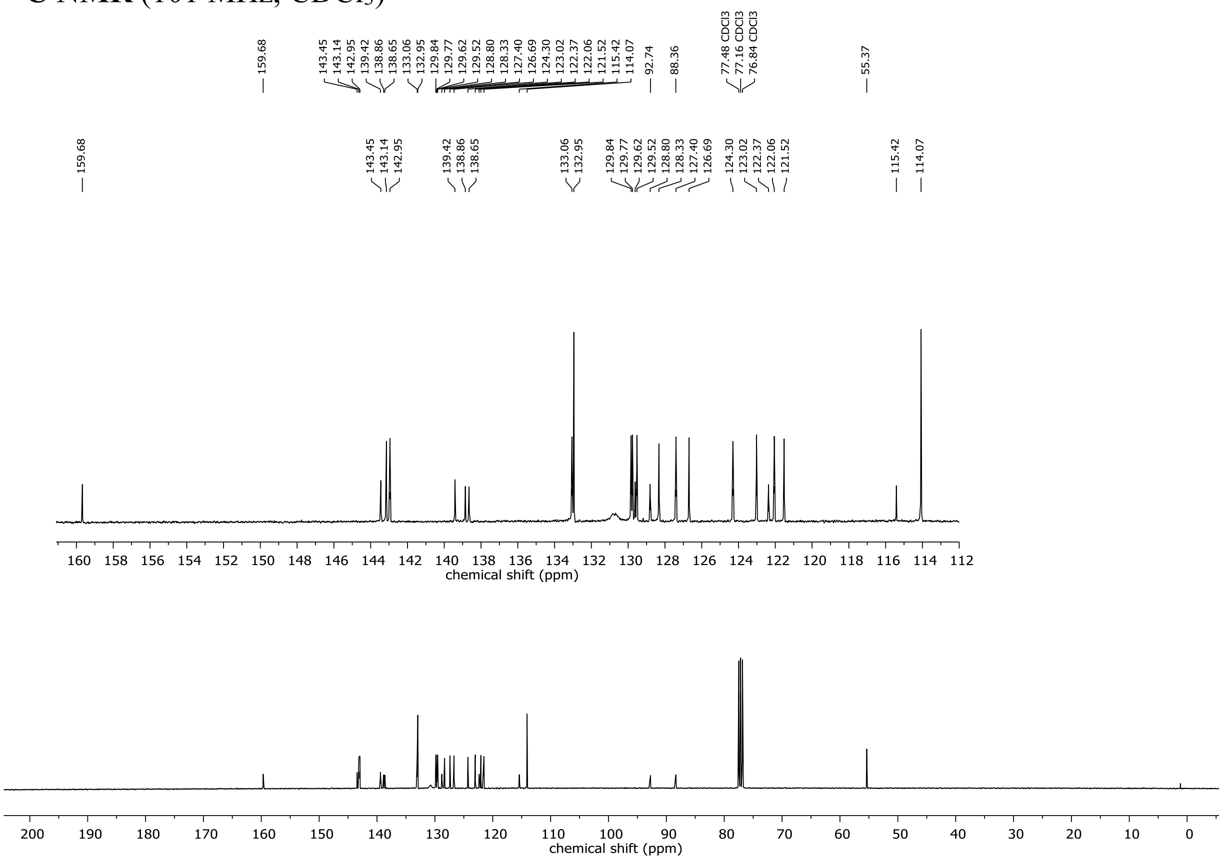

^{11}B NMR (128 MHz, $CDCl_3$)

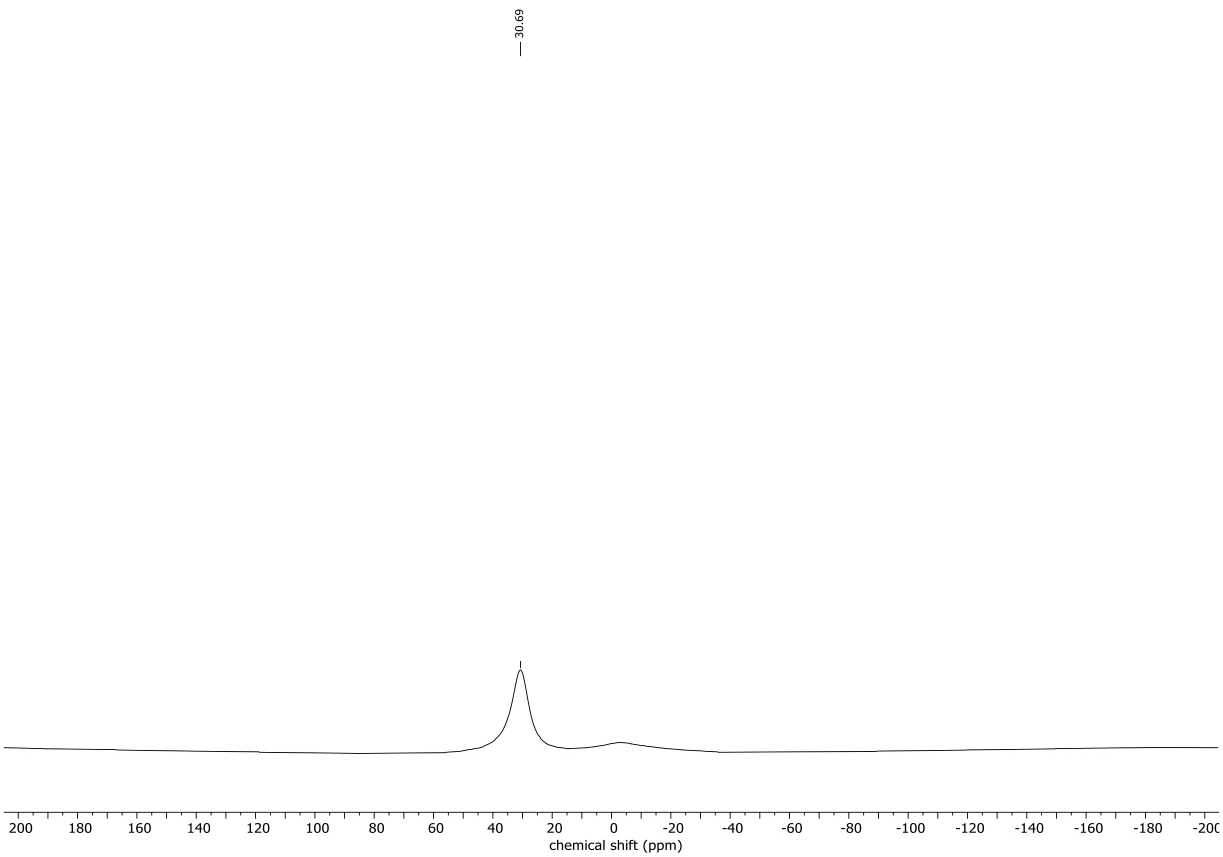

^{1}H NMR (400 MHz, $CDCl_3$) **7e**

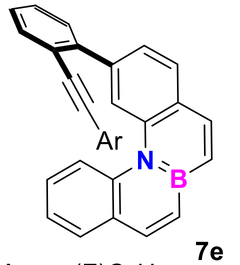

7e

Ar= p-(F)C_6H_4

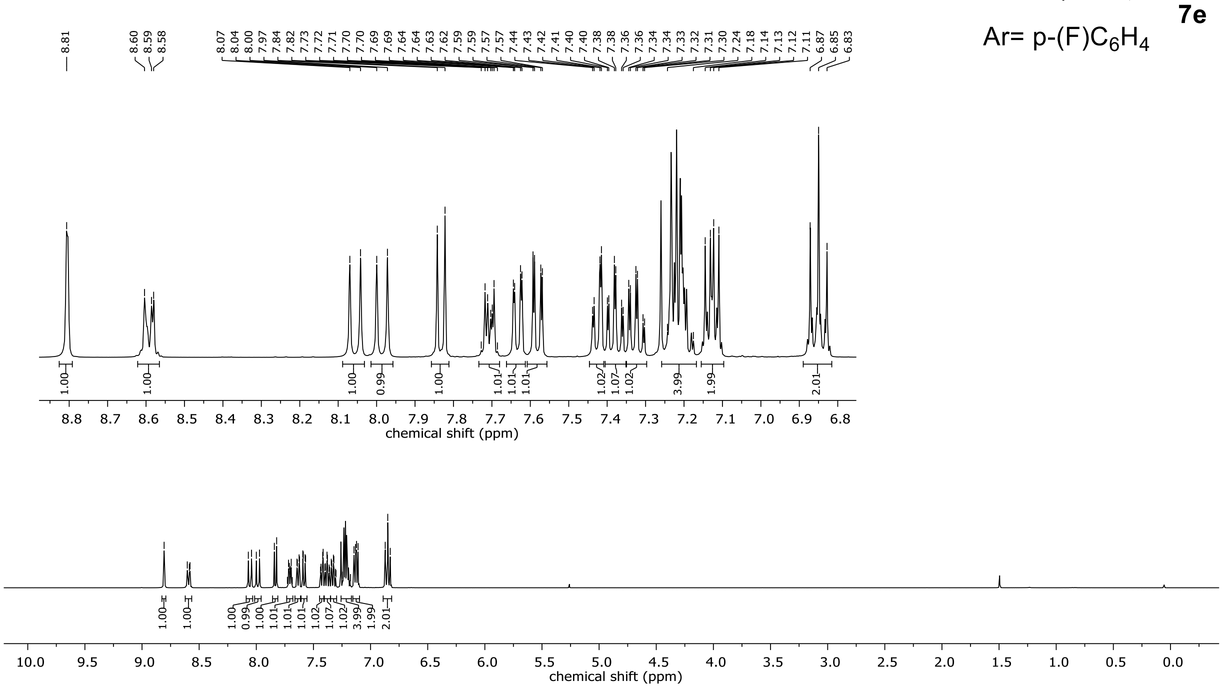

^{13}C NMR (101 MHz, $CDCl_3$)

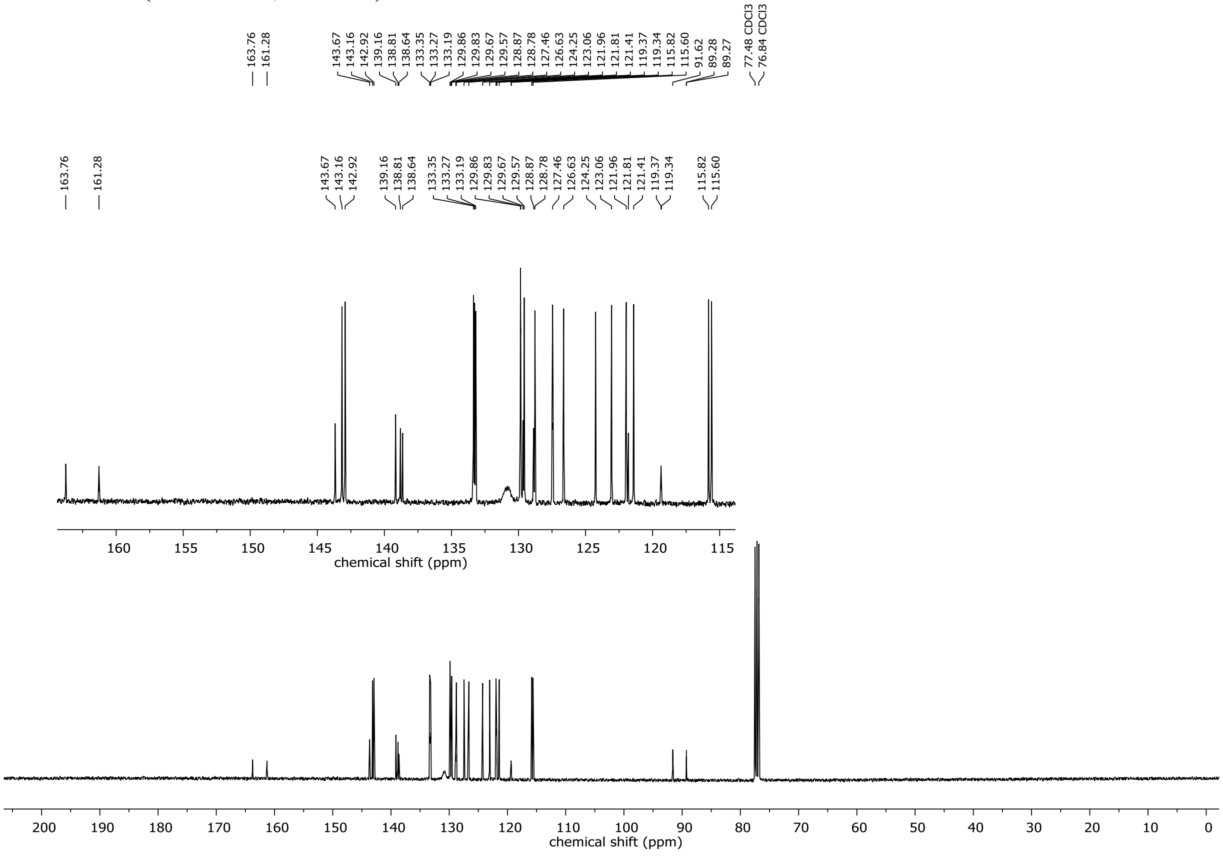

^{11}B NMR (161 MHz, $CDCl_3$)

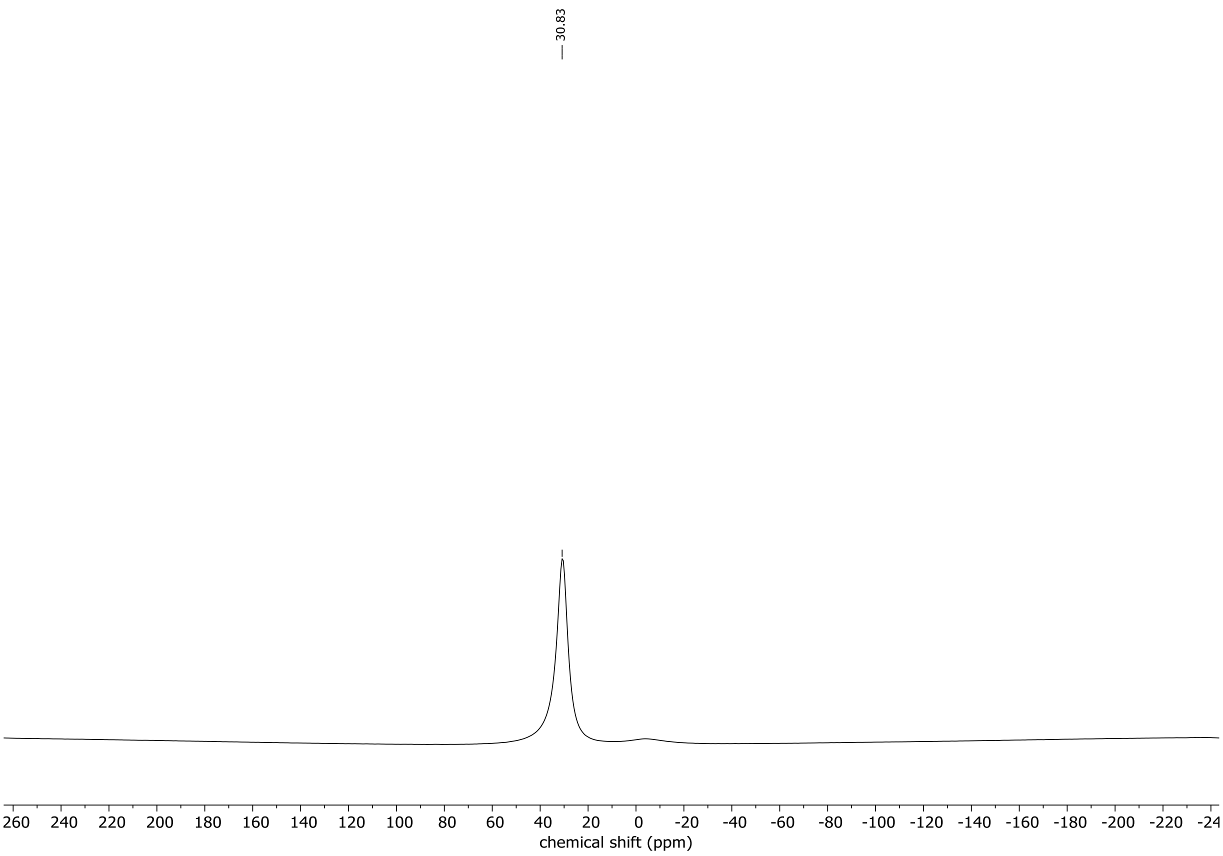

^{19}F NMR (282 MHz, $CDCl_3$)

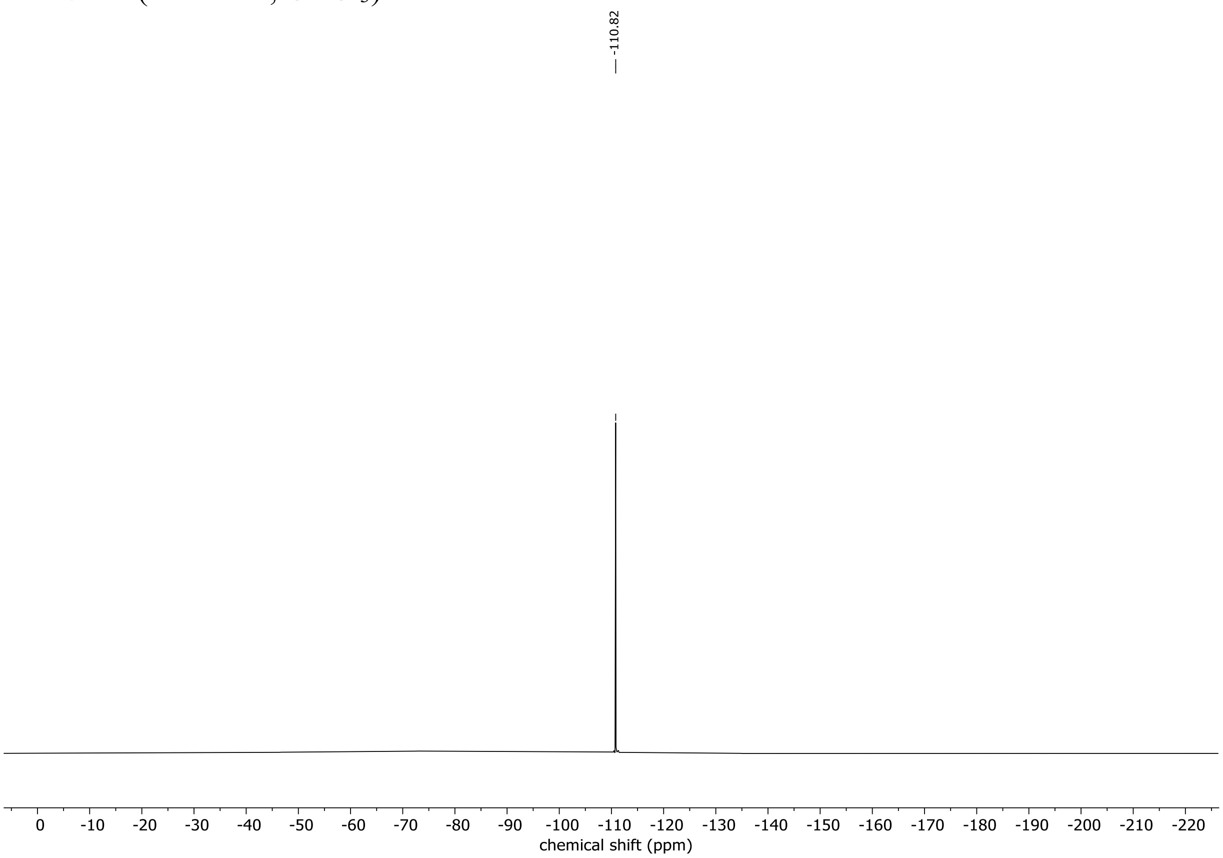

^{1}H NMR (400 MHz, $CDCl_3$) 7h

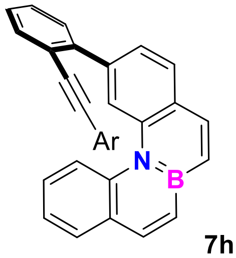

7h

Ar= p-$(CF_3)C_6H_4$

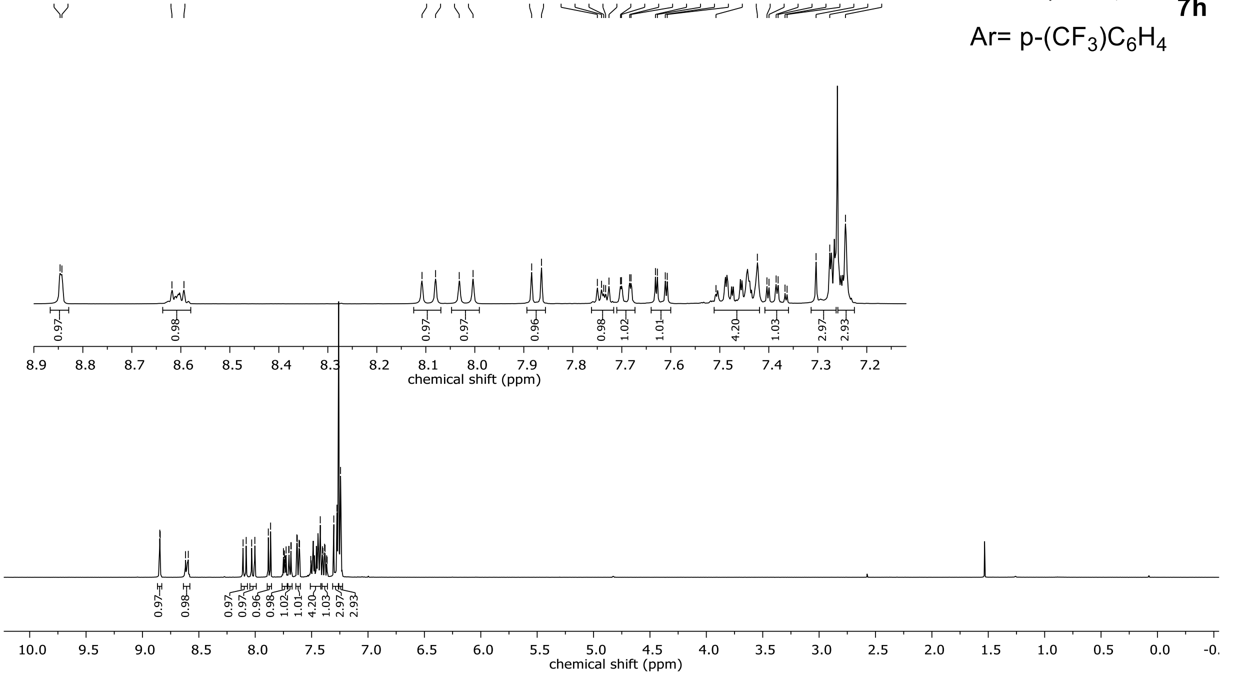

^{13}C NMR (126 MHz, $CDCl_3$)

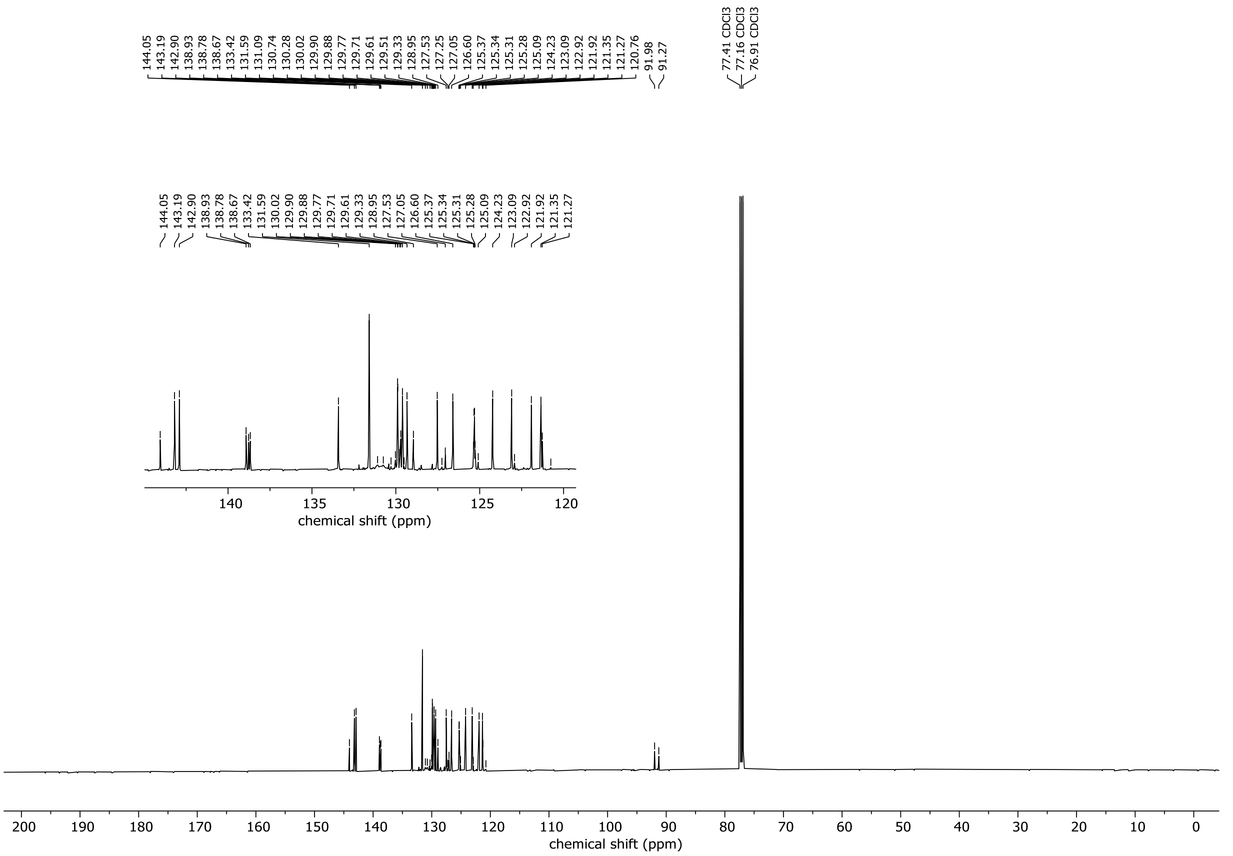

^{11}B NMR (161 MHz, $CDCl_3$)

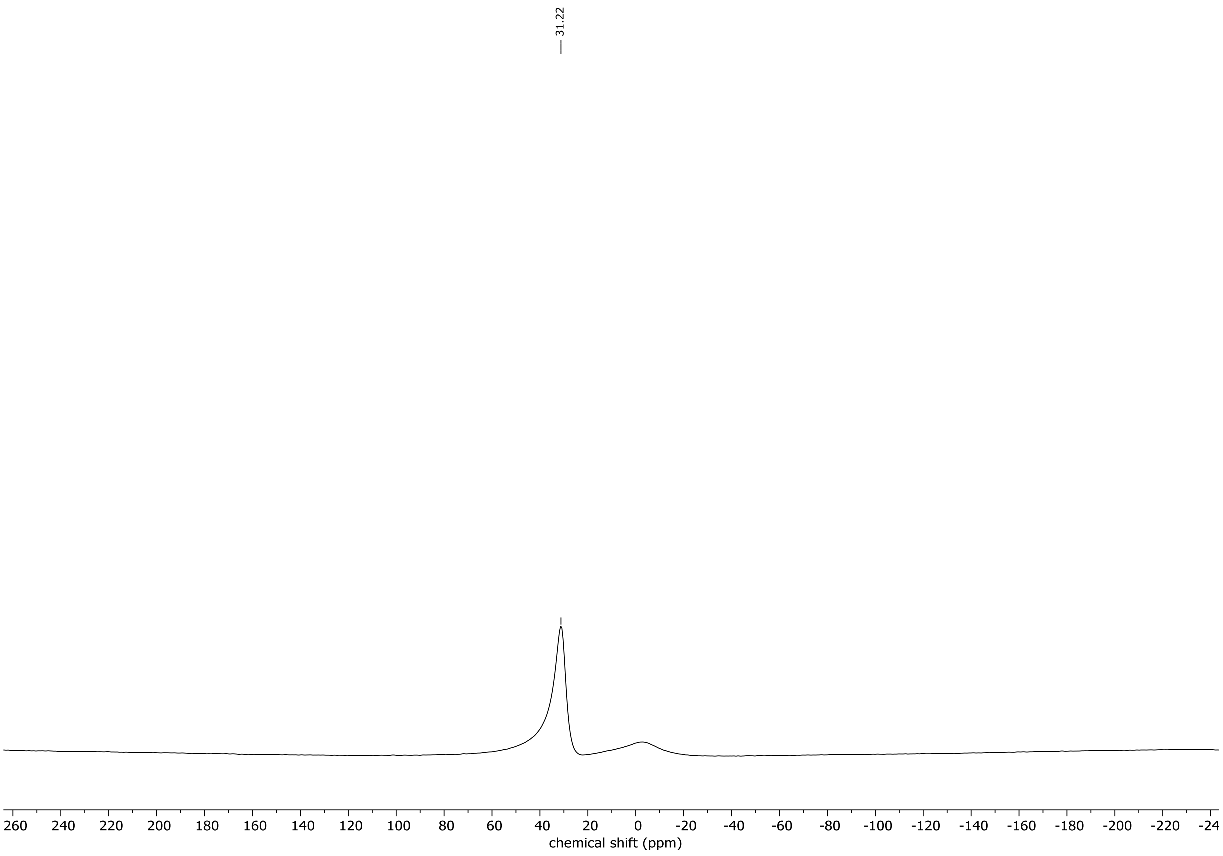

^{19}F NMR (377 MHz, $CDCl_3$)

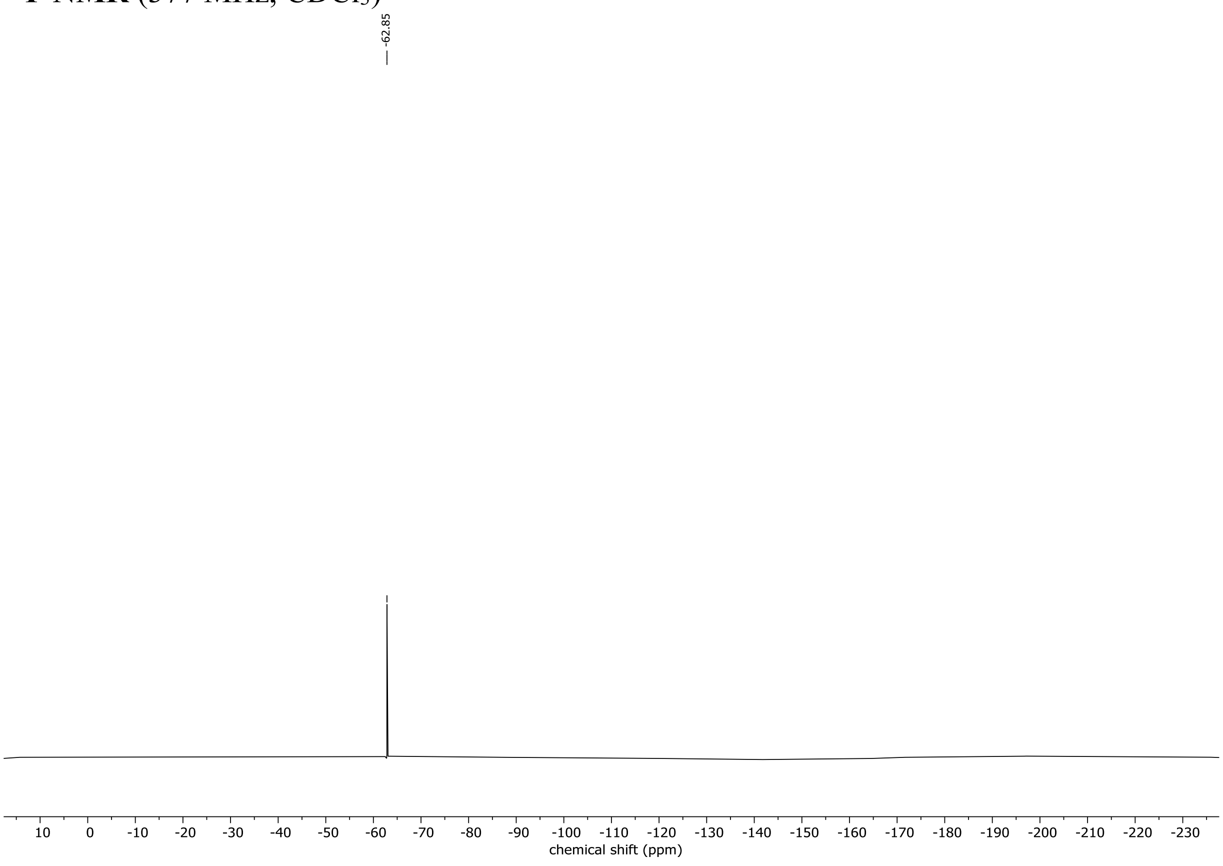

^{1}H NMR (500 MHz, CD_2Cl_2, –35 °C) **1a**

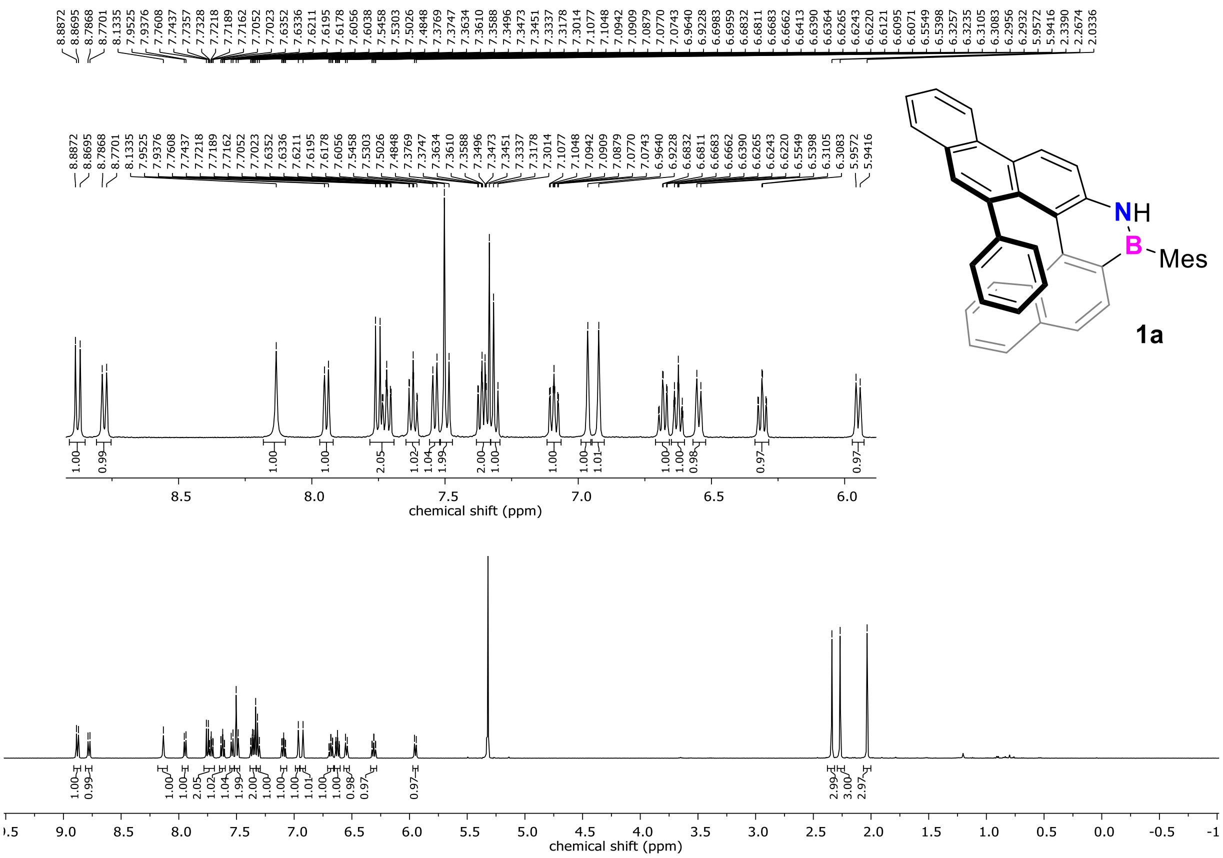

^{1}H NMR (300 MHz, CD_2Cl_2; rt)

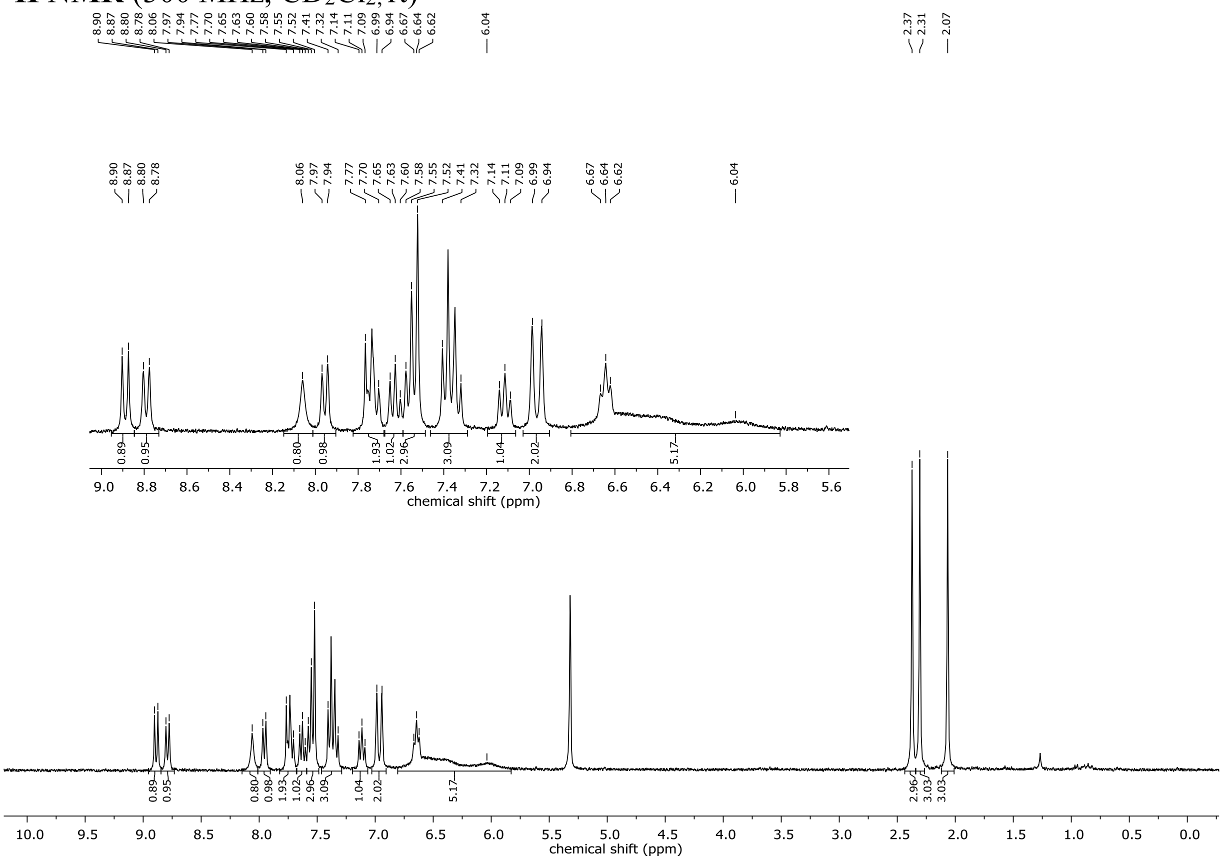

^{13}C NMR (126 MHz, CD_2Cl_2, –35 °C)

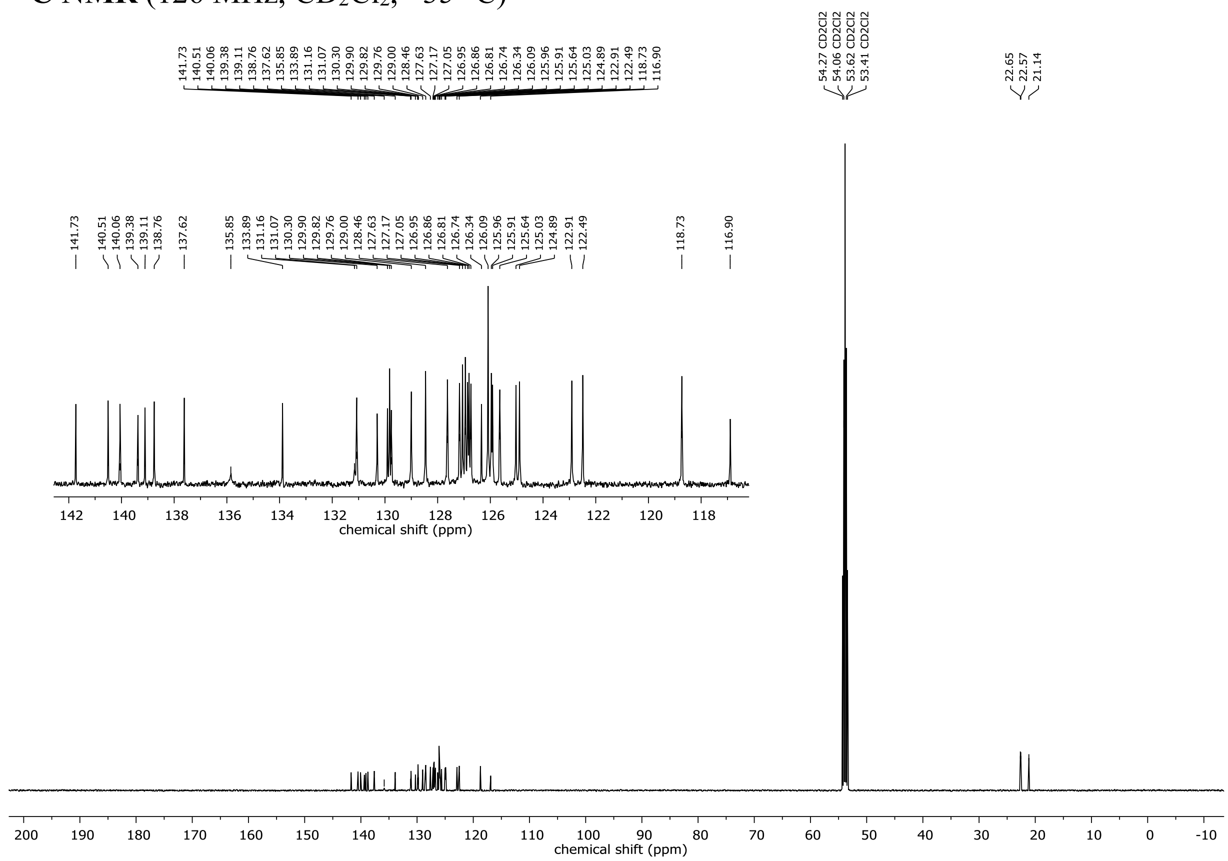

^{11}B NMR (161 MHz, CD_2Cl_2)

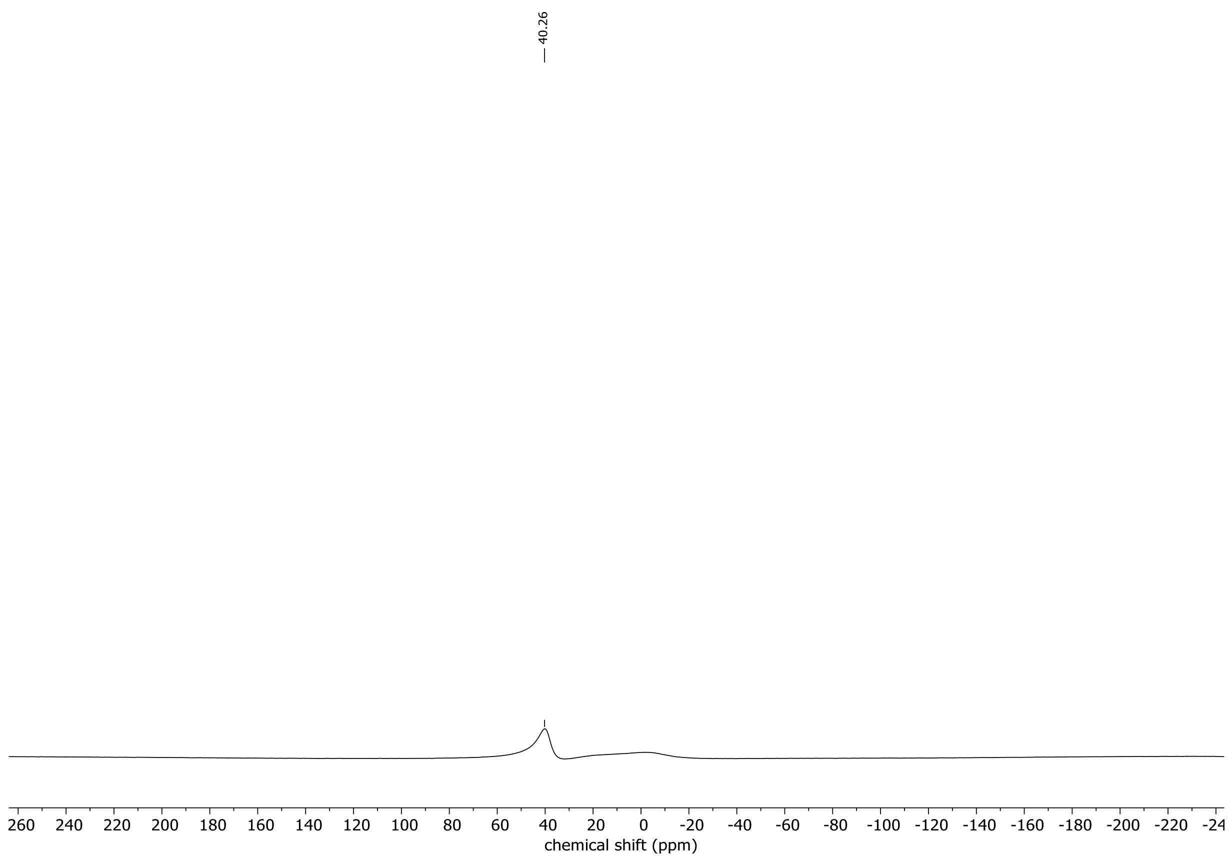

^{1}H NMR (600 MHz, CD_2Cl_2, -35ºC) 1b

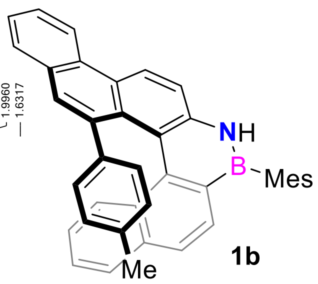

1b

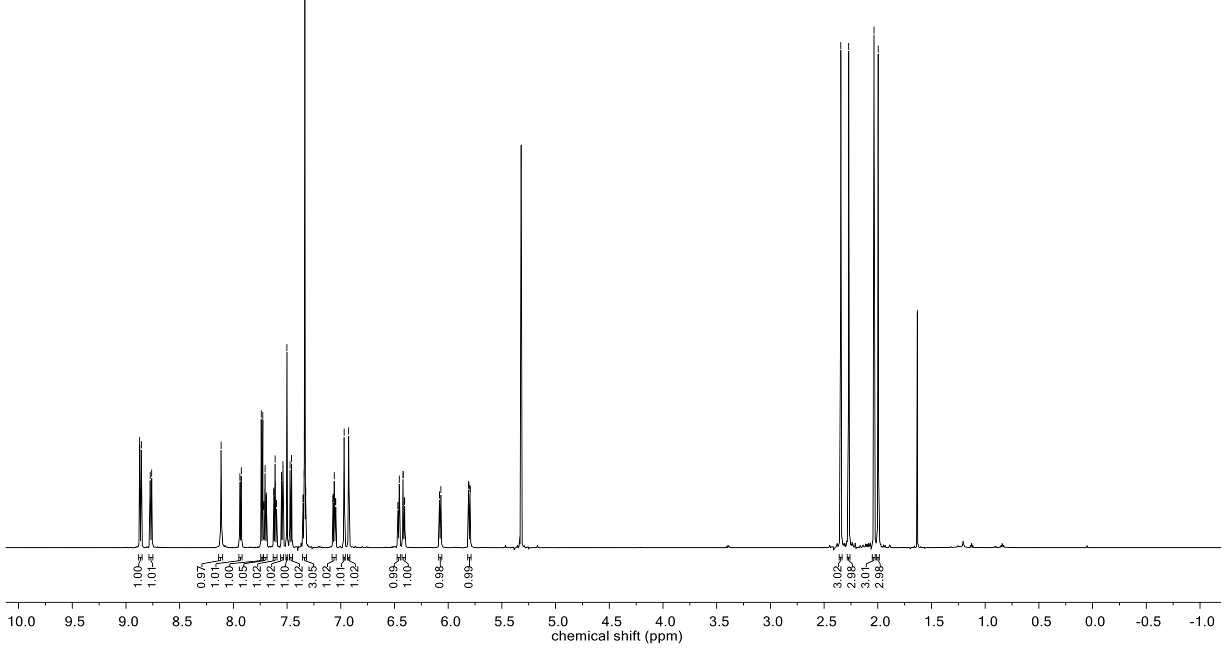

^{1}H NMR (300 MHz, CD_2Cl_2; rt)

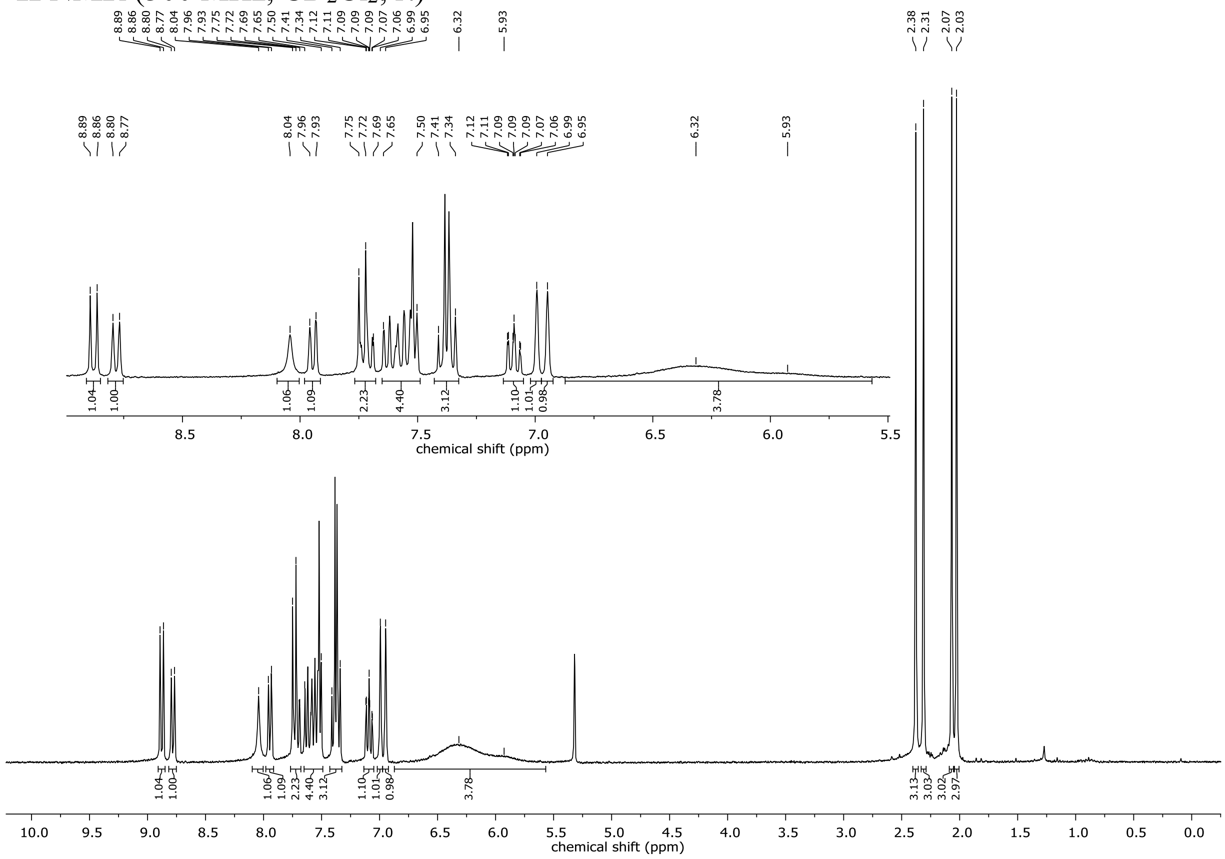

^{13}C NMR (151 MHz, CD_2Cl_2, -35ºC)

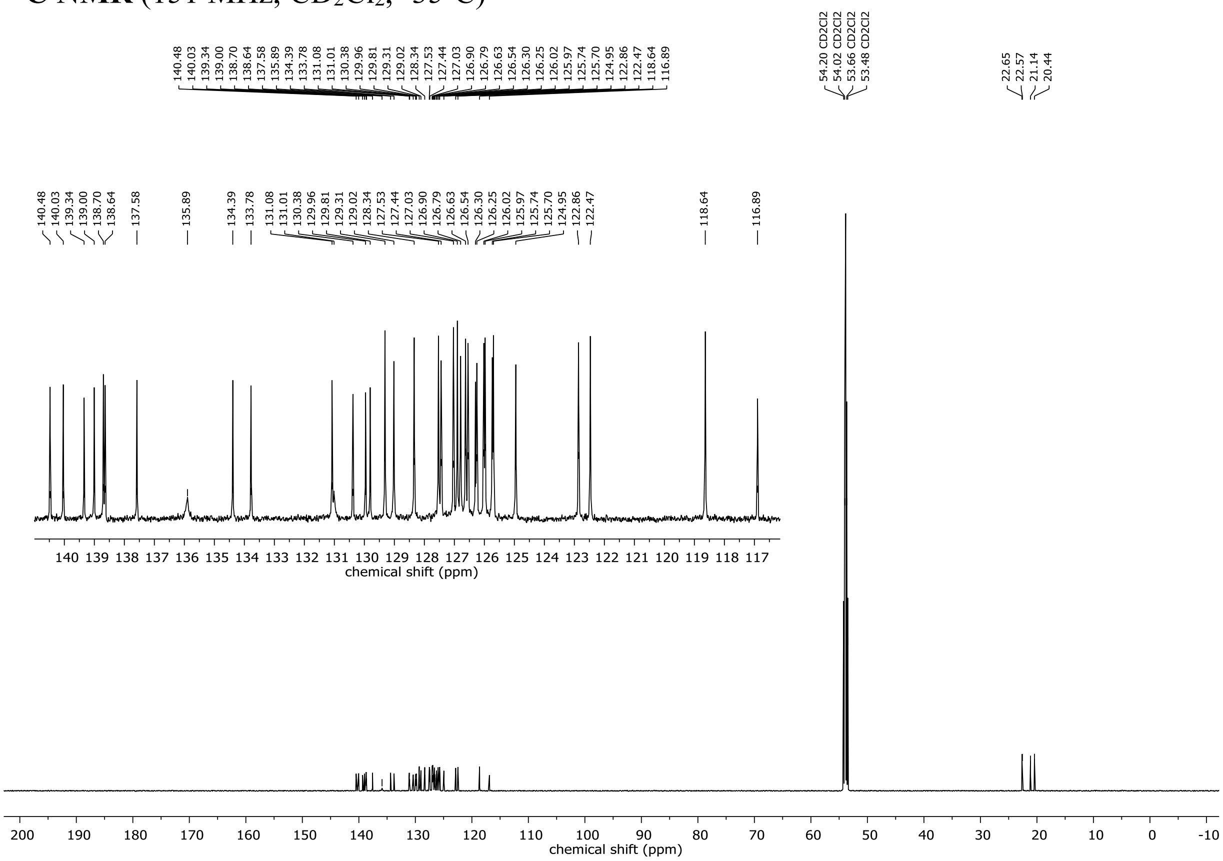

^{11}B NMR (161 MHz, CD_2Cl_2)

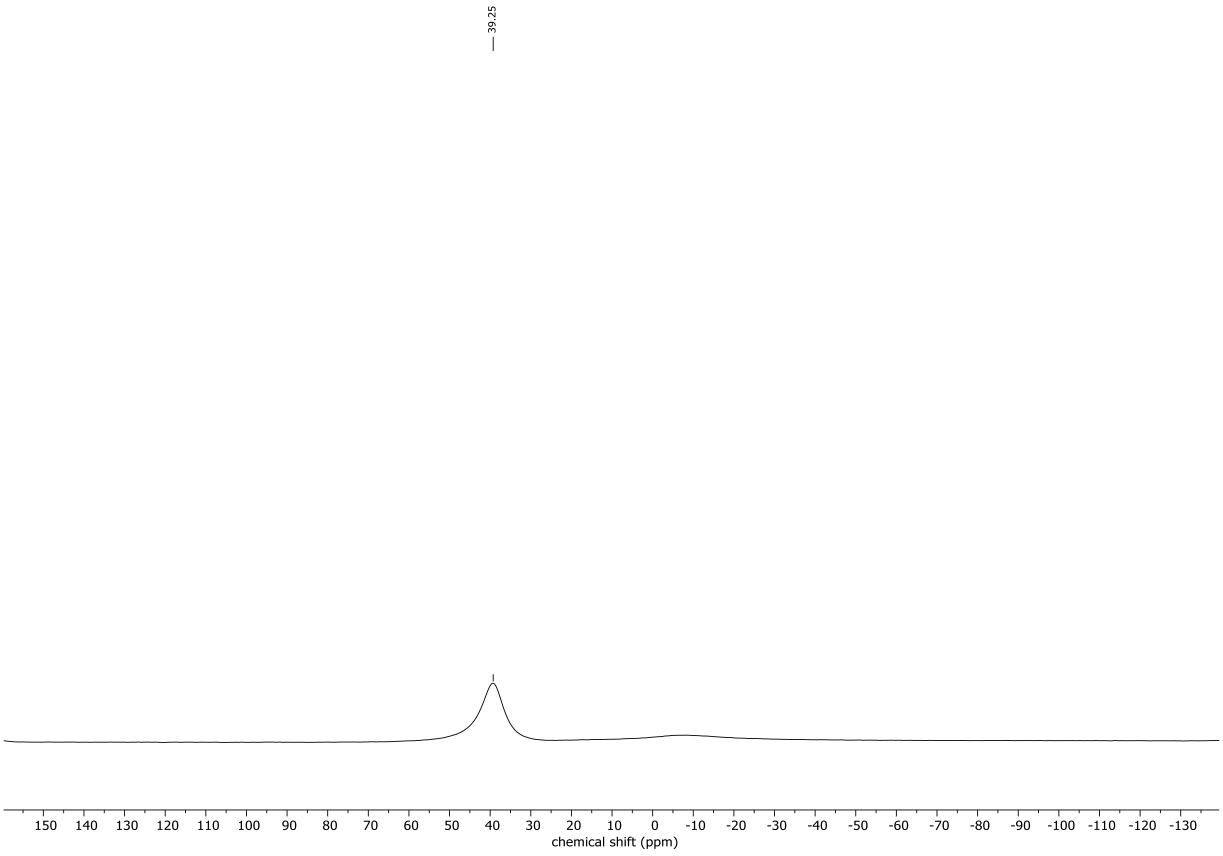

^{1}H NMR (600 MHz, CD_2Cl_2, -35ºC) **1c**

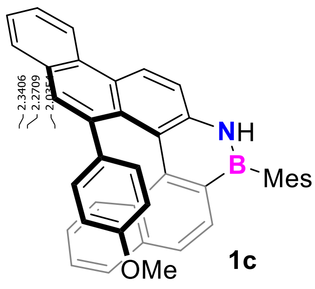

1c

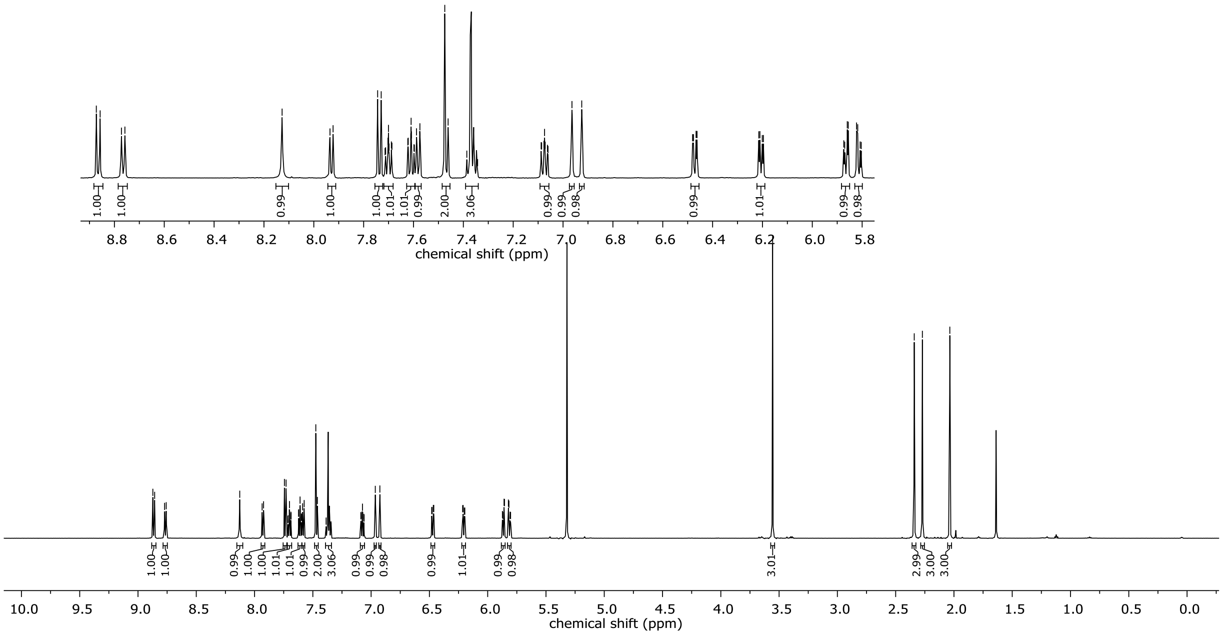

^{1}H NMR (300 MHz, CD_2Cl_2; rt)

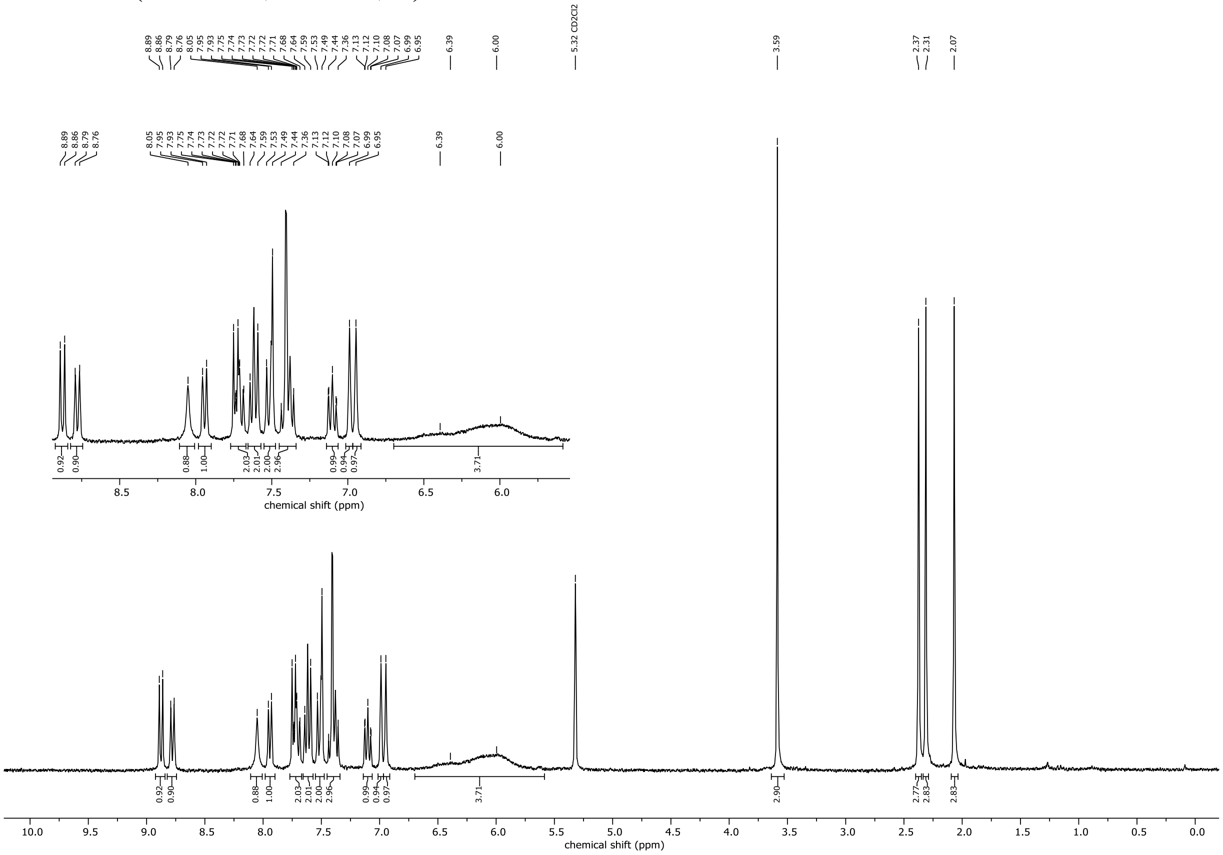

^{13}C NMR (151 MHz, CD_2Cl_2, -35ºC)

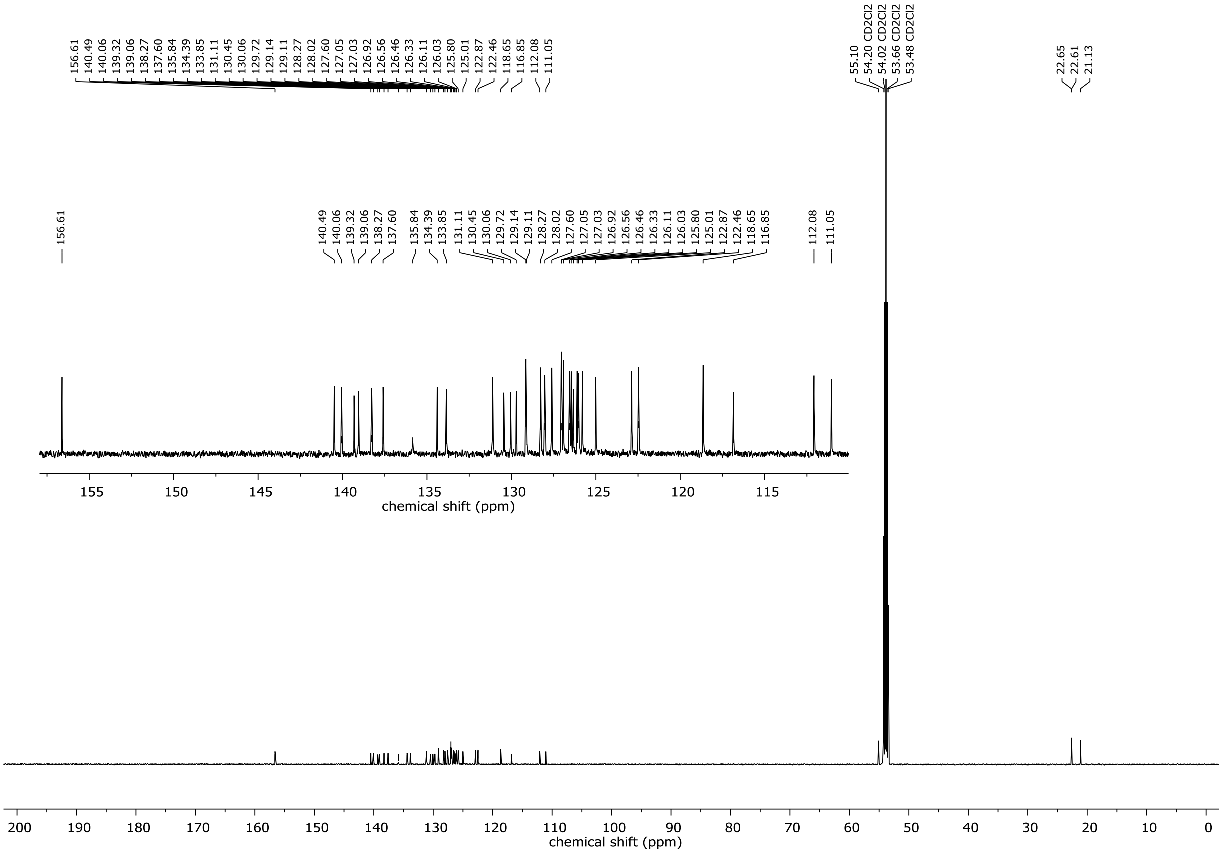

^{11}B NMR (161 MHz, CD_2Cl_2)

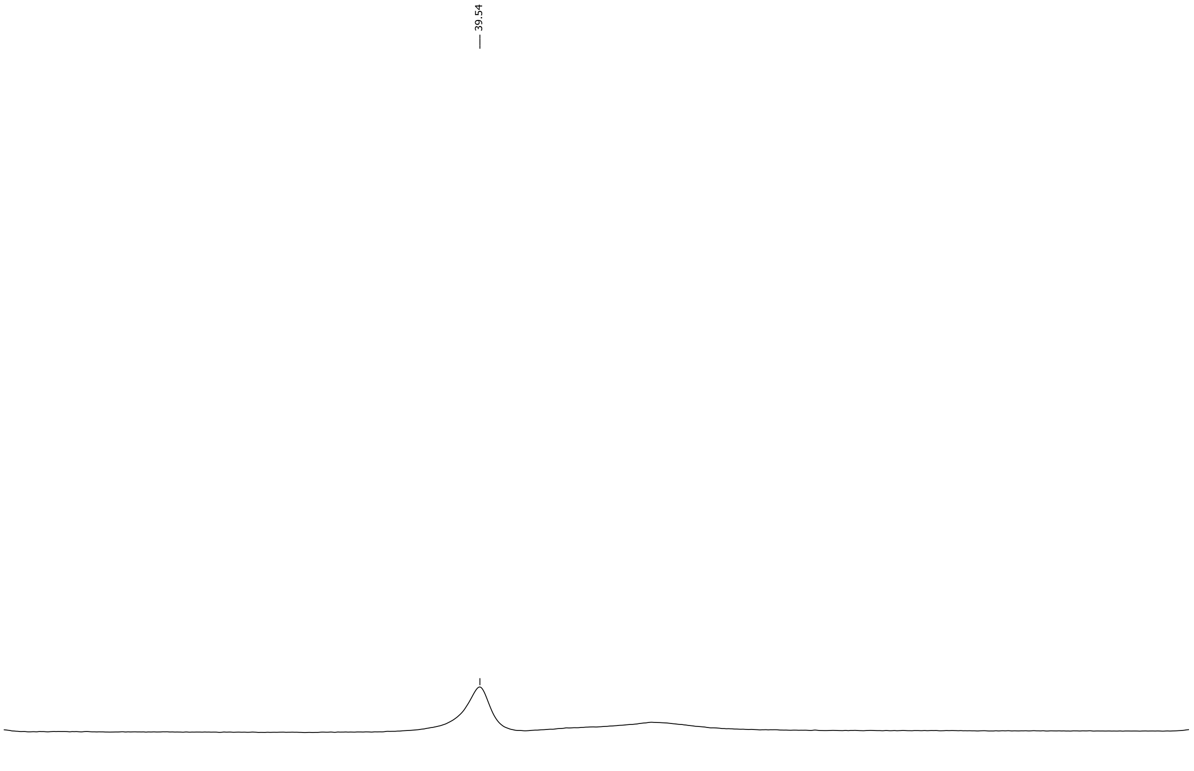

^{1}H NMR (500 MHz, THF, -35ºC) 1d

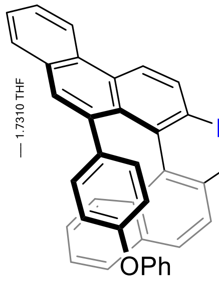

1d

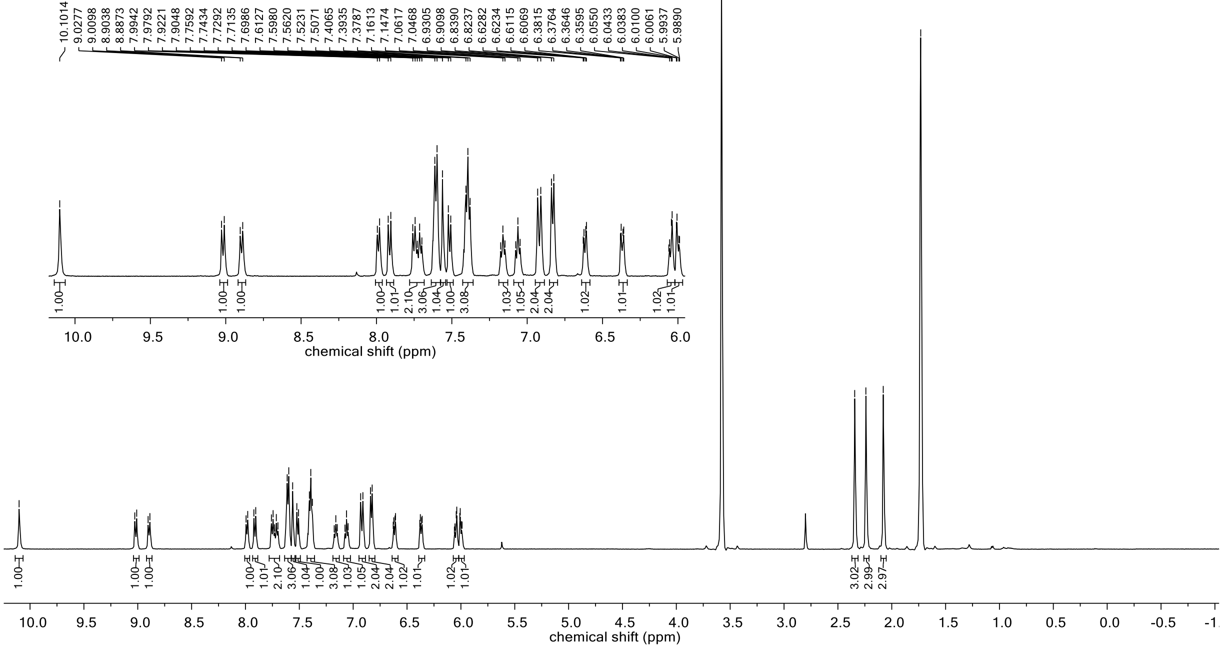

^{1}H NMR (400 MHz, THF; rt)

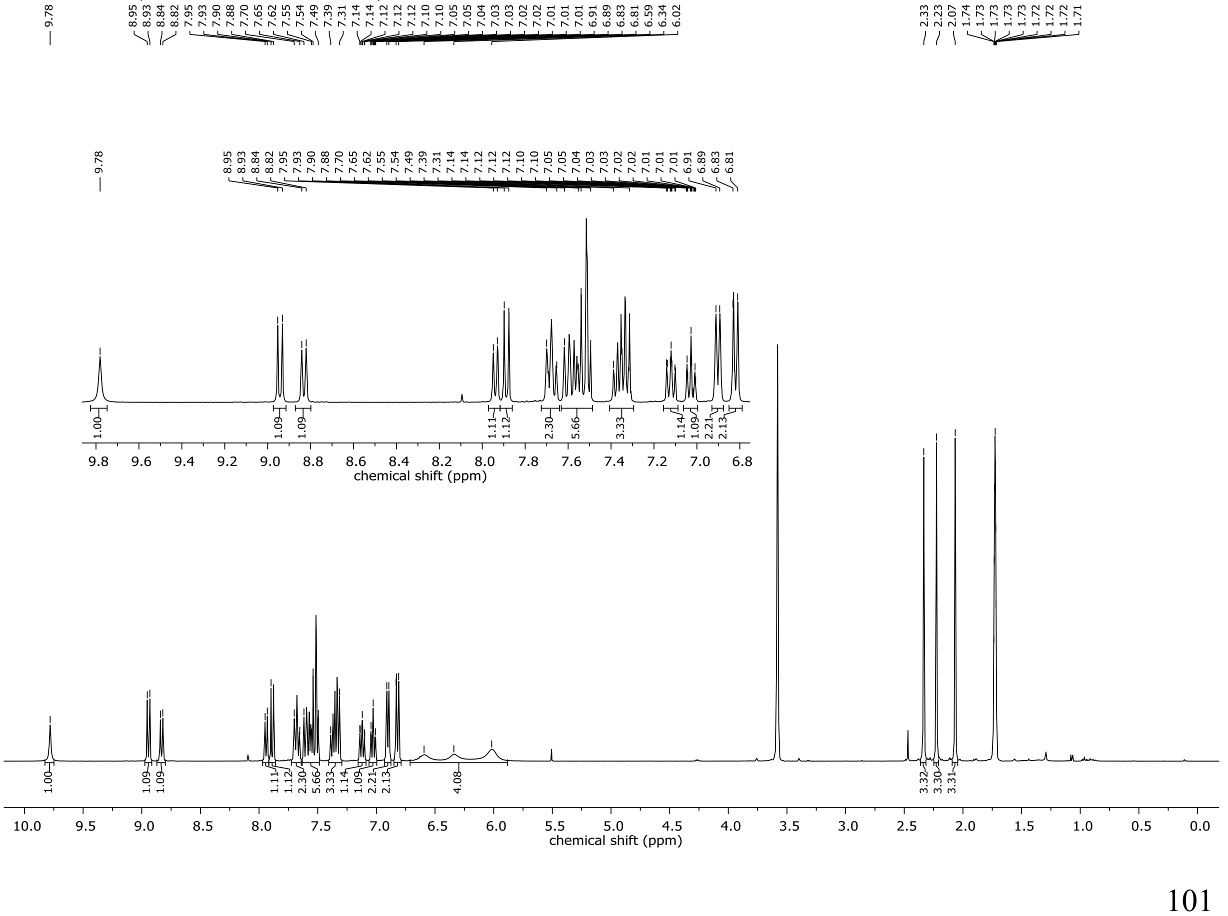

^{13}C NMR (126 MHz, THF, -35ºC)

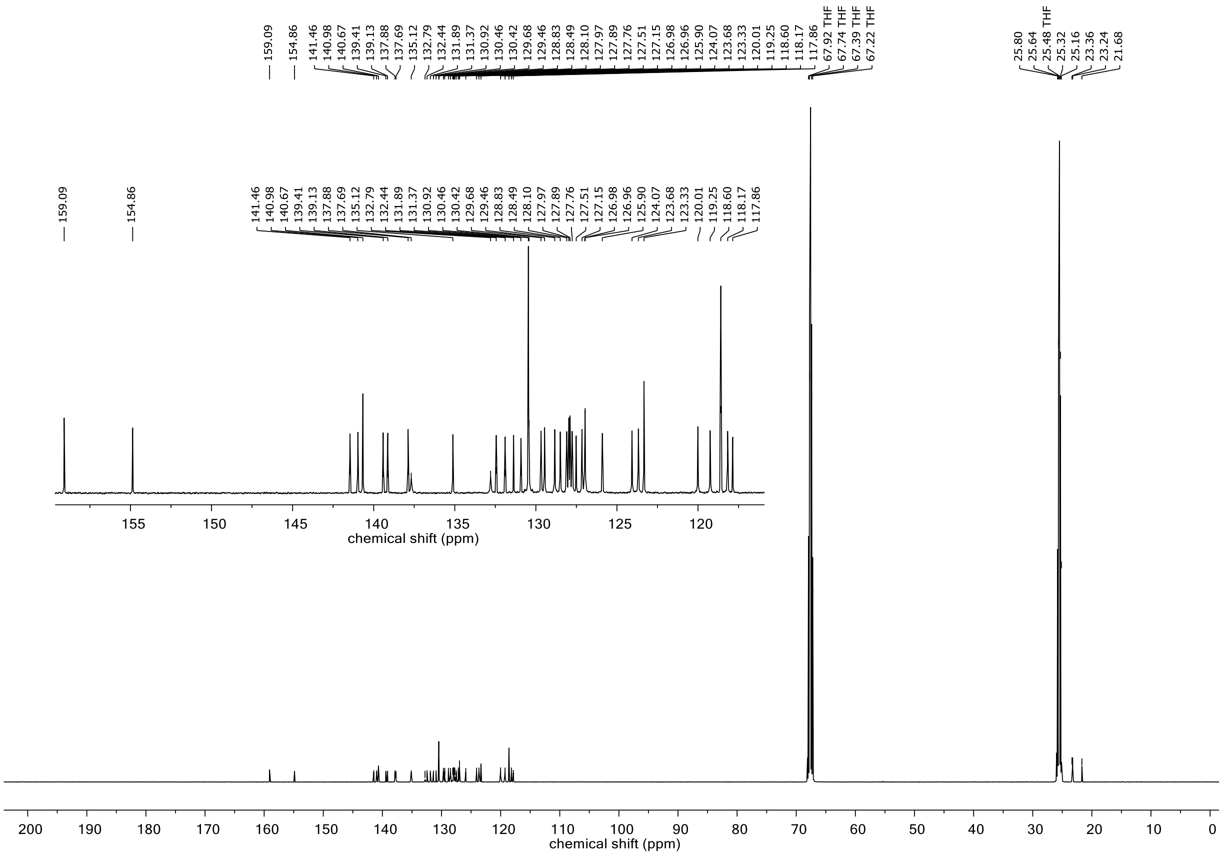

^{11}B NMR (161 MHz, THF)

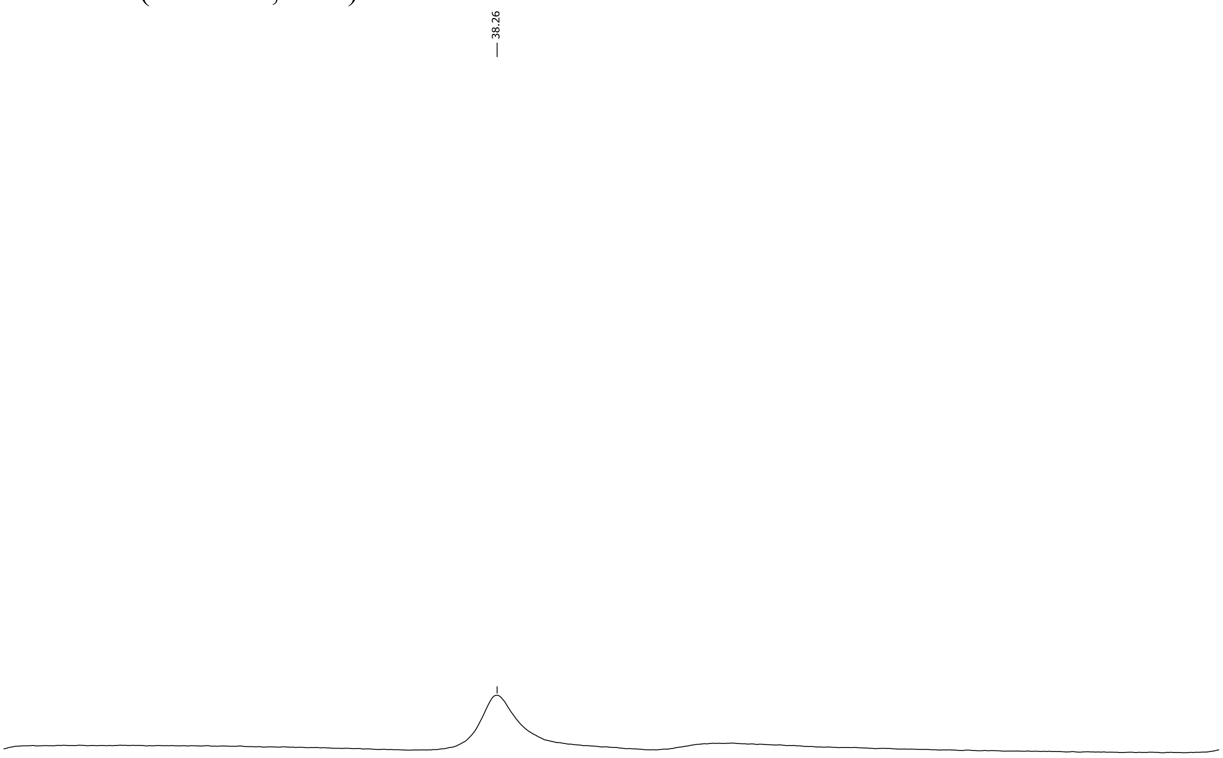

^{1}H NMR (600 MHz, CD_2Cl_2, -35°C) **1e**

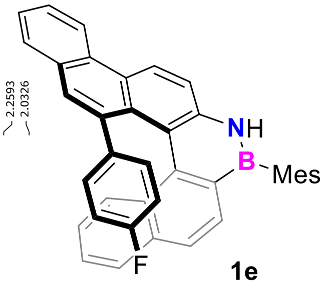

1e

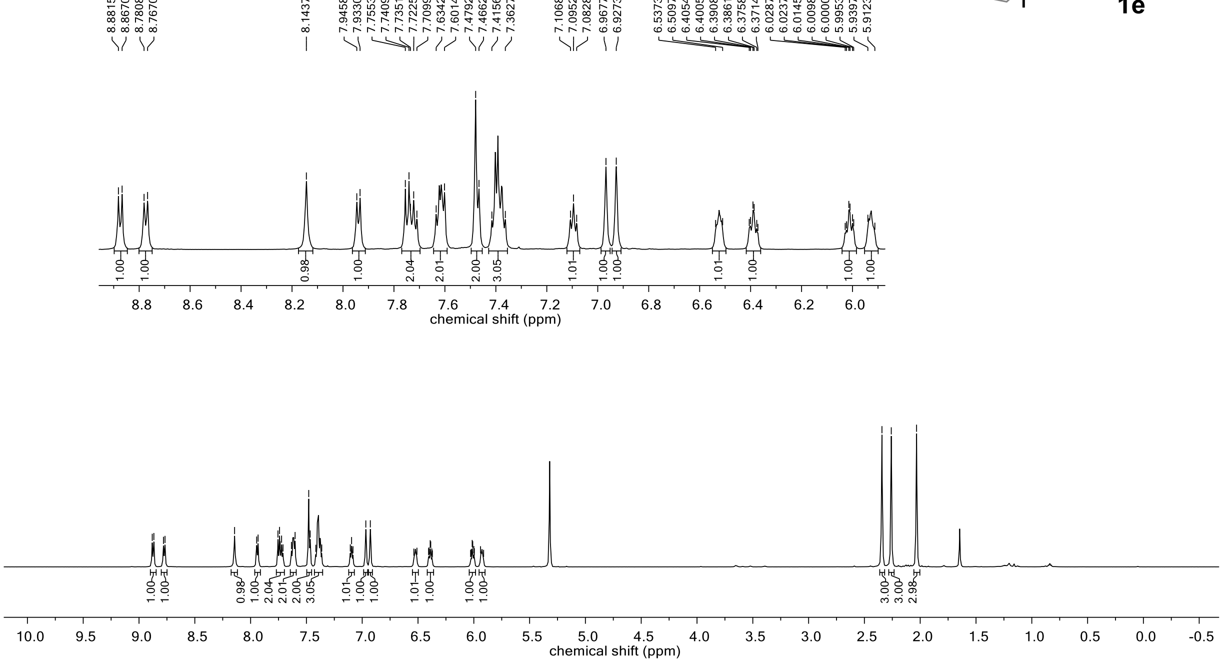

^{1}H NMR (300 MHz, CD_2Cl_2; rt)

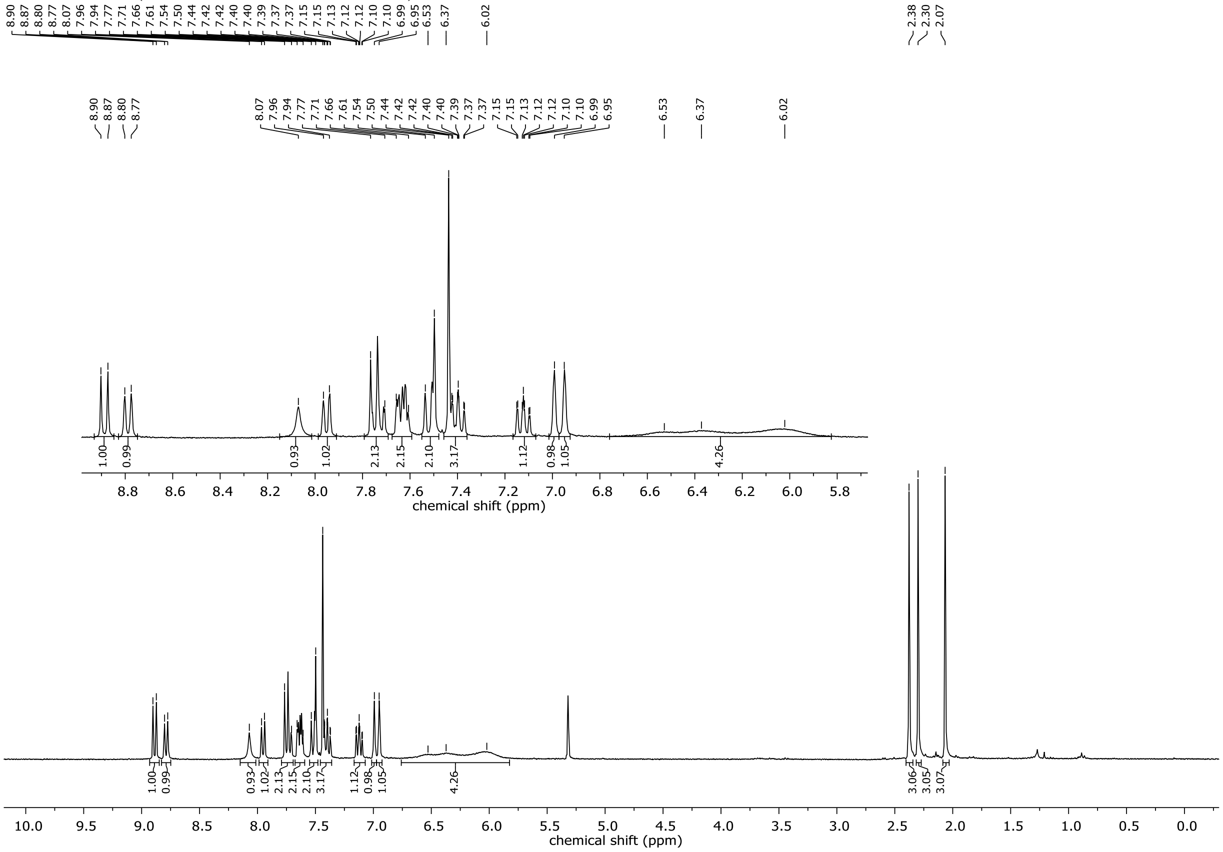

^{13}C NMR (151 MHz, CD_2Cl_2, -35ºC)

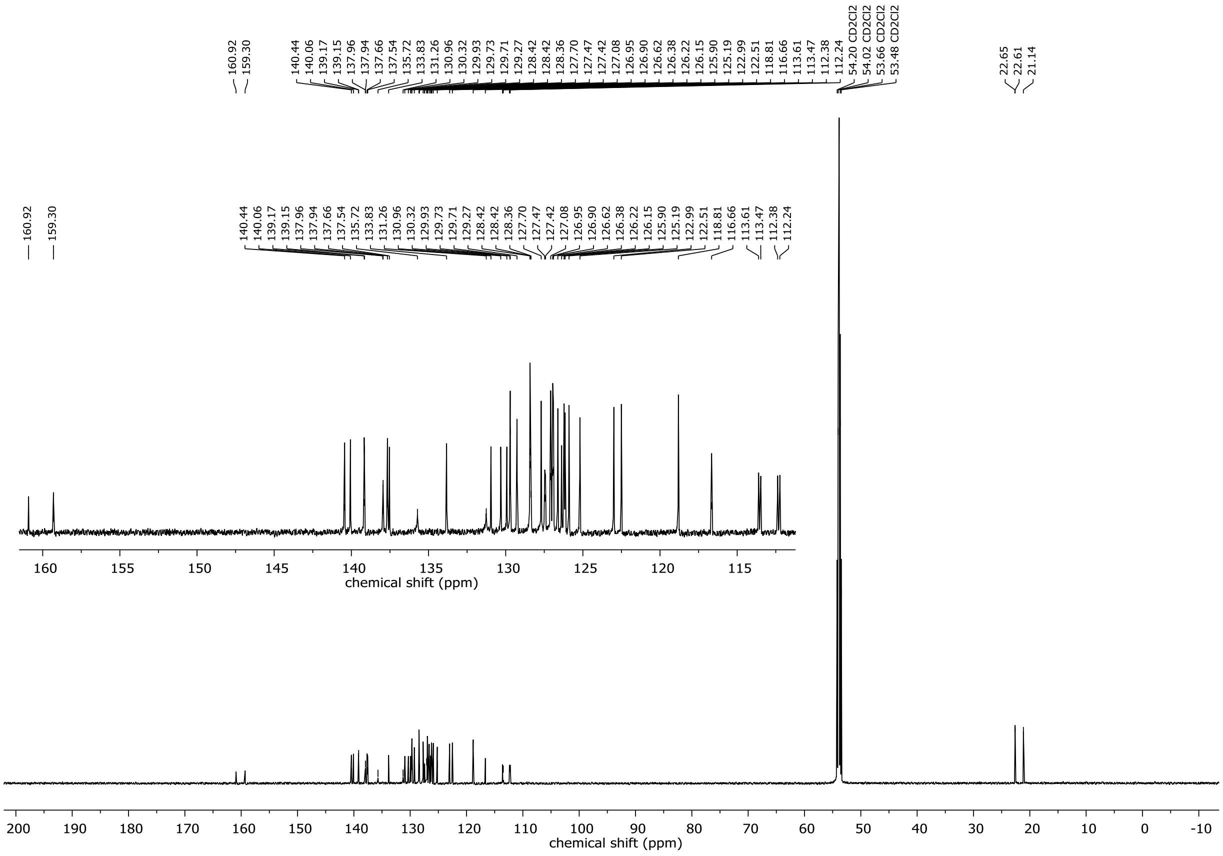

^{11}B NMR (161 MHz, CD_2Cl_2)

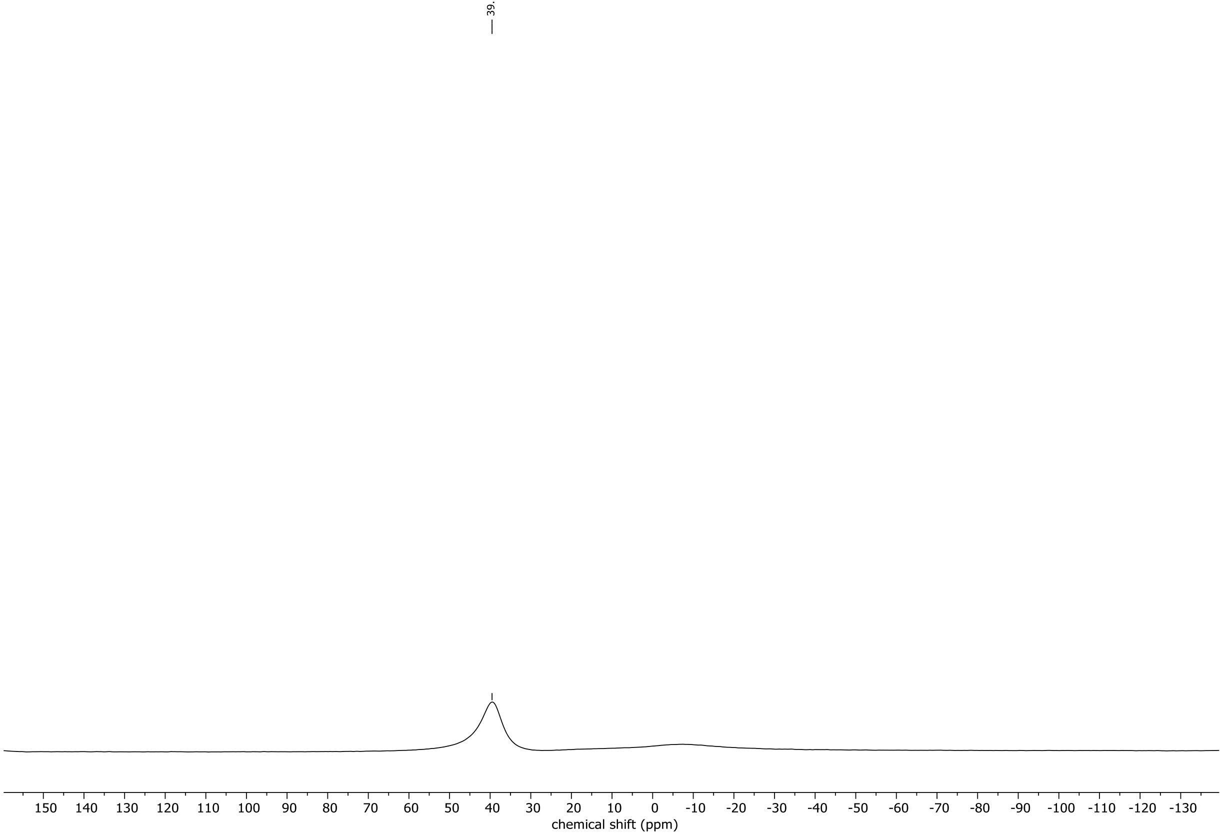

^{19}F NMR (282 MHz, CD_2Cl_2)

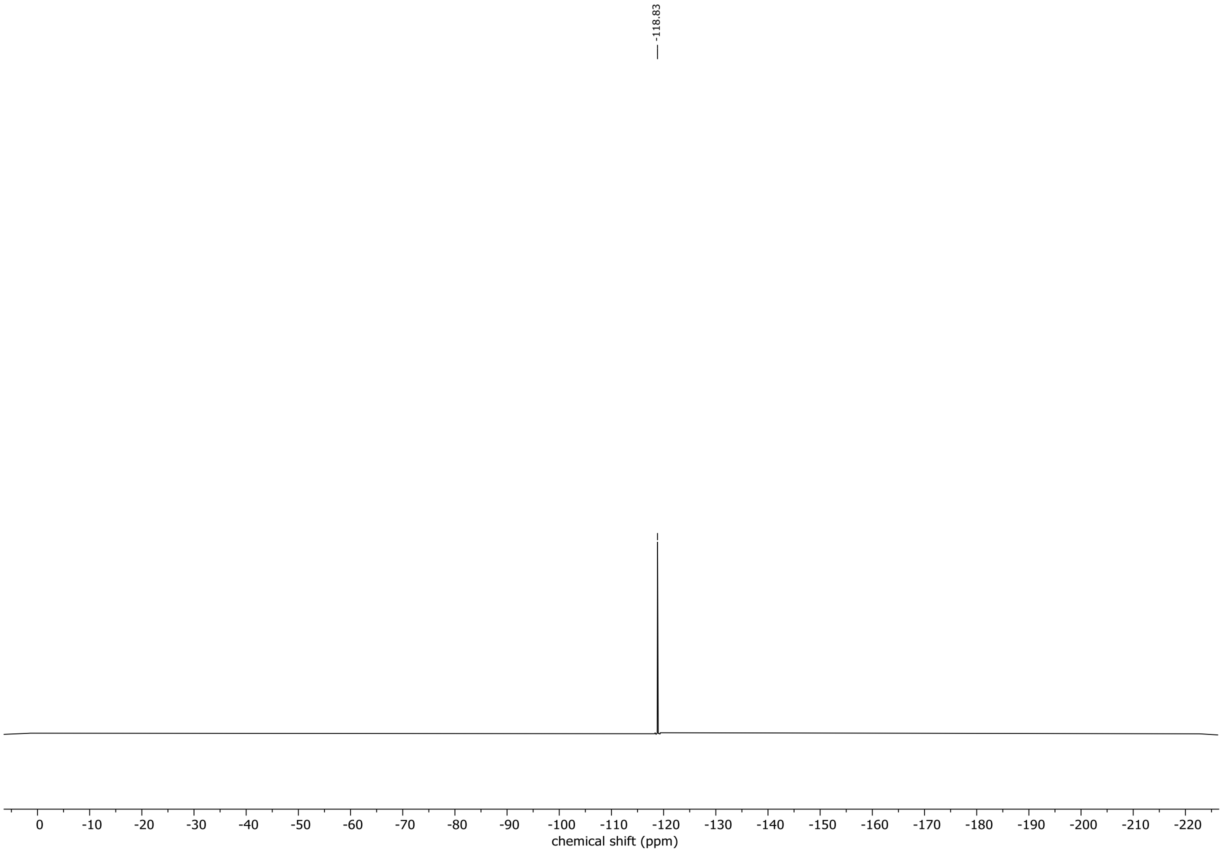

^{1}H NMR (500 MHz, CD_2Cl_2, –35 °C) 1f

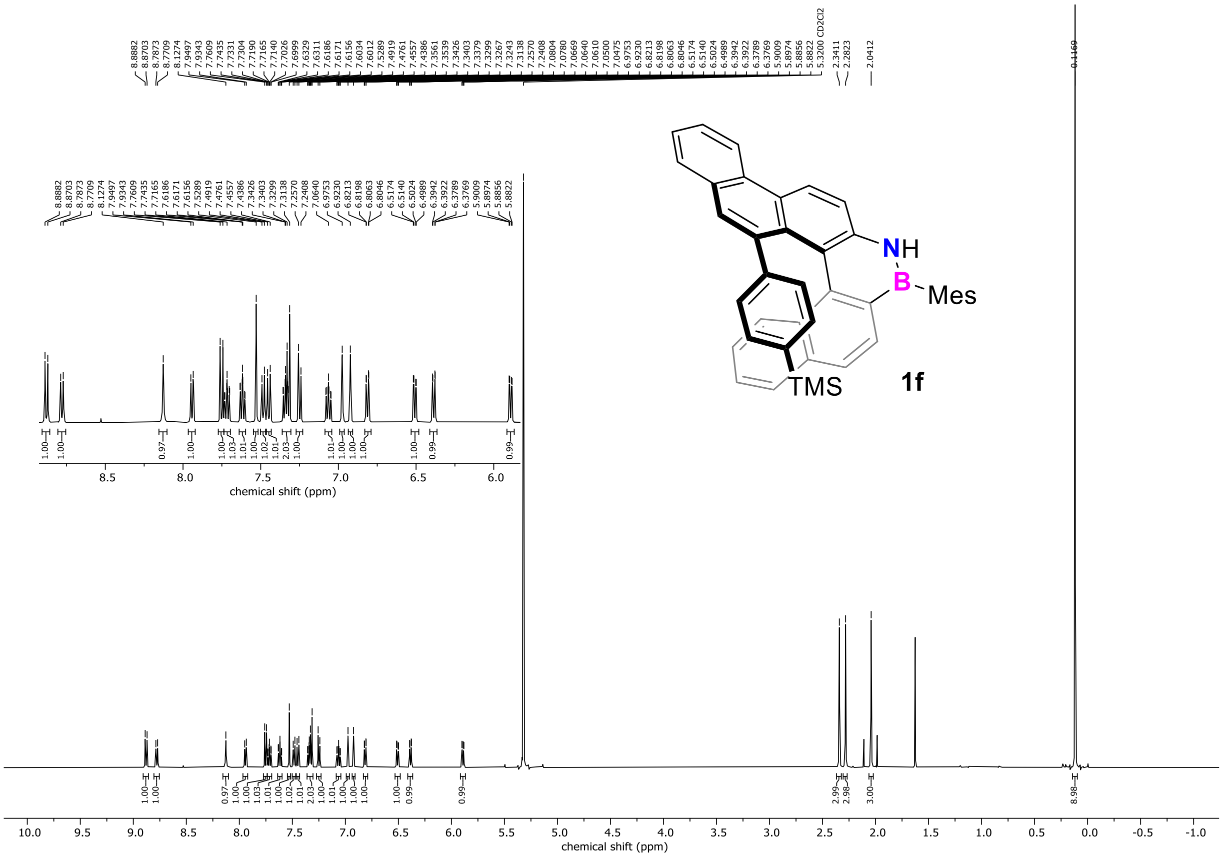

^{1}H NMR (300 MHz, CD_2Cl_2; rt)

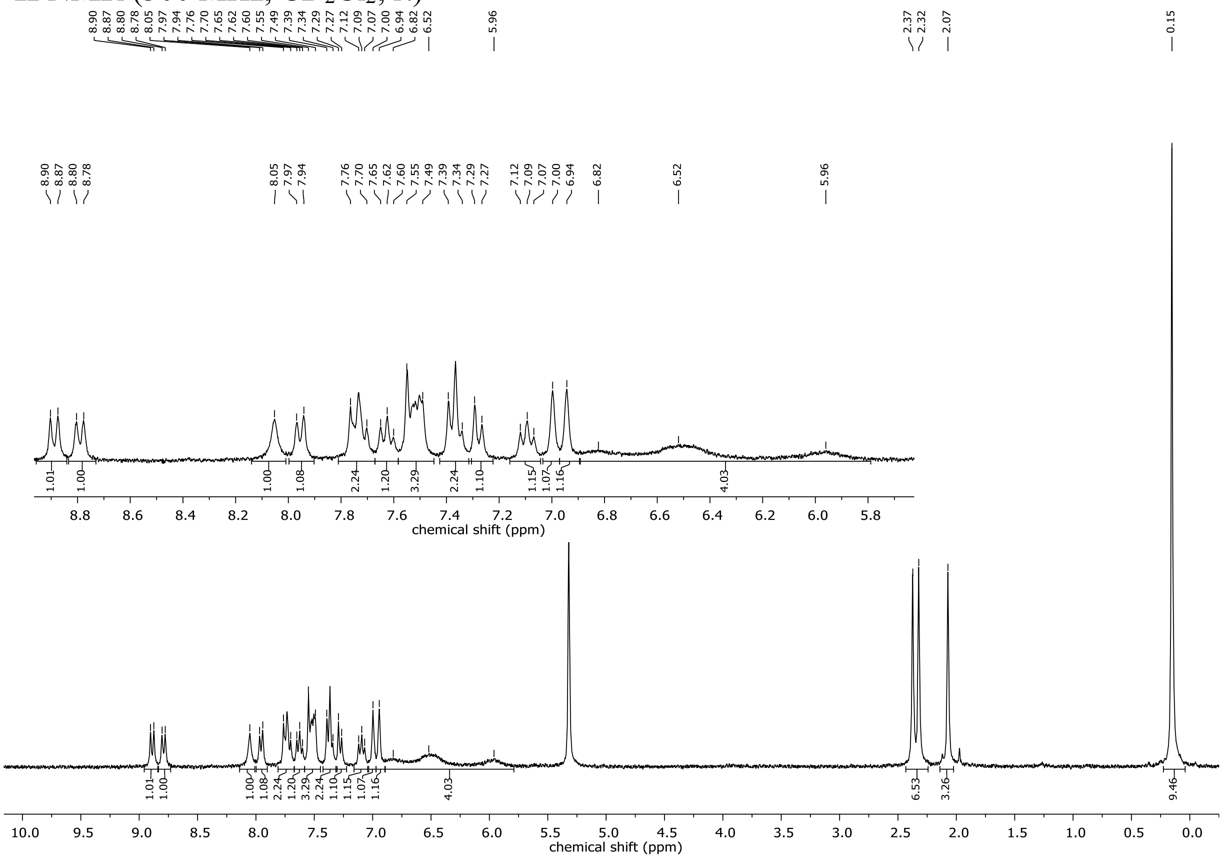

^{13}C NMR (126 MHz, CD_2Cl_2, –35 °C)

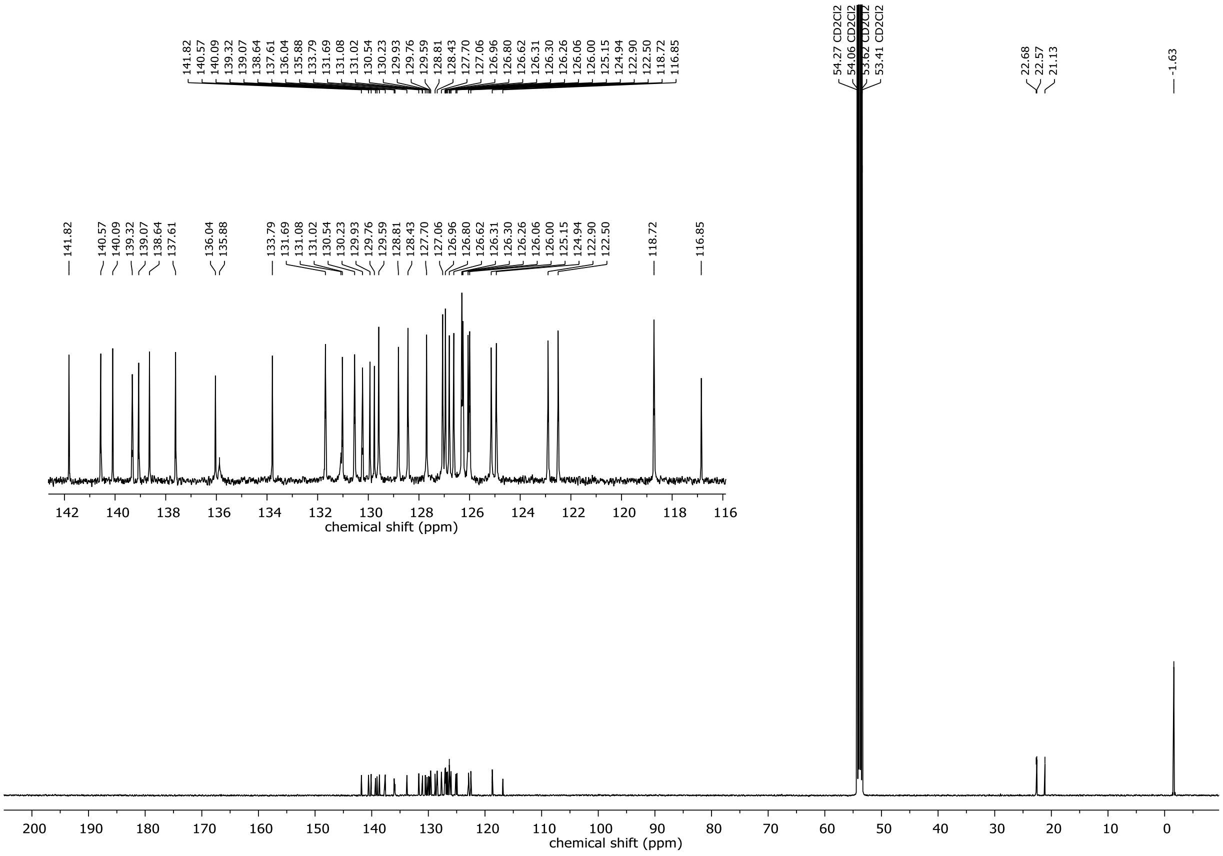

^{11}B NMR (161 MHz, CD_2Cl_2)

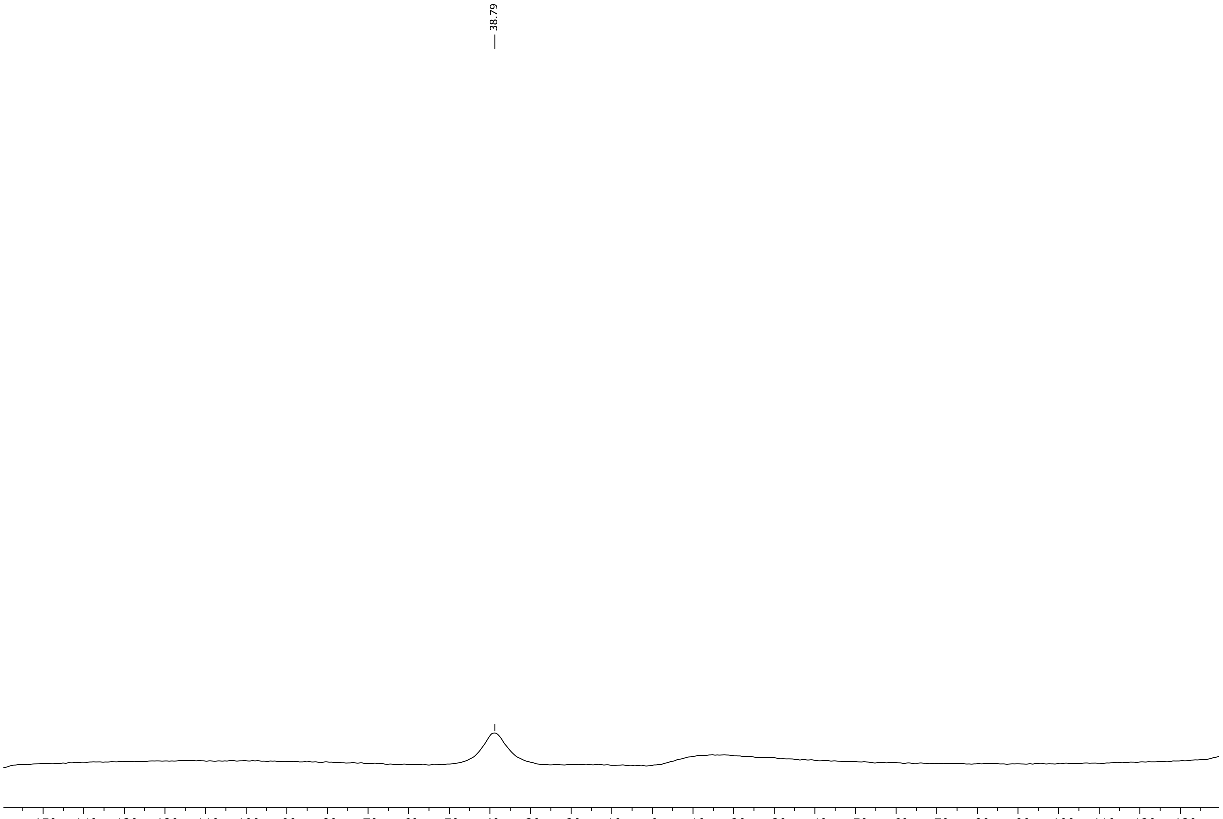

^{1}H NMR (500 MHz, CD_2Cl_2, –35 °C) **1g**

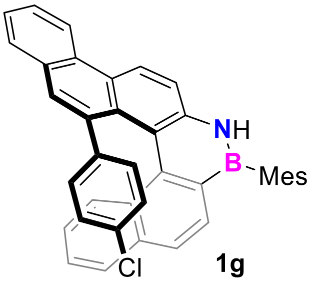

1g

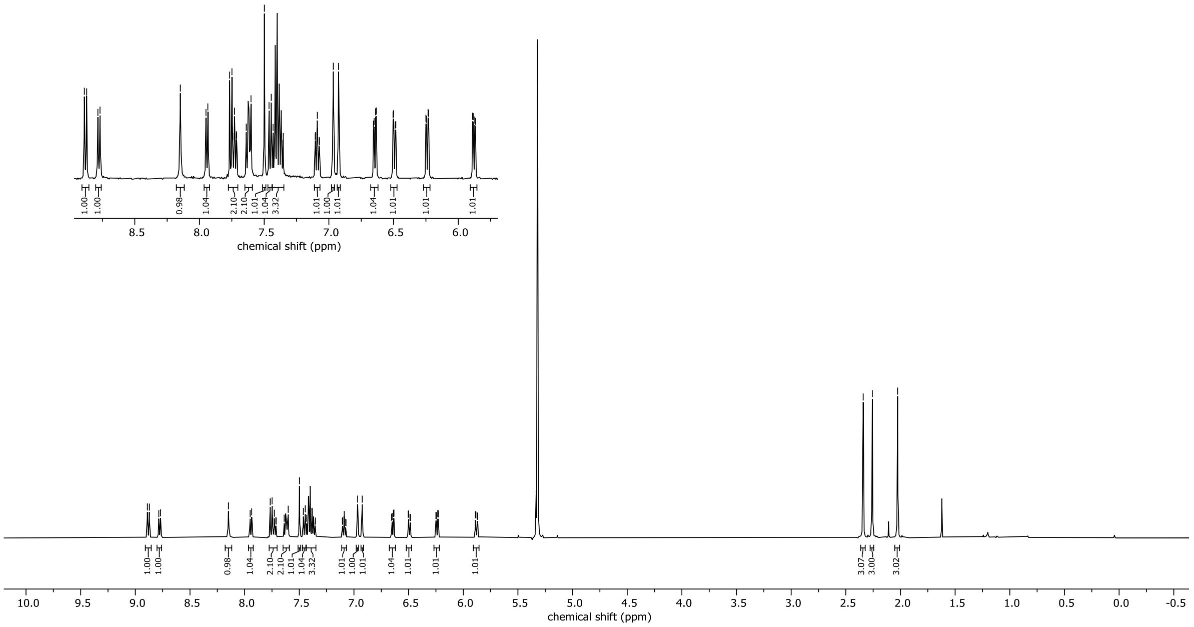

^{1}H NMR (300 MHz, CD_2Cl_2; rt)

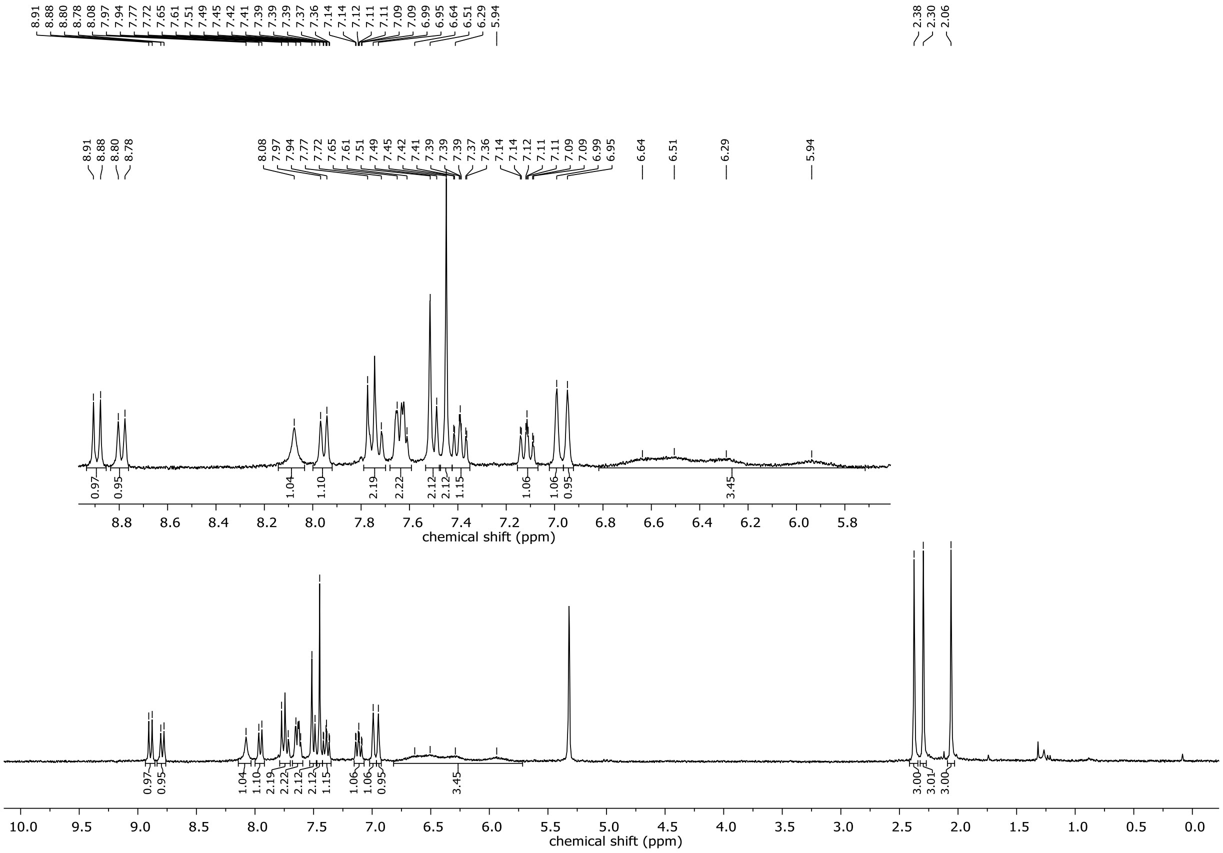

^{13}C NMR (126 MHz, CD_2Cl_2, –35 °C)

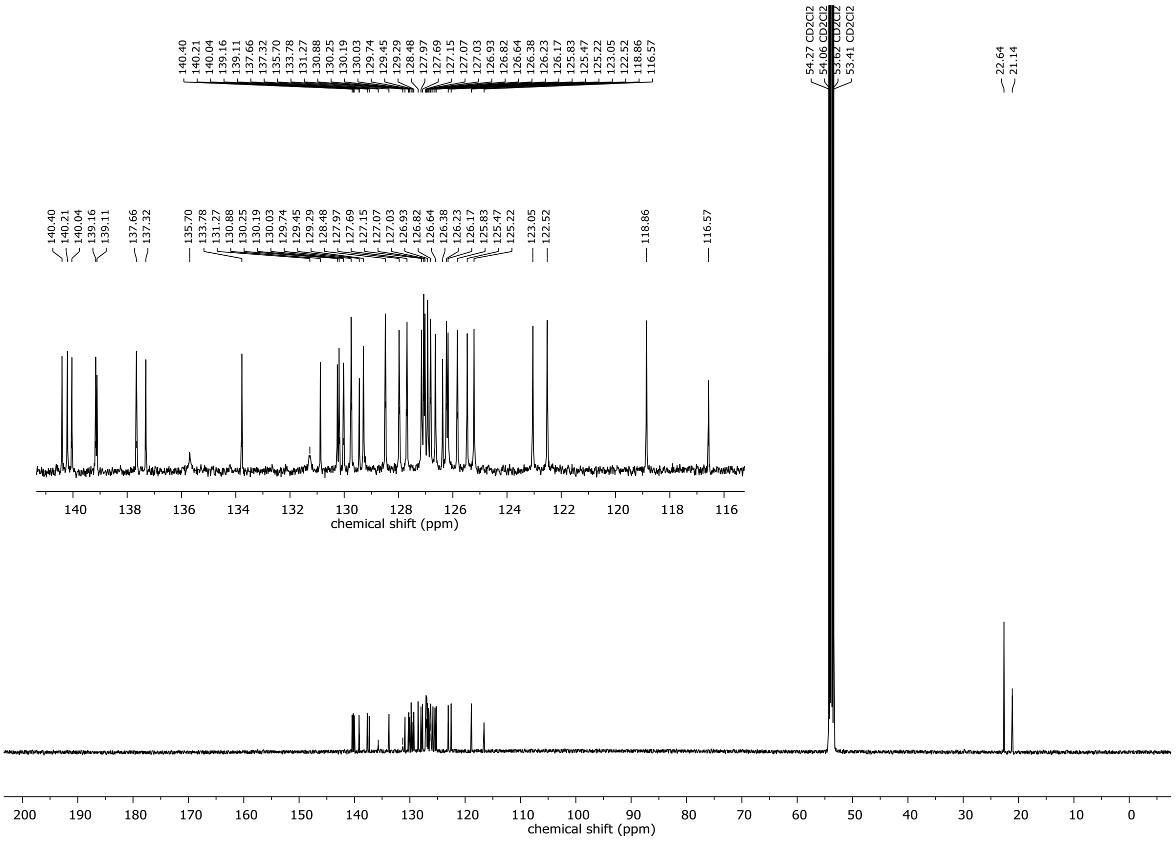

^{11}B NMR (161 MHz, CD_2Cl_2)

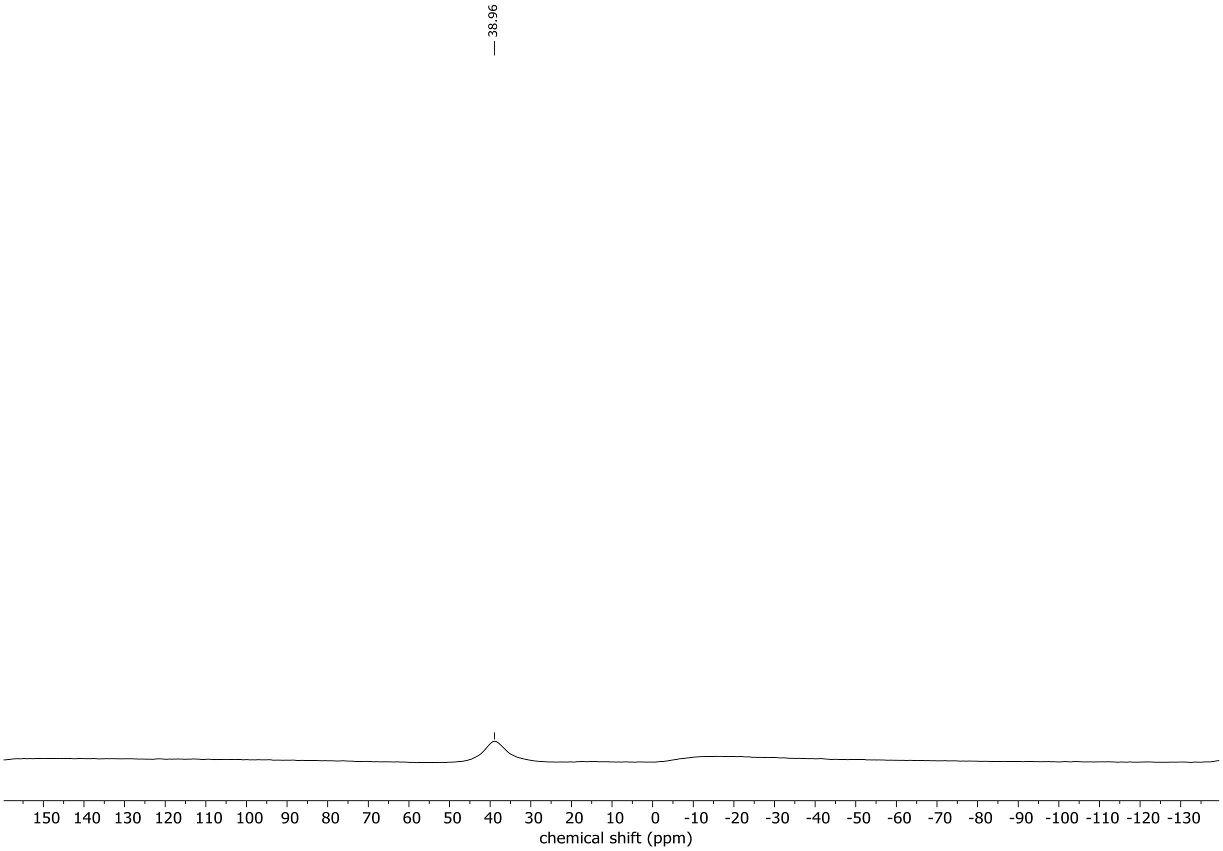

^{1}H NMR (500 MHz, CD_2Cl_2, –35 °C) **1h**

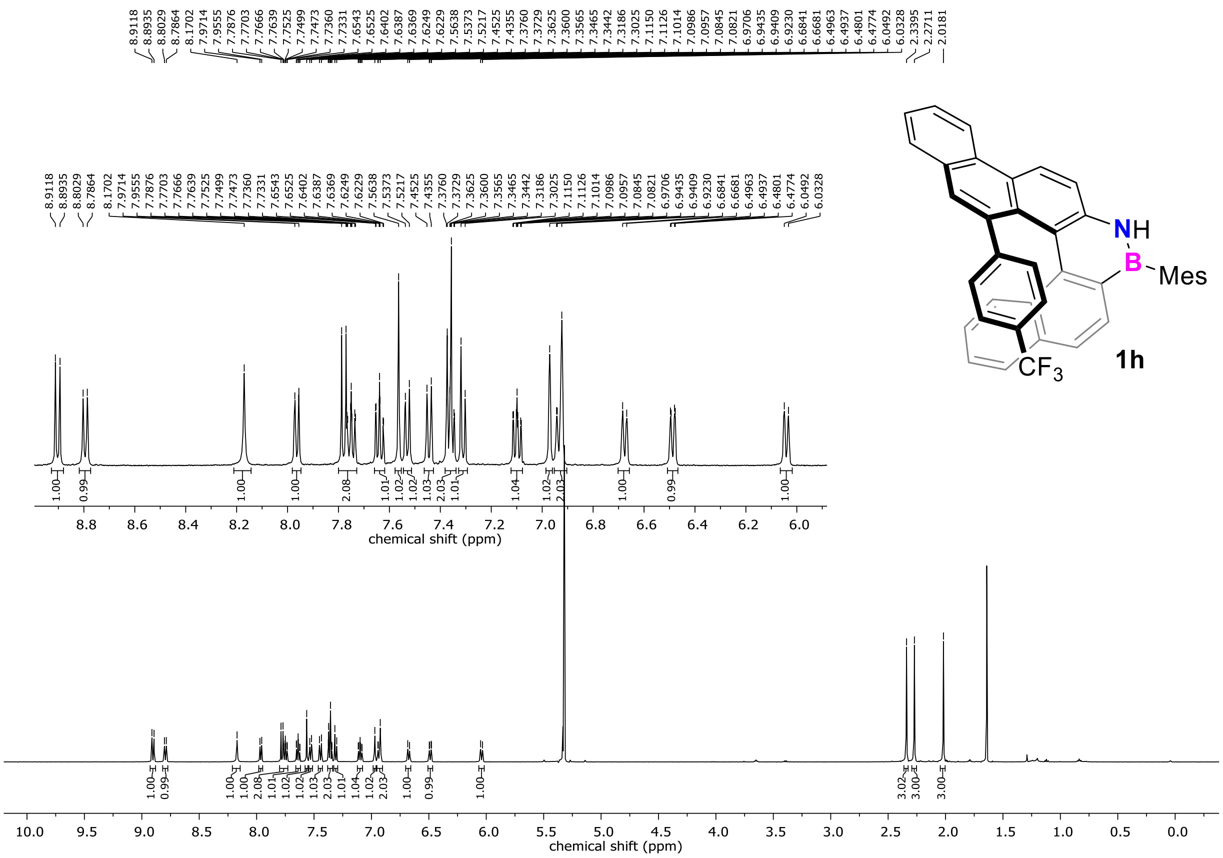

^{1}H NMR (300 MHz, CD_2Cl_2; rt)

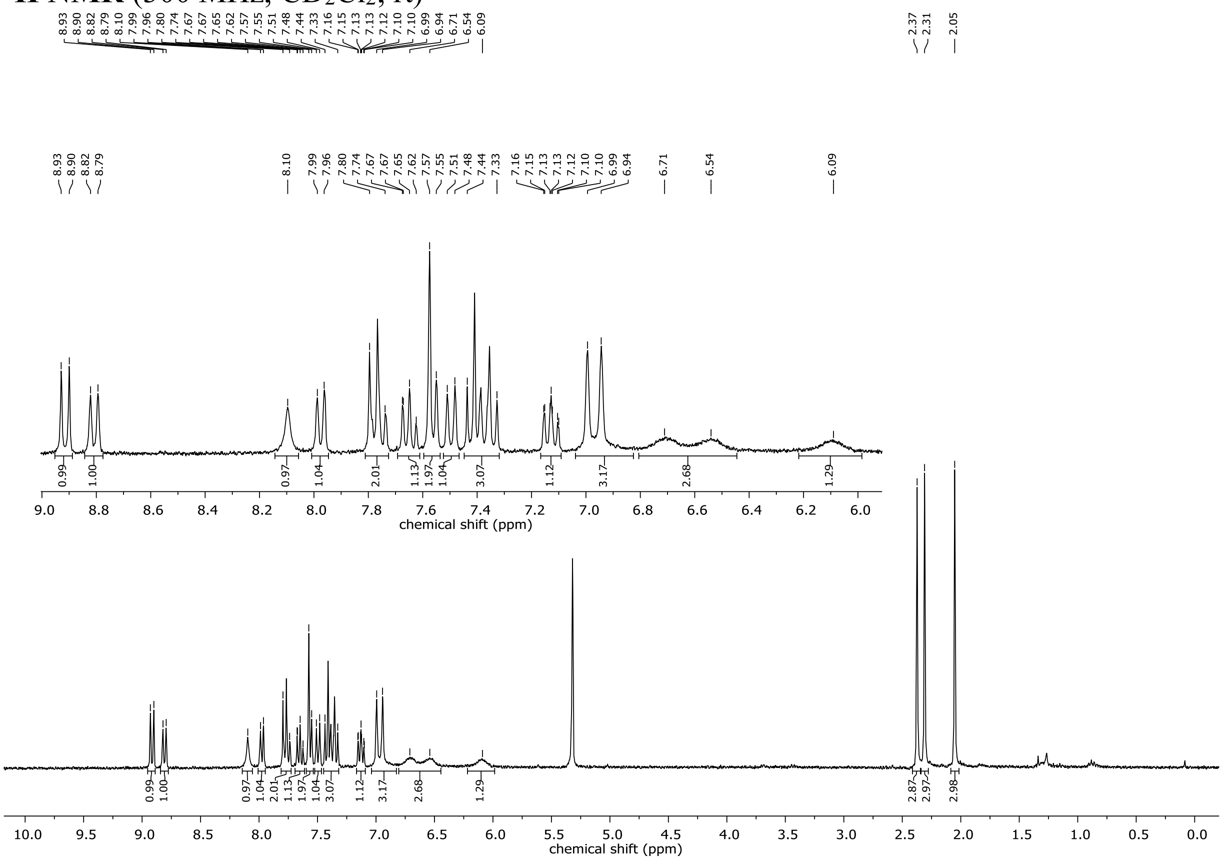

^{13}C NMR (126 MHz, CD_2Cl_2)

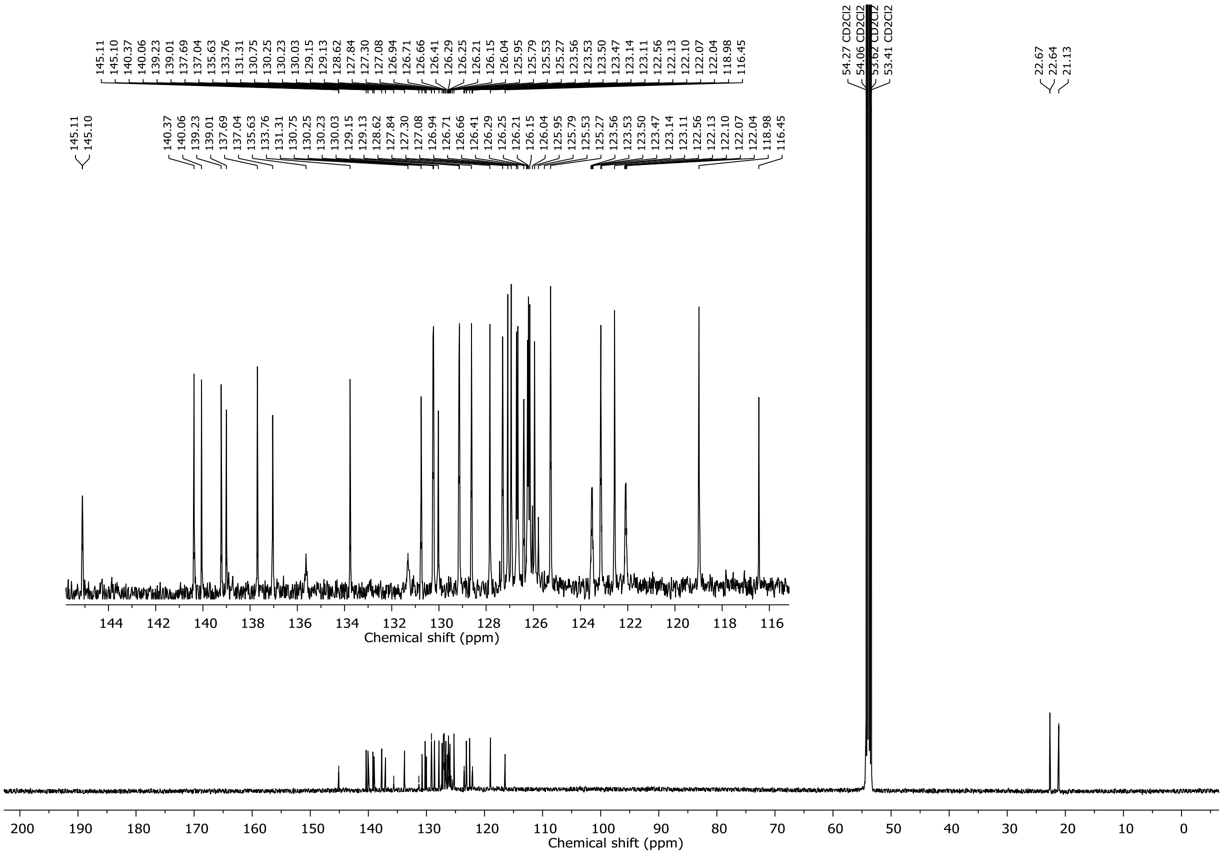

^{11}B NMR (161 MHz, CD_2Cl_2)

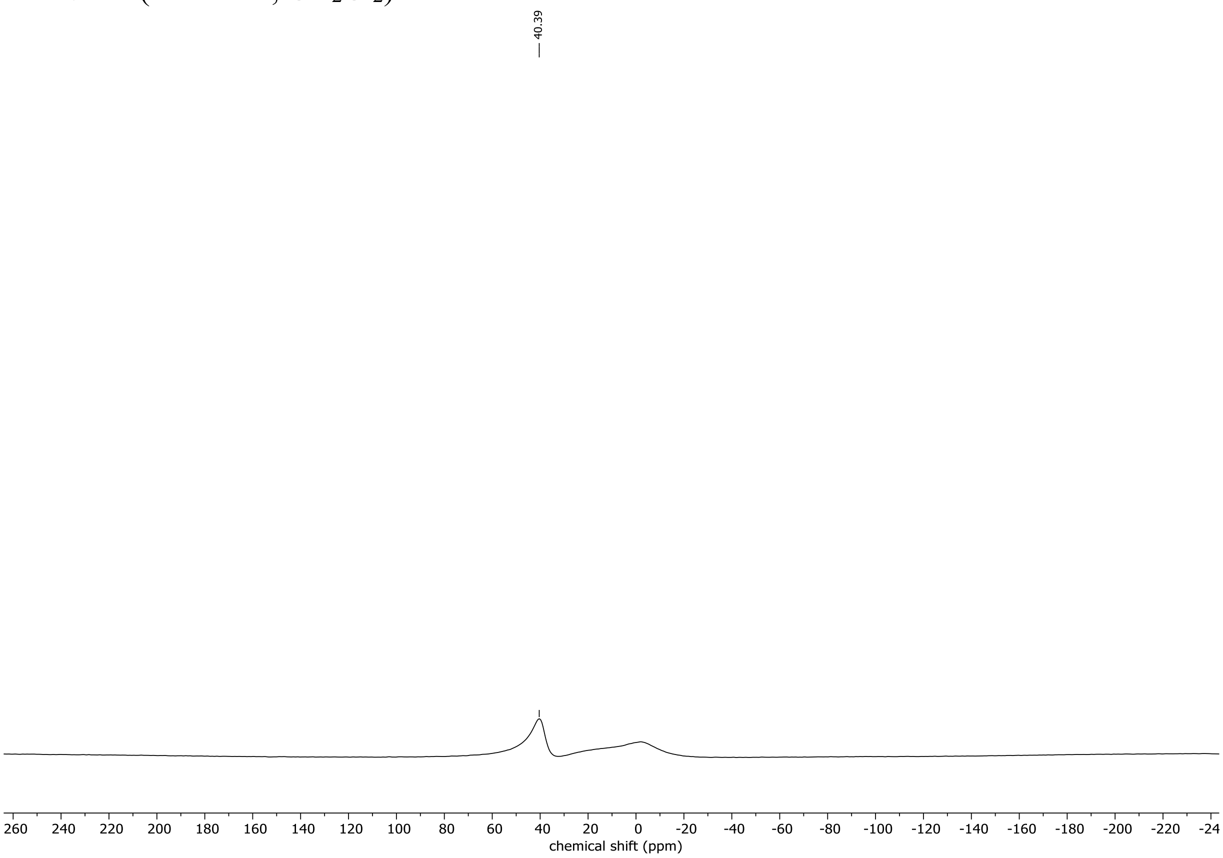

^{19}F NMR (377 MHz, $CDCl_3$)

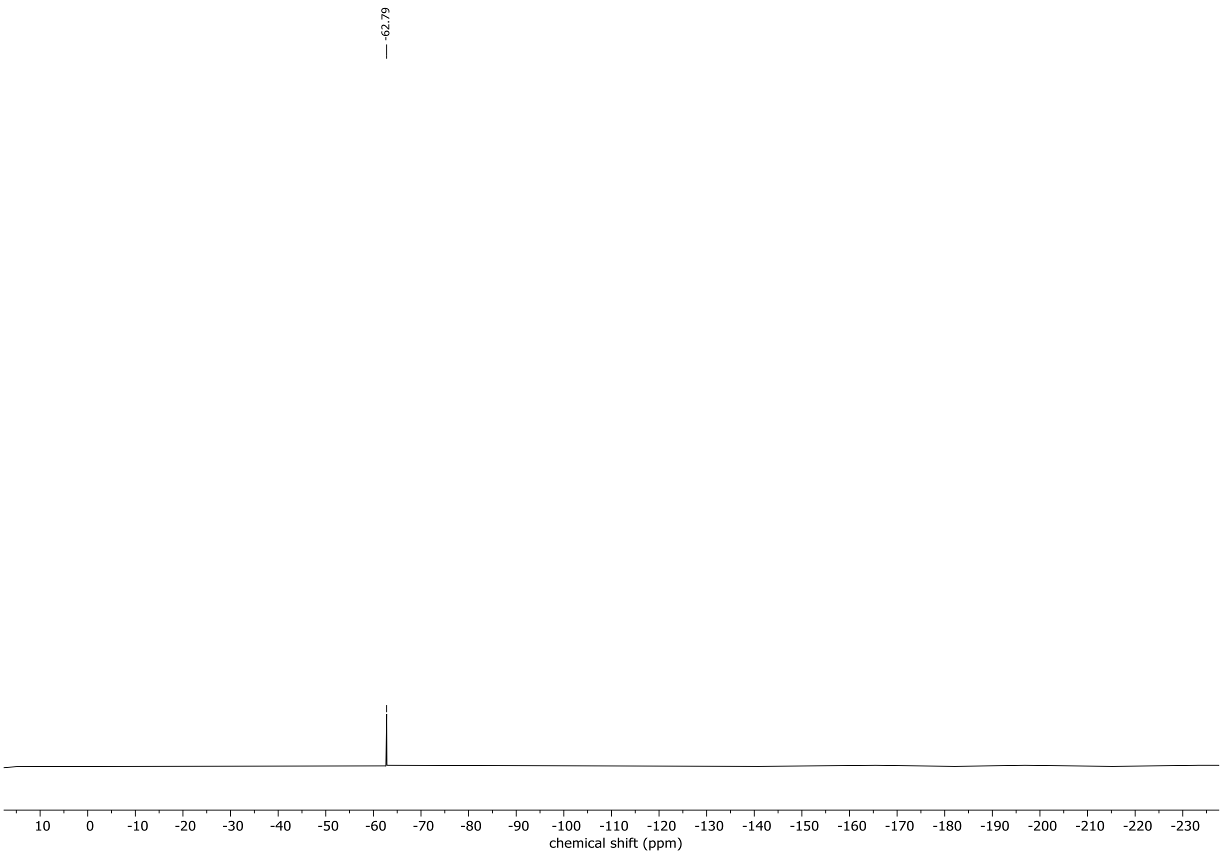

^{1}H NMR (400 MHz, $C_2D_2Cl_4$, 140 °C) **1i**

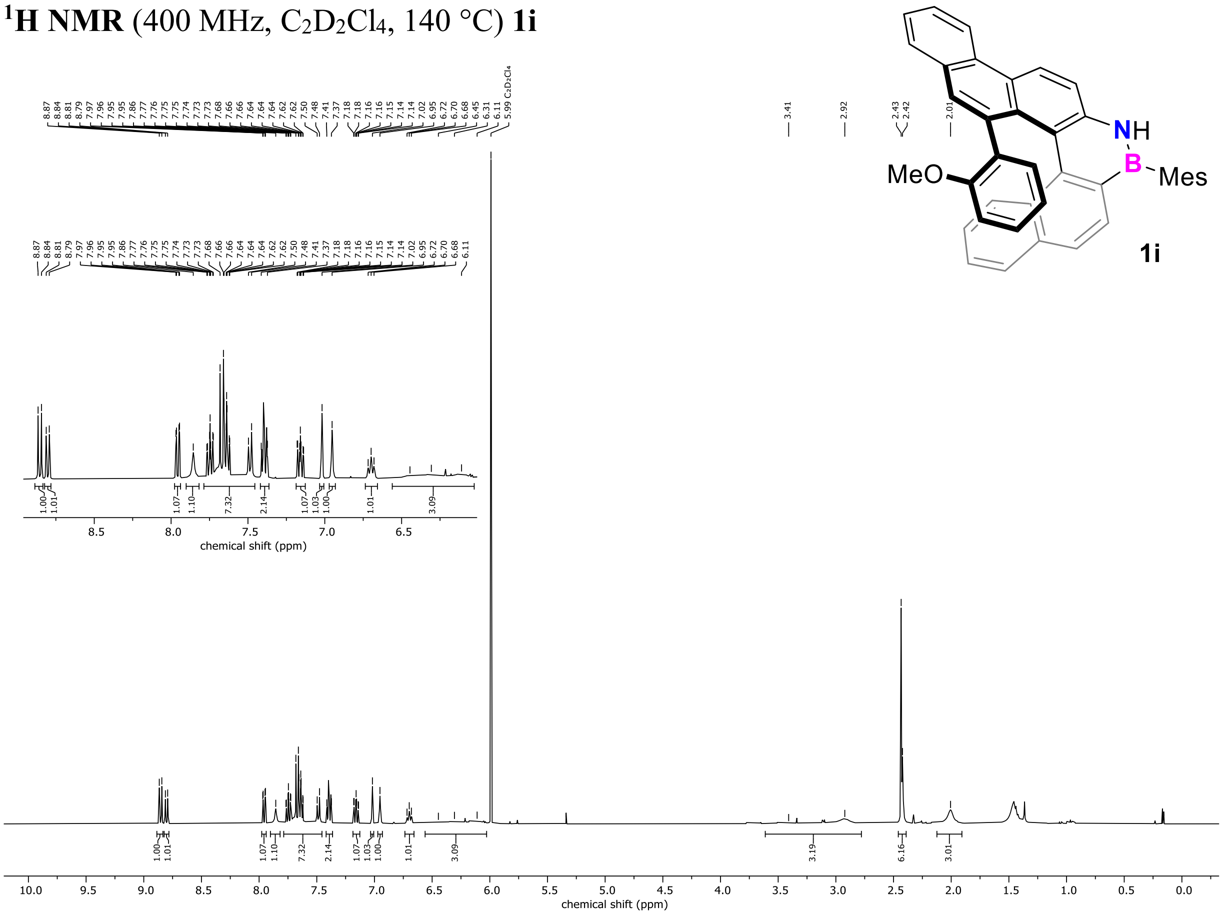

^{1}H NMR (400 MHz, $CDCl_3$, rt)

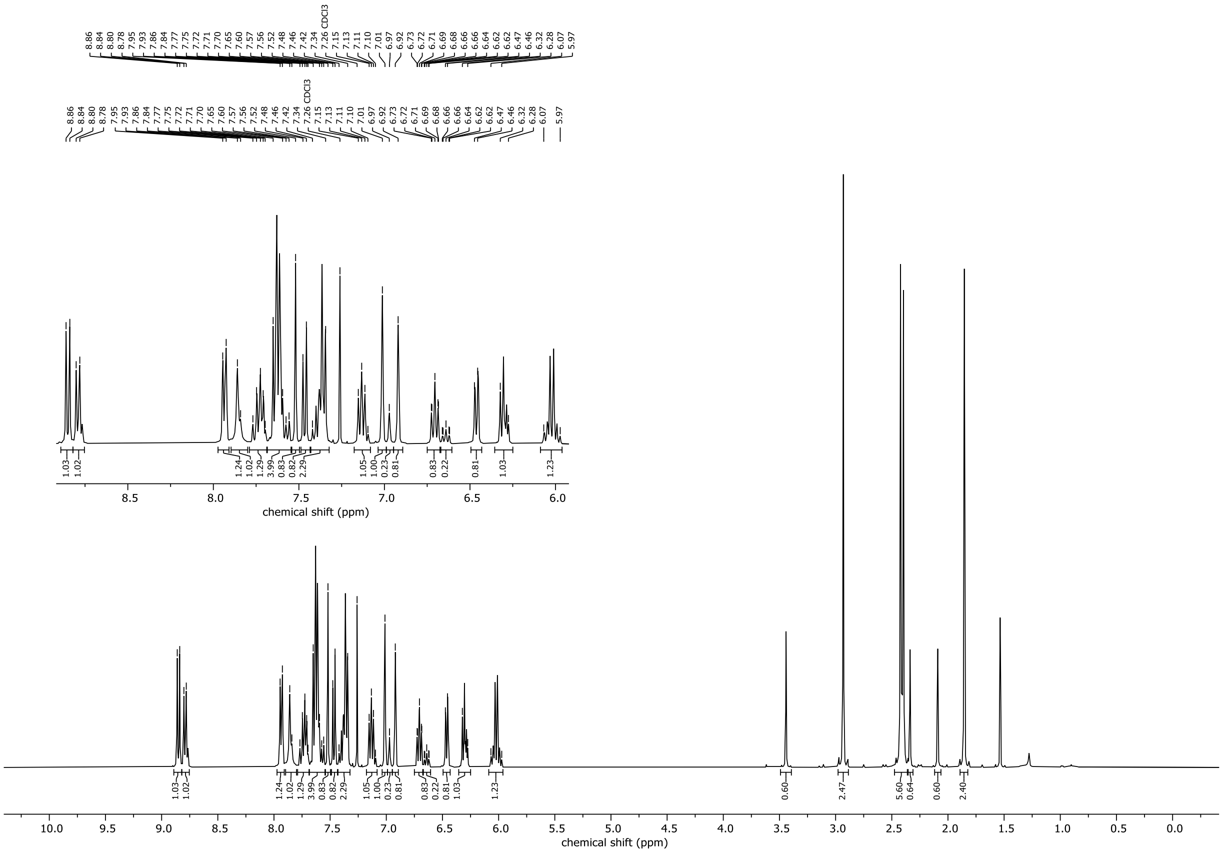

^{13}C NMR (101 MHz, $CDCl_3$, 25 °C)

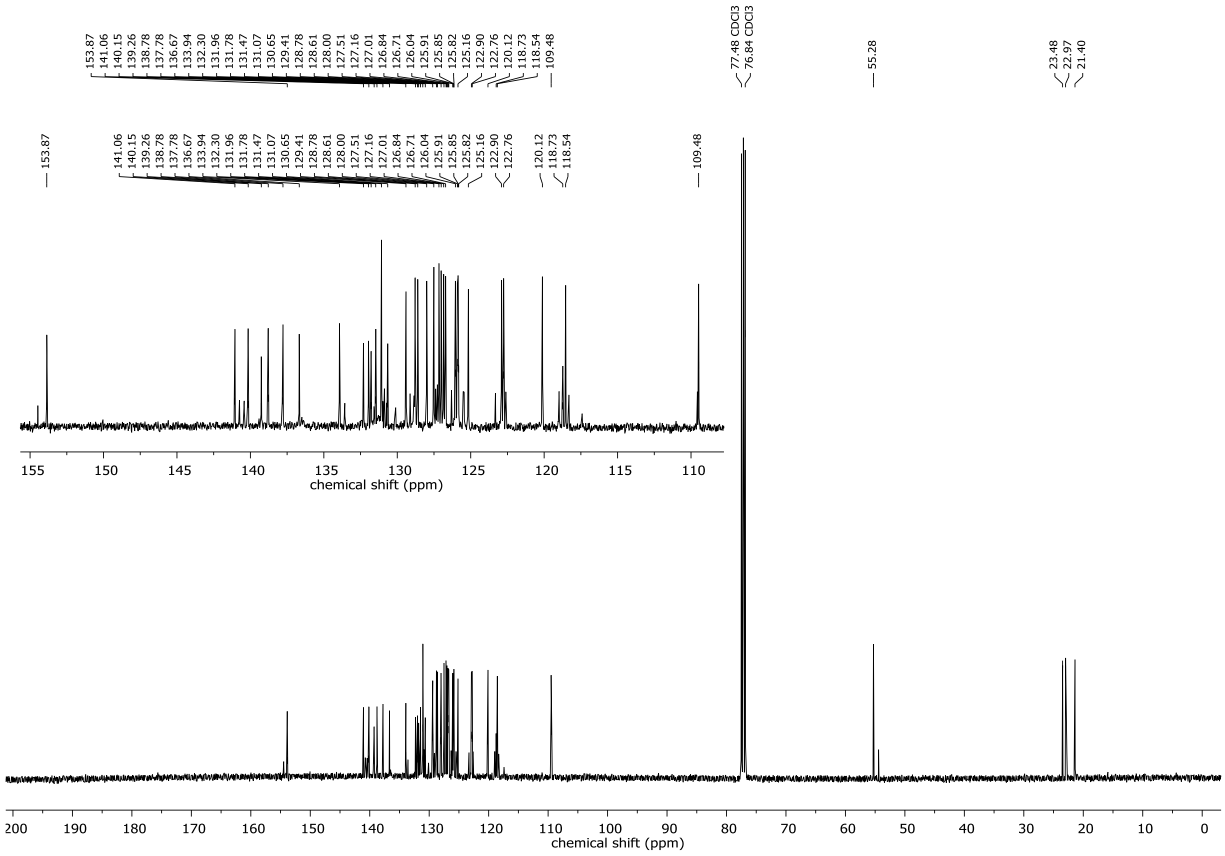

^{11}B NMR (128 MHz, $CDCl_3$, 25 °C)

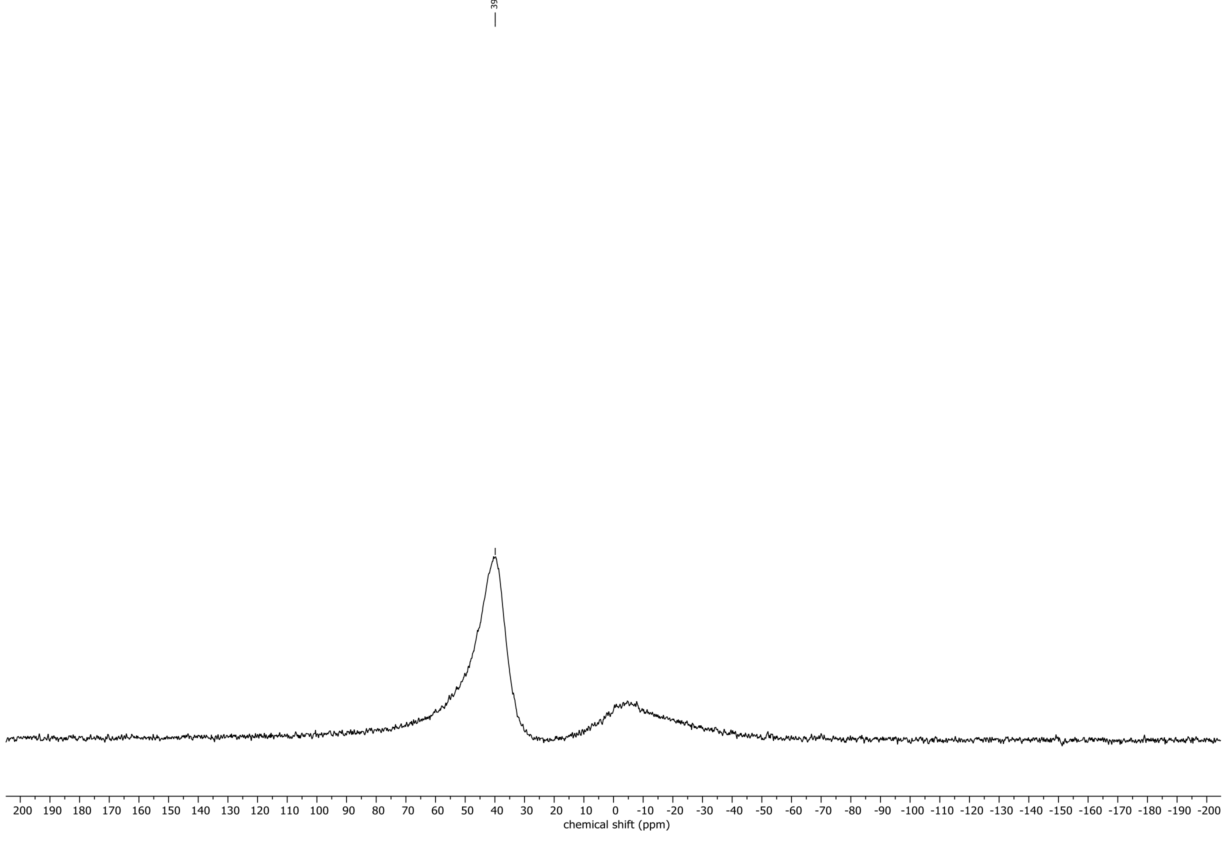

^{1}H NMR (600 MHz, $CDCl_3$, 60 °C) **1j**

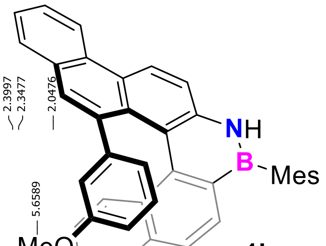

1j

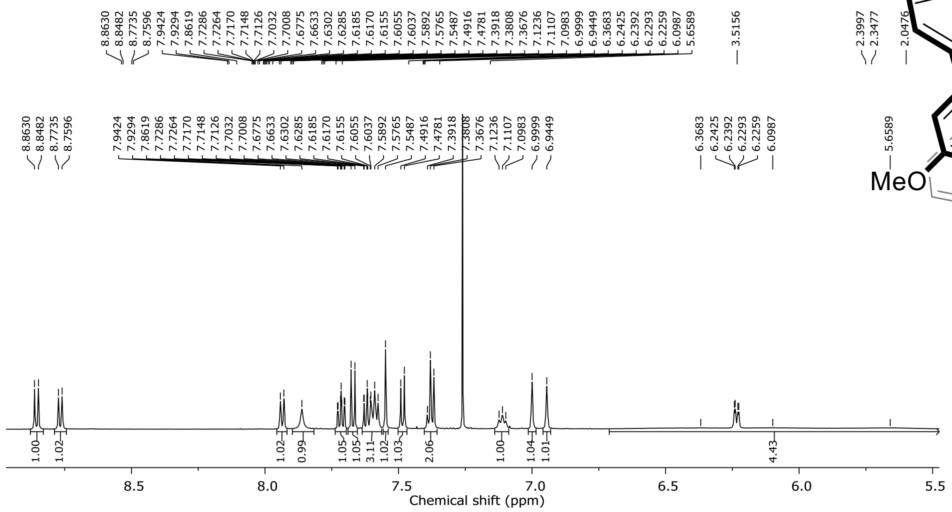

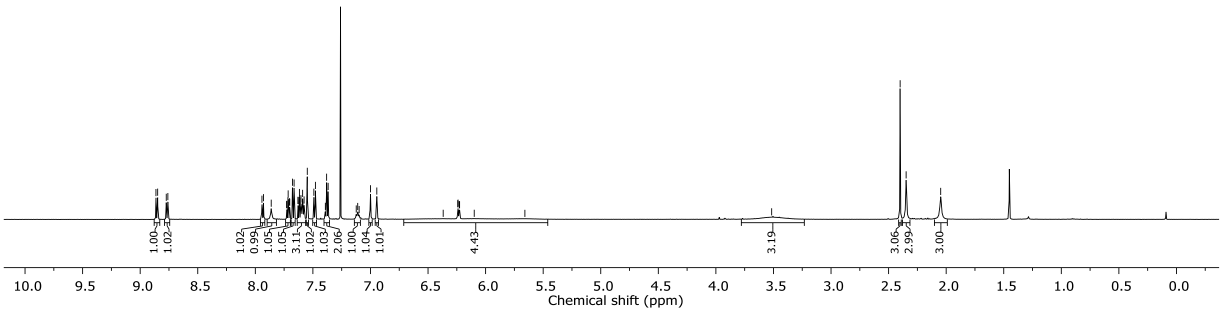

^{13}C NMR (126 MHz, $CDCl_3$, 60 °C)

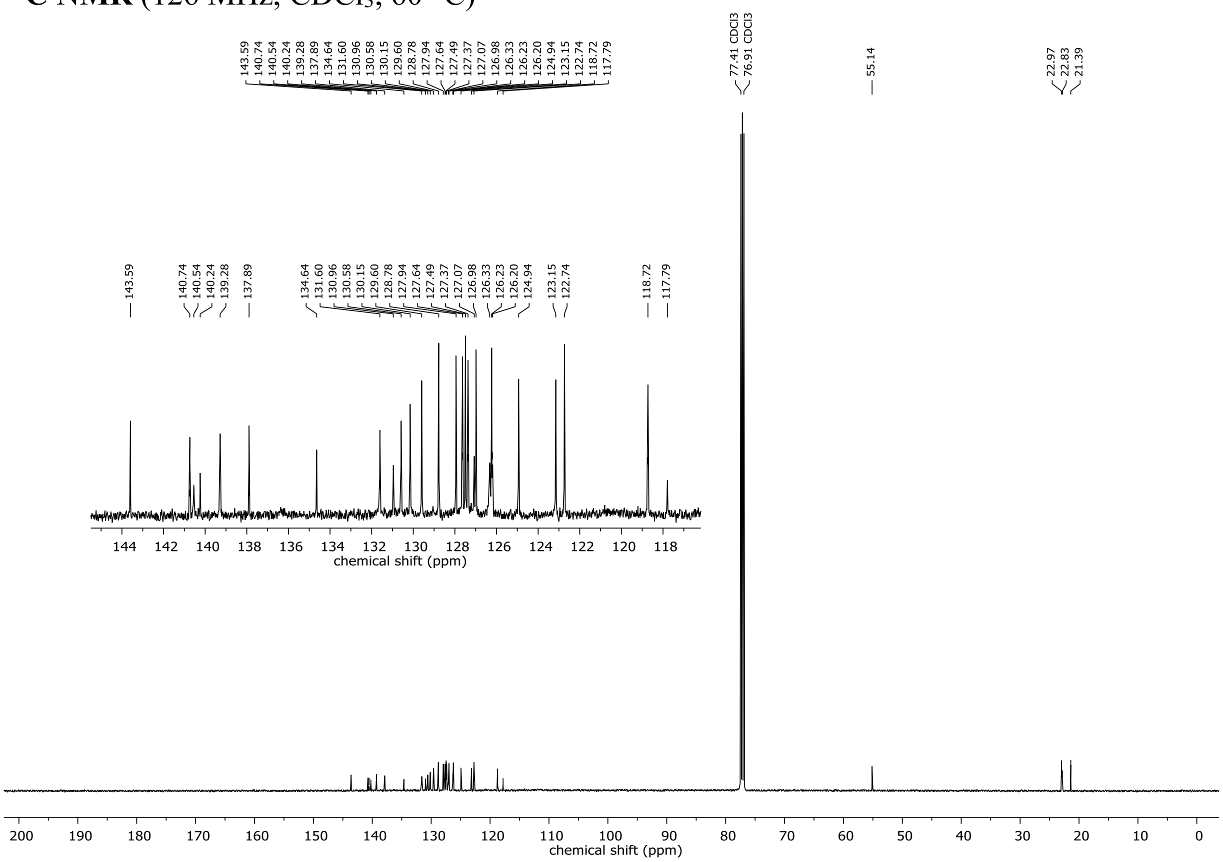

^{11}B NMR (161 MHz, CD_2Cl_2)

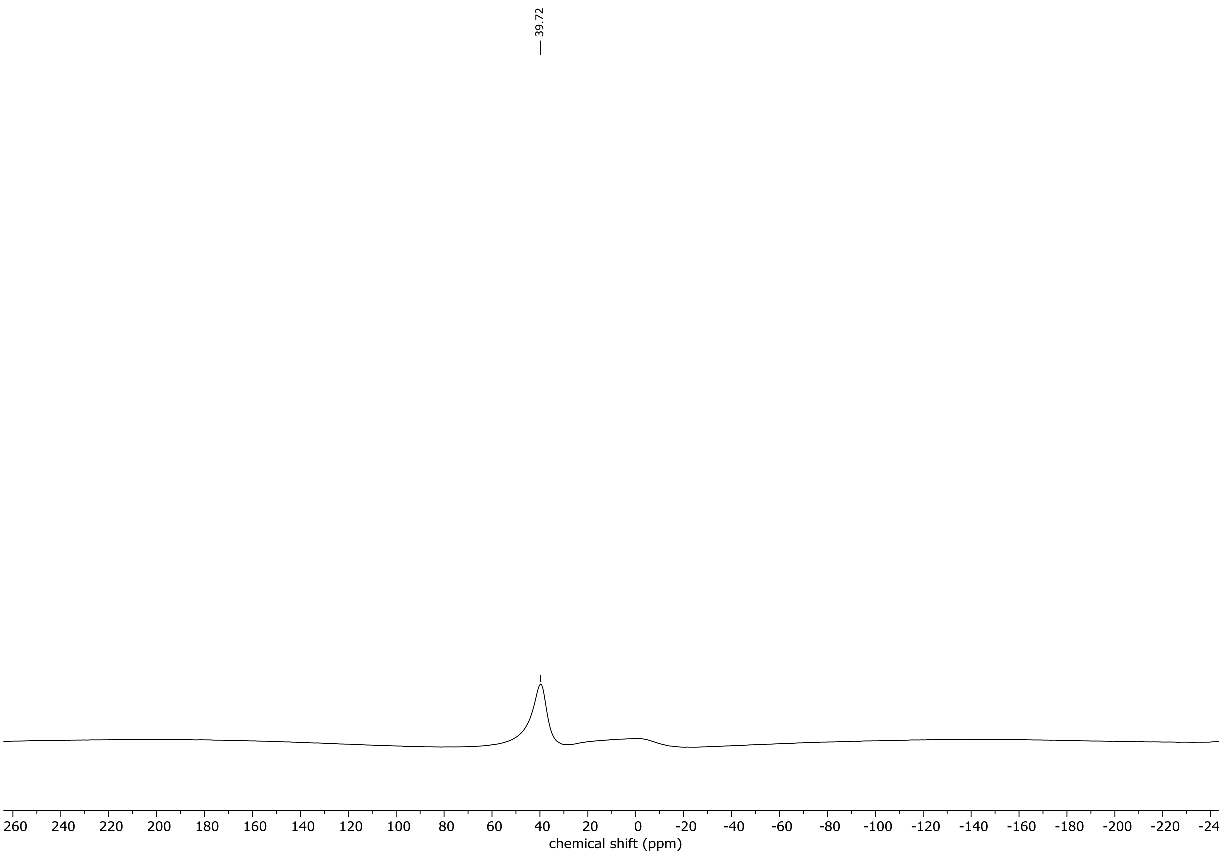

^{1}H NMR (600 MHz, CD_2Cl_2, -50ºC) 1b(BPh)

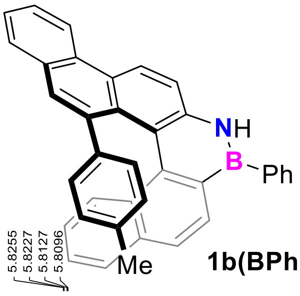

1b(BPh)

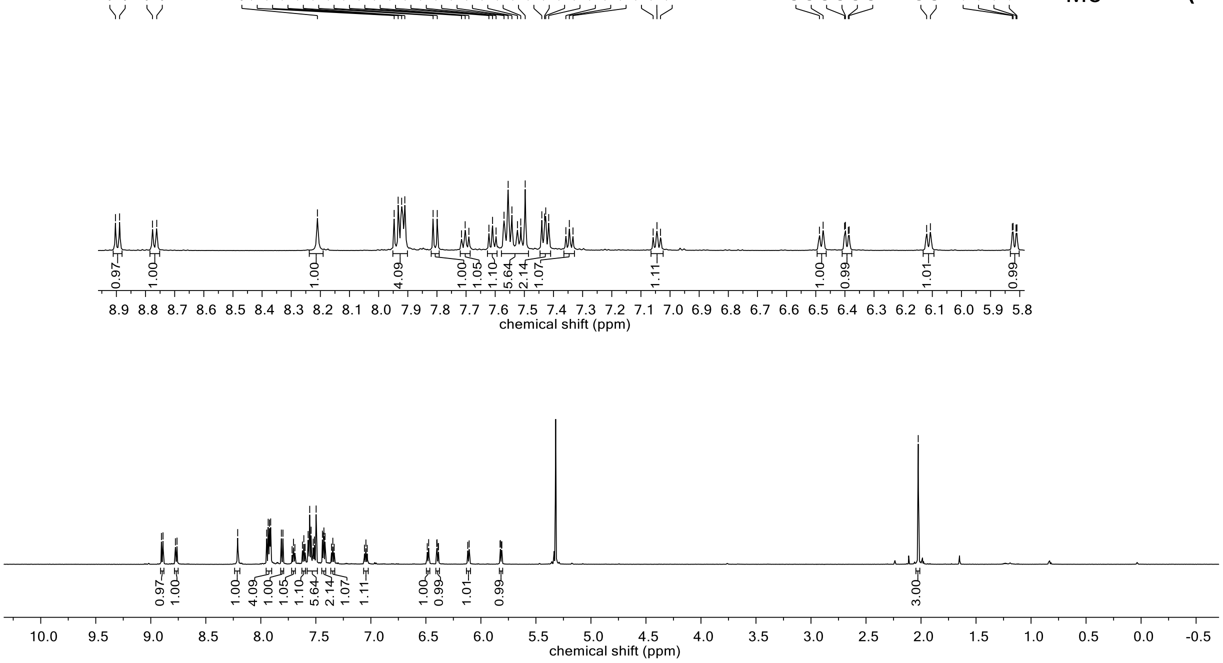

^{1}H NMR (300 MHz, CD_2Cl_2; rt)

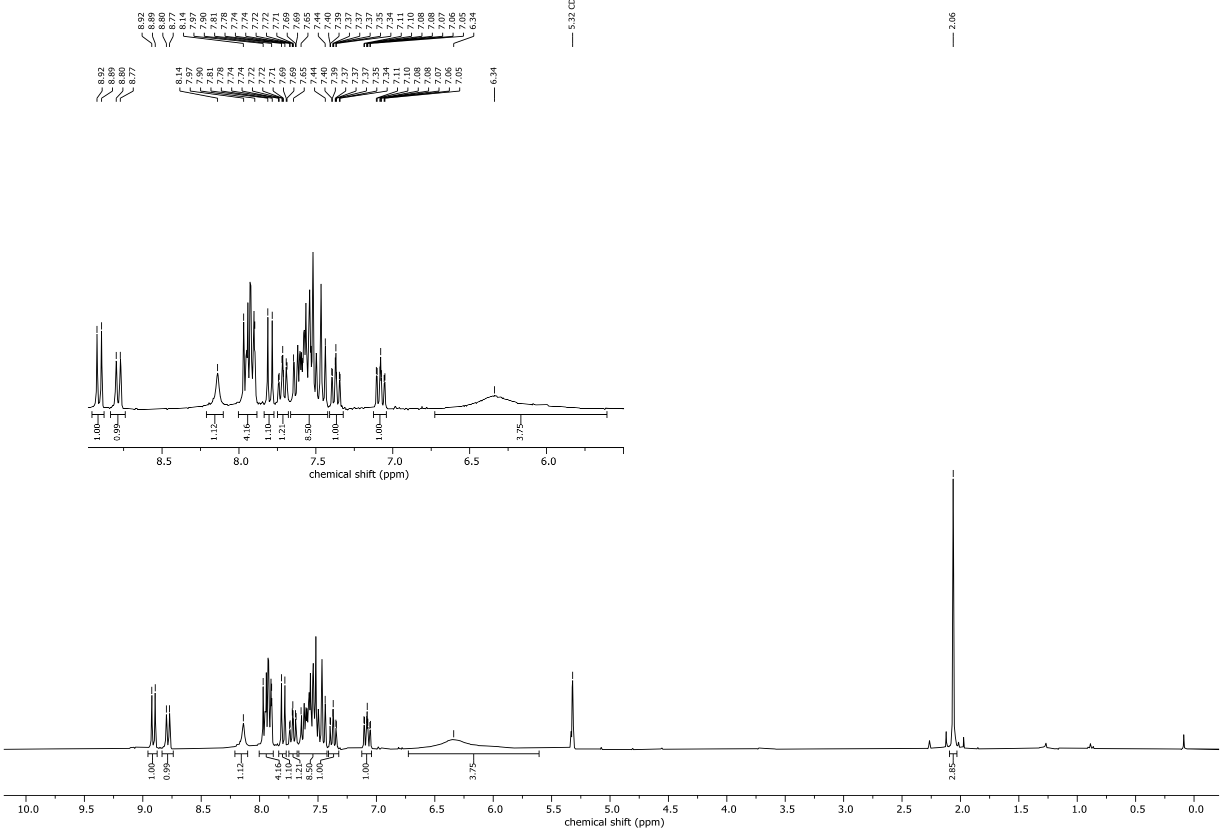

^{13}C NMR (126 MHz, CD_2Cl_2, -35ºC)

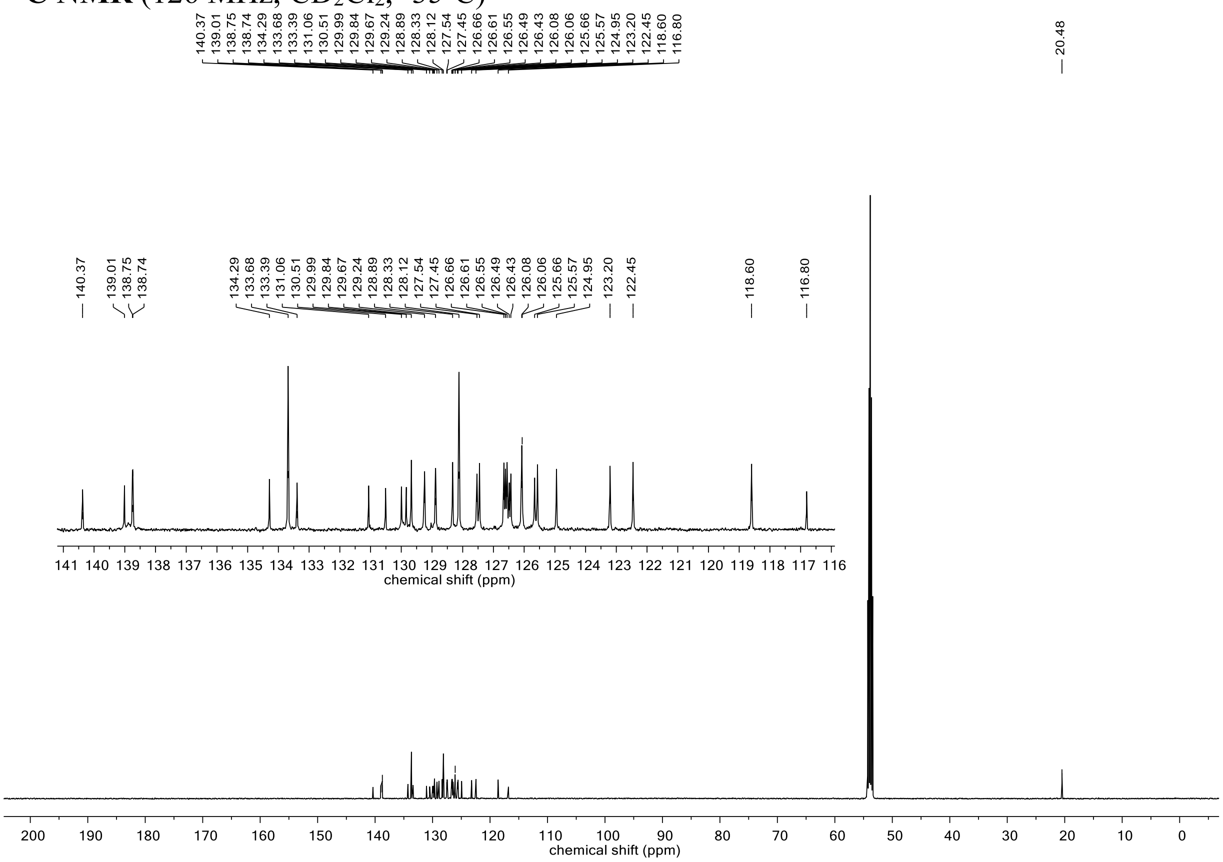

^{11}B NMR (161 MHz, CD_2Cl_2)

^{1}H NMR (500 MHz, CD_2Cl_2, –35 °C) **1b(NMe)**

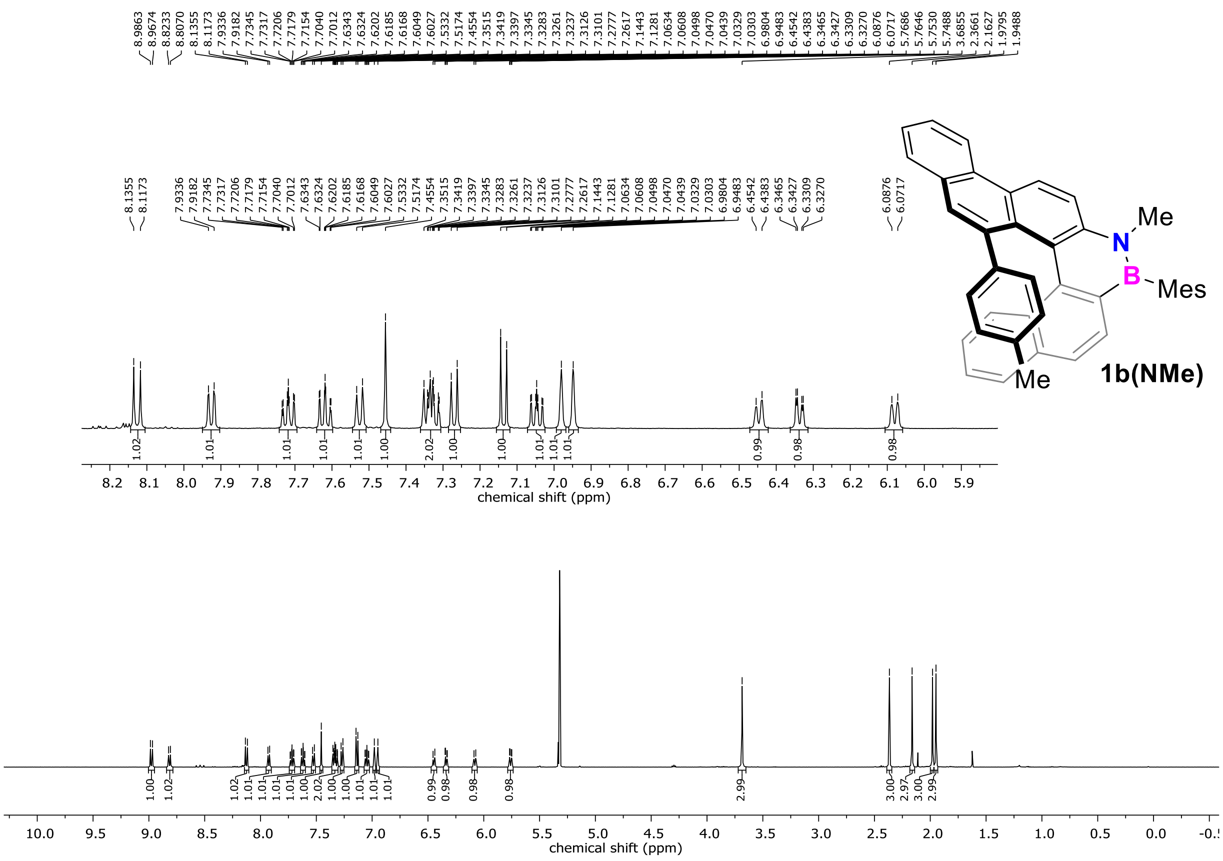

^{13}C NMR (126 MHz, CD_2Cl_2, –35 °C)

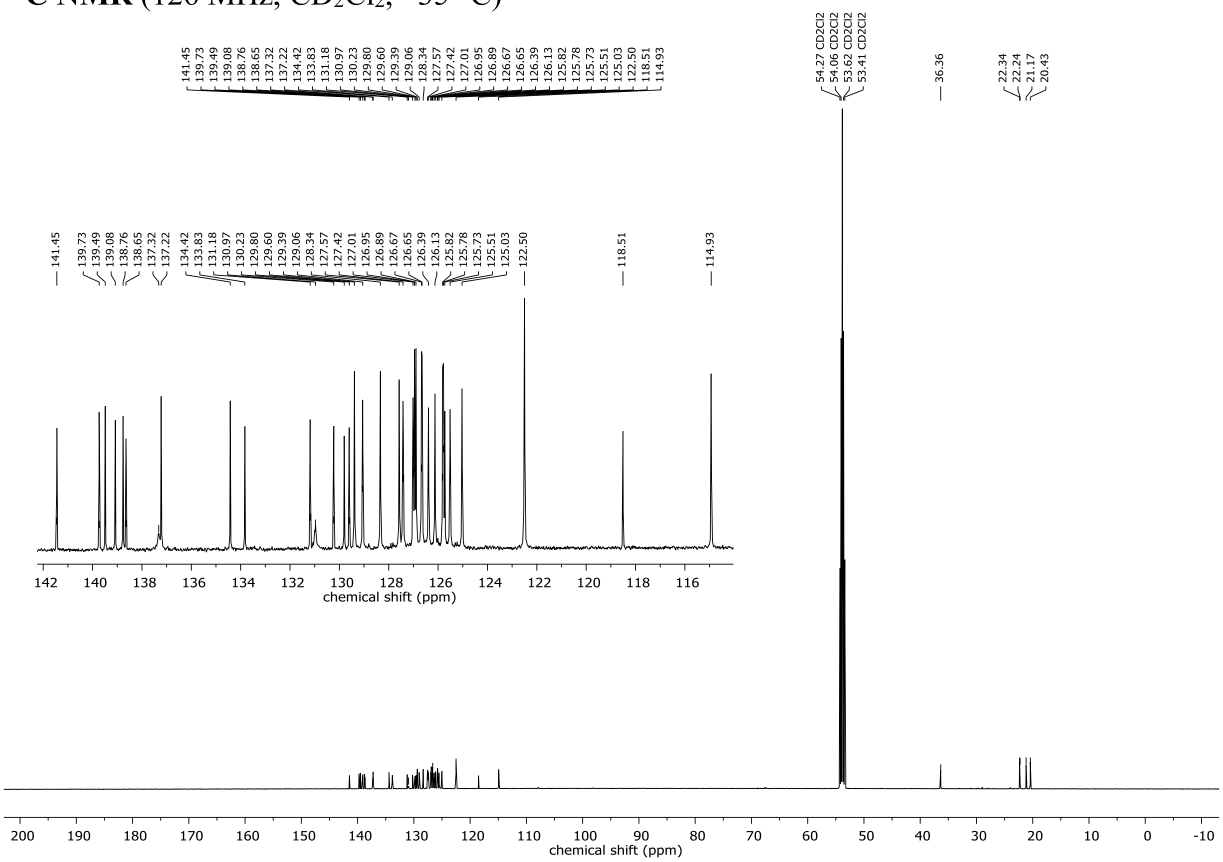

^{11}B NMR (161 MHz, CD_2Cl_2)

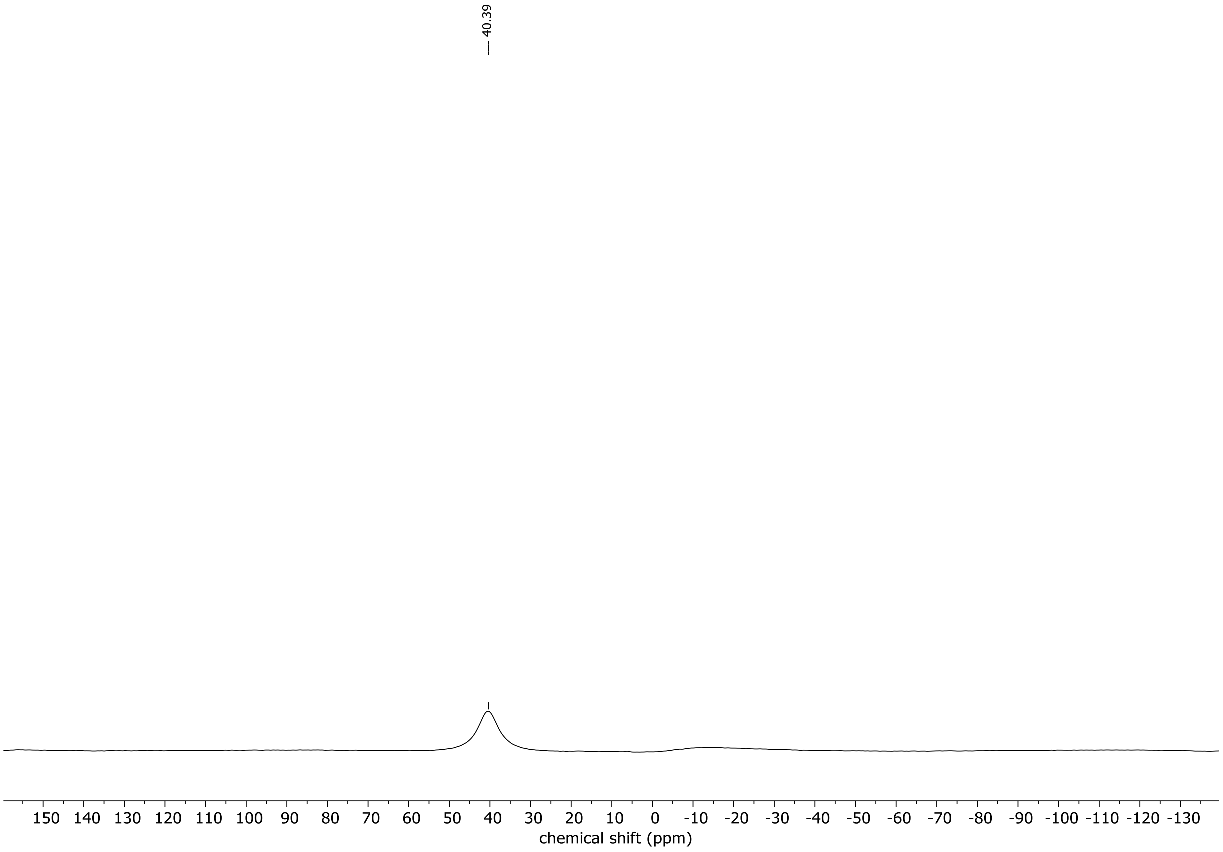

^{1}H NMR (500 MHz, CD_2Cl_2, –35 °C) 2b

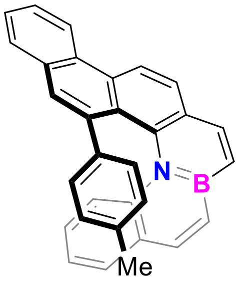

2b

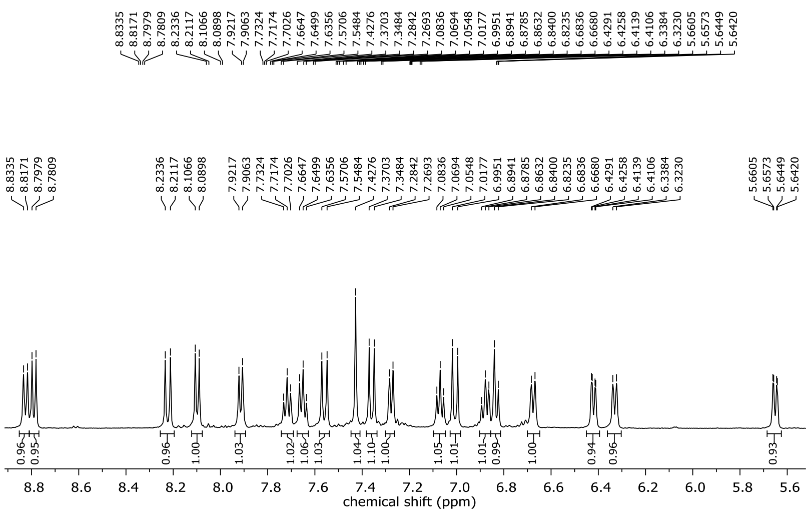

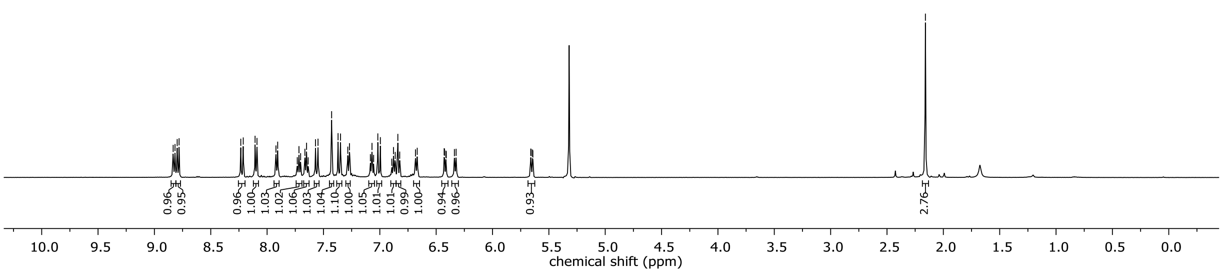

^{1}H NMR (300 MHz, CD_2Cl_2; rt)

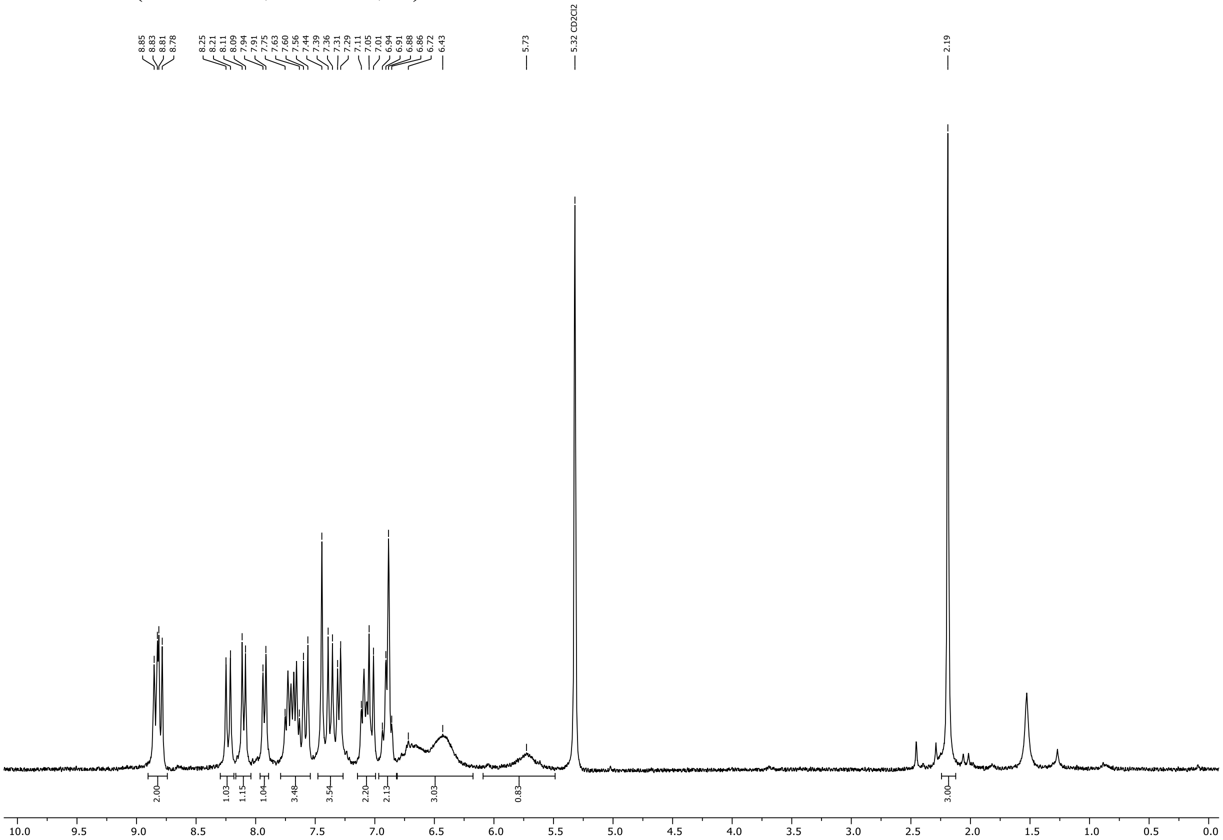

^{13}C NMR (126 MHz, CD_2Cl_2, –35 °C)

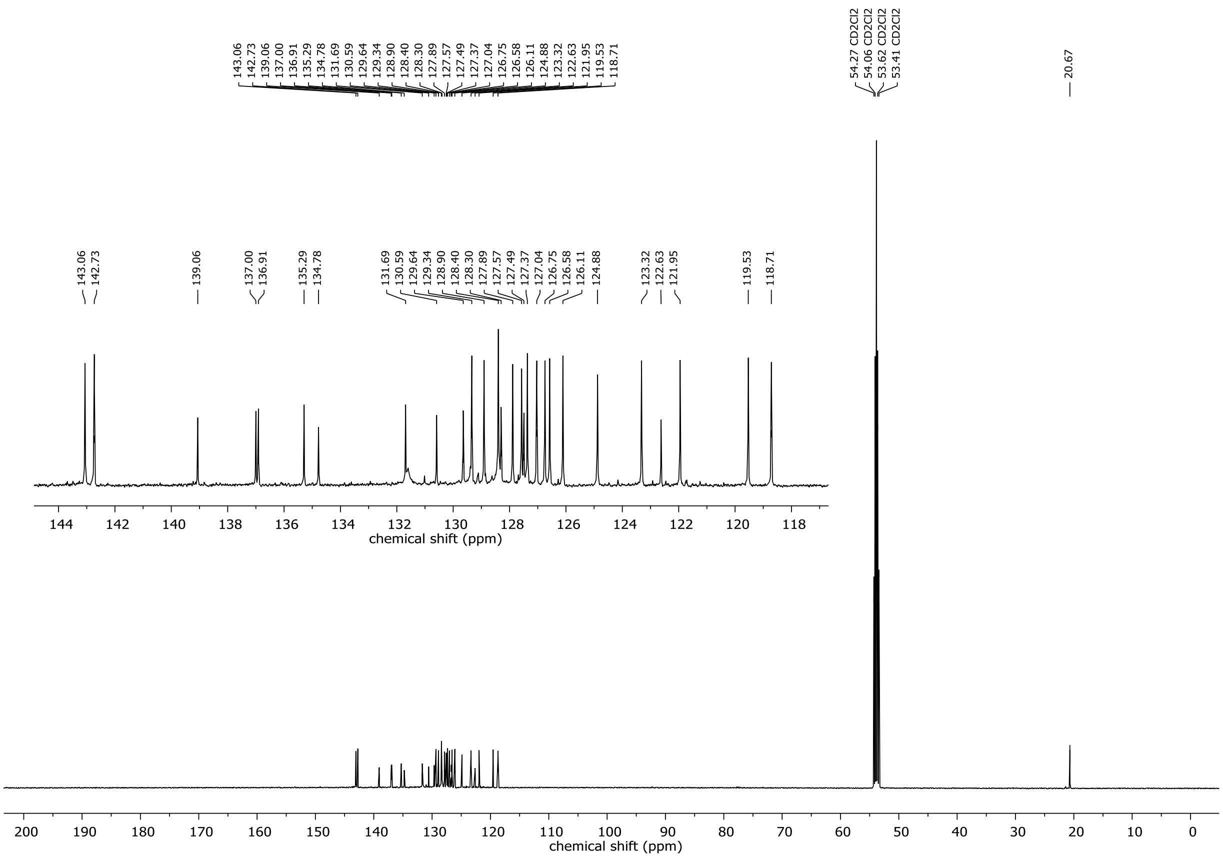

^{11}B NMR (161 MHz, $CDCl_3$)

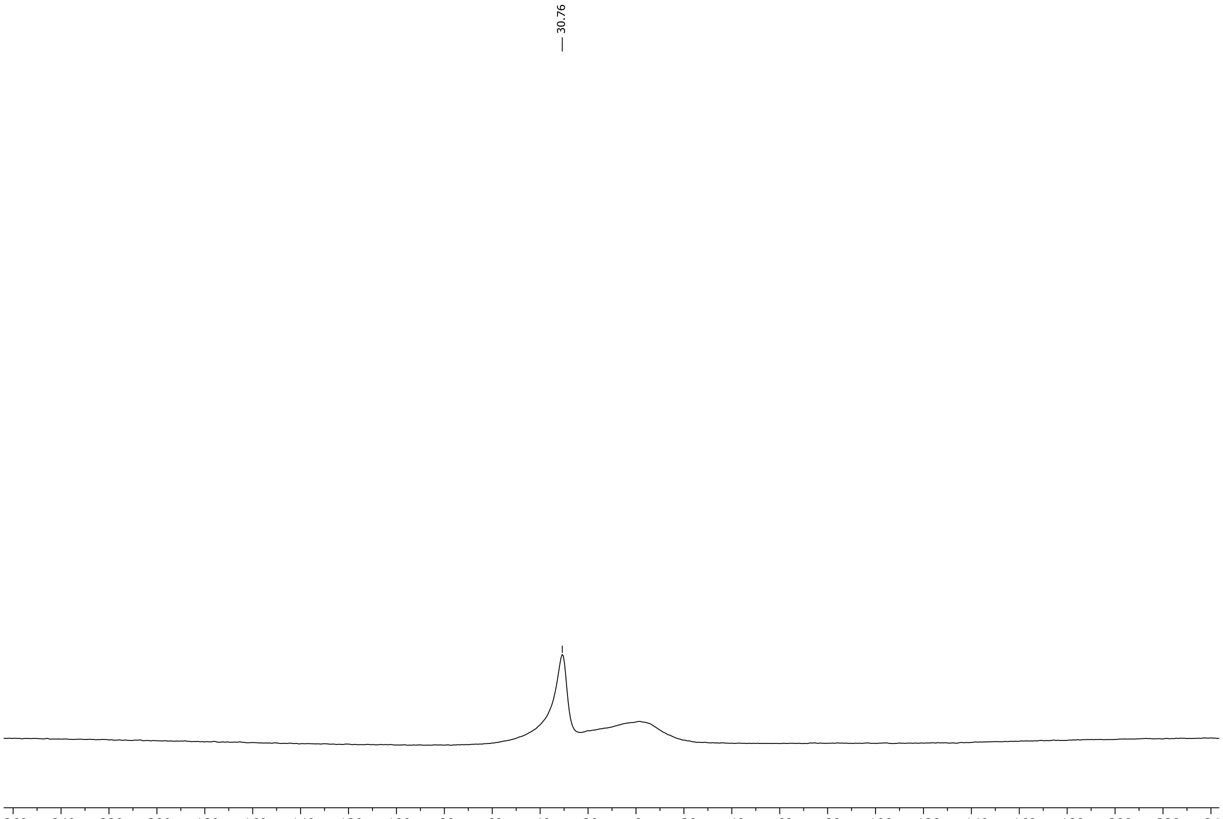

^{1}H NMR (600 MHz, $CDCl_3$, –35 °C) **2c**

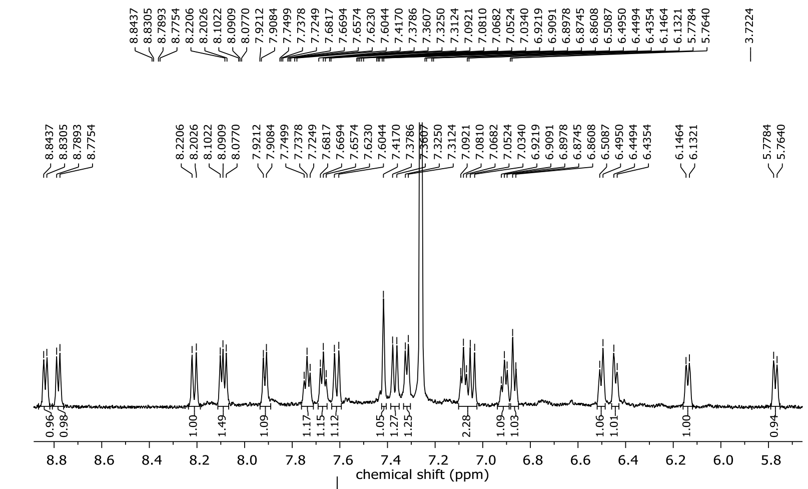

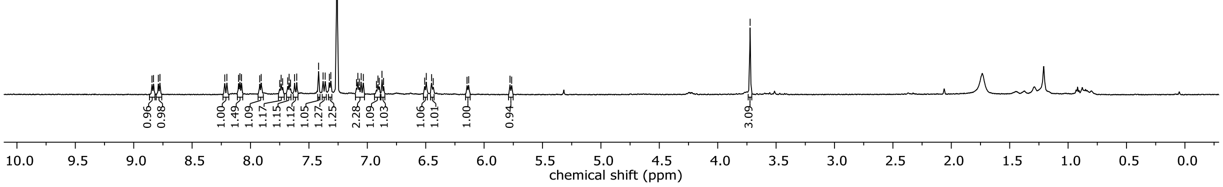

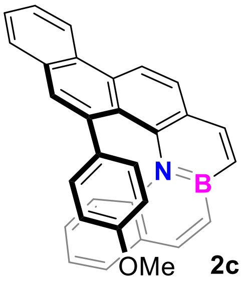

2c

^{13}C NMR (151 MHz, $CDCl_3$, –35 °C)

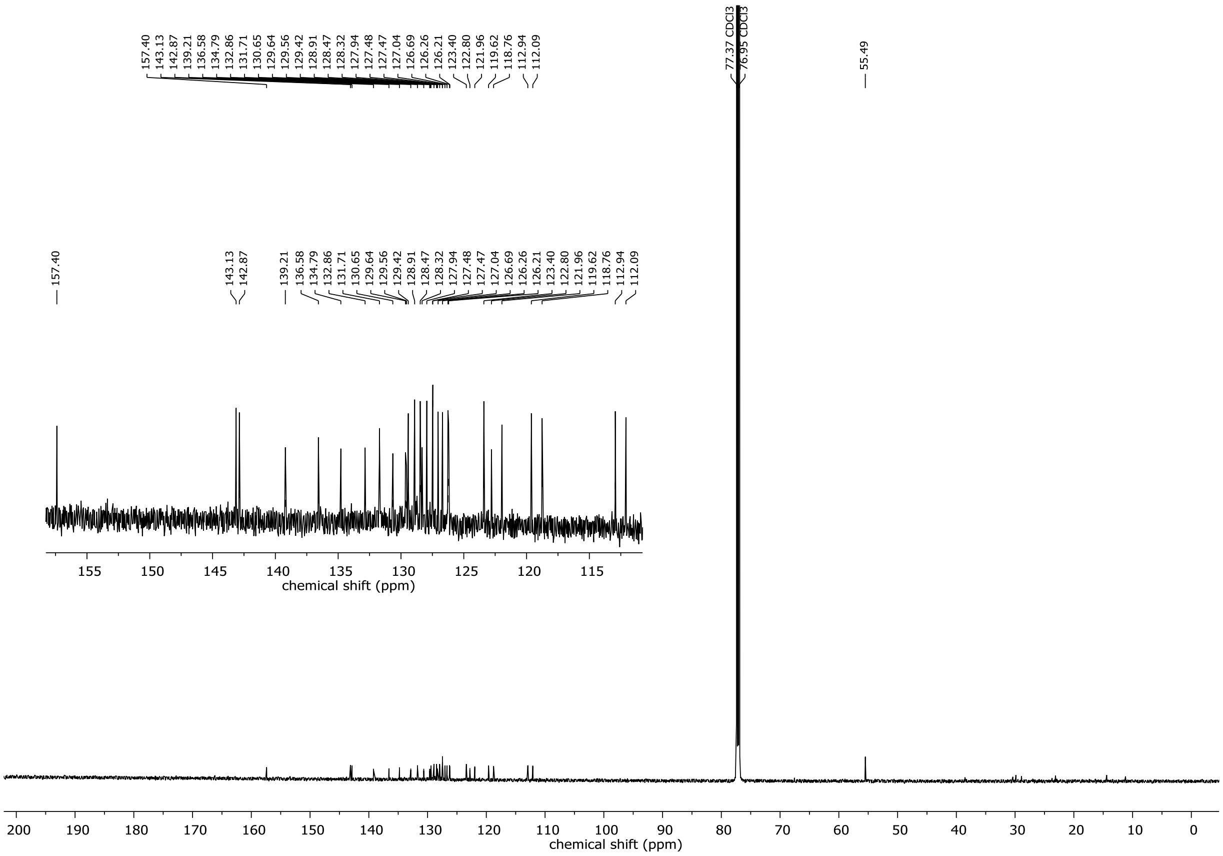

^{11}B NMR (128 MHz, $CDCl_3$)

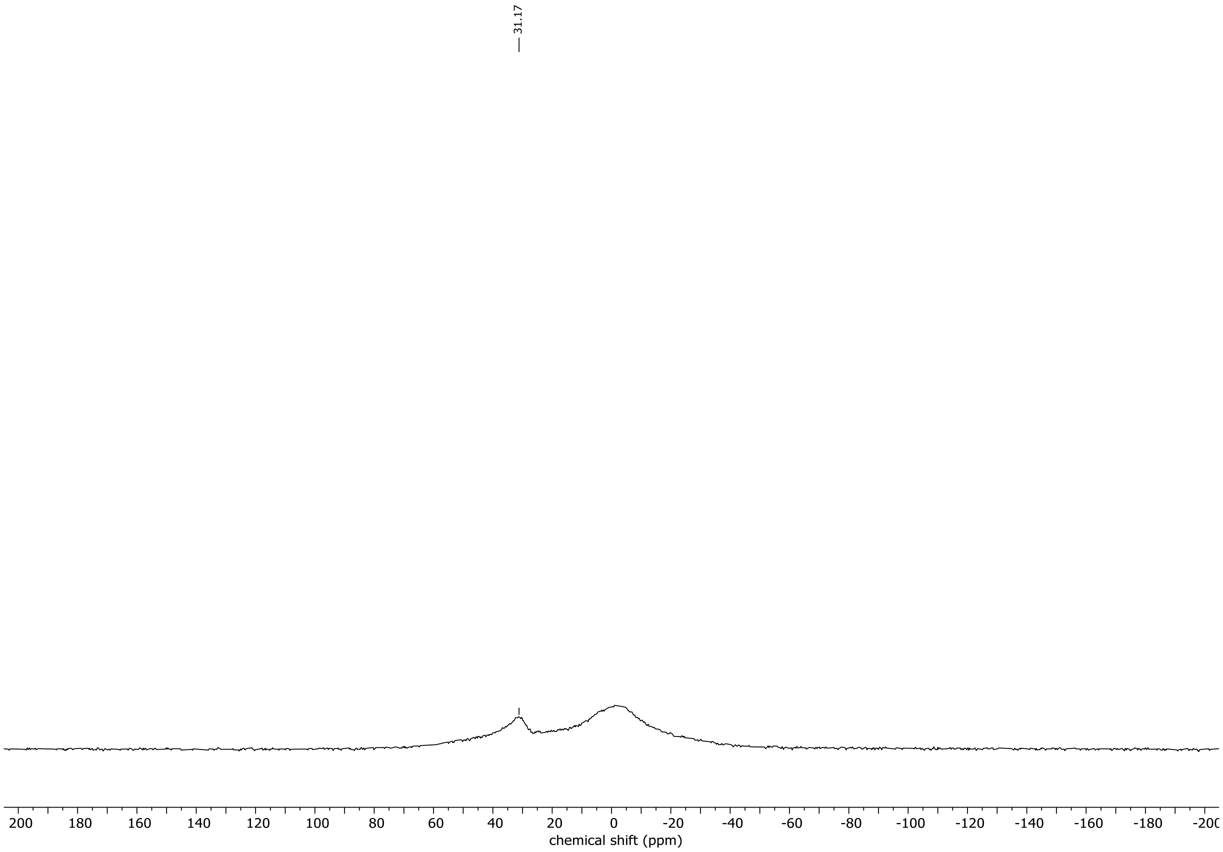

^{1}H NMR (500 MHz, CDCl$_3$, –35 °C) 2e

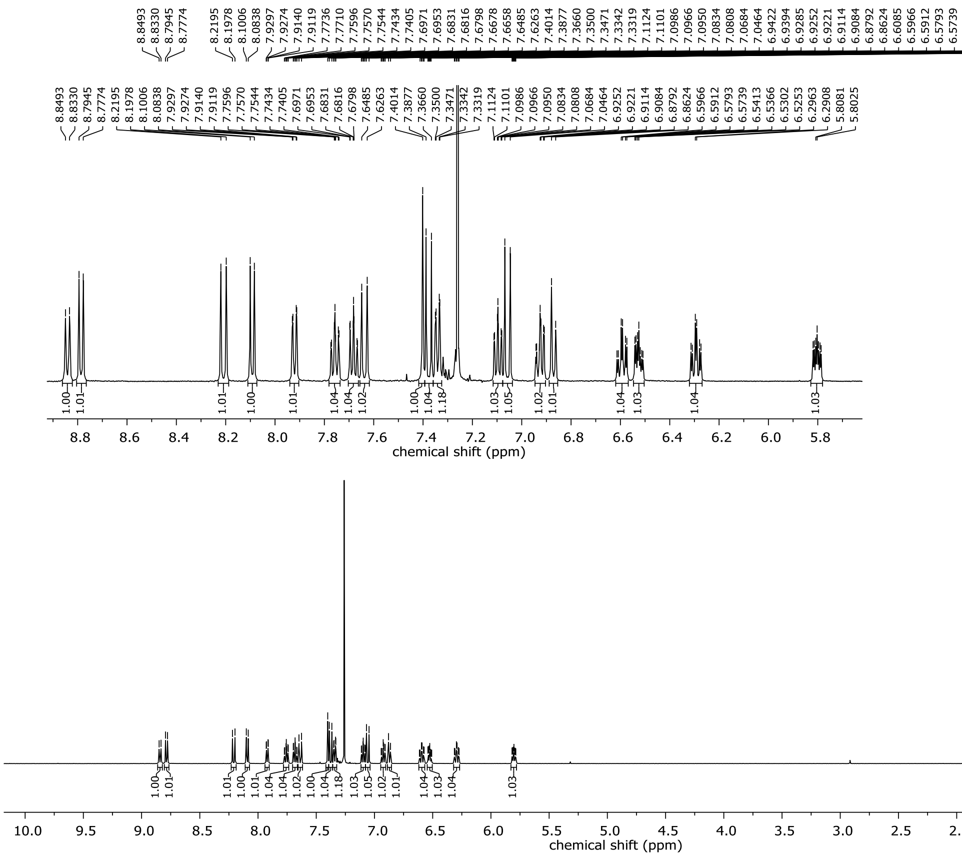

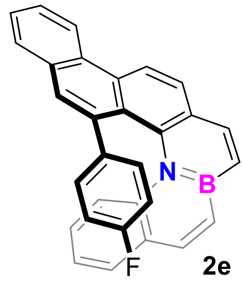

2e

^{1}H NMR (300 MHz, CDCl$_3$; rt)

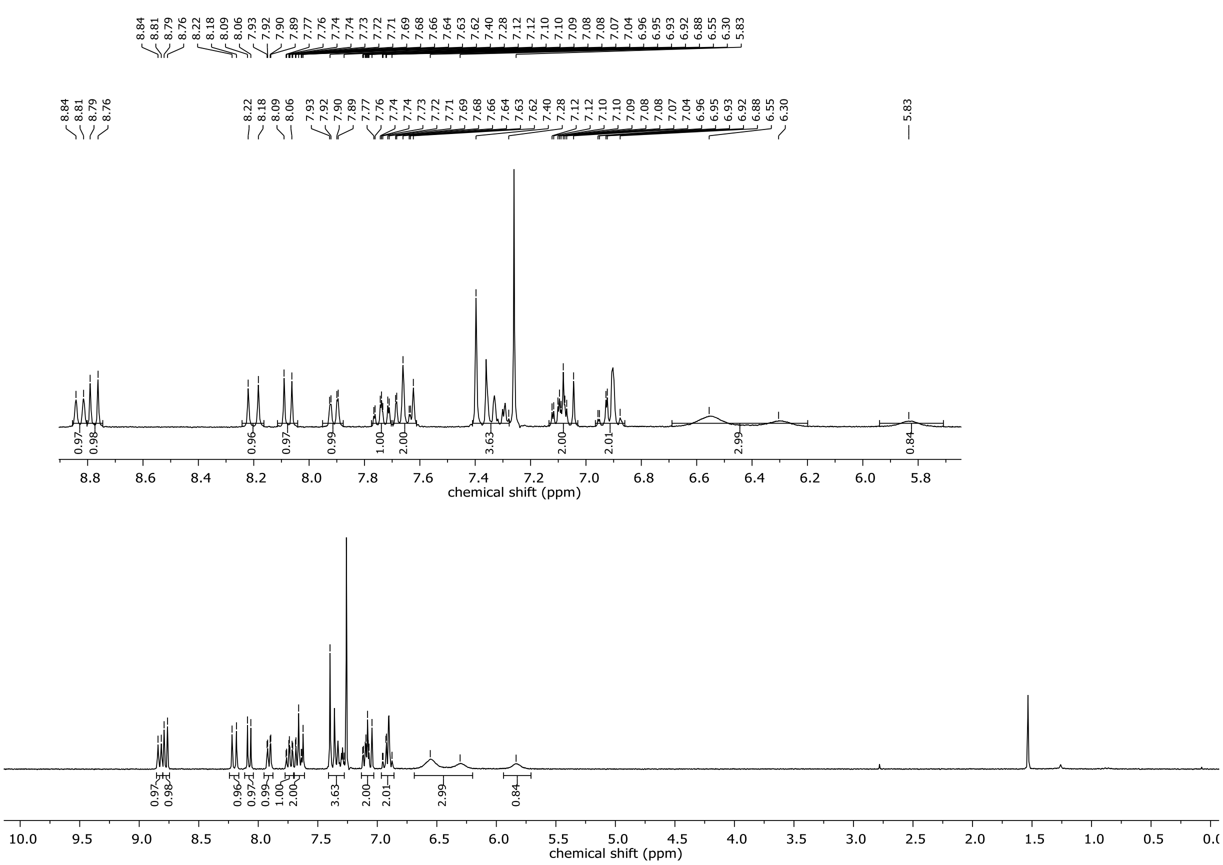

^{13}C NMR (126 MHz, $CDCl_3$, –35 °C)

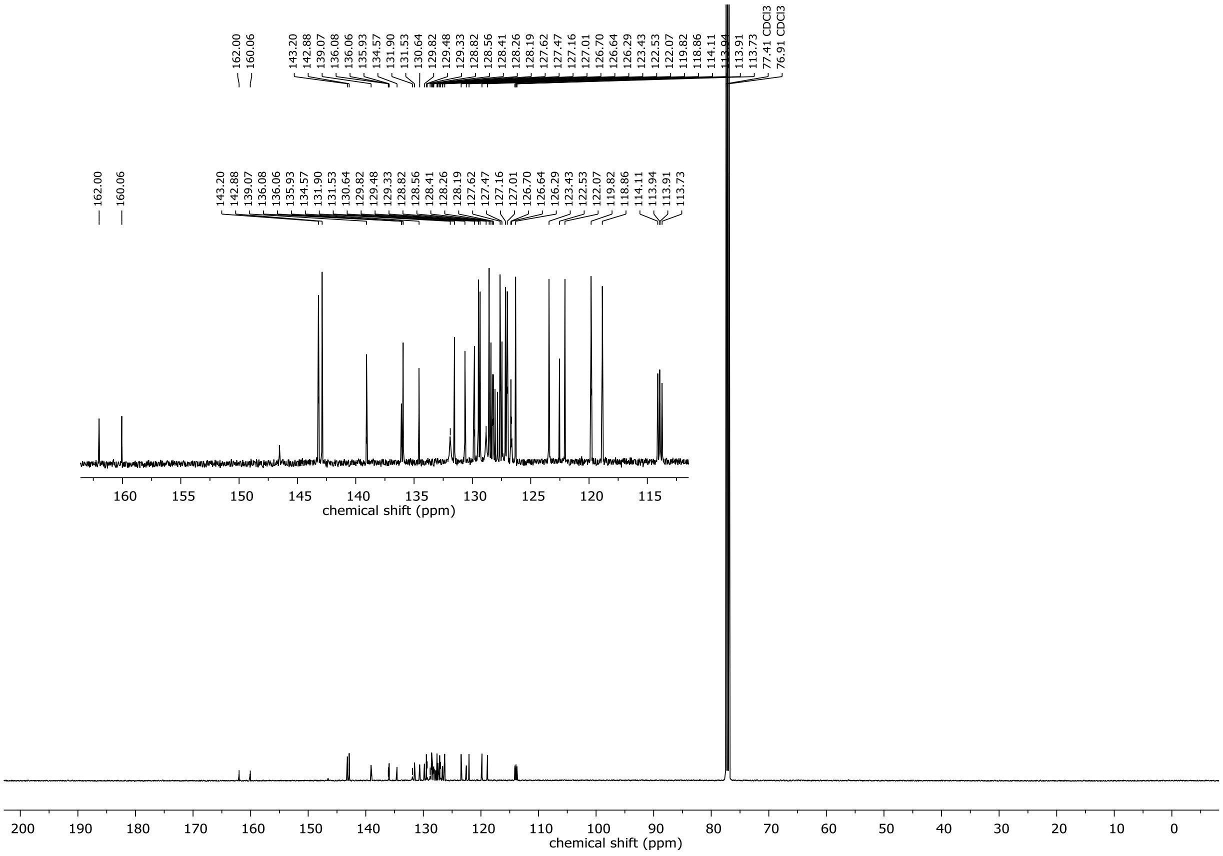

^{11}B NMR (161 MHz, $CDCl_3$)

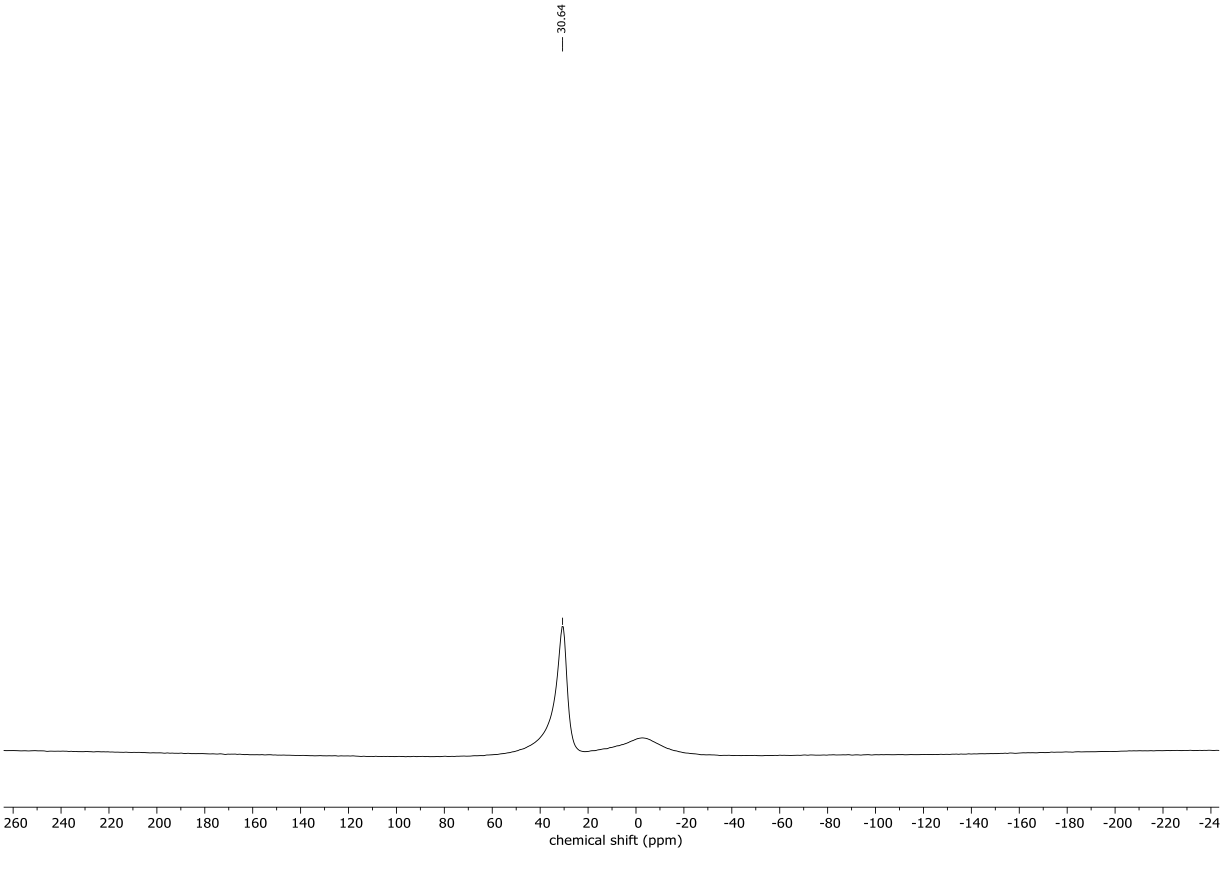

^{19}F NMR (282 MHz, $CDCl_3$)

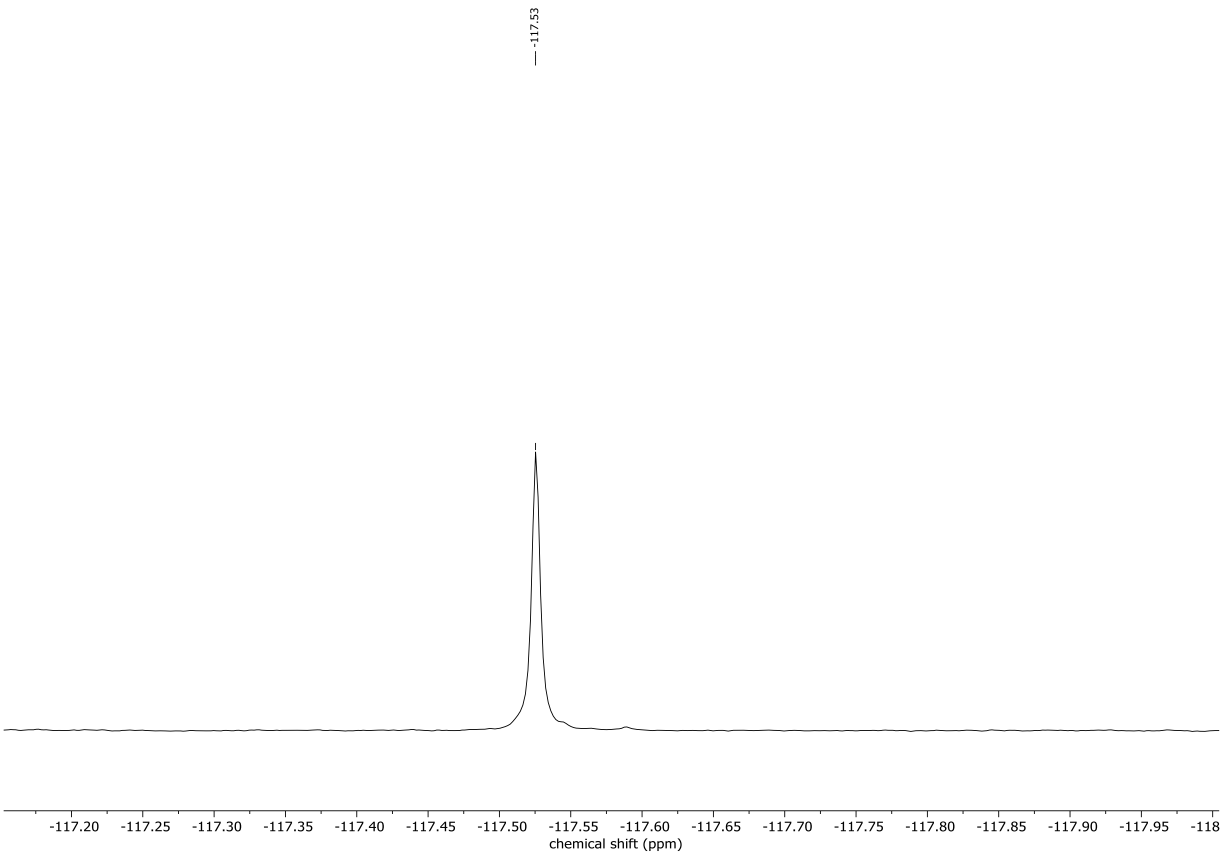

^{1}H NMR (500 MHz, $CDCl_3$, –35 °C) **2h**

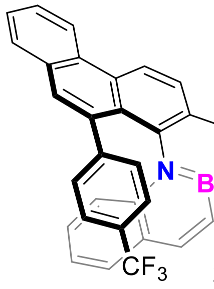

2h

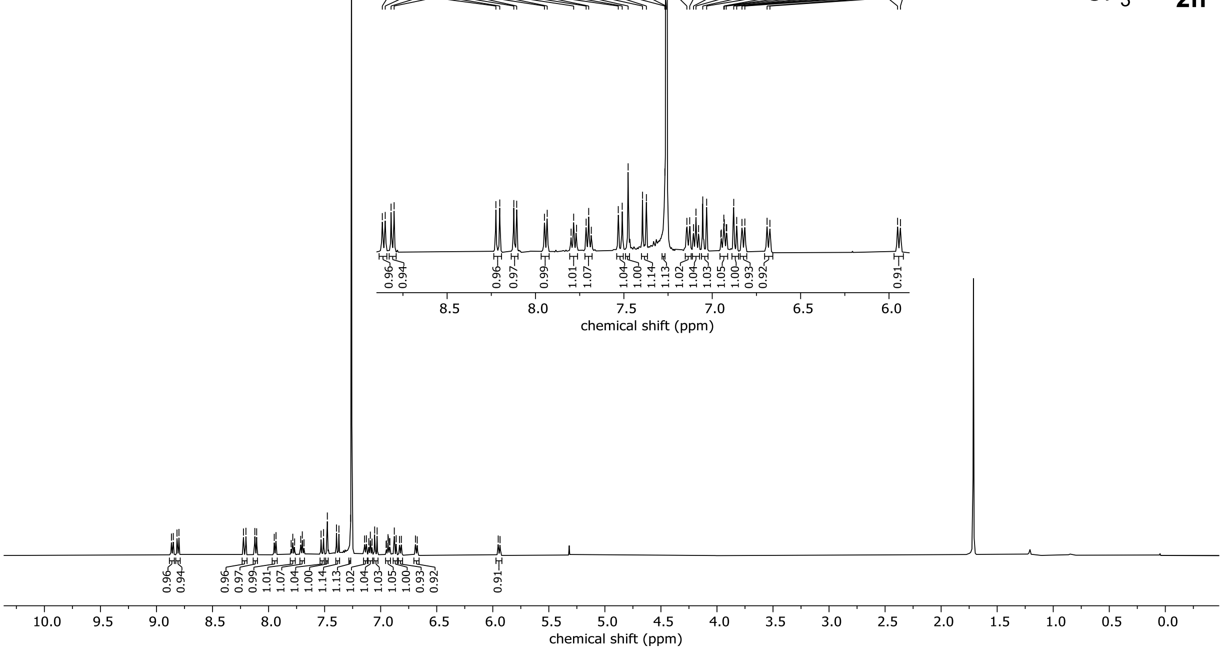

^{1}H NMR (300 MHz, $CDCl_3$; rt)

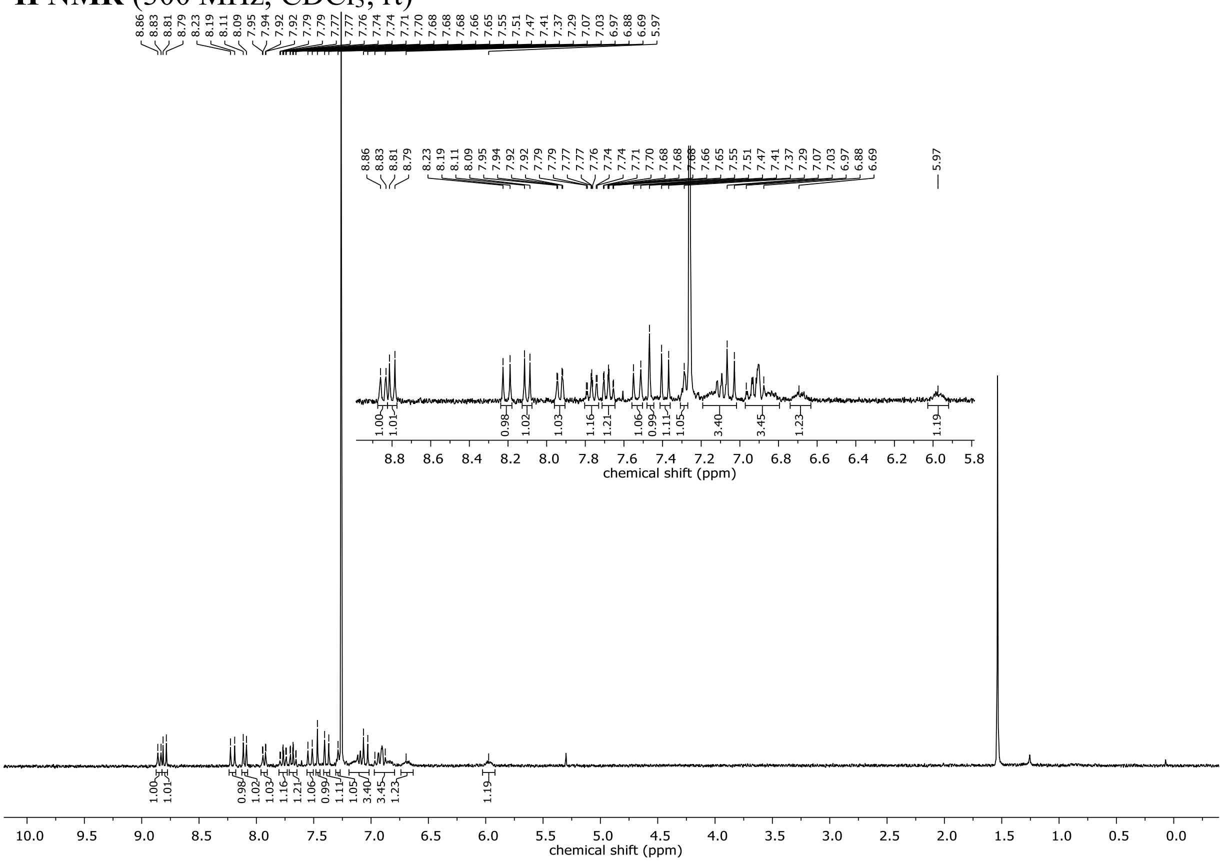

^{13}**C NMR** (126 MHz, $CDCl_3$, –35 °C)

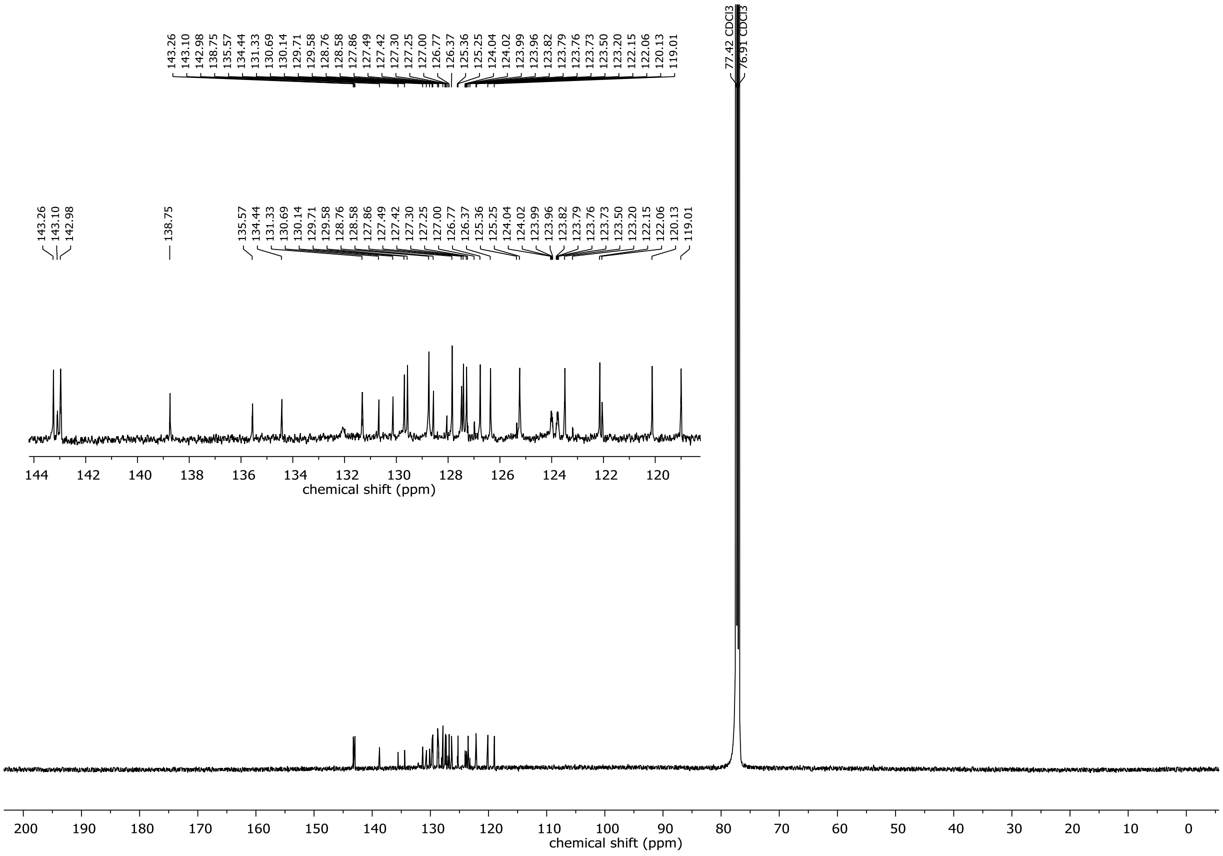

^{11}**B NMR** (161 MHz, $CDCl_3$)

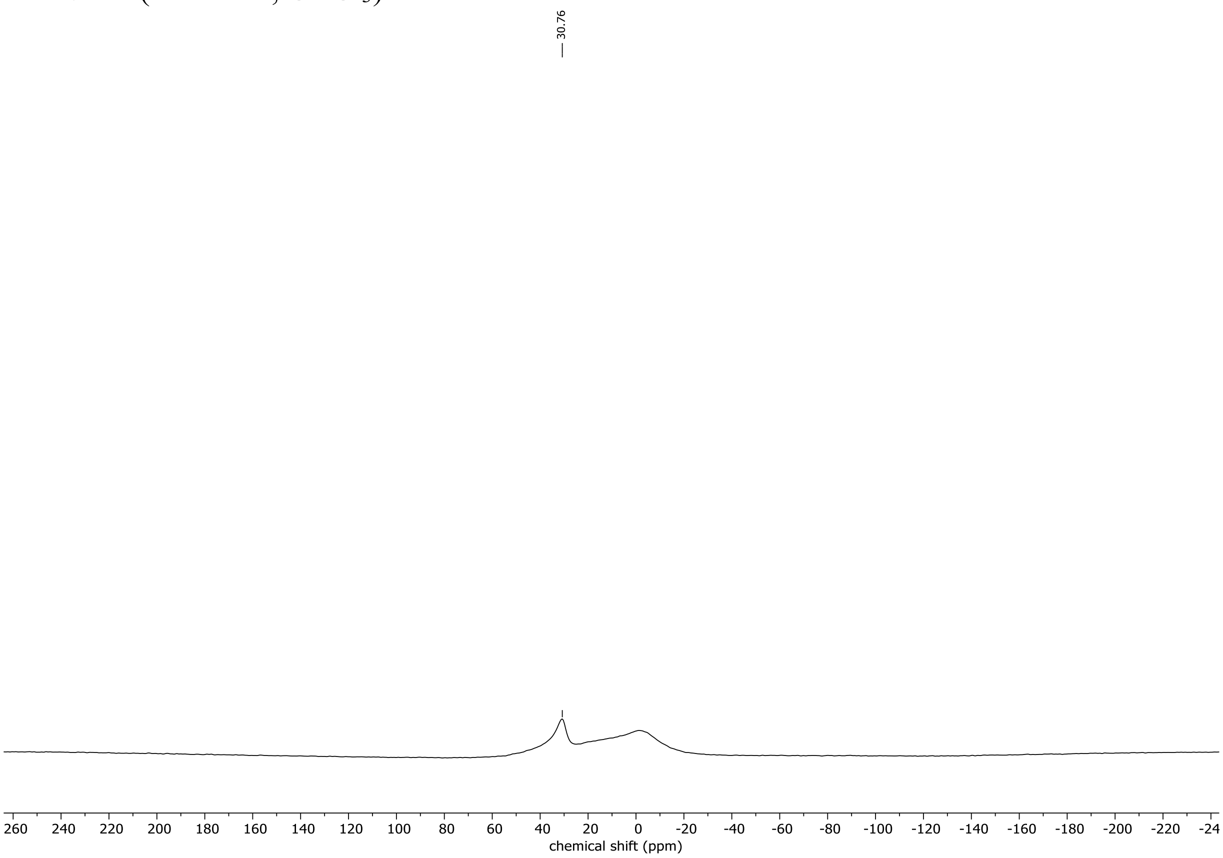

^{19}F NMR (282 MHz, $CDCl_3$)

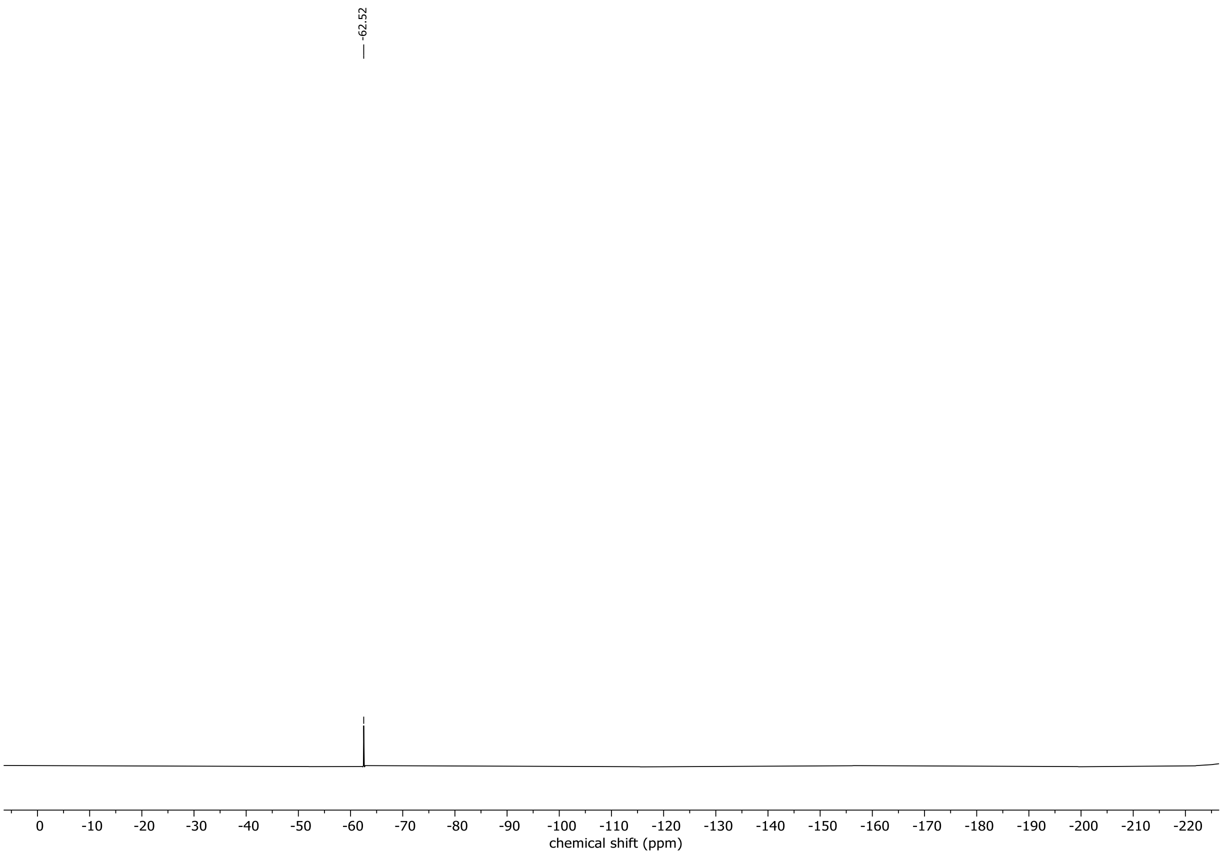

^{1}H NMR (300 MHz, $CDCl_3$) **S10a**

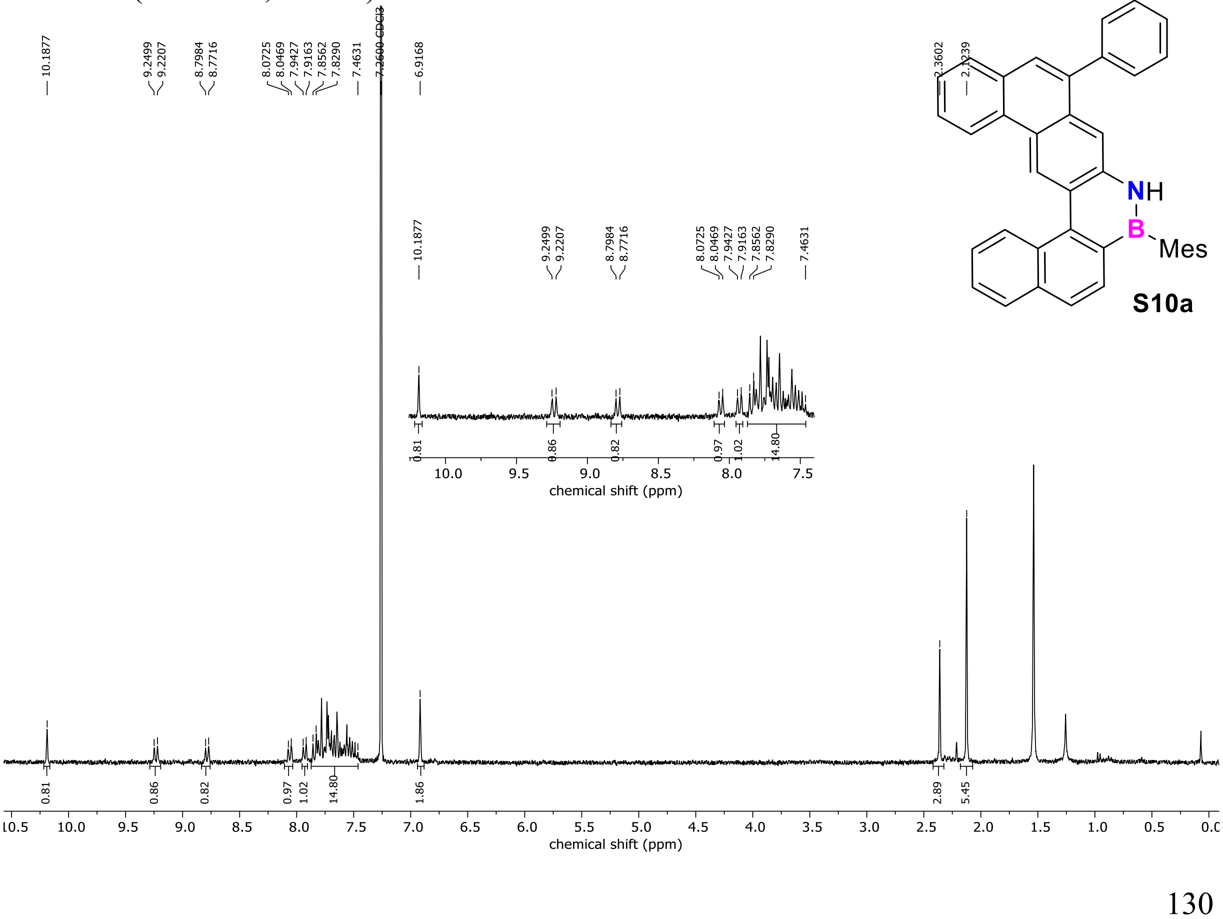

^{13}C NMR (126 MHz, $CDCl_3$)

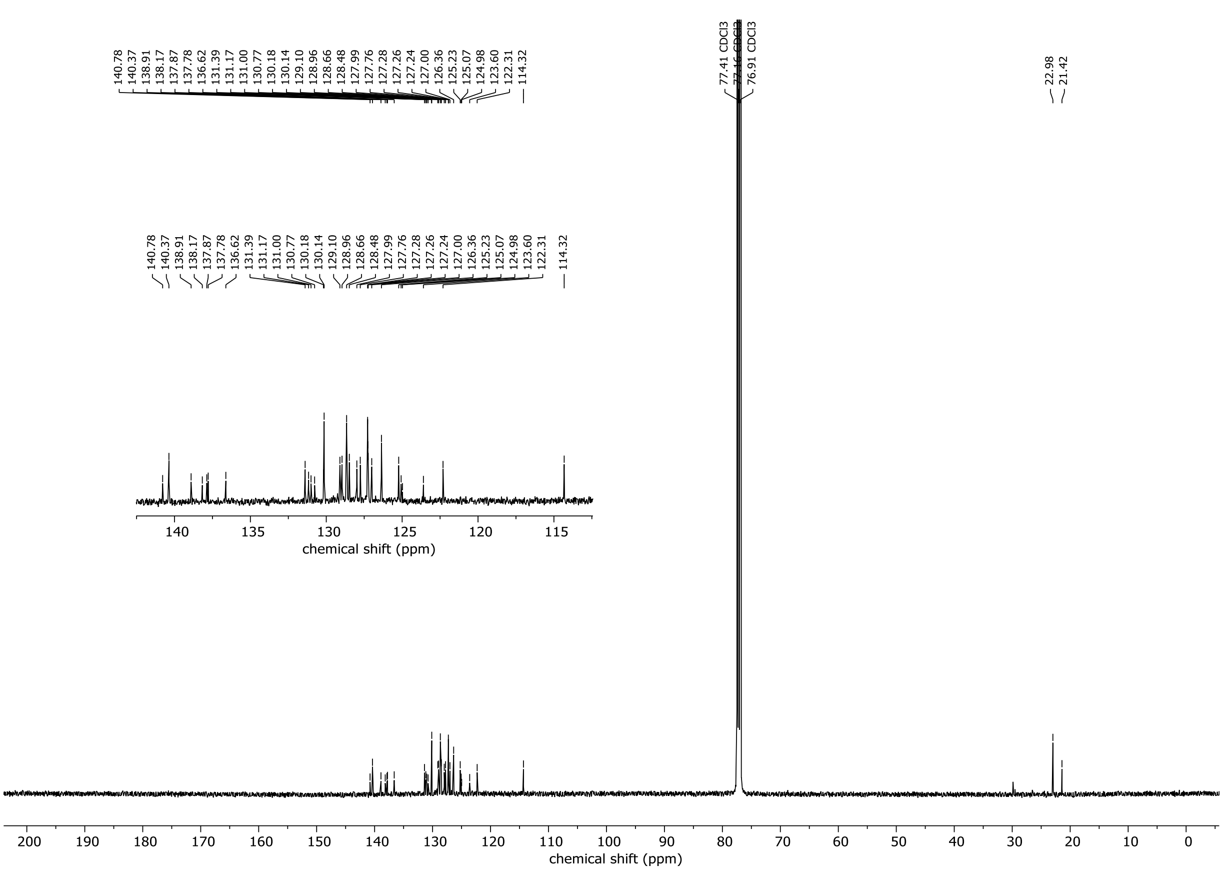

^{11}B NMR (161 MHz, $CDCl_3$)

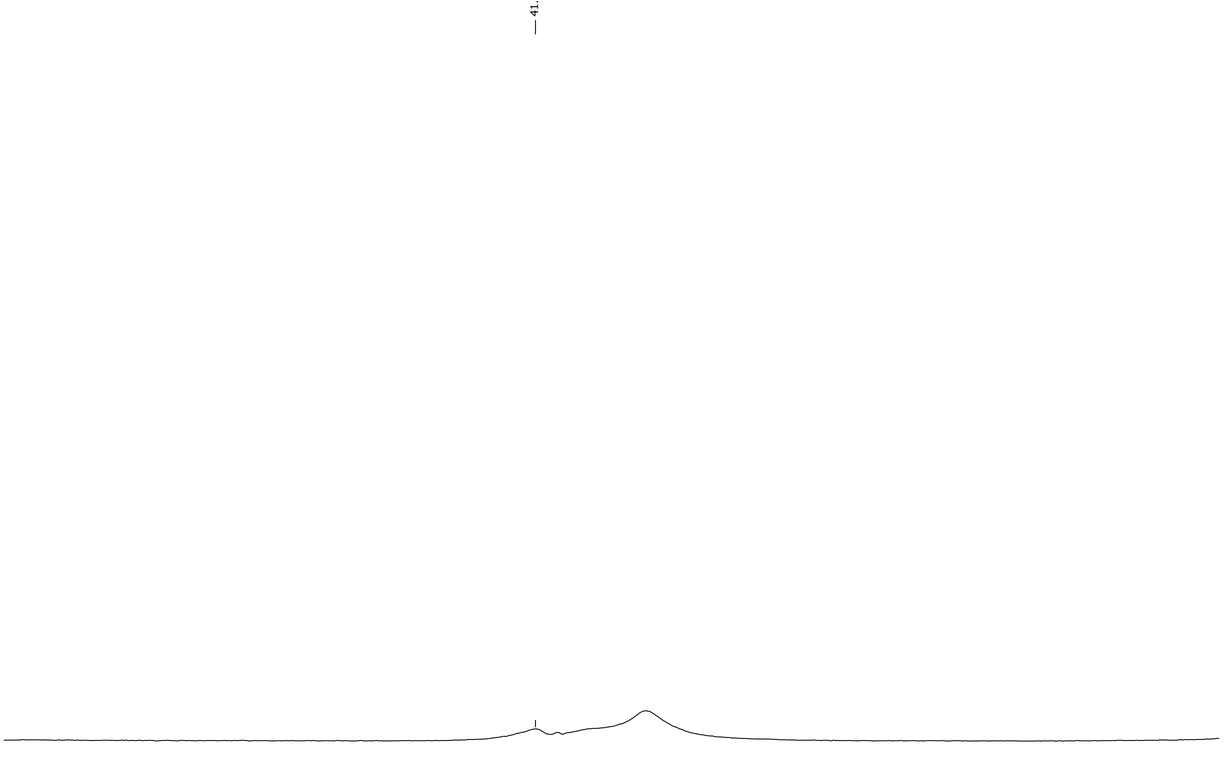

^{1}H NMR (400 MHz, CD_2Cl_2) **10b**

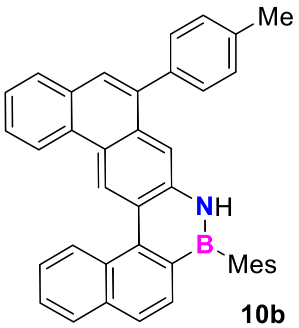

10b

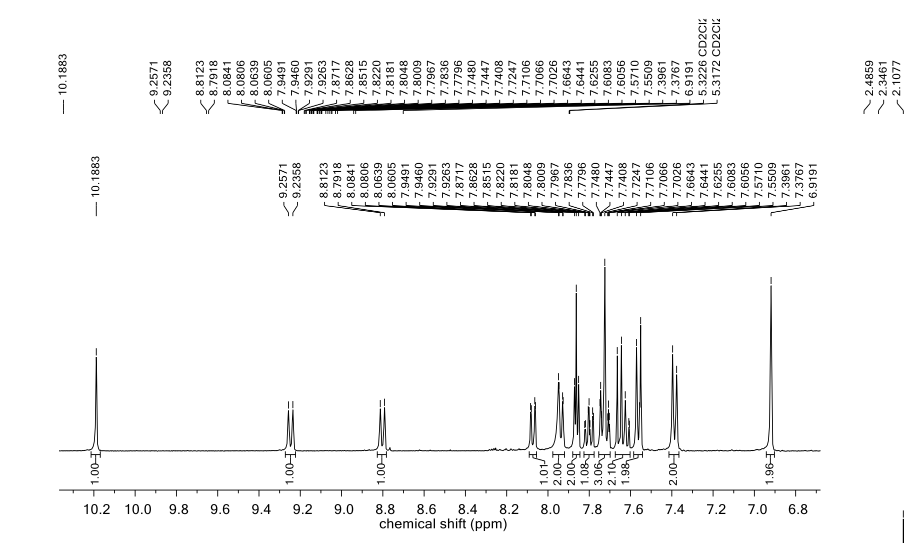
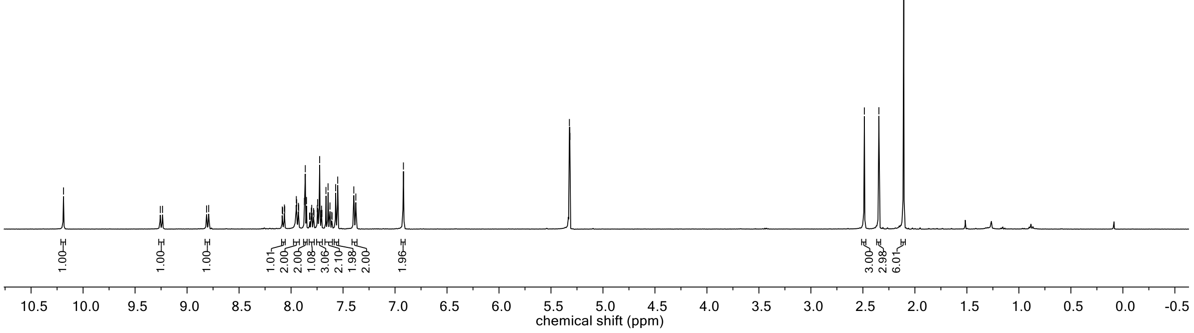

^{13}C NMR (101 MHz, CD_2Cl_2)

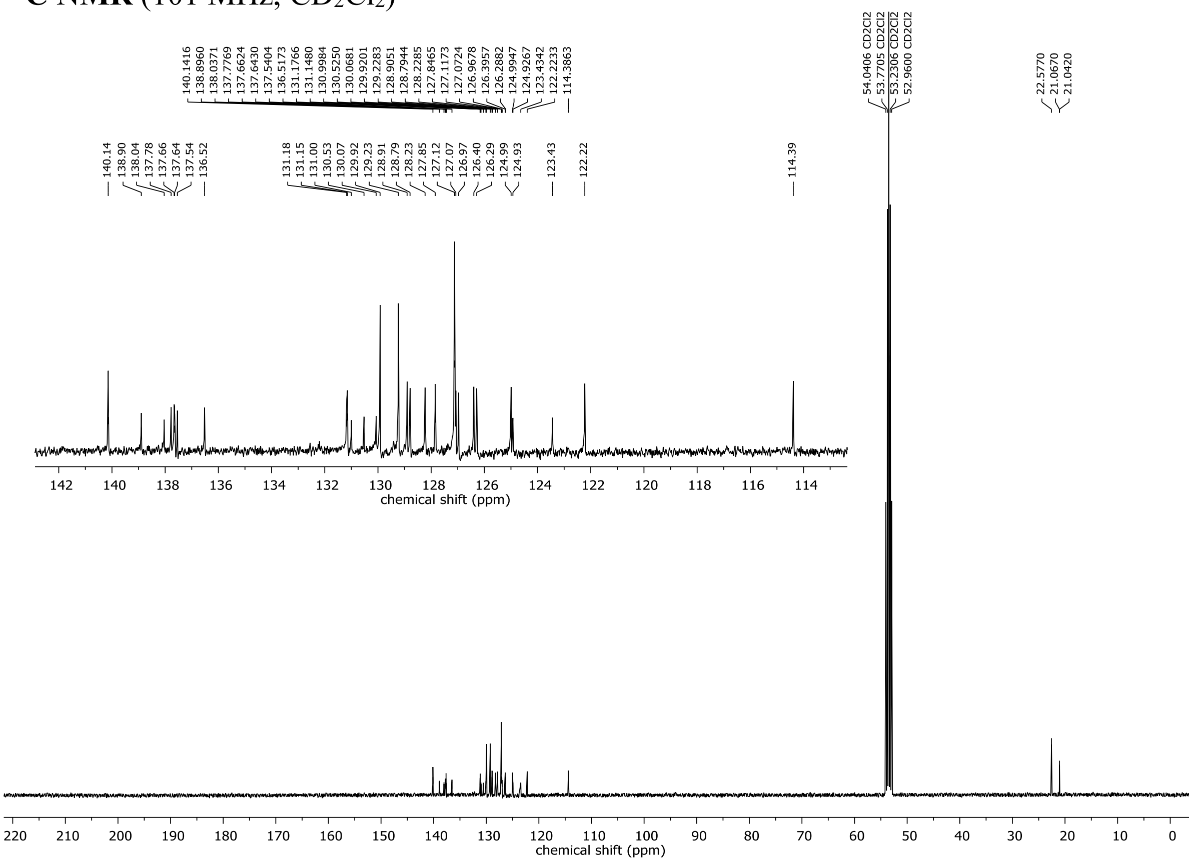

^{11}B NMR (161 MHz, CD_2Cl_2)

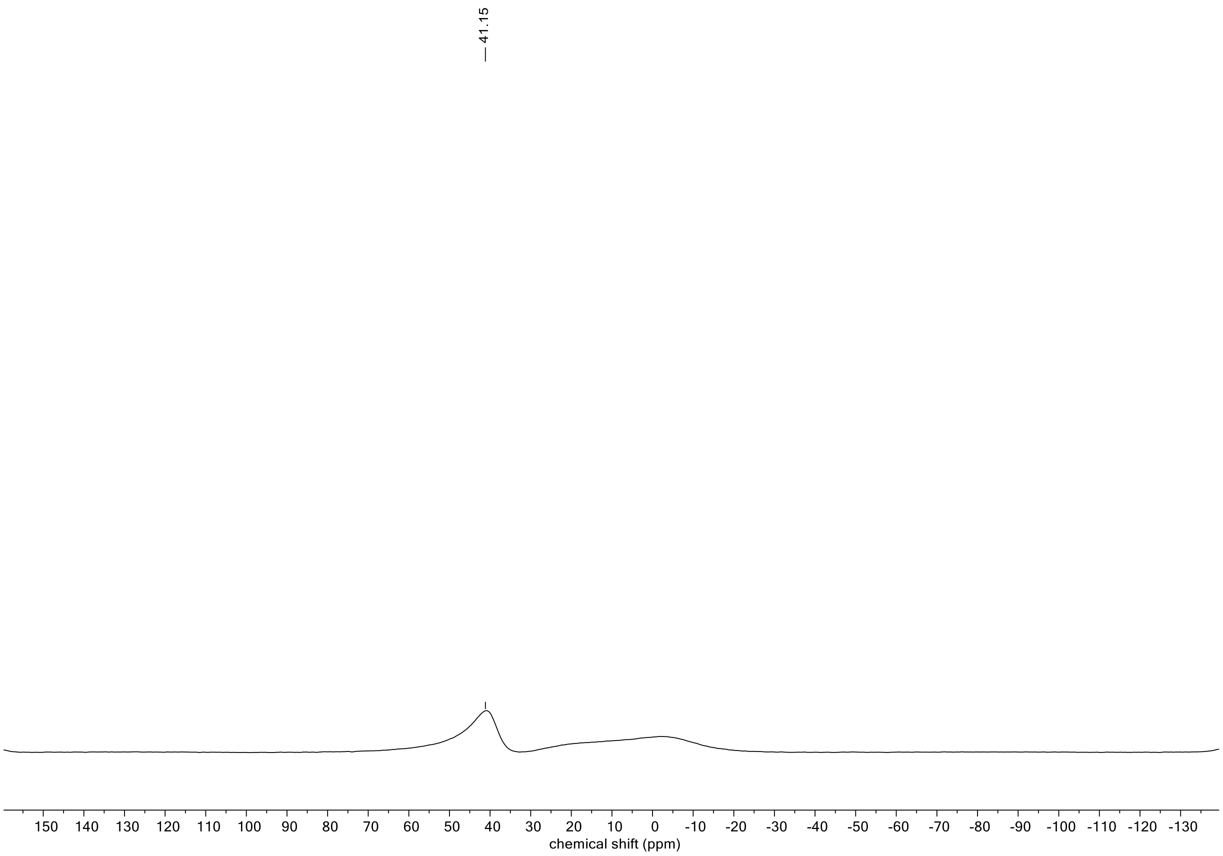

^{1}H NMR (400 MHz, CD_2Cl_2) S10c

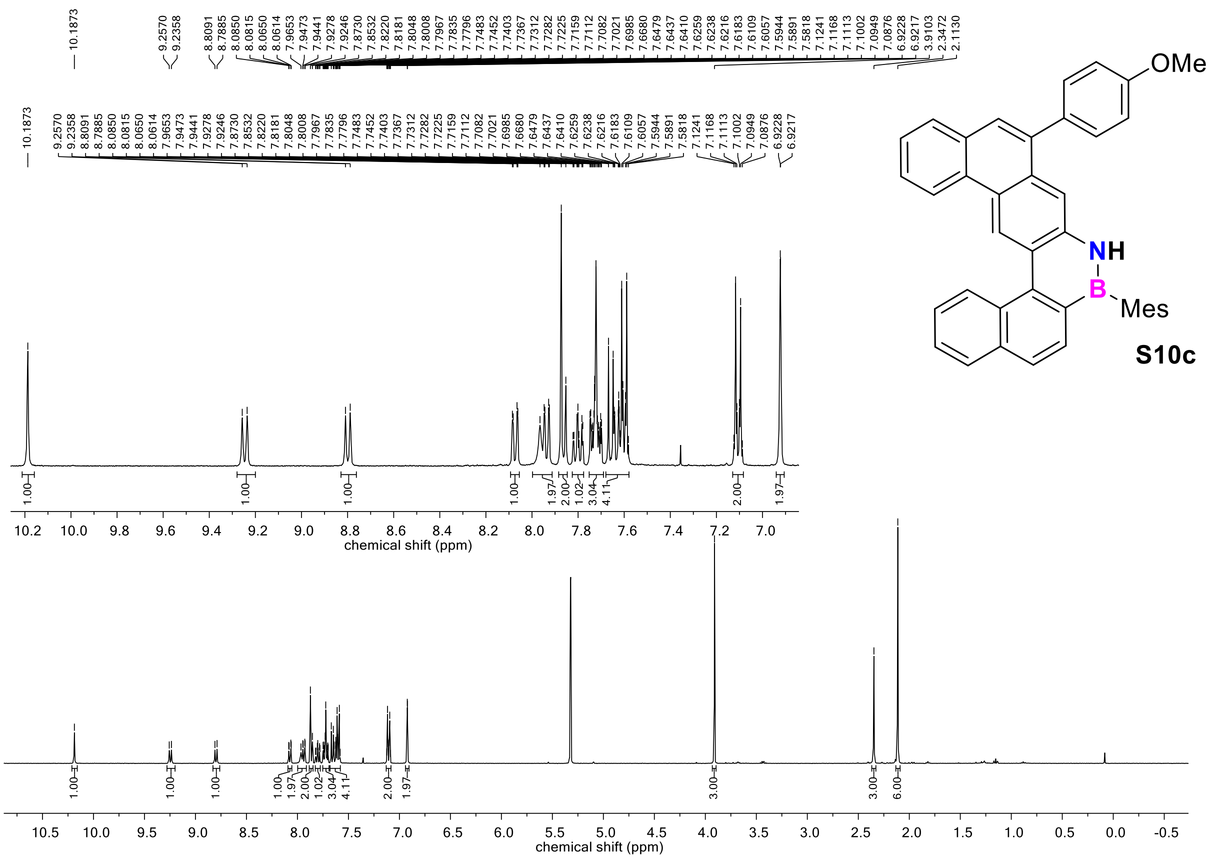

S10c

^{13}C NMR (101 MHz, CD_2Cl_2)

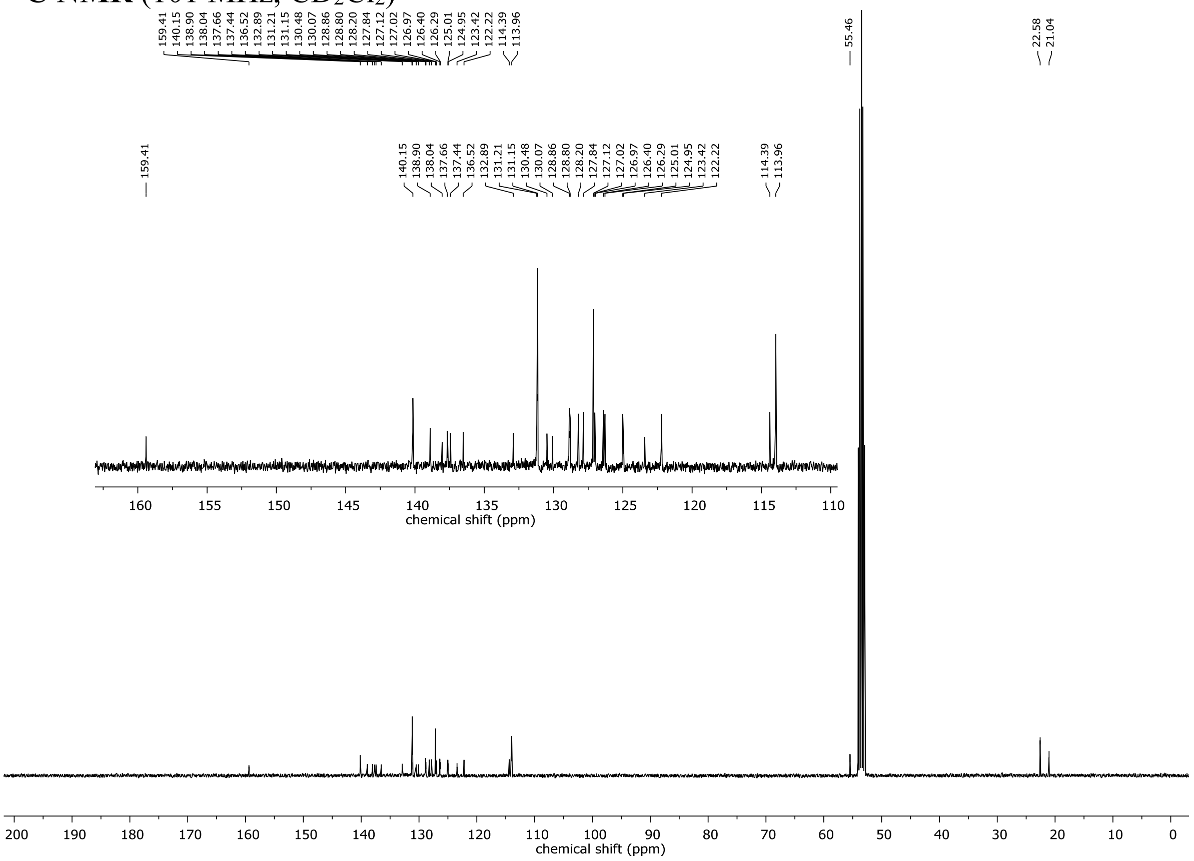

^{11}B NMR (161 MHz, CD_2Cl_2)

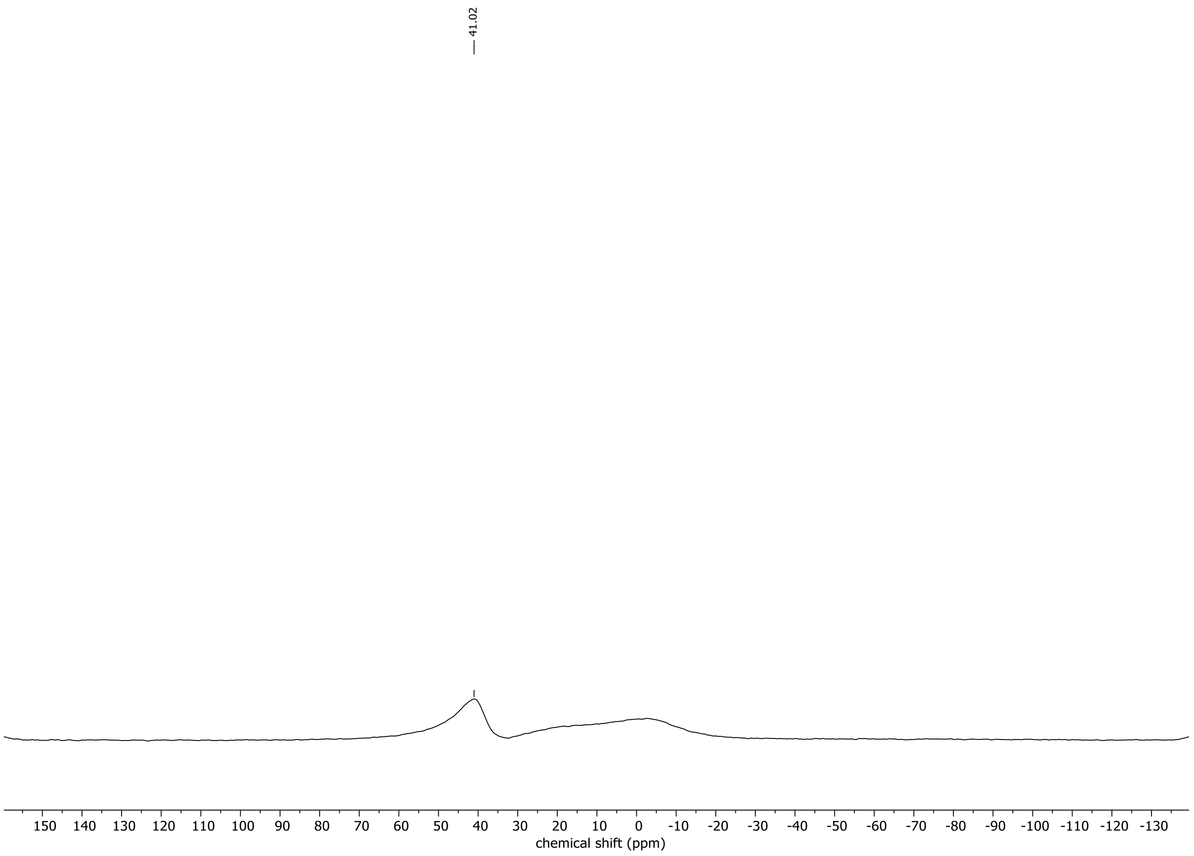

^{1}H NMR (400 MHz, CD_2Cl_2) **S10d**

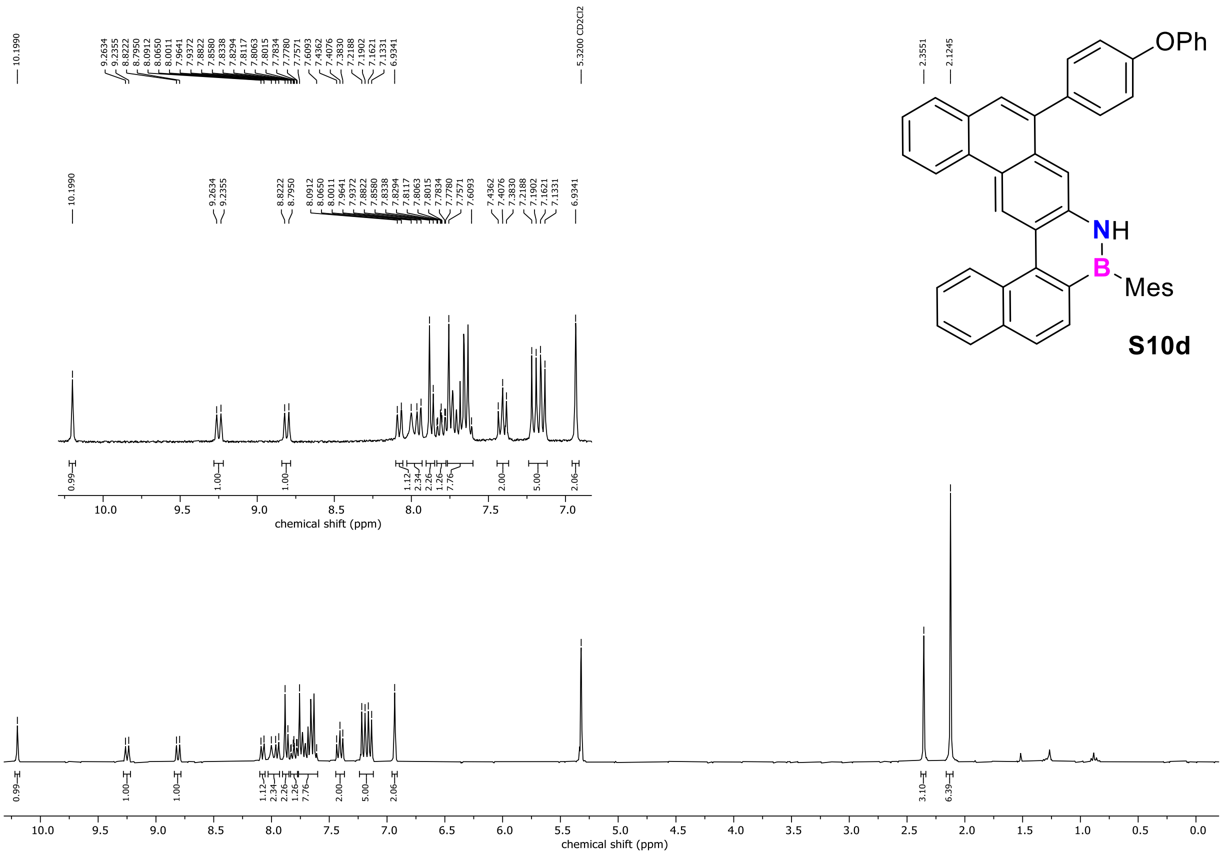

^{13}C NMR (101 MHz, CD_2Cl_2)

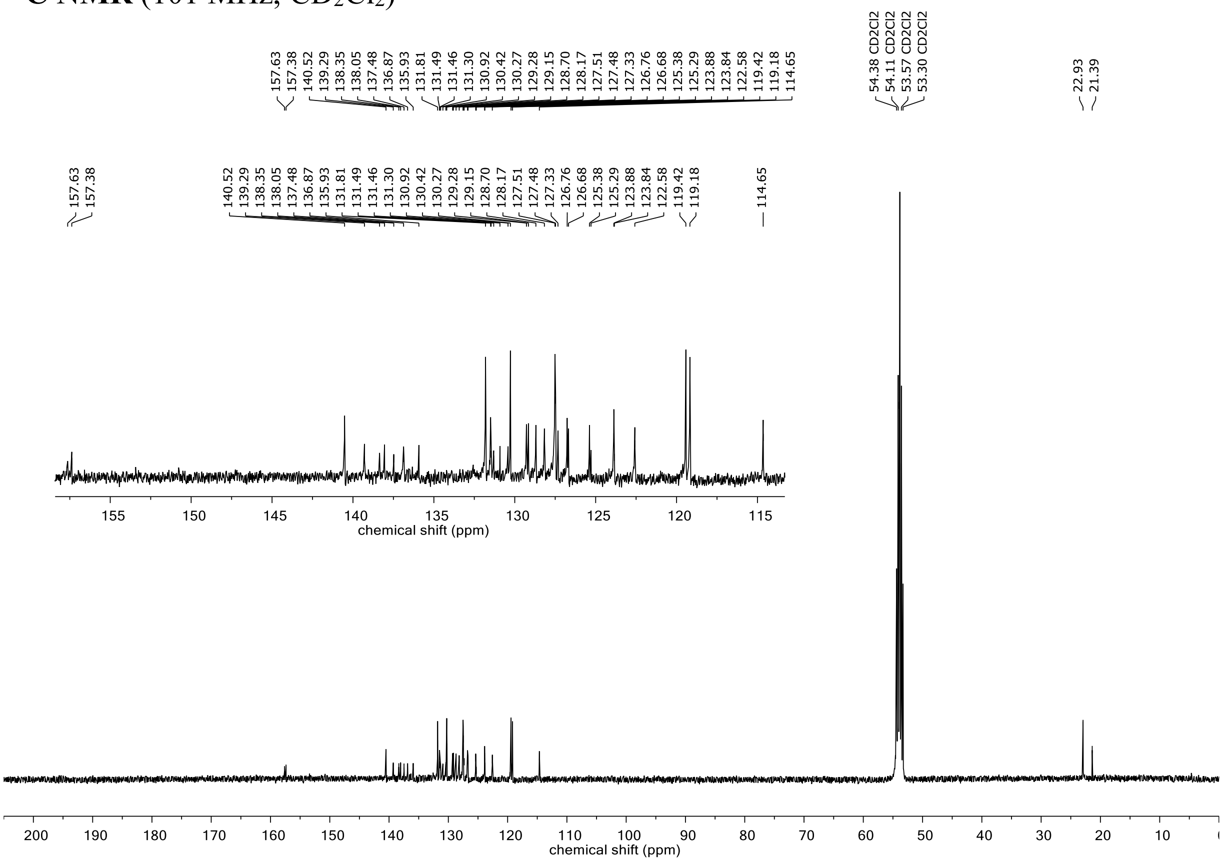

^{11}B NMR (161 MHz, CD_2Cl_2)

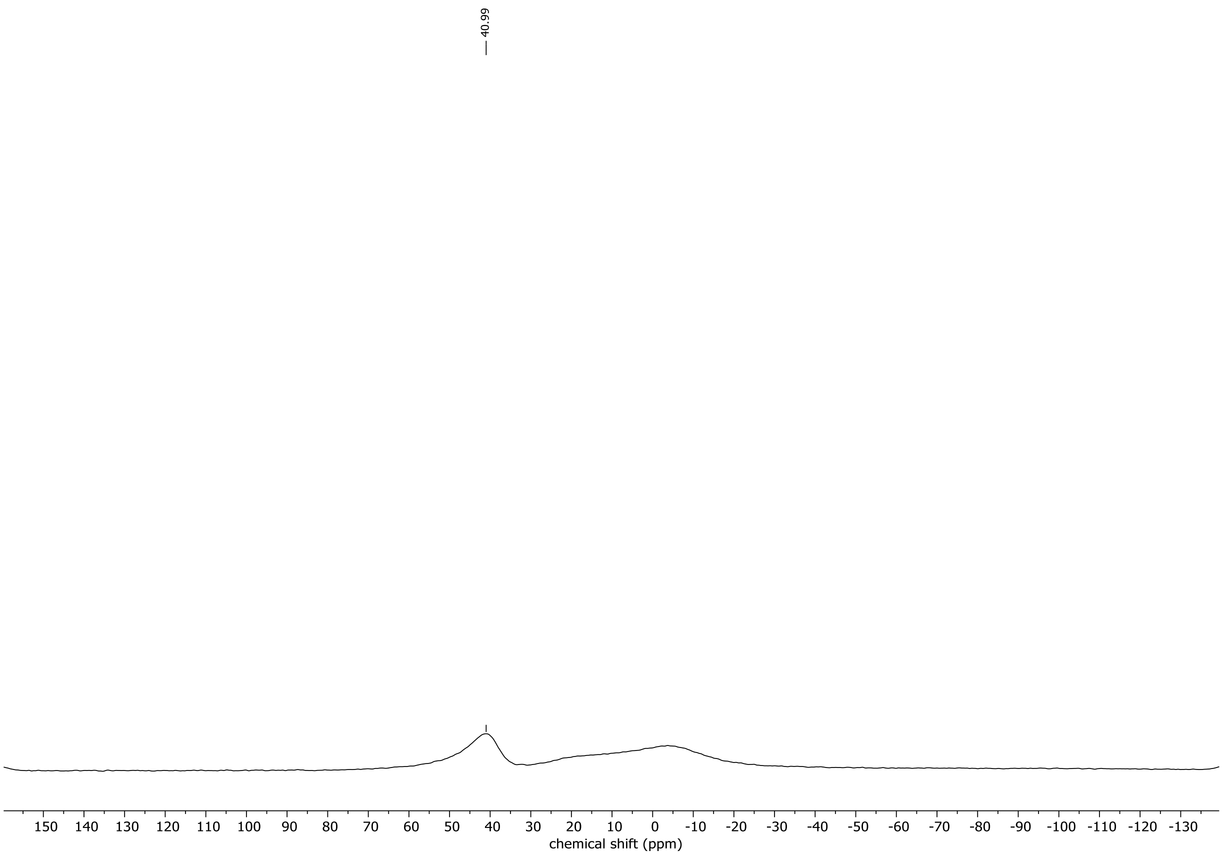

^{1}H NMR (500 MHz, CD_2Cl_2) **S10e**

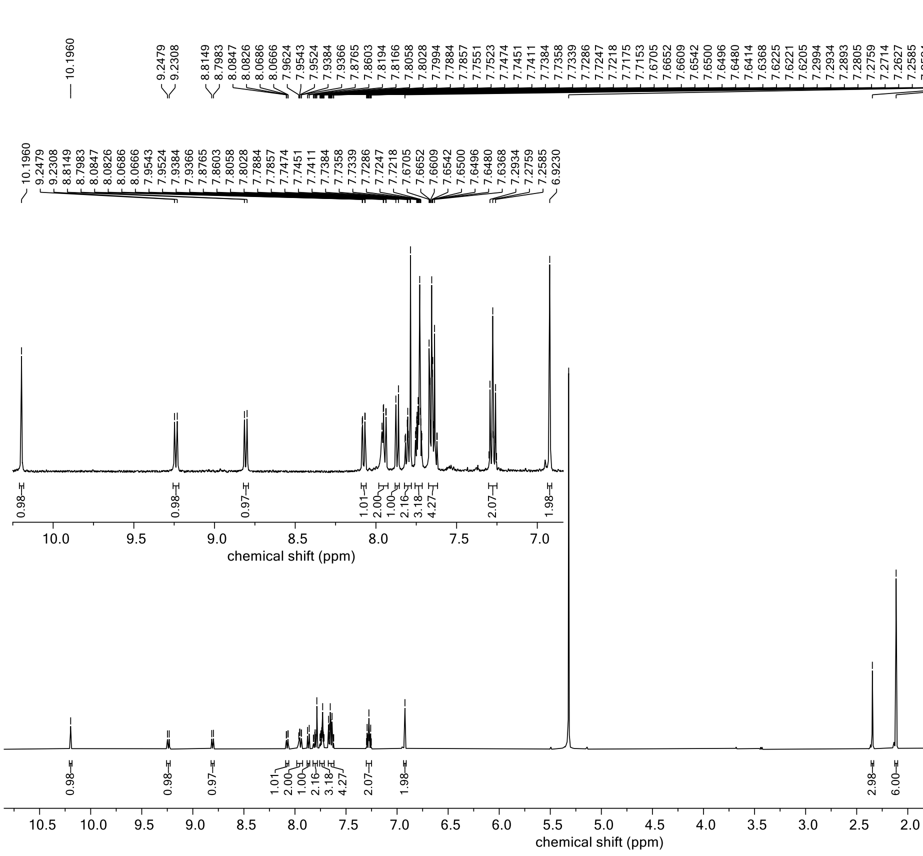

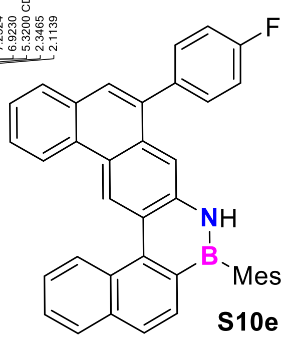
S10e

^{13}C NMR (126 MHz, CD_2Cl_2)

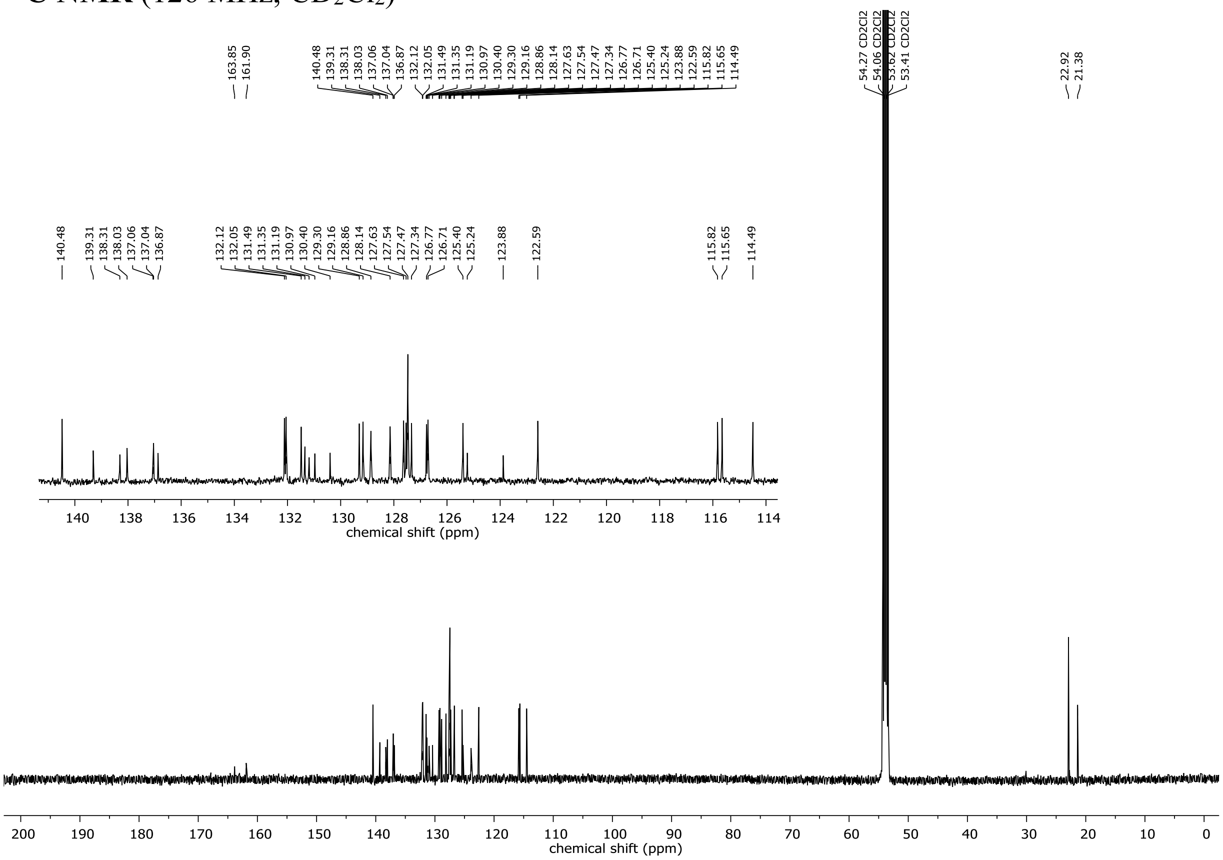

^{11}B NMR (161 MHz, CD_2Cl_2)

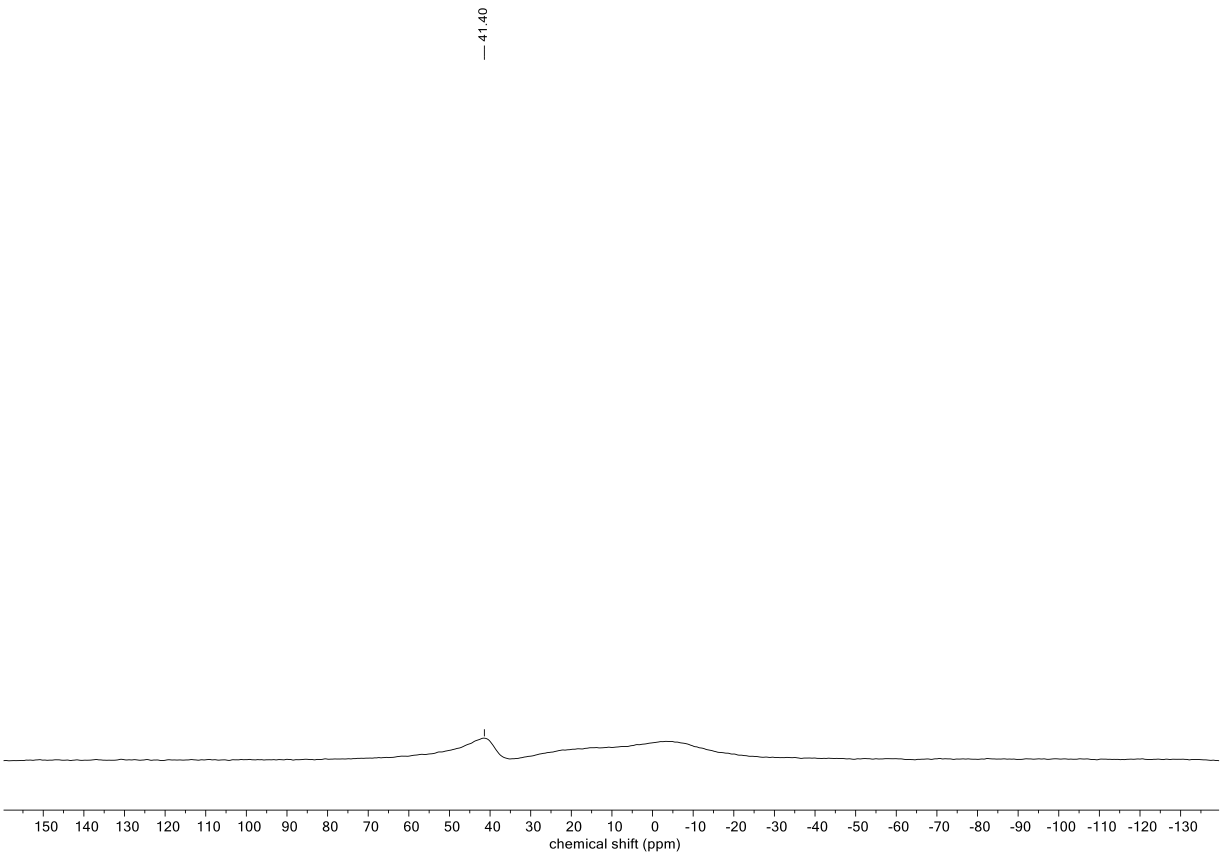

^{19}F NMR (282 MHz, CD_2Cl_2)

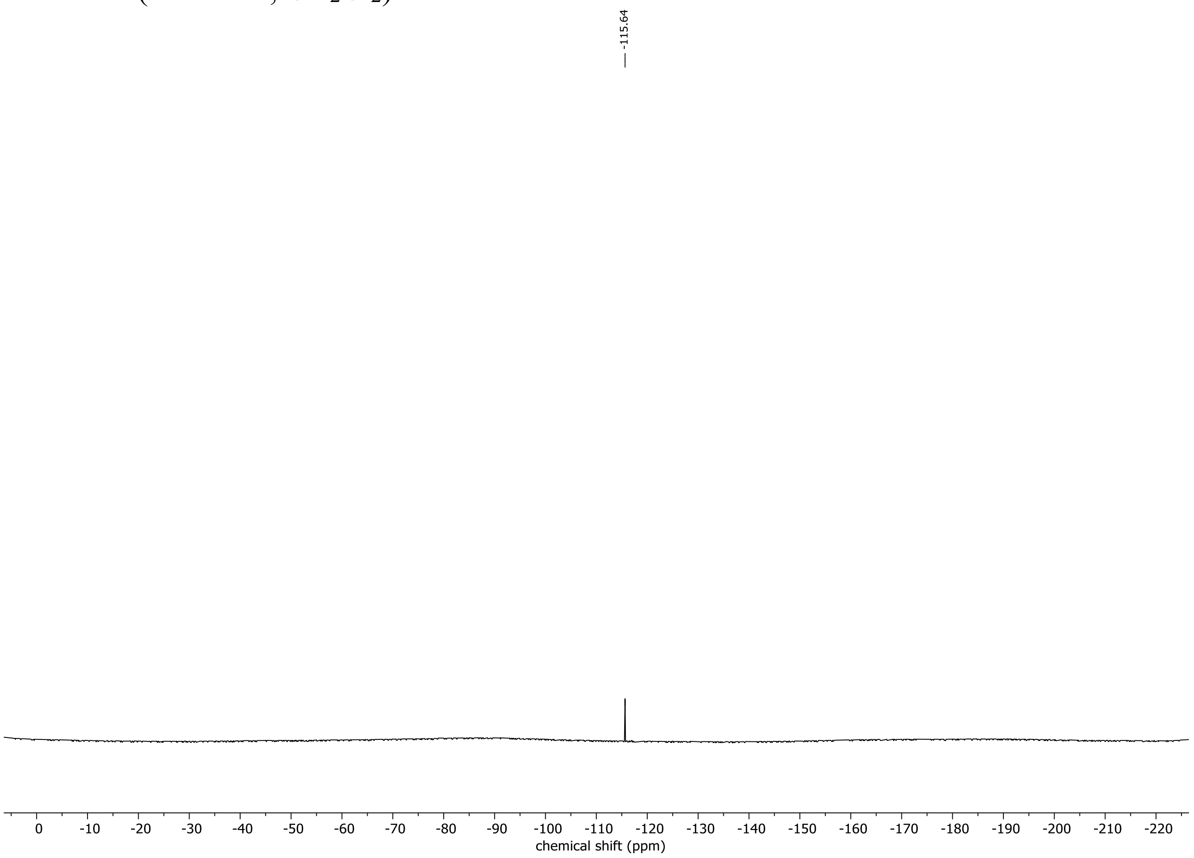

^{1}H NMR (300 MHz, CD_2Cl_2) S10i

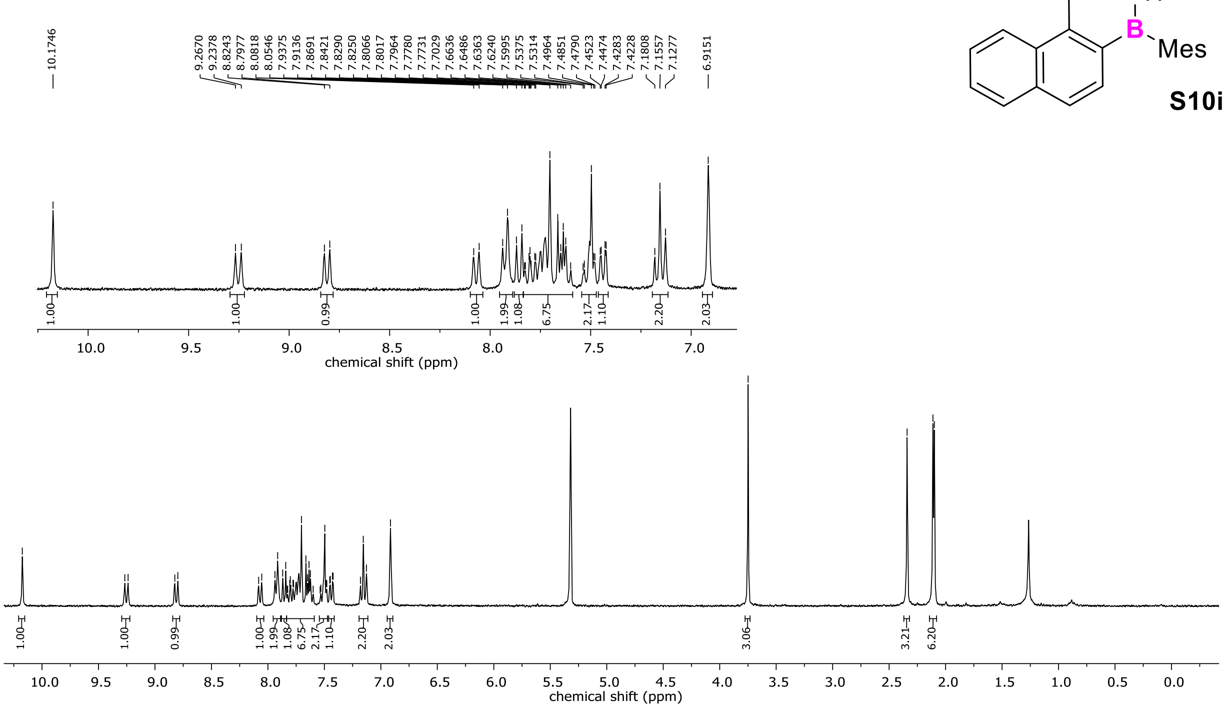

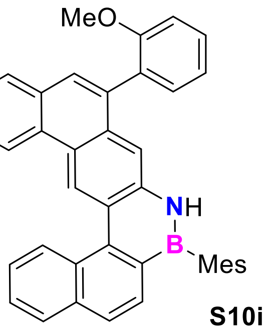

S10i

^{13}C NMR (126 MHz, $CDCl_3$)

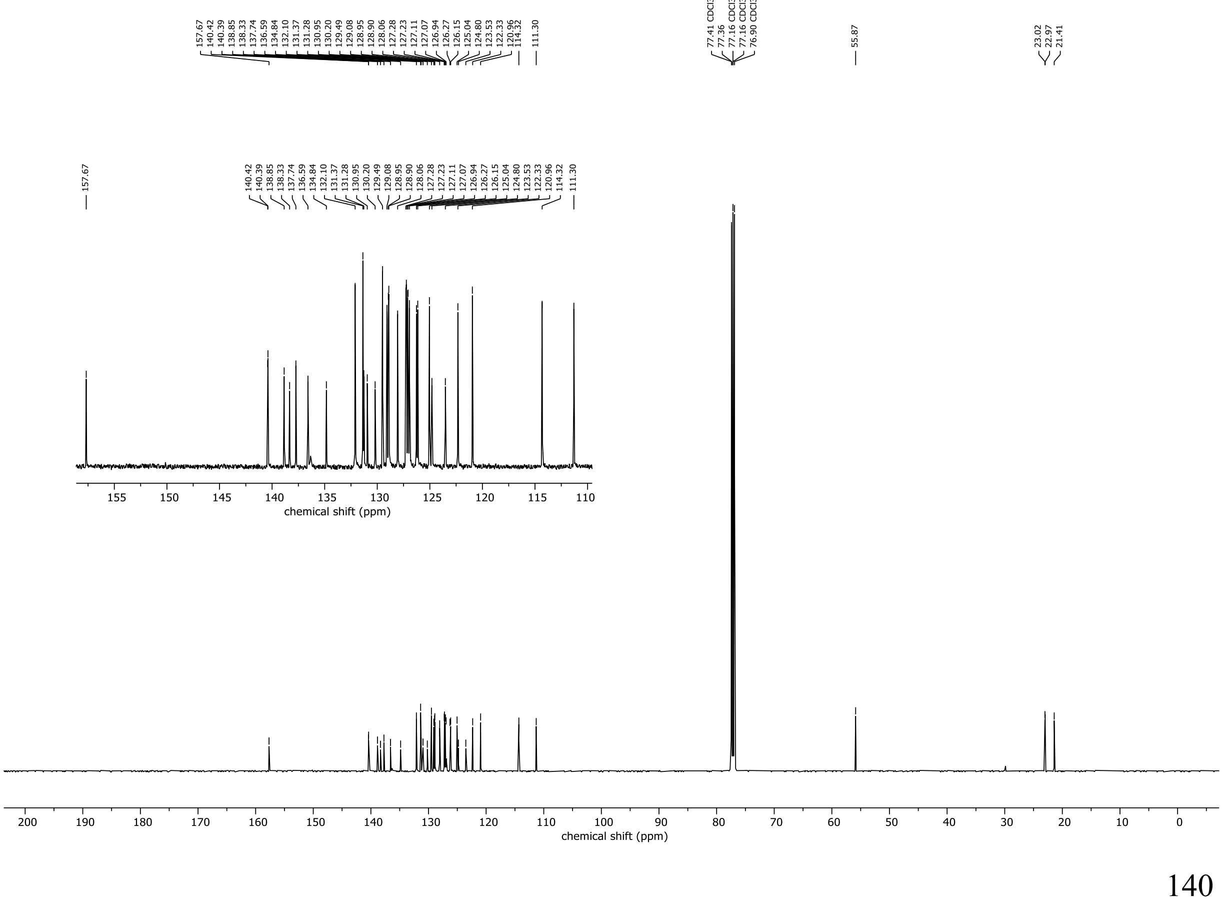

^{11}B NMR (128 MHz, $CDCl_3$)

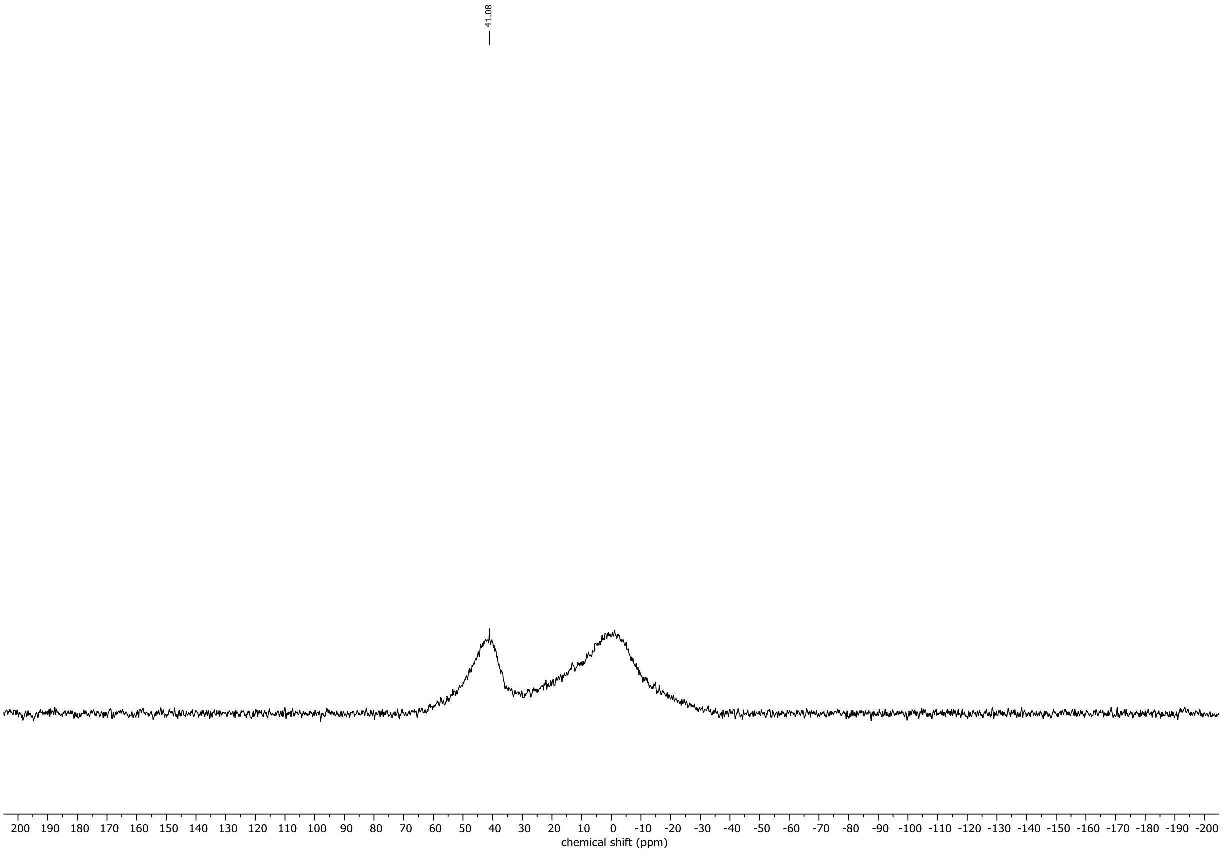

^{1}H NMR (500 MHz, CD_2Cl_2) **S10j**

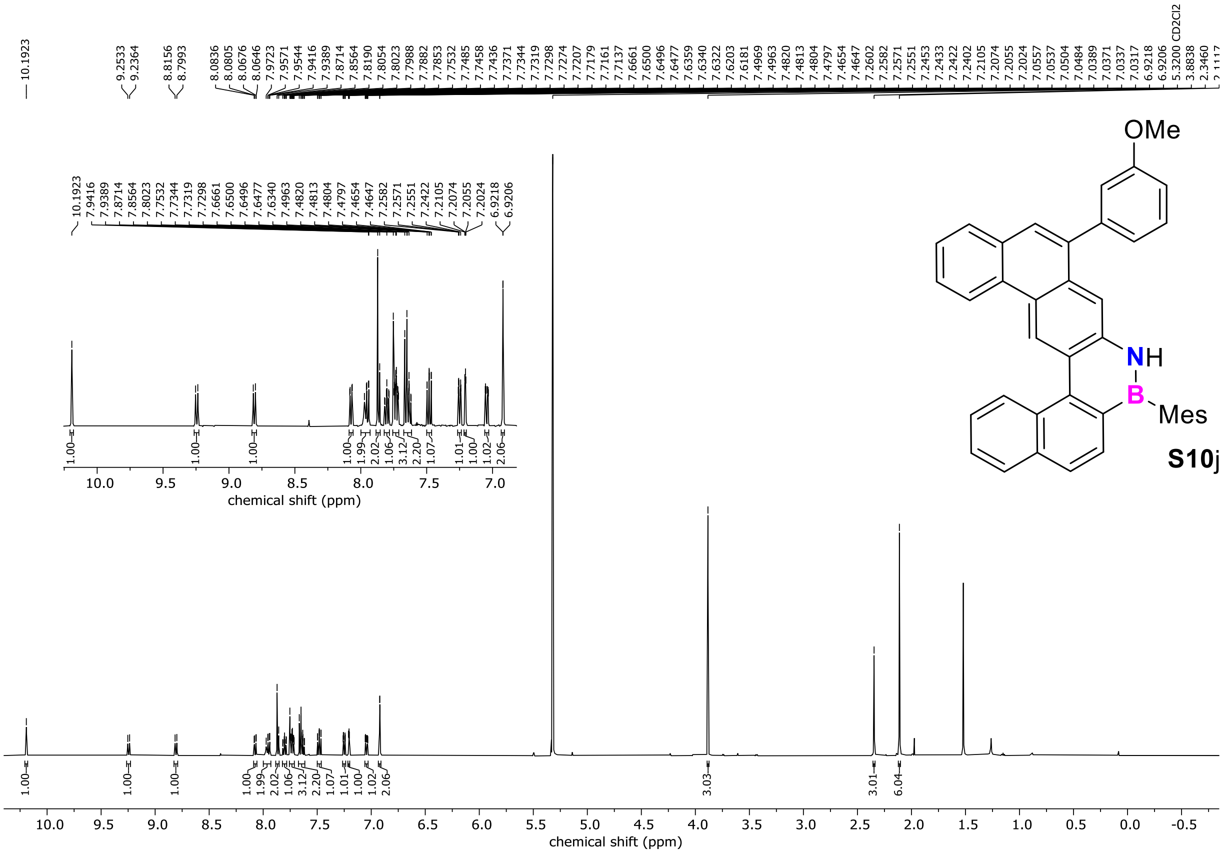

^{13}C NMR (126 MHz, CD_2Cl_2)

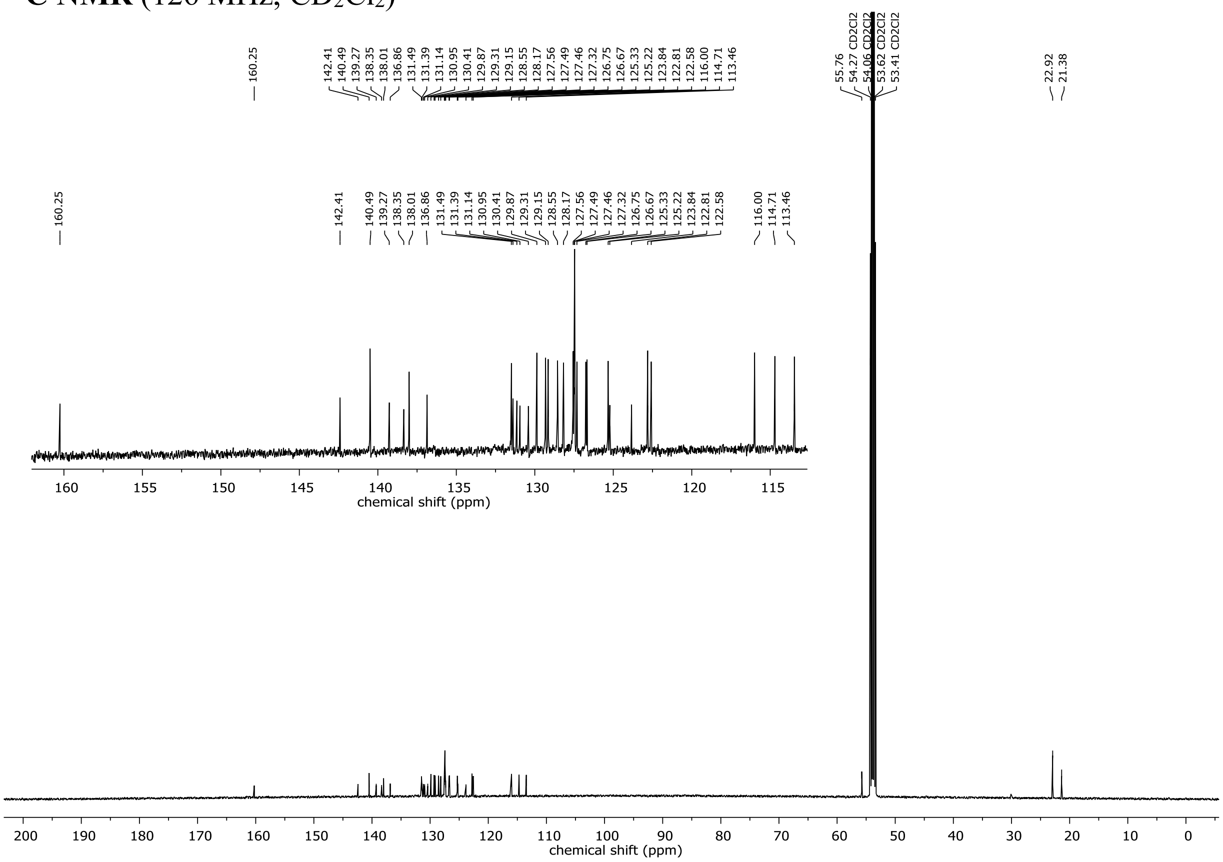

^{11}B NMR (161 MHz, CD_2Cl_2)

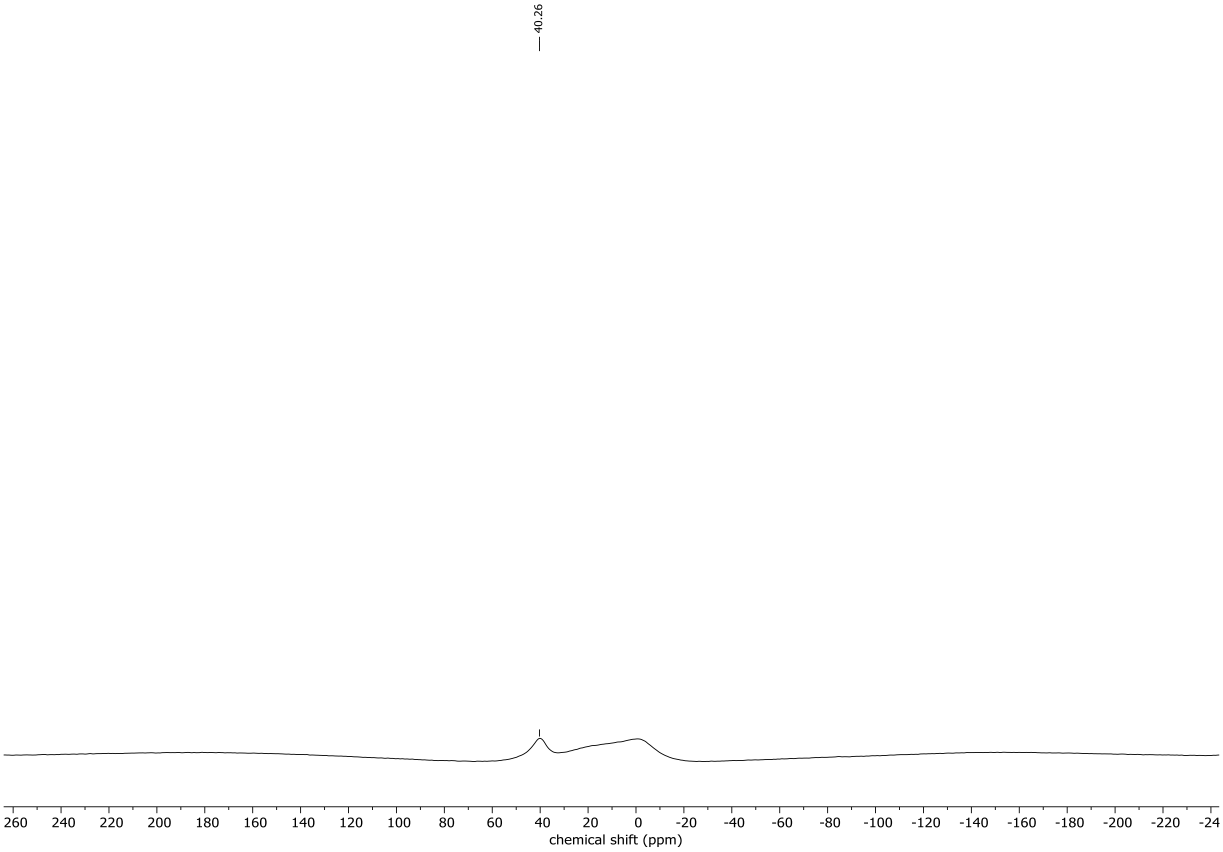

^{1}H NMR (400 MHz, $CDCl_3$) S10b(BPh)

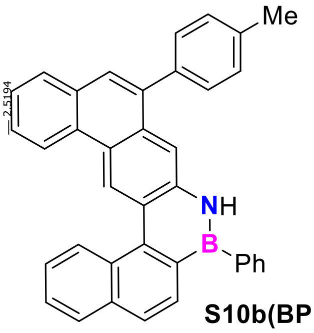

S10b(BPh)

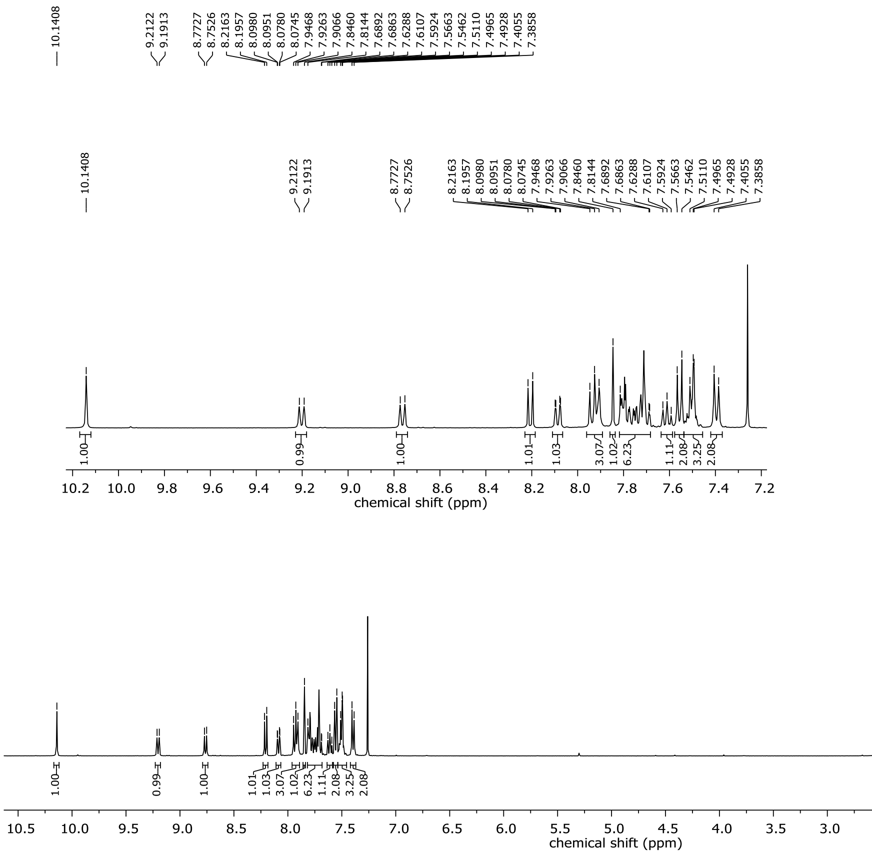

^{13}C NMR (101 MHz, $CDCl_3$)

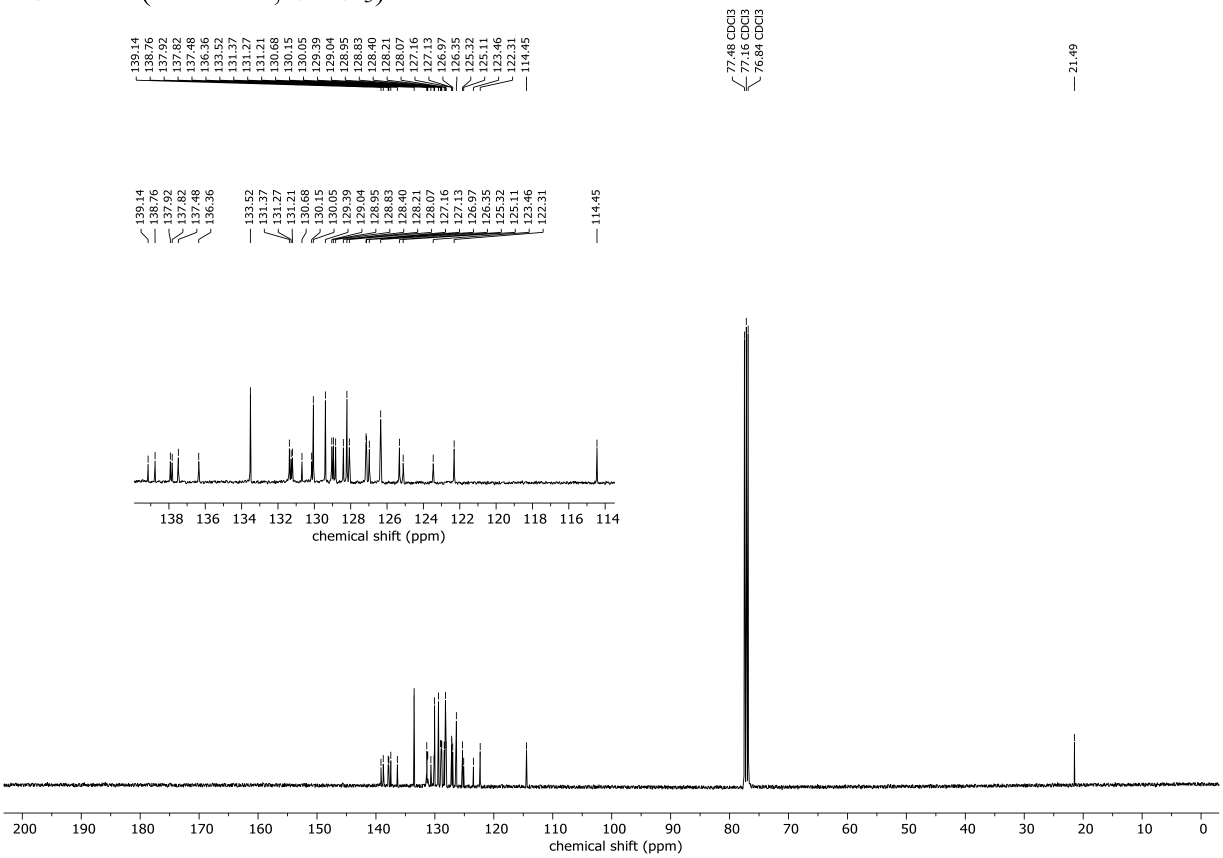

^{11}B NMR (161 MHz, $CDCl_3$)

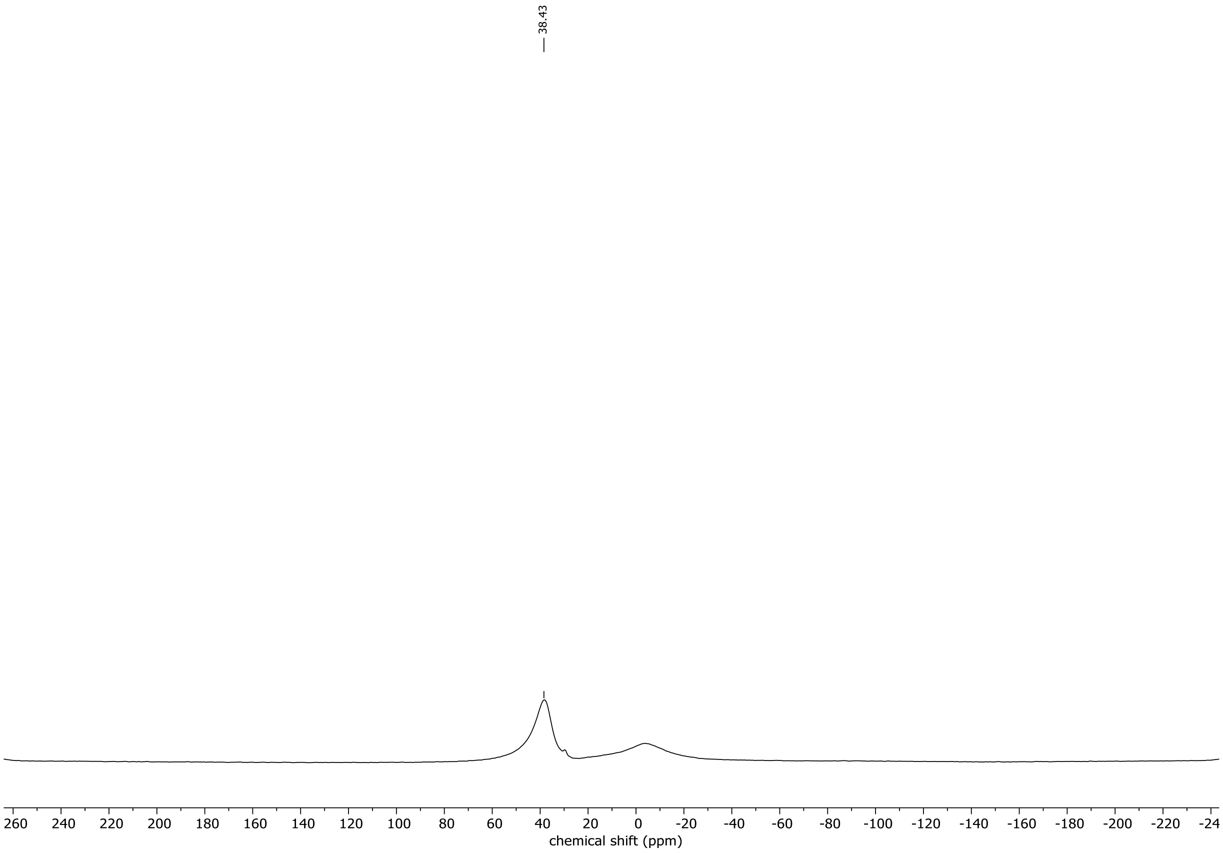

^{1}H NMR (500 MHz, CD_2Cl_2) **S10b(NMe)**

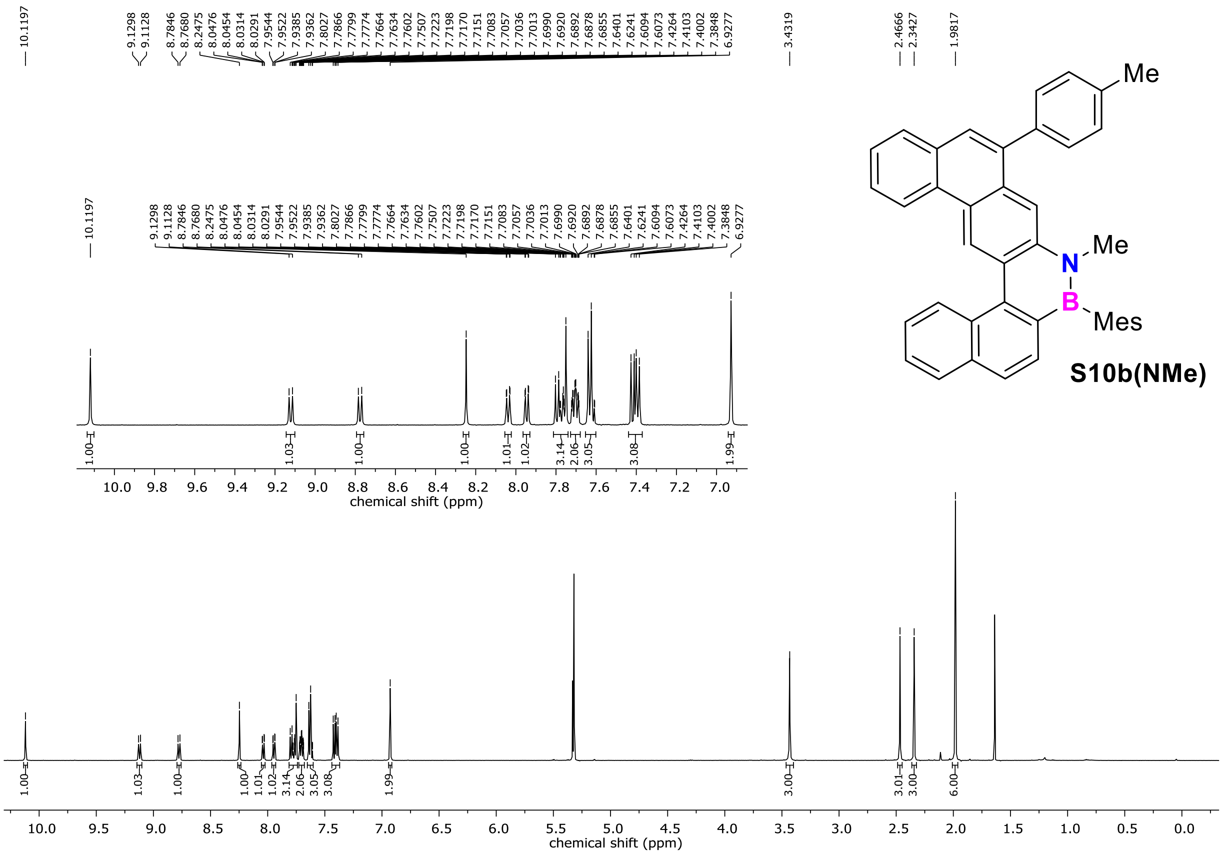

S10b(NMe)

^{13}C NMR (126 MHz, CD_2Cl_2)

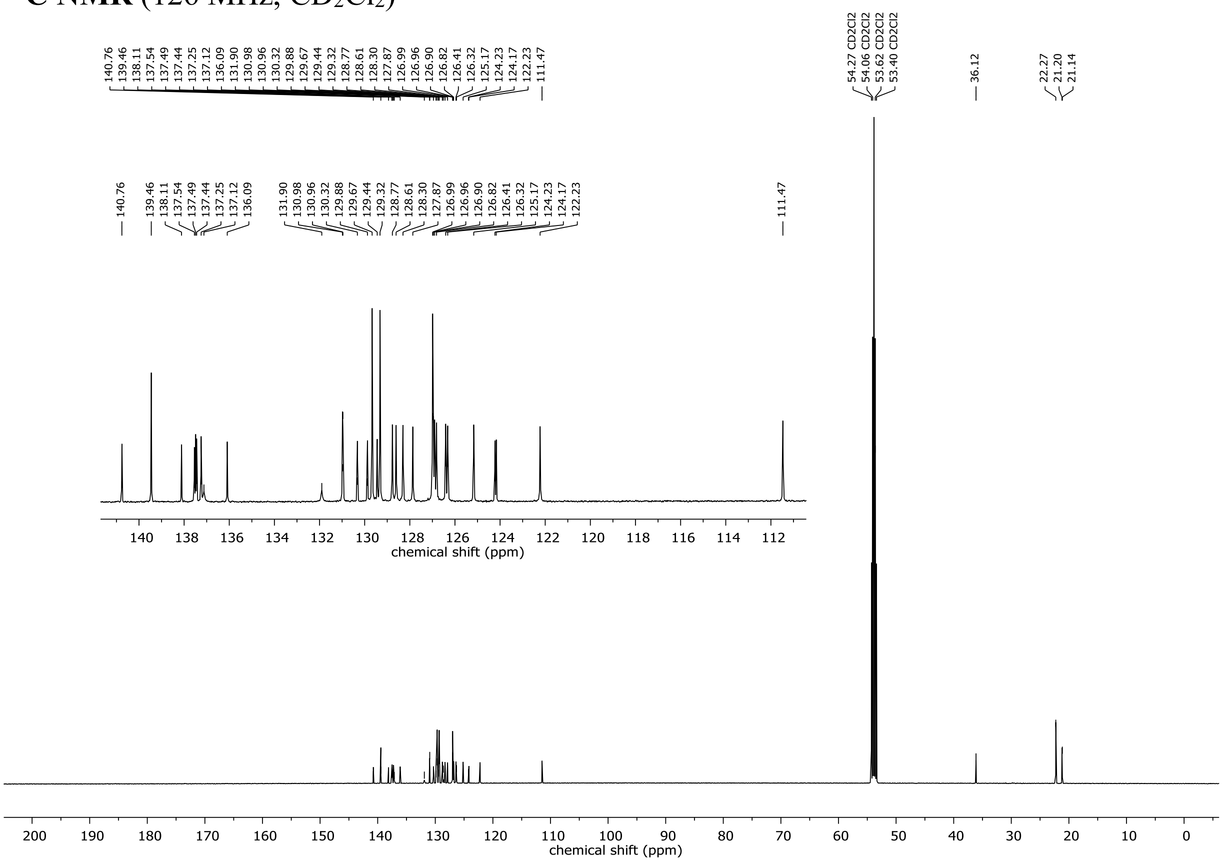

^{11}B NMR (161 MHz, CD_2Cl_2)

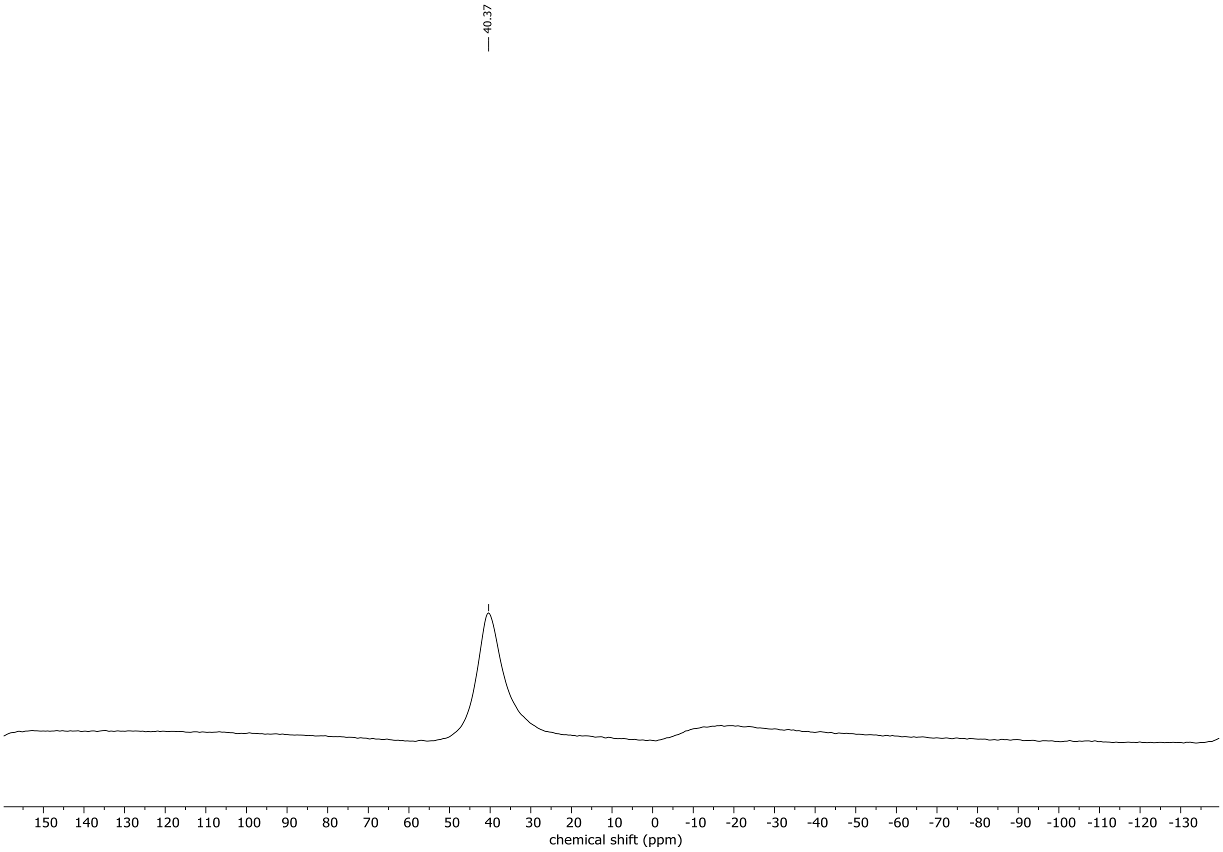

^{1}H NMR (400 MHz, $CDCl_3$) **11b**

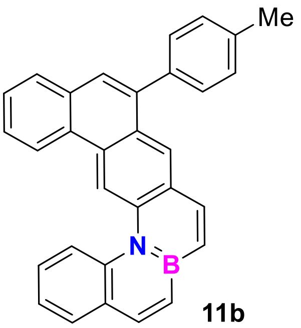

11b

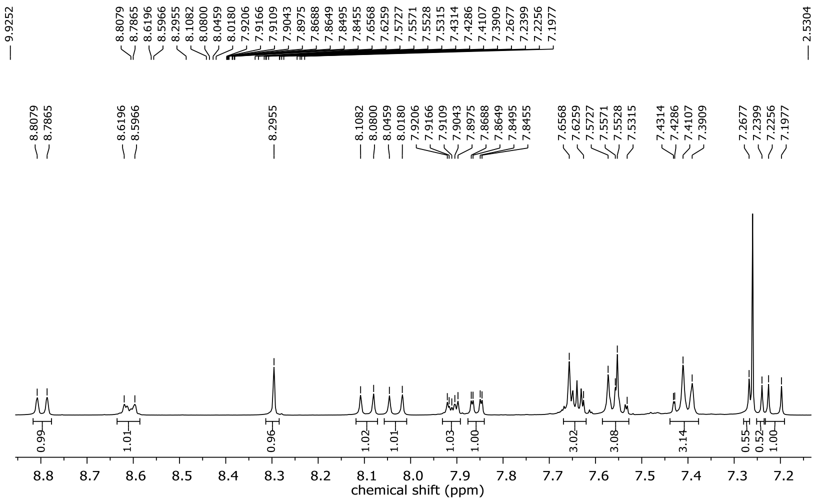

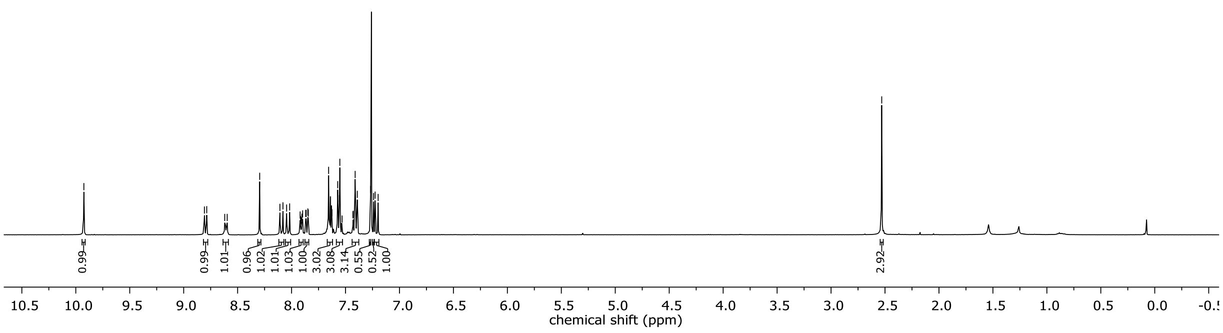

^{13}C NMR (101 MHz, $CDCl_3$)

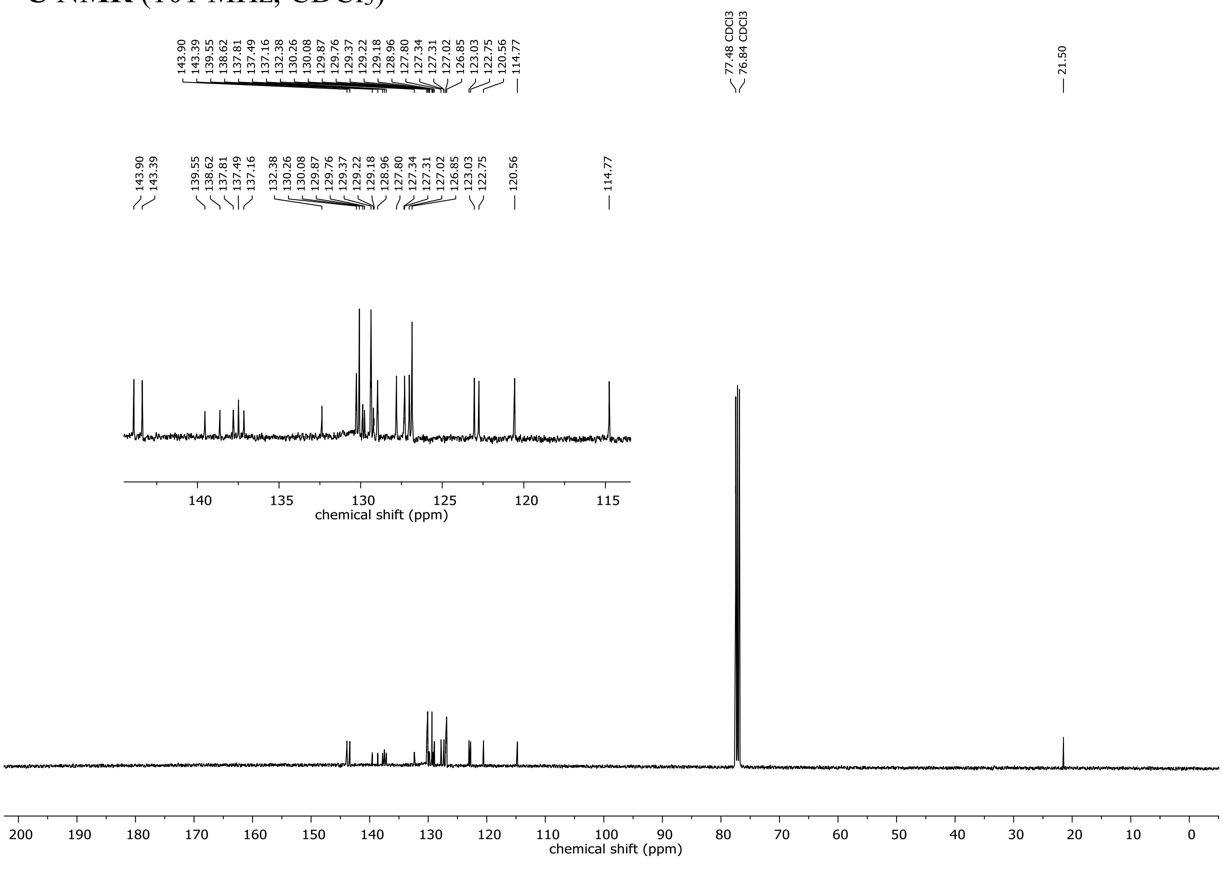

^{11}B NMR (161 MHz, $CDCl_3$)

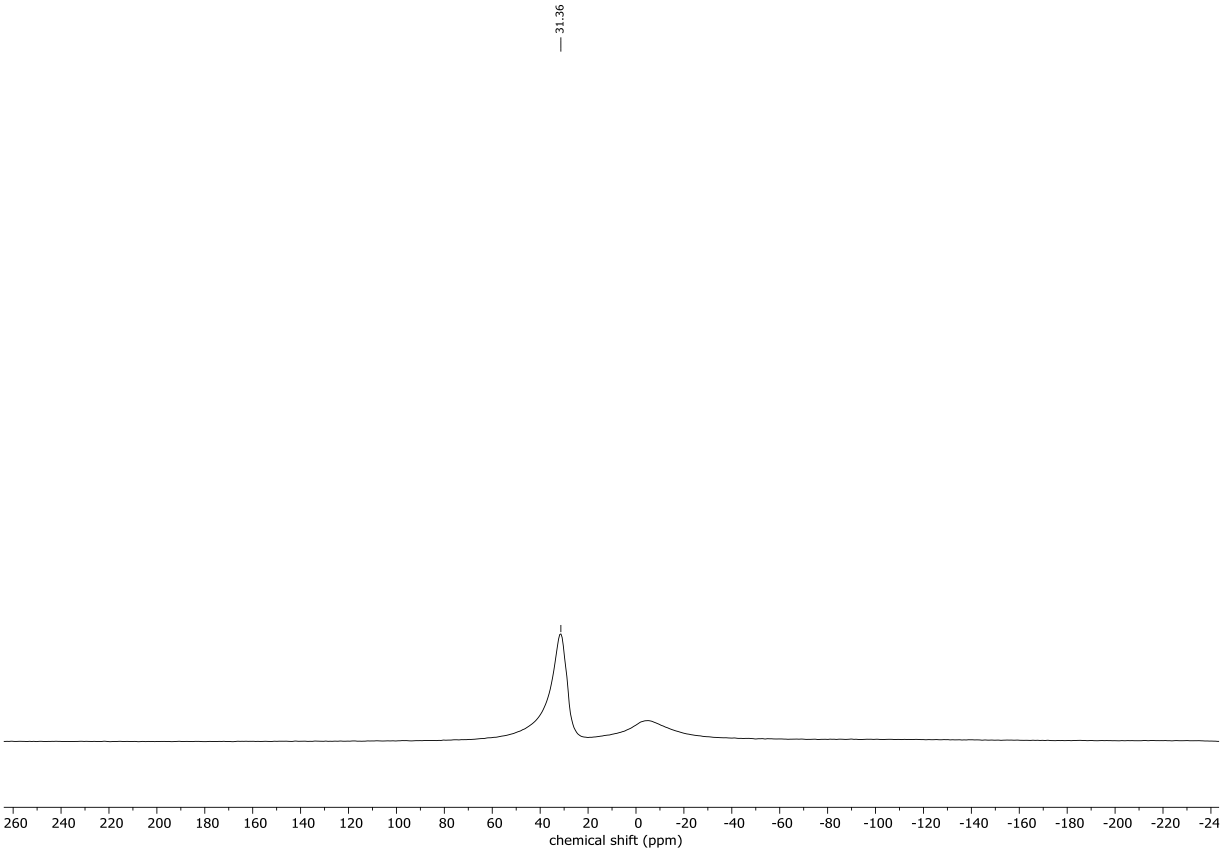

^{1}H NMR (400 MHz, $CDCl_3$) S11c

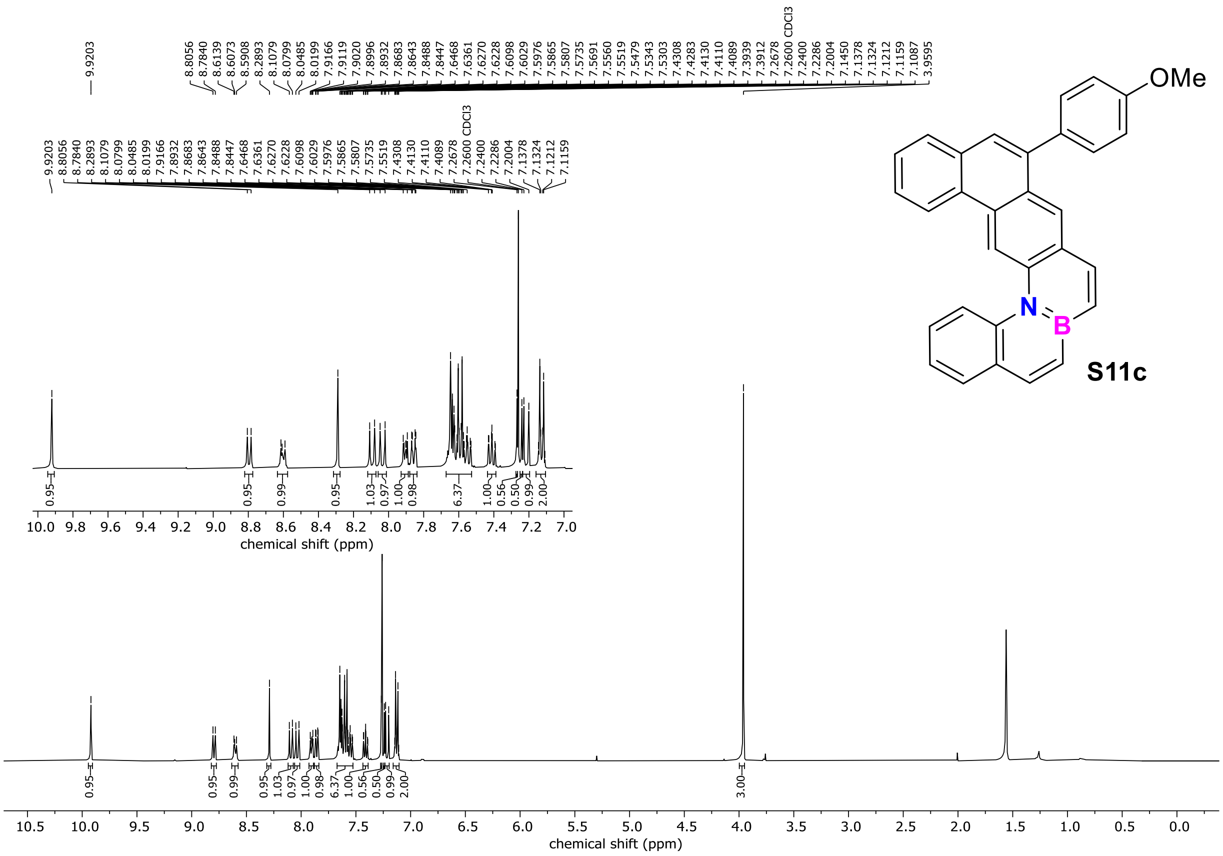

^{13}C NMR (101 MHz, $CDCl_3$)

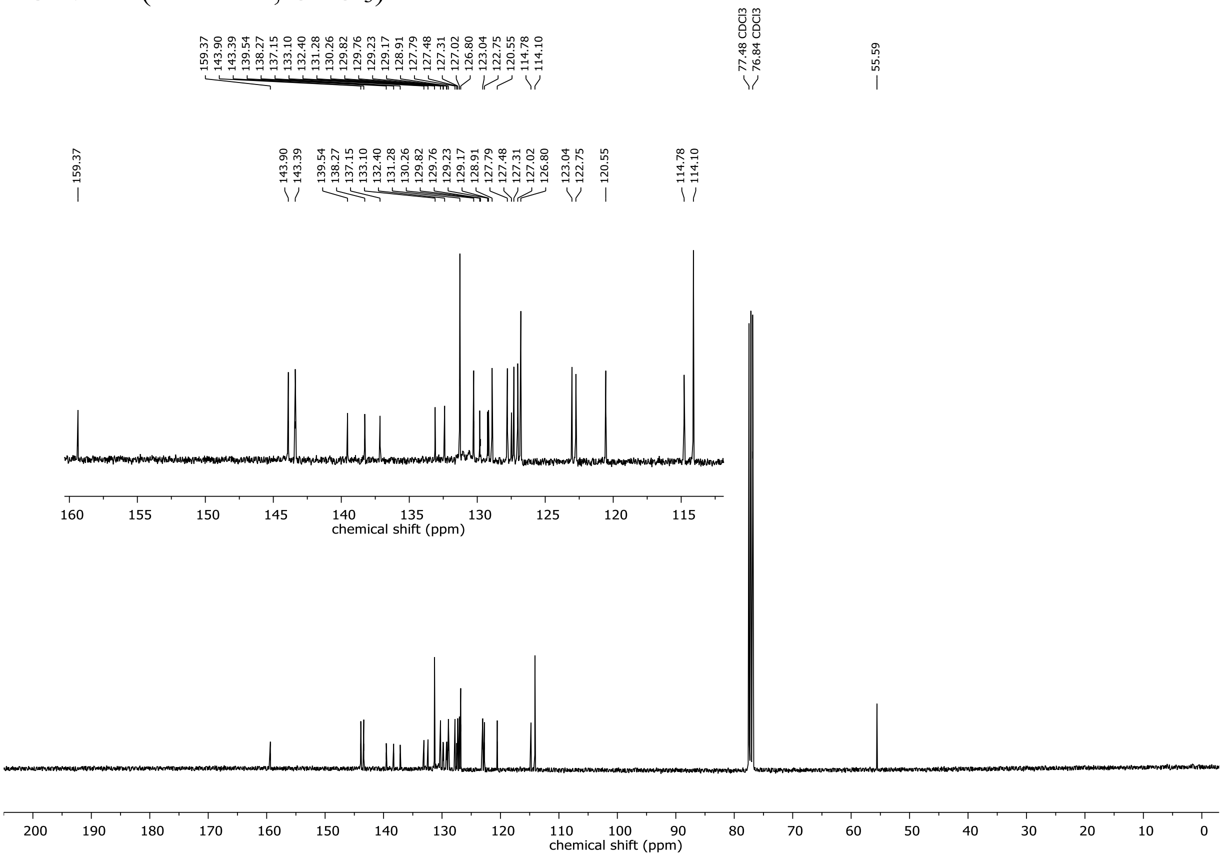

^{11}B NMR (128 MHz, $CDCl_3$)

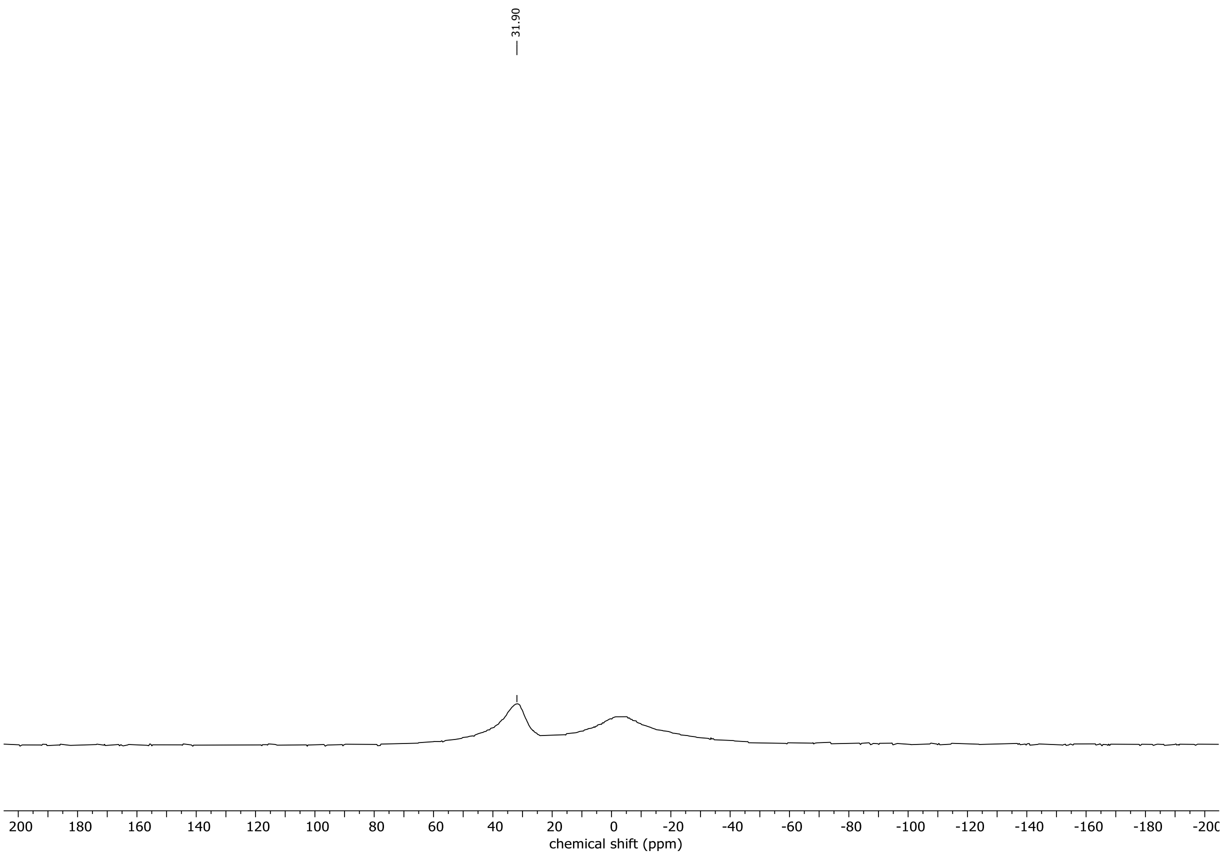

^{1}H NMR (400 MHz, $CDCl_3$) S11e

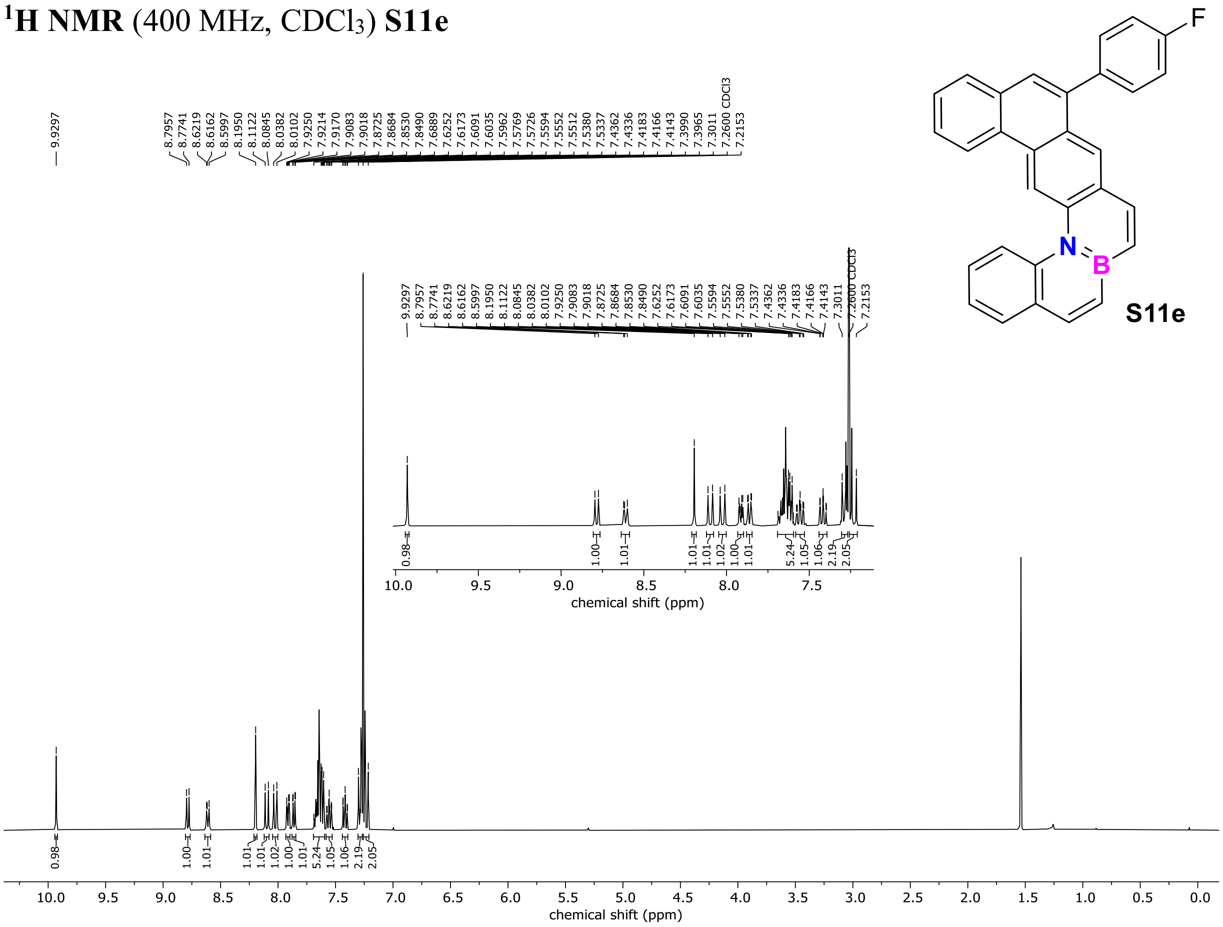

S11e

^{13}C NMR (101 MHz, $CDCl_3$)

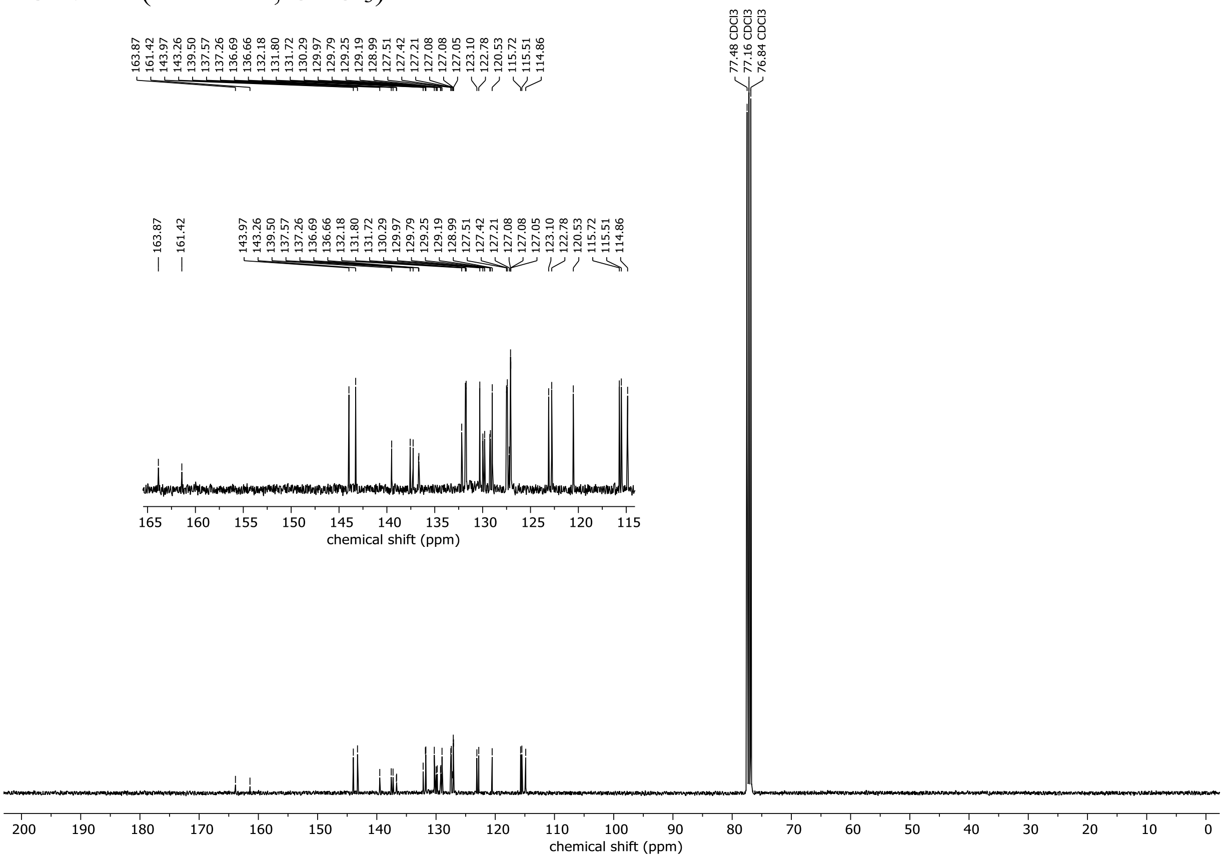

^{11}B NMR (161 MHz, $CDCl_3$)

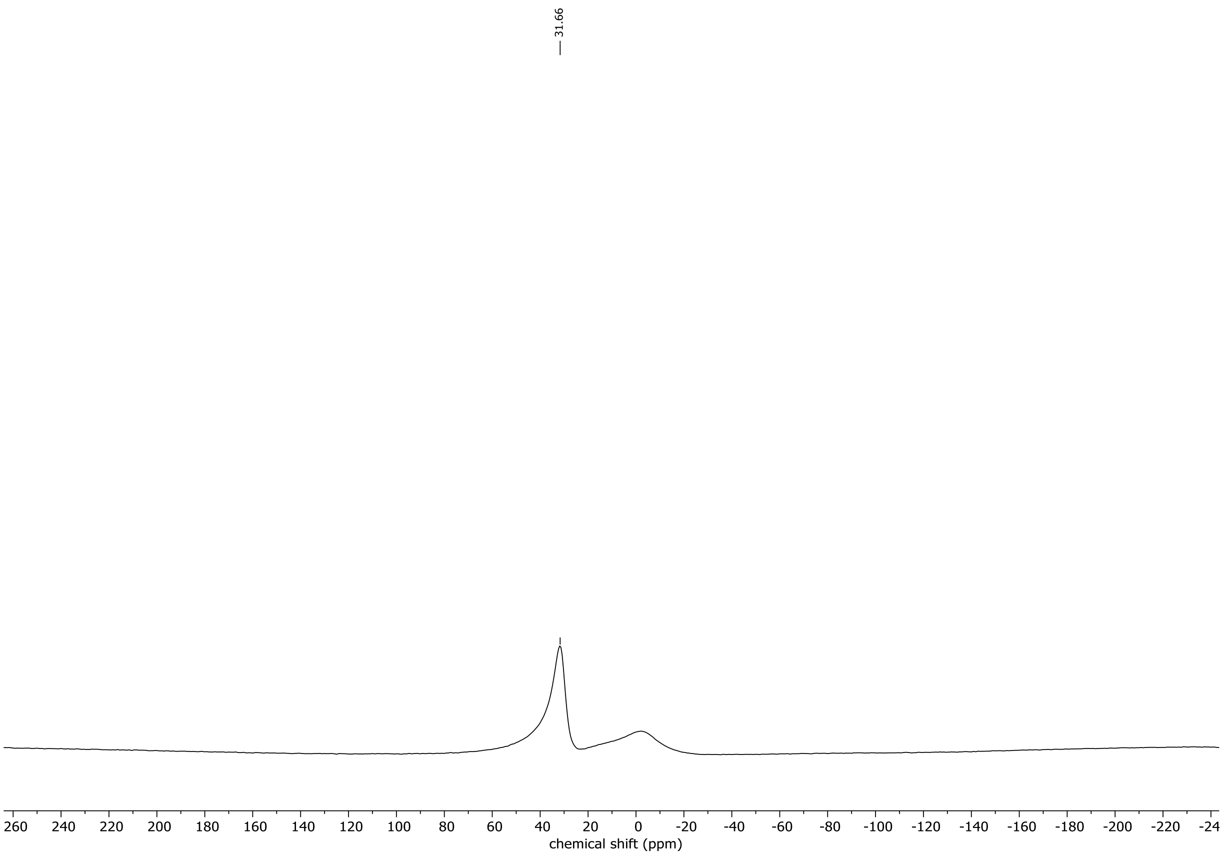

^{19}F NMR (282 MHz, $CDCl_3$)

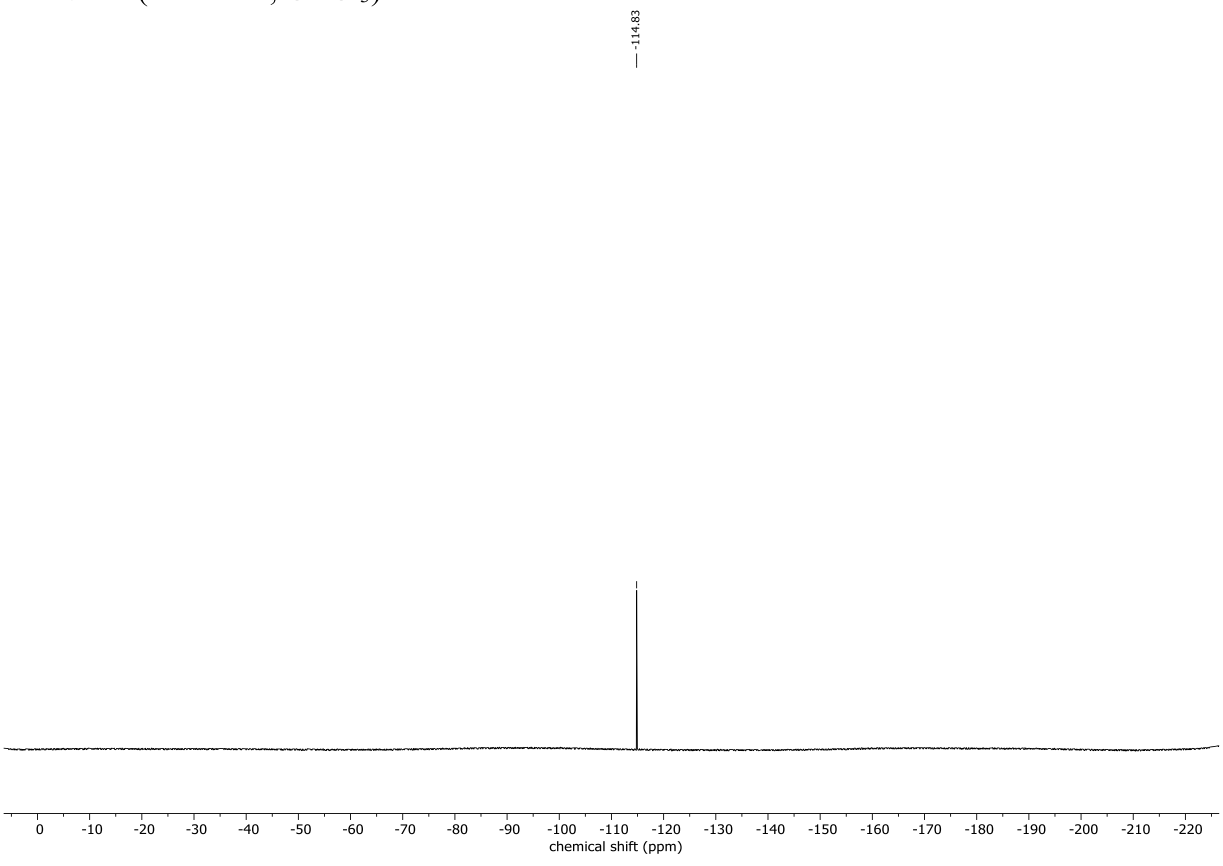

^{1}H NMR (500 MHz, CD_2Cl_2, –35 °C) **12b**

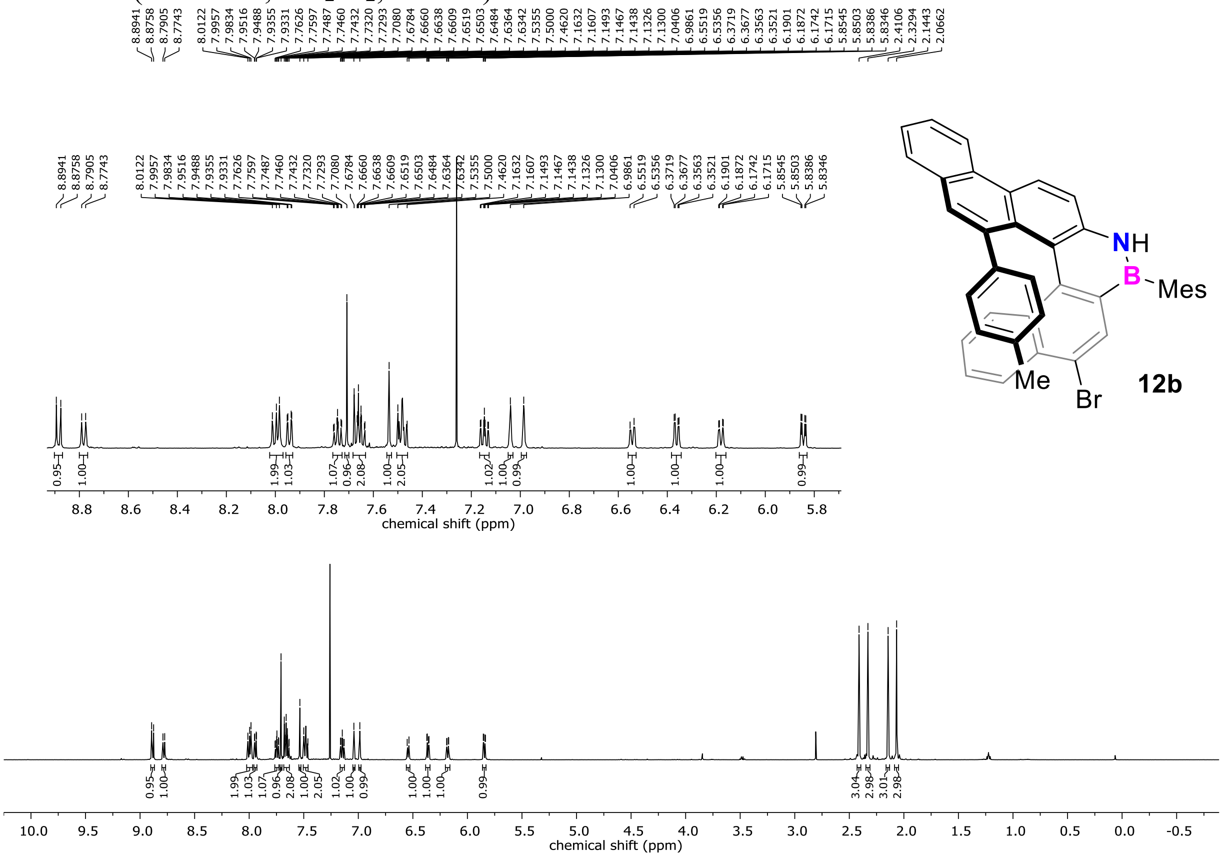

12b

^{1}H NMR (300 MHz, $CDCl_3$; rt)

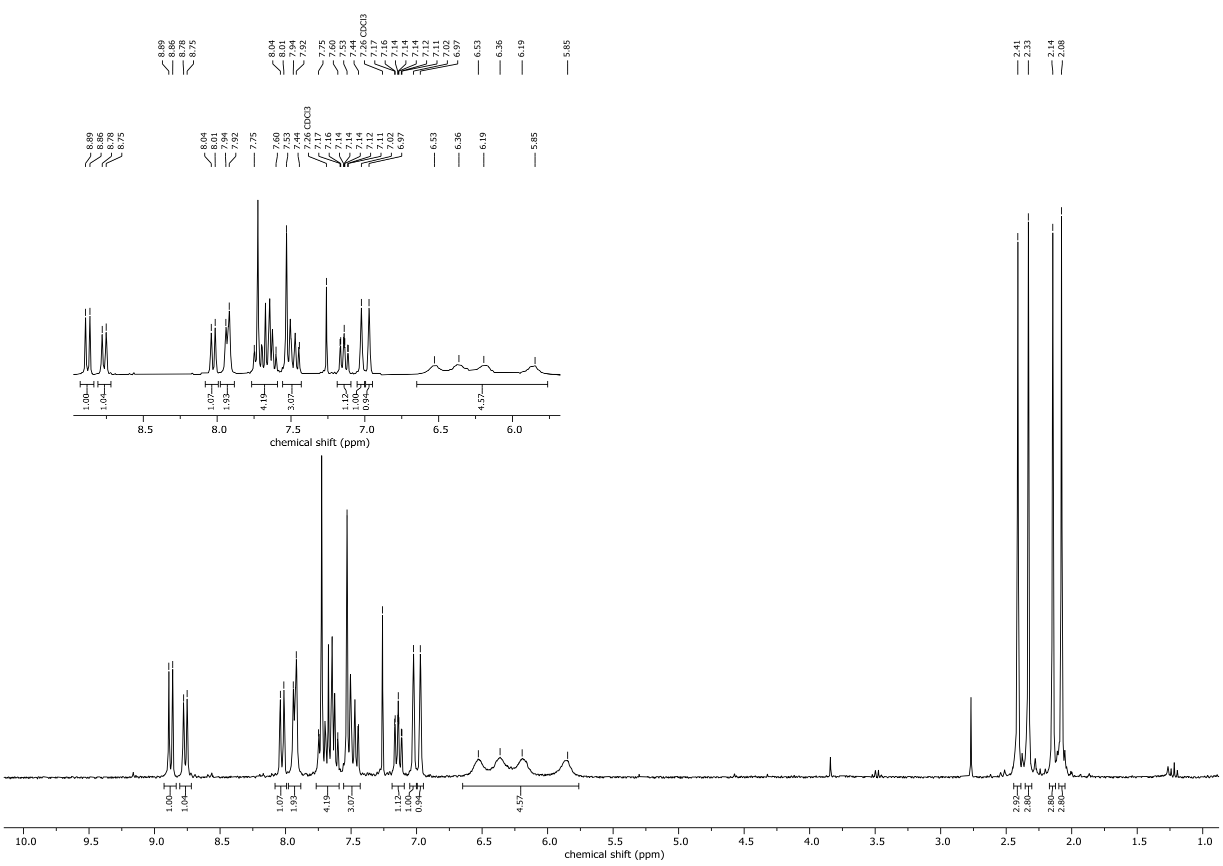

^{13}C NMR (126 MHz, CD_2Cl_2, –35 °C)

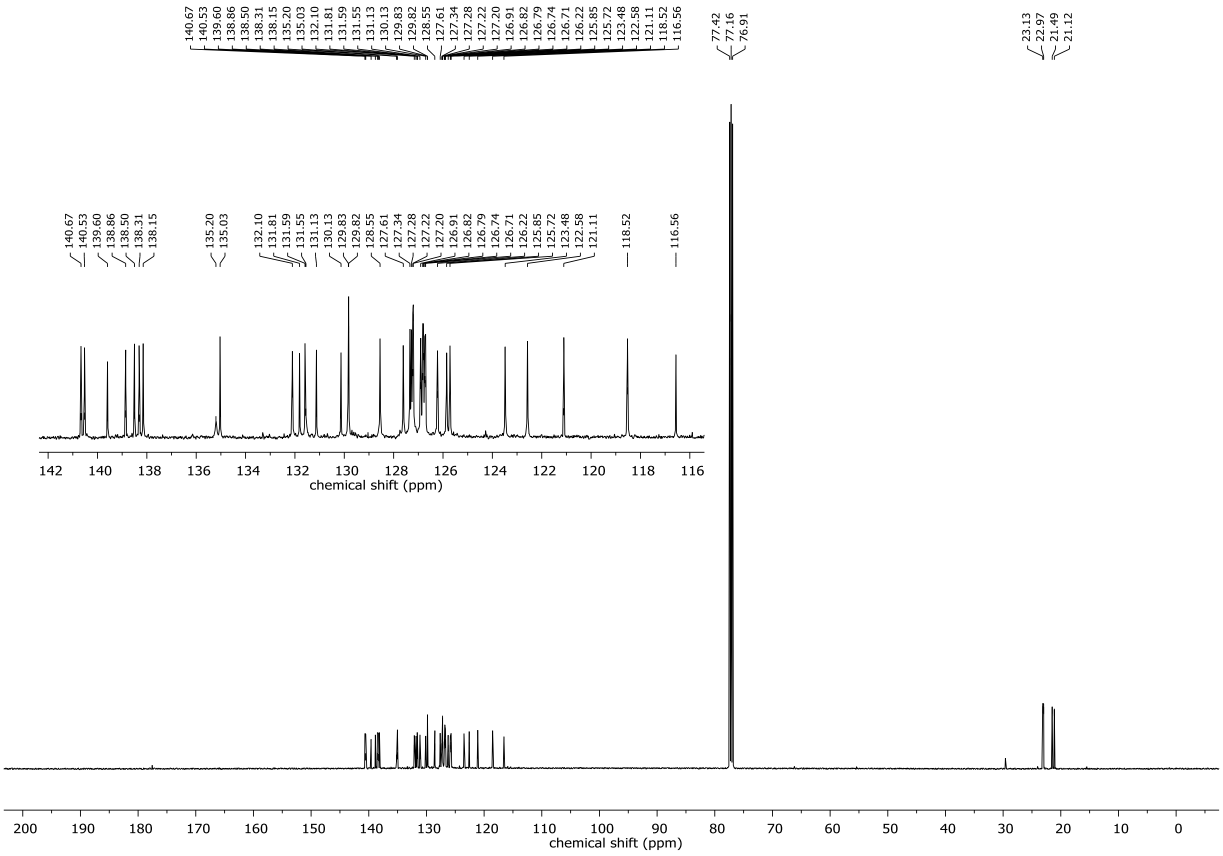

^{11}B NMR (161 MHz, $CDCl_3$)

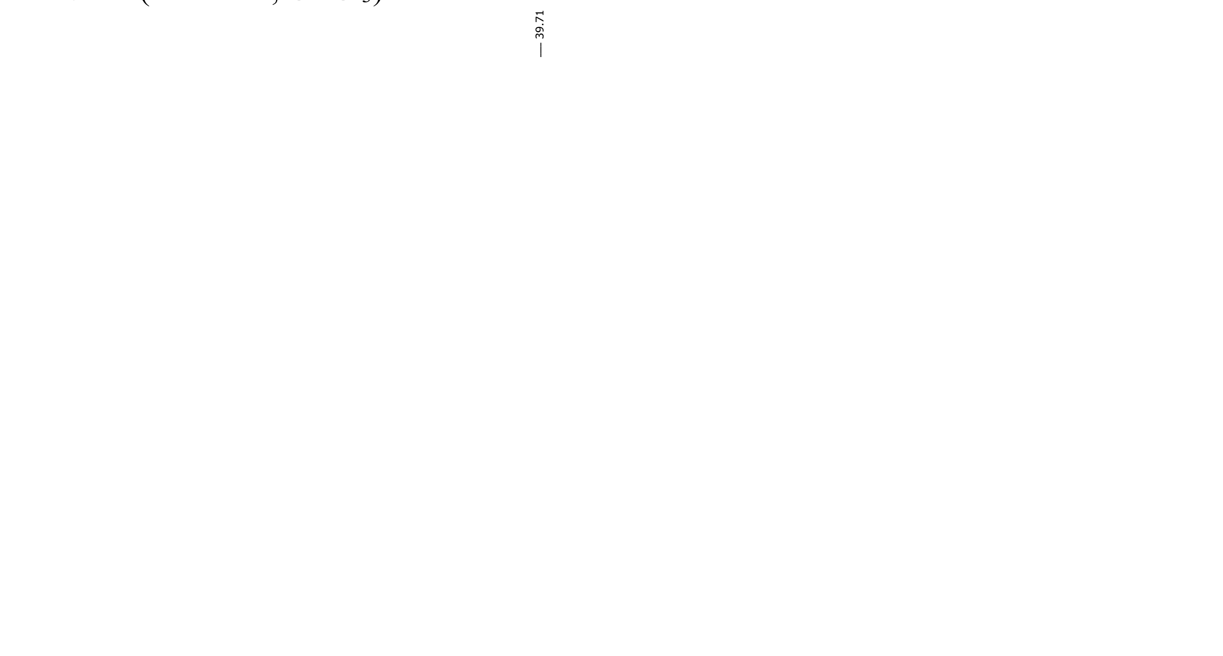

^{1}H NMR (500 MHz, $CDCl_3$, –35 °C) **13b**

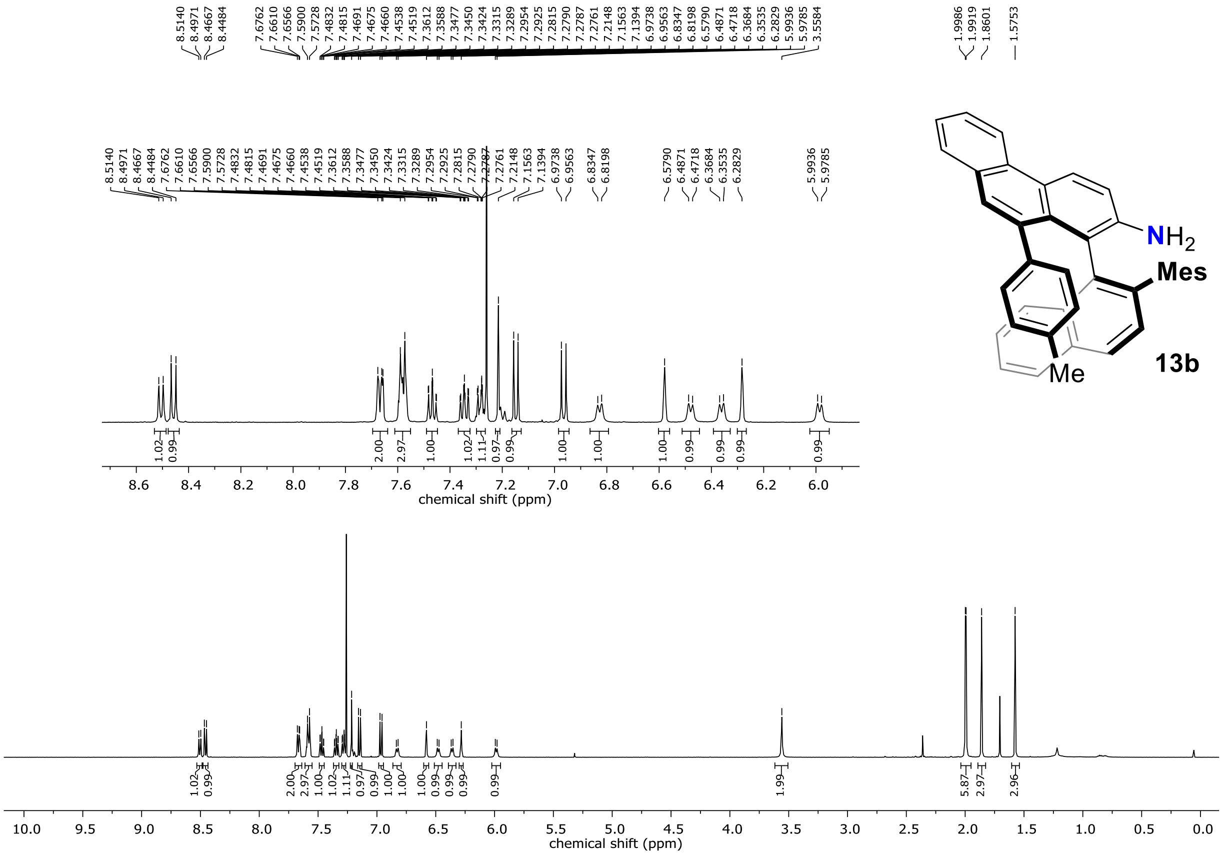

13b

^{1}H NMR (400 MHz, $CDCl_3$; rt)

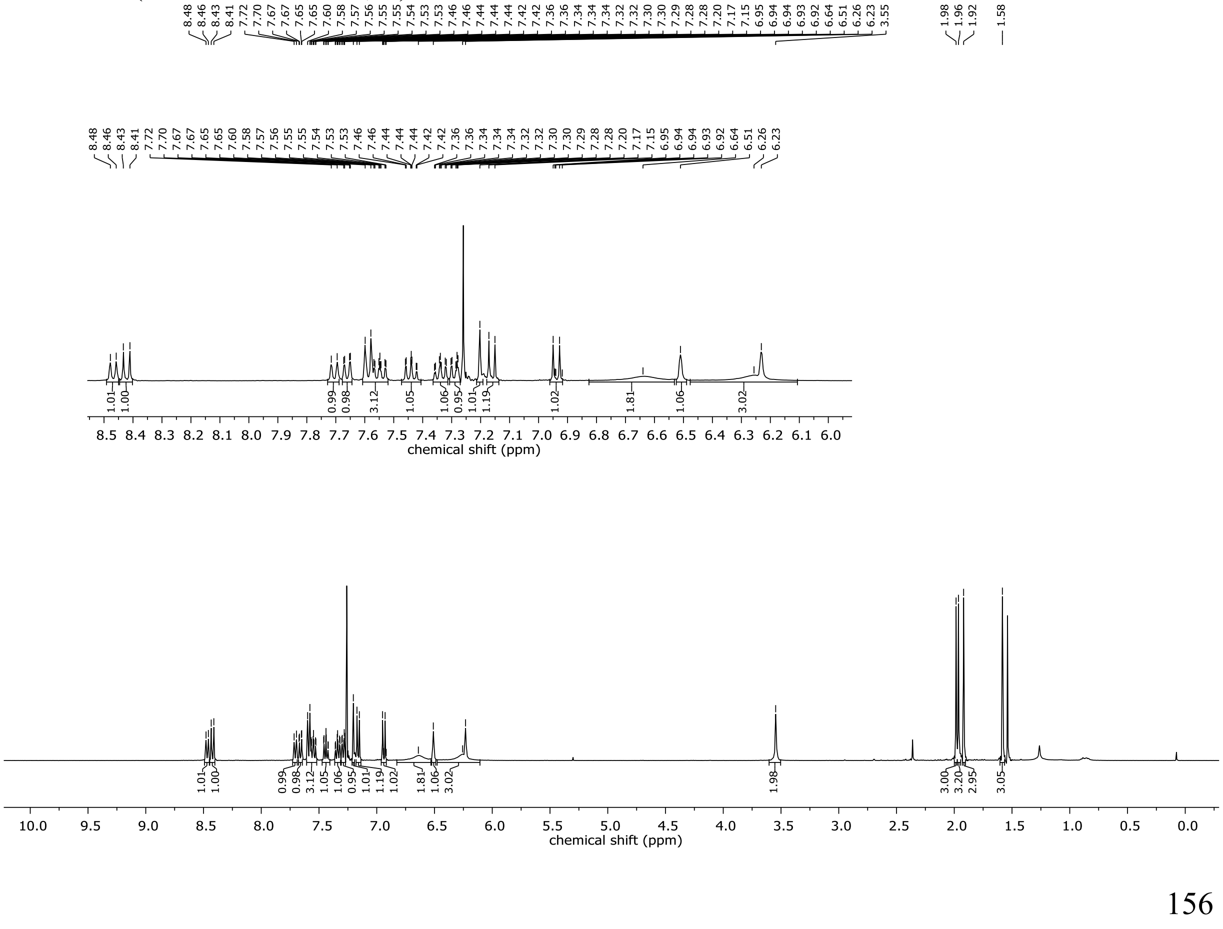

^{13}**C NMR** (126 MHz, $CDCl_3$, –35 °C)

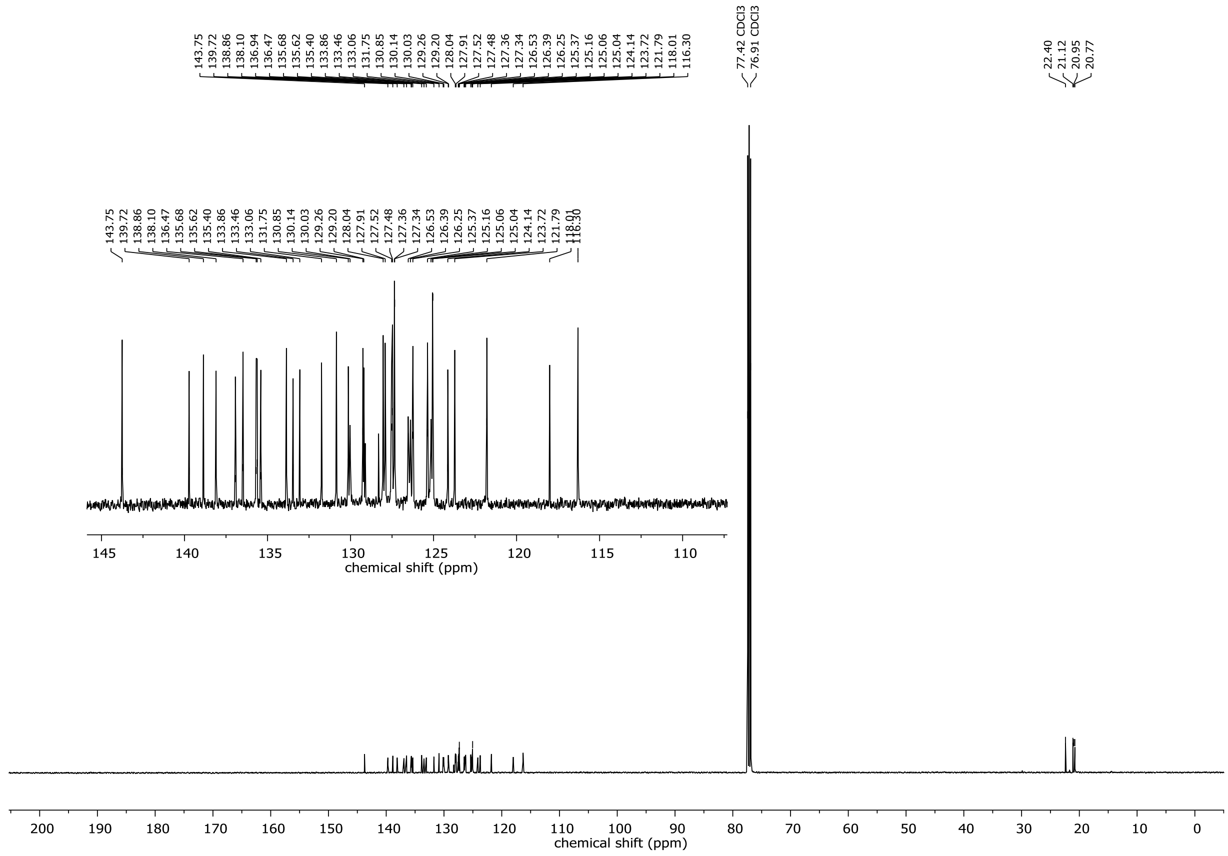

4. HPLC-Chromatograms

Machine: Waters MDLC; Column: Daicel CHIRALPAK IC-U (3.0 x 100 mm), 1.6 µm; Eluent: MeCN:H_2O = 90:10, isocratic 0.5 mL/min, 303 K. Detection : 254 nm.

Racemic-1a

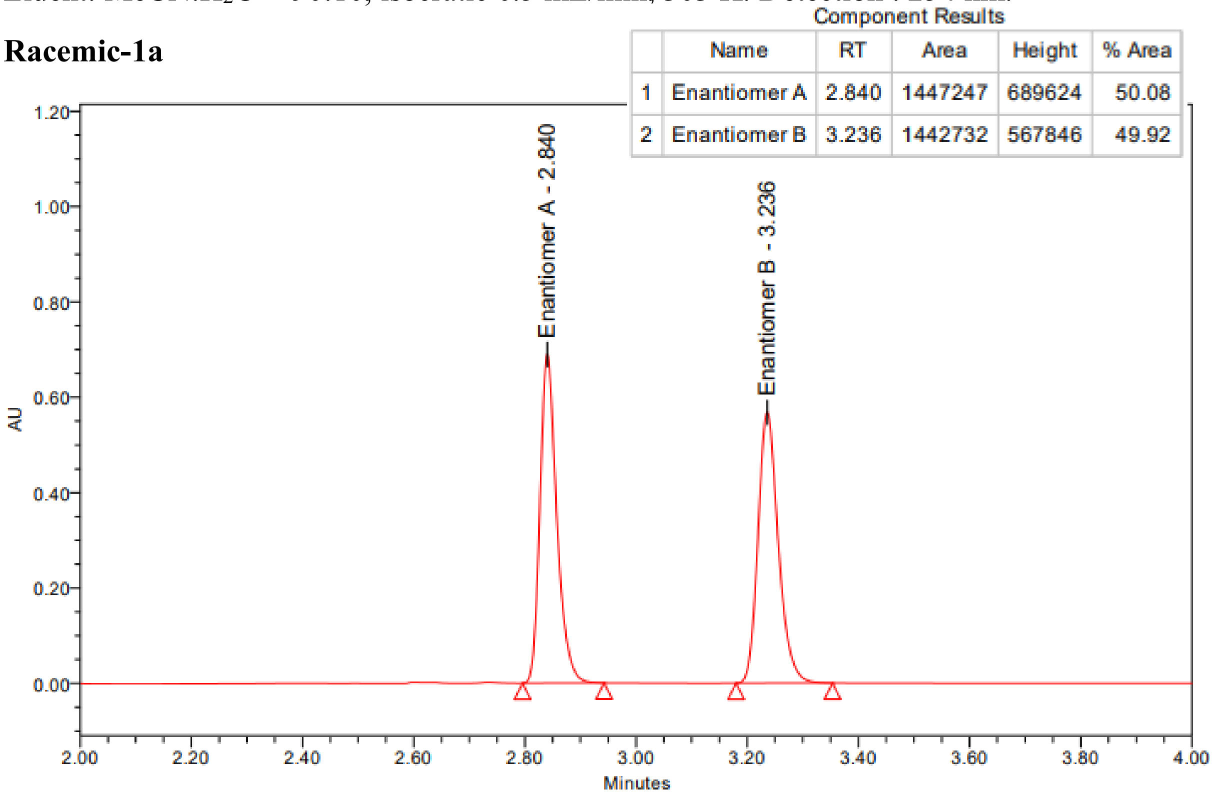

Component Results

	Name	RT	Area	Height	% Area
1	Enantiomer A	2.840	1447247	689624	50.08
2	Enantiomer B	3.236	1442732	567846	49.92

Enantioenriched-1a

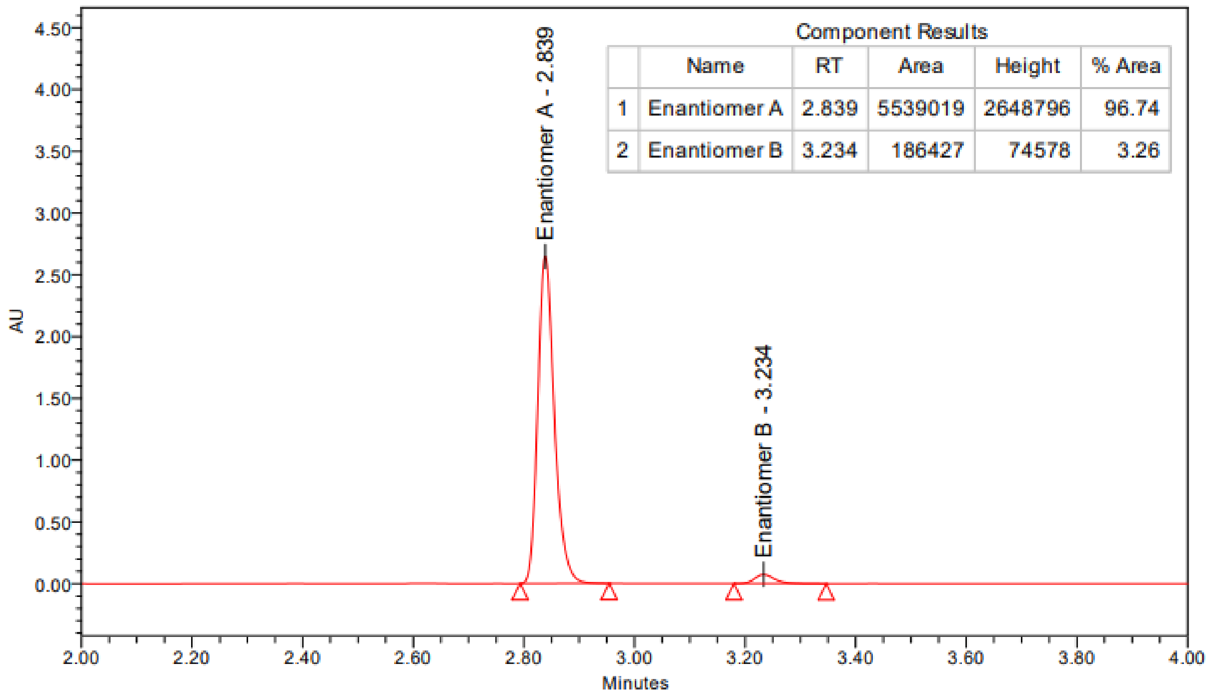

Component Results

	Name	RT	Area	Height	% Area
1	Enantiomer A	2.839	5539019	2648796	96.74
2	Enantiomer B	3.234	186427	74578	3.26

Machine: Waters MDLC; Column: Daicel CHIRALPAK IC-U (3.0 x 100 mm), 1.6 µm; Eluent: MeCN:H_2O = 90:10, isocratic 0.5mL/min, 303 K. Detection : 310 nm.

Racemic-1b

Component Results

	Name	RT	Area	Height	% Area
1	Enantiomer A	2.919	1190510	523178	50.23
2	Enantiomer B	3.536	1179795	393091	49.77

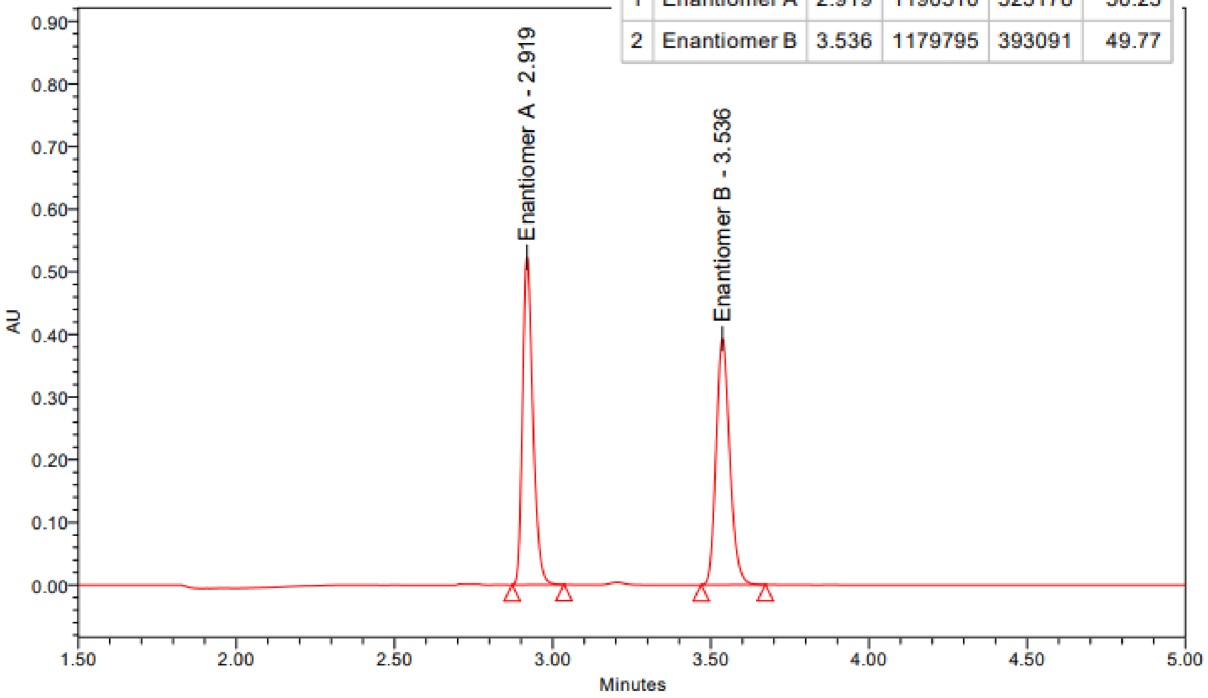

Enantioenriched-1b

Component Results

	Name	RT	Area	Height	% Area
1	Enantiomer A	2.913	7966057	3195451	98.31
2	Enantiomer B	3.529	136564	45206	1.69

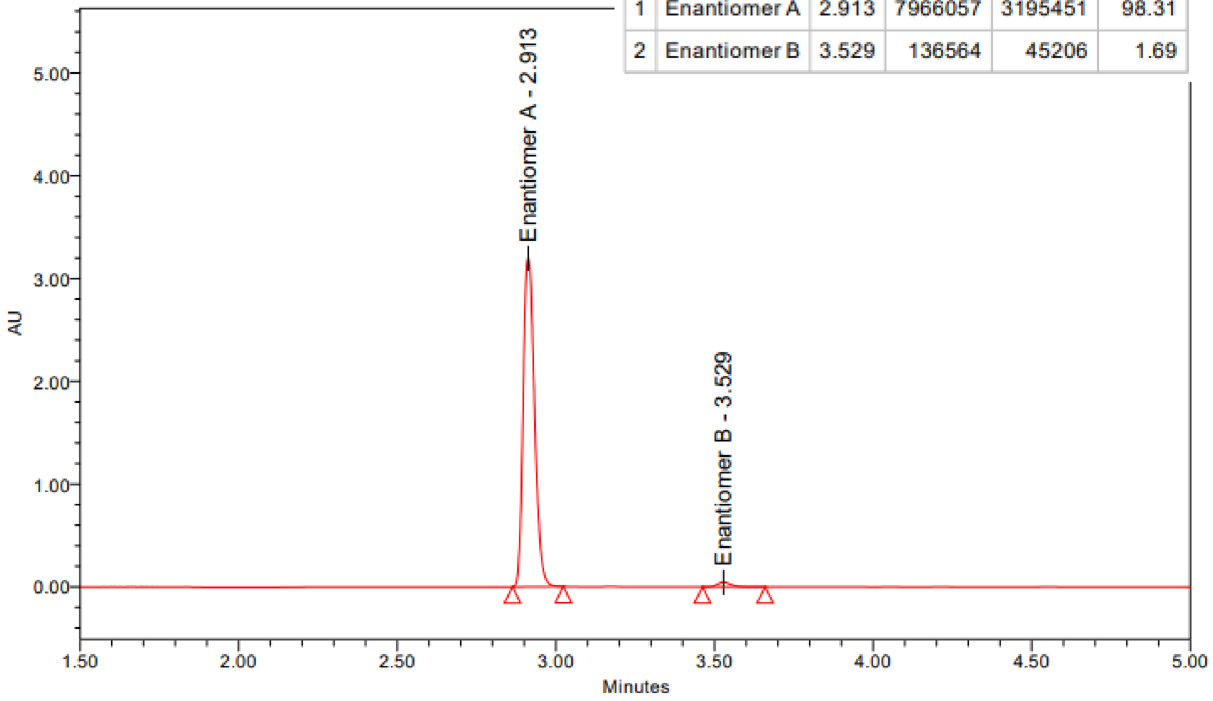

Machine: Waters MDLC; Column: Daicel CHIRALPAK IC-U (3.0 x 100 mm), 1.6 µm; Eluent: MeCN:H_2O = 95:5, isocratic 0.5 mL/min, 303 K. Detection : 254 nm.

Racemic-1c

Component Results

	Name	RT	Area	Height	% Area
1	Enantiomer A	2.534	389377	223590	50.03
2	Enantiomer B	2.787	388962	190741	49.97

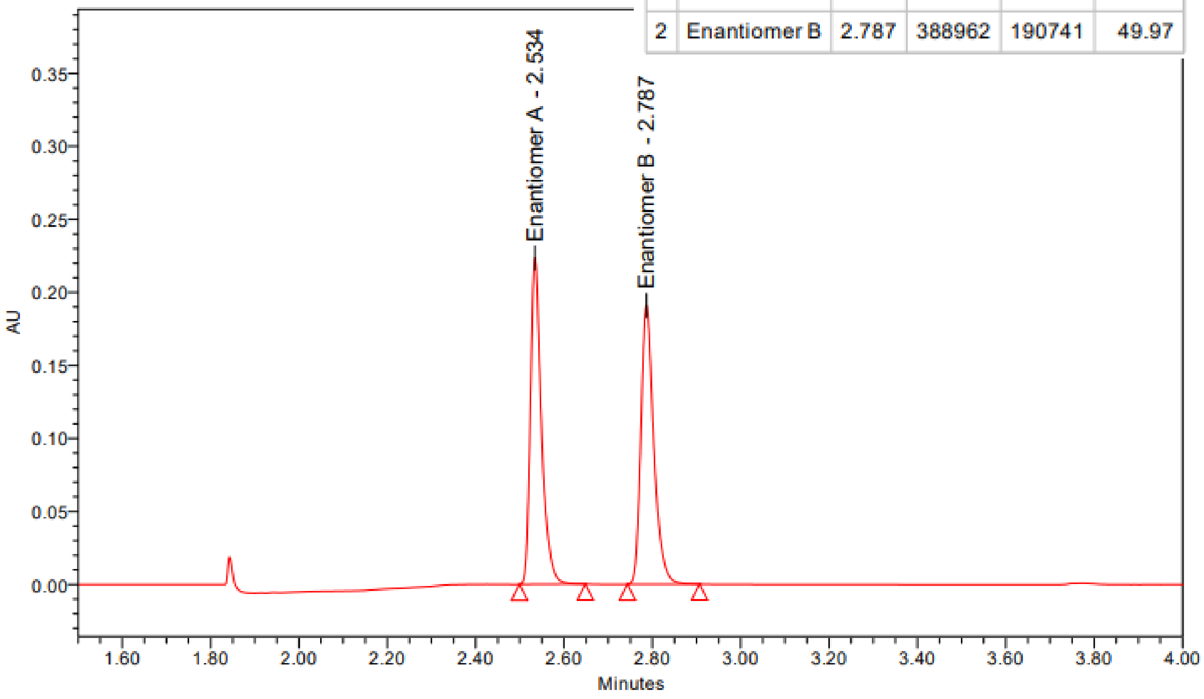

Enantioenriched-1c

Component Results

	Name	RT	Area	Height	% Area
1	Enantiomer A	2.529	6237933	3174548	97.69
2	Enantiomer B	2.781	147557	71246	2.31

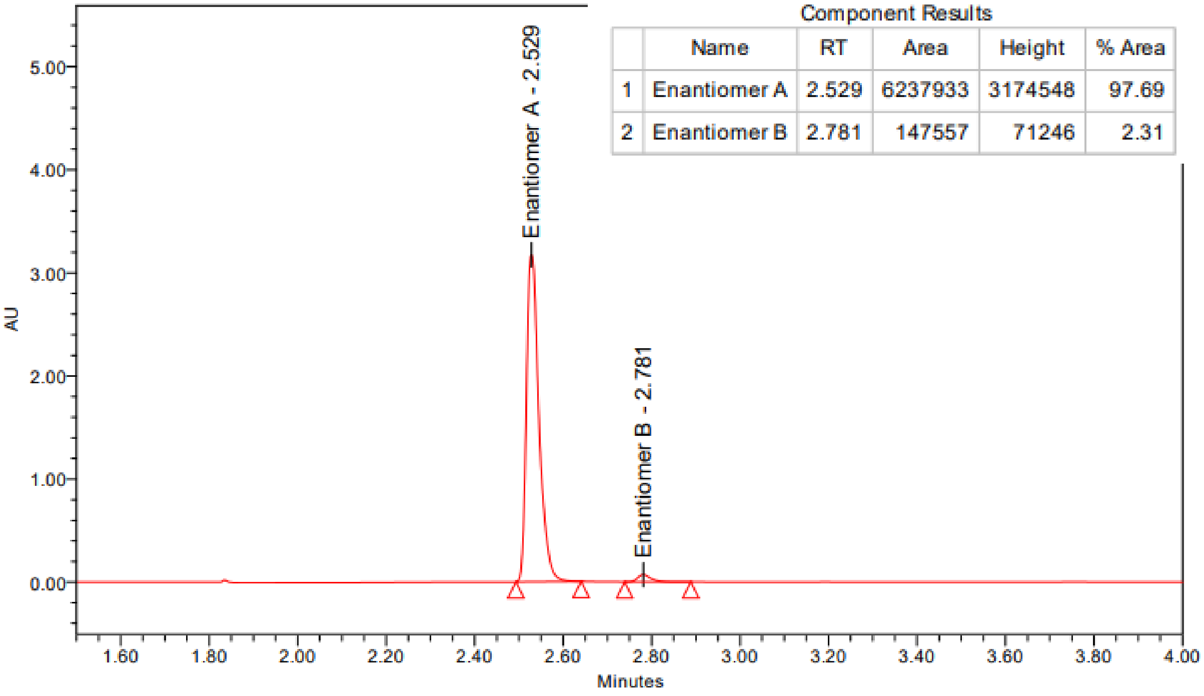

Machine: Waters MDLC; Column: Daicel CHIRALPAK IC-U (3.0 x 100 mm), 1.6 µm; Eluent: MeCN:H_2O = 90:10, isocratic 0.5 mL/min, 303 K. Detection : 254 nm.

Racemic-1d

Component Results

	Name	RT	Area	Height	% Area
1	Enantiomer A	3.096	1405578	575031	50.20
2	Enantiomer B	3.713	1394508	423224	49.80

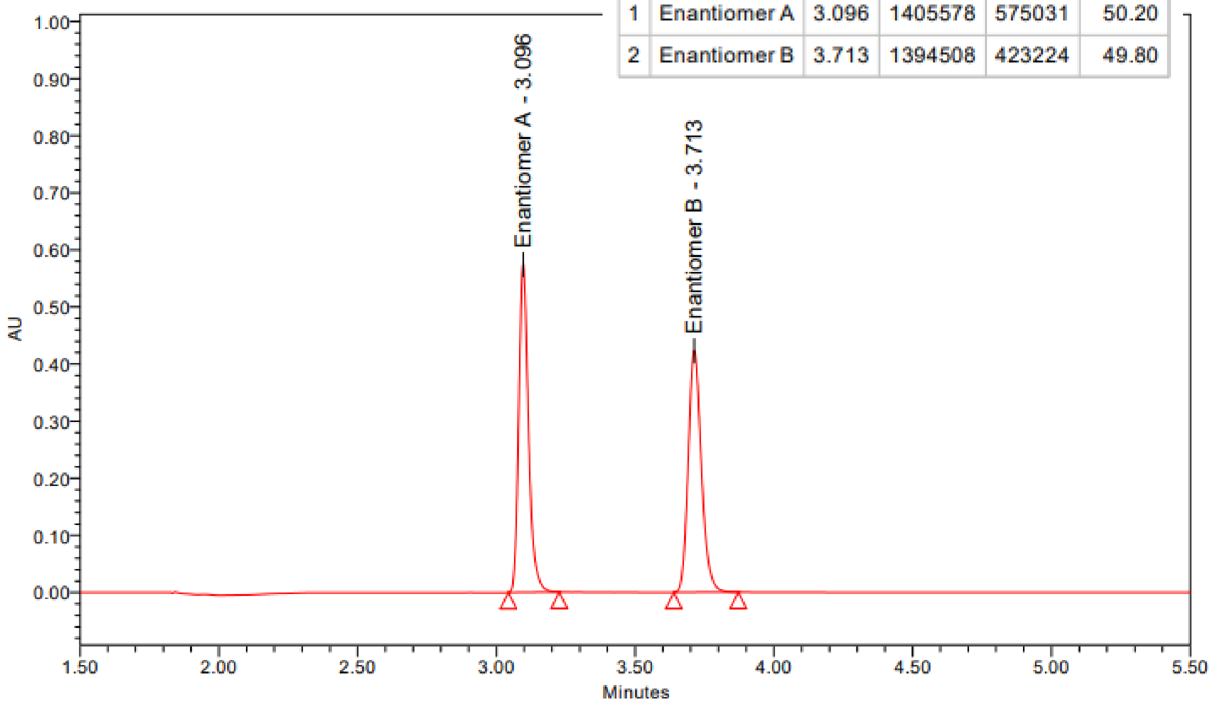

Enantioenriched-1d

Component Results

	Name	RT	Area	Height	% Area
1	Enantiomer A	3.098	6646417	2662825	96.45
2	Enantiomer B	3.712	244970	73332	3.55

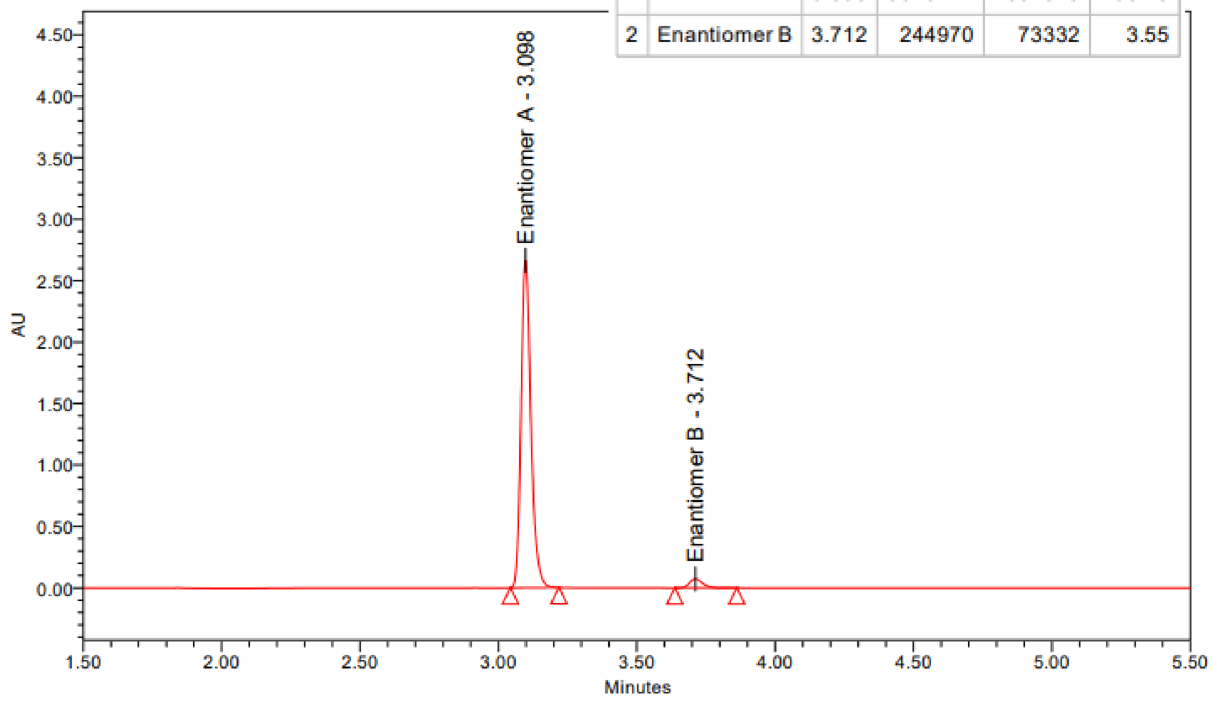

Machine: Waters MDLC; Column: Daicel CHIRALPAK IC-U (3.0 x 100 mm), 1.6 µm; Eluent: MeCN:H_2O = 90:10, isocratic 0.5 mL/min, 303 K. Detection : 254 nm.

Racemic-1e

Component Results

	Name	RT	Area	Height	% Area
1	Enantiomer A	2.784	2408073	1173059	49.90
2	Enantiomer B	3.203	2418050	943701	50.10

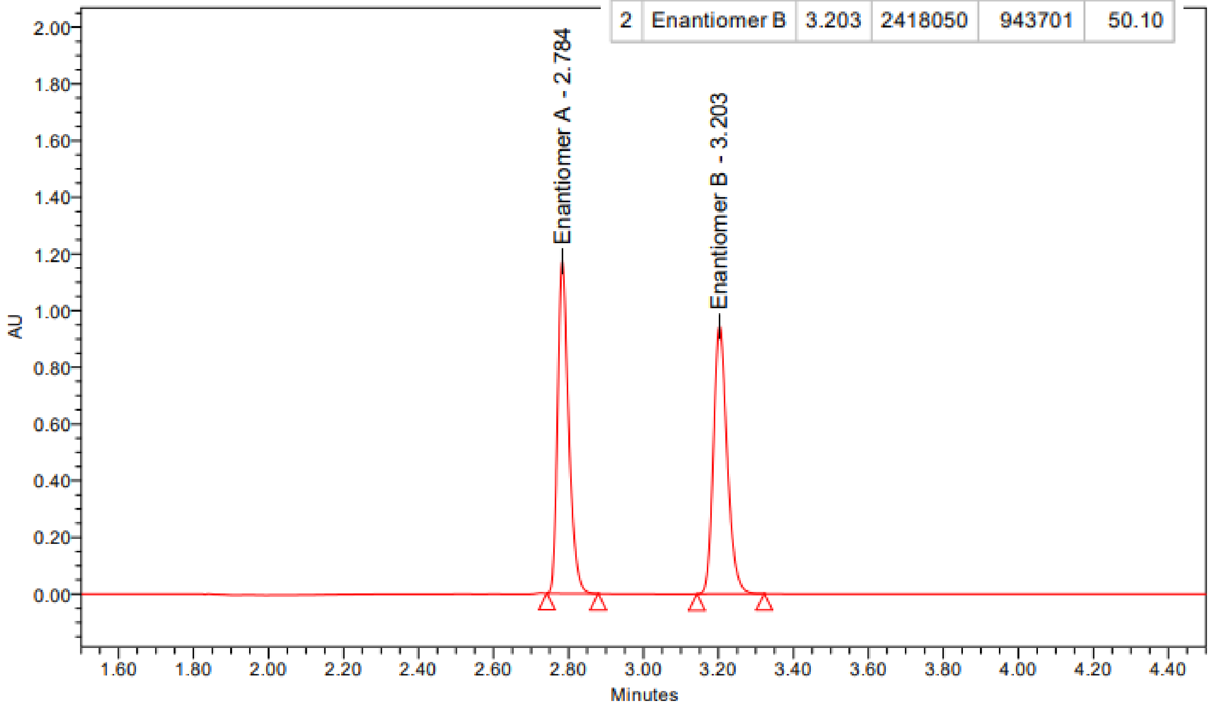

Enantioenriched-1e

Component Results

	Name	RT	Area	Height	% Area
1	Enantiomer A	2.786	4963500	2430303	95.66
2	Enantiomer B	3.205	225230	86896	4.34

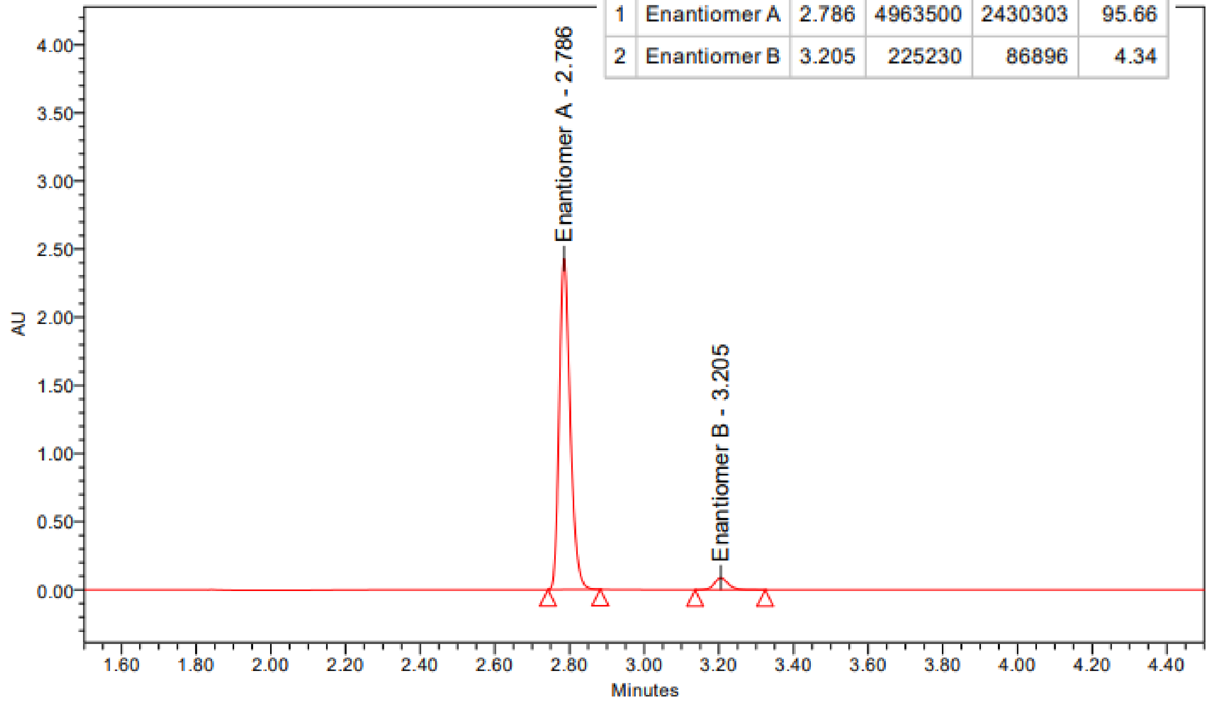

Machine: Waters MDLC; Column: Daicel CHIRALPAK IC-U (3.0 x 100 mm), 1.6 µm; Eluent: MeCN:H_2O = 90:10, isocratic 0.5 mL/min, 303 K. Detection : 254 nm.

Racemic-1f

Component Results

	Name	RT	Area	Height	% Area
1	Enantiomer A	2.977	613816	262878	50.19
2	Enantiomer B	3.385	609227	205193	49.81

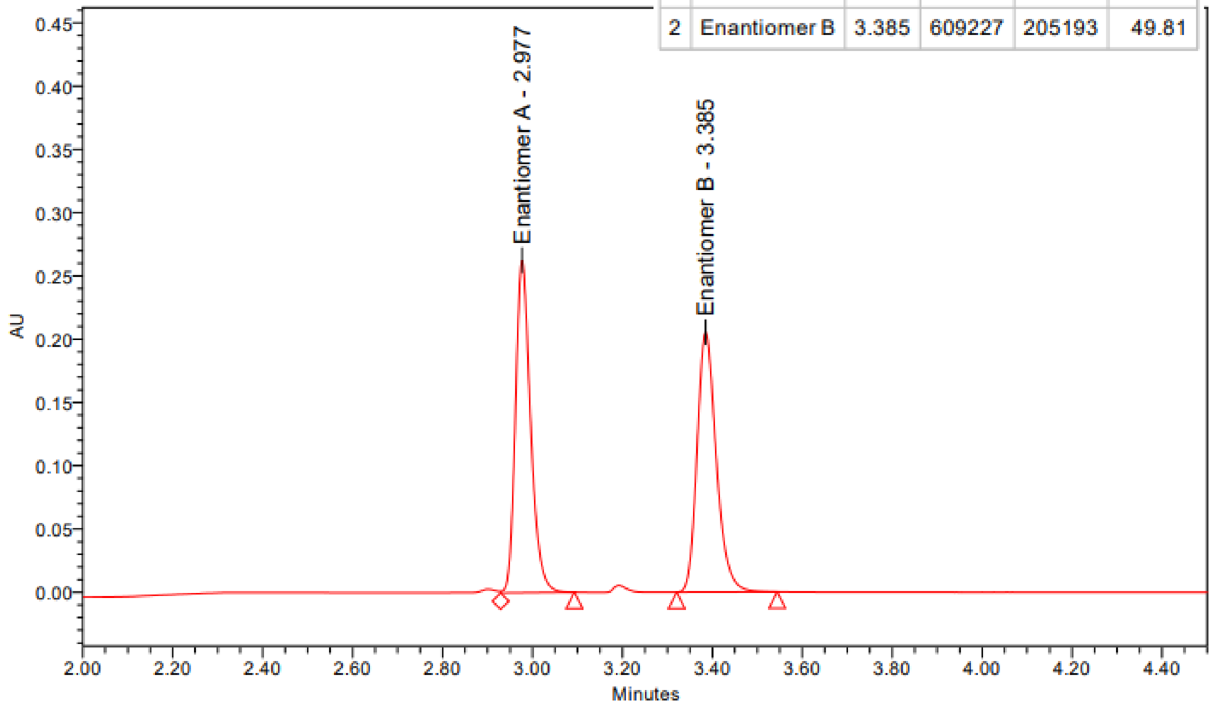

Enantioenriched-1f

Component Results

	Name	RT	Area	Height	% Area
1	Enantiomer A	2.975	2937830	1282734	99.61
2	Enantiomer B	3.382	11456	3952	0.39

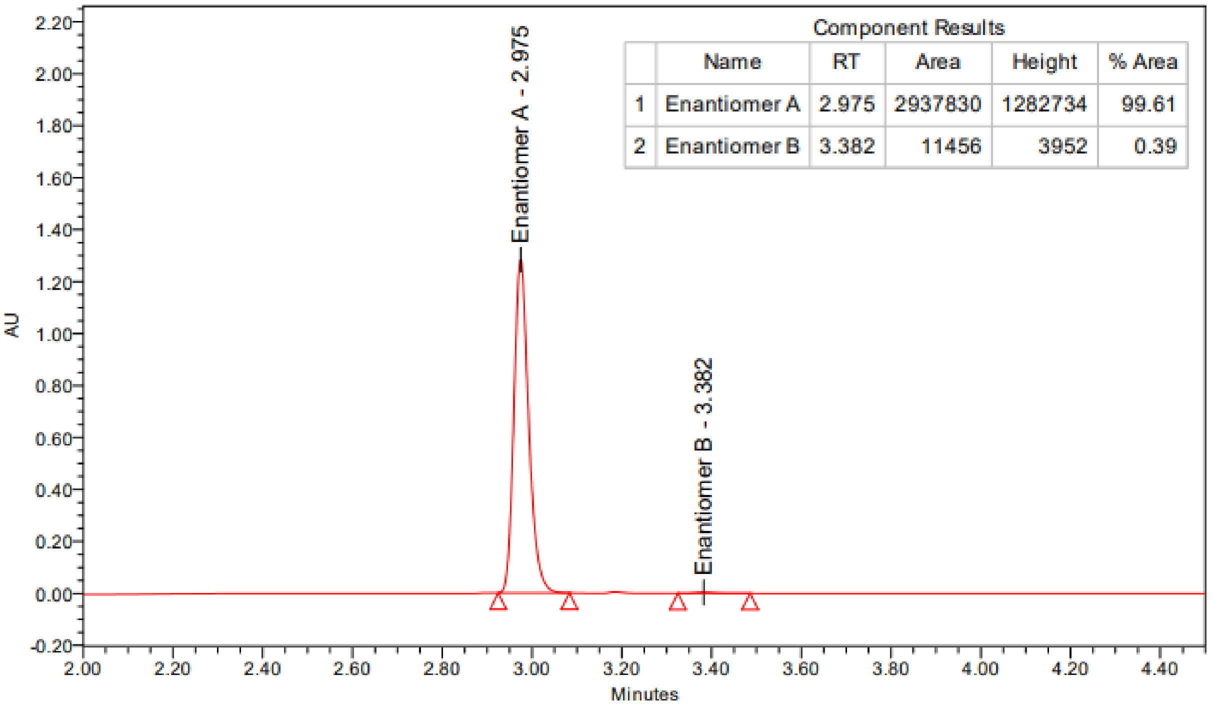

Machine: Waters MDLC; Column: Daicel CHIRALPAK IC-U (3.0 x 100 mm), 1.6 µm; Eluent: MeCN:H_2O = 90:10, isocratic 0.5 mL/min, 303 K. Detection : 254 nm.

Racemic-1g

Component Results

	Name	RT	Area	Height	% Area
1	Enantiomer A	3.025	407550	178085	50.01
2	Enantiomer B	3.555	407388	134836	49.99

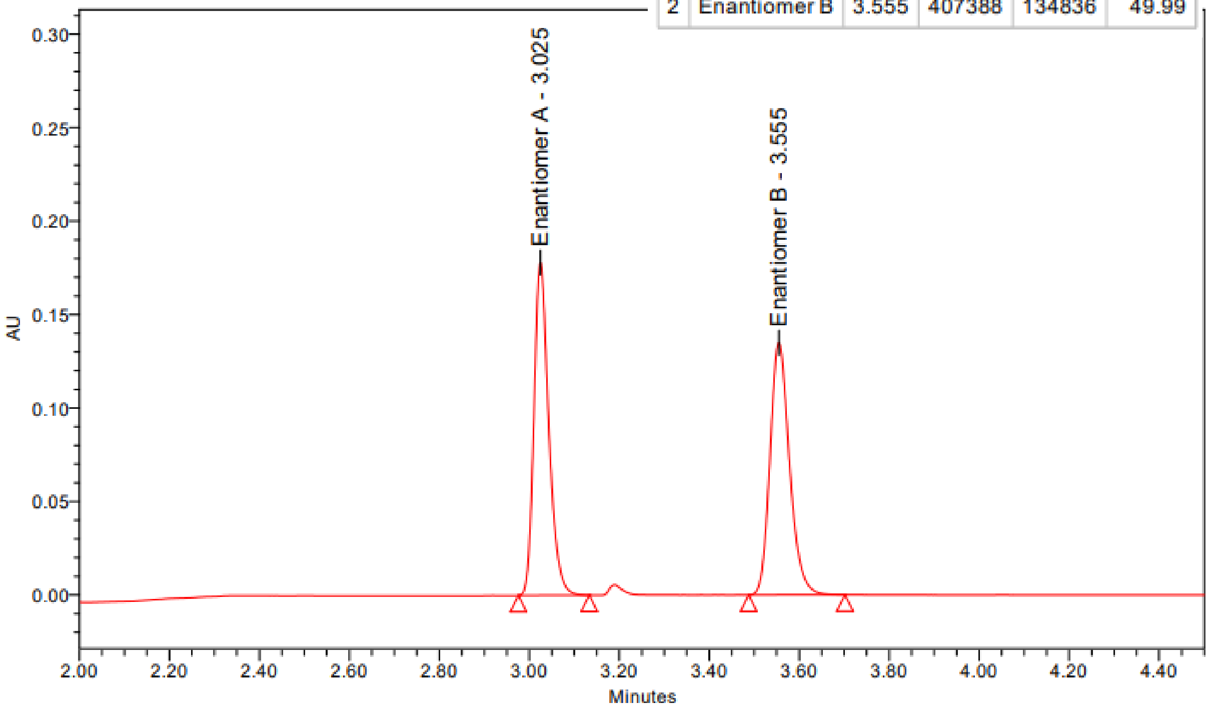

Enantioenriched-1g

Component Results

	Name	RT	Area	Height	% Area
1	Enantiomer A	3.034	1299012	561678	94.09
2	Enantiomer B	3.568	81641	27001	5.91

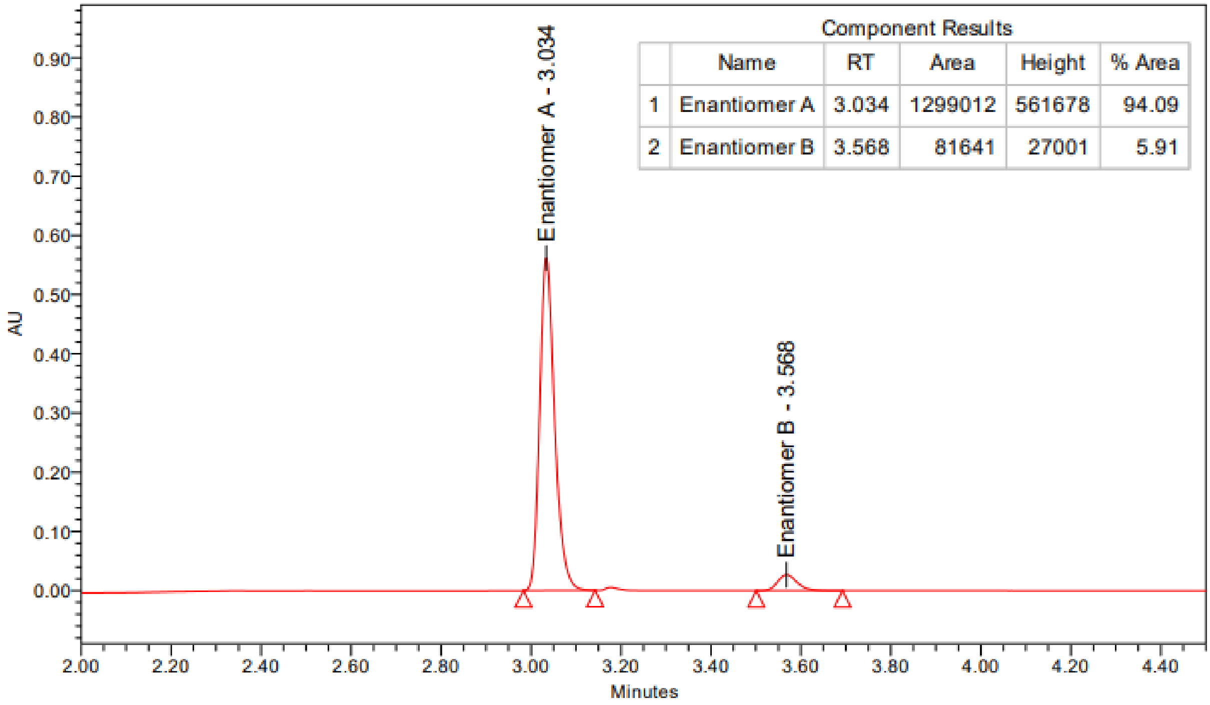

Machine: Waters MDLC; Column: Daicel CHIRALPAK IC-U (3.0 x 100 mm), 1.6 µm; Eluent: MeCN:H_2O = 90:10, isocratic 0.5 mL/min, 303 K. Detection : 254 nm.

Racemic-1h

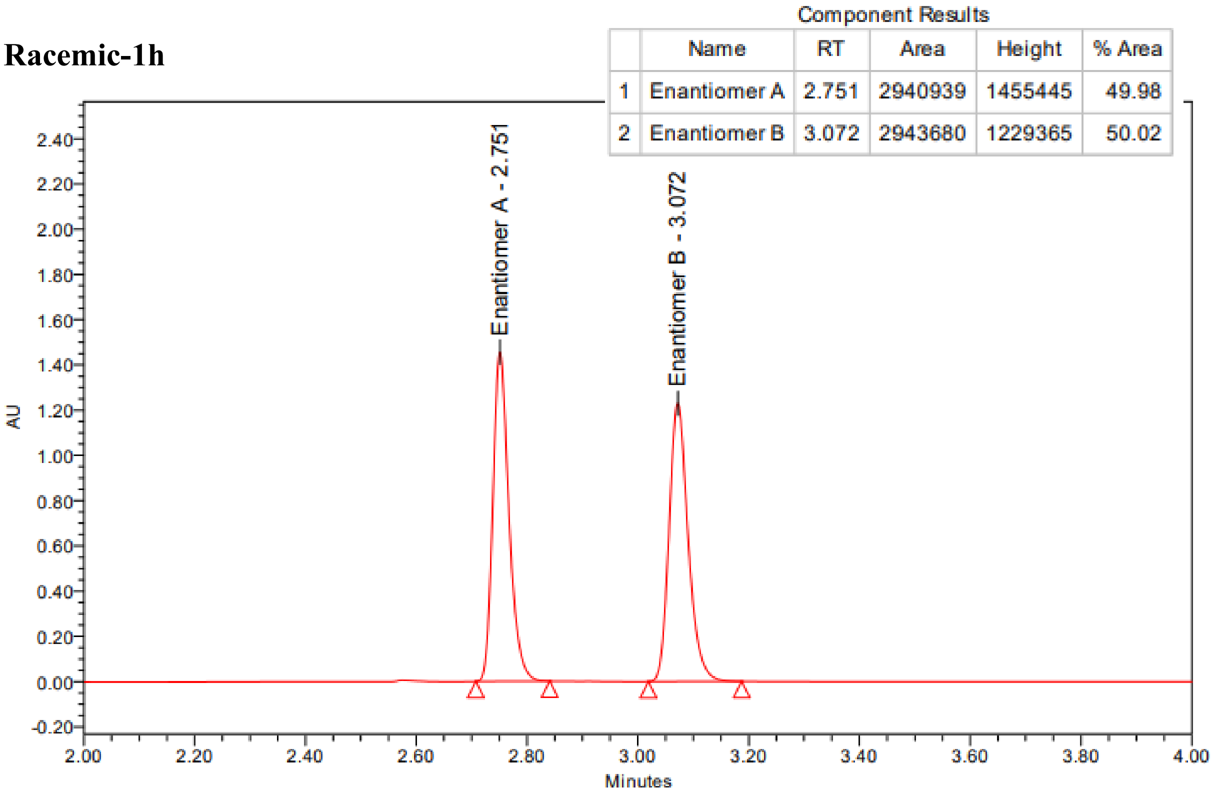

Component Results

	Name	RT	Area	Height	% Area
1	Enantiomer A	2.751	2940939	1455445	49.98
2	Enantiomer B	3.072	2943680	1229365	50.02

Enantioenriched-1h

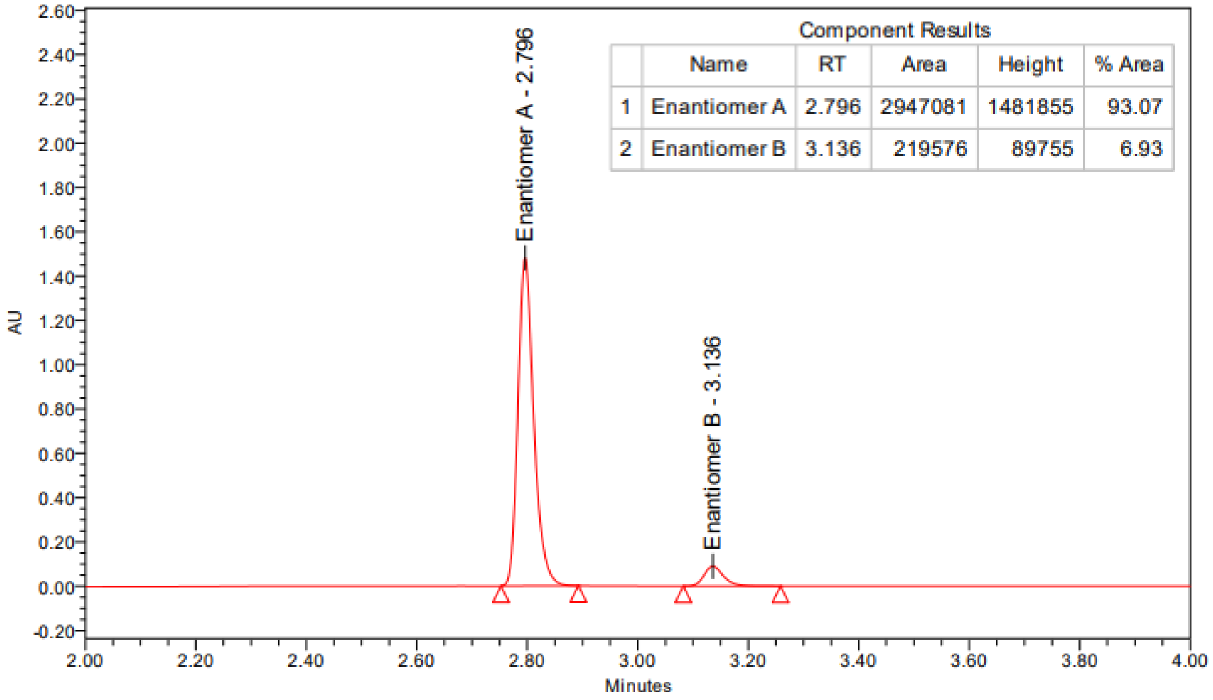

Component Results

	Name	RT	Area	Height	% Area
1	Enantiomer A	2.796	2947081	1481855	93.07
2	Enantiomer B	3.136	219576	89755	6.93

Machine: Waters MDLC; Column: Daicel CHIRALPAK IG-U (3.0 x 100 mm), 1.6 µm; Eluent: MeCN:H_2O = 80:20, isocratic 0.5 mL/min, 303 K. Detection : 254 nm.

Racemic-1i

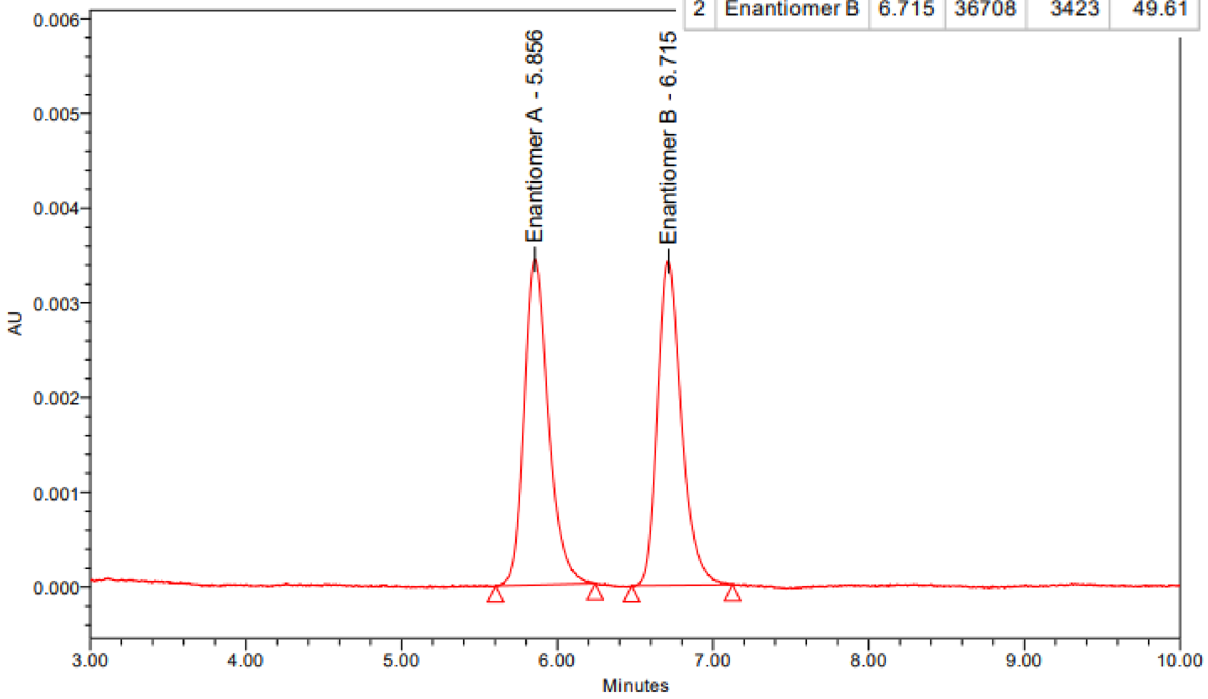

Component Results

	Name	RT	Area	Height	% Area
1	Enantiomer A	5.856	37284	3439	50.39
2	Enantiomer B	6.715	36708	3423	49.61

Enantioenriched-1i

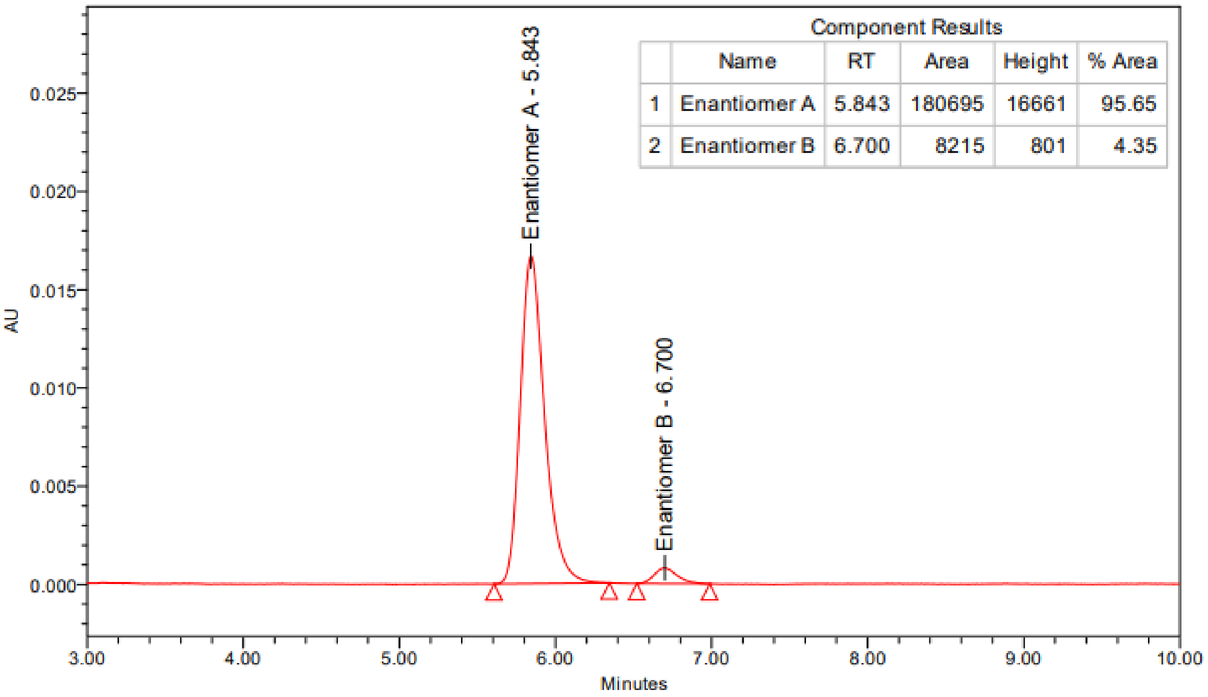

Component Results

	Name	RT	Area	Height	% Area
1	Enantiomer A	5.843	180695	16661	95.65
2	Enantiomer B	6.700	8215	801	4.35

Machine: Waters MDLC; Column: Daicel CHIRALPAK IC-U (3.0 x 100 mm), 1.6 µm; Eluent: MeCN:H_2O = 80:20, isocratic 0.5 mL/min, 303 K. Detection : 254 nm.

Racemic-1j

Component Results

	Name	RT	Area	Height	% Area
1	Enantiomer A	4.427	205464	51415	50.11
2	Enantiomer B	5.022	204565	43008	49.89

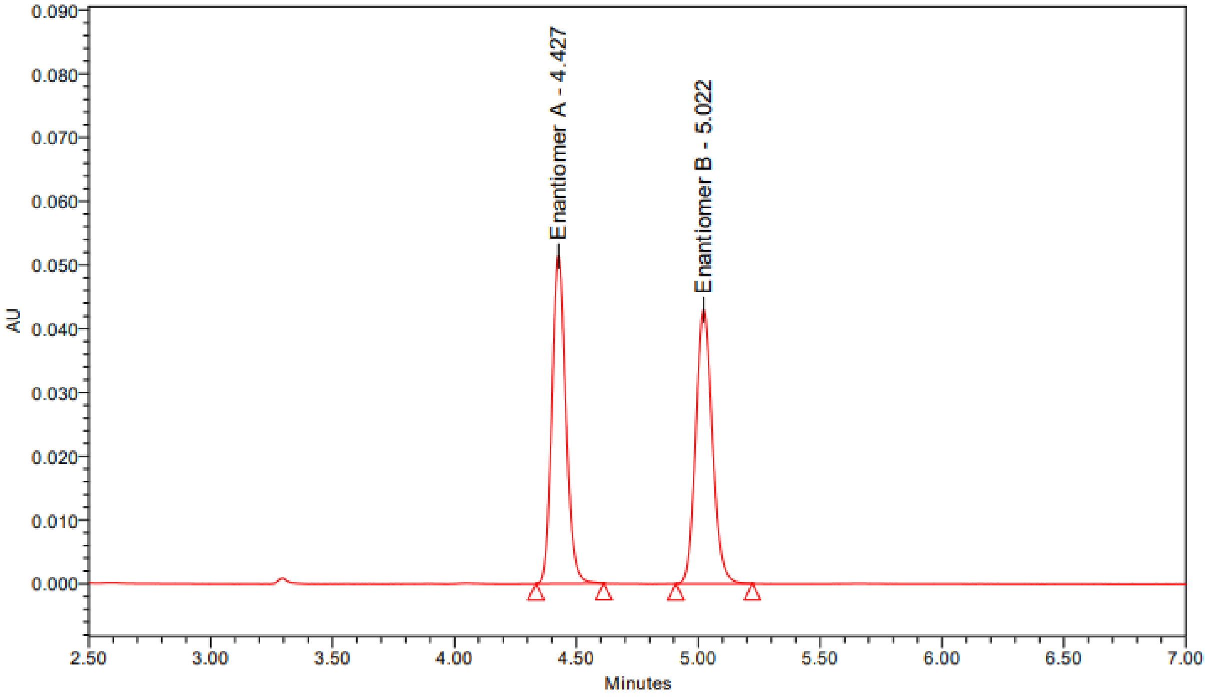

Enantioenriched-1j

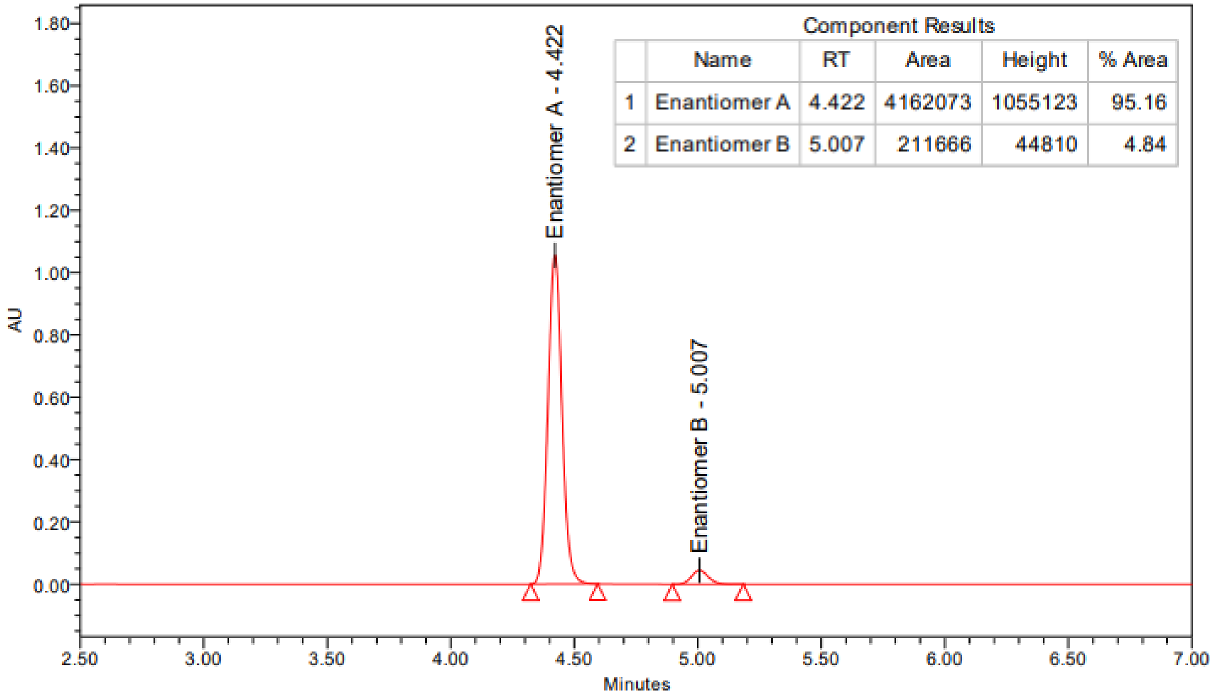

Component Results

	Name	RT	Area	Height	% Area
1	Enantiomer A	4.422	4162073	1055123	95.16
2	Enantiomer B	5.007	211666	44810	4.84

Machine: Waters MDLC; Column: Daicel CHIRALPAK IG-U (3.0 x 100 mm), 1.6 µm; Eluent: MeOH:H_2O = 97:3, isocratic 0.4 mL/min, 303 K. Detection : 254 nm.

Racemic-1b(BPh)

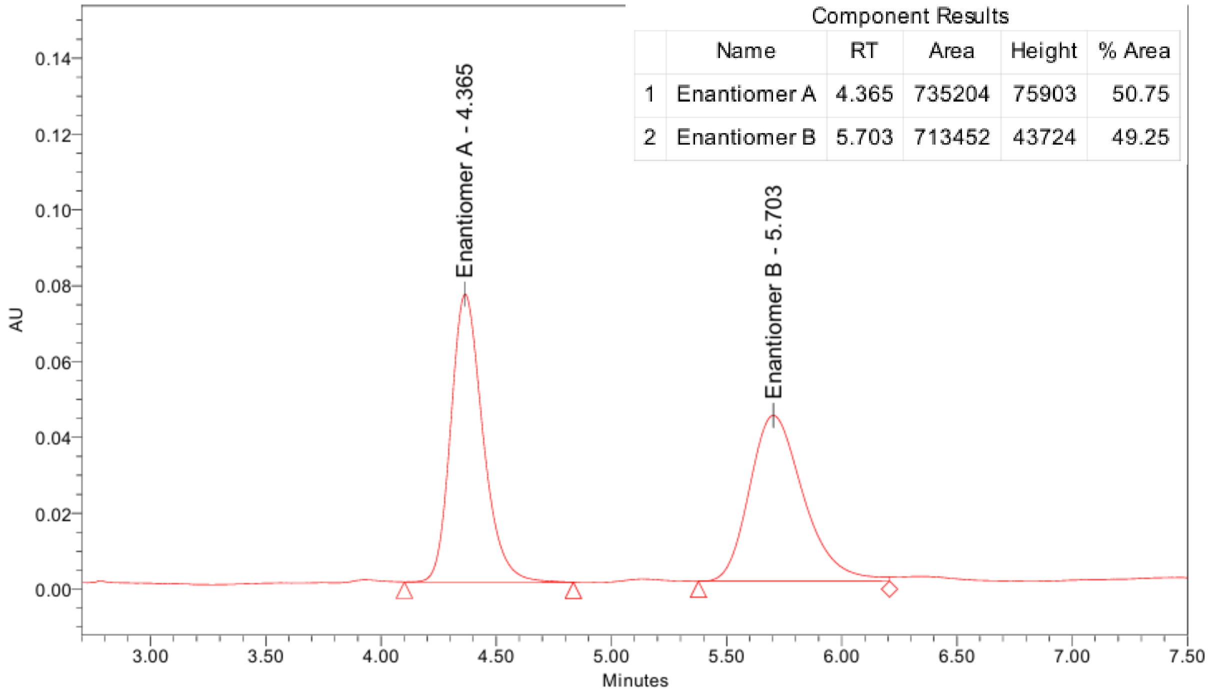

Component Results

	Name	RT	Area	Height	% Area
1	Enantiomer A	4.365	735204	75903	50.75
2	Enantiomer B	5.703	713452	43724	49.25

Enantioenriched-1b(BPh)

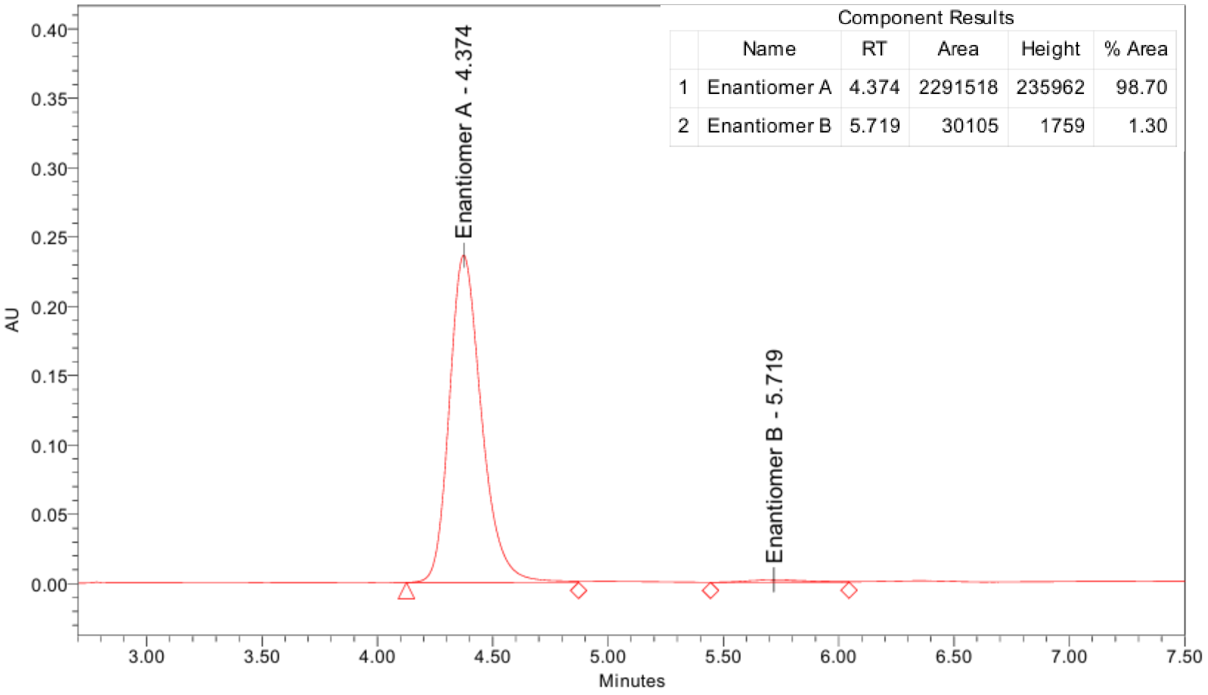

Component Results

	Name	RT	Area	Height	% Area
1	Enantiomer A	4.374	2291518	235962	98.70
2	Enantiomer B	5.719	30105	1759	1.30

Machine: Waters MDLC; Column: Daicel CHIRALPAK IC-U (3.0 x 100 mm), 1.6 µm; Eluent: MeCN:H_2O = 90:10, isocratic 0.5 mL/min, 303 K. Detection : 254 nm.

Racemic-1b(NMe)

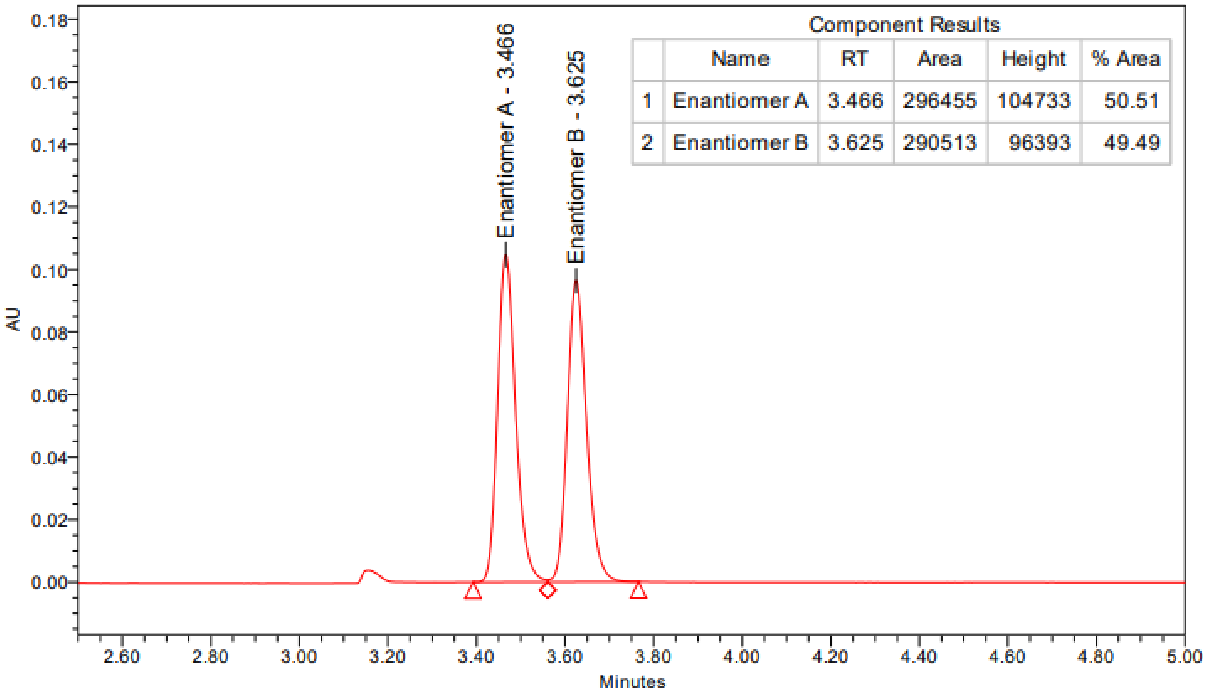

Component Results

	Name	RT	Area	Height	% Area
1	Enantiomer A	3.466	296455	104733	50.51
2	Enantiomer B	3.625	290513	96393	49.49

Enantioenriched-1b(NMe)

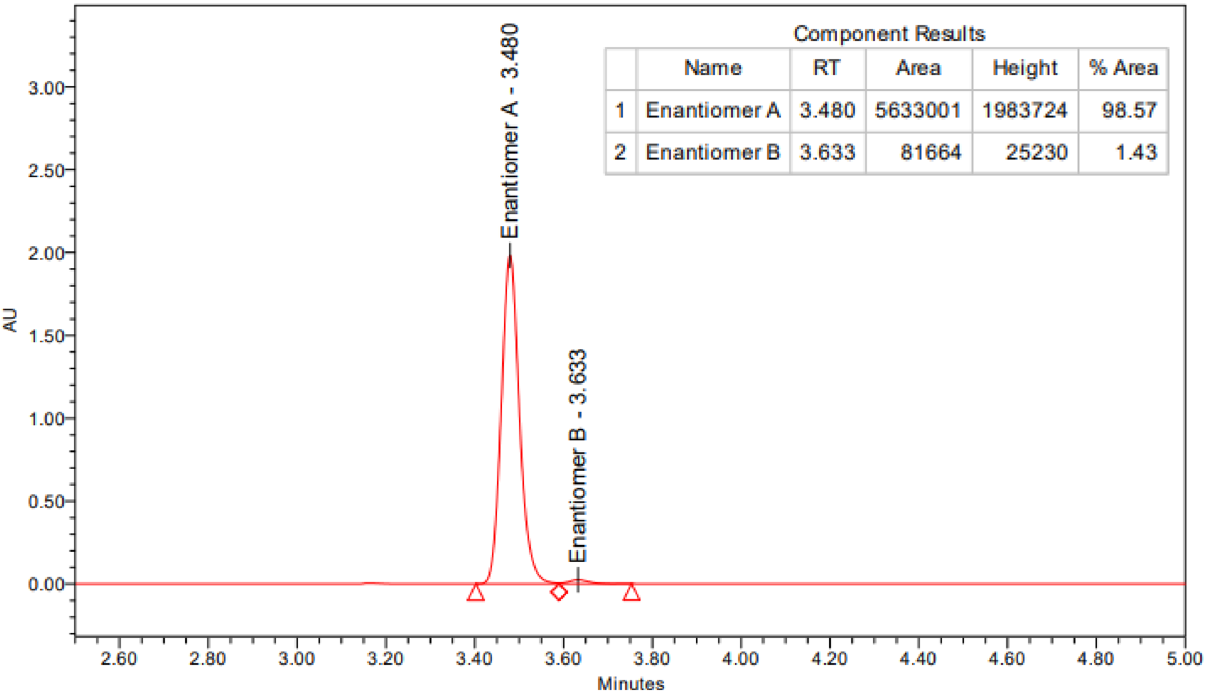

Component Results

	Name	RT	Area	Height	% Area
1	Enantiomer A	3.480	5633001	1983724	98.57
2	Enantiomer B	3.633	81664	25230	1.43

Machine: Waters MDLC, Column: Daicel CHIRALPAK IG-U (3.0 x 100 mm), 1.6 µm; Eluent: MeCN:H_2O = 80:20, isocratic 0.5 mL/min, 303 K. Detection : 254 nm.

Racemic-2b

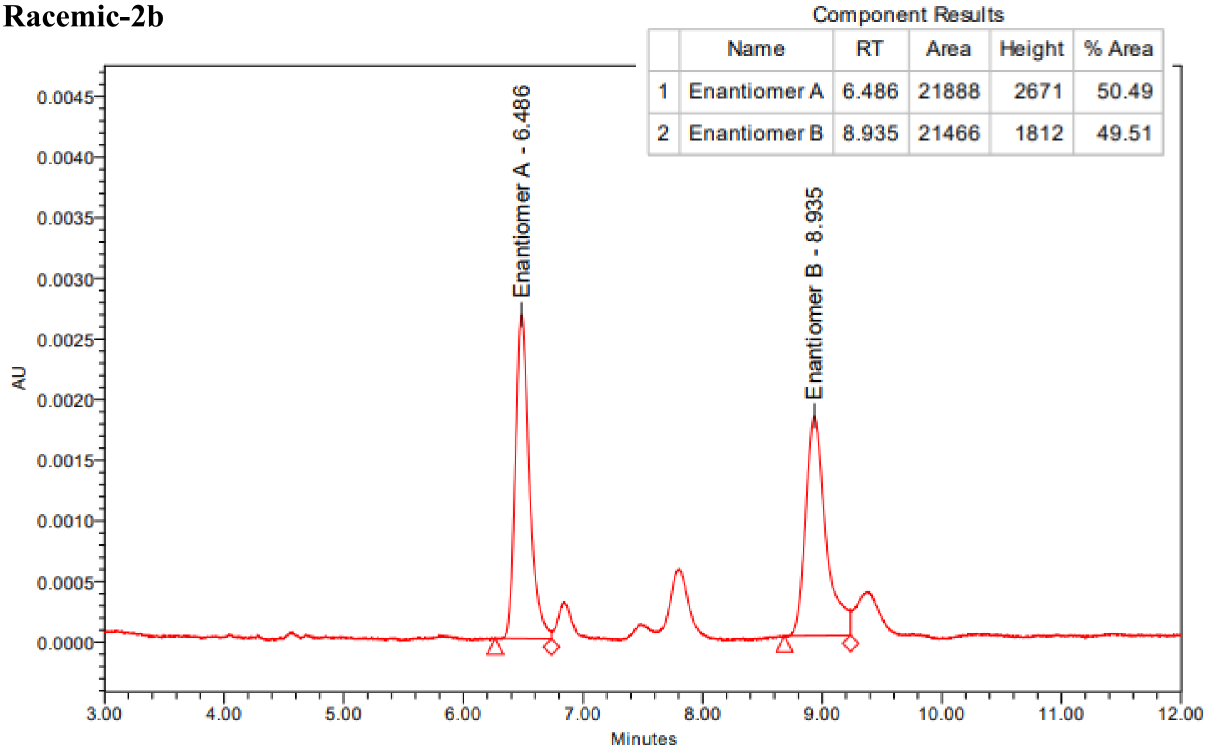

Component Results

	Name	RT	Area	Height	% Area
1	Enantiomer A	6.486	21888	2671	50.49
2	Enantiomer B	8.935	21466	1812	49.51

Enantioenriched-2b

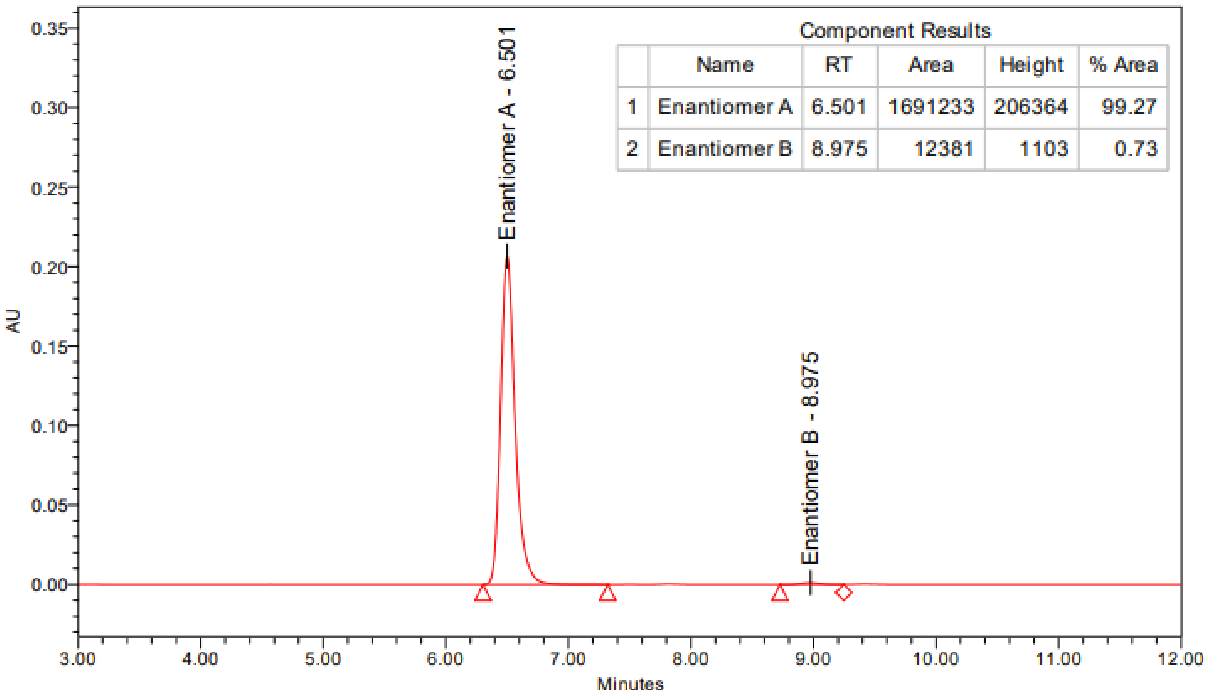

Component Results

	Name	RT	Area	Height	% Area
1	Enantiomer A	6.501	1691233	206364	99.27
2	Enantiomer B	8.975	12381	1103	0.73

Machine: Waters MDLC; Column: Daicel CHIRALPAK IG-U (3.0 x 100 mm), 1.6 µm; Eluent: MeCN:H_2O = 80:20, isocratic, 0.5 mL/min, 303 K. Detection : 254 nm.

Racemic-2c

Peak Results

	Name	RT	% Area	Area	Height
1	Enantiomer A	5.695	50.22	34503	4814
2	Enantiomer B	7.843	49.78	34195	3108

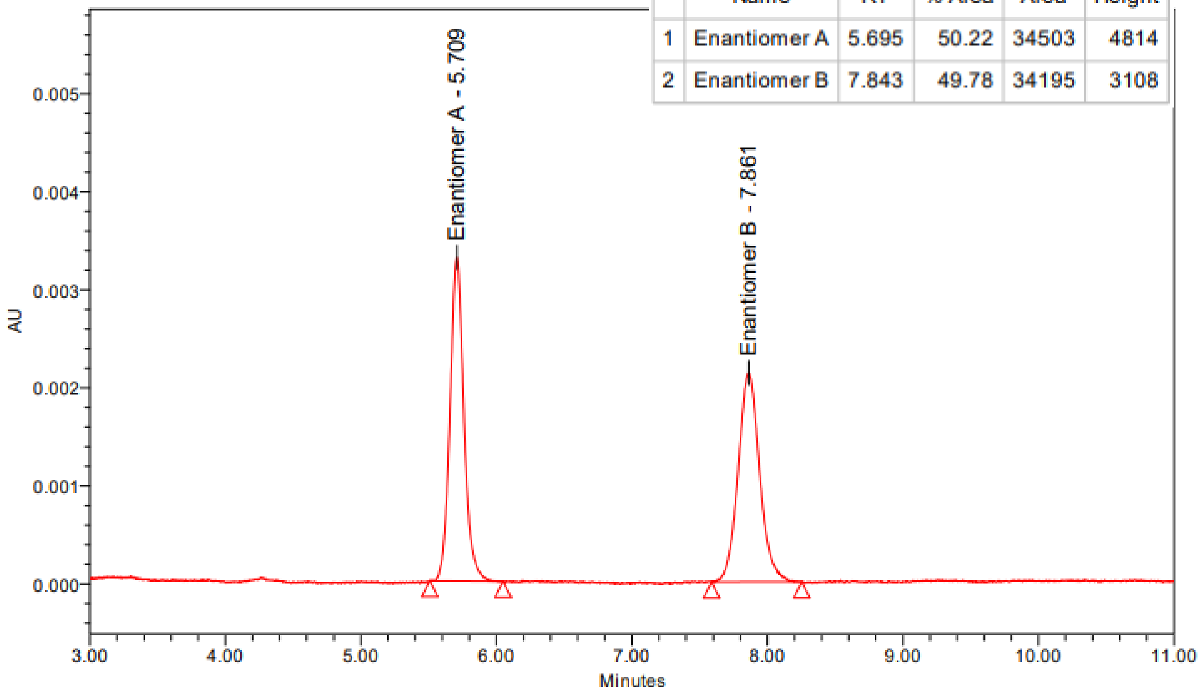

Enantioenriched-2c

Peak Results

	Name	RT	% Area	Area	Height
1	Enantiomer A	5.938	92.14	331351	44676
2	Enantiomer B	8.235	7.86	28248	2476

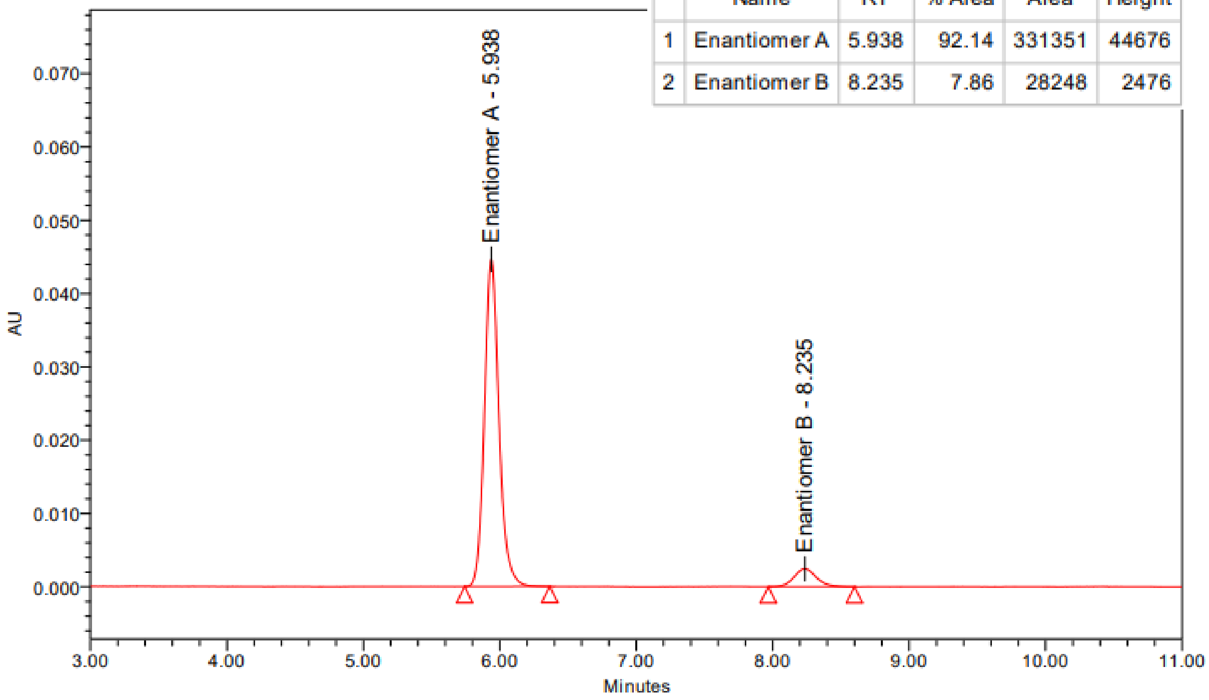

Machine: Waters MDLC; Column: Daicel CHIRALPAK IG-U (3.0 x 100 mm), 1.6 µm; Eluent: MeCN:H_2O = 85:15, isocratic 0.5 mL/min, 303 K. Detection : 254 nm.

Racemic-2e

Component Results

	Name	RT	Area	Height	% Area
1	Enantiomer A	4.187	45697	11372	50.69
2	Enantiomer B	4.925	44445	8755	49.31

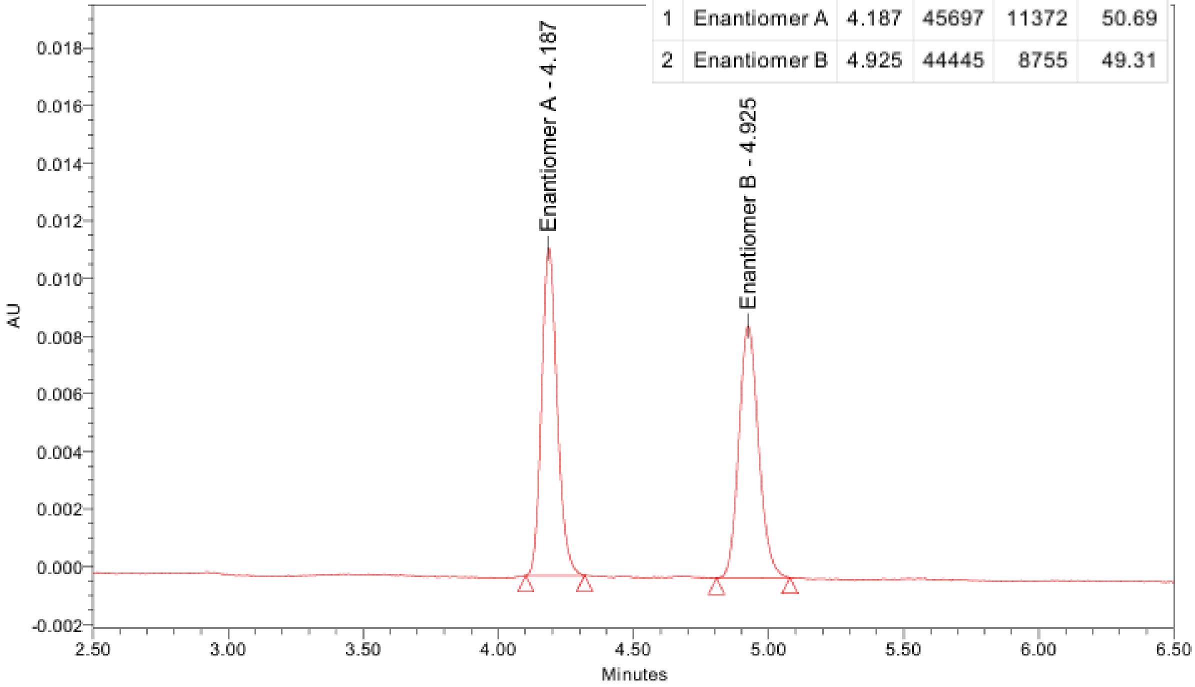

Enantioenriched-2e

Component Results

	Name	RT	Area	Height	% Area
1	Enantiomer A	4.103	748005	188941	98.26
2	Enantiomer B	4.814	13213	2590	1.74

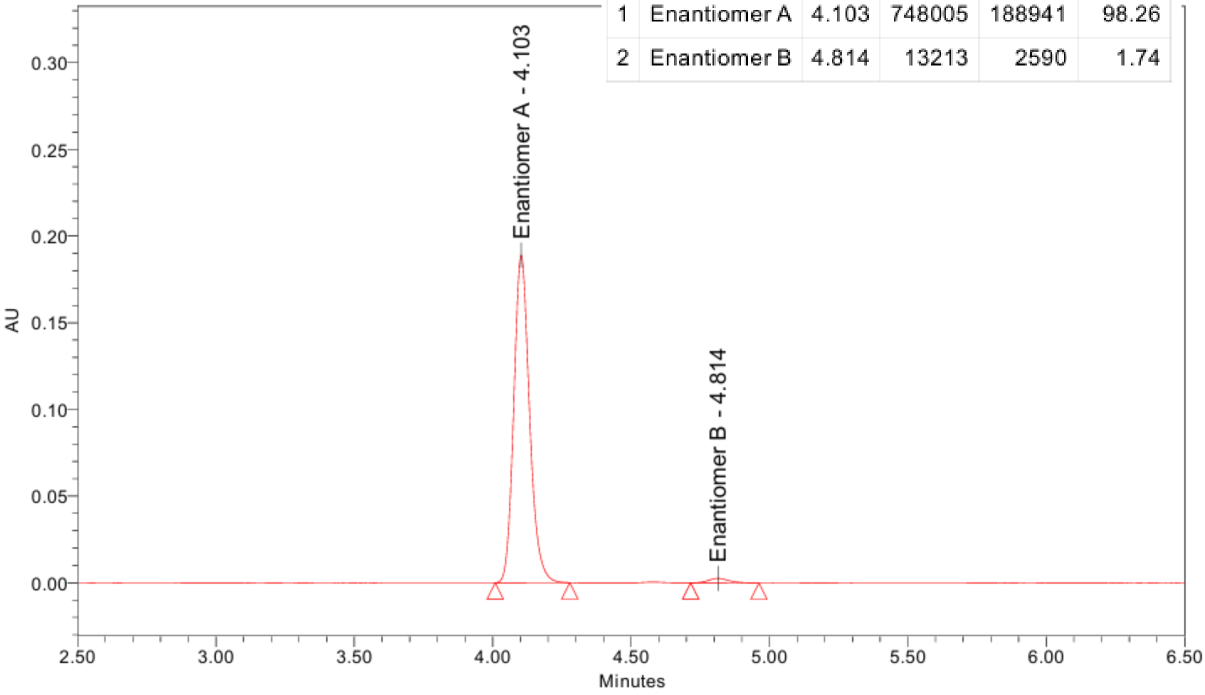

Machine: Waters MDLC; Column: Daicel CHIRALPAK IC-U (3.0 x 100 mm), 1.6 µm; Eluent: MeCN:H_2O = 80:20, isocratic 0.5 mL/min, 303 K. Detection : 320 nm.

Racemic-2h

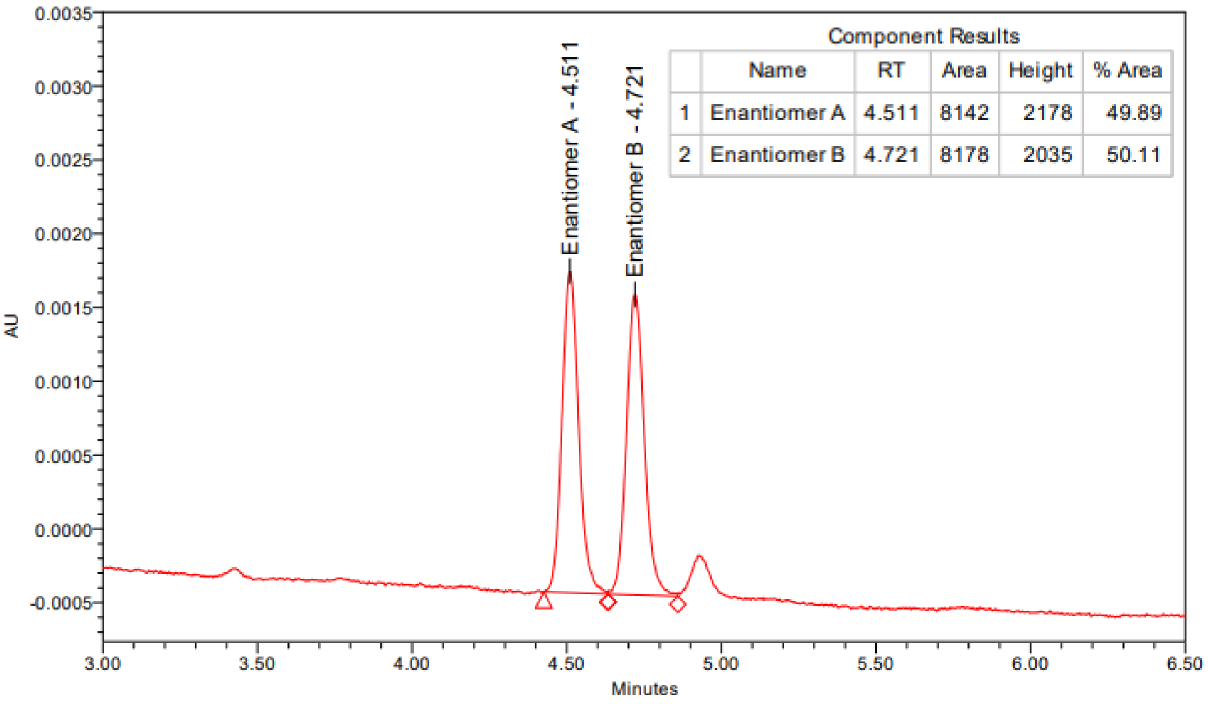

Enantioenriched-2h

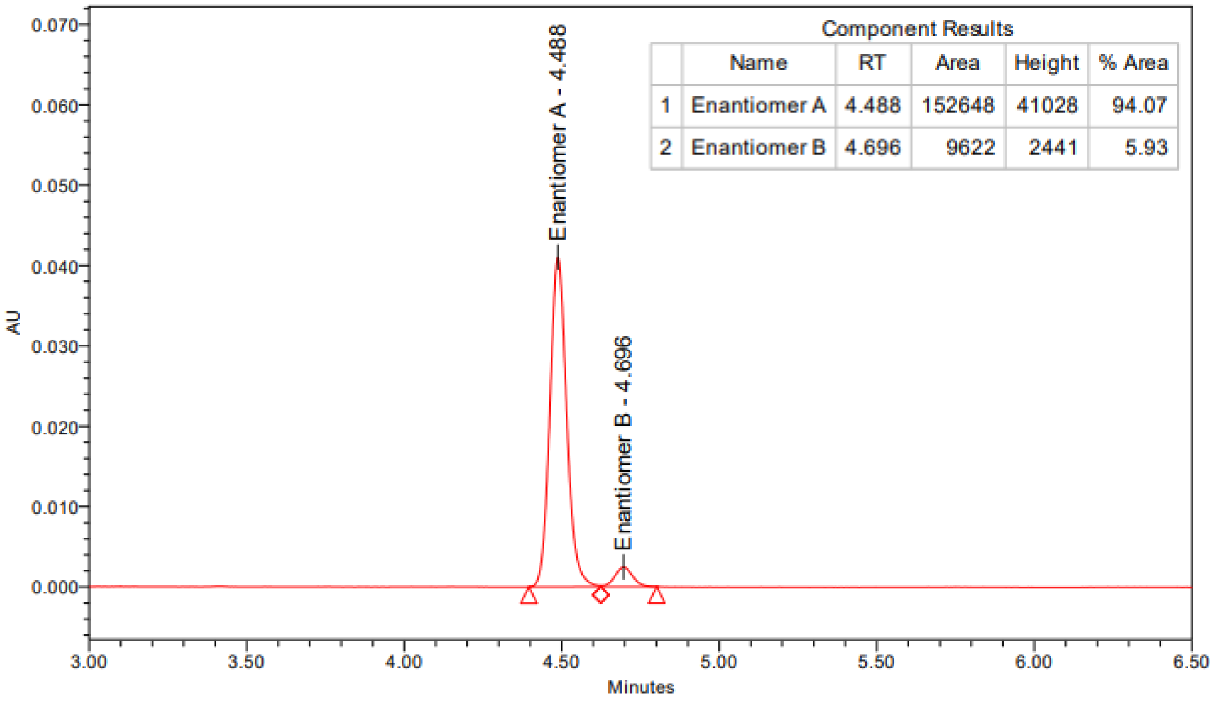

Machine: Waters MDLC; Column: Daicel CHIRALPAK IG-U (3.0 x 100 mm), 1.6 µm; Eluent: MeCN:H_2O = 90:10, isocratic 0.5 mL/min, 303 K. Detection : 254 nm.

Racemic-12b

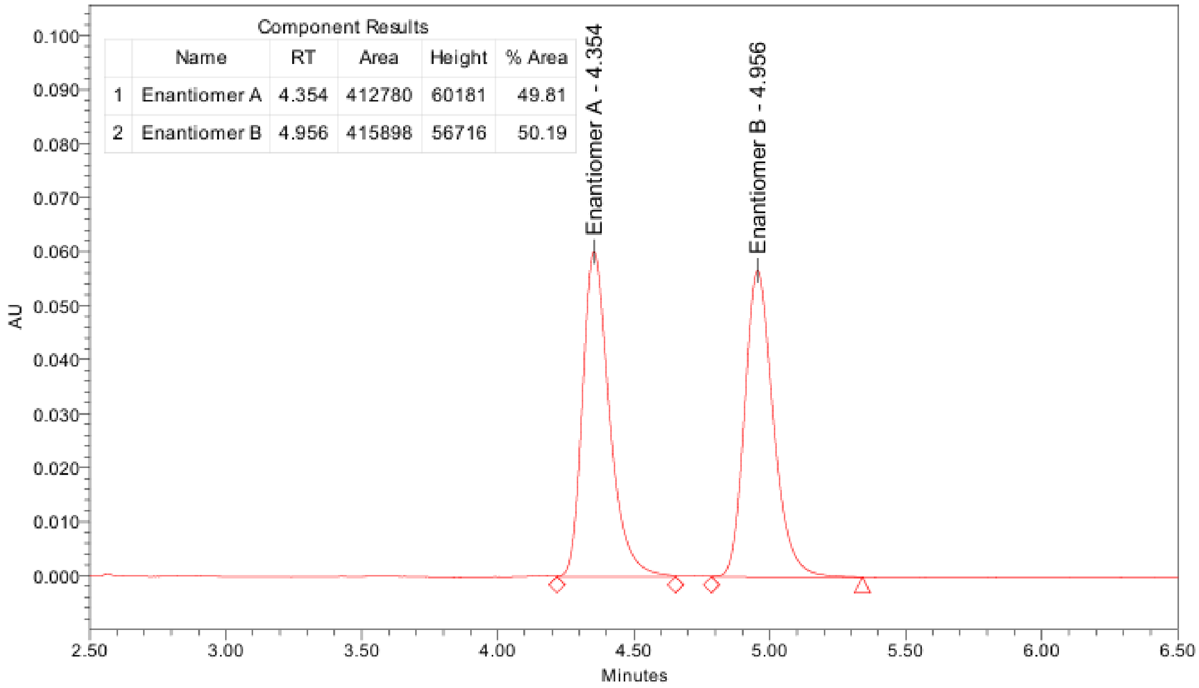

	Name	RT	Area	Height	% Area
1	Enantiomer A	4.354	412780	60181	49.81
2	Enantiomer B	4.956	415898	56716	50.19

Enantioenriched-12b

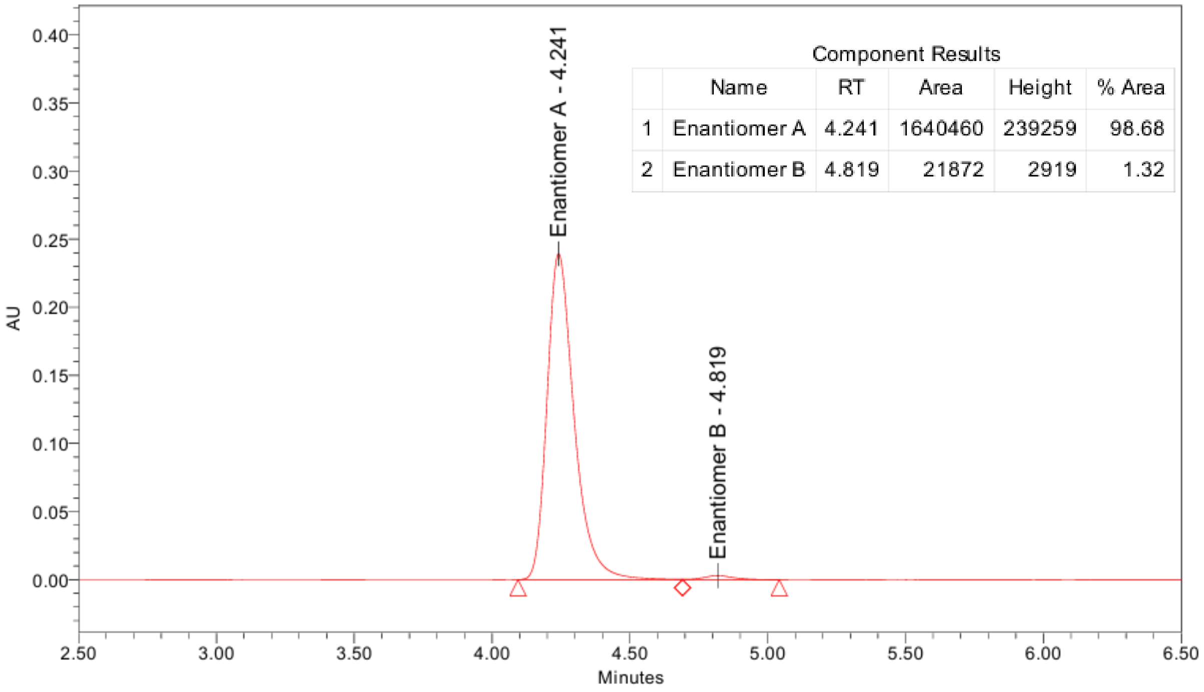

	Name	RT	Area	Height	% Area
1	Enantiomer A	4.241	1640460	239259	98.68
2	Enantiomer B	4.819	21872	2919	1.32

Machine: Waters MDLC; Column: Daicel CHIRALPAK IG-U (3.0 x 100 mm), 1.6 µm; Eluent: MeCN:H_2O = 90:10, isocratic 0.5 mL/min, 303 K. Detection : 254 nm.

Racemic-13b

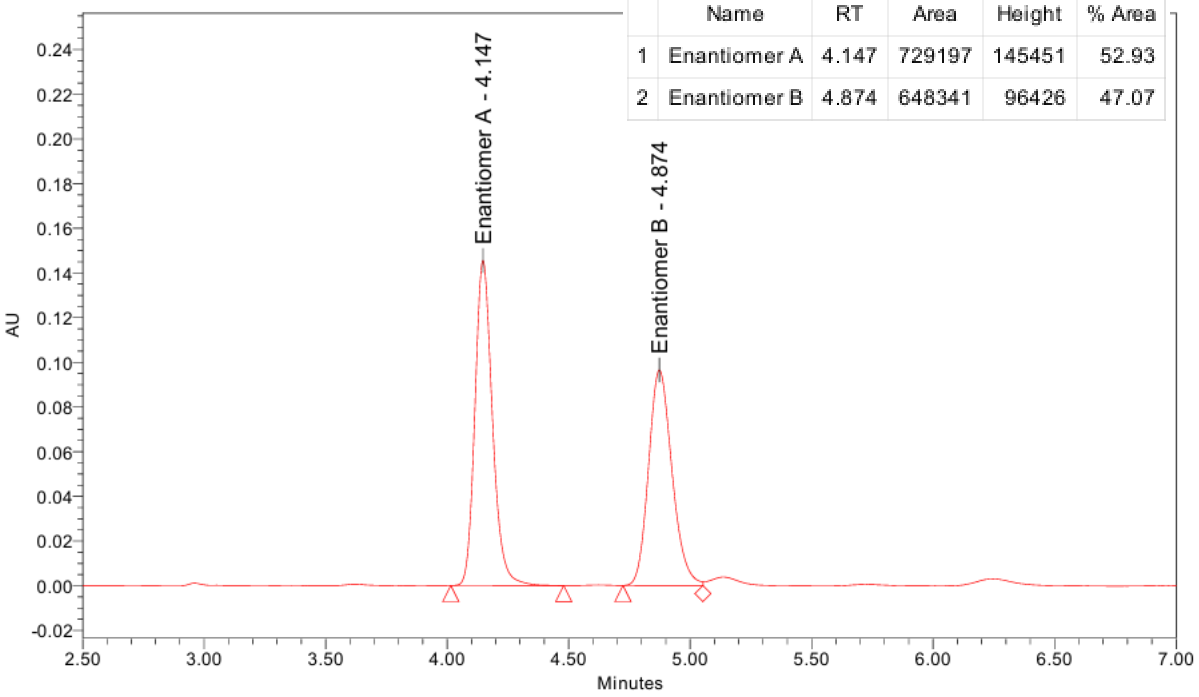

Component Results

	Name	RT	Area	Height	% Area
1	Enantiomer A	4.147	729197	145451	52.93
2	Enantiomer B	4.874	648341	96426	47.07

Enantioenriched-13b

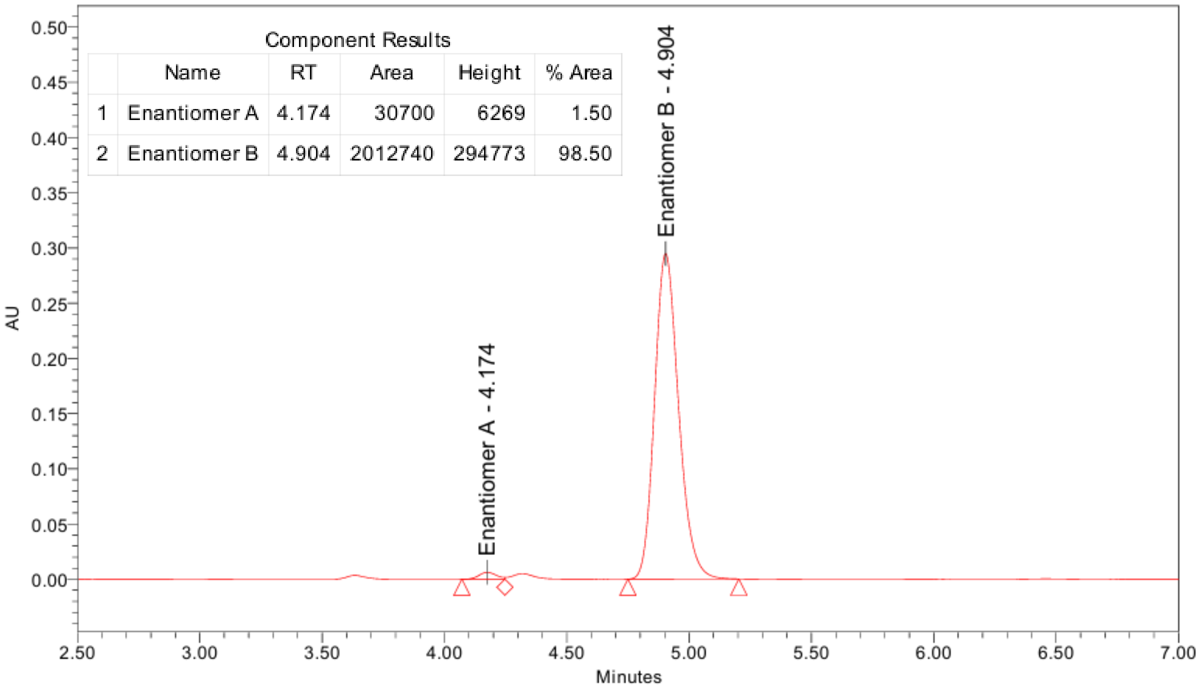

Component Results

	Name	RT	Area	Height	% Area
1	Enantiomer A	4.174	30700	6269	1.50
2	Enantiomer B	4.904	2012740	294773	98.50

5. Electro-Optical Properties

5.1. Absorption (UV-vis) and Fluorescence spectra

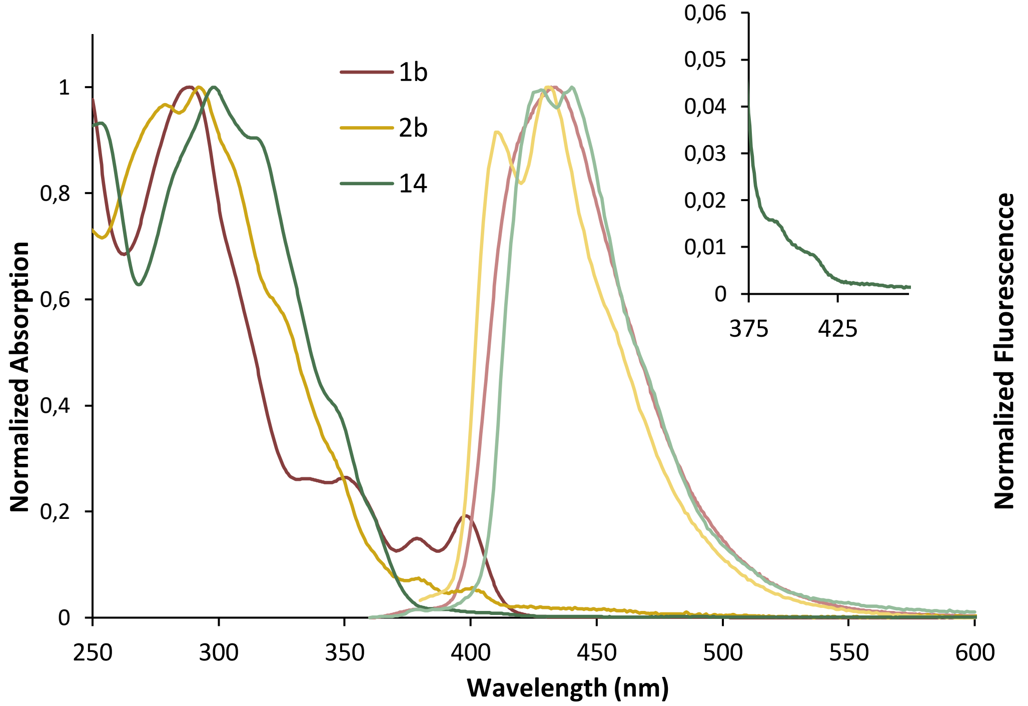

Figure S1 Normalised UV/Vis (solid line) and fluorescence (faded line) spectra of selected compounds measured in CH_2Cl_2 (1×10^{-5} M to 2×10^{-5} M) at r.t. λ_{exc}= 350 nm; λ_{exc}= 370 nm (compound **2b**).

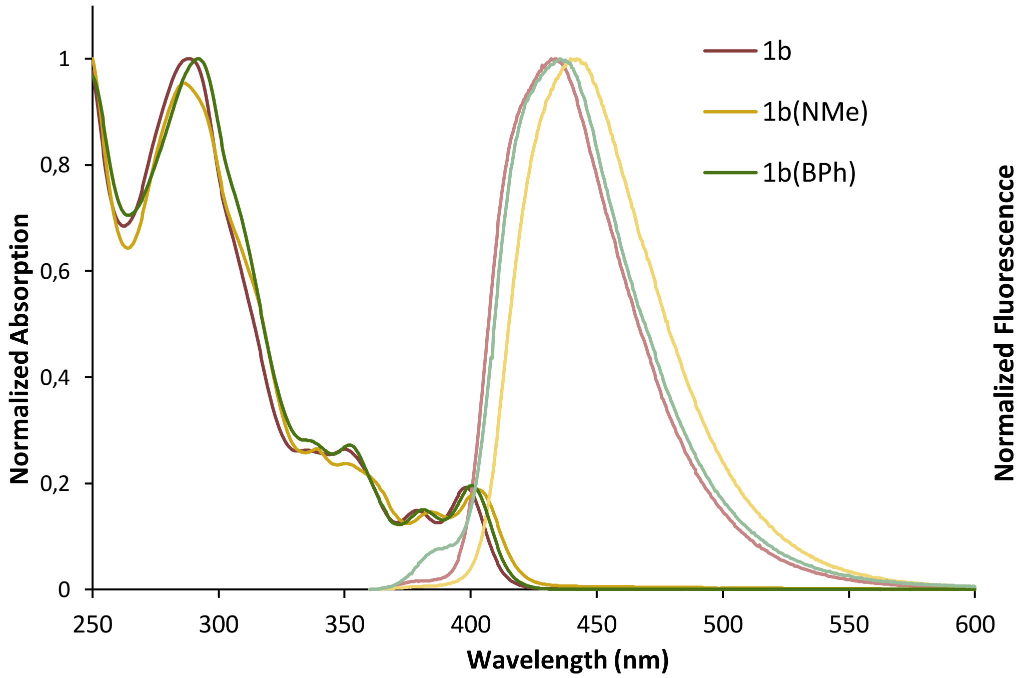

Figure S2 Normalised UV/Vis (solid line) and fluorescence (faded line) spectra of selected compounds measured in CH_2Cl_2 (1×10^{-5} M to 2×10^{-5} M) at r.t. λ_{exc}= 350 nm.

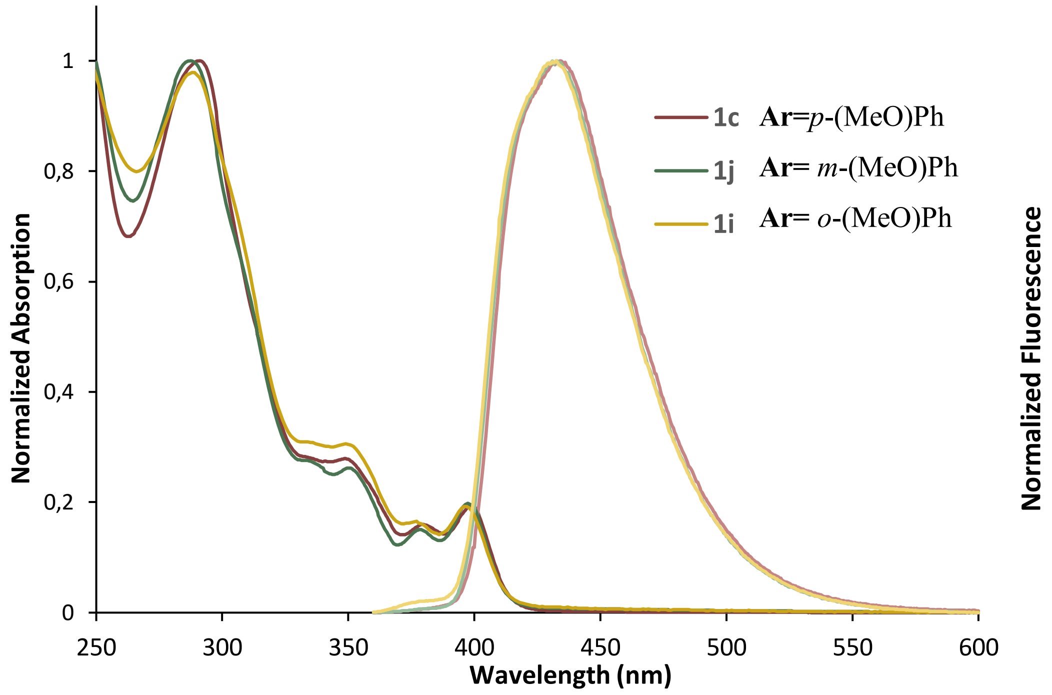

Figure S3 Normalised UV/Vis (solid line) and fluorescence (faded line) spectra of selected compounds measured in CH_2Cl_2 (1×10^{-5} M) at r.t. λ_{exc}= 350 nm.

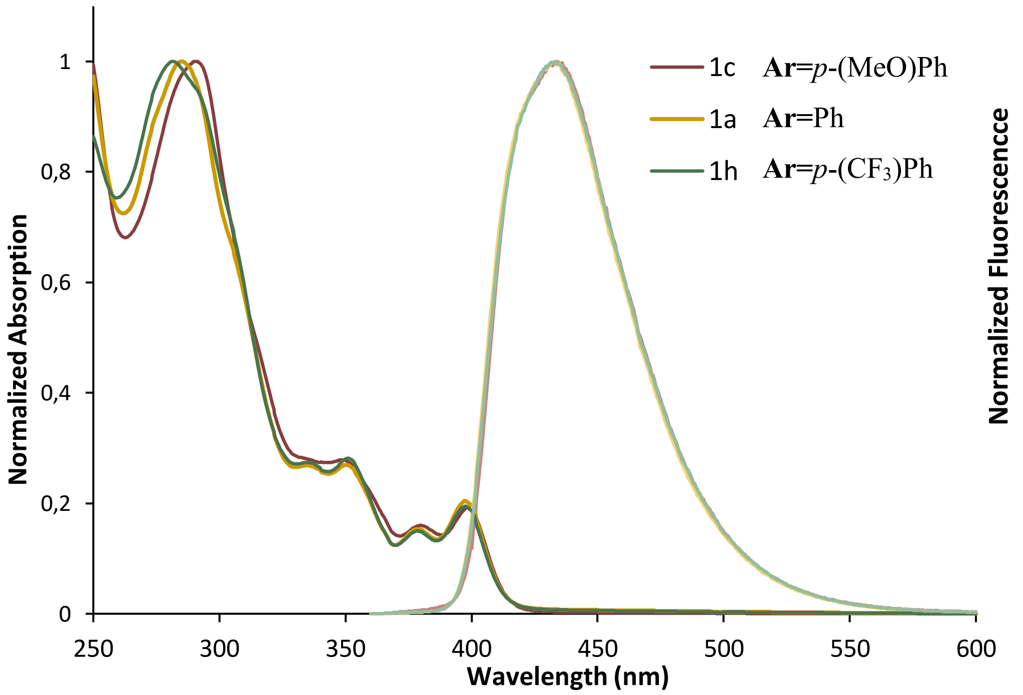

Figure S4 Normalised UV/Vis (solid line) and fluorescence (faded line) spectra of selected compounds measured in CH_2Cl_2 (1×10^{-5} M) at r.t. λ_{exc}= 350 nm.

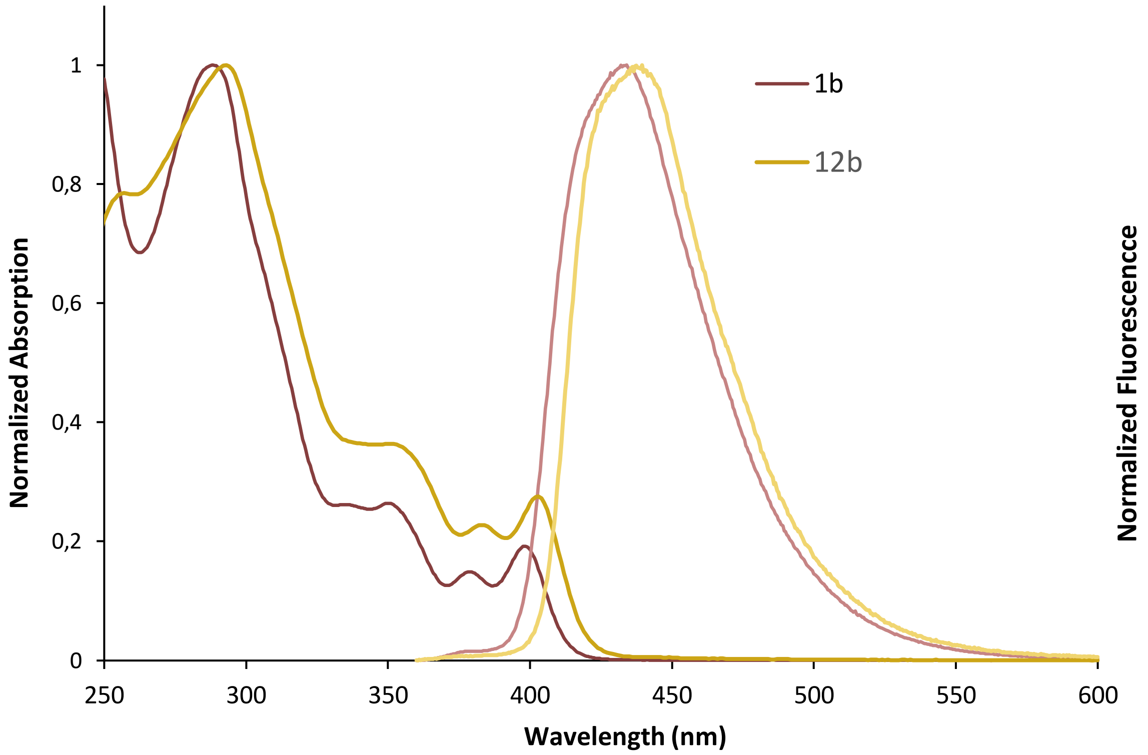

Figure S5 Normalised UV/Vis (solid line) and fluorescence (faded line) spectra of selected compounds measured in CH_2Cl_2 (1×10^{-5} M to 2×10^{-5} M) at r.t. λ_{exc}= 350 nm.

5.2. Quantum Yield

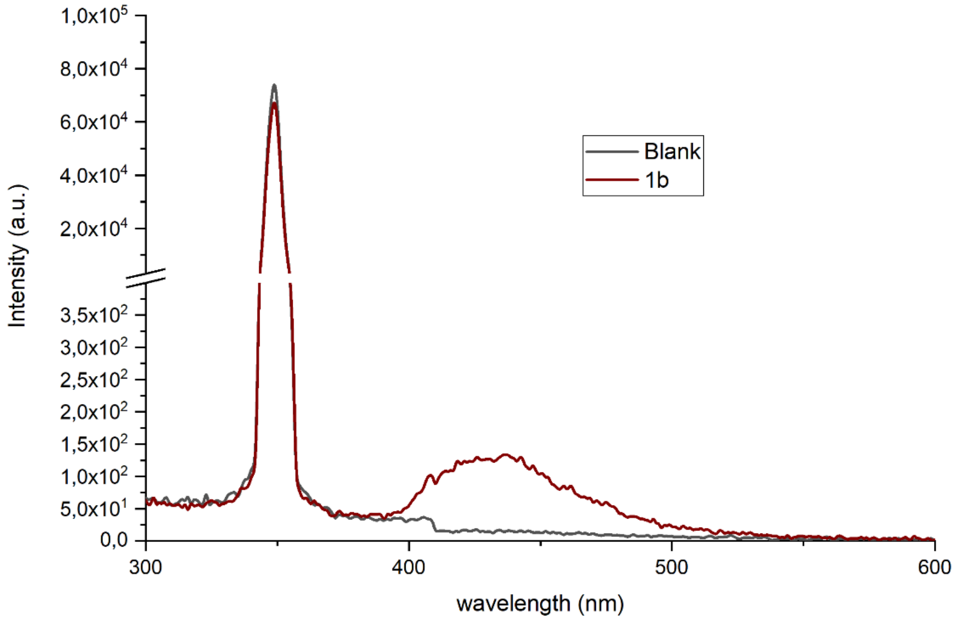

Figure S6 Emission spectra of blank and helicene **1b** measured with an integrating sphere under identical conditions, λ_{ex} = 350 nm. Background correction was applied to the blank spectrum prior to subtraction. Φ_f = 0.20

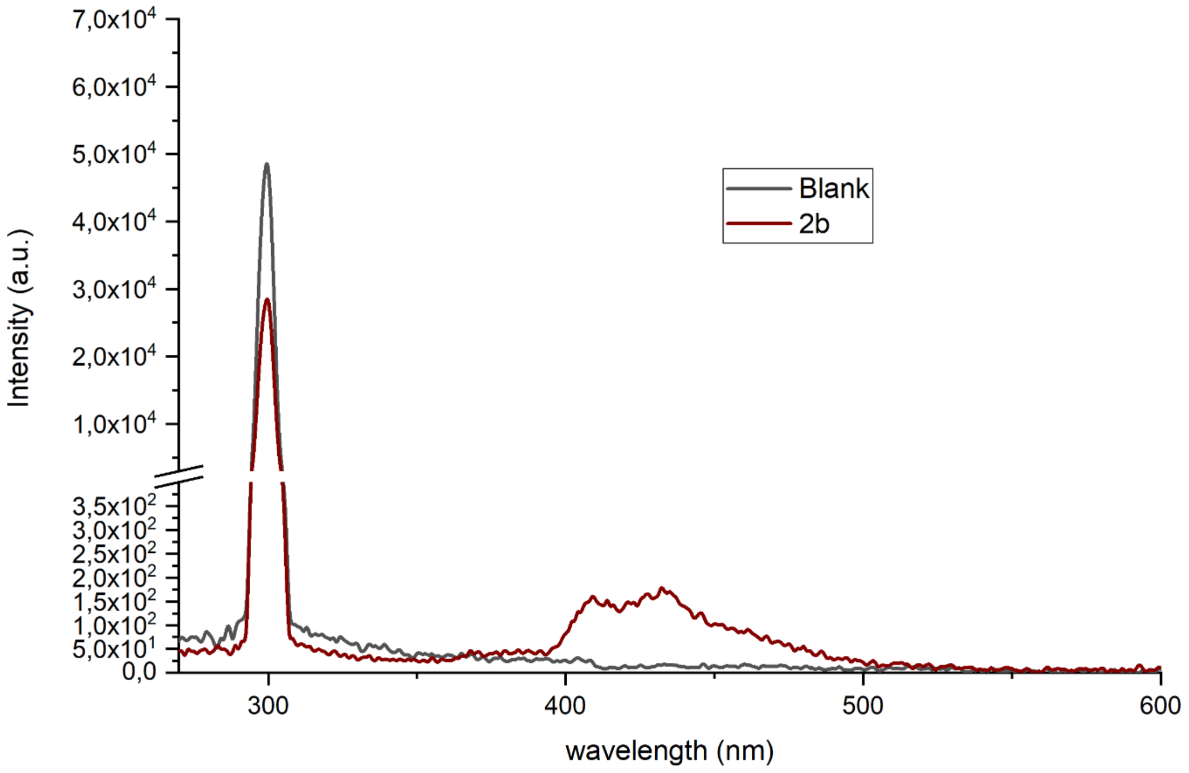

Figure S7 Emission spectra of blank and helicene **2b** measured with an integrating sphere under identical conditions, λ_{ex} = 300 nm. Background correction was applied to the blank spectrum prior to subtraction. Φ_f = 0.10

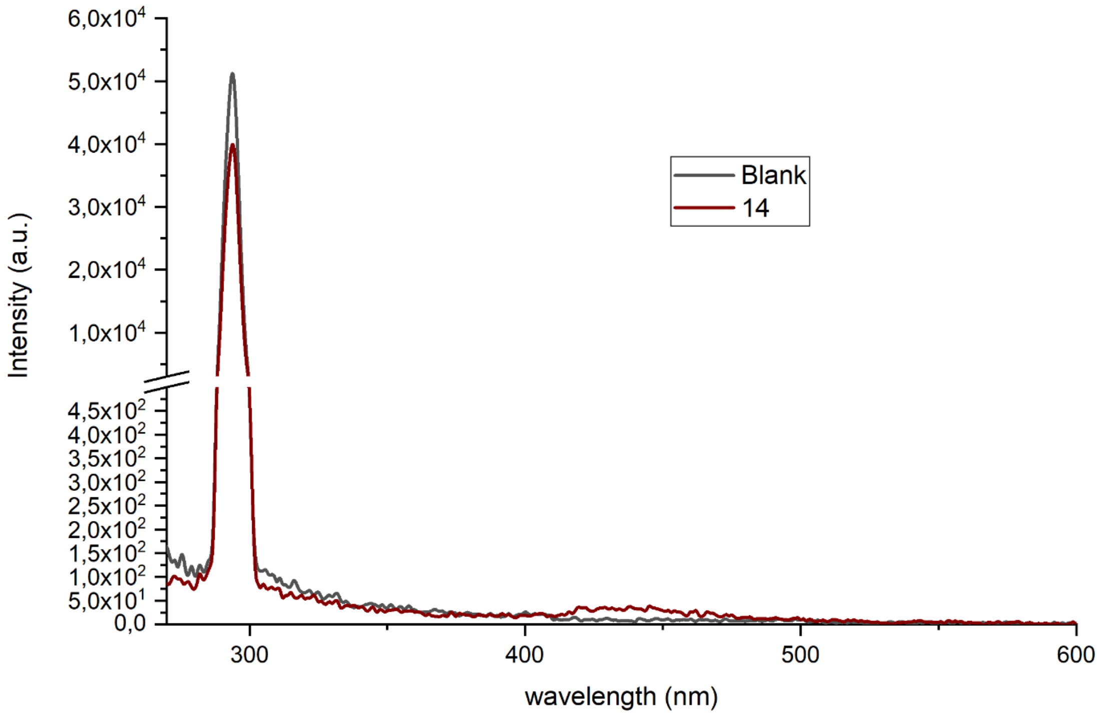

Figure S8 Emission spectra of blank and helicene **14** measured with an integrating sphere under identical conditions, λ_{ex} = 295 nm. Background correction was applied to the blank spectrum prior to subtraction. Φ_f = 0.02

5.3. Lifetime measurement

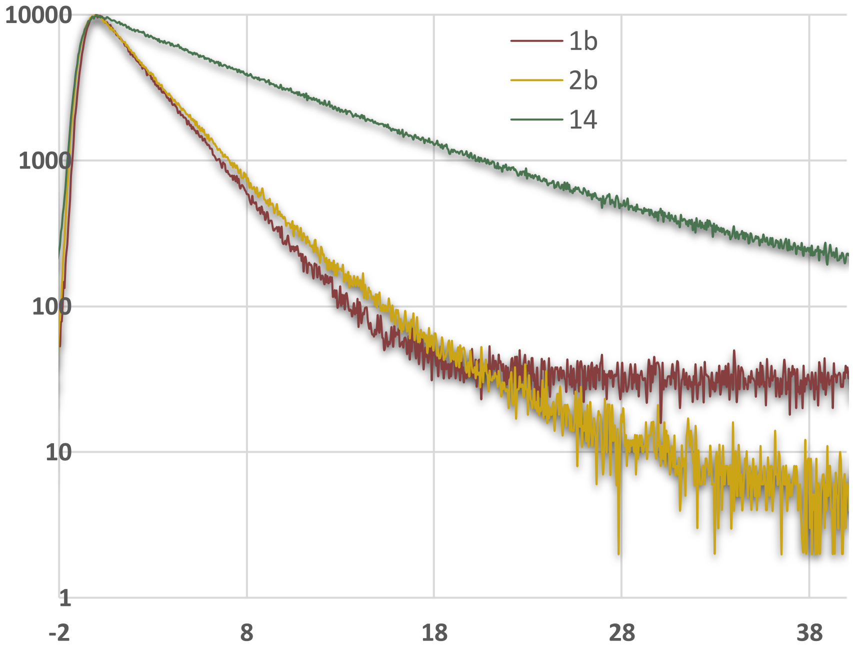

Figure S9 Fluorescence decay profiles

5.4. Circular Dichroism of Helicenes

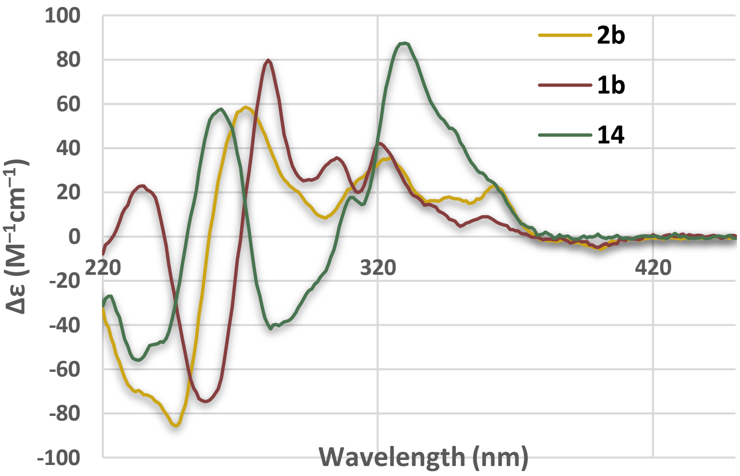

Figure S10 Circular Dichroism (80 µM in DCM) of selected helicenes **2b**, **1b** and **14**.

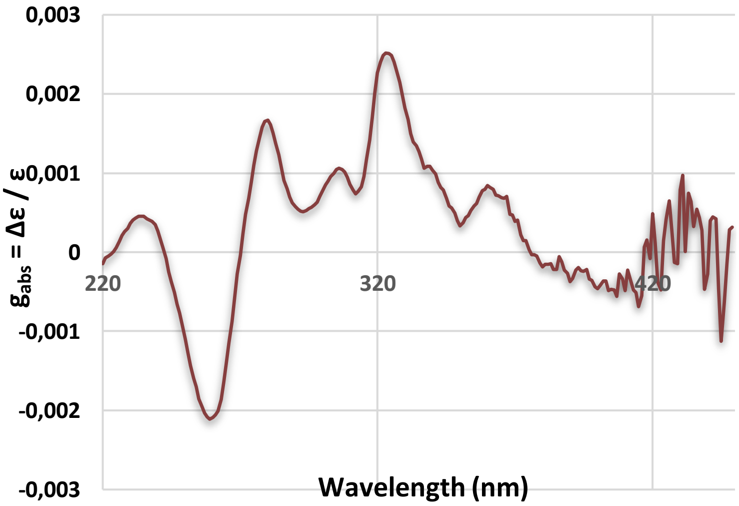

Figure S11 Plot for the absorption dissymmetry factor (g_{abs}) of **1b** in DCM solution

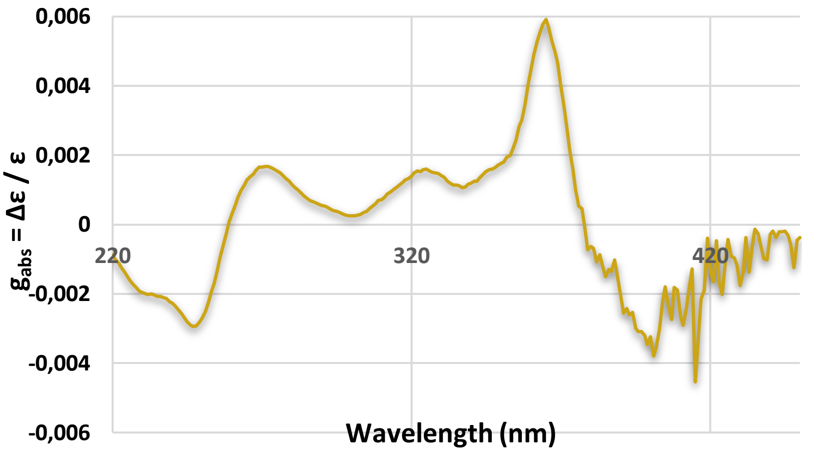

Figure S12 Plot for the absorption dissymmetry factor (g_{abs}) of **2b** in DCM solution

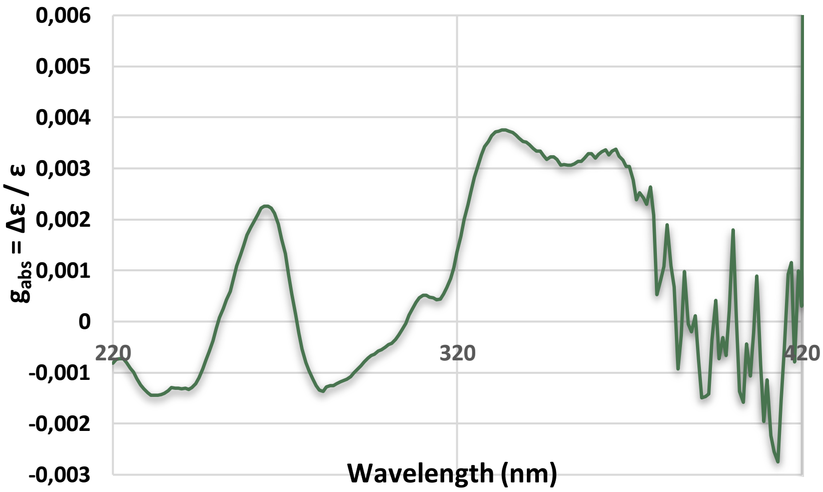

Figure S13 Plot for the absorption dissymmetry factor (g_{abs}) of **14** in DCM solution

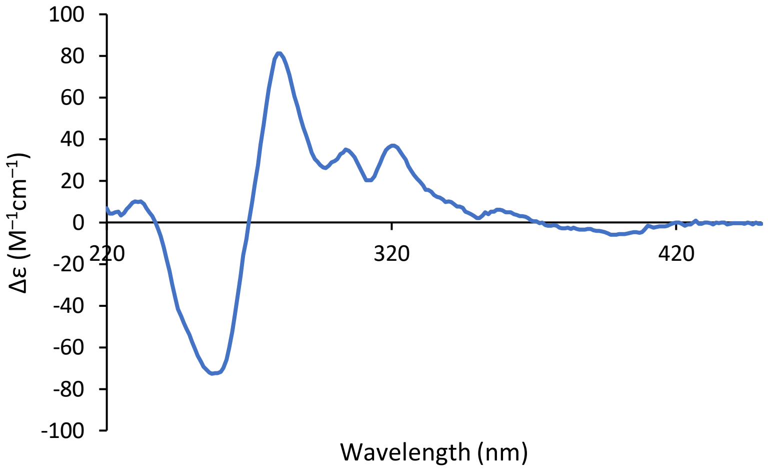

Figure S14 Circular Dichroism (80 μM in DCM) of Helicene **1a**.

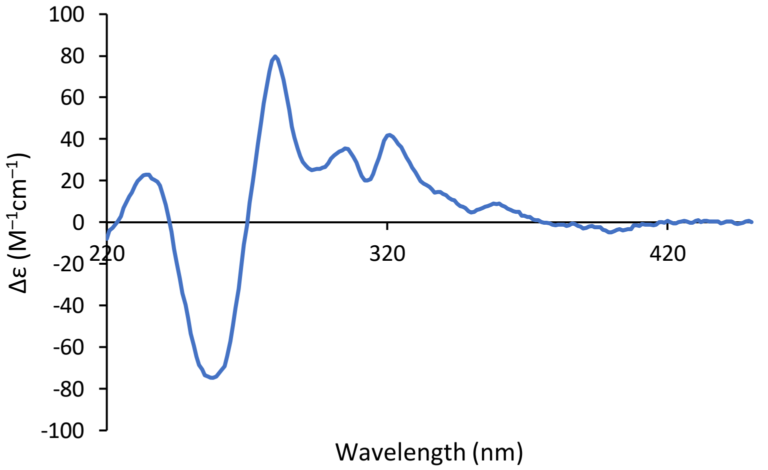

Figure S15 Circular Dichroism (80 μM in DCM) of Helicene **1b**.

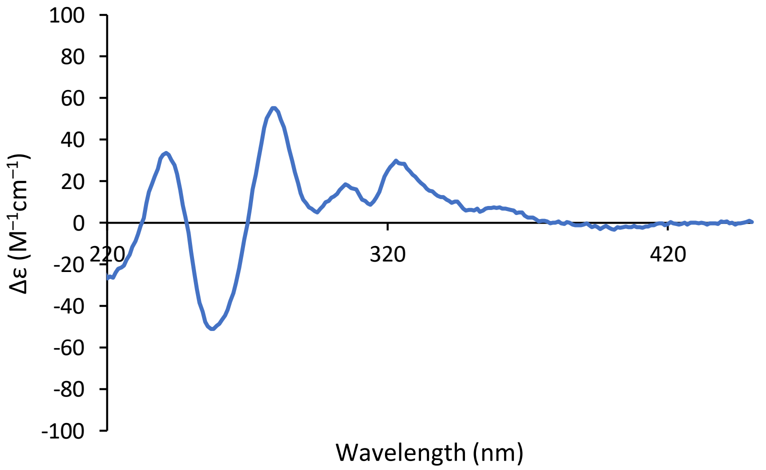

Figure S16 Circular Dichroism (80 μM in DCM) of Helicene **1c**.

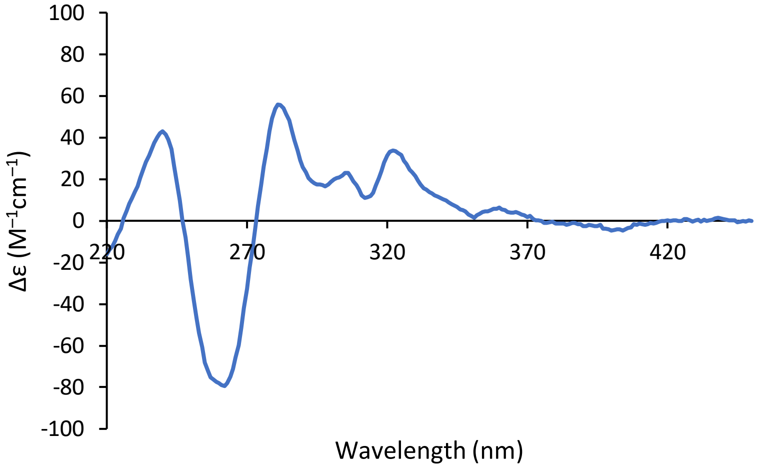

Figure S17 Circular Dichroism (80 μM in DCM) of Helicene **1d**.

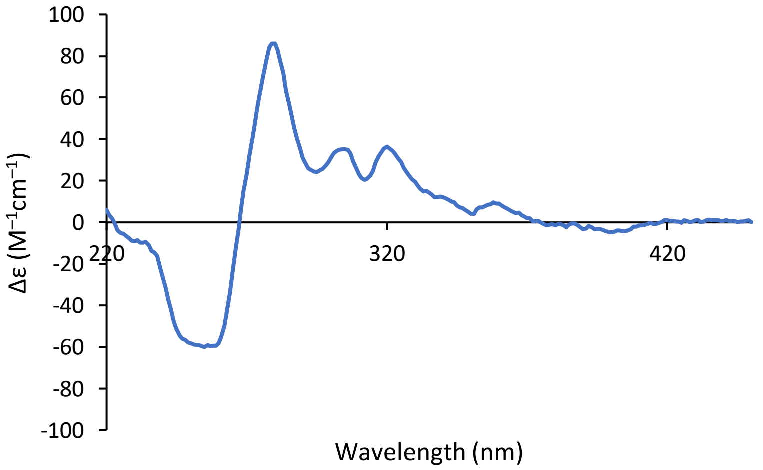

Figure S18 Circular Dichroism (80 μM in DCM) of Helicene **1e**.

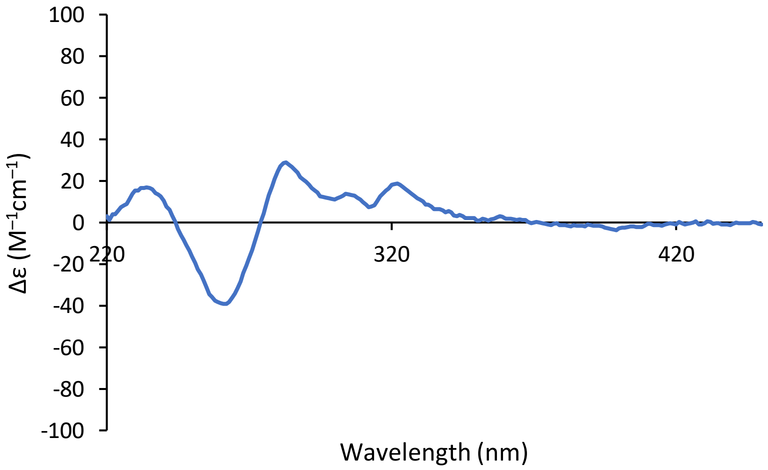

Figure S19 Circular Dichroism (80 μM in DCM) of Helicene **1f**.

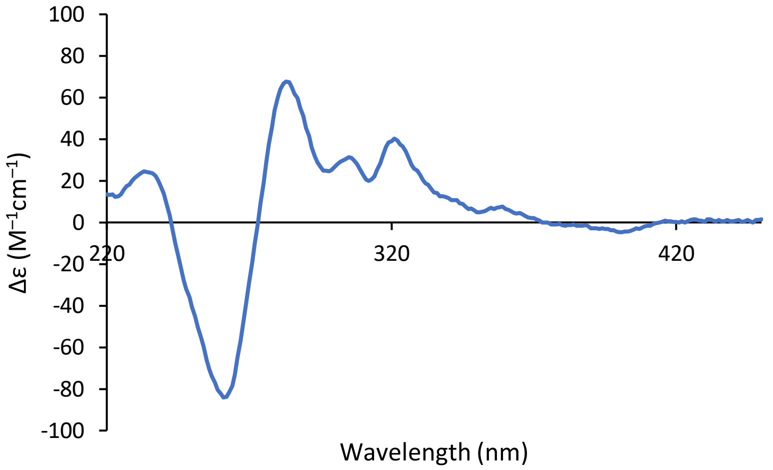

Figure S20 Circular Dichroism (80 µM in DCM) of Helicene **1g**.

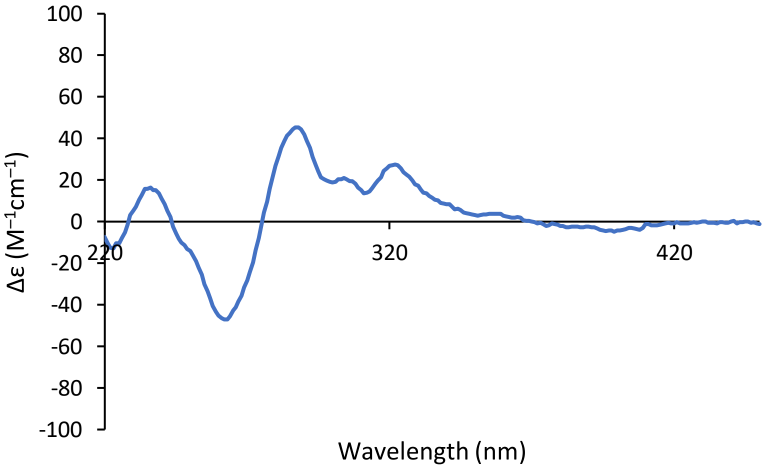

Figure S21 Circular Dichroism (80 µM in DCM) of Helicene **1h**.

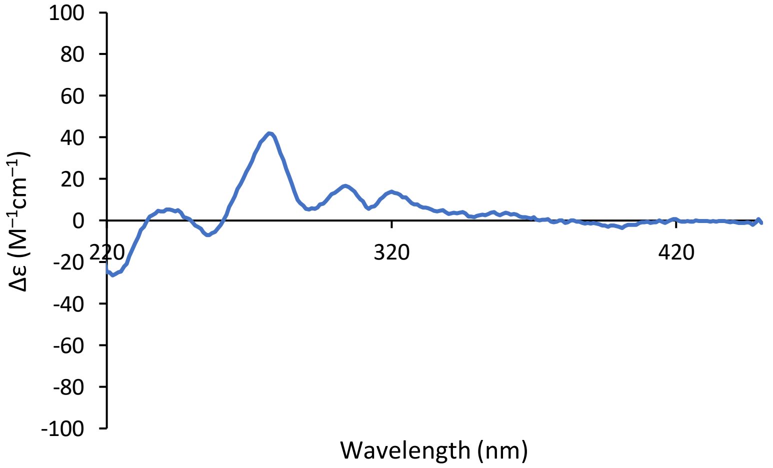

Figure S22 Circular Dichroism (80 µM in DCM) of Helicene **1i**.

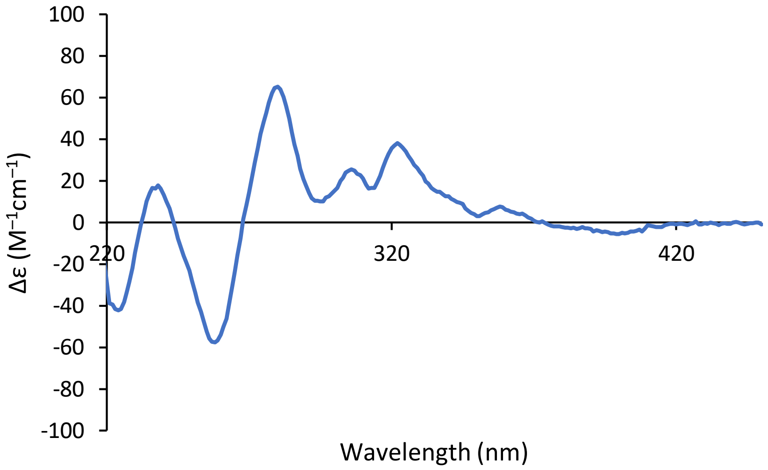

Figure S23 Circular Dichroism (80 µM in DCM) of Helicene **1j**.

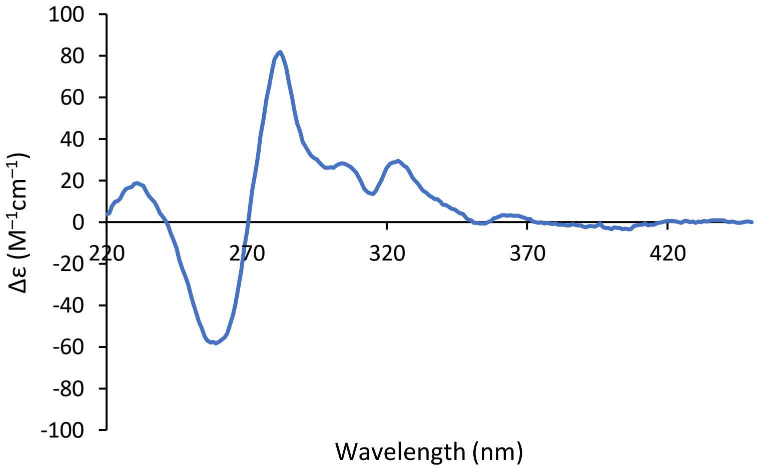

Figure S24 Circular Dichroism (80 µM in DCM) of Helicene **1b(BPh)**.

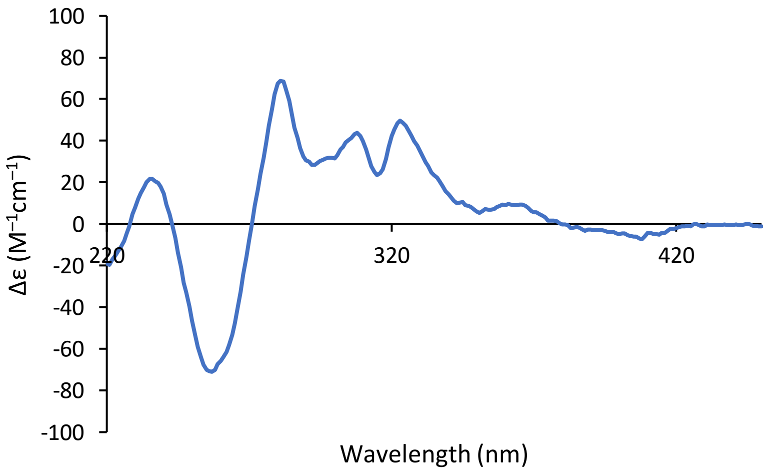

Figure S25 Circular Dichroism (80 µM in DCM) of Helicene **1b(NMe)**.

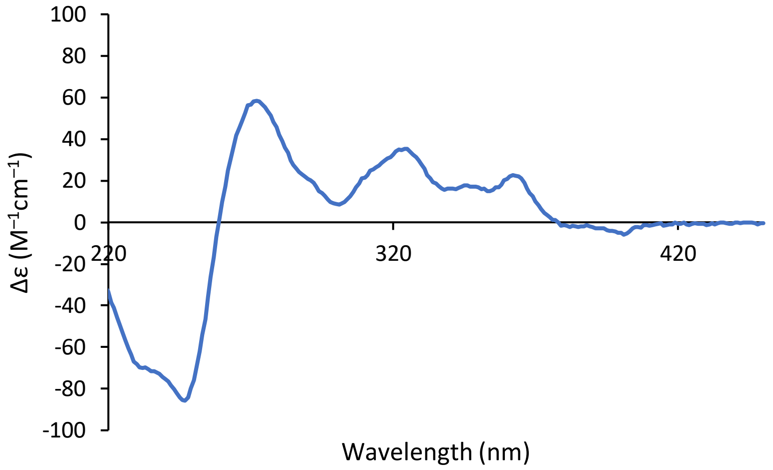

Figure S26 Circular Dichroism (80 μM in DCM) of Helicene **2b**.

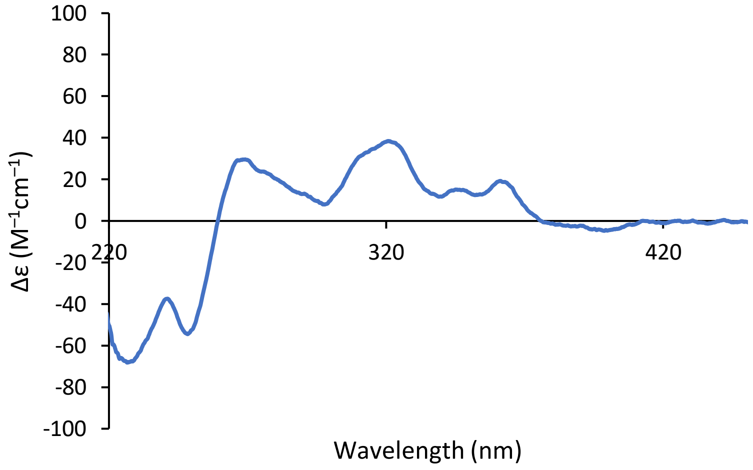

Figure S27 Circular Dichroism (80 μM in DCM) of Helicene **2c**.

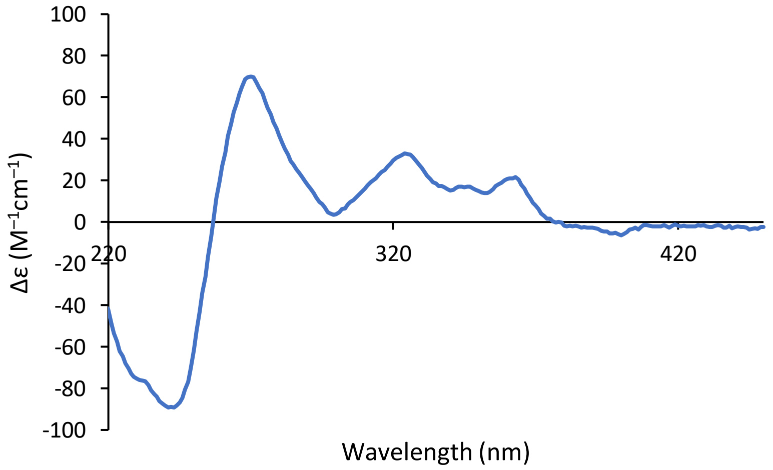

Figure S28 Circular Dichroism (80 μM in DCM) of Helicene **2e**.

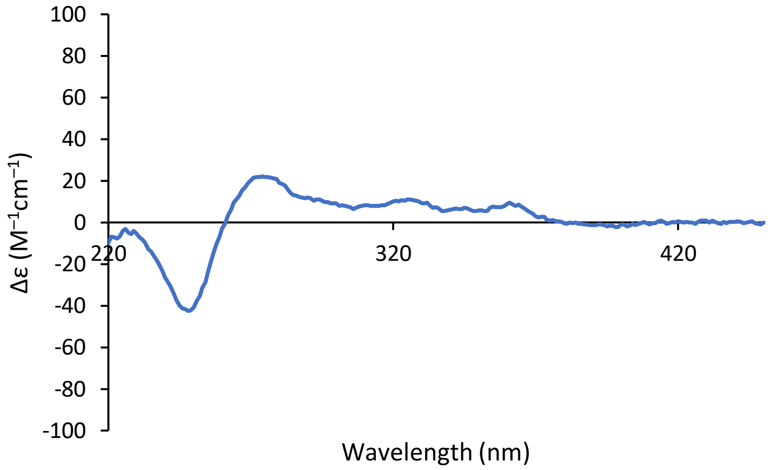

Figure S29 Circular Dichroism (80 µM in DCM) of Helicene **2h**.

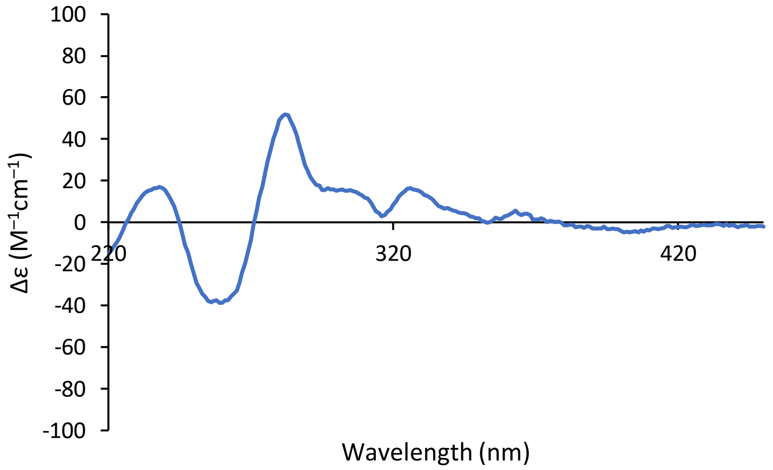

Figure S30 Circular Dichroism (80 µM in DCM) of Helicene **12b**.

5.5. Circular Polarized Luminescence of Helicenes

For helicenes **1i** and **1b(NMe)**, g_{lum} values were extracted from the CPL spectra at the emission maximum. For helicenes **1b** and **2b** g_{lum} values were obtained from CPL measurements at three wavelengths around the emission maximum to minimize acquisition time due to sample decomposition; full CPL spectra were measured separately.

For helicene **1b** g_{lum} was determined from CPL measurements at 430–432 nm (1 s per point, 3 points), with 500 scans averaged.

For helicene **2b** g_{lum} was determined from CPL measurements at 430–433 nm (1 s per point, 3 points), with 50 scans averaged.

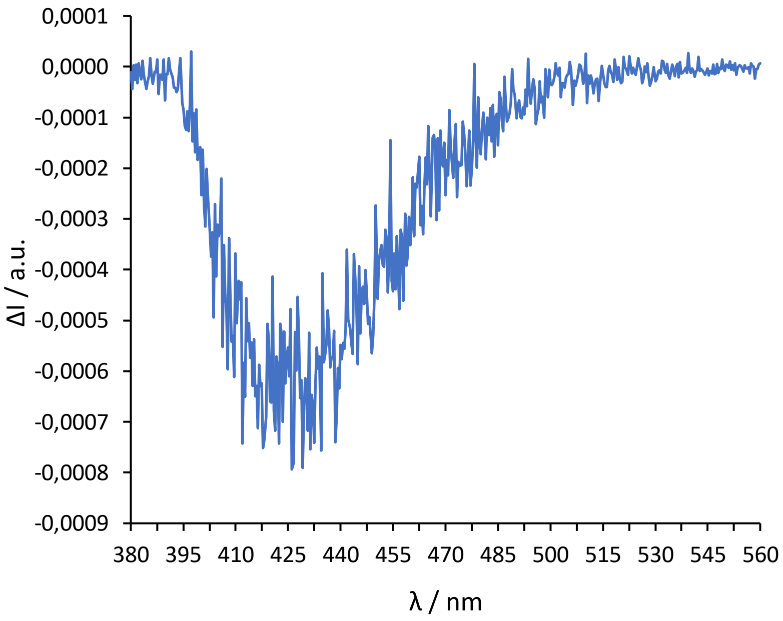

Figure S31 CPL spectrum of helicene **1i** (1.0×10^{-5} M in CH_2Cl_2, λ_{exc}=310 nm); the spectrum (380–560 nm, 3 s per point, 481 points) was averaged from 31 scans; g_{lum}= -8.4×10^{-4} (438 nm).

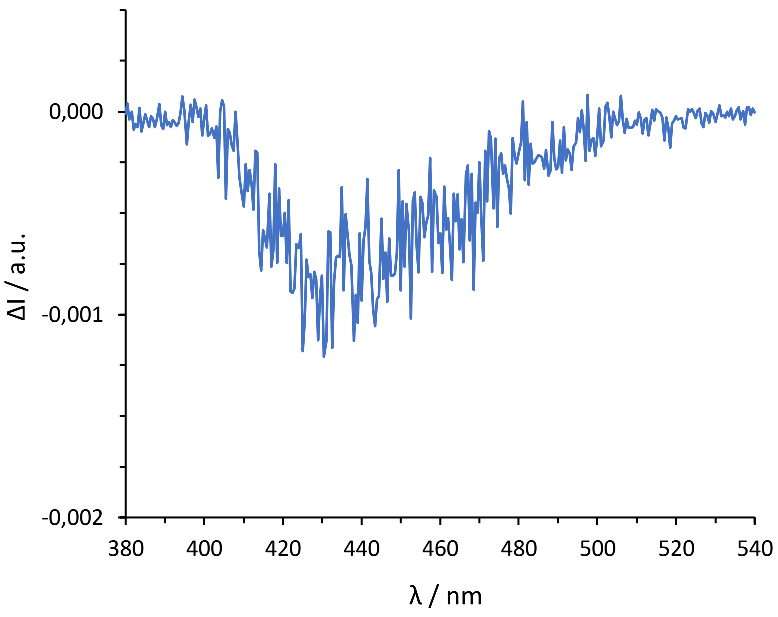

Figure S32 CPL spectrum of helicene **1b(NMe)** (1.0 × 10^{-5} M in CH_2Cl_2, λ_{exc}=310 nm); the spectrum (380–540 nm, 3 s per point, 321 points) was averaged from 82 scans; g_{lum}= -1.1 × 10^{-3} (438 nm).

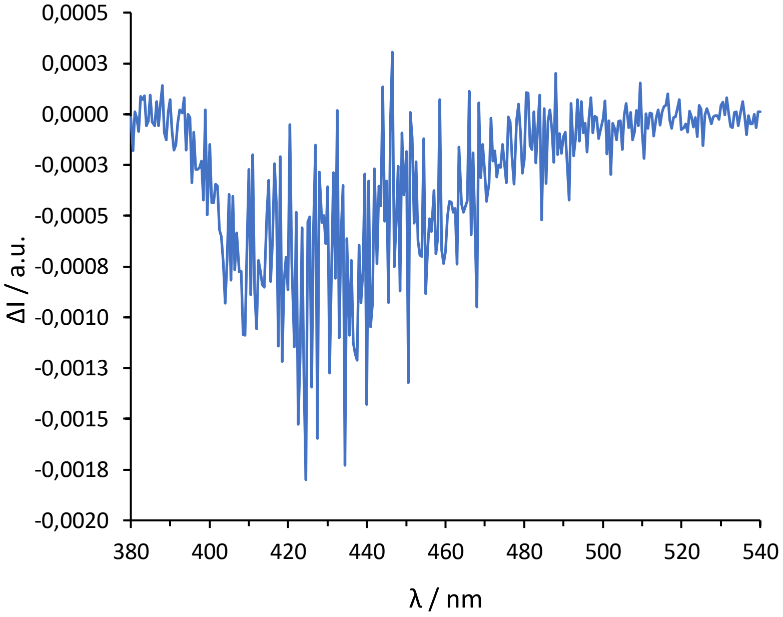

Figure S33 CPL spectrum of helicene **1b** (1.0 × 10^{-5} M in CH_2Cl_2, λ_{exc}=310 nm); the spectrum (380–540 nm, 2 s per point, 321 points) was averaged from 30 scans, sample refreshed every 3 scans; g_{lum}= -1.0 × 10^{-3} (432 nm). For g_{lum} determination: 430–432 nm, 1 s per point, 3 points, averaged from 500 scans.

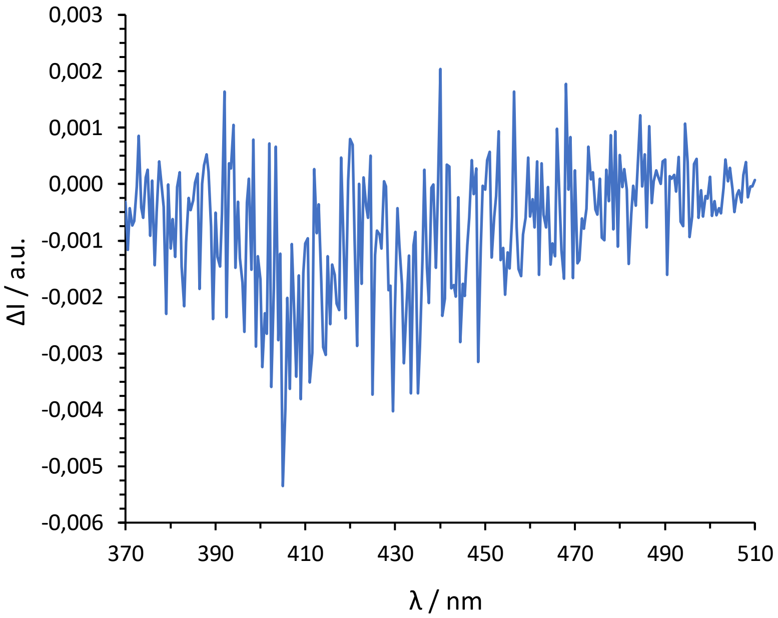

Figure S34 CPL spectrum of helicene **2b** (1.0×10^{-5} M in CH_2Cl_2, λ_{exc}=270 nm); the spectrum (370–510 nm, 1 s per point, 281 points) was averaged from 29 scans; g_{lum}= -1.7×10^{-3} (430 nm).

6. Racemization Experiments

An enantio-enriched helicene (1 mg) was dissolved in 1,2,4-trichlorobenzene (1.5 mL), and the solution was heated at the indicated temperature. The time-course of enantiomeric ratio was monitored by HPLC. Helicenes **1b** and **2b** were used as model compounds for the experiments.

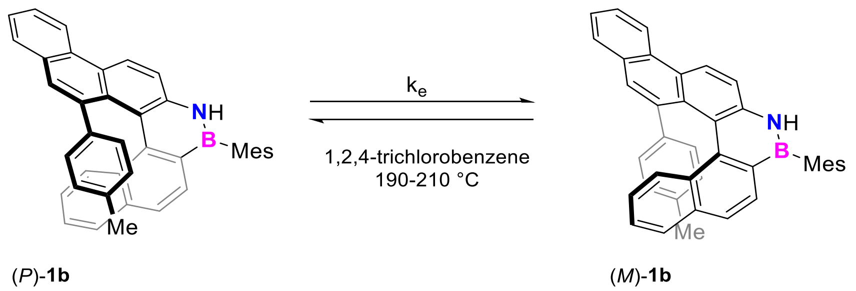

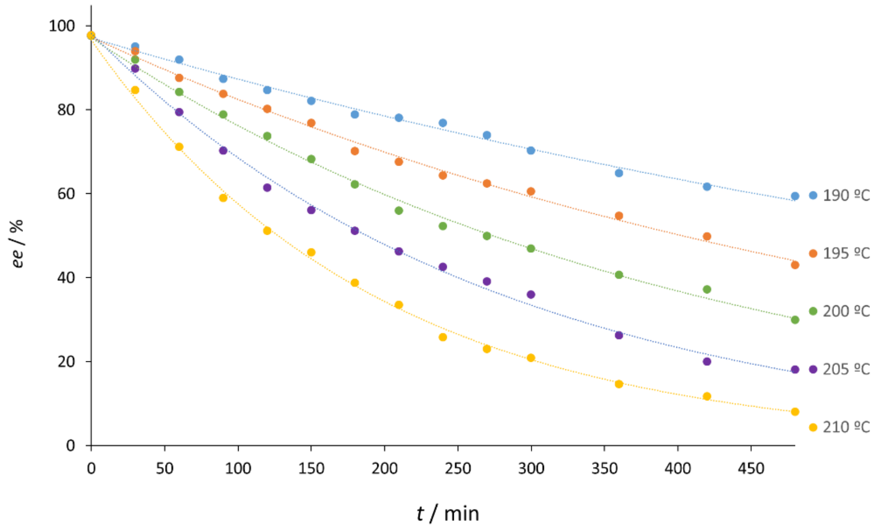

Figure S35 Time course of the ee of **1b** over time at various temperatures.

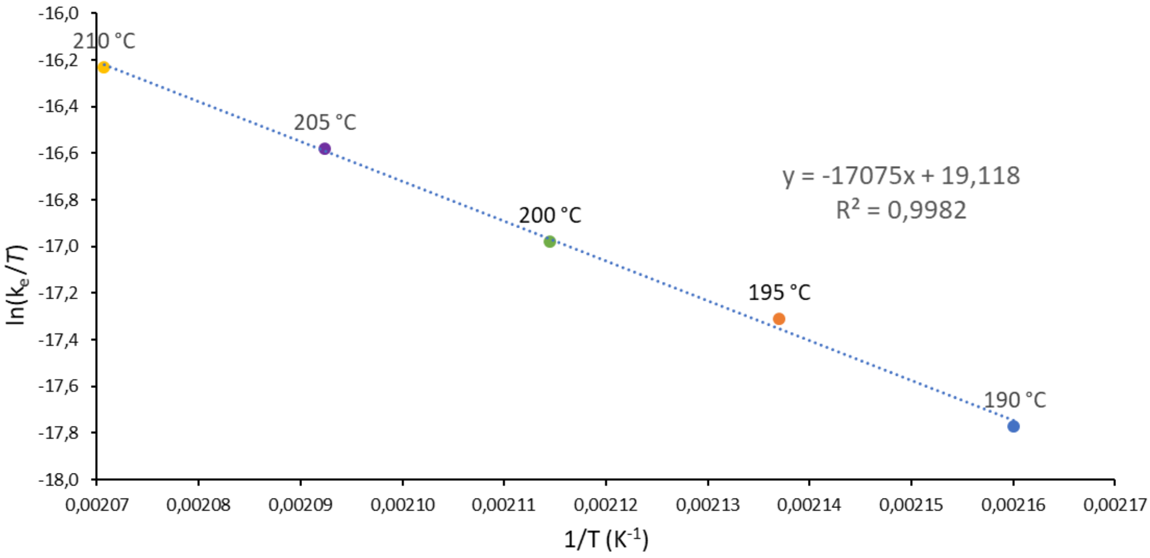

Figure S36 Eyring plot for **1b.**

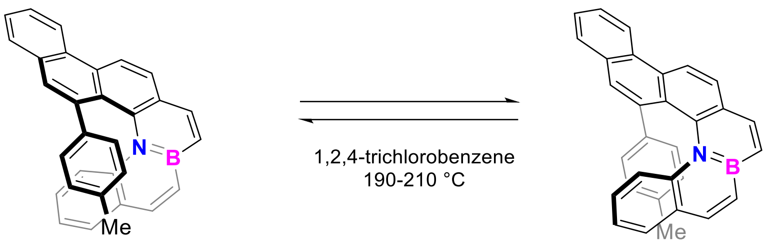

(*P*)-**2b** (*M*)-**2b**

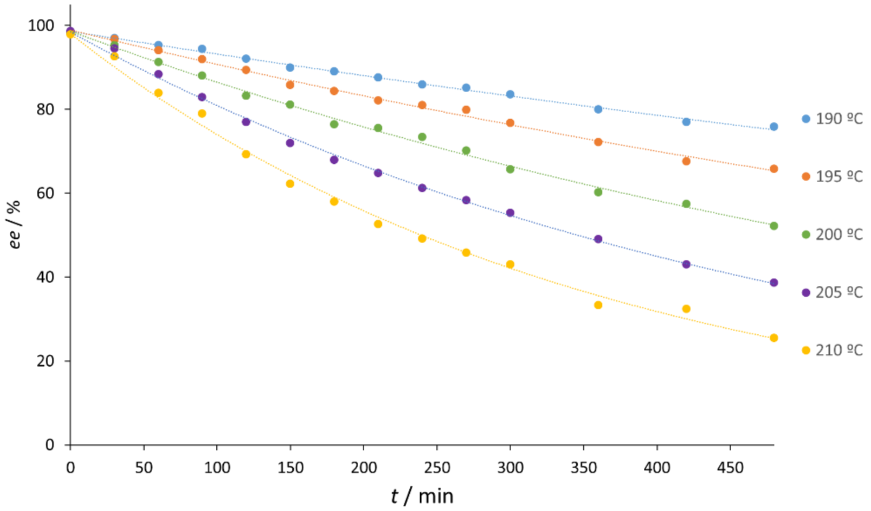

Figure S37 Time course of the ee of **2b** over time at various temperatures.

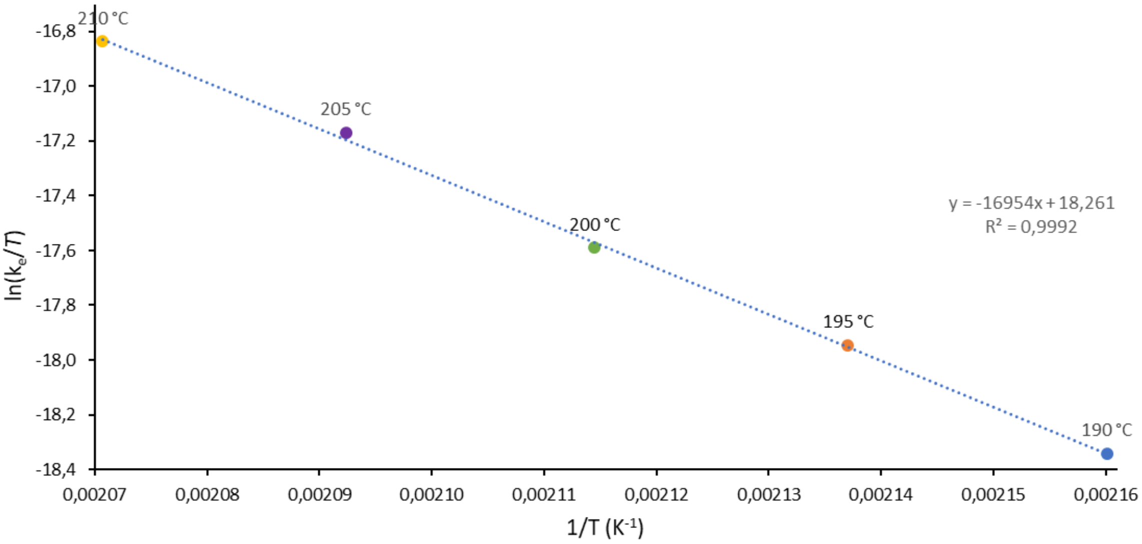

Figure S38 Eyring plot for **2b.**

7. Crystallographic Supplement

7.1. **1b**◦MeCN

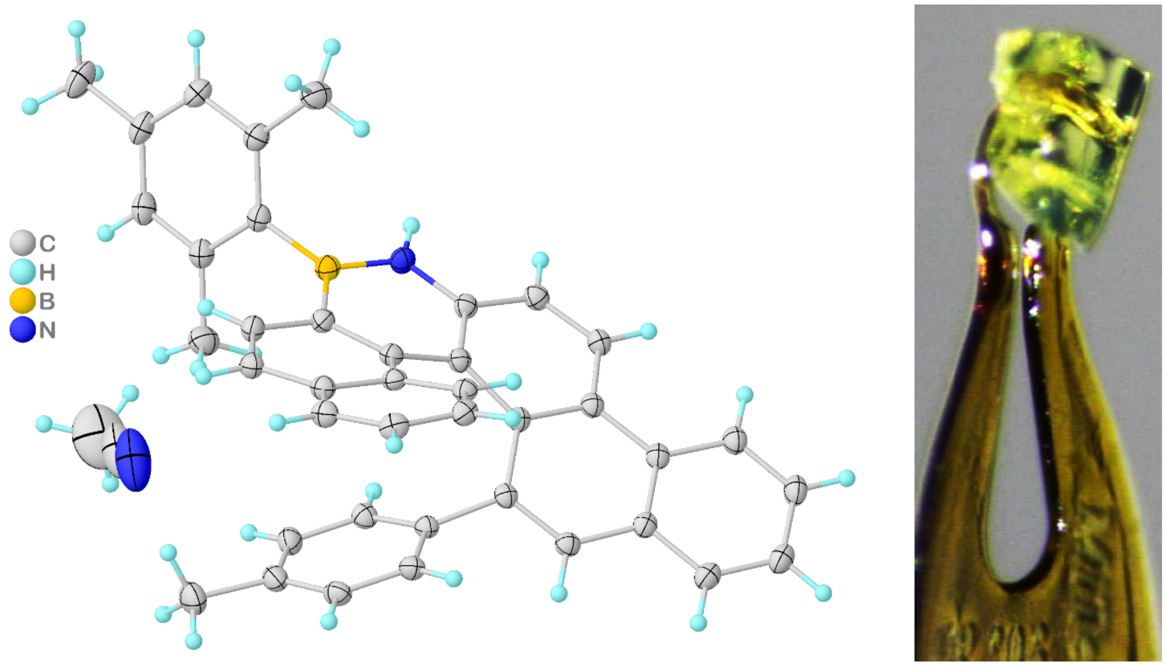

Figure S39: Full asymmetric unit of **1b**◦MeCN. Anisotropic displacement ellipsoids drawn at 50% probability level. Single crystals were obtained from a solution in acetonitrile.

CCDC number	2506342
Empirical formula	$C_{42}H_{35}BN_2$
Formula weight	578.53
Temperature [K]	100.00
Crystal system	Orthorhombic
Space group (number)	$P2_12_12_1$ (19)
a [Å]	8.3302(6)
b [Å]	12.2120(9)
c [Å]	31.850(2)
α [°]	90
β [°]	90
γ [°]	90
Volume [Å^3]	3240.1(4)
Z	4
ρ_{calc} [gcm^{-3}]	1.186
μ [mm^{-1}]	0.517
F(000)	1224
Crystal size [mm^3]	0.353×0.291×0.247
Crystal colour	Colourless
Crystal shape	Block
Radiation	CuK_α (λ=1.54178 Å)
2θ range [°]	7.75 to 157.70 (0.79 Å)
Index ranges	$-10 \leq h \leq 9$<br>$-15 \leq k \leq 15$<br>$-40 \leq l \leq 40$

Reflections collected	97003
Independent reflections	6984<br>R_{int} = 0.0458<br>R_{sigma} = 0.0177
Completeness to θ = 67.679°	100.0 %
Data / Restraints / Parameters	6984/1/416
Absorption correction T_{min}/T_{max} (method)	0.7575/0.9830 (numerical)
Goodness-of-fit on F^2	1.033
Final R indexes [$I \geq 2\sigma(I)$]	R_1 = 0.0312<br>wR_2 = 0.0831
Final R indexes [all data]	R_1 = 0.0315<br>wR_2 = 0.0833
Largest peak/hole [eÅ^{-3}]	0.21/−0.17
Flack X parameter	−0.07(9)
Extinction coefficient	0.0038(3)

7.2.**1d**◦CH_2Cl_2

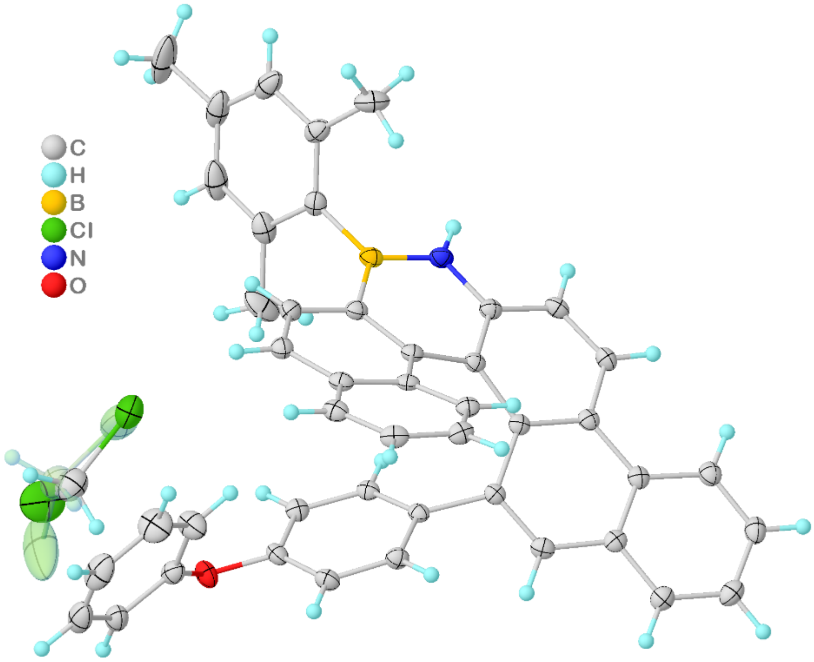

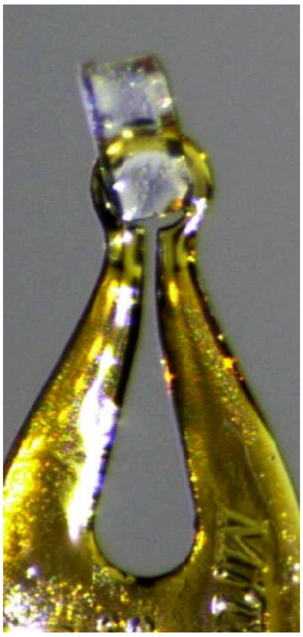

Figure S40: Full asymmetric unit of **1d**◦CH_2Cl_2. Anisotropic displacement ellipsoids drawn at 50% probability level; disordered molecule parts in PART 2 drawn in green hue. The co-crystallized dichloromethane exhibits some positional disorder. Single crystals were obtained from a solution in dichloromethane.

CCDC number	2506343
Empirical formula	$C_{46}H_{36}BCl_2NO$
Formula weight	700.47
Temperature [K]	100.00
Crystal system	Monoclinic
Space group (number)	$P2_1$ (4)
a [Å]	8.4475(2)
b [Å]	12.6614(5)
c [Å]	16.7944(5)
α [°]	90
β [°]	91.358(2)
γ [°]	90
Volume [Å^3]	1795.78(10)
Z	2
ρ_{calc} [gcm^{-3}]	1.295
μ [mm^{-1}]	1.911
F(000)	732
Crystal size [mm^3]	0.365×0.142×0.06
Crystal colour	Colourless
Crystal shape	Plate
Radiation	CuK_α (λ=1.54178 Å)
2θ range [°]	5.26 to 159.62 (0.78 Å)

Index ranges	$-10 \leq h \leq 10$<br>$-16 \leq k \leq 15$<br>$-21 \leq l \leq 21$
Reflections collected	70578
Independent reflections	7623<br>R_{int} = 0.0476<br>R_{sigma} = 0.0200
Completeness to θ = 67.679°	100.0 %
Data / Restraints / Parameters	7623/11/496
Absorption correction T_{min}/T_{max} (method)	0.6485/0.9468 (numerical)
Goodness-of-fit on F^2	1.030
Final *R* indexes [$I \geq 2\sigma(I)$]	R_1 = 0.0337<br>wR_2 = 0.0875
Final *R* indexes [all data]	R_1 = 0.0343<br>wR_2 = 0.0882
Largest peak/hole [eÅ^{-3}]	0.58/−0.46
Flack X parameter	−0.004(5)

Extinction coefficient	0.0026(3)

7.3. **1e**∘MeCN

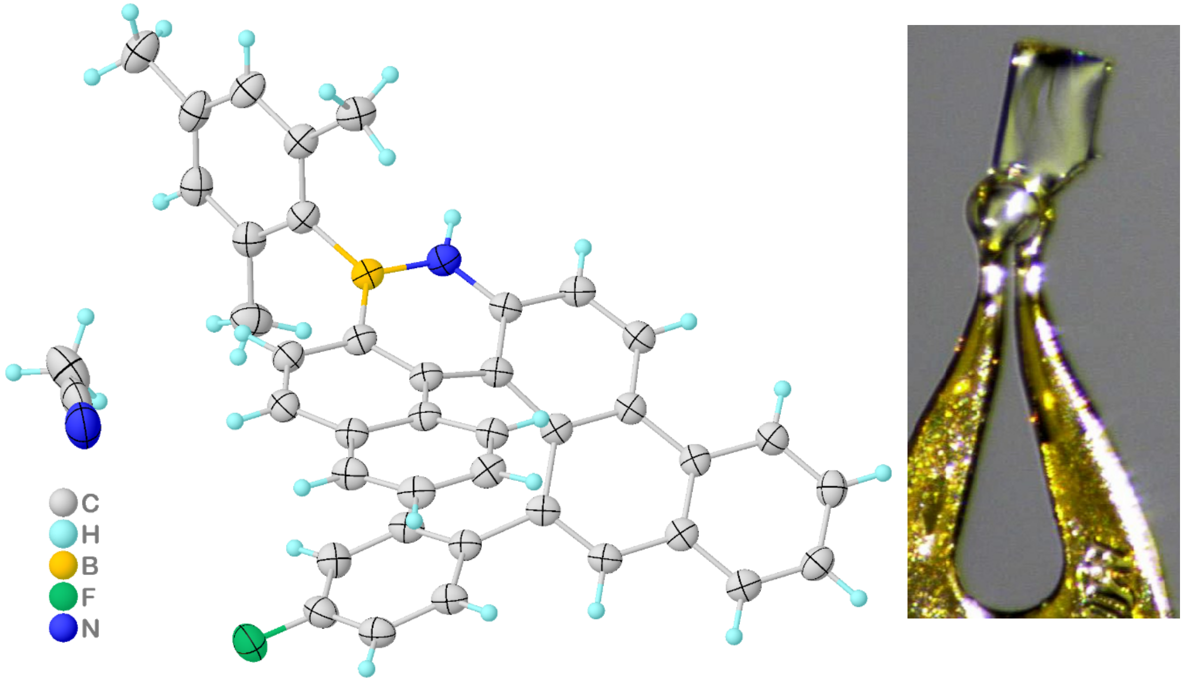

Figure S41: Full asymmetric unit of **1e**∘MeCN. Anisotropic displacement ellipsoids drawn at 50% probability level. Single crystals were obtained from a solution in acetonitrile.

CCDC number	2506344
Empirical formula	$C_{80}H_{61}B_2F_2N_3$
Formula weight	1123.93
Temperature [K]	100.00
Crystal system	Orthorhombic
Space group (number)	$P2_12_12_1$ (19)
a [Å]	8.2943(11)
b [Å]	12.2566(7)
c [Å]	31.139(2)
α [°]	90
β [°]	90
γ [°]	90
Volume [Å^3]	3165.6(5)
Z	2
ρ_{calc} [gcm^{-3}]	1.179
μ [mm^{-1}]	0.558
F(000)	1180
Crystal size [mm^3]	0.5×0.144×0.094
Crystal colour	Colourless
Crystal shape	Plate
Radiation	CuK_α (λ=1.54178 Å)
2θ range [°]	7.75 to 158.86 (0.78 Å)
Index ranges	−8 ≤ h ≤ 10<br>−15 ≤ k ≤ 15<br>−39 ≤ l ≤ 38

Reflections collected	67824
Independent reflections	6826<br>R_{int} = 0.0745<br>R_{sigma} = 0.0373
Completeness to θ = 67.679°	99.7 %
Data / Restraints / Parameters	6826/0/410
Absorption correction T_{min}/T_{max} (method)	0.7580/0.9949 (numerical)
Goodness-of-fit on F^2	1.117
Final R indexes [I≥2σ(I)]	R_1 = 0.0688<br>wR_2 = 0.2099
Final R indexes [all data]	R_1 = 0.0716<br>wR_2 = 0.2123
Largest peak/hole [eÅ^{-3}]	0.34/−0.28
Flack X parameter	0.02(10)

7.4.**1f**

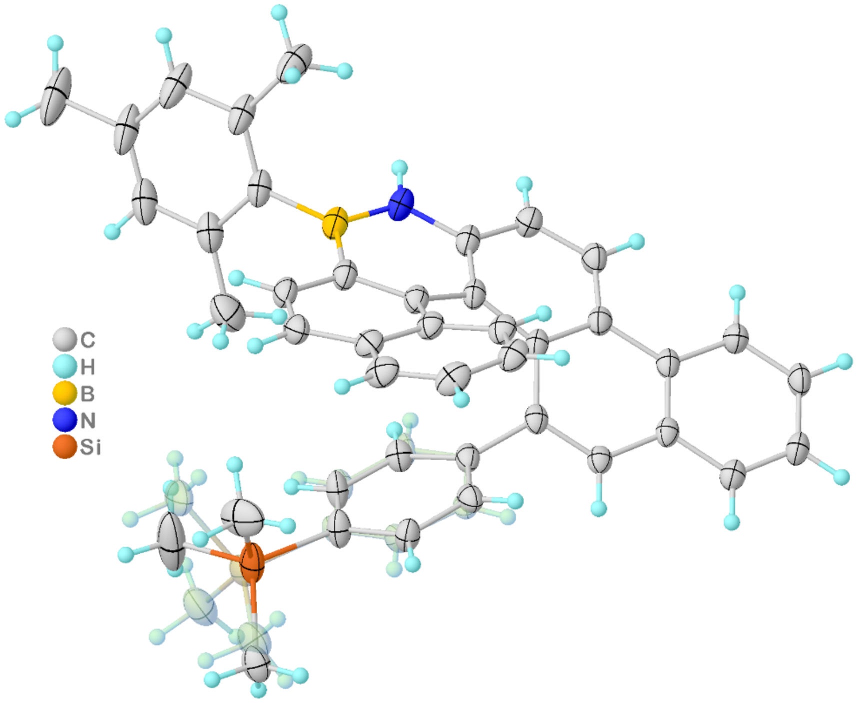

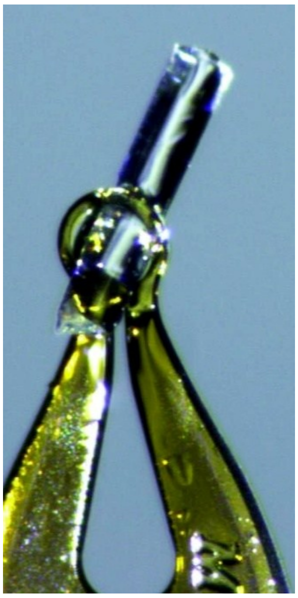

Figure S42: Full asymmetric unit of **1f**. Anisotropic displacement ellipsoids drawn at 50% probability level; disordered molecule parts in PART 2 drawn in green hue. Single crystals were obtained from a solution in acetonitrile.

CCDC number	2506345
Empirical formula	$C_{42}H_{38}BNSi$
Formula weight	595.63
Temperature [K]	100.00
Crystal system	Orthorhombic
Space group (number)	$P2_12_12_1$ (19)
a [Å]	8.2406(9)
b [Å]	13.1465(19)
c [Å]	31.983(4)
α [°]	90
β [°]	90
γ [°]	90
Volume [Å^3]	3464.9(8)
Z	4
ρ_{calc} [gcm^{-3}]	1.142
μ [mm^{-1}]	0.097
F(000)	1264
Crystal size [mm^3]	0.895×0.101×0.075
Crystal colour	Colourless
Crystal shape	Needle
Radiation	MoK_α (λ=0.71073 Å)
2θ range [°]	4.01 to 54.22 (0.78 Å)

Index ranges	$-10 \le h \le 10$<br>$-16 \le k \le 16$<br>$-41 \le l \le 41$
Reflections collected	109371
Independent reflections	7652<br>R_{int} = 0.0413<br>R_{sigma} = 0.0155
Completeness to θ = 25.242°	99.9 %
Data / Restraints / Parameters	7652/41/498
Absorption correction T_{min}/T_{max} (method)	0.9428/1.0000 (numerical)
Goodness-of-fit on F^2	1.057
Final *R* indexes [$I \geq 2\sigma(I)$]	R_1 = 0.0360<br>wR_2 = 0.0951
Final *R* indexes [all data]	R_1 = 0.0402<br>wR_2 = 0.0995
Largest peak/hole [eÅ^{-3}]	0.27/−0.16
Flack X parameter	0.04(4)
Extinction coefficient	0.0046(9)

7.5. **1g**∘MeCN

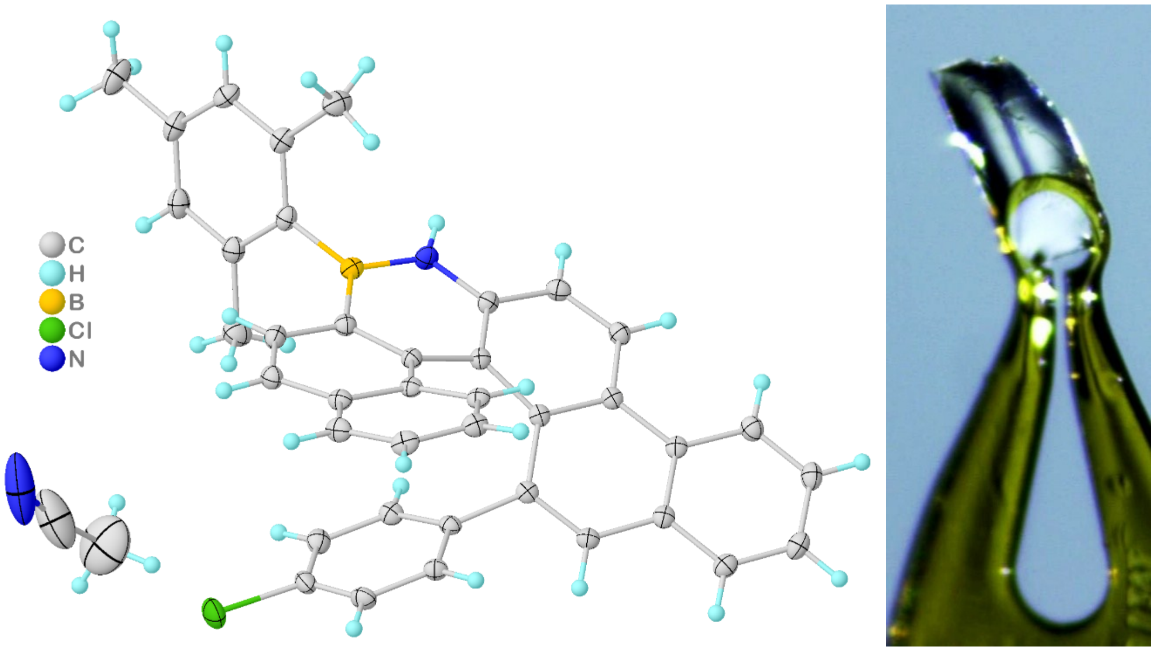

Figure S43: Full asymmetric unit of **1g**∘MeCN. Anisotropic displacement ellipsoids drawn at 50% probability level. Single crystals were obtained from a solution in acetonitrile.

CCDC number	2506346
Empirical formula	$C_{41}H_{32}BClN_2$
Formula weight	598.94
Temperature [K]	100.00
Crystal system	Orthorhombic
Space group (number)	$P2_12_12_1$ (19)
a [Å]	8.3058(10)
b [Å]	12.2298(10)
c [Å]	31.682(3)
α [°]	90
β [°]	90
γ [°]	90
Volume [Å^3]	3218.2(6)
Z	4
ρ_{calc} [gcm^{-3}]	1.236
μ [mm^{-1}]	0.151
F(000)	1256
Crystal size [mm^3]	0.449×0.302×0.02
Crystal colour	Colourless
Crystal shape	Plate
Radiation	MoK_α (λ=0.71073 Å)
2θ range [°]	4.21 to 57.43 (0.74 Å)
Index ranges	$-11 \le h \le 11$<br>$-16 \le k \le 13$<br>$-42 \le l \le 42$
Reflections collected	86758

Independent reflections	8317<br>R_{int} = 0.0493<br>R_{sigma} = 0.0248
Completeness to θ = 25.242°	99.9 %
Data / Restraints / Parameters	8317/1/413
Absorption correction T_{min}/T_{max} (method)	0.8850/1.0000 (numerical)
Goodness-of-fit on F^2	1.020
Final R indexes [$I \geq 2\sigma(I)$]	R_1 = 0.0384<br>wR_2 = 0.0901
Final R indexes [all data]	R_1 = 0.0440<br>wR_2 = 0.0938
Largest peak/hole [eÅ^{-3}]	0.54/−0.27
Flack X parameter	0.000(17)

7.6. **1h**

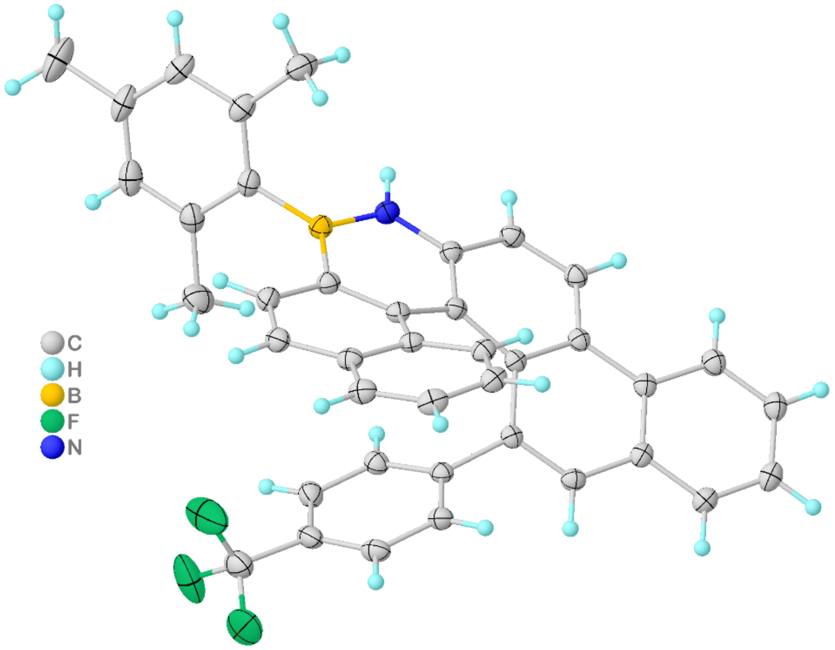

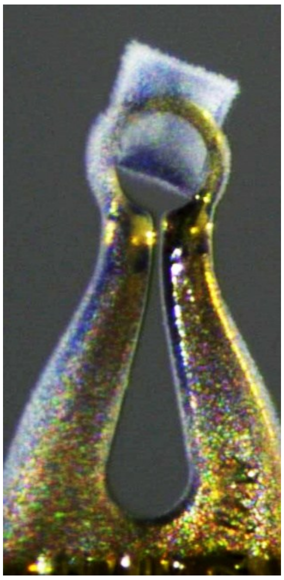

Figure S44: Full asymmetric unit of **1h**. Anisotropic displacement ellipsoids drawn at 50% probability level. There appears to be a solvent accesible void triggering an A level alert, but no relevant residual density is there to be found. Solvent mask was used, but it finds only a cavity content of 1 electron in 130 Å^3. Single crystals were obtained from a solution in dichloromethane.

CCDC number	2506347
Empirical formula	$C_{40}H_{29}BF_3N$
Formula weight	591.45
Temperature [K]	100.00
Crystal system	Orthorhombic
Space group (number)	$P2_12_12_1$ (19)
a [Å]	8.2373(5)
b [Å]	12.6200(8)
c [Å]	31.678(2)
α [°]	90
β [°]	90
γ [°]	90
Volume [Å^3]	3293.1(4)
Z	4
ρ_{calc} [gcm^{-3}]	1.193
μ [mm^{-1}]	0.647
F(000)	1232
Crystal size [mm^3]	0.04×0.245×0.256
Crystal colour	Colourless
Crystal shape	Plate
Radiation	CuK_α (λ=1.54178 Å)
2θ range [°]	7.54 to 158.15 (0.79 Å)

Index ranges	$-10 \le h \le 9$<br>$-16 \le k \le 16$<br>$-40 \le l \le 39$
Reflections collected	96476
Independent reflections	7084<br>R_{int} = 0.0615<br>R_{sigma} = 0.0229
Completeness to θ = 67.679°	99.9 %
Data / Restraints / Parameters	7084 / 0 / 410
Absorption correction T_{min}/T_{max} (method)	0.7737 / 1.0000 (numerical)
Goodness-of-fit on F^2	1.033
Final R indexes [$I \geq 2\sigma(I)$]	R_1 = 0.0314<br>wR_2 = 0.0828
Final R indexes [all data]	R_1 = 0.0330<br>wR_2 = 0.0843
Largest peak/hole [eÅ^{-3}]	0.27/−0.28
Extinction coefficient	0.0019(2)
Flack X parameter	0.01(4)

7.7. **4**

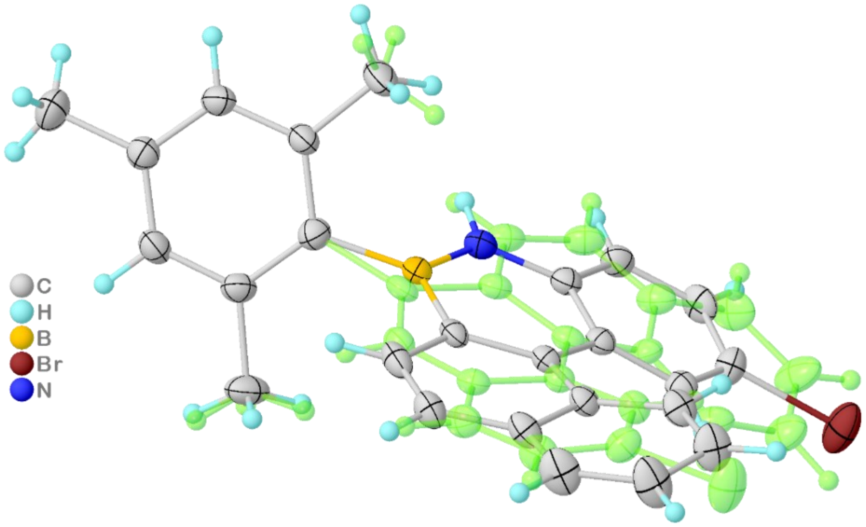

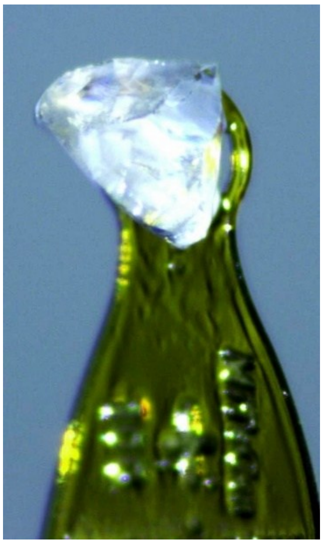

Figure S45: Full asymmetric unit of **4**. Anisotropic displacement ellipsoids drawn at 50% probability level; disordered molecule parts in PART 2 drawn in green hue. The BN-tetrahelicene moiety exhibits local pseudosymmetry, with both disorder parts having an occupancy of 50%. Single crystals were obtained from a solution in dichloromethane.

CCDC number	2506348
Empirical formula	$C_{25}H_{21}BBrN$
Formula weight	426.15
Temperature [K]	100.00
Crystal system	Tetragonal
Space group (number)	$P4_3$ (78)
a [Å]	10.2945(4)
b [Å]	10.2945(4)
c [Å]	18.5686(12)
α [°]	90
β [°]	90
γ [°]	90
Volume [Å^3]	1967.8(2)
Z	4
ρ_{calc} [gcm^{-3}]	1.438
μ [mm^{-1}]	2.099
F(000)	872
Crystal size [mm^3]	0.443×0.43×0.42
Crystal colour	Colourless
Crystal shape	Block
Radiation	MoK_α (λ=0.71073 Å)
2θ range [°]	3.96 to 63.23 (0.68 Å)
Index ranges	$-14 \leq h \leq 15$<br>$-15 \leq k \leq 9$<br>$-27 \leq l \leq 27$

Reflections collected	43027
Independent reflections	6505<br>R_{int} = 0.0557<br>R_{sigma} = 0.0292
Completeness to θ = 25.242°	99.8 %
Data / Restraints / Parameters	6505/1/430
Absorption correction T_{min}/T_{max} (method)	0.4858/0.6534 (numerical)
Goodness-of-fit on F^2	1.043
Final R indexes [$I \geq 2\sigma(I)$]	R_1 = 0.0375<br>wR_2 = 0.0870
Final R indexes [all data]	R_1 = 0.0421<br>wR_2 = 0.0888
Largest peak/hole [eÅ^{-3}]	0.24/−0.33
Flack X parameter	0.001(6)
Extinction coefficient	0.0048(14)

7.8. **S4(NMe)**

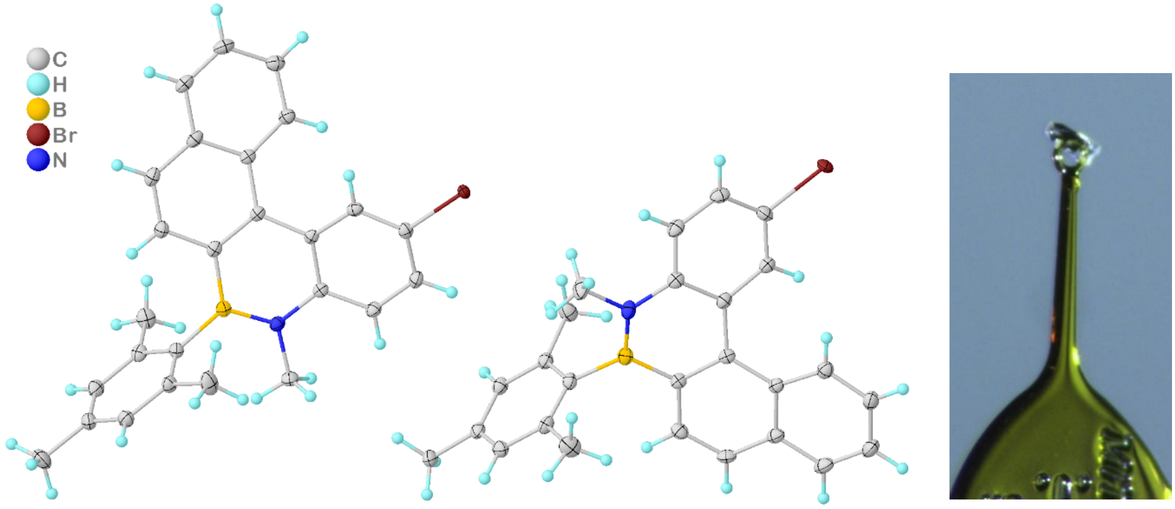

Figure S46: Full asymmetric unit of **S4(NMe)** (Z'=2). Anisotropic displacement ellipsoids drawn at 50% probability level. Single crystals were obtained from a solution in dichloromethane.

CCDC number	2506358
Empirical formula	$C_{26}H_{23}BBrN$
Formula weight	440.17
Temperature [K]	100.00
Crystal system	Monoclinic
Space group (number)	$P2_1/c$ (14)
a [Å]	16.0992(19)
b [Å]	15.892(3)
c [Å]	16.165(3)
α [°]	90
β [°]	92.065(5)
γ [°]	90
Volume [Å^3]	4133.3(12)
Z	8
ρ_{calc} [gcm^{-3}]	1.415
μ [mm^{-1}]	2.001
F(000)	1808
Crystal size [mm^3]	0.162×0.093×0.059
Crystal colour	Colourless
Crystal shape	Needle
Radiation	MoK_α (λ=0.71073 Å)
2θ range [°]	4.34 to 61.10 (0.70 Å)

Index ranges	$-23 \leq h \leq 19$<br>$-22 \leq k \leq 22$<br>$-23 \leq l \leq 23$
Reflections collected	91001
Independent reflections	12614<br>R_{int} = 0.0553<br>R_{sigma} = 0.0381
Completeness to θ = 25.242°	99.9 %
Data / Restraints / Parameters	12614/0/531
Absorption correction T_{min}/T_{max} (method)	0.8030/0.9243 (numerical)
Goodness-of-fit on F^2	1.113
Final *R* indexes [$I \geq 2\sigma(I)$]	R_1 = 0.0439<br>wR_2 = 0.0935
Final *R* indexes [all data]	R_1 = 0.0601<br>wR_2 = 0.1007
Largest peak/hole [eÅ^{-3}]	1.04/−0.88

7.9. **S4(BPh)**

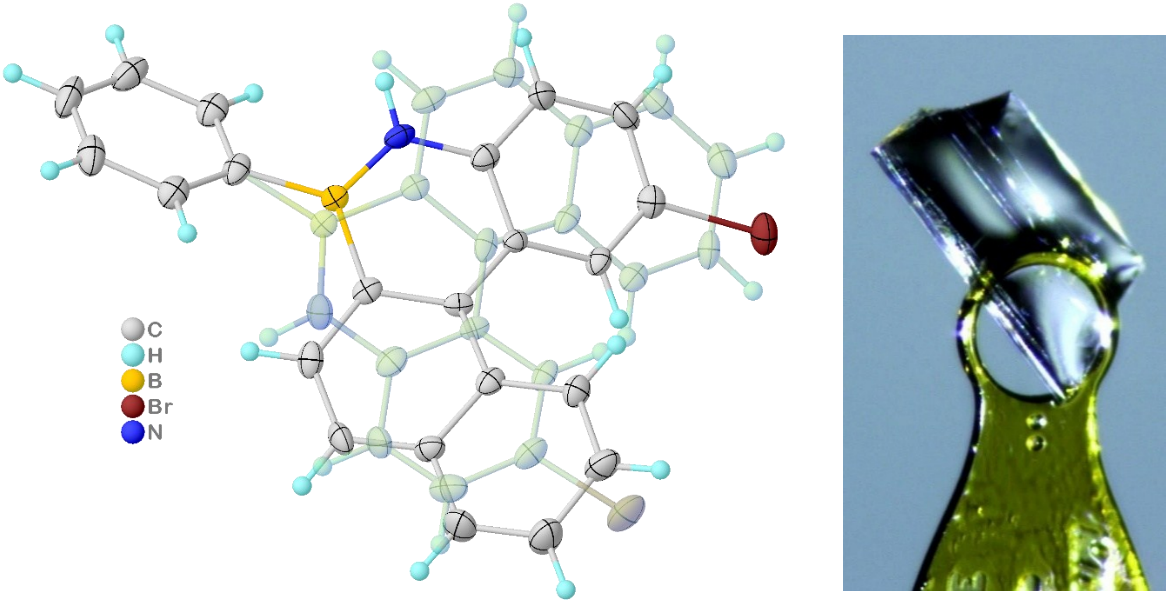

Figure S47: Full asymmetric unit of **S4(BPh)**. Anisotropic displacement ellipsoids drawn at 50% probability level; disordered molecule parts in PART 2 drawn in green hue. The BN-tetrahelicene moiety exhibits local pseudosymmetry, with both disorder parts having an occupancy of approximately 50%. Non-merohedral twinning was found, with both domains related by (0.494 0 0.514 / 0 -1 0 / 1.472 0 -0.494) which corresponds to a rotation of 180 °; final refinement was carried out against hklf5 data, with a refined batch scale factor of 0.3977(14). Crystals were obtained from a mixture of dichloromethane and hexane.

CCDC number	2506357
Empirical formula	$C_{22}H_{15}BBrN$
Formula weight	384.07
Temperature [K]	100.00
Crystal system	Monoclinic
Space group (number)	$P2_1/n$ (14)
a [Å]	11.2579(11)
b [Å]	7.1245(6)
c [Å]	21.2105(18)
α [°]	90
β [°]	100.818(4)
γ [°]	90
Volume [Å^3]	1671.0(3)
Z	4
ρ_{calc} [gcm^{-3}]	1.527
μ [mm^{-1}]	2.463
F(000)	776
Crystal size [mm^3]	0.777×0.364×0.064
Crystal colour	Colourless
Crystal shape	Plate
Radiation	MoK_α (λ=0.71073 Å)
2θ range [°]	3.91 to 56.57 (0.75 Å)

Index ranges	$-15 \leq h \leq 14$<br>$0 \leq k \leq 9$<br>$0 \leq l \leq 28$
Reflections collected	4149
Independent reflections	4149<br>R_{int} = 0.0463<br>R_{sigma} = 0.0327
Completeness to θ = 25.242°	100.0 %
Data / Restraints / Parameters	4149/300/399
Absorption correction T_{min}/T_{max} (method)	0.143251/0.209190 (multi-scan)
Goodness-of-fit on F^2	1.104
Final *R* indexes [$I \geq 2\sigma(I)$]	R_1 = 0.0398<br>wR_2 = 0.0926
Final *R* indexes [all data]	R_1 = 0.0564<br>wR_2 = 0.1013
Largest peak/hole [eÅ^{-3}]	0.42/−0.32

7.10. **5a**

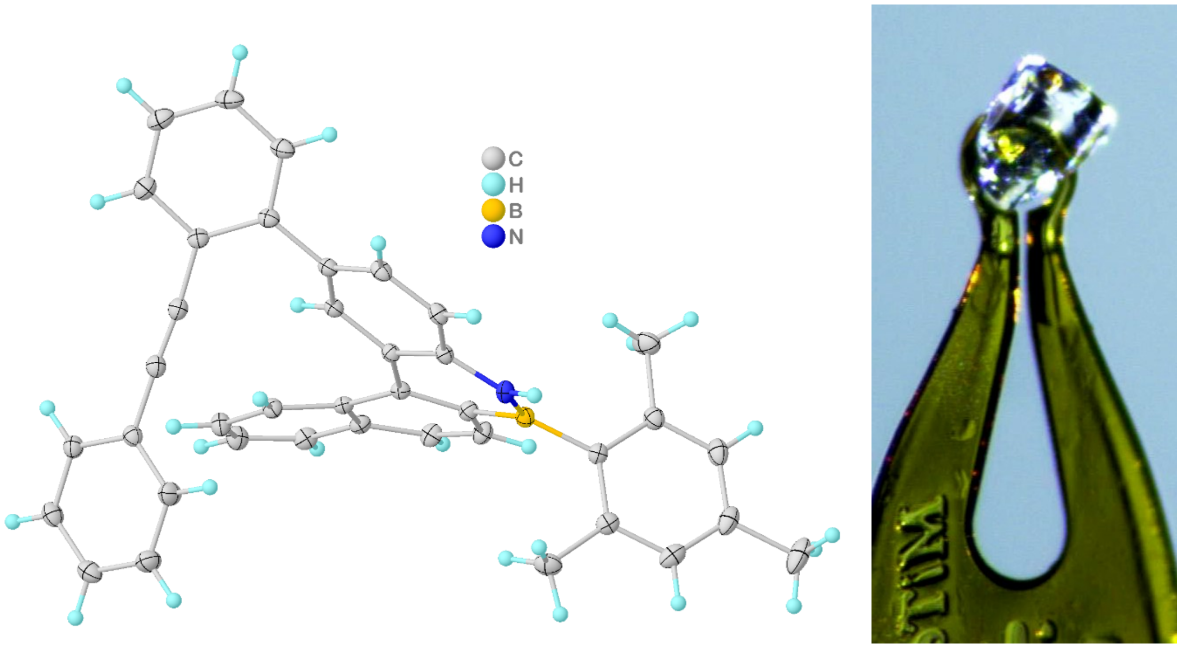

Figure S48: Full asymmetric unit of **5a**. Anisotropic displacement ellipsoids drawn at 50% probability level. Single crystals were obtained from a solution in acetonitrile.

CCDC number	2506349
Empirical formula	$C_{39}H_{30}BN$
Formula weight	523.45
Temperature [K]	100.00
Crystal system	Trigonal
Space group (number)	$R\bar{3}:H$ (148)
a [Å]	37.971(2)
b [Å]	37.971(2)
c [Å]	10.0812(7)
α [°]	90
β [°]	90
γ [°]	120
Volume [$Å^3$]	12587.4(16)
Z	18
ρ_{calc} [gcm^{-3}]	1.243
μ [mm^{-1}]	0.071
F(000)	4968
Crystal size [mm^3]	0.301×0.181×0.12
Crystal colour	Colourless
Crystal shape	Block
Radiation	MoK_α (λ=0.71073 Å)
2θ range [°]	4.23 to 61.02 (0.70 Å)

Index ranges	$-54 \leq h \leq 54$<br>$-54 \leq k \leq 54$<br>$-14 \leq l \leq 14$
Reflections collected	119699
Independent reflections	8555<br>R_{int} = 0.0363<br>R_{sigma} = 0.0154
Completeness to θ = 25.242°	100.0 %
Data / Restraints / Parameters	8555/1/377
Absorption correction T_{min}/T_{max} (method)	0.9705/0.9952 (numerical)
Goodness-of-fit on F^2	1.023
Final R indexes [$I \geq 2\sigma(I)$]	R_1 = 0.0424<br>wR_2 = 0.1099
Final R indexes [all data]	R_1 = 0.0493<br>wR_2 = 0.1158
Largest peak/hole [$eÅ^{-3}$]	0.45/−0.22

7.11. **5c**∘$HCCl_3$

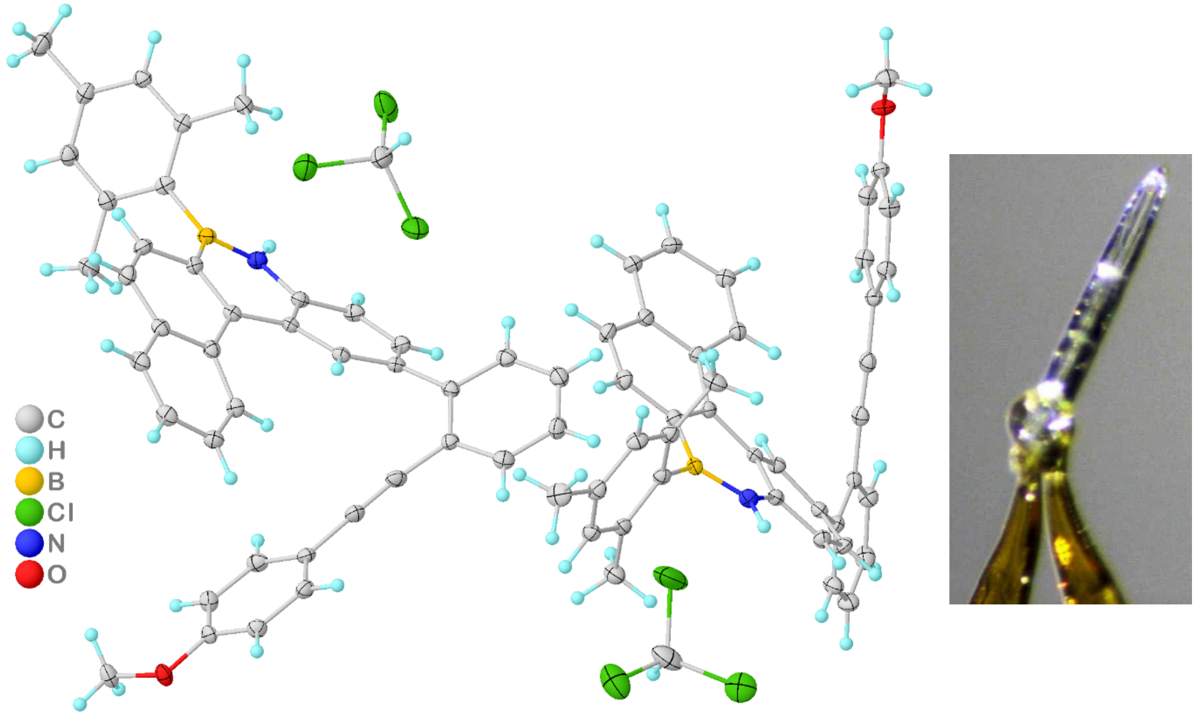

Figure S49: Full asymmetric unit of **5c**∘$HCCl_3$ (Z'=2). Anisotropic displacement ellipsoids drawn at 50% probability level. Single crystals were obtained from a solution in chloroform.

CCDC number	2506350
Empirical formula	$C_{41}H_{33}BCl_3NO$
Formula weight	672.84
Temperature [K]	100.00
Crystal system	Orthorhombic
Space group (number)	$Pca2_1$ (29)
a [Å]	13.0664(5)
b [Å]	27.9409(12)
c [Å]	18.5017(8)
α [°]	90
β [°]	90
γ [°]	90
Volume [Å^3]	6754.7(5)
Z	8
ρ_{calc} [gcm^{-3}]	1.323
μ [mm^{-1}]	0.306
F(000)	2800
Crystal size [mm^3]	1.292×0.054×0.05
Crystal colour	Colourless
Crystal shape	Needle
Radiation	MoK_α (λ=0.71073 Å)
2θ range [°]	4.09 to 55.02 (0.77 Å)
Index ranges	$-16 \le h \le 16$<br>$-36 \le k \le 36$<br>$-24 \le l \le 24$

Reflections collected	231402
Independent reflections	15511<br>R_{int} = 0.1086<br>R_{sigma} = 0.0462
Completeness to θ = 25.242°	100.0 %
Data / Restraints / Parameters	15511/3/863
Absorption correction T_{min}/T_{max} (method)	0.7924/1.0000 (numerical)
Goodness-of-fit on F^2	1.074
Final R indexes [$I \ge 2\sigma(I)$]	R_1 = 0.0466<br>wR_2 = 0.1092
Final R indexes [all data]	R_1 = 0.0650<br>wR_2 = 0.1189
Largest peak/hole [eÅ^{-3}]	0.63/−0.55
Flack X parameter	0.44(6)
Extinction coefficient	0.0019(2)

7.12. **7h**

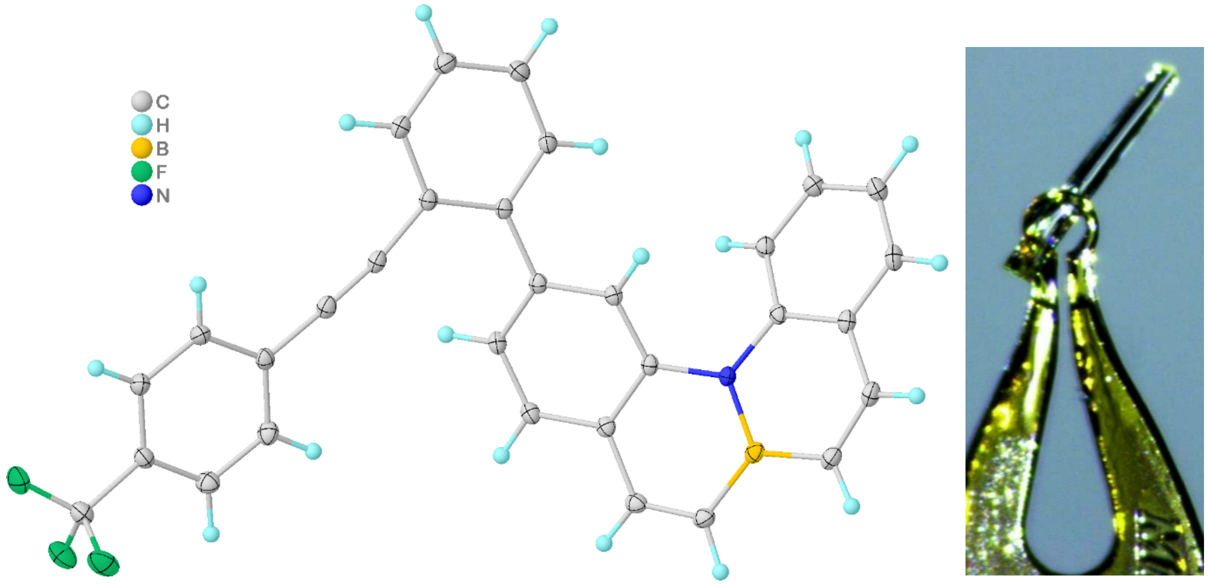

Figure S50: Full asymmetric unit of **7h**. Anisotropic displacement ellipsoids drawn at 50% probability level. Single crystals were obtained from a solution in dichloromethane.

CCDC number	2506351
Empirical formula	$C_{31}H_{19}BF_3N$
Formula weight	473.28
Temperature [K]	100.00
Crystal system	Monoclinic
Space group (number)	$P2_1/c$ (14)
a [Å]	21.3134(10)
b [Å]	14.1947(6)
c [Å]	7.4674(3)
α [°]	90
β [°]	94.591(2)
γ [°]	90
Volume [Å^3]	2251.92(17)
Z	4
ρ_{calc} [gcm^{-3}]	1.396
μ [mm^{-1}]	0.098
F(000)	976
Crystal size [mm^3]	0.053×0.067×0.649
Crystal colour	Colourless
Crystal shape	Needle
Radiation	MoK_α (λ=0.71073 Å)

2θ range [°]	3.83 to 61.04 (0.70 Å)
Index ranges	−30 ≤ h ≤ 30<br>−20 ≤ k ≤ 19<br>−9 ≤ l ≤ 10
Reflections collected	78638
Independent reflections	6887<br>R_{int} = 0.0448<br>R_{sigma} = 0.0207
Completeness to θ = 25.242°	100.0 %
Data / Restraints / Parameters	6887 / 0 / 401
Absorption correction T_{min}/T_{max} (method)	0.7121 / 0.7461 (numerical)
Goodness-of-fit on F^2	1.042
Final *R* indexes [I≥2σ(I)]	R_1 = 0.0400<br>wR_2 = 0.1005
Final *R* indexes [all data]	R_1 = 0.0505<br>wR_2 = 0.1087
Largest peak/hole [eÅ^{-3}]	0.43/−0.29

7.13. **S10b**(NMe)◦0.5CH_2Cl_2

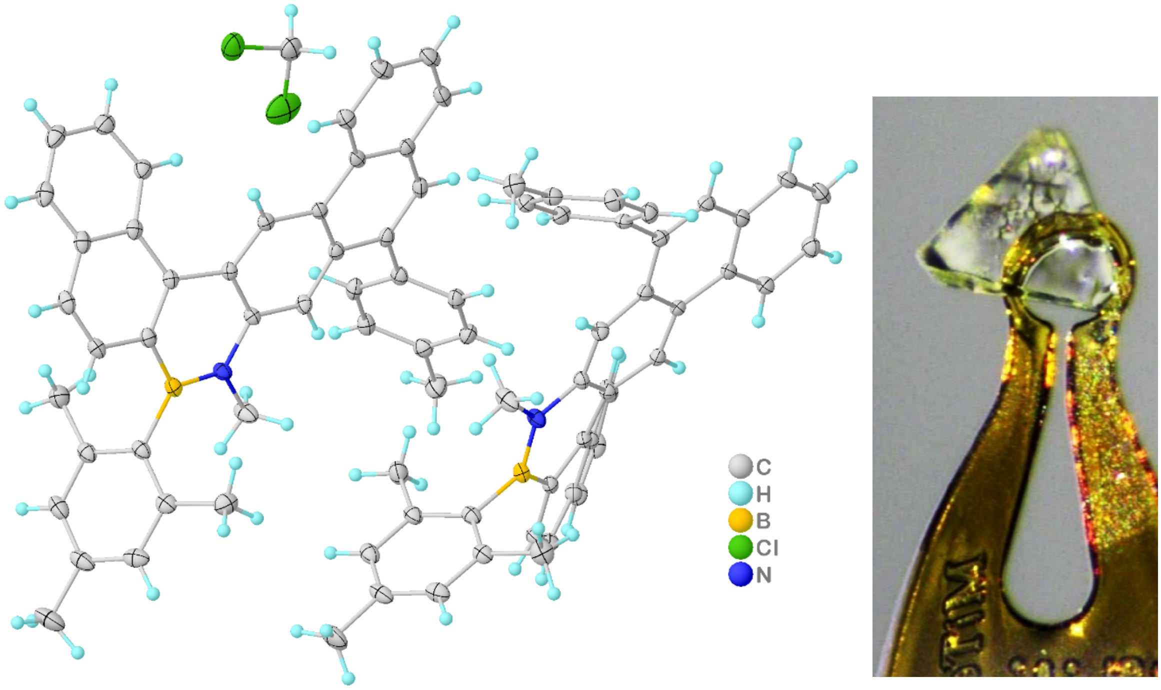

Figure S51: Full asymmetric unit of **S10b(NMe)**◦0.5CH_2Cl_2 (Z'=2). Anisotropic displacement ellipsoids drawn at 50% probability level. Non-merohedral twinning was found, with both domains related by (-1 0 0.008 / 0 -1 0 / 0.097 0 1) which corresponds to a rotation of 180 °; final refinement was carried out against hklf5 data, with a refined batch scale factor of 0.4314(7). Crystals were obtained from a solution in dichloromethane.

CCDC number	2506352
Empirical formula	$C_{83}H_{70}B_2Cl_2N_2$
Formula weight	1187.93
Temperature [K]	100.00
Crystal system	Monoclinic
Space group (number)	$P2_1/n$ (14)
a [Å]	20.1693(7)
b [Å]	12.1225(4)
c [Å]	25.9034(8)
α [°]	90
β [°]	91.8780(10)
γ [°]	90
Volume [Å^3]	6330.0(4)
Z	4
ρ_{calc} [gcm^{-3}]	1.246
μ [mm^{-1}]	0.152
F(000)	2504
Crystal size [mm^3]	0.395×0.386×0.06
Crystal colour	Colourless
Crystal shape	Plate
Radiation	MoK_α (λ=0.71073 Å)
2θ range [°]	3.71 to 54.38 (0.78 Å)

Index ranges	$-25 \le h \le 25$<br>$0 \le k \le 15$<br>$0 \le l \le 33$
Reflections collected	14435
Independent reflections	14435<br>R_{int} = 0.0625<br>R_{sigma} = 0.0275
Completeness to θ = 25.242°	99.9 %
Data / Restraints / Parameters	14435/0/813
Absorption correction T_{min}/T_{max} (method)	0.562570/0.801361 (multi-scan)
Goodness-of-fit on F^2	1.029
Final R indexes [$I \geq 2\sigma(I)$]	R_1 = 0.0419<br>wR_2 = 0.0974
Final R indexes [all data]	R_1 = 0.0492<br>wR_2 = 0.1023
Largest peak/hole [eÅ^{-3}]	0.49/−0.68

7.14. **11b**

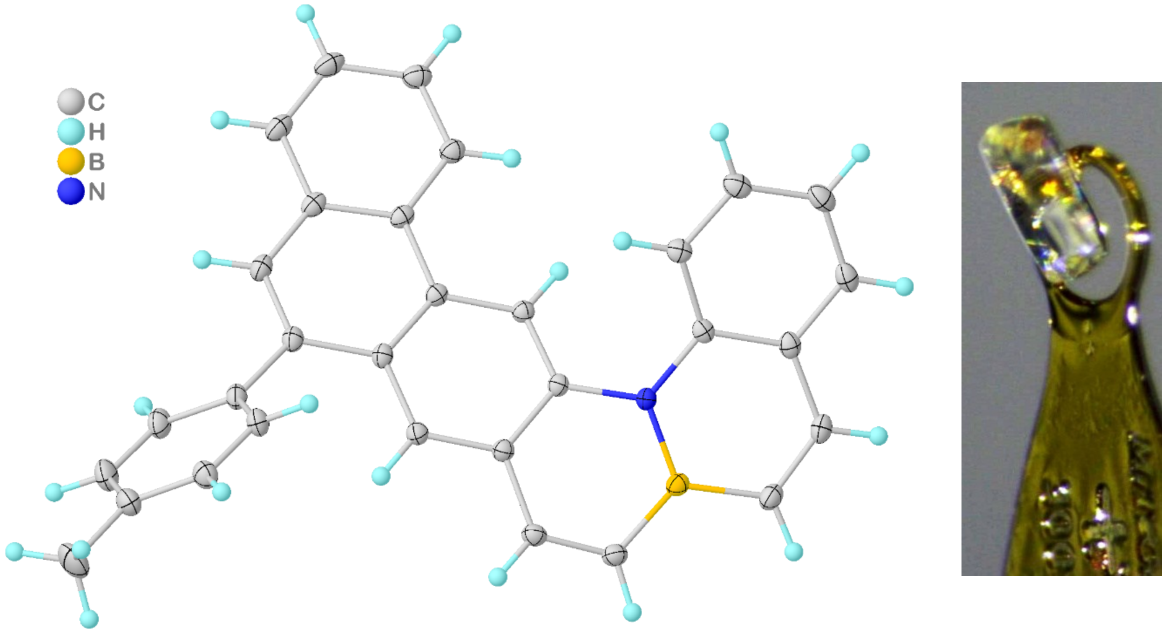

Figure S52: Full asymmetric unit of **11b**. Anisotropic displacement ellipsoids drawn at 50% probability level. Single crystals were obtained from a mixture of dichloromethane and methanol.

CCDC number	2506353
Empirical formula	$C_{31}H_{22}BN$
Formula weight	419.30
Temperature [K]	100.00
Crystal system	Hexagonal
Space group (number)	$P6_2$ (171)
a [Å]	20.0698(9)
b [Å]	20.0698(9)
c [Å]	9.2934(5)
α [°]	90
β [°]	90
γ [°]	120
Volume [Å^3]	3241.8(3)
Z	6
ρ_{calc} [gcm^{-3}]	1.289
μ [mm^{-1}]	0.073
F(000)	1320
Crystal size [mm^3]	0.16×0.181×0.441
Crystal colour	Colourless
Crystal shape	Block
Radiation	MoK_α (λ=0.71073 Å)
2θ range [°]	4.06 to 61.15 (0.70 Å)

Index ranges	−28 ≤ h ≤ 28<br>−28 ≤ k ≤ 28<br>−13 ≤ l ≤ 13
Reflections collected	119816
Independent reflections	6627<br>R_{int} = 0.0423<br>R_{sigma} = 0.0138
Completeness to θ = 25.242°	99.9 %
Data / Restraints / Parameters	6627 / 1 / 299
Absorption correction T_{min}/T_{max} (method)	0.8221 / 1.0000 (numerical)
Goodness-of-fit on F^2	1.023
Final R indexes [I≥2σ(I)]	R_1 = 0.0364<br>wR_2 = 0.1022
Final R indexes [all data]	R_1 = 0.0373<br>wR_2 = 0.1033
Largest peak/hole [eÅ^{-3}]	0.35/−0.17
Flack X parameter	-0.9(10)

7.15. **12b**

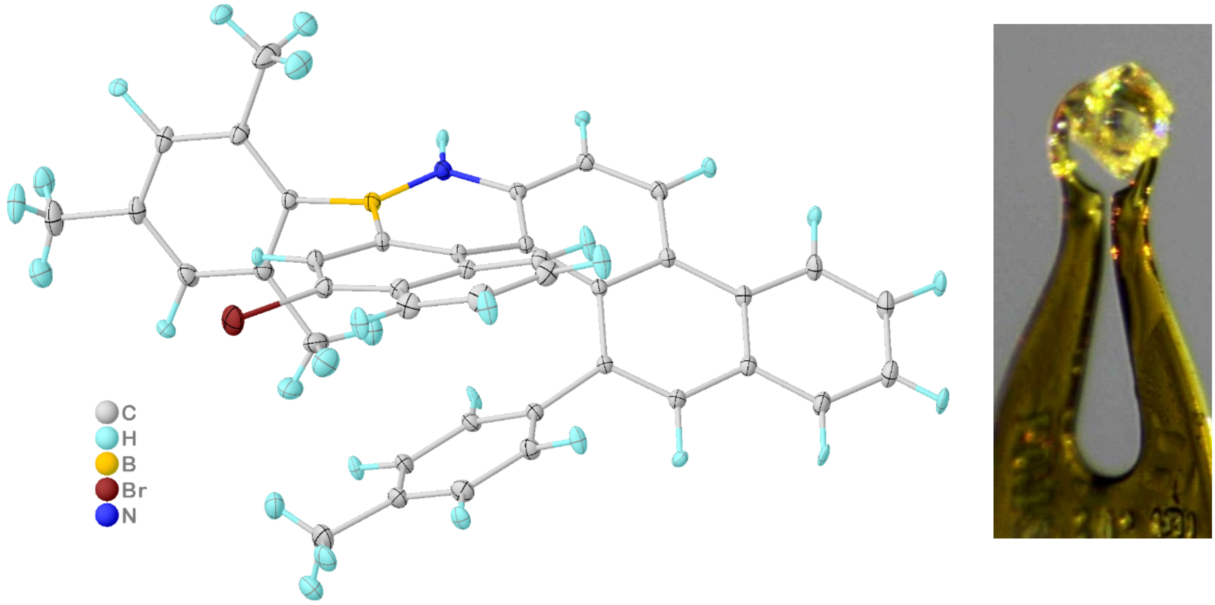

Figure S53: Full asymmetric unit of **12b**. Anisotropic displacement ellipsoids drawn at 50% probability level. Aspherical atomic form factors were used within the frameworks of NoSpherA2 implementation of HAR in OLEX^2 using the R^{2}SCAN method and x2c-TZVP basis set.[19] Single crystals were obtained from a mixture of dichloromethane and acetonitrile by solvent vapor diffusion method.

CCDC number	2506354
Empirical formula	$C_{40}H_{31}BBrN$
Formula weight	616.426
Temperature [K]	100.00
Crystal system	Triclinic
Space group (number)	$P\bar{1}$ (2)
a [Å]	8.4889(5)
b [Å]	13.7216(8)
c [Å]	14.3661(9)
α [°]	116.387(2)
β [°]	94.687(2)
γ [°]	97.658(2)
Volume [Å^3]	1466.83(16)
Z	2
ρ_{calc} [gcm^{-3}]	1.396
μ [mm^{-1}]	1.432
F(000)	635.913
Crystal size [mm^3]	0.149×0.199×0.202
Crystal colour	Yellow
Crystal shape	Block
Radiation	Mo K_α (λ=0.71073 Å)

2θ range [°]	4.90 to 72.74 (0.60 Å)
Index ranges	$-13 \le h \le 13$<br>$-19 \le k \le 22$<br>$-23 \le l \le 23$
Reflections collected	123822
Independent reflections	13713<br>R_{int} = 0.0445<br>R_{sigma} = 0.0268
Completeness to θ = 25.2417°	99.9 %
Data / Restraints / Parameters	13713 / 657 / 667
Absorption correction T_{min}/T_{max} (method)	0.7468 / 0.9807 (numerical)
Goodness-of-fit on F^2	1.0477
Final *R* indexes [$I \geq 2\sigma(I)$]	R_1 = 0.0302<br>wR_2 = 0.0774
Final *R* indexes [all data]	R_1 = 0.0380<br>wR_2 = 0.0804
Largest peak/hole [eÅ^{-3}]	1.34/−0.98

7.16. **12b**◦[0.5 CH_2Cl_2; 0.5 MeCN]

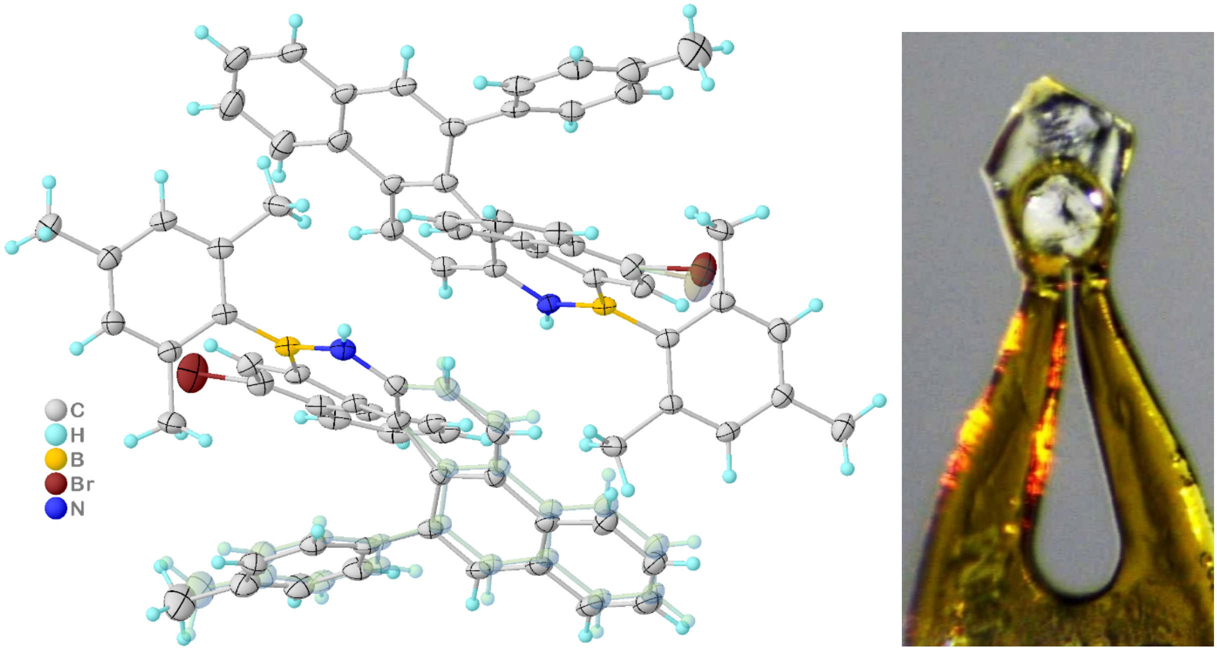

Figure S54: Full asymmetric unit of **12b**◦[0.5 CH_2Cl_2; 0.5 MeCN] (Z'=2). Anisotropic displacement ellipsoids drawn at 50% probability level; disordered molecule parts in PART 2 drawn in green hue. The co-crystalized solvent molecules are located in a continuous cavity along the a axis and could not be modelled; solvent mask was used to remove the unmodelled electron density. Single crystals were obtained from a mixture of dichloromethane and acetonitrile by solvent vapor diffusion method.

CCDC number	2506355
Empirical formula	$C_{41.50}H_{33.50}BBrClN_{1.50}$
Formula weight	679.37
Temperature [K]	100.00
Crystal system	Monoclinic
Space group (number)	$P2_1/n$ (14)
a [Å]	16.220(7)
b [Å]	13.047(5)
c [Å]	31.385(13)
α [°]	90
β [°]	90.805(8)
γ [°]	90
Volume [$Å^3$]	6641(5)
Z	8
ρ_{calc} [gcm^{-3}]	1.359
μ [mm^{-1}]	1.351
F(000)	2800
Crystal size [mm^3]	0.058×0.278×0.417
Crystal colour	Colourless
Crystal shape	Plate
Radiation	MoK_α (λ=0.71073 Å)

2θ range [°]	4.01 to 57.43 (0.74 Å)
Index ranges	−21 ≤ h ≤ 21<br>−11 ≤ k ≤ 17<br>−42 ≤ l ≤ 42
Reflections collected	182183
Independent reflections	17099<br>R_{int} = 0.0575<br>R_{sigma} = 0.0313
Completeness to θ = 25.242°	100.0 %
Data / Restraints / Parameters	17099 / 391 / 961
Absorption correction T_{min}/T_{max} (method)	0.5811 / 1.0000 (numerical)
Goodness-of-fit on F^2	1.028
Final *R* indexes [I≥2σ(I)]	R_1 = 0.0499<br>wR_2 = 0.1287
Final *R* indexes [all data]	R_1 = 0.0693<br>wR_2 = 0.1415
Largest peak/hole [e$Å^{-3}$]	1.20/−1.17

7.17. **13b**∘1.5 $HCCl_3$

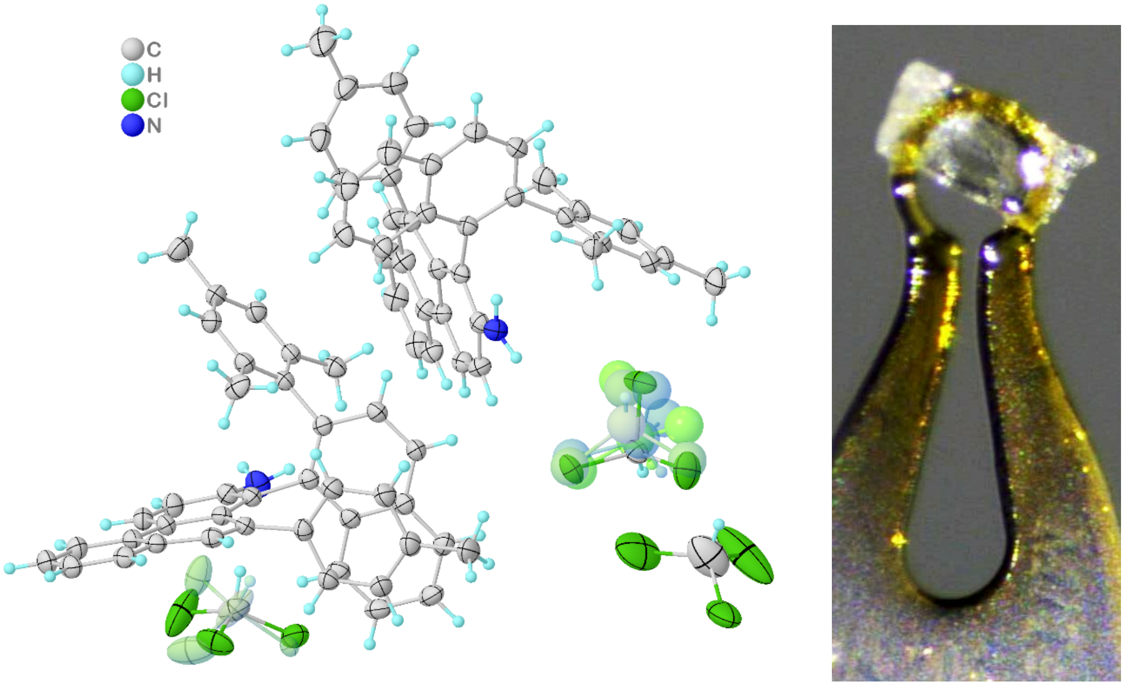

Figure S55: Full asymmetric unit of **13b**∘1.5 $HCCl_3$ (Z'=2). Anisotropic displacement ellipsoids drawn at 50% probability level; disorder parts are drawn in translucent coloured hues. The co-crystalized chloroform exhibits extensive positional disorder and may even partly escape the lattice, because the refined occupancies converge on a non-integer sum formula. Single crystals were obtained from a solution in chloroform.

CCDC number	2506356
Empirical formula	$C_{41.08}H_{34.08}Cl_{3.26}N$
Formula weight	657.19
Temperature [K]	100.00
Crystal system	Orthorhombic
Space group (number)	$P2_12_12_1$ (19)
a [Å]	11.7361(3)
b [Å]	20.0189(5)
c [Å]	28.7963(8)
α [°]	90
β [°]	90
γ [°]	90
Volume [Å^3]	6765.5(3)
Z	8
ρ_{calc} [gcm^{-3}]	1.290
μ [mm^{-1}]	2.862
F(000)	2743
Crystal size [mm^3]	0.082×0.14×0.327
Crystal colour	Colourless
Crystal shape	Plate
Radiation	CuK_α (λ=1.54178 Å)

2θ range [°]	5.38 to 158.76 (0.78 Å)
Index ranges	−14 ≤ h ≤ 14<br>−25 ≤ k ≤ 22<br>−36 ≤ l ≤ 36
Reflections collected	158797
Independent reflections	14564<br>R_{int} = 0.0612<br>R_{sigma} = 0.0266
Completeness to θ = 67.679°	100.0 %
Data / Restraints / Parameters	14564 / 187 / 906
Absorption correction T_{min}/T_{max} (method)	0.4894 / 0.8796 (numerical)
Goodness-of-fit on F^2	1.030
Final R indexes [I≥2σ(I)]	R_1 = 0.0576<br>wR_2 = 0.1456
Final R indexes [all data]	R_1 = 0.0606<br>wR_2 = 0.1488
Largest peak/hole [eÅ^{-3}]	0.77/−0.91
Flack X parameter	-0.007(4)

8. Computational Details

All geometry optimisations were carried out with Gaussian 16, Rev A03.[20] For this purpose, the B3LYP functional[21] and Karlsruhe def2-TZVP basis set[22] were used. Grimme's D3 correction[23] and Becke-Johnson damping[24] were included. Solvation effects were accounted for through the polarizable conductor calculation model[25] with dichloromethane as solvent. For thermodynamic modelling a temperature of 473.15K was assumed. The rigid-rotor-harmonic-oscillator approximation was used to compute vibrational entropy corrections for all structures.[26]

For the theoretical UV-Vis spectra, the simplified TD-DFT (sTD-DFT)[27] approach of Bannwarth and Grimme was applied, computed with the ORCA 6.0.1 program package.[28] The functional used was the CAM-B3LYP functional.[29] The excitation energies were all shifted by -0.4 eV. Such corrections are commonly used in the description of such conjugated systems. The intensities are based on the length gauge of the oscillator strengths.

The absolute energy contributions for calculating $\Delta G, \Delta H$ and ΔS are listed in the table below:

	Electronic Energy [kcal/mol]	Zero-Point Energy [kcal/mol]	Thermal Energy [kcal/mol]	Entropy [cal/mol]
(M)-**14**	-797828.95	277.61	35.02	212.44
(M)-**1b**	-1019107.28	378.52	50.09	277.43
**1b** TS	-1019068.38	377.97	49.37	274.94
(M)-**2b**	-799979.10	276.24	35.39	213.88
**2b** TS	-799939.06	275.41	34.77	212.27

The computed electronic excitations of **1b**, **2b** and **14** are provided below. The energies (E, in eV) listed are non-shifted (direct sTD-DFT result), the wavelengths (λ, in nm) are computed from the shifted excitation energies.

**1b**			**2b**			**14**		
E	λ (shift)	f_L	E	λ (shift)	f_L	E	λ (shift)	f_L
3.515	398.0	0.324505	3.557	392.7	0.029898	3.456	405.7	0.001664
3.799	364.8	0.078371	3.793	365.4	0.00484	3.675	378.6	0.085024
3.918	352.4	0.025728	4.032	341.4	0.559382	3.914	352.8	0.061847
4.025	342.0	0.023748	4.145	331.1	0.093615	4.022	342.3	0.546406
4.233	323.5	0.051999	4.162	329.6	0.072431	4.078	337.1	0.042929
4.363	312.9	0.283864	4.37	312.3	0.121426	4.24	322.9	0.209581
4.404	309.7	0.033272	4.423	308.2	0.16836	4.295	318.3	0.236448
4.587	296.1	0.439291	4.583	296.4	0.034112	4.502	302.3	0.611522
4.639	292.5	0.048014	4.653	291.5	0.757247	4.643	292.2	0.100805
4.714	287.4	0.641757	4.74	285.7	0.041026	4.689	289.1	0.137848
4.757	284.6	0.188872	4.776	283.3	0.222168	4.771	283.7	0.028257
4.818	280.6	0.051941	4.892	276.0	0.214128	4.845	278.9	0.078098
4.864	277.7	0.102019	4.932	273.6	0.057294	4.943	272.9	0.013927
4.935	273.4	0.063632	5.004	269.3	0.007744	5.044	267.0	0.054056
4.99	270.1	0.020134	5.068	265.6	0.214568	5.092	264.2	0.064119
5.05	266.6	0.00581	5.088	264.5	0.078841	5.136	261.8	0.020381
5.125	262.4	0.064025	5.146	261.2	0.07331	5.154	260.8	0.089886
5.141	261.5	0.09924	5.222	257.1	0.037699	5.188	258.9	0.029653
5.216	257.4	0.022199	5.268	254.7	0.027893	5.241	256.1	0.232253
5.278	254.2	0.068933	5.357	250.1	0.0789	5.332	251.4	0.122102
5.297	253.2	0.139762	5.432	246.4	0.057426	5.372	249.4	0.051611

XYZ Structures and Gibbs free energies

System *(M)*-**14**

G=-797613.07 kcal/mol

55

C	2.885657000	0.045671000	1.439704000
C	1.499375000	0.333316000	1.299806000
C	-0.264243000	1.650895000	0.018063000
C	-1.255230000	0.612671000	-0.162233000
C	-0.917170000	-0.759346000	-0.485202000
C	0.426365000	-1.162292000	-0.969154000
C	1.033082000	-2.333654000	-0.514735000
H	0.535262000	-2.932405000	0.235283000
C	2.290857000	-2.708739000	-0.963537000
H	2.747715000	-3.609430000	-0.571239000
C	2.987674000	-1.932182000	-1.887706000
C	2.362752000	-0.782826000	-2.373213000
H	2.870784000	-0.170296000	-3.108632000
C	1.106595000	-0.404288000	-1.925721000
H	0.651983000	0.496371000	-2.315430000
C	-1.899596000	-1.707145000	-0.470789000
H	-1.652959000	-2.726118000	-0.740724000
C	-3.258644000	-1.404645000	-0.190890000
C	-3.644833000	-0.046156000	-0.064342000
C	-2.623759000	0.976087000	-0.131222000
C	-2.972428000	2.350696000	-0.224058000
H	-4.012550000	2.638109000	-0.245860000
C	-2.017211000	3.304366000	-0.399549000
H	-2.295769000	4.332718000	-0.592665000
C	-0.641767000	2.980918000	-0.293054000
C	0.356632000	3.968185000	-0.513374000
H	0.044640000	4.964875000	-0.798933000
C	1.674968000	3.646373000	-0.421672000
H	2.442026000	4.368839000	-0.670703000
C	-5.011260000	0.238917000	0.140209000
H	-5.340821000	1.260703000	0.257220000
C	-5.945038000	-0.769584000	0.225855000
H	-6.984964000	-0.523815000	0.397188000
C	-5.556691000	-2.112965000	0.096215000
H	-6.297440000	-2.899201000	0.160983000
C	-4.233744000	-2.421027000	-0.113320000
H	-3.919486000	-3.451821000	-0.221920000
C	0.591697000	-0.414391000	2.079983000
H	-0.455720000	-0.156897000	2.066451000

C	1.016015000	-1.457234000	2.868918000
H	0.296698000	-2.008803000	3.460313000
C	2.374883000	-1.808707000	2.913223000
H	2.699241000	-2.646324000	3.516866000
C	3.291832000	-1.056953000	2.219385000
H	4.349220000	-1.282254000	2.285369000
C	4.381905000	-2.296127000	-2.316085000
H	5.122158000	-1.804314000	-1.677862000
H	4.578336000	-1.982070000	-3.341990000
H	4.552504000	-3.370827000	-2.245254000
C	3.846566000	0.918779000	0.853691000
C	3.452082000	2.078132000	0.268366000
H	4.897416000	0.676098000	0.950657000
H	4.182820000	2.793009000	-0.089106000
C	1.090278000	1.430665000	0.452715000
C	2.070547000	2.381562000	0.092054000

System *(M)*-**1b**

G=-1018806.18 kcal/mol

74

C	3.204188163	-0.7440830053	-1.6735753597
C	1.8010640316	-0.8533019566	-1.4453199325
C	1.3345122499	-1.7299137082	-0.4018887766
C	-0.042215932	-1.7108720797	0.0818503829
C	-0.8776209699	-0.5392444502	0.1383410259
C	-0.3443656617	0.8082665357	0.2324438943
C	1.0652548362	1.0888264719	0.6001783739
C	1.7951165757	2.0819489755	-0.0537764898
H	1.3386124686	2.6257604236	-0.8692465147
C	3.1168557762	2.3362548989	0.2816467228
H	3.6668980616	3.0933017666	-0.2644382502
C	3.7579922223	1.6128125115	1.2858599063
C	3.0143481551	0.6499471849	1.9689807154
H	3.4795880319	0.0845551921	2.7677715271
C	1.6937116649	0.3930295651	1.6364231794
H	1.1475225548	-0.3678289589	2.1768668707
C	-1.1917974183	1.8701902536	0.1081175894
H	-0.7959345412	2.8728165707	0.20940734
C	-2.5947262832	1.7266950311	-0.0687318676

C	-3.1655031861	0.4333269981	0.0274650366
C	-2.2869150295	-0.7033809934	0.208649304
C	-2.8042964192	-1.9873441816	0.4920204196
H	-3.8700646231	-2.1312411651	0.5835861069
C	-1.974343623	-3.0423226116	0.7461208181
H	-2.3766785459	-3.9962430993	1.0639948373
C	-0.5845469668	-2.9134796171	0.5662880004
H	-0.2303560007	-4.7871691681	1.2461502625
C	-4.5670502099	0.3191520379	-0.0781155218
H	-5.0383573795	-0.6513728164	-0.0266937774
C	-5.3608143401	1.4275461311	-0.277216678
H	-6.4328058563	1.3088845567	-0.3674309002
C	-4.7894224461	2.7066264419	-0.3679640977
H	-5.4200846222	3.5725194743	-0.5213059906
C	-3.4262199174	2.8494599864	-0.2604746411
H	-2.9694402525	3.8297192212	-0.3216509639
C	0.9340856987	-0.1495868055	-2.3122854285
H	-0.1321073401	-0.2833532949	-2.2169688882
C	1.4216131832	0.6907987459	-3.2819622488
H	0.7349673568	1.2117697234	-3.9365532592
C	2.808266611	0.8776103011	-3.4307875605
H	3.183933324	1.5615901708	-4.1808232329
C	3.678578195	0.1621998137	-2.6485540947
H	4.7485418501	0.2606311064	-2.7851295671
C	5.2137328948	1.8280058694	1.5924354926
H	5.8347226446	1.1351148362	1.0165985654
H	5.4288398239	1.655429165	2.6477799844
H	5.5297026348	2.8398273584	1.3365966926
N	0.2433530615	-3.9727184129	0.8830113924
B	1.6564068669	-3.9043370799	0.8546037251
C	2.5315217409	-5.0597371081	1.454102197
C	3.1159411082	-4.8974440127	2.7222013559
C	2.7820024673	-6.2398632052	0.7362870854
C	3.9164367791	-5.9064136086	3.2524425601
C	3.5910495884	-7.2306359043	1.2906265129
C	4.1632401496	-7.085065907	2.551680235
H	4.3623511333	-5.7683182362	4.2315912832
H	3.7842232066	-8.1342152726	0.7225357279
C	2.1917252848	-6.436686983	-0.637777203

H	1.1057124461	-6.5495164364	-0.5930842051
H	2.3946767896	-5.5782992174	-1.2824712761
H	2.5993892184	-7.3256957369	-1.1186680878
C	2.8855632292	-3.6287915667	3.5049005445
H	3.2577575836	-2.756888122	2.9611610187
H	1.8208008191	-3.4582039682	3.6822987428
H	3.3866674935	-3.6635368076	4.4719874373
C	5.0065414636	-8.1781222259	3.1509266597
H	5.7861006406	-7.7698461746	3.7955488568
H	4.3976580435	-8.8499083253	3.7629020569
H	5.4810200555	-8.7826103499	2.3770927891
C	2.2219445266	-2.6794448253	0.1242393032
C	3.6088185594	-2.5689022064	-0.1536817261
C	4.0975344087	-1.5869125397	-0.963418018
H	5.1620235115	-1.479012987	-1.1322081389

System **1b** Transition State

G=-1018767.37 kcal/mol

74

C	0.315269000	-2.071078000	2.311369000
C	-0.217752000	-1.671431000	1.043025000
C	0.405433000	-0.573989000	0.332198000
C	-0.224341000	0.415521000	-0.587977000
C	-1.537144000	1.024670000	-0.385236000
C	-2.760481000	0.394901000	0.106349000
C	-3.283639000	-0.863564000	-0.473464000
C	-3.049976000	-1.140339000	-1.823126000
H	-2.403012000	-0.489268000	-2.395581000
C	-3.628987000	-2.242816000	-2.437748000
H	-3.423353000	-2.435024000	-3.484031000
C	-4.476290000	-3.099263000	-1.736608000
C	-4.734456000	-2.800027000	-0.396488000
H	-5.387452000	-3.448009000	0.176431000
C	-4.152463000	-1.707835000	0.222919000
H	-4.344076000	-1.529986000	1.272702000
C	-3.667889000	1.149184000	0.796480000
H	-4.583867000	0.684197000	1.136470000
C	-3.586648000	2.565173000	0.880539000
C	-2.670711000	3.209933000	0.014317000

C	-1.682513000	2.407742000	-0.680084000
C	-0.821671000	2.993916000	-1.632437000
H	-1.019621000	3.989210000	-2.000688000
C	0.268973000	2.317950000	-2.082497000
H	0.943871000	2.767647000	-2.799640000
C	0.653254000	1.126645000	-1.435946000
H	2.496469000	1.263354000	-2.268742000
C	1.764217000	-0.365378000	0.636415000
C	-2.710152000	4.616034000	-0.063242000
H	-2.016386000	5.145894000	-0.699491000
C	-3.600384000	5.345809000	0.696264000
H	-3.600189000	6.425967000	0.629001000
C	-4.497897000	4.700283000	1.560177000
H	-5.192685000	5.281328000	2.152337000
C	-4.492955000	3.326606000	1.643207000
H	-5.193065000	2.811490000	2.289438000
C	-1.164885000	-2.529883000	0.456113000
H	-1.441602000	-2.368989000	-0.568927000
C	-1.679815000	-3.613645000	1.130170000
H	-2.385404000	-4.263393000	0.632101000
C	-1.297480000	-3.873591000	2.454207000
H	-1.737075000	-4.701486000	2.995506000
C	-0.304891000	-3.117216000	3.028666000
H	0.078149000	-3.366204000	4.010734000
C	-5.079709000	-4.312891000	-2.387329000
H	-4.981342000	-4.270561000	-3.471963000
H	-4.585604000	-5.226185000	-2.044393000
H	-6.138973000	-4.409108000	-2.142164000
N	1.994567000	0.796897000	-1.527353000
B	2.680133000	0.228243000	-0.436604000
C	4.247388000	0.177352000	-0.395125000
C	4.940207000	-1.002936000	-0.715583000
C	4.979029000	1.312171000	-0.009467000
C	6.331366000	-1.027625000	-0.656801000
C	6.370958000	1.255126000	0.047099000
C	7.066295000	0.092714000	-0.273549000
H	6.855016000	-1.942181000	-0.913739000
H	6.924375000	2.137800000	0.348793000
C	4.183846000	-2.242162000	-1.122994000

C	4.266541000	2.592903000	0.348568000
H	3.555206000	-2.055808000	-1.996966000
H	4.863945000	-3.058677000	-1.364500000
H	3.520693000	-2.578617000	-0.322379000
H	3.790972000	3.043263000	-0.526350000
H	3.476064000	2.416031000	1.081897000
H	4.957256000	3.325196000	0.766085000
C	8.567352000	0.035289000	-0.180762000
H	9.006602000	1.031542000	-0.238954000
H	8.883352000	-0.408562000	0.767641000
H	8.991282000	-0.574956000	-0.979751000
C	1.560612000	-1.548592000	2.746844000
C	2.309063000	-0.836830000	1.860657000
H	3.339226000	-0.588599000	2.083952000

System *(M)*-**2b**

G=-799764.91 kcal/mol

55

C	3.3753807939	-0.5440450604	-0.8003022017
C	1.9740920454	-0.6983637582	-0.6635204592
C	0.1099209797	-1.3216720073	0.8001752185
C	-0.6759957824	-0.1275494394	0.6568284989
C	-0.1129955051	1.2087815146	0.5837944593
C	1.2754497928	1.5279008349	0.9934737033
C	2.04714411	2.4331583352	0.2622992828
H	1.6496647499	2.851279598	-0.6522753413
C	3.335439941	2.7566759378	0.6585536618
H	3.9200618952	3.4419921188	0.0565557446
C	3.9017425576	2.1912275142	1.8001593217
C	3.1179703234	1.312414364	2.5483000108
H	3.5264484806	0.8688021139	3.4484086638
C	1.8295539033	0.9863350534	2.1558430468
H	1.2467150722	0.3001997362	2.7560431427
C	-0.9249550556	2.2529595697	0.2524709865
H	-0.5111821304	3.2529255509	0.2319721948
C	-2.3173181663	2.1083035376	0.0096009055
C	-2.9266457568	0.8535956951	0.2545131354
C	-2.0930827793	-0.2625441913	0.6522694499
C	-2.6702205918	-1.4850079036	1.0636094509

H	-3.743271692	-1.5895116996	1.1019336519
C	-1.8855223811	-2.511422986	1.5022205541
H	-2.3329475855	-3.4032226588	1.9224550658
C	-0.4805999371	-2.4551848969	1.3917436242
C	0.3247186955	-3.512059007	1.9402159523
H	-0.2103552098	-4.3441287131	2.3878792228
C	1.6739879851	-3.4226967021	1.9713981979
H	2.2424892808	-4.1965163693	2.4756036335
C	-4.3216974389	0.7490684802	0.076531804
H	-4.8213568872	-0.1942778371	0.2387014099
C	-5.071884018	1.8297864608	-0.3318840851
H	-6.1390889125	1.718196609	-0.4723974252
C	-4.4617760617	3.0713634337	-0.5698508121
H	-5.0586144029	3.9163219163	-0.8873598006
C	-3.1046734627	3.205406909	-0.3960990482
H	-2.6192114944	4.1580474989	-0.568650777
C	1.1431762127	-0.2134042132	-1.6833348117
H	0.0827545073	-0.4026712441	-1.6473472958
C	1.6639588317	0.5030085272	-2.7420453103
H	0.9972898555	0.8651910064	-3.5136905466
C	3.0359240796	0.7546096844	-2.8245430246
H	3.4380630224	1.3352424594	-3.6439075546
C	3.8724650796	0.216957987	-1.8709309785
H	4.9441641142	0.3547889875	-1.9432713022
C	5.3241760237	2.4822711261	2.1897028767
H	5.999043968	1.7262618568	1.7773279933
H	5.4505344613	2.472837996	3.2730882301
H	5.6498523241	3.452067071	1.8124306188
C	4.2849852056	-1.2174947808	0.0853355668
C	3.8431153893	-2.134270594	0.9737156602
H	5.3413002114	-1.0038722588	-0.0474503936
H	4.5659535557	-2.6864582409	1.5648114002
N	1.4642449792	-1.4094723492	0.4398466178
B	2.345004614	-2.3246820327	1.1594747898

System **2b** Transition State

G=-799725.56 kcal/mol

55

C	-1.975052000	2.131381000	-1.841106000

C	-1.661525000	1.449004000	-0.635542000
C	0.298298000	1.329974000	0.964213000
C	0.988833000	0.140628000	0.485891000
C	0.458672000	-1.013071000	-0.238497000
C	-0.718322000	-1.767431000	0.253105000
C	-0.982021000	-1.807851000	1.624211000
H	-0.388447000	-1.206098000	2.299346000
C	-2.000902000	-2.602852000	2.134140000
H	-2.186558000	-2.605394000	3.201520000
C	-2.779931000	-3.402337000	1.300530000
C	-2.494167000	-3.381747000	-0.067659000
H	-3.087251000	-3.986536000	-0.743745000
C	-1.487144000	-2.584339000	-0.581309000
H	-1.322202000	-2.563279000	-1.650162000
C	1.273403000	-1.682164000	-1.106852000
H	0.893241000	-2.558968000	-1.613786000
C	2.671383000	-1.435739000	-1.188917000
C	3.252983000	-0.659208000	-0.157305000
C	2.386403000	0.074796000	0.746712000
C	2.919277000	0.754217000	1.863969000
H	3.940370000	0.580789000	2.167042000
C	2.141969000	1.627179000	2.558917000
H	2.532442000	2.145462000	3.424944000
C	0.897381000	2.039590000	2.032128000
C	0.451331000	3.343104000	2.446004000
H	0.853889000	3.717977000	3.381986000
C	-0.264876000	4.120380000	1.606770000
H	-0.454464000	5.156514000	1.864316000
C	4.657255000	-0.566390000	-0.106995000
H	5.135127000	0.031628000	0.654873000
C	5.446008000	-1.196596000	-1.046577000
H	6.522314000	-1.096753000	-0.995388000
C	4.862651000	-1.956547000	-2.071010000
H	5.488761000	-2.447969000	-2.804175000
C	3.493025000	-2.078401000	-2.134201000
H	3.029156000	-2.678581000	-2.907186000
C	-2.463663000	0.376954000	-0.261738000
H	-2.368191000	-0.024042000	0.731121000
C	-3.417073000	-0.140416000	-1.124992000

H	-4.029791000	-0.968254000	-0.798628000
C	-3.584016000	0.401966000	-2.395609000
H	-4.306156000	-0.019280000	-3.082340000
C	-2.878933000	1.542731000	-2.735581000
H	-3.082787000	2.052587000	-3.669082000
N	-0.721256000	2.018931000	0.258344000
B	-0.755426000	3.482899000	0.322210000
C	-1.180739000	4.223021000	-0.948097000
C	-1.592219000	3.510980000	-2.018294000
H	-1.041380000	5.294728000	-1.044153000
H	-1.786044000	3.963643000	-2.985683000
C	-3.884036000	-4.265800000	1.844390000
H	-4.832556000	-4.052344000	1.346541000
H	-3.669783000	-5.325560000	1.685003000
H	-4.019293000	-4.107380000	2.913979000

9. References

[1] W. Fu, V. Pelliccioli, M. von Geyso, P. Redero, C. Böhmer, M. Simon, C. Golz, M. Alcarazo, *Adv. Mater.* 2023, **35**, e2211279.

[2] P. Redero, T. Hartung, J. Zhang, L. D. M. Nicholls, G. Zichen, M. Simon, C. Golz, M. Alcarazo, *Angew. Chem. Int. Ed.* 2020, **59**, 23527–23531.

[3] L. D. M. Nicholls, M. Marx, T. Hartung, E. González-Fernández, C. Golz, M. Alcarazo, *ACS Catal.* 2018, **8**, 6079–6085.

[4] G. M. Sheldrick, *Acta Cryst.* 2008, **64**, 112–122.

[5] O. V. Dolomanov, L. J. Bourhis, R. J. Gildea, J. Howard, H. Puschmann, *J. Appl. Cryst.* 2009, **42**, 339–341.

[6] E. Sans-Panadés, J. J. Vaquero, M. A. Fernández-Rodríguez, P. García-García, *Org. Lett.* 2022, **24**, 5860–5865.

[7] R. P. Alexander, et al., Fused Thiazole Derivatives as Kinase Inhibitors, U.S. Patent 8,242,116, 14 August 2012.

[8] A. Abengózar, P. García-García, D. Sucunza, A. Pérez-Redondo, J. J. Vaquero, *Chem. Commun.* 2018, **54**, 2467–2470.

[9] K. A. Asserese, H. Huang, *Org. Lett.* 2023, ***25***, 1109–1113.

[10] A. C. Shaikh, D. S. Ranade, P. R. Rajamohanan, P. P. Kulkarni, N. T. Patil, *Angew. Chem. Int. Ed.* 2017, ***56***, 757–761.

[11] C. Körner, P. Starkov, T. D. Sheppard, *J. Am. Chem. Soc.* 2010, **132**, 5968–5969.

[12] M. Chen, N. Su, T. Deng, D. J. Wink, Y. Zhao, T. G. Driver, *Org. Lett.* 2019, **21**, 1555–1558.

[13] K. Naveen, P. T. Perumal, D.-H. Cho, *Org. Lett.* 2019, **21**, 4350–4354.

[14] A. K. Verma, R. R. Jha, R. Chaudhary, R. K. Tiwari, K. S. K. Reddy, A. Danodia, *J. Org. Chem.* 2012, **77**, 8191–8205.

[15] R. D. Mule, A. C. Shaikh, A. B. Gade, N. T. Patil, *Chem. Commun.* 2018, **54**, 11909–11912.

[16] S. Suárez-Pantiga, P. Redero, X. Aniban, M. Simon, C. Golz, R. A. Mata, M. Alcarazo, *Chem. Eur. J.* 2021, **27**, 13358–13366.

[17] A. Krasovskiy, P. Knochel, *Synthesis* 2006, 890–891.

[18] T. Tamai, K. Fujiwara, S. Higashimae, A. Nomoto, A. Ogawa, *Org. Lett.* 2016, **18**, 2114–2117.

[19] F. Kleemiss, O. V. Dolomanov, M. Bodensteiner, N. Peyerimhoff, L. Midgley, L. J. Bourhis, A. Genoni, L. A. Malaspina, D. Jayatilaka, J. L. Spencer, F. White, B. Grundkötter-Stock, S. Steinhauer, D. Lentz, H. Puschmann, S. Grabowsky, *Chem. Sci.* 2020, **12**, 1675–1692.

[20] M. J. Frisch, G. W. Trucks, H. B. Schlegel, G. E. Scuseria, M. A. Robb, J. R. Cheeseman, G. Scalmani, V. Barone, G. A. Petersson, H. Nakatsuji, X. Li, M. Caricato, A. V. Marenich, J. Bloino, B. G. Janesko, R. Gomperts, B. Mennucci, H. P. Hratchian, J. V. Ortiz, A. F. Izmaylov, J. L. Sonnenberg, D. Williams-Young, F. Ding, F. Lipparini, F. Egidi, J. Goings, B. Peng, A. Petrone, T. Henderson, D. Ranasinghe, V. G. Zakrzewski, J. Gao, N. Rega, G. Zheng, W. Liang, M. Hada, M. Ehara, K. Toyota, R. Fukuda, J. Hasegawa, M. Ishida, T. Nakajima, Y. Honda, O. Kitao, H. Nakai, T. Vreven, K. Throssell, J. A. Montgomery, Jr., J. E. Peralta, F. Ogliaro, M. J. Bearpark, J. J. Heyd, E. N. Brothers, K. N. Kudin, V. N. Staroverov, T. A. Keith, R. Kobayashi, J. Normand, K. Raghavachari, A. P. Rendell, J. C. Burant, S. S. Iyengar, J. Tomasi, M. Cossi, J. M. Millam, M. Klene, C. Adamo, R. Cammi, J. W. Ochterski, R. L. Martin, K. Morokuma, O. Farkas, J. B. Foresman, D. J. Fox, *Gaussian 16*, Revision C.01, Gaussian, Inc., Wallingford, CT, 2016.

[21] A. D. Becke, *J. Chem. Phys.* 1993, **98**, 5648–5652.

[22] F. Weigend, R. Ahlrichs, P*hys. Chem. Chem. Phys.* 2005, **7**, 3297–3305.

[23] S. Grimme, J. Antony, S. Ehrlich, H. Krieg, *J. Chem. Phys*. 2010, **132**, 154104.

[24] S. Grimme, S. Ehrlich, L. Goerigk, *J. Comput. Chem*. 2011, **32**, 1456–1465.

[25] a) V. Barone, M. Cossi, *J. Phys. Chem. A* 1998, **102**, 1995–2001. b) M. Cossi, N. Rega, G. Scalmani, V. Barone, *J. Comput. Chem.* 2003, **24**, 669–681.

[26] S. Grimme, *Chem. Eur. J.* 2012, **18**, 9955–9964.

[27] C. Bannwarth, S. Grimme, *Comput. Theor. Chem.* 2014, **1040–1041**, 45–53.

[28] F. Neese, *WIREs Comput. Mol. Sci.* 2025, **15**, e70019.

[29] T. Yanai, D. P. Tew, N. C. Handy, *Chem. Phys. Lett.* 2004, **393**, 51–57.
